# Endovascular Treatment With and Without Intravenous Thrombolysis in Large Vessel Occlusions Stroke: A Systematic Review and Meta-Analysis

**DOI:** 10.3389/fneur.2021.697478

**Published:** 2021-08-30

**Authors:** Shuo Li, Dan-Dan Liu, Guo Lu, Yun Liu, Jun-Shan Zhou, Qi-Wen Deng, Fu-Ling Yan

**Affiliations:** ^1^Department of Neurology, Affiliated ZhongDa Hospital, School of Medicine, Southeast University, Nanjing, China; ^2^Department of Neurology, Dezhou People's Hospital, Dezhou, China; ^3^Department of Neurology, Nanjing First Hospital, Nanjing Medical University, Nanjing, China; ^4^Key Laboratory of Developmental Genes and Human Disease, Southeast University, Nanjing, China

**Keywords:** acute ischemic stroke, large vessel occlusion, thrombectomy, endovascular treatment, bridging therapy

## Abstract

**Background:** Previous studies have shown conflicting results about the benefits of pretreatment with intravenous thrombolysis before endovascular treatment (EVT) in patients with acute ischemic stroke (AIS) with large vessel occlusions (LVOs). This study aimed to investigate the clinical efficacy and safety of EVT alone vs. bridging therapy (BT) in patients with AIS with LVOs.

**Methods:** A systematic review with meta-analysis of all available studies comparing clinical outcomes between BT and EVT alone was conducted by searching the National Center for Biotechnology Information/National Library of Medicine PubMed and Web of Science databases for relevant literature from database inception to October 20, 2020.

**Results:** A total of 93 studies enrolling 45,190 patients were included in the present analysis. In both unadjusted and adjusted analyses, BT was associated with a higher likelihood of 90-day good outcome (crude odds ratio [cOR] 1.361, 95% confidence interval [CI] 1.234–1.502 and adjusted OR [aOR] 1.369, 95% CI 1.217–1.540) and successful reperfusion (cOR 1.271, 95% CI 1.149–1.406 and aOR 1.267, 95% CI 1.095–1.465) and lower odds of 90-day mortality (cOR 0.619, 95% CI 0.560–0.684 and aOR 0.718, 95% CI 0.594–0.868) than EVT alone. The two groups did not differ in the occurrence of symptomatic intracranial hemorrhage (sICH) (cOR 1.062, 95% CI 0.915–1.232 and aOR 1.20, 95% CI 0.95–1.47), 24-h early recovery (cOR 1.306, 95% CI 0.906–1.881 and aOR 1.46, 95% CI 0.46–2.19), and number of thrombectomy device passes ≤ 2 (aOR 1.466, 95% CI 0.983–2.185) after sensitivity analyses and adjustment for publication bias.

**Conclusions:** BT provides more benefits than EVT alone in terms of clinical functional outcomes without compromising safety in AIS patients with LVOs.

## Introduction

Stroke is the leading cause of disability and the second major cause of death among adults worldwide, with ischemic stroke accounting for ≥80% of the cases (1, 2). The primary principle of acute ischemic stroke (AIS) treatment is recanalization and reperfusion of the occluded artery. To date, intravenous thrombolysis (IVT) is the recommended standard therapy and first-choice treatment for all eligible patients with AIS within the first 4.5 h after the onset of symptoms, irrespective of the AIS subtype (3). However, in addition to the narrow therapeutic time window and various contraindications and complications of IVT, numerous studies have reported that IVT seems less effective in AIS patients with large vessel occlusions (LVOs) (4–6).

More recently, with the development of mechanical thrombectomy (MT) devices, endovascular treatment (EVT) coupled with standard medical treatment has been demonstrated to be more beneficial than standard medical treatment alone in AIS patients with anterior circulation LVOs (6, 7). EVT, in addition to pretreatment with IVT (bridging therapy, BT), is now recommended for all eligible AIS patients with LVOs within 6 h after symptom onset based on class I level A evidence (3). However, many clinical trials and meta-analyses published from 2016 to 2020 have shown the clinical efficacy of EVT alone without pretreatment with IVT in AIS patients (8–12), giving rise to debates about the benefits of EVT alone.

Arguments in favor of BT suggest that pretreatment with IVT could improve post-MT clinical outcomes by promoting thrombus softening and fibrinolytic processes, thus increasing the likelihood of early successful recanalization (13–15). Moreover, IVT could offer the chance of reperfusion in AIS patients in whom MT failed because of the inability to reach the target occlusion. Furthermore, IVT may lead to recanalization of distal occlusions in some patients, thereby avoiding subsequent MT, or may result in the reperfusion of any remaining distal occlusions after MT (14). Conversely, pretreatment with IVT may increase the risk of bleeding complications, especially intracranial hemorrhage (ICH) (8, 16), and facilitate thrombus fragmentation, which increases the potential for migration from proximal to distal vessels (where EVT is impossible to achieve). Furthermore, pretreatment with IVT may delay the start of subsequent EVT procedures and limit additional interventions, such as antiplatelet and heparin administration (17). Additionally, an analysis performed in the United States indicated that IVT before EVT leads to significantly higher costs (18).

Considering the aforementioned uncertainties, this systematic review with meta-analysis of published studies was conducted to investigate the clinical efficacy and safety of BT vs. EVT alone in AIS patients with LVOs.

## Methods

### Data Sources and Search Strategy

The present study was performed following the Preferred Reporting Items for Systematic Reviews and Meta-analyses (PRISMA) statement guidelines (19). We conducted an electronic search of eligible studies without language restriction in the National Center for Biotechnology Information/National Library of Medicine (NCBI/NLM) PubMed and Web of Science databases using the following terms: (thrombectomy OR bridging therapy OR embolectomy OR endovascular) AND (thrombolysis OR tissue plasminogen activator OR plasminogen) AND (stroke OR brain ischemia OR cerebrovascular accident). The search covered the period from the inception of the databases to October 20, 2020. The reference lists of all retrieved articles were also manually searched to ensure maximum sensitivity and integrity of the search strategy. A reference manager (EndNote X7; Thompson Reuters, Philadelphia, PA, USA) was employed to remove duplicate references generated from the searches based on the fields “Author,” “Year,” and “Title.” The requirements for ethical approval and patient informed consent were waived for this study by the Ethics Committee of Southeast University owing to the origin of the analyzed data. Data supporting the findings of this study are available from the corresponding author on reasonable request.

### Study Selection

**Figure 1:**
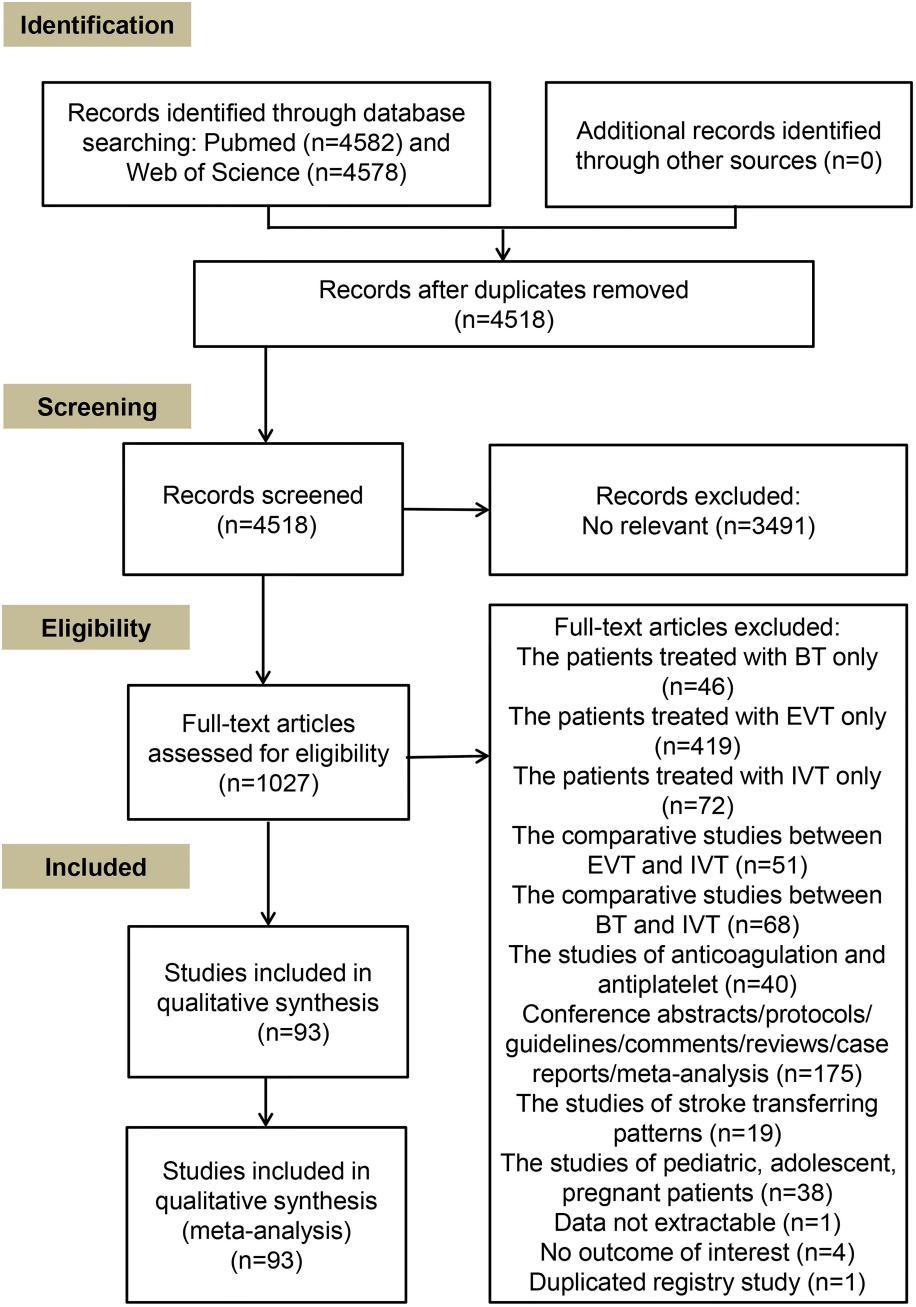
Flow chart for manuscripts selection in this meta-analysis.

The inclusion criteria for eligible studies were as follows: (1) enrolled AIS patients with LVOs; (2) compared outcomes between EVT alone and BT, or investigated the correlation between IVT and outcomes in patients undergoing EVT; and (3) reported raw data on both EVT alone and BT treatments, or odds ratios (ORs) or risk ratios (RRs) and 95% confidence intervals (CIs) for outcomes from regression analyses. The exclusion criteria were as follows: (1) lack of data on outcomes of AIS patients stratified by IVT treatment before EVT; (2) no reported data on the outcomes of interest of this study; (3) conference abstracts, study protocols, guidelines, comments, review articles, case reports, and other meta-analyses; (4) inclusion of pediatric, adolescent, or pregnant patients; and (5) un-extractable data related to outcomes. When duplicated data for each outcome of interest were reported in different studies, we included the study with the larger sample size. The detailed screening process of full-text articles is shown in Figure 1.

### Data Extraction and Quality Assessment

Two reviewers (SL and D-DL) independently extracted data from qualified studies using predefined electronic forms. The forms were selected by two of the authors (SL and GL) according to the inclusion and exclusion criteria. In case of disagreements, the full-text articles were retrieved to reach a consensus among all authors. As the enrolled studies presented different control sets, we defined “treatment with EVT alone” as the reference and “treatment with BT” as the intervention group. The following study characteristics were robustly extracted: name of the first author, publication year, registry name or data source, prospective or retrospective design, sample size, age of patients, ethnicity, National Institutes of Health Stroke Scale (NIHSS) score on admission, time from onset to groin puncture, time from groin puncture to reperfusion, time from onset to reperfusion, thrombolysis dose, EVT device, location of the occluded artery, time to follow-up, clinical outcome, statistical method, crude and adjusted results (RR, OR, 95% CI, and *p*-value) for clinical outcomes, and confounder adjustment.

The Newcastle-Ottawa Scale (NOS) was used by two of the authors (SL and Q-WD) to independently evaluate the quality of cohort studies and *post hoc* analysis of clinical trials and to investigate potential causes of bias in eligible studies. Differences in NOS scores were settled through discussion and mutual consensus.

### Clinical Outcome Evaluation

The primary efficacy outcome in this study was the degree of disability assessed using the modified Rankin Scale (mRS) at discharge and 90 days or the longest available follow-up time point, and categorized as follows: (1) excellent outcome (defined as an mRS score of 0–1), (2) good outcome (defined as an mRS score of 0–2), and (3) favorable outcome (defined as an mRS score of 0–3). The secondary efficacy outcomes included the following: (1) successful reperfusion (defined as thrombolysis in cerebral infarction [TICI]/modified TICI [mTICI]/expanded TICI [eTICI] score ≥2b or thrombolysis in myocardial infarction [TIMI] score ≥2 or their equivalents), (2) complete reperfusion (defined as TICI/mTICI/eTICI/TIMI=3 or their equivalents) after the first-line strategy or at end of the procedure or 24 h after EVT, (3) early recovery (defined as ΔNIHSS score ≥4 at least or NIHSS score 0–2 at 24 h after admission), (4) a dramatic improvement (defined as ΔNIHSS score ≥8), (5) a good improvement (defined as ΔNIHSS score ≥2 at least or NIHSS <5 at discharge or 7 days or 3 months), and (6) number of the thrombectomy device passes ≤ 2.

The primary safety outcomes were mortality during the hospital stay or at 90 days after onset or the longest available follow-up. The secondary safety outcomes were the proportions of patients with any bleeding at the longest available follow-up, including (1) intracranial hemorrhage (ICH), (2) subarachnoid hemorrhage (SAH), (3) symptomatic intracranial hemorrhage (sICH), (4) asymptomatic intracranial hemorrhage (aICH), (5) hemorrhagic transformation (HT), (6) parenchymal hematoma (PH) type1/2, and (7) hemorrhagic infarction (HI) type1/2. Complications (clot migration, groin hematoma, pneumonia, rescue therapy, vasospasm, vessel dissection, and vessel perforation) and recurrent stroke were also evaluated. The definitions of the various bleeding types and complications are summarized in Supplementary Table 1.

### Data Synthesis and Statistical Analysis

We calculated the cOR or mean difference (MD) values and 95% CIs with an ordinal logistic regression analysis (Review Manager 5.3 software package; Nordic Cochrane Centre, Cochrane Collaboration, Copenhagen, Denmark) using data from studies that did not report relevant crude RR (cRR) or crude OR (cOR) values but provided dichotomous data on the clinical outcomes of AIS patients with LVOs stratified by IVT treatment before EVT. Heterogeneity among the enrolled studies was assessed using Cochran's *Q* and Higgins *I*^2^ statistics. A random-effect model (DerSimonian-Laird) was applied to calculate summary ratios (ORs and RRs) with 95% CIs if Cochran's *Q p* < 0.10 or *I*^2^ > 50%; otherwise, a fixed-effect model (Mantel-Haenszel) was used (20, 21). In case of heterogeneity (Cochran's *Q p* < 0.10 or *I*^2^ > 50%) of any outcome across overall studies, we conducted related subgroup analysis with a random-effect model based on the ethnicity of the studied population, study type, location of the occluded artery, and timing of functional outcome assessment. Subgroups that included fewer than two individual studies were not analyzed. The sensitivity analysis was performed through the sequential elimination of each study to identify the effect of an individual study on the pooled results. Begg's funnel plots and Egger's linear regression tests, with the logarithm of RR in the *y*-axis vs. the logarithm of the standard error of RR in the *x*-axis, were used to graphically show and assess publication bias at a statistical significance level of 0.10. A “trim-and-fill” analysis was conducted to verify and adjust for publication bias if *p* < 0.1 in Egger's linear regression test.

All ratios (ORs and RRs) and corresponding 95% CIs from each study (summarized in Supplementary Table 1) were pooled and analyzed using STATA software version 11.0 (Stata Corporation, College Station, TX, USA).

## Results

### Study Selection and Characteristics

The search strategy in the NCBI/NLM PubMed and Web of Science databases yielded 4,582 and 4,578 results, respectively. A total of 1,027 articles with available full-text were assessed after removing duplicated and non-relevant articles. Thereafter, 934 articles were further excluded owing to unavailability of data use of other treatment methods, analysis of a targeted specific population, and the nature of the study (conference abstracts, descriptive and summative studies, or duplicated registry studies). Finally, 93 studies, including 6 randomized controlled trials (RCTs), met the inclusion criteria and were incorporated in the qualitative synthesis (Figure 1). The included studies enrolled 45,190 patients, and ~54% of the patients were treated with BT and showed anterior and posterior circulation involvement. The mean ± standard deviation age ranged from 57.9 ± 11.8 to 77 ± 14 years; the median (interquartile range) age ranged from 61 (55–66) to 92 (90–93) years, and the admission NIHSS score ranged from 5 to 20. The mean duration from onset to treatment ranged from within 4.5 h to within 24 h and the symptom onset to reperfusion time ranged from 144 to 415 min. The time from symptom onset to reperfusion (MD −26.57, 95% CI −61.25–8.11) (Supplementary Figure 1A) and the time from groin puncture to reperfusion (MD 0.24, 95% CI −4.20–4.67) (Supplementary Figure 1B) were similar in BT and EVT alone groups. However, the meta-analysis of 12 studies showed that the time from onset to groin puncture was shorter in patients treated with BT (MD −58.37, 95% CI −90.76 to −25.98) (Supplementary Figure 1C). The main characteristics of the studies and the reported ORs/RRs for the primary and secondary clinical outcomes are shown in Supplementary Tables 1–4.

### Study Quality Assessment

Considering that lack of appropriate adjustments for potential confounders may lead to biases in the reported risks and results, we extracted the adjusted results in 66 of the studies (Supplementary Table 1) and listed the related confounders in 61 studies (5 studies had no available data) in Supplementary Table 5. Furthermore, the cOR/cRR and adjusted OR/RR (aOR/aRR) values with corresponding 95% CIs for the clinical outcomes were, respectively, synthesized, and the pooled results are provided in Supplementary Tables 6, 7. The overall NOS score of the enrolled studies was 696/837 (83%), and each study had a score of ≥6, which is considered to indicate an overall high quality (Supplementary Table 8).

### Functional Outcomes

**Figure 2:**
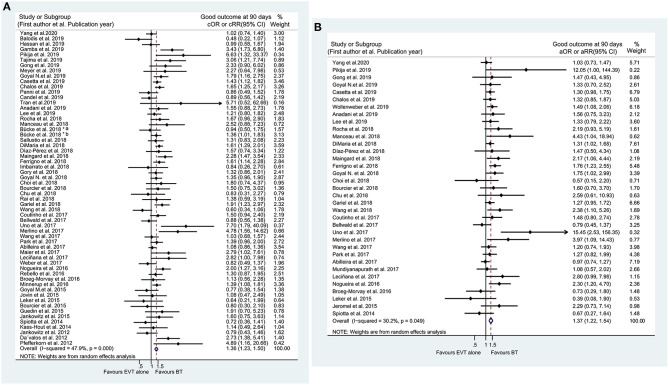
Forest plots of studies assessing good outcome at 90 days in unadjusted **(A)** and adjusted **(B)** analysis. ICA, internal carotid artery. *The acute intracranial vessel occlusion patients with (a) or without (b) concomitant ipsilateral ICA-occlusion or high-grade stenosis were investigated, respectively, in the study by Bücke et al. (22).

On the basis of the unadjusted analysis, BT was associated with a higher likelihood of a good outcome at 90 days in the meta-analysis of 58 studies (cOR 1.361, 95% CI 1.234–1.502) (Figure 2A) and a good outcome at discharge in the synthesis of 8 studies (cOR 1.691, 95% CI 1.203–2.377) than EVT alone (Supplementary Figure 2A). These associations remained significant after adjusting for potential confounders (aOR 1.369, 95% CI 1.217–1.540 at 90 days and aOR 2.032, 95% CI 1.022–4.043 at discharge) (Figure 2B and Supplementary Figure 2B). Furthermore, sequential omission of one study in the sensitivity analyses revealed a significant difference in the achievement of a good outcome at discharge between BT and EVT alone in the unadjusted analysis (cOR 1.691, 95% CI 1.10–2.78), but not in the adjusted analysis (aOR 2.032, 95% CI 0.77–7.24) (Supplementary Figure 3). Notably, the subgroup analysis revealed that BT could improve the rate of 90-day good outcome regardless of the nature of the occlusion (anterior or posterior circulation involvement) (Supplementary Figure 4). Moreover, both unadjusted and adjusted analyses showed that the rate of an excellent outcome at 90 days was higher in the BT group than in the EVT alone group (cOR 1.354, 95% CI 1.170–1.566; aOR 1.328, 95% CI 1.118–1.577) (Supplementary Figure 5). No significant effects of IVT pretreatment on excellent outcome at discharge, good outcome at 6 months or 1 year, and favorable outcome at 90 days were found (Supplementary Figures 6, 7). The detailed summaries of the subgroup analyses by study type, location of the occluded artery, and ethnicity are shown in Supplementary Tables 6, 7, and in Supplementary Figures 4, 8–19.

### Mortality

**Figure 3:**
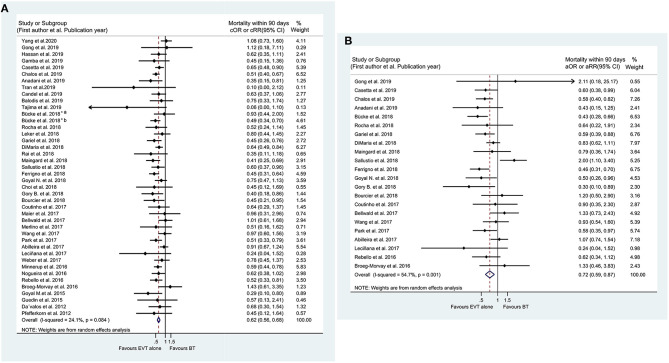
Forest plots of studies assessing mortality within 90 days in unadjusted **(A)** and adjusted **(B)** analysis. ICA, internal carotid artery. *The acute intracranial vessel occlusion patients with (a) or without (b) concomitant ipsilateral ICA-occlusion or high-grade stenosis were investigated, respectively, in the study by Bücke et al. (22).

The pooled results from 41 studies suggested that BT resulted in a lower rate of mortality within 90 days than EVT alone (cOR 0.619, 95% CI 0.560–0.684; aOR 0.718, 95% CI 0.594–0.868) (Figure 3). Similar results were also observed for in-hospital mortality (cOR 0.714, 95% CI 0.592–0.862; aOR 0.805, 95% CI 0.741–0.874) (Supplementary Figure 20). Meanwhile, the sensitivity analyses with adjusted ORs showed similar in-hospital mortality between the two groups after the exclusion of individual studies (aOR 0.80, 95% CI 0.60–1.07) (Supplementary Figure 21B). Furthermore, regardless of the location of the occluded artery, patients undergoing BT had a lower likelihood of mortality within 90 days (Supplementary Figure 22). However, in particular, similar rates of mortality within 90 days were noted between the two groups when the analyses were limited to Asian patients (cOR 0.706, 95% CI 0.451–1.106; aOR 0.742, 95% CI 0.497–1.109) (Supplementary Figure 23). Additionally, without considering the follow-up time, treatment with BT still resulted in a lower mortality rate at the longest available follow-up (cOR 0.624, 95% CI 0.566–0.688; aOR 0.742, 95% CI 0.640–0.860) (Supplementary Figure 24). The results of related stratified analyses are presented in Supplementary Tables 6, 7 and Supplementary Figures 22, 23, 25–30.

### Reperfusion

**Figure 4:**
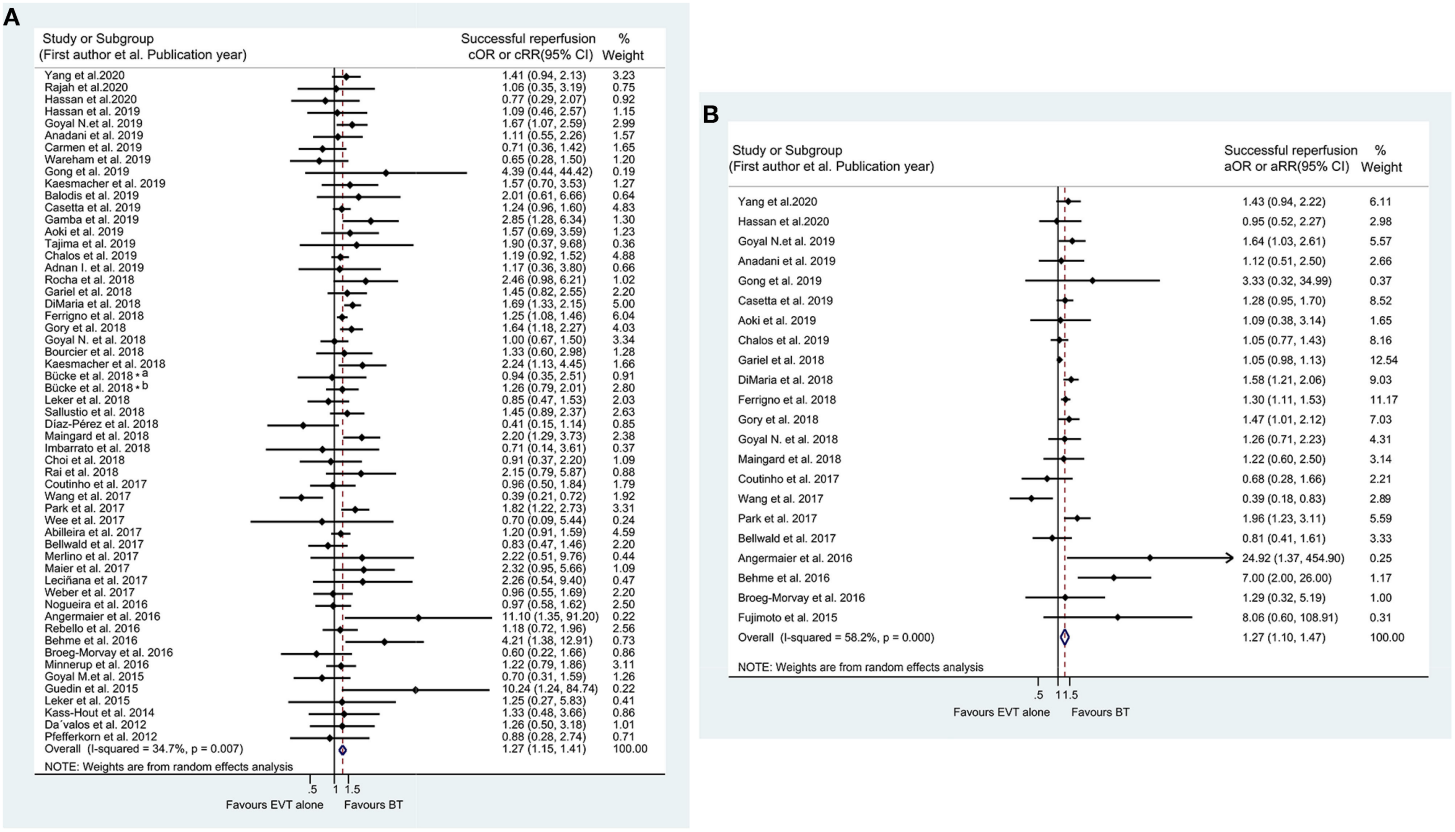
Forest plots of studies assessing successful reperfusion in unadjusted **(A)** and adjusted **(B)** analysis. ICA, internal carotid artery. *The acute intracranial vessel occlusion patients with (a) or without (b) concomitant ipsilateral ICA-occlusion or high-grade stenosis were investigated, respectively, in the study by Bücke et al. (22).

The unadjusted meta-analysis of 55 eligible studies showed a significantly higher rate of successful reperfusion in the BT group than in the EVT alone group (cOR 1.271, 95% CI 1.149–1.406) (Figure 4A), which was consistent with the result of the adjusted analysis of 22 studies (aOR 1.267, 95% CI 1.095–1.465) (Figure 4B). Particularly, subgroup analysis by the location of the occluded artery also indicated that BT could increase the rate of successful reperfusion in AIS patients with tandem occluded lesions (cOR 1.552, 95% CI 1.138–2.117) (Supplementary Figure 31). Meanwhile, the subgroup analysis involving Asian patients showed no significant difference between the two treatment groups (cOR 1. 206, 95% CI 0.731–1.989; aOR 1.178, 95% CI 0.643–2.159) (Supplementary Figure 32). Moreover, no differences between BT and EVT alone were detected in the unadjusted and adjusted analyses for complete reperfusion (cOR 1.084, 95% CI 0.947–1.241; aOR 0.988, 95% CI 0.800–1.219) (Supplementary Figure 33). The results of subgroup analyses are detailed in Supplementary Tables 6, 7, as well as in Supplementary Figures 32, 34–38.

### ICH

**Figure 5:**
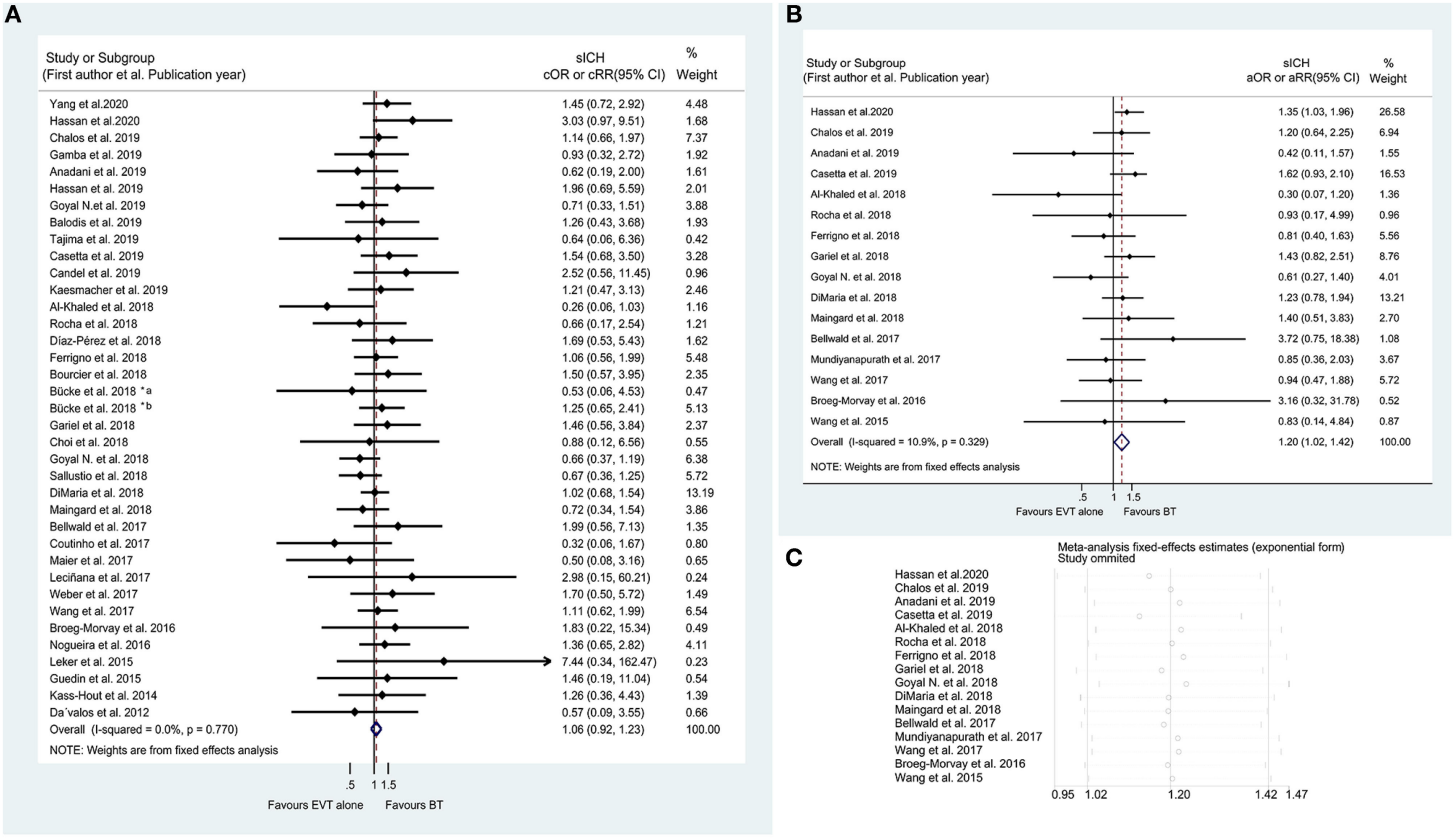
Forest plots of studies assessing sICH in unadjusted **(A)** and adjusted **(B)** analysis with related sensitivity analysis **(C)**. sICH, symptomatic intracranial hemorrhage; ICA, internal carotid artery. *The acute intracranial vessel occlusion patients with (a) or without (b) concomitant ipsilateral ICA-occlusion or high-grade stenosis were investigated respectively in the study by Bücke et al. (22).

The incidence of ICH was reported in 46 studies in the unadjusted analyses and in 20 studies in the adjusted analyses, with all studies indicating increased ICH incidence in the BT group (cOR 1.153, 95% CI 1.026–1.295; aOR 1.214, 95% CI 1.040–1.417) (Supplementary Figure 39). However, the unadjusted meta-analysis of 36 studies showed a similar incidence of sICH between the BT and EVT alone groups (cOR 1.062, 95% CI 0.915–1.232) (Figure 5A). Although BT has been considered to be associated with a higher incidence of sICH in the adjusted analysis (aOR 1.204, 95% CI 1.021–1.421) (Figure 5B), the sensitivity analysis revealed that the incidence of sICH did not significantly differ between the BT and EVT alone groups (aOR 1.204, 95% CI 0.95–1.47) (Figure 5C). Similarly, the pooled results showed that the BT group had higher rates of HT than the EVT alone group (cOR 1.152, 95% CI 1.021–1.301; aOR 1.355, 95% CI 1.014–1.811) (Supplementary Figure 40). However, the sensitivity analysis produced negative results for HT in both groups (cOR 1.15, 95% CI 0.95–1.304; aOR 1.35, 95% CI 0.95–2.05) (Supplementary Figure 41). Nevertheless, the incidence of aICH was higher in the BT group, without significant heterogeneity (*I*^2^ = 3.1%, *p* for Cochran's *Q* = 0.402 in the unadjusted analysis and *I*^2^ = 0.0%, *p* for Cochran *Q* = 0.903 in the adjusted analysis) (cOR 1.524, 95% CI 1.233–1.882; aOR 1.936, 95% CI 1.384–2.708) (Supplementary Figure 42). In addition, treatment with BT was found to be more likely to cause bleeding in any part of the body than treatment with EVT alone (aOR 1.215, 95% CI 1.040–1.420) (Supplementary Figure 43B). The results of the meta-analysis on SAH, HI, and PH (including the PH-1 and PH-2 subtypes) and those of all related subgroup analyses are presented in Supplementary Tables 6, 7 and Supplementary Figures 44–66.

### Symptom Improvement According to NIHSS Scores

**Figure 6:**
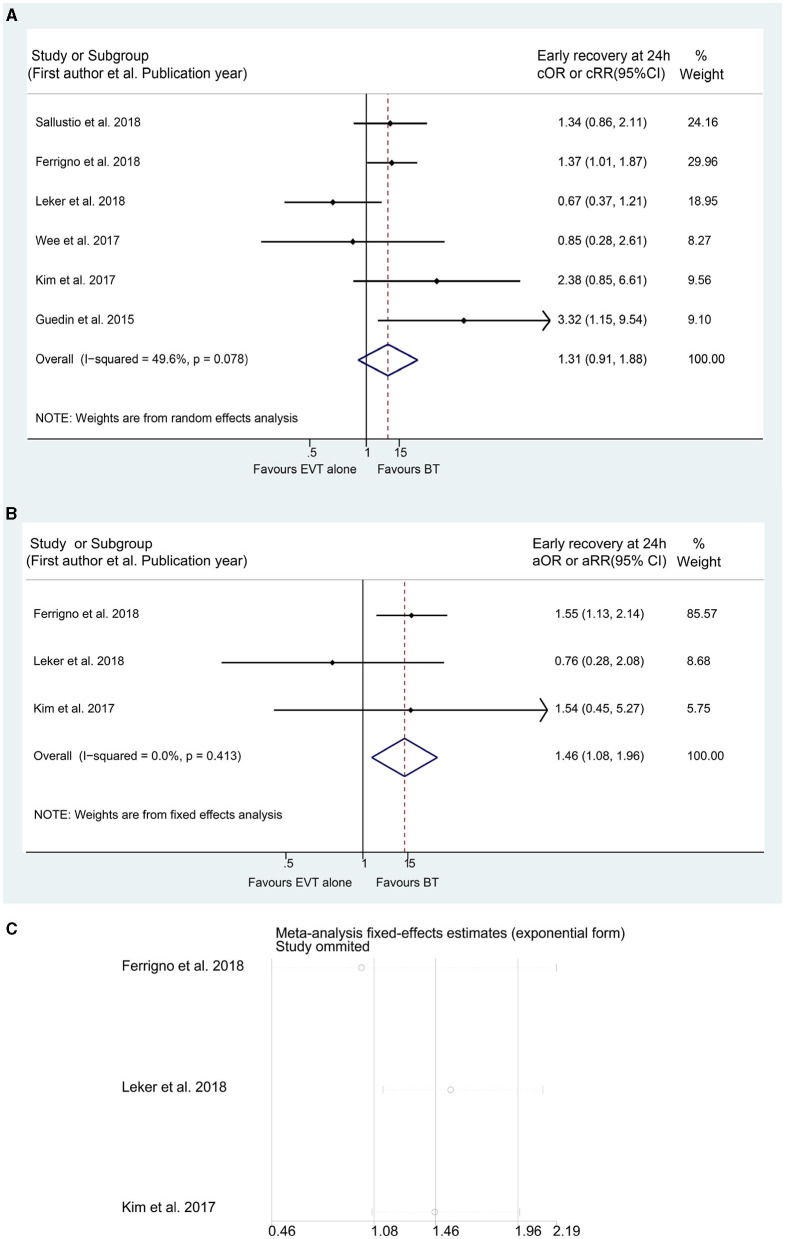
Forest plots of studies assessing early recovery at 24 h in unadjusted **(A)** and adjusted **(B)** analysis with related sensitivity analysis **(C)**.

In addition to mRS scores, NIHSS scores were also used to evaluate the prognosis with respect to the secondary efficacy outcomes. The unadjusted analysis showed that a dramatic improvement at discharge/7 days was more frequently observed in the BT group than in the EVT alone group (cOR 1.402, 95% CI 1.143–1.719) (Supplementary Figure 67), whereas no significant differences in early recovery at 24 h after admission (cOR 1.306, 95% CI 0.906–1.881) and good improvement at discharge/7 days (cOR 2.623, 95% CI 0.993–6.931) or at 3 months (cOR 1.499, 95% CI 0.866–2.595) were observed (Figure 6A and Supplementary Figure 68). Furthermore, the subgroup analysis indicated similarities between the two groups in the occurrence of a dramatic improvement at discharge/7 days in AIS patients with occluded anterior circulation (cOR 1.621, 95% CI 0.983–2.673) (Supplementary Figure 69A). For the adjusted analysis on the above outcomes, only three studies on early recovery at 24 h after admission were included in the meta-analysis. Although the adjusted results showed that BT was associated with a higher probability of early recovery at 24 h after admission (aOR 1.457, 95% CI 1.084–1.957) (Figure 6B), the sensitivity analyses suggested an instability of the pooled results (aOR 1.46, 95% CI 0.46–2.19) (Figure 6C). The detailed results of subgroup analyses are shown in Supplementary Tables 6, 7 and Supplementary Figures 70–72.

### Number of Passes of the Thrombectomy Device

**Figure 7:**
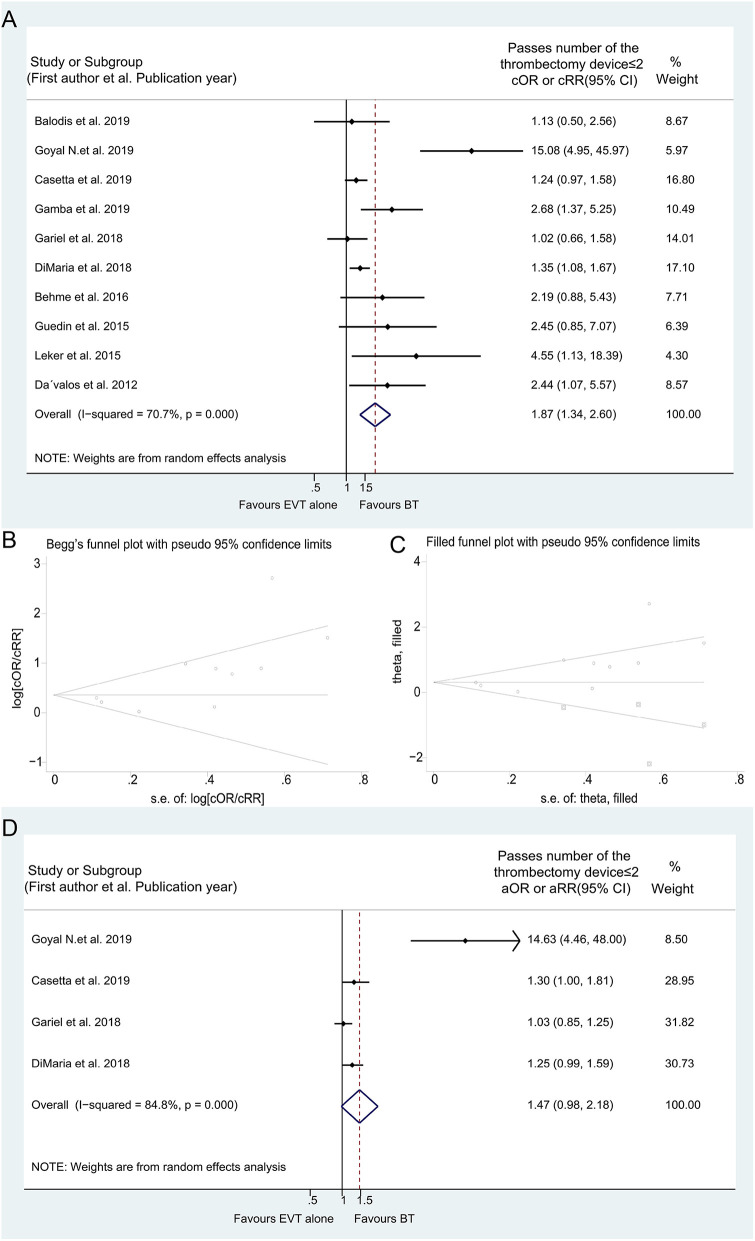
Forest plots of studies assessing number of passes of the thrombectomy device ≤ 2 in unadjusted analysis **(A)** with related Begg's funnel plots **(B)** and “Trim and fill” analysis **(C)**, and in adjusted analysis **(D)**.

A significant difference in number of thrombectomy device passes ≤ 2 was found in the unadjusted analysis, which showed that patients treated with BT required fewer passes (cOR 1.870, 95% CI 1.344–2.603) (Figure 7A). However, the result was debatable because of publication bias confirmed by Egger's linear regression test (*P*_E_ = 0.024), Begg's funnel plots (Figure 7B), and “trim-and-fill” analyses (Figure 7C and Supplementary Table 9). Moreover, this difference remained not significant in the adjusted analysis (aOR 1.466, 95% CI 0.983–2.185) (Figure 7D). Additionally, the unadjusted meta-analysis of three studies on number of thrombectomy device pass = 1 showed similar attempts during the MT procedure (cOR 1.605, 95% CI 0.926–2.781) (Supplementary Figure 73A). The results of subgroup analyses are presented in Supplementary Tables 6, 7 and Supplementary Figures 73–75.

### Complications and Recurrent Stroke

The rates of procedural complications (any complications, clot migration, groin hematoma, rescue therapy, vasospasm, vessel dissection, and vessel perforation), pneumonia, and recurrent stroke were comparable between the BT and EVT alone groups (Supplementary Tables 6, 7 and Supplementary Figures 76–95).

### Sensitivity Analysis

Sensitivity analysis was performed by sequentially omitting individual studies and evaluating the pooled results of the remaining studies. In the unadjusted analysis, sensitivity analysis data related to all outcomes except HT were consistently reported with the full pooled results, suggesting the stability and reliability of the analyses (Supplementary Figure 41). Sensitivity analyses with adjusted data produced inconsistent results for in-hospital mortality, good outcome at discharge, sICH, HT, and early recovery at 24 h in the BT group. However, no divergent trends were observed in other outcomes (Figures 5C, 6C and Supplementary Figures 3, 10, 21, 41, 96–115).

### Publication Bias

Begg's funnel plots in the unadjusted and adjusted analyses exhibited slight asymmetry, and Egger's linear regression tests confirmed the presence of publication bias in the analysis of some efficiency and safety outcomes (Supplementary Figures 116–138), involving the unadjusted analysis of aICH (*P*_E_ = 0.088), vessel dissection (*P*_E_ = 0.068), and number of thrombectomy device passes ≤ 2 (*P*_E_ = 0.024) and in the adjusted analysis of 90-day good outcome (*P*_E_ = 0.018), successful reperfusion (*P*_E_ = 0.053), number of thrombectomy device passes ≤ 2 (*P*_E_ = 0.011), any procedural complications (*P*_E_ = 0.002), and clot migration (*P*_E_ = 0.016). However, after adjustment using the “trim-and-fill” method, the test results for all outcomes remained stable except for the unadjusted analysis of number of the thrombectomy device passes ≤ 2 in the random model (Supplementary Table 9).

### RCT Analysis

**Figure 8:**
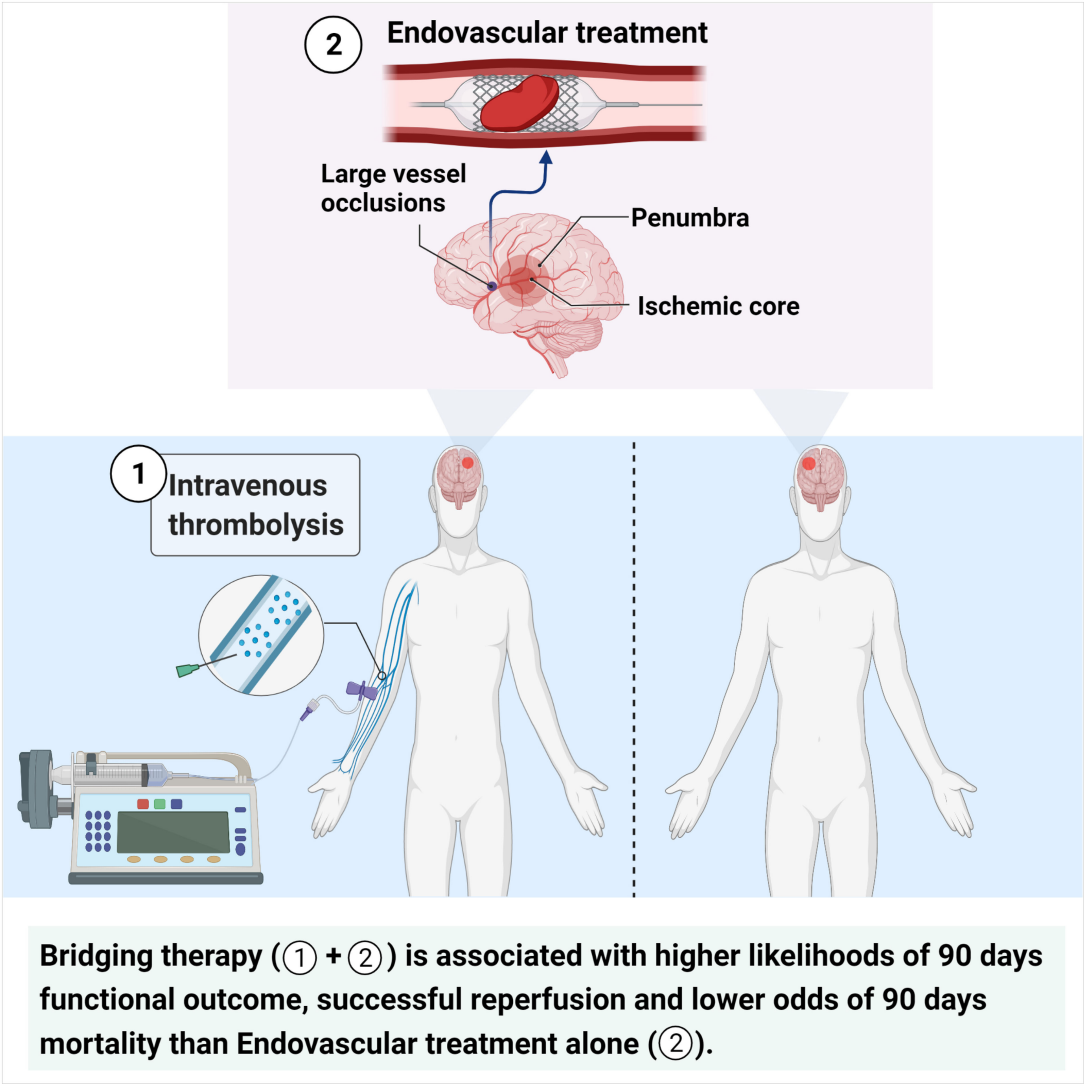
The graphical abstract showing the conclusion in the study.

Considering that RCTs could provide a higher level of evidence, we made further comparisons in the six RCT studies included in the present meta-analysis (Supplementary Table 10). The unadjusted and adjusted analyses showed a similar rate of 90-day good outcome between the BT and EVT alone groups (cOR 1.293, 95% CI 0.940–1.779; aOR 1.201, 95% CI 0.987–1.461) (Supplementary Figures 139, 140). Moreover, no significant effect of IVT pretreatment on 90- day excellent outcome was found (cOR 1.035, 95% CI 0.803–1.334; aOR 1.015, 95% CI 0.781–1.319) (Supplementary Figures 141, 142). However, BT treatment resulted in lower mortality within 90 days than EVT alone treatment (cOR 0.567, 95% CI 0.349–0.921; aOR 0.584, 95% CI 0.446–0.765) (Supplementary Figures 143, 144), consistent with the above overall results. Moreover, the unadjusted analysis revealed that BT treatment performed better than EVT alone in terms of successful reperfusion (cOR 1.228, 95% CI 1.011–1.492), whereas the adjusted analysis showed no difference between the two treatments (aOR 1.058, 95% CI 0.988–1.133) (Supplementary Figures 145, 146). In addition, a similar incidence of sICH was observed between BT and EVT alone, in keeping with the overall pooled results (cOR 1.281, 95% CI 0.864–1.899; aOR 1.323, 95% CI 0.871–2.010) (Supplementary Figures 147, 148). The graphical abstract is shown in Figure 8.

## Discussion

The present meta-analysis based on 93 studies with available full-text source suggested a potential beneficial effect of BT on 90-day functional outcomes and successful reperfusion in AIS patients with LVOs compared with EVT alone. Moreover, the rate of 90-day mortality was lower with BT, without any increase in the incidence of sICH. These benefits were consistently observed in both adjusted and unadjusted analyses. Although the adjusted analysis indicated that BT may be associated with sICH and HT, the sensitivity analysis confirmed a lack of statistical correlation after the exclusion of individual studies. Notably, IVT pretreatment did not result in a delay in the time from onset to groin puncture in EVT. The current study also showed that BT was associated with an increase in any ICH compared with EVT alone, mostly a higher incidence of aICH. Furthermore, significant differences in the occurrence of a dramatic improvement, evaluated using the NIHSS score at discharge/7days, were detected in favor of BT, but not in early recovery at 24 h after admission. In addition, the likelihood of number of thrombectomy device passes ≤ 2, procedural complications, pneumonia, and recurrent stroke was similar between BT and EVT alone.

A previous study published in 2012 by Dávalos et al. showed that compared with EVT alone, BT with Solitaire FR was associated with better functional outcomes, whereas it was not relevant to successful recanalization, sICH, and mortality (23). Meanwhile, Pfefferkorn et al. also revealed that patients treated with BT were more likely to have a better outcome than those treated with EVT alone, whereas there was no significant difference in successful recanalization and mortality (24). Furthermore, Guedin et al. disclosed that BT could facilitate successful recanalization although it was not associated with better functional outcomes, mortality, and sICH incidence in AIS patients compared with EVT alone (25). In 2018, Ferrigno et al. suggested that pretreatment with IVT tended to improve functional outcomes and successful reperfusion, as well as reduced the mortality rate without increasing the sICH incidence (26). In contrast, Kass-Hout et al. showed that the odds of good functional outcomes, successful reperfusion, lower mortality rate, and sICH were not significantly different between treatments with BT and EVT alone, consistent with the results of Abilleira et al. and Gong et al. (11, 27, 28). Moreover, according to the study by Hassan et al. in 2020, BT was not related to the likelihood of good functional outcomes, successful reperfusion, and lower mortality. However, it was associated with a higher incidence of sICH (29). Therefore, whether pretreatment with IVT could benefit AIS patients with LVOs remains unclear. The current meta-analysis indicated that BT was associated with the likelihood of good functional outcomes, successful reperfusion, and lower mortality without time delays and sICH occurrence, thus providing theoretical evidence in favor of BT.

Given the controversial results from different studies, the present meta-analysis considered and summarized the reasons for the discrepancies. First, there was a selection bias due to the treatment indication of IVT (e.g., most patients in the EVT alone group were ineligible for IVT, whereas BT was performed in patients who were eligible for IVT) (9, 10). Second, most studies comparing BT and EVT alone were observational *post hoc* analyses of clinical trials, including RCTs, that divided patients into the EVT and IVT groups (9, 30). Third, the criteria of time from onset to intervention were different for the BT and EVT alone groups, and the indication of the timing of EVT treatment also differed across various studies (e.g., within 6 or 8 h from onset to treatment) (9, 10). Finally, the discrepancies in results could also be attributed to the various MT devices and the different IVT drugs and their doses used in AIS patients in numerous studies (9, 27, 30). Larger RCTs with complete adjustments for confounding factors are needed in the future.

Recently, the DIRECT-MT (Direct Intraarterial Thrombectomy in Order to Revascularize Acute Ischemic Stroke Patients with Large Vessel Occlusion Efficiently in Chinese Tertiary Hospitals: A Multicenter Randomized Clinical Trial) study disclosed that among patients who were eligible for both intravenous alteplase treatment and EVT, EVT alone was non-inferior to BT in terms of functional outcomes (12). The results of this RCT were consistent with those of the meta-analysis by Kim et al. (31), which was also supported by the findings of a meta-analysis in tissue plasminogen activator eligible patients (32). Meanwhile, our subgroup analysis with six RCTs indicated that BT and EVT alone performed similarly in improving the functional outcomes. However, compared with EVT alone in RCTs, BT treatment resulted in lower mortality within 90 days, consistent with our overall results. Although other recent meta-analyses and the present study all showed that IVT pretreatment provided additional benefits to those of EVT in terms of clinical outcomes without evidence of safety concerns, a notable point is that these studies did not perform adjustment for various biases, especially when patients eligible and ineligible to IVT were all included in the BT group (33, 34). Therefore, the meta-analysis results should be further validated in well-designed studies in the future.

The studies included in the current meta-analysis mainly focused on anterior circulation occlusion rather than posterior circulation occlusion, and only seven studies compared BT with EVT alone in AIS patients with posterior circulation occlusion (Supplementary Table 3). According to the pooled results, BT could improve the 90-day functional outcomes and reduce 90-day mortality and the incidence of any bleeding in posterior circulation occlusion (Supplementary Figures 4, 22, 56), which indicates the benefits of BT for AIS patients with posterior circulation occlusion. Subgroup analyses by ethnicity were still conducted for the comparison of each outcome although the proportion of studies performed in an Asian population was low. The synthesized results in the adjusted analysis showed that BT was associated with 90-day functional outcomes, whereas the rates of mortality and successful reperfusion were similar in the BT and EVT alone groups in Asian patients, which differ from the results in Caucasian patients (Supplementary Figures 9, 23, 32). Furthermore, the meta-analysis with only three studies on tandem lesions showed that the rate of 90-day good outcome did not significantly differ between the two groups. However, the rate of successful reperfusion seemed to be higher in the BT group than in the EVT alone group (Supplementary Figures 10, 31). The number of studies and sample sizes in the above subgroup analyses were relatively small. Hence, more cohort studies are warranted to verify these findings.

Several limitations of the present meta-analysis should be acknowledged. First, selection bias due to IVT indication and unmeasured confounders was not considered in the current meta-analysis despite the pooling of adjusted outcome data from available studies (Supplementary Table 5). Second, a pooled subgroup analysis by thrombectomy device type and used drugs, as well as the doses of IVT drugs, was not conducted in this meta-analysis to further investigate the differences in outcomes between the two groups (although these variables are summarized in Supplementary Table 3). Third, sensitivity analyses with adjusted data showed inconsistent results for in-hospital mortality, good outcome at discharge, sICH, HT, and early recovery at 24 h, which suggested that the relevant results should be explained on the basis homogeneous studies. Nevertheless, the sensitivity analysis showed stability and reliability of the unadjusted analysis for the above outcomes in addition to HT. Fourth, the definitions of sICH were diverse among the original studies (Supplementary Table 1), which might have resulted in heterogeneous outcomes. Finally, publication biases for some unadjusted and adjusted outcomes were detected in the included studies. Nevertheless, after adjusting for publication bias, the results for all outcomes remained stable except for the unadjusted outcome of number of thrombectomy device passes ≤ 2 (Supplementary Table 9). Hence, the results from the unadjusted analysis of number of thrombectomy device passes should be interpreted with caution, and the significant association may not be true.

In conclusion, compared with EVT alone, pretreatment with IVT is associated with a higher likelihood of 90-day good and excellent functional outcomes and successful reperfusion, and lower odds of 90-day mortality in patients with AIS with LVOs. Moreover, the occurrence of sICH, 24 h early recovery, and number of thrombectomy device passes ≤ 2 did not significantly differ between the two groups. The results of this study provided evidence for the clinical choice of IVT before EVT, although further studies are needed to confirm these findings.

## Data Availability Statement

The original contributions presented in the study are included in the article/Supplementary Material, further inquiries can be directed to the corresponding author/s.

## Author Contributions

Q-WD and F-LY conceived and designed the study and advised on critically revising the manuscript and interpreting the data. SL and GL selected the extracted the qualified studies from databases. SL and D-DL extracted data with predefined electronic forms. SL, J-SZ, and Q-WD participated in synthesized data and statistical analysis. YL and J-SZ carried out the sensitivity analysis and publication bias. SL and Q-WD performed the NOS score. SL, D-DL, GL, and YL prepared figures and tables. SL drafted the manuscript. All authors have read and approved the final manuscript.

## Conflict of Interest

The authors declare that the research was conducted in the absence of any commercial or financial relationships that could be construed as a potential conflict of interest.

## Publisher's Note

All claims expressed in this article are solely those of the authors and do not necessarily represent those of their affiliated organizations, or those of the publisher, the editors and the reviewers. Any product that may be evaluated in this article, or claim that may be made by its manufacturer, is not guaranteed or endorsed by the publisher.
