# Supplementary material for: Endovascular Treatment With and Without Intravenous Thrombolysis in Large Vessel Occlusions Stroke: A Systematic Review and Meta-Analysis
Source: Front Neurol. 2021 Aug 30;12:697478. doi: 10.3389/fneur.2021.697478 (PMC8437100; doi:10.3389/fneur.2021.697478)

## SUPPLEMENTAL FIGURES

**Supplemental Figure 1.** Forest plots of studies assessing the time from symptom onset to reperfusion (A), from groin puncture to reperfusion (B), from onset to groin puncture (C)

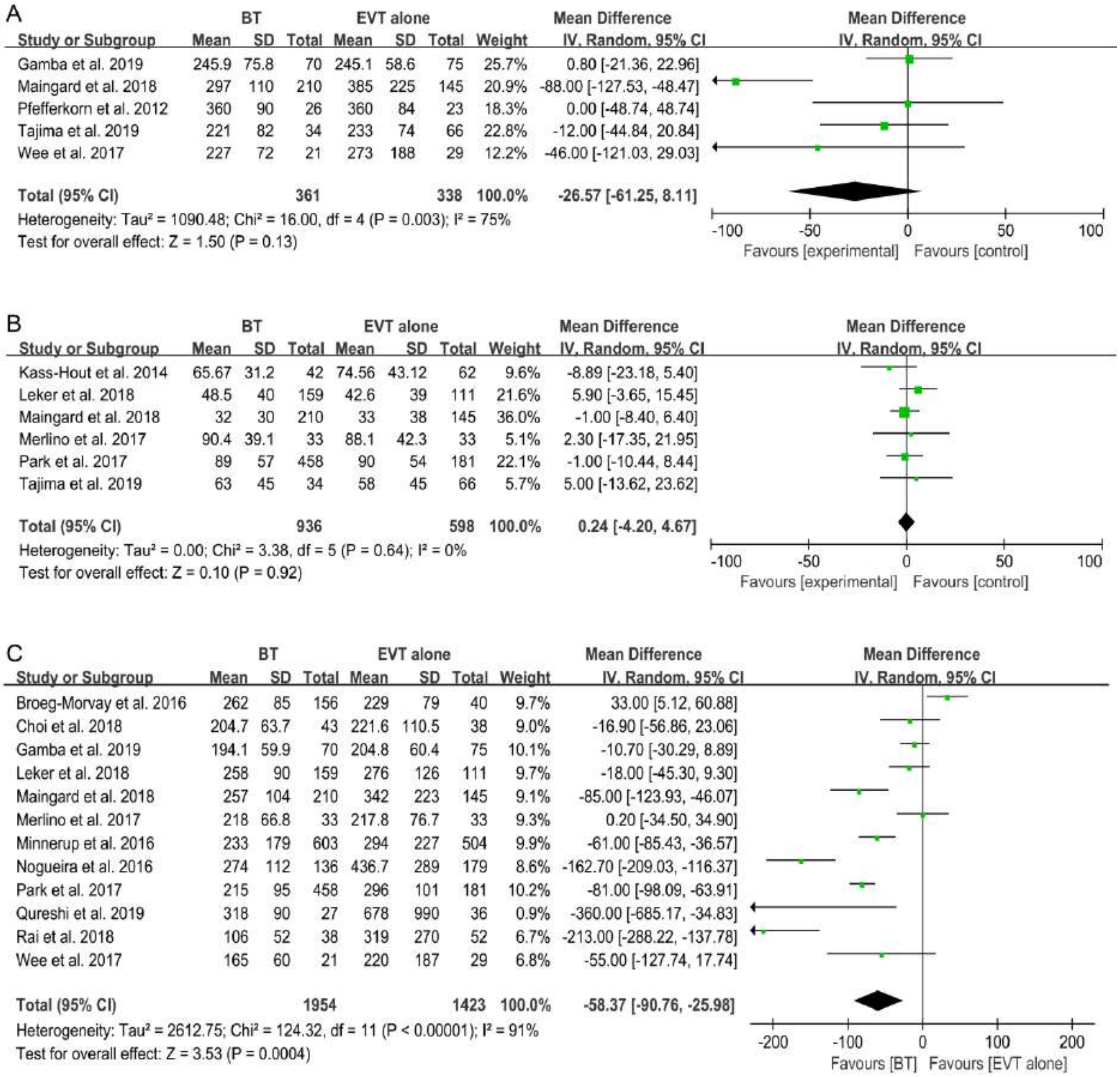

**Supplemental Figure 2.** Forest plots of studies assessing good outcome at discharge in unadjusted (A) and adjusted (B) analysis.

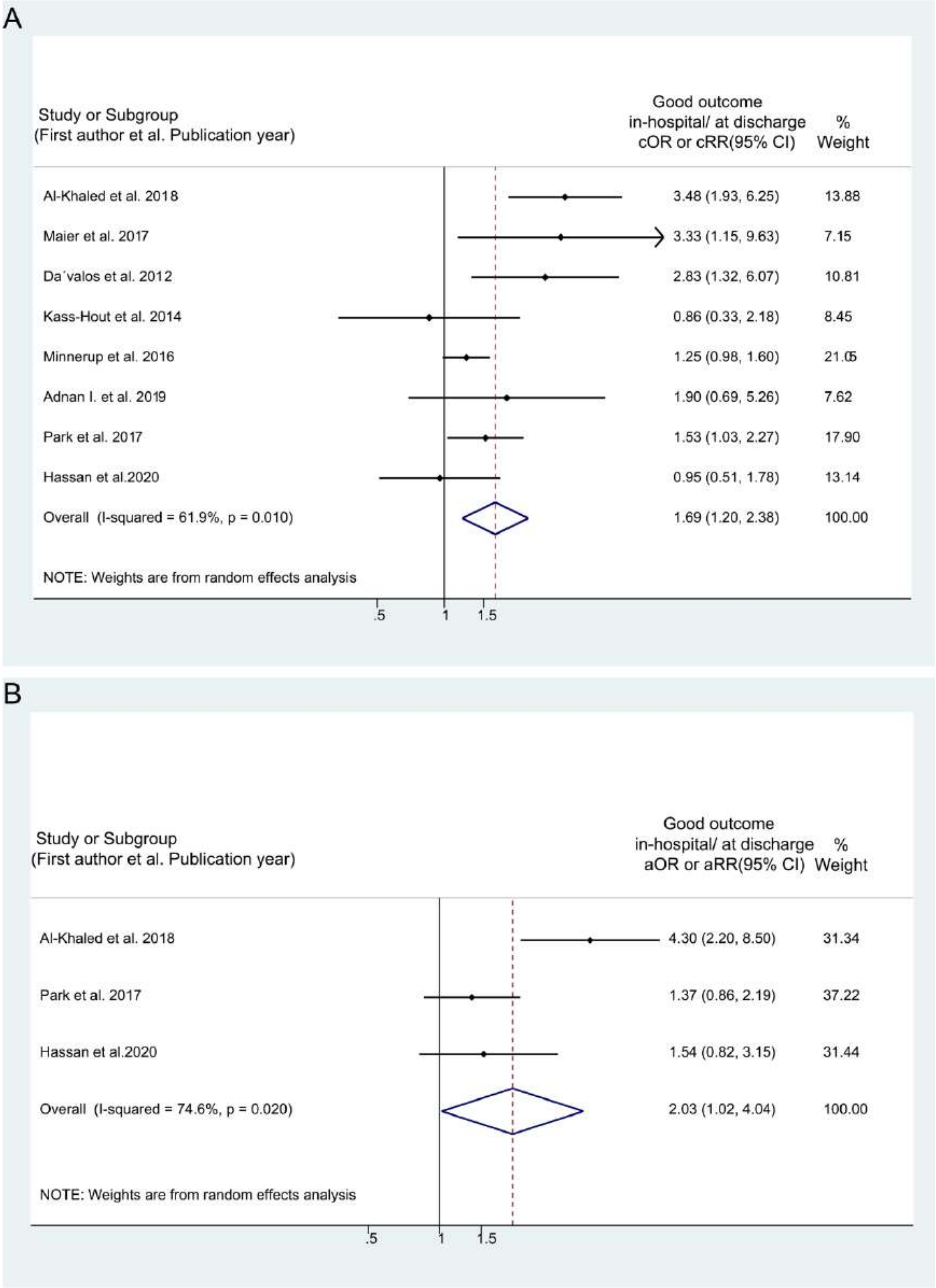

**Supplemental Figure 3.** Sensitivity analysis of the effect of individual studies on the cOR (A) and aOR (B) for good outcome at discharge. cOR, crude odds ratio; aOR, adjusted odds ratio.

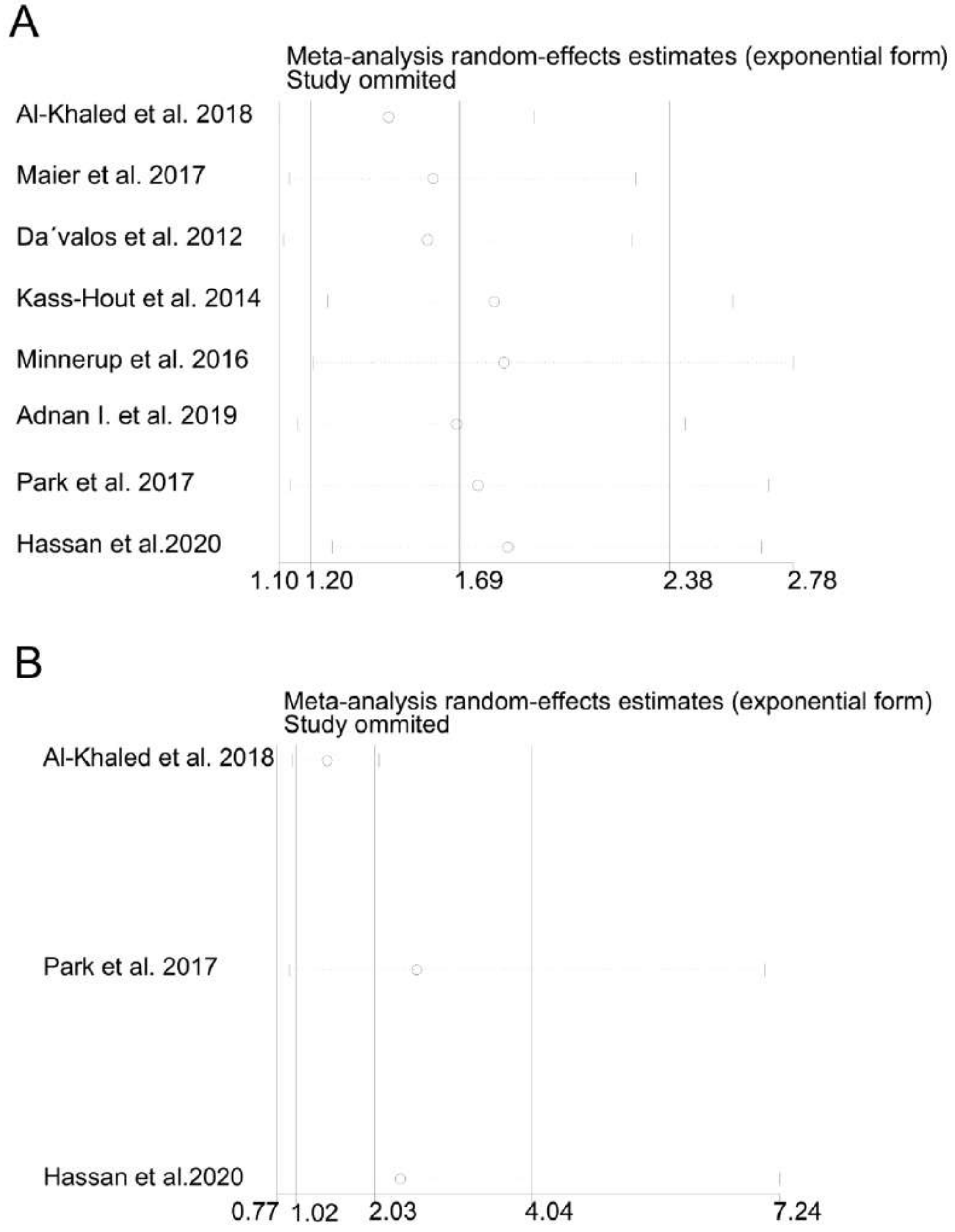

**Supplemental Figure 4.** Forest plots of subgroup analysis assessing good outcome at 90 days by location of occluded artery in unadjusted (A) and adjusted (B) analysis. ICA, internal carotid artery. [*]The acute intracranial vessel occlusion patients with (a) or without (b) concomitant ipsilateral ICA-occlusion or high-grade stenosis were investigated respectively in the study by Bücke et al. 2018.

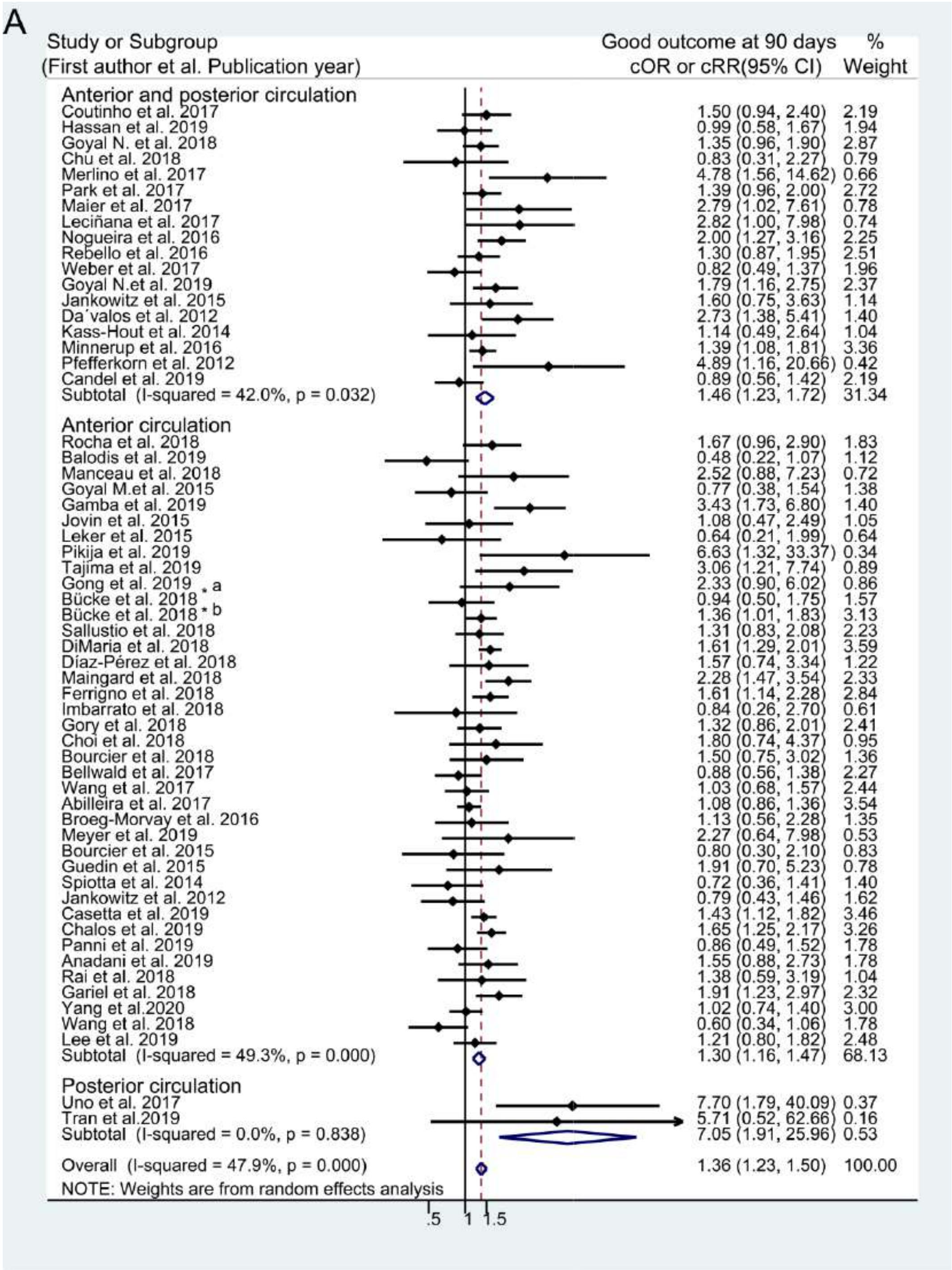

B

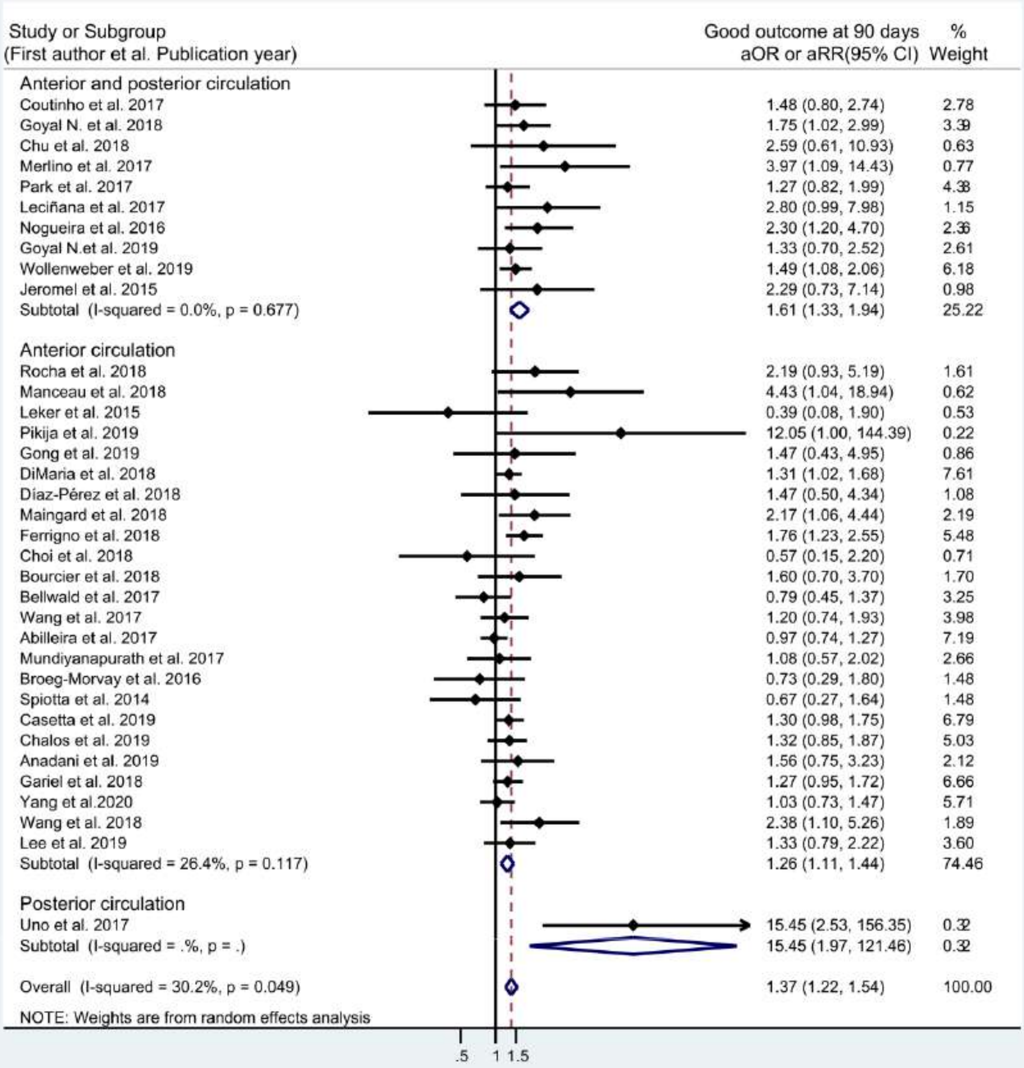
Study or Subgroup
(First author et al. Publication year)
Good outcome at 90 days
aOR or aRR(95% CI)
%
Weight
Anterior and posterior circulation
Coutinho et al. 2017 1.48 (0.80, 2.74) 2.78
Goyal N. et al. 2018 1.75 (1.02, 2.99) 3.39
Chu et al. 2018 2.59 (0.61, 10.93) 0.63
Merlino et al. 2017 3.97 (1.09, 14.43) 0.77
Park et al. 2017 1.27 (0.82, 1.99) 4.38
Leciñana et al. 2017 2.80 (0.99, 7.98) 1.15
Nogueira et al. 2016 2.30 (1.20, 4.70) 2.36
Goyal N.et al. 2019 1.33 (0.70, 2.52) 2.61
Wollenweber et al. 2019 1.49 (1.08, 2.06) 6.18
Jeromel et al. 2015 2.29 (0.73, 7.14) 0.98
Subtotal (I-squared = 0.0%, p = 0.677) 1.61 (1.33, 1.94) 25.22
Anterior circulation
Rocha et al. 2018 2.19 (0.93, 5.19) 1.61
Manceau et al. 2018 4.43 (1.04, 18.94) 0.62
Leker et al. 2015 0.39 (0.08, 1.90) 0.53
Pikija et al. 2019 12.05 (1.00, 144.39) 0.22
Gong et al. 2019 1.47 (0.43, 4.95) 0.86
DiMaria et al. 2018 1.31 (1.02, 1.68) 7.61
Díaz-Pérez et al. 2018 1.47 (0.50, 4.34) 1.08
Maingard et al. 2018 2.17 (1.06, 4.44) 2.19
Ferrigno et al. 2018 1.76 (1.23, 2.55) 5.48
Choi et al. 2018 0.57 (0.15, 2.20) 0.71
Bourcier et al. 2018 1.60 (0.70, 3.70) 1.70
Bellwald et al. 2017 0.79 (0.45, 1.37) 3.25
Wang et al. 2017 1.20 (0.74, 1.93) 3.98
Abilleira et al. 2017 0.97 (0.74, 1.27) 7.19
Mundiyanapurath et al. 2017 1.08 (0.57, 2.02) 2.66
Broeg-Morvay et al. 2016 0.73 (0.29, 1.80) 1.48
Spiotta et al. 2014 0.67 (0.27, 1.64) 1.48
Casetta et al. 2019 1.30 (0.98, 1.75) 6.79
Chalos et al. 2019 1.32 (0.85, 1.87) 5.03
Anadani et al. 2019 1.56 (0.75, 3.23) 2.12
Gariel et al. 2018 1.27 (0.95, 1.72) 6.66
Yang et al.2020 1.03 (0.73, 1.47) 5.71
Wang et al. 2018 2.38 (1.10, 5.26) 1.89
Lee et al. 2019 1.33 (0.79, 2.22) 3.60
Subtotal (I-squared = 26.4%, p = 0.117) 1.26 (1.11, 1.44) 74.46
Posterior circulation
Uno et al. 2017 15.45 (2.53, 156.35) 0.32
Subtotal (I-squared = .%, p = .) 15.45 (1.97, 121.46) 0.32
Overall (I-squared = 30.2%, p = 0.049) 1.37 (1.22, 1.54) 100.00
NOTE: Weights are from random effects analysis
.5
1
1.5

**Supplemental Figure 5.** Forest plots of studies assessing excellent outcome at 90 days in unadjusted (A) and adjusted (B) analysis.

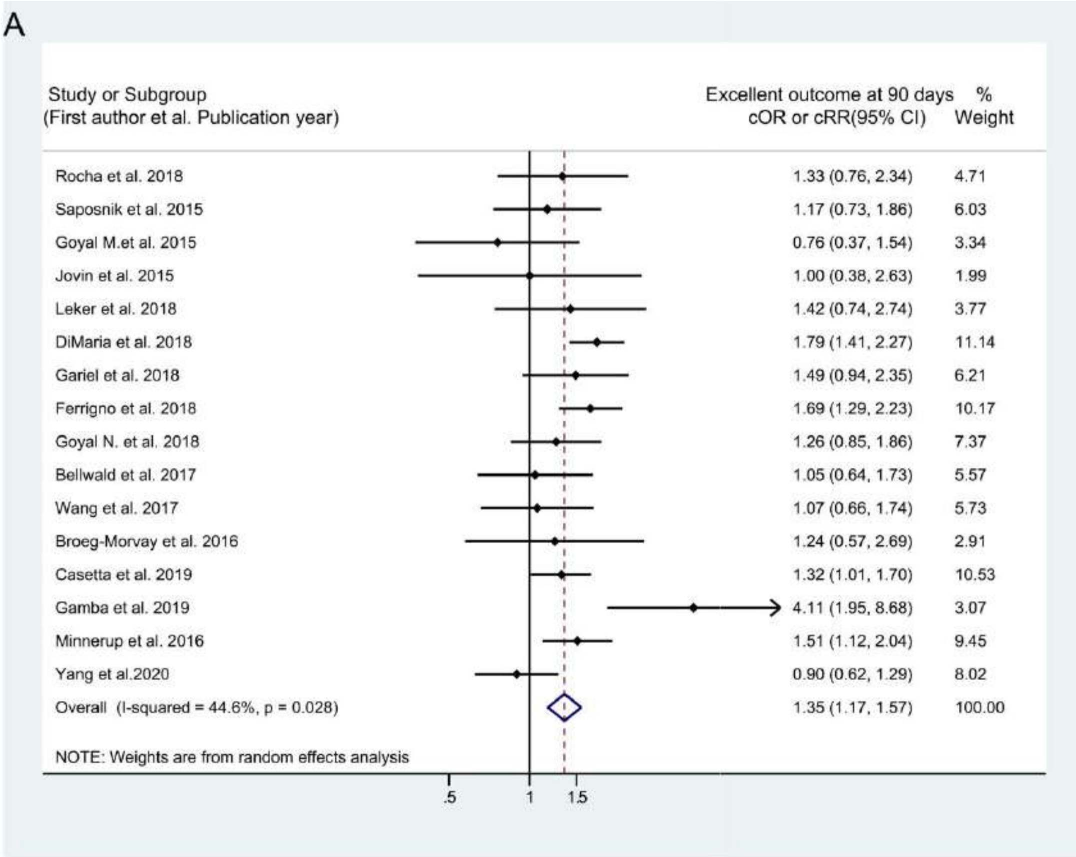


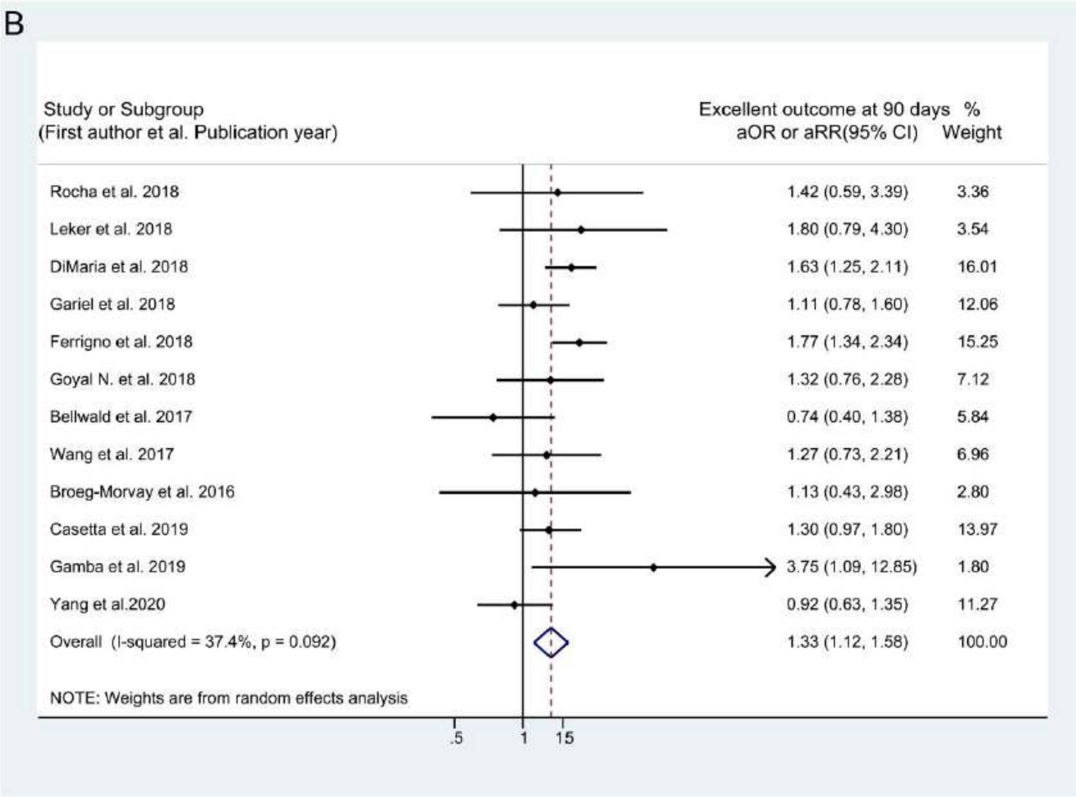

**Supplemental Figure 6.** Forest plots of studies assessing excellent outcome at discharge (A) and good outcome at 6 months or 1 year (B) in unadjusted analysis.

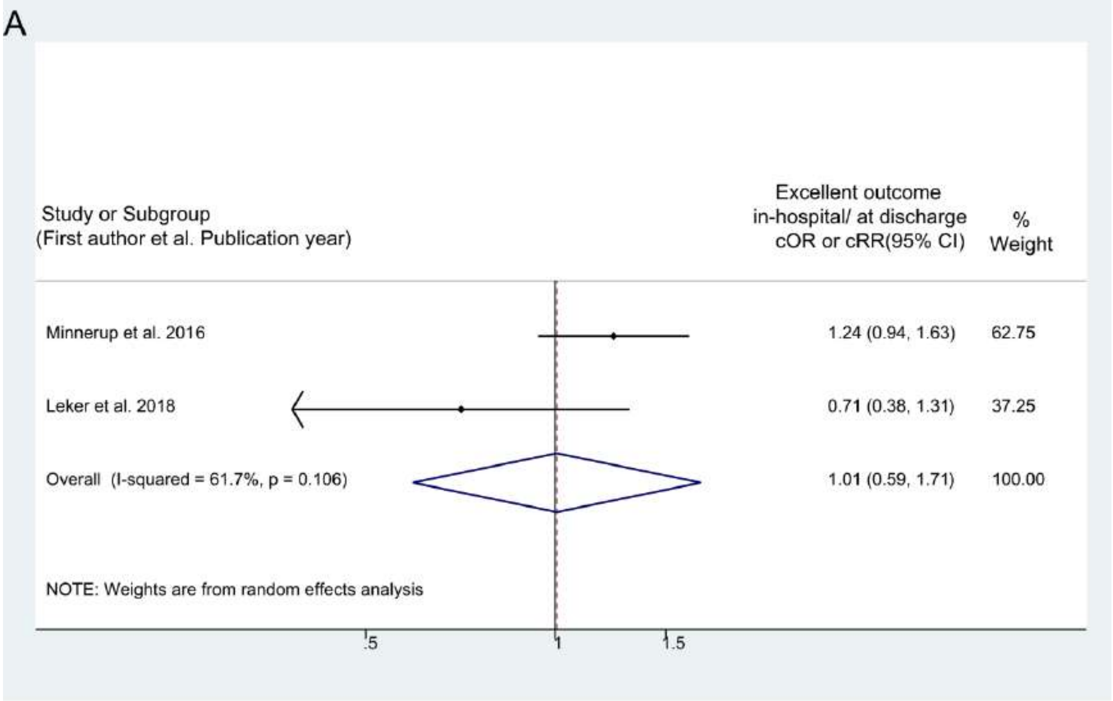


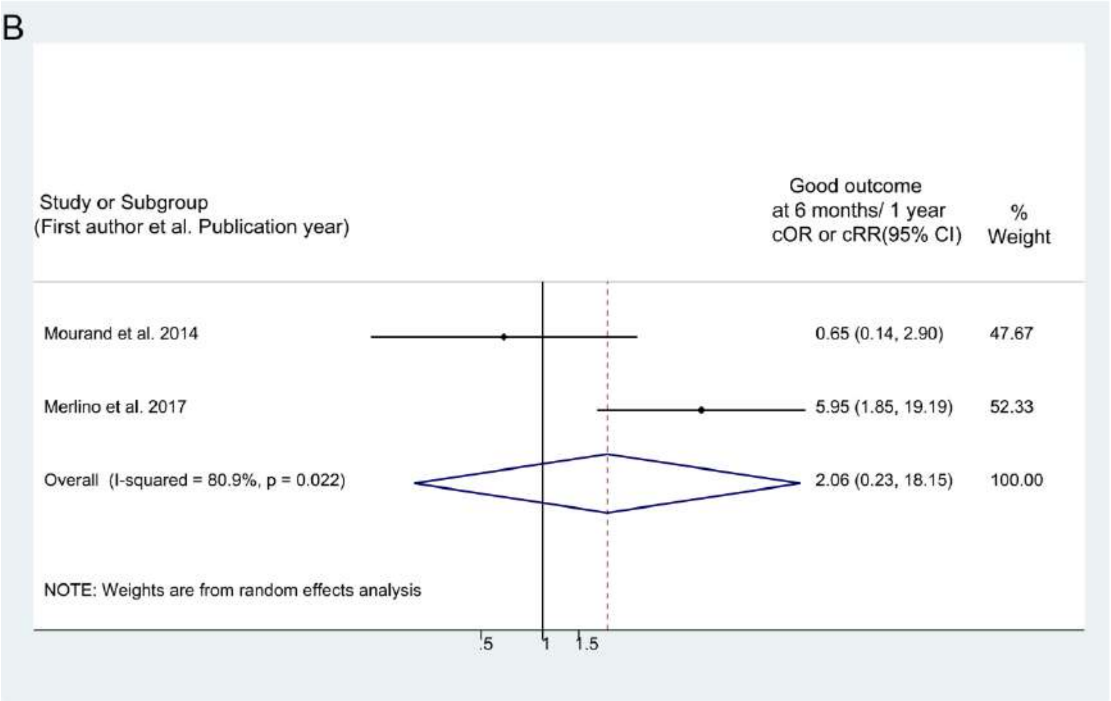

**Supplemental Figure 7.** Forest plots of studies assessing favorable outcome at 90 days in unadjusted analysis (A) and adjusted analysis (B).

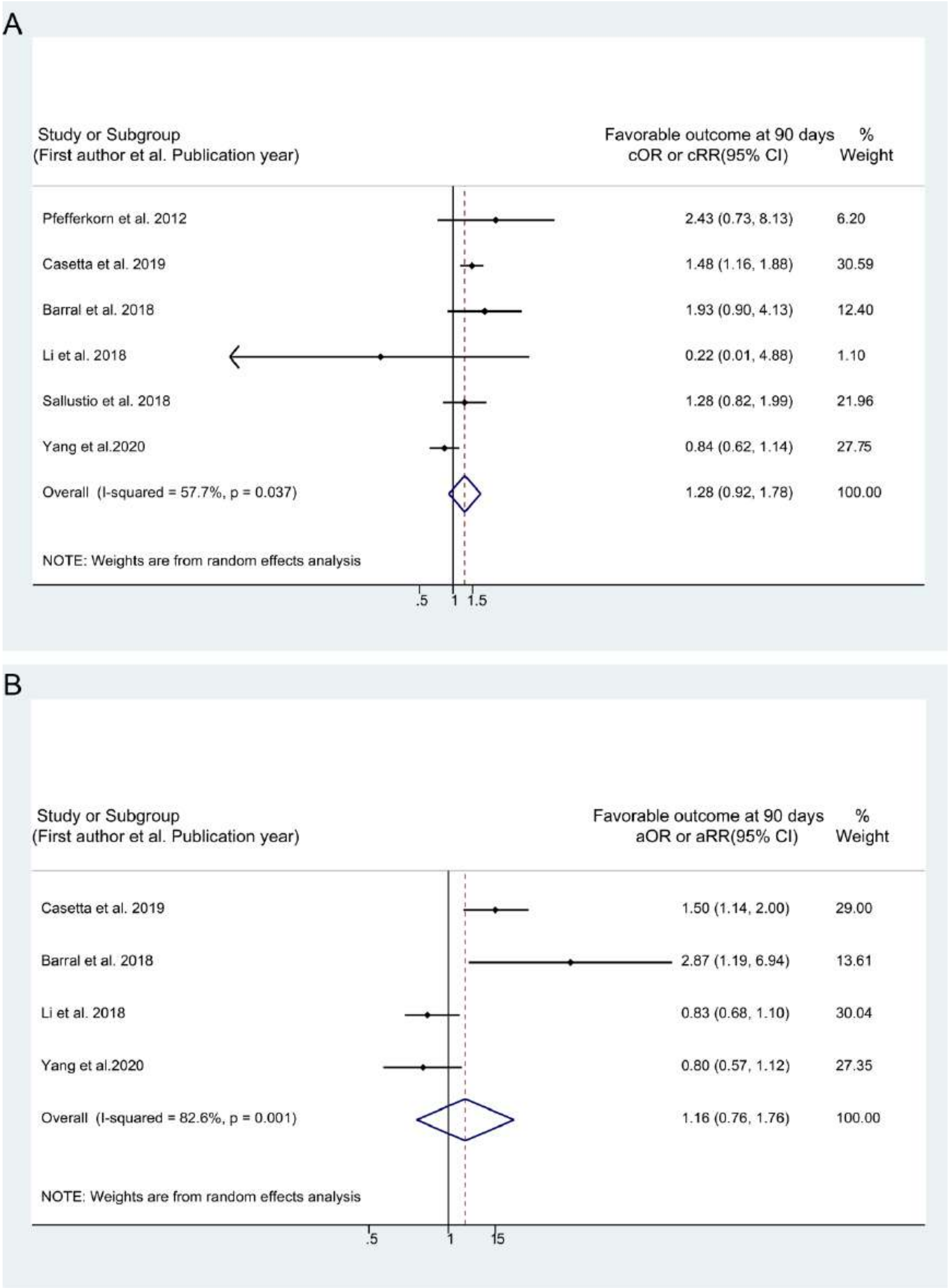

**Supplemental Figure 8.** Forest plots of subgroup analysis assessing good outcome at 90 days by study type in unadjusted (A) and adjusted (B) analysis. ICA, internal carotid artery. [*]The acute intracranial vessel occlusion patients with (a) or without (b) concomitant ipsilateral ICA-occlusion or high-grade stenosis were investigated respectively in the study by Bücke et al. 2018.

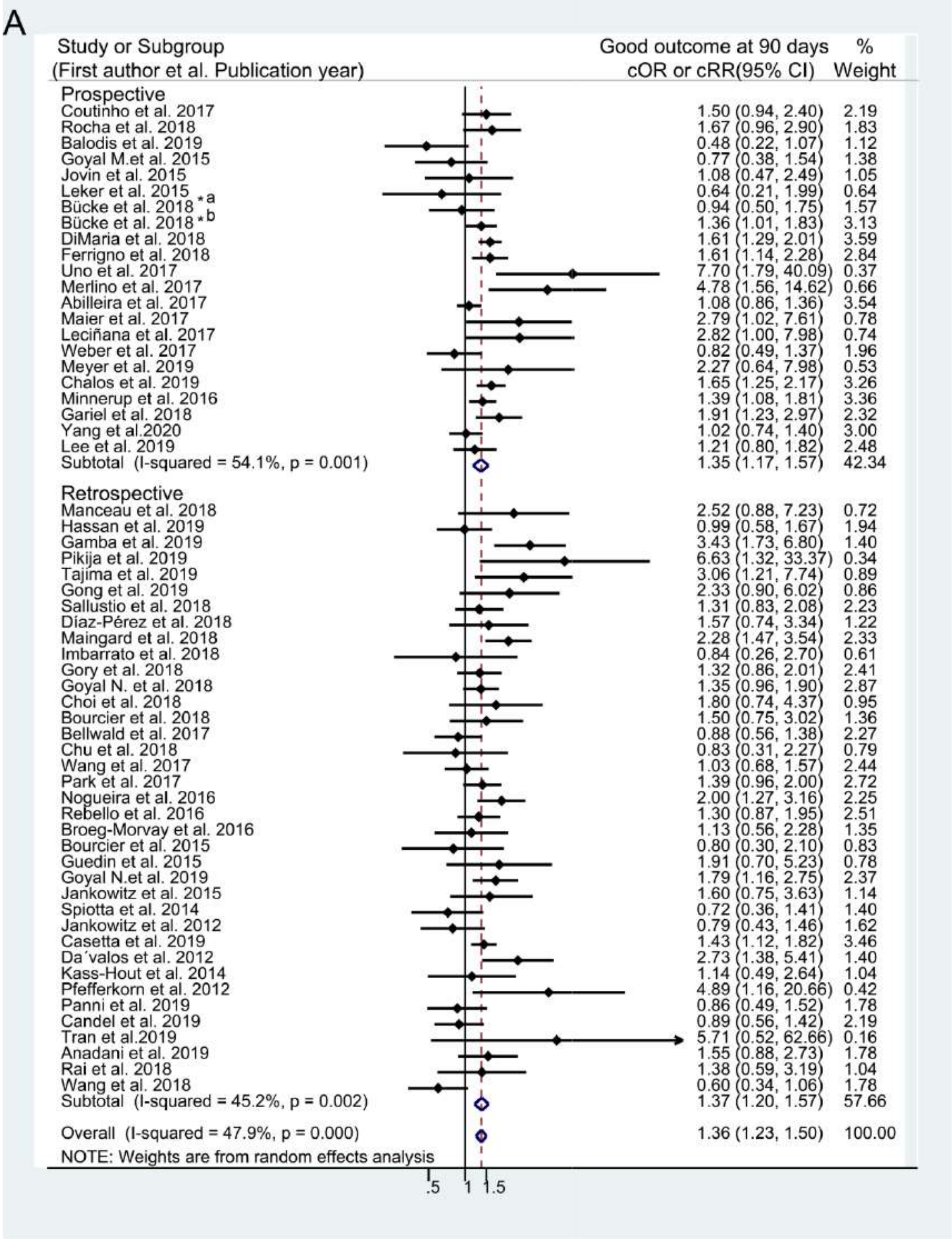

B

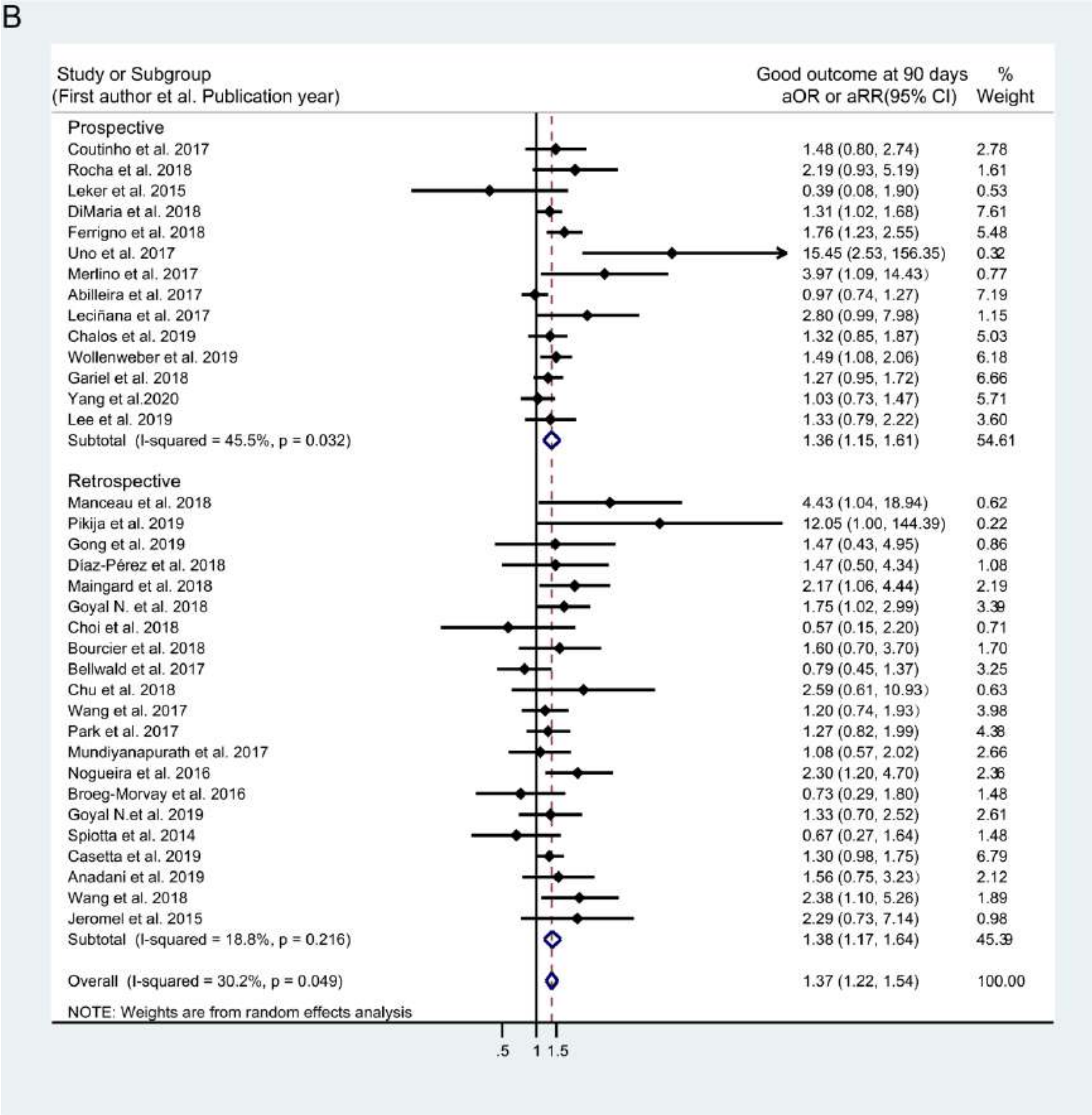

Study or Subgroup
(First author et al. Publication year)
Good outcome at 90 days
aOR or aRR(95% CI)
%
Weight
Prospective
Coutinho et al. 2017 1.48 (0.80, 2.74) 2.78
Rocha et al. 2018 2.19 (0.93, 5.19) 1.61
Leker et al. 2015 0.39 (0.08, 1.90) 0.53
DiMaria et al. 2018 1.31 (1.02, 1.68) 7.61
Ferrigno et al. 2018 1.76 (1.23, 2.55) 5.48
Uno et al. 2017 15.45 (2.53, 156.35) 0.32
Merlino et al. 2017 3.97 (1.09, 14.43) 0.77
Abilleira et al. 2017 0.97 (0.74, 1.27) 7.19
Leciñana et al. 2017 2.80 (0.99, 7.98) 1.15
Chalos et al. 2019 1.32 (0.85, 1.87) 5.03
Wollenweber et al. 2019 1.49 (1.08, 2.06) 6.18
Gariel et al. 2018 1.27 (0.95, 1.72) 6.66
Yang et al.2020 1.03 (0.73, 1.47) 5.71
Lee et al. 2019 1.33 (0.79, 2.22) 3.60
Subtotal (I-squared = 45.5%, p = 0.032) 1.36 (1.15, 1.61) 54.61
Retrospective
Manceau et al. 2018 4.43 (1.04, 18.94) 0.62
Pikija et al. 2019 12.05 (1.00, 144.39) 0.22
Gong et al. 2019 1.47 (0.43, 4.95) 0.86
Díaz-Pérez et al. 2018 1.47 (0.50, 4.34) 1.08
Maingard et al. 2018 2.17 (1.06, 4.44) 2.19
Goyal N. et al. 2018 1.75 (1.02, 2.99) 3.39
Choi et al. 2018 0.57 (0.15, 2.20) 0.71
Bourcier et al. 2018 1.60 (0.70, 3.70) 1.70
Bellwald et al. 2017 0.79 (0.45, 1.37) 3.25
Chu et al. 2018 2.59 (0.61, 10.93) 0.63
Wang et al. 2017 1.20 (0.74, 1.93) 3.98
Park et al. 2017 1.27 (0.82, 1.99) 4.38
Mundiyanapurath et al. 2017 1.08 (0.57, 2.02) 2.66
Nogueira et al. 2016 2.30 (1.20, 4.70) 2.36
Broeg-Morvay et al. 2016 0.73 (0.29, 1.80) 1.48
Goyal N.et al. 2019 1.33 (0.70, 2.52) 2.61
Spiotta et al. 2014 0.67 (0.27, 1.64) 1.48
Casetta et al. 2019 1.30 (0.98, 1.75) 6.79
Anadani et al. 2019 1.56 (0.75, 3.23) 2.12
Wang et al. 2018 2.38 (1.10, 5.26) 1.89
Jeromel et al. 2015 2.29 (0.73, 7.14) 0.98
Subtotal (I-squared = 18.8%, p = 0.216) 1.38 (1.17, 1.64) 45.39
Overall (I-squared = 30.2%, p = 0.049) 1.37 (1.22, 1.54) 100.00
NOTE: Weights are from random effects analysis
.5
1
1.5

**Supplemental Figure 9.** Forest plots of subgroup analysis assessing good outcome at 90 days by ethnicity in unadjusted (A) and adjusted (B) analysis. ICA, internal carotid artery. *The acute intracranial vessel occlusion patients with (a) or without (b) concomitant ipsilateral ICA-occlusion or high-grade stenosis were investigated respectively in the study by Bücke et al. 2018.

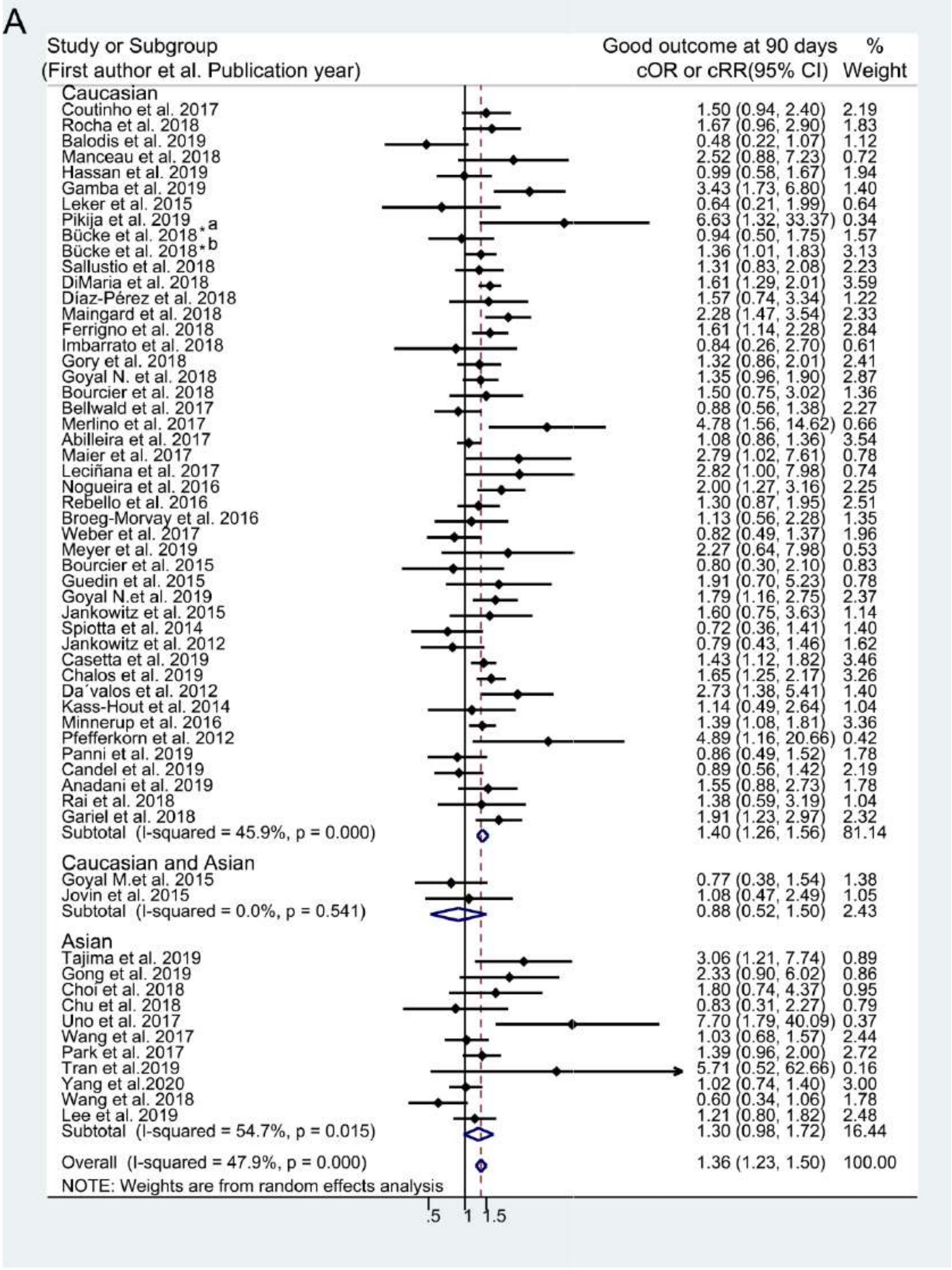

B

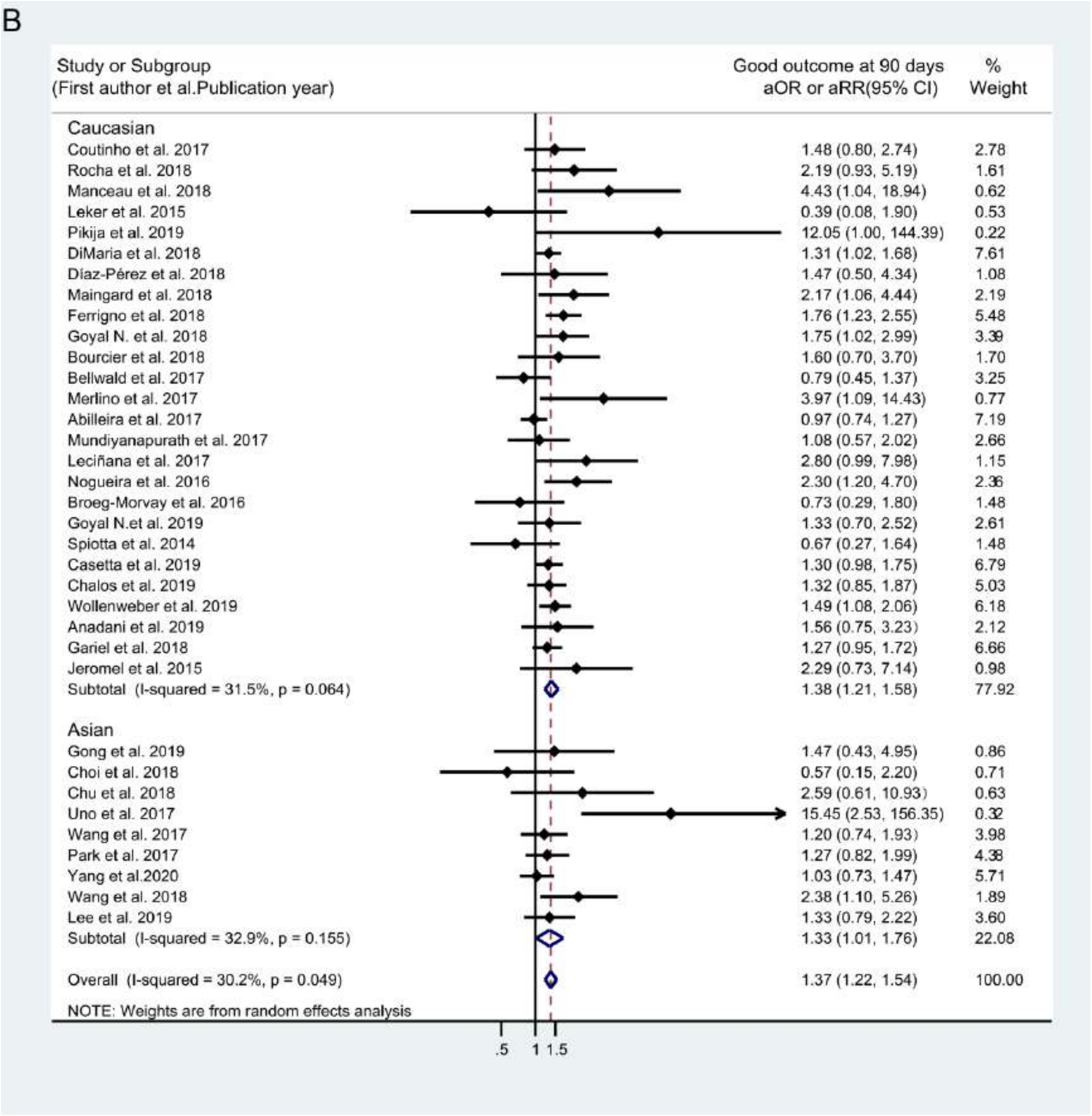
Study or Subgroup
(First author et al.Publication year)
Good outcome at 90 days
aOR or aRR(95% CI)
%
Weight
Caucasian
Coutinho et al. 2017 1.48 (0.80, 2.74) 2.78
Rocha et al. 2018 2.19 (0.93, 5.19) 1.61
Manceau et al. 2018 4.43 (1.04, 18.94) 0.62
Leker et al. 2015 0.39 (0.08, 1.90) 0.53
Pikija et al. 2019 12.05 (1.00, 144.39) 0.22
DiMaria et al. 2018 1.31 (1.02, 1.68) 7.61
Díaz-Pérez et al. 2018 1.47 (0.50, 4.34) 1.08
Maingard et al. 2018 2.17 (1.06, 4.44) 2.19
Ferrigno et al. 2018 1.76 (1.23, 2.55) 5.48
Goyal N. et al. 2018 1.75 (1.02, 2.99) 3.39
Bourcier et al. 2018 1.60 (0.70, 3.70) 1.70
Bellwald et al. 2017 0.79 (0.45, 1.37) 3.25
Merlino et al. 2017 3.97 (1.09, 14.43) 0.77
Abilleira et al. 2017 0.97 (0.74, 1.27) 7.19
Mundiyanapurath et al. 2017 1.08 (0.57, 2.02) 2.66
Leciñana et al. 2017 2.80 (0.99, 7.98) 1.15
Nogueira et al. 2016 2.30 (1.20, 4.70) 2.36
Broeg-Morvay et al. 2016 0.73 (0.29, 1.80) 1.48
Goyal N.et al. 2019 1.33 (0.70, 2.52) 2.61
Spiotta et al. 2014 0.67 (0.27, 1.64) 1.48
Casetta et al. 2019 1.30 (0.98, 1.75) 6.79
Chalos et al. 2019 1.32 (0.85, 1.87) 5.03
Wollenweber et al. 2019 1.49 (1.08, 2.06) 6.18
Anadani et al. 2019 1.56 (0.75, 3.23) 2.12
Gariel et al. 2018 1.27 (0.95, 1.72) 6.66
Jeromel et al. 2015 2.29 (0.73, 7.14) 0.98
Subtotal (I-squared = 31.5%, p = 0.064) 1.38 (1.21, 1.58) 77.92
Asian
Gong et al. 2019 1.47 (0.43, 4.95) 0.86
Choi et al. 2018 0.57 (0.15, 2.20) 0.71
Chu et al. 2018 2.59 (0.61, 10.93) 0.63
Uno et al. 2017 15.45 (2.53, 156.35) 0.32
Wang et al. 2017 1.20 (0.74, 1.93) 3.98
Park et al. 2017 1.27 (0.82, 1.99) 4.38
Yang et al.2020 1.03 (0.73, 1.47) 5.71
Wang et al. 2018 2.38 (1.10, 5.26) 1.89
Lee et al. 2019 1.33 (0.79, 2.22) 3.60
Subtotal (I-squared = 32.9%, p = 0.155) 1.33 (1.01, 1.76) 22.08
Overall (I-squared = 30.2%, p = 0.049) 1.37 (1.22, 1.54) 100.00
NOTE: Weights are from random effects analysis
.5
1
1.5

**Supplemental Figure 10.** Forest plots of studies assessing good outcome at 90 days in patients with tandem lesion (A), relevant analysis of sensitivity (B) and publication bias (C).

A

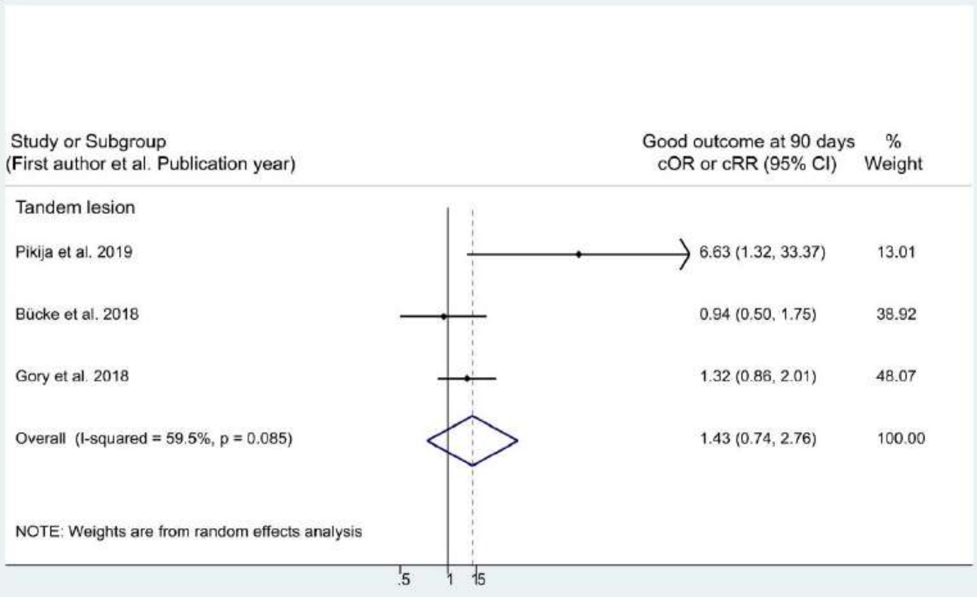


B

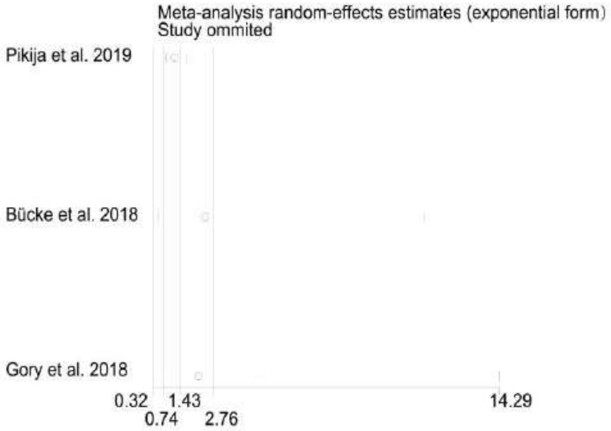


C

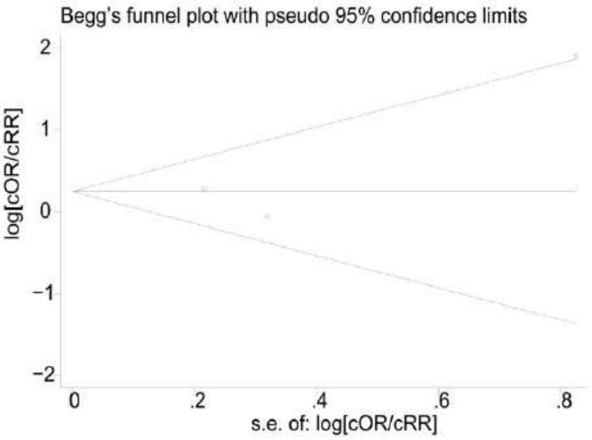

**Supplemental Figure 11.** Forest plots of subgroup analysis assessing good outcome at discharge by location of occluded artery in unadjusted (A) and adjusted (B) analysis.

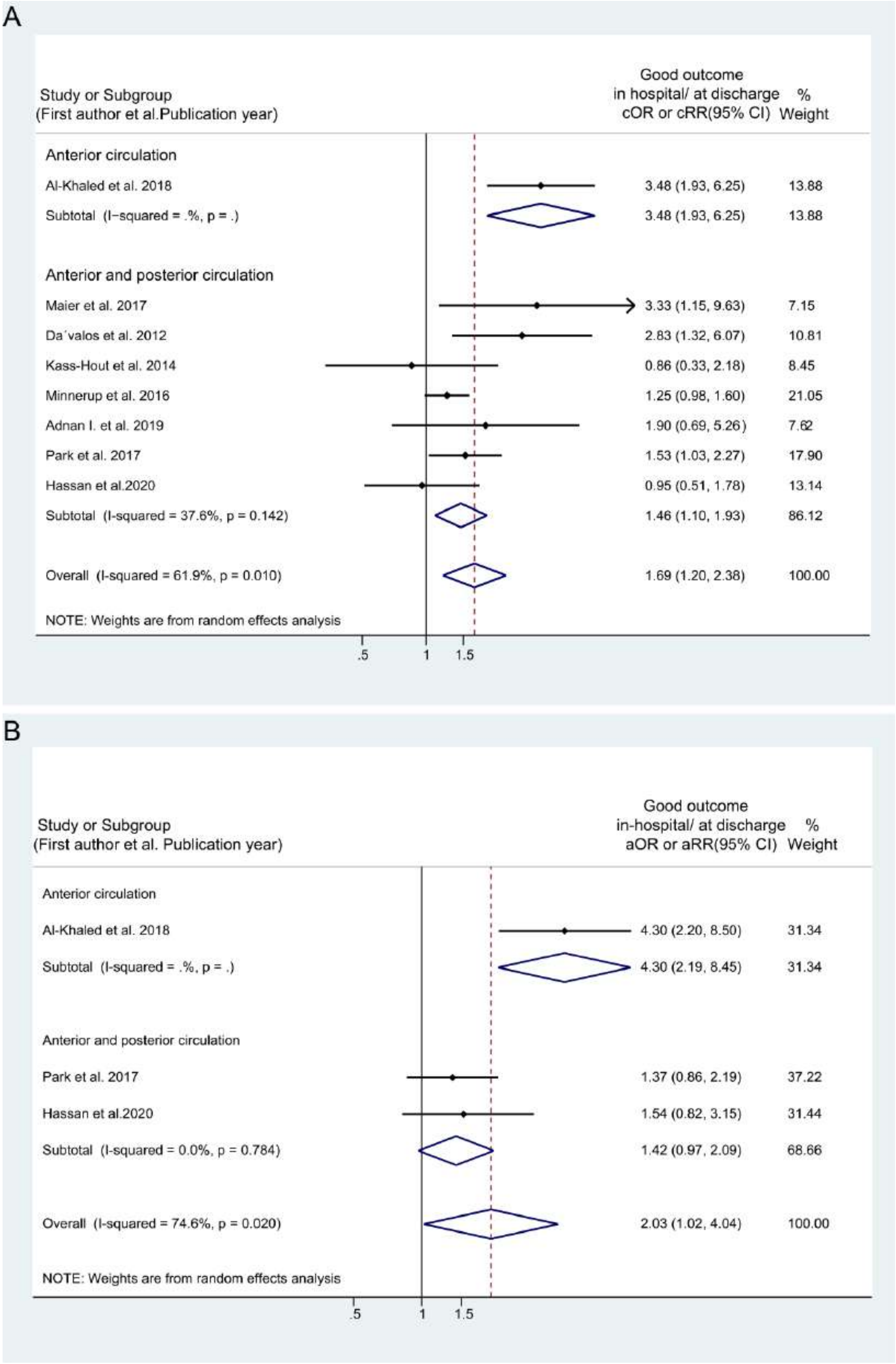

**Supplemental Figure 12.** Forest plots of subgroup analysis assessing good outcome at discharge by study type in unadjusted (A) and adjusted (B) analysis.

A

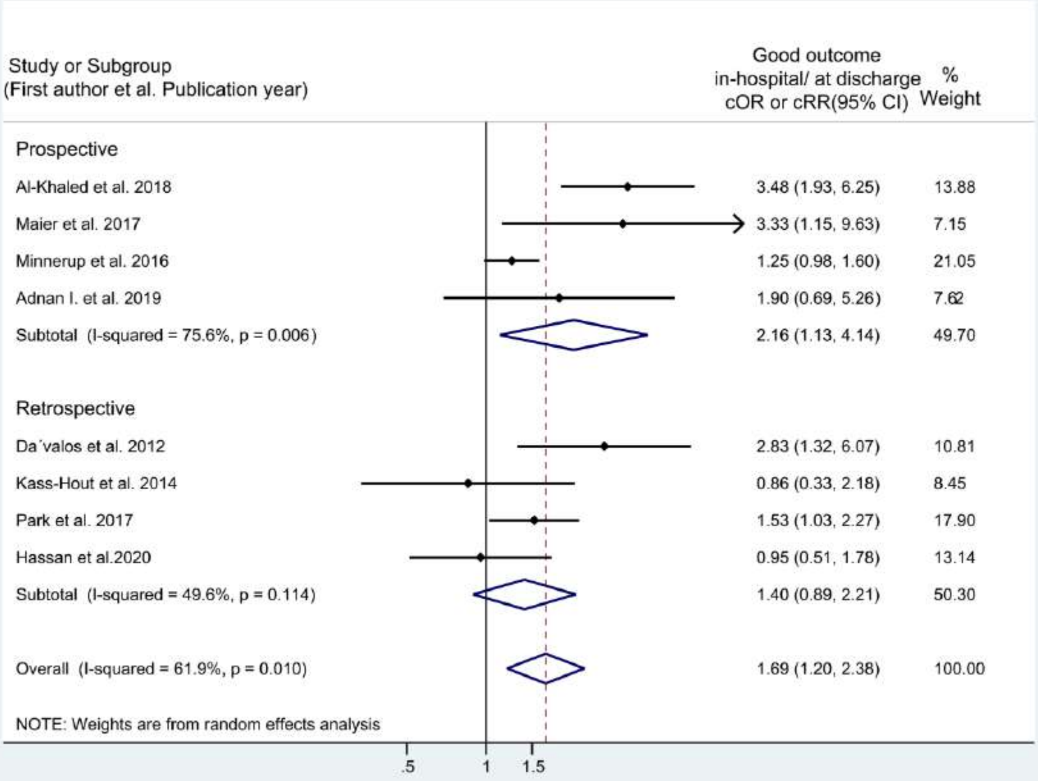


B

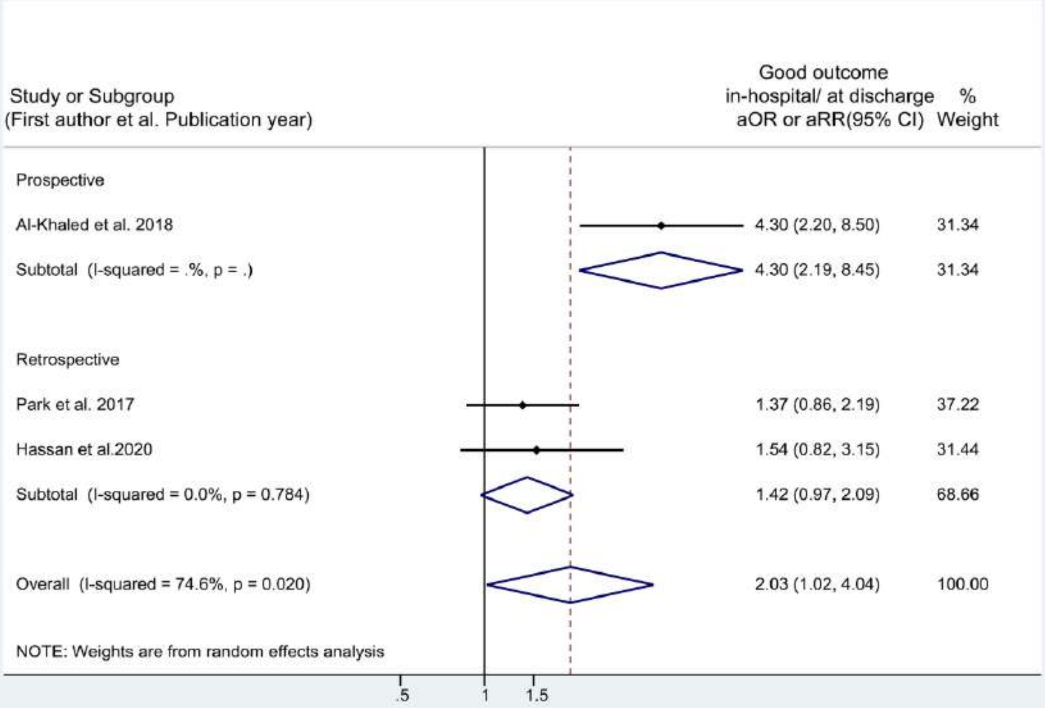

**Supplemental Figure 13.** Forest plots of subgroup analysis assessing good outcome at discharge by ethnicity in unadjusted (A) and adjusted (B) analysis.

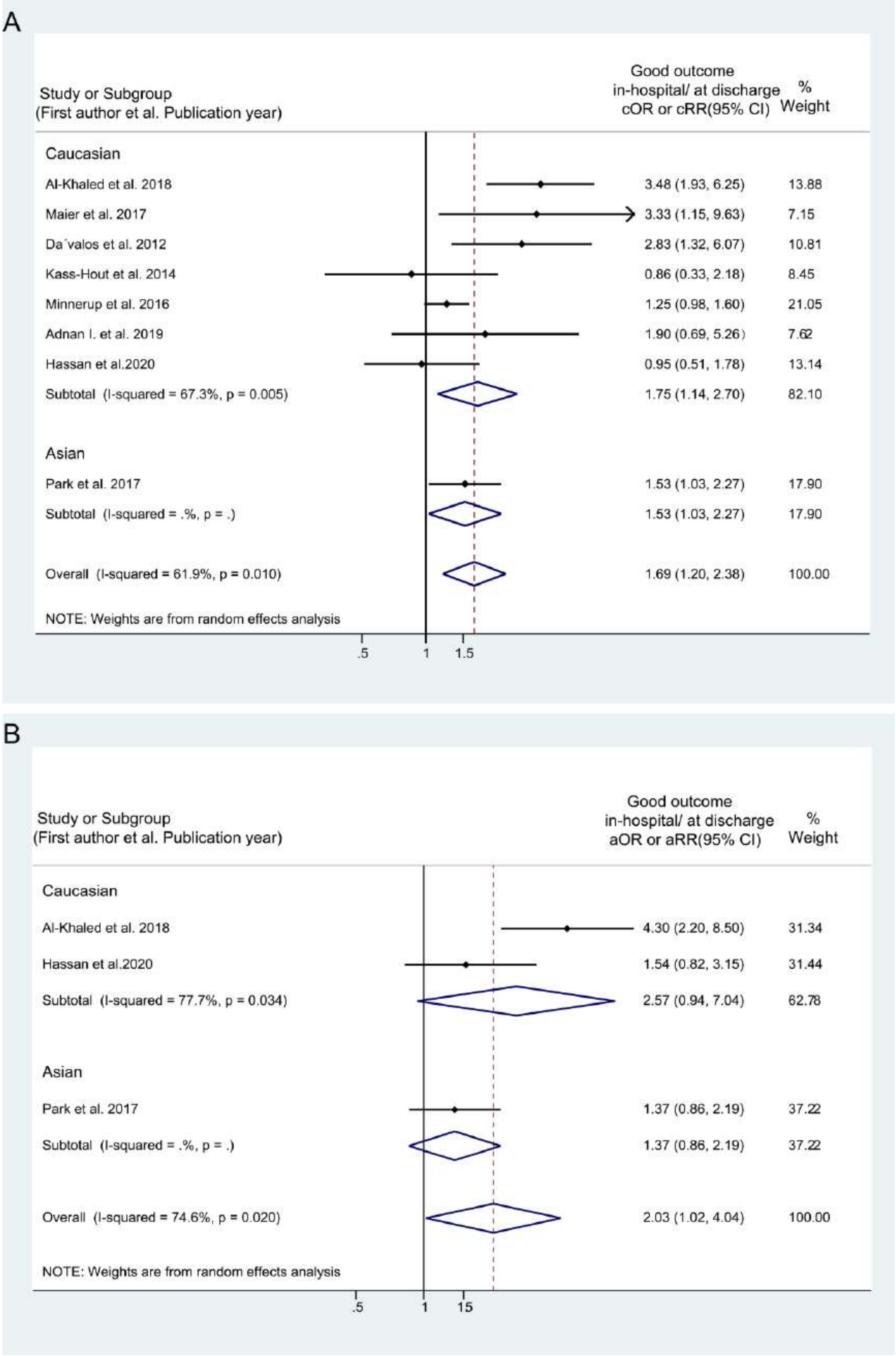

**Supplemental Figure 14.** Forest plots of subgroup analysis assessing excellent outcome at 90 days by location of occluded artery in unadjusted (A) and adjusted (B) analysis.

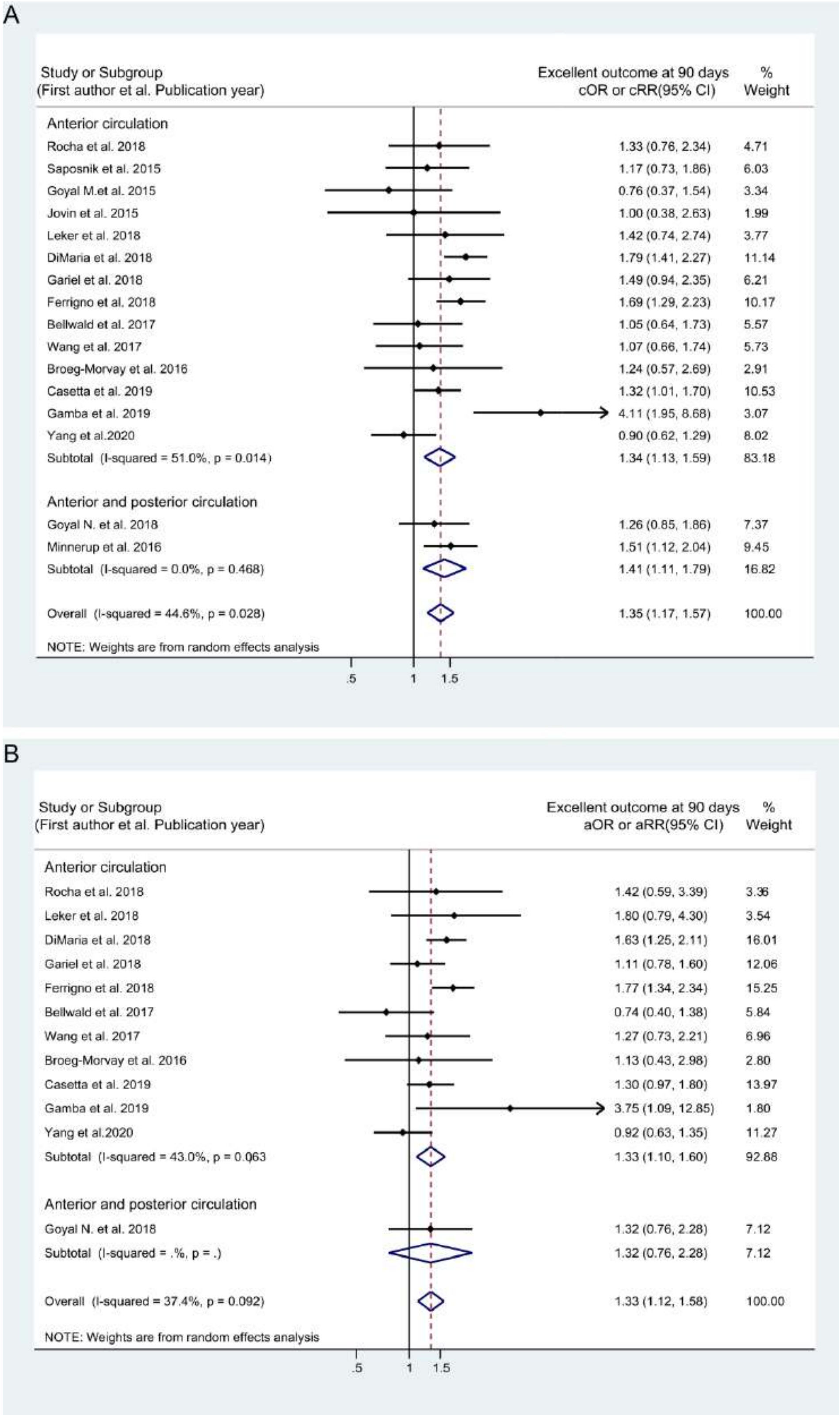

**Supplemental Figure 15.** Forest plots of subgroup analysis assessing excellent outcome at 90 days by study type in unadjusted (A) and adjusted (B) analysis.

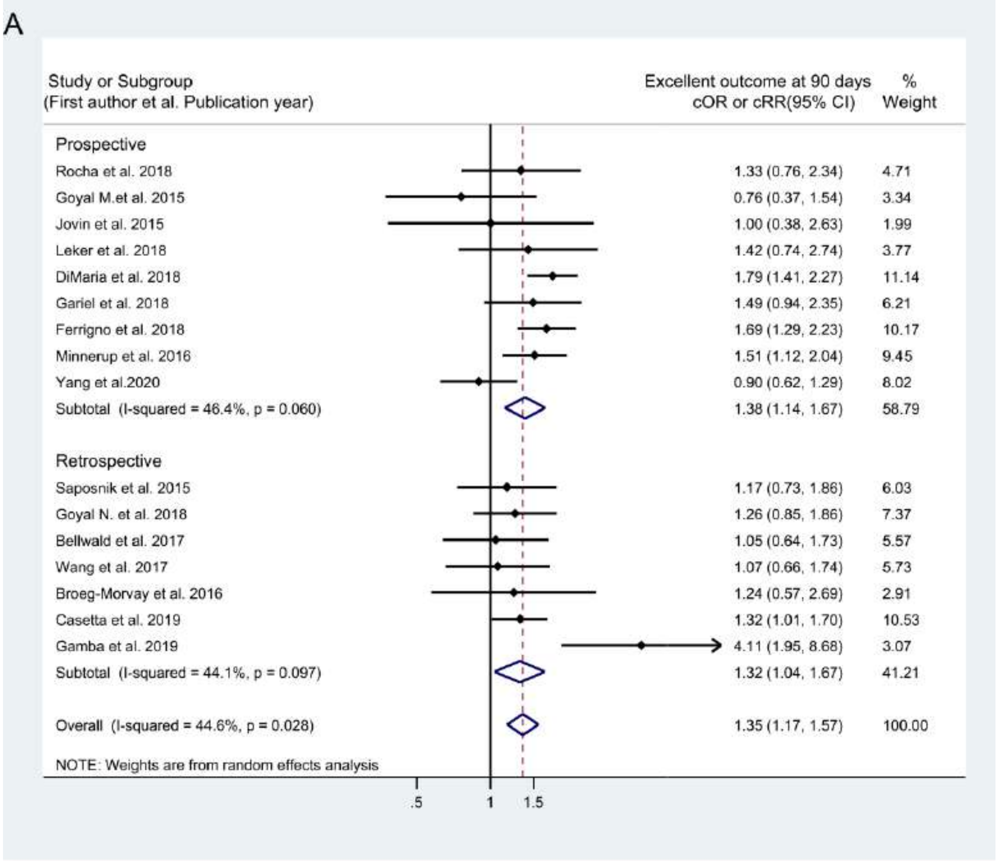


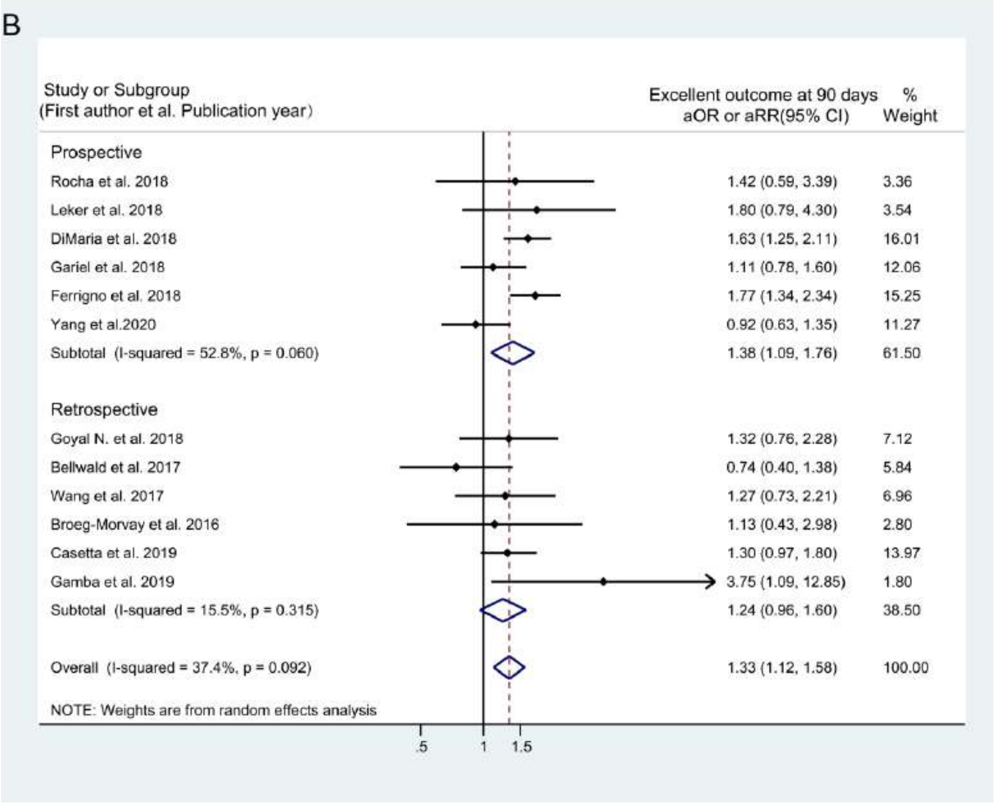

**Supplemental Figure 16.** Forest plots of subgroup analysis assessing excellent outcome at 90 days by ethnicity in unadjusted (A) and adjusted (B) analysis.

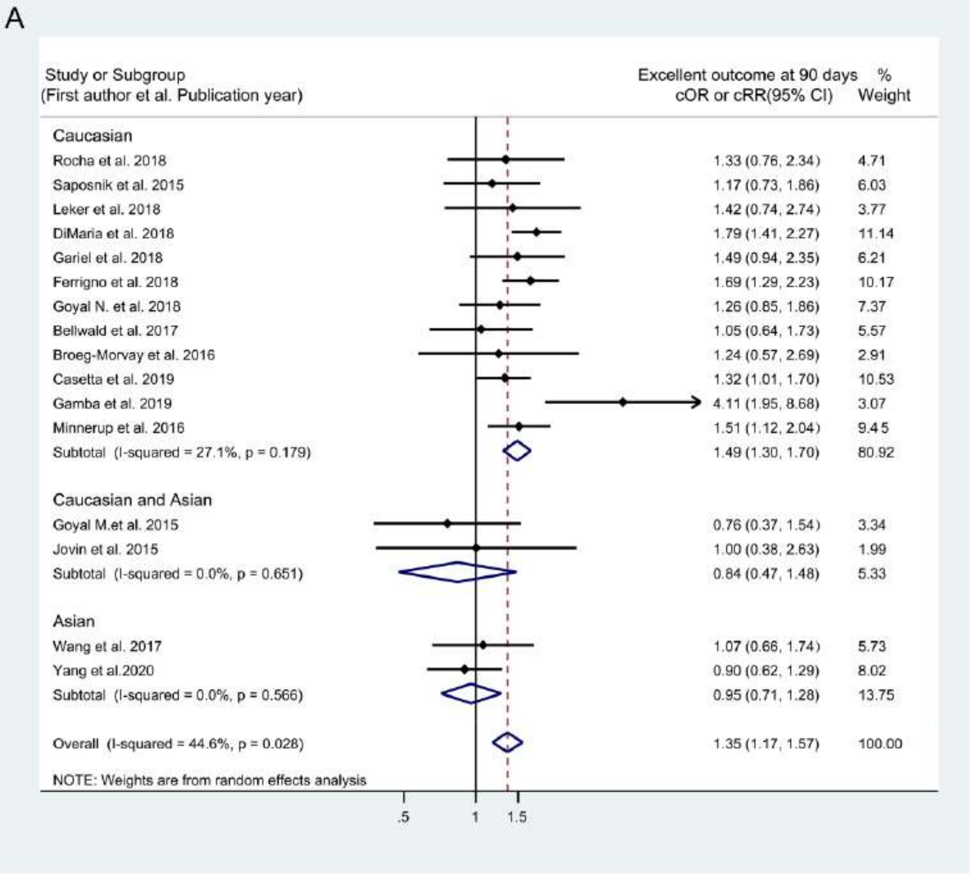


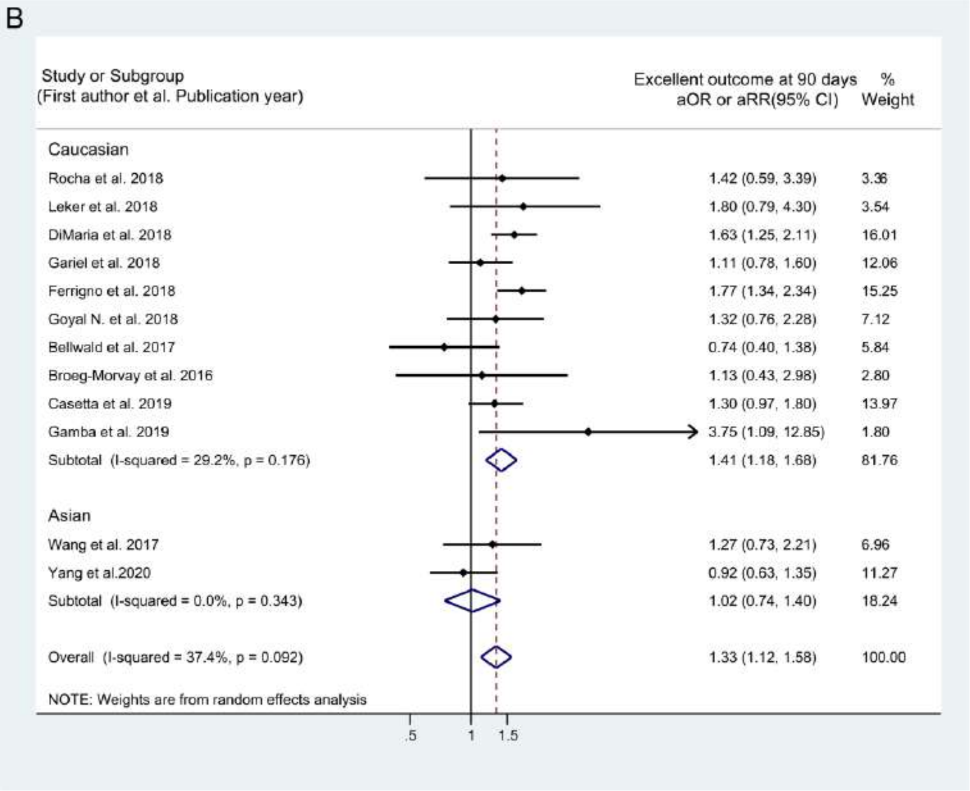

**Supplemental Figure 17.** Forest plots of subgroup analysis assessing favorable outcome at 90 days by location of occluded artery in unadjusted (A) and adjusted (B) analysis.

A

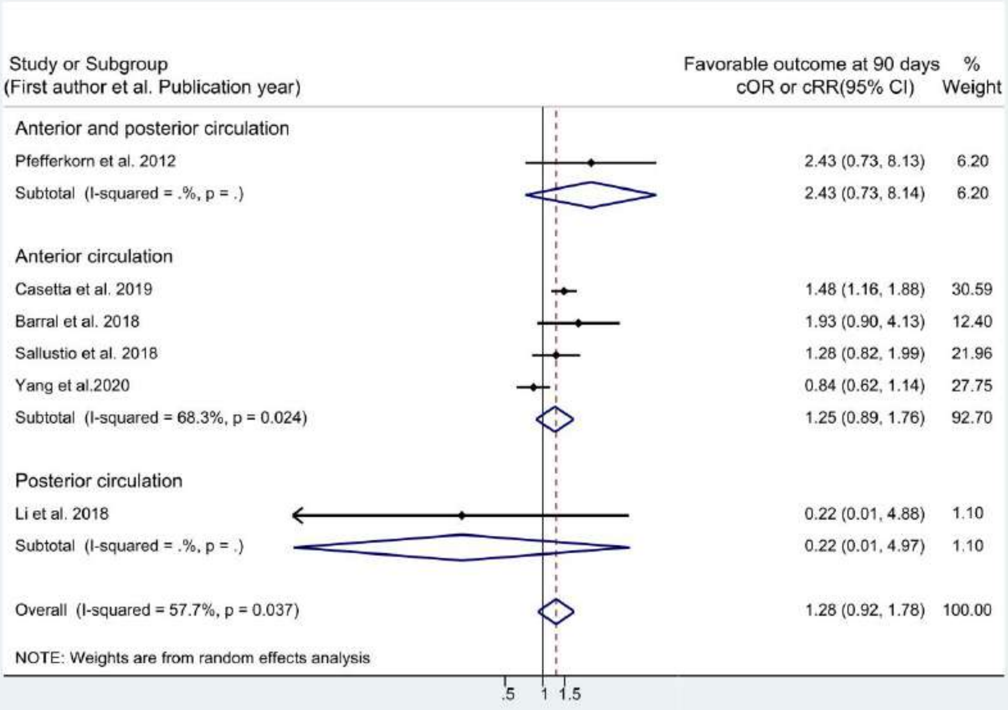


B

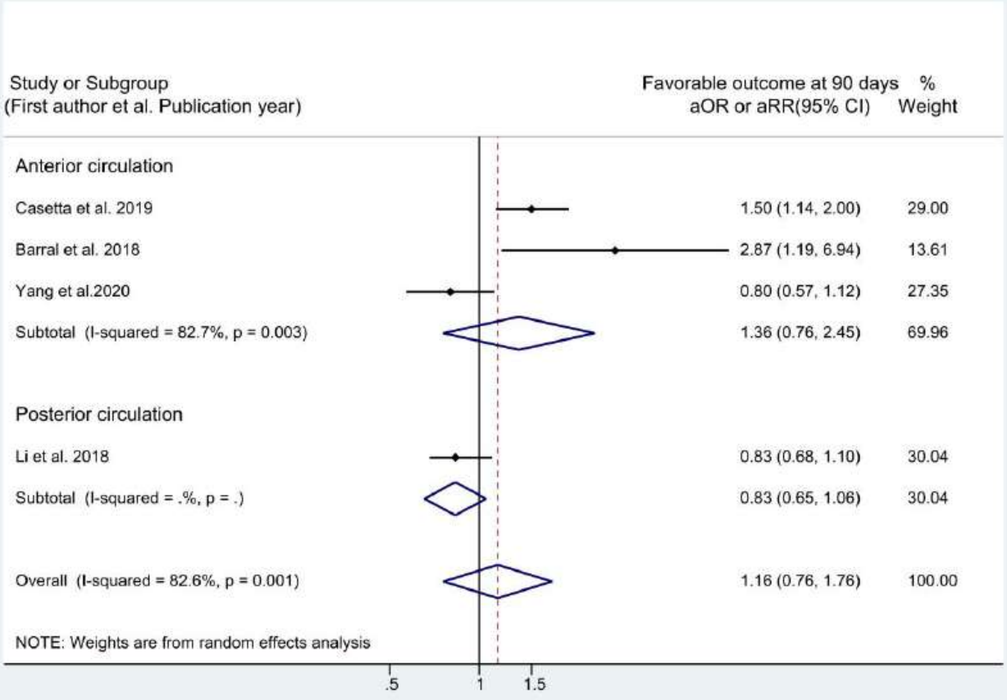

**Supplemental Figure 18.** Forest plots of subgroup analysis assessing favorable outcome at 90 days by study type in unadjusted (A) and adjusted (B) analysis.

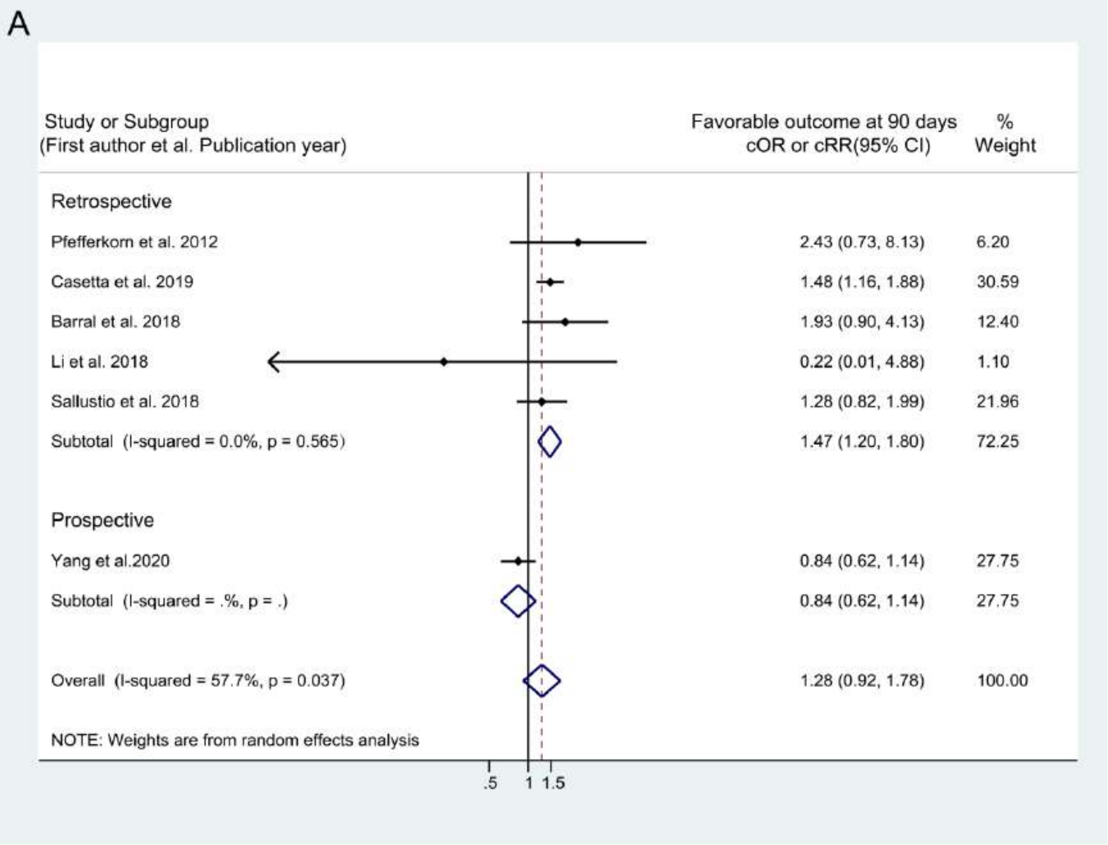


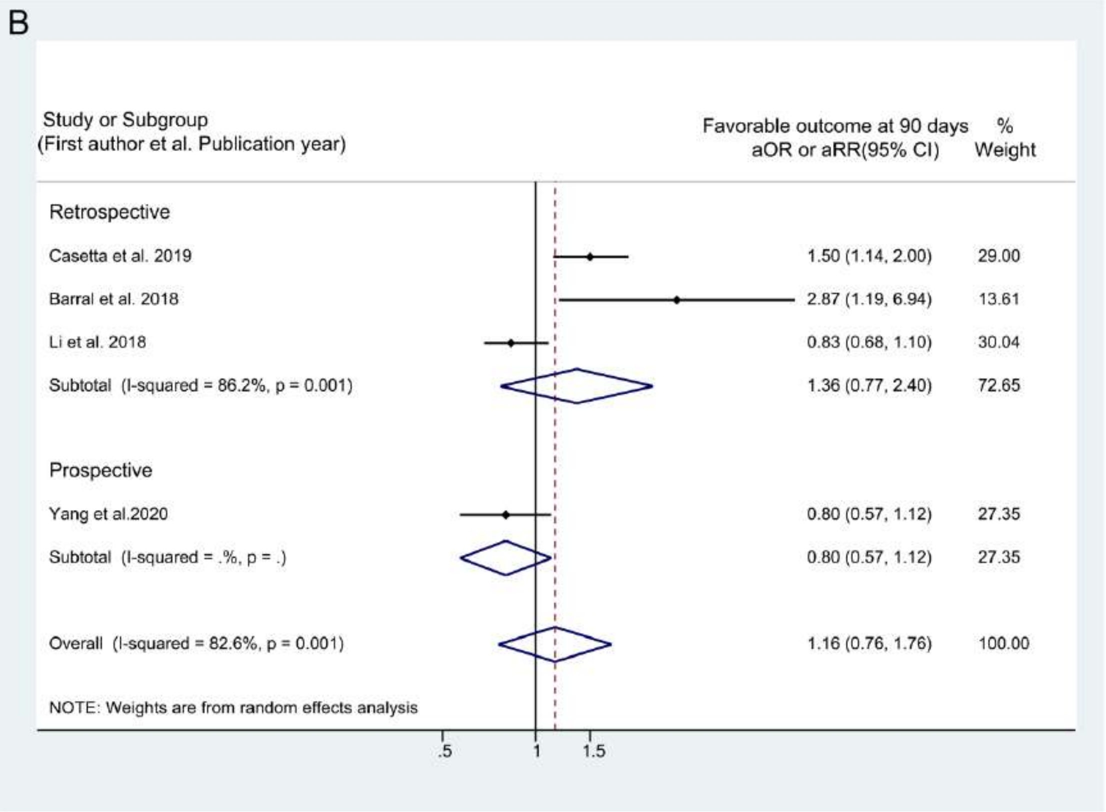

**Supplemental Figure 19.** Forest plots of subgroup analysis assessing favorable outcome at 90 days by ethnicity in unadjusted (A) and adjusted (B) analysis.

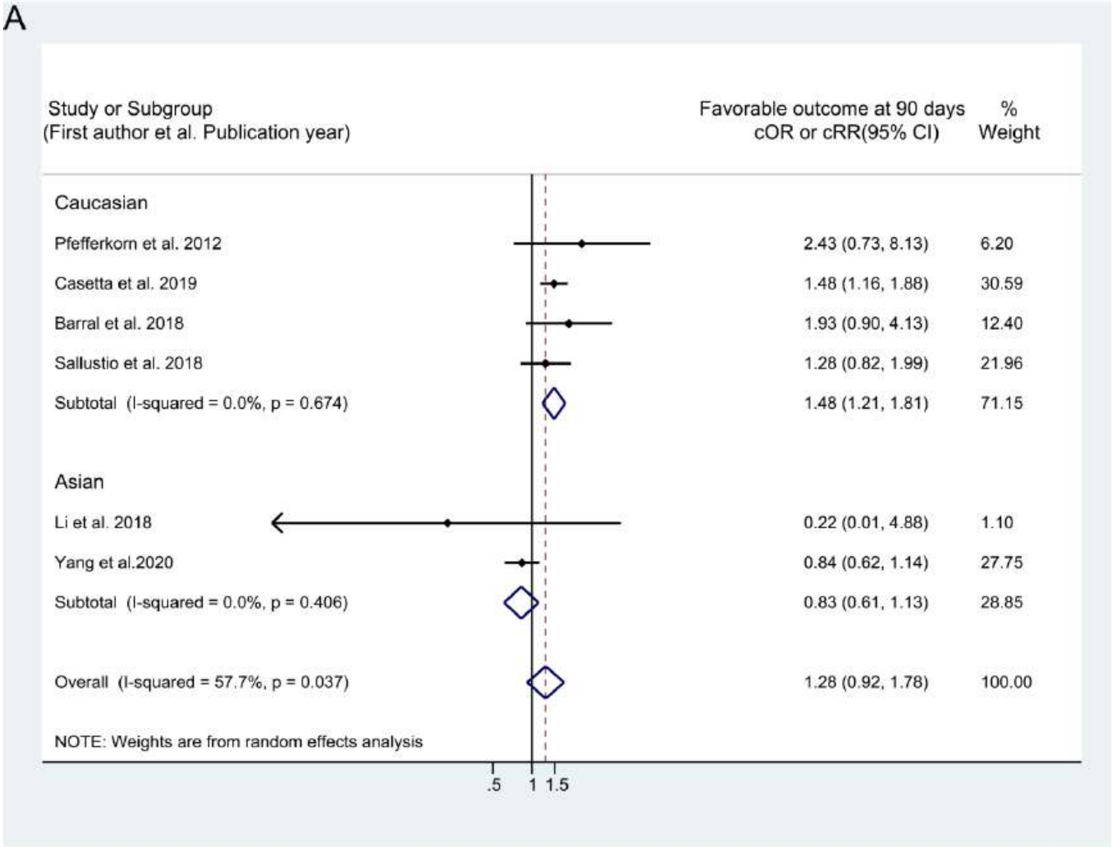


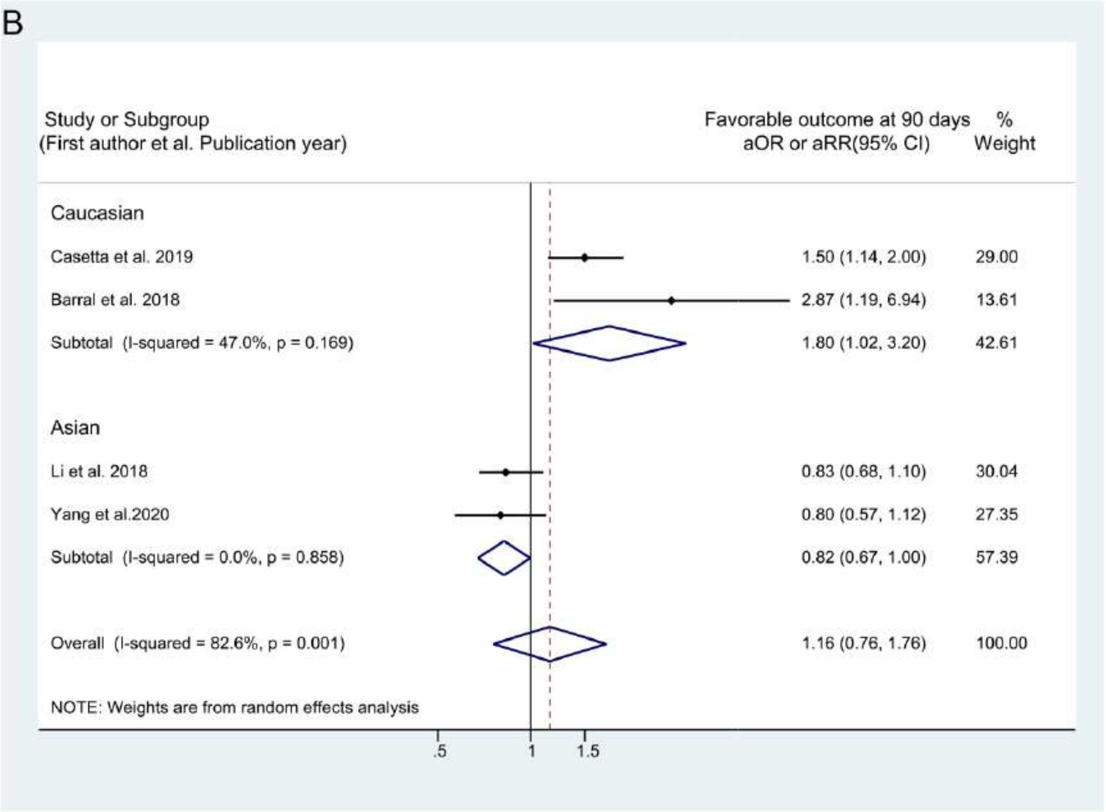

**Supplemental Figure 20.** Forest plots of studies assessing in-hospital mortality in unadjusted (A) and adjusted (B) analysis.

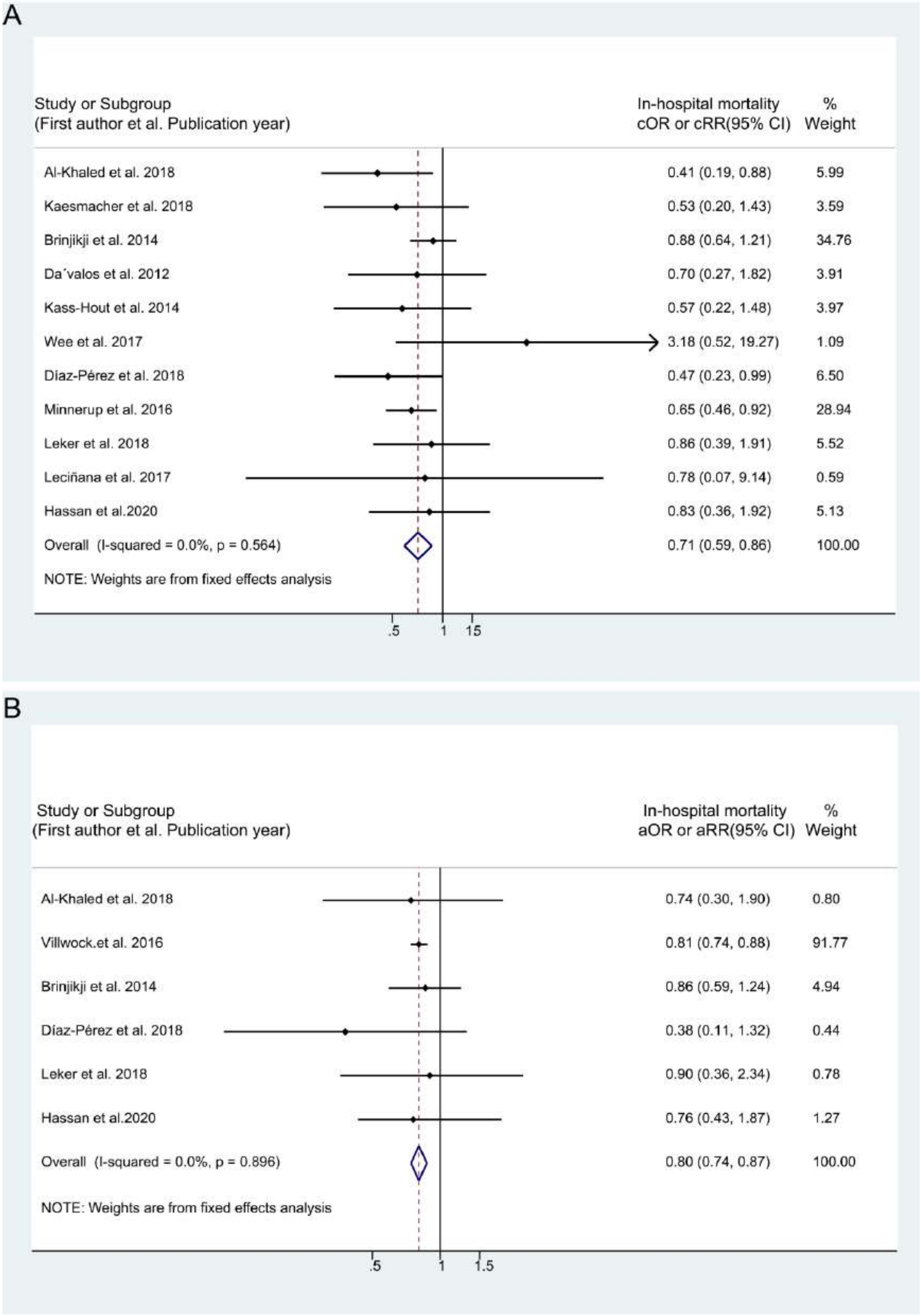

**Supplemental Figure 21.** Sensitivity analysis of the effect of individual studies on the cOR (A) and aOR (B) for in-hospital mortality. cOR, crude odds ratio; aOR, adjusted odds ratio.

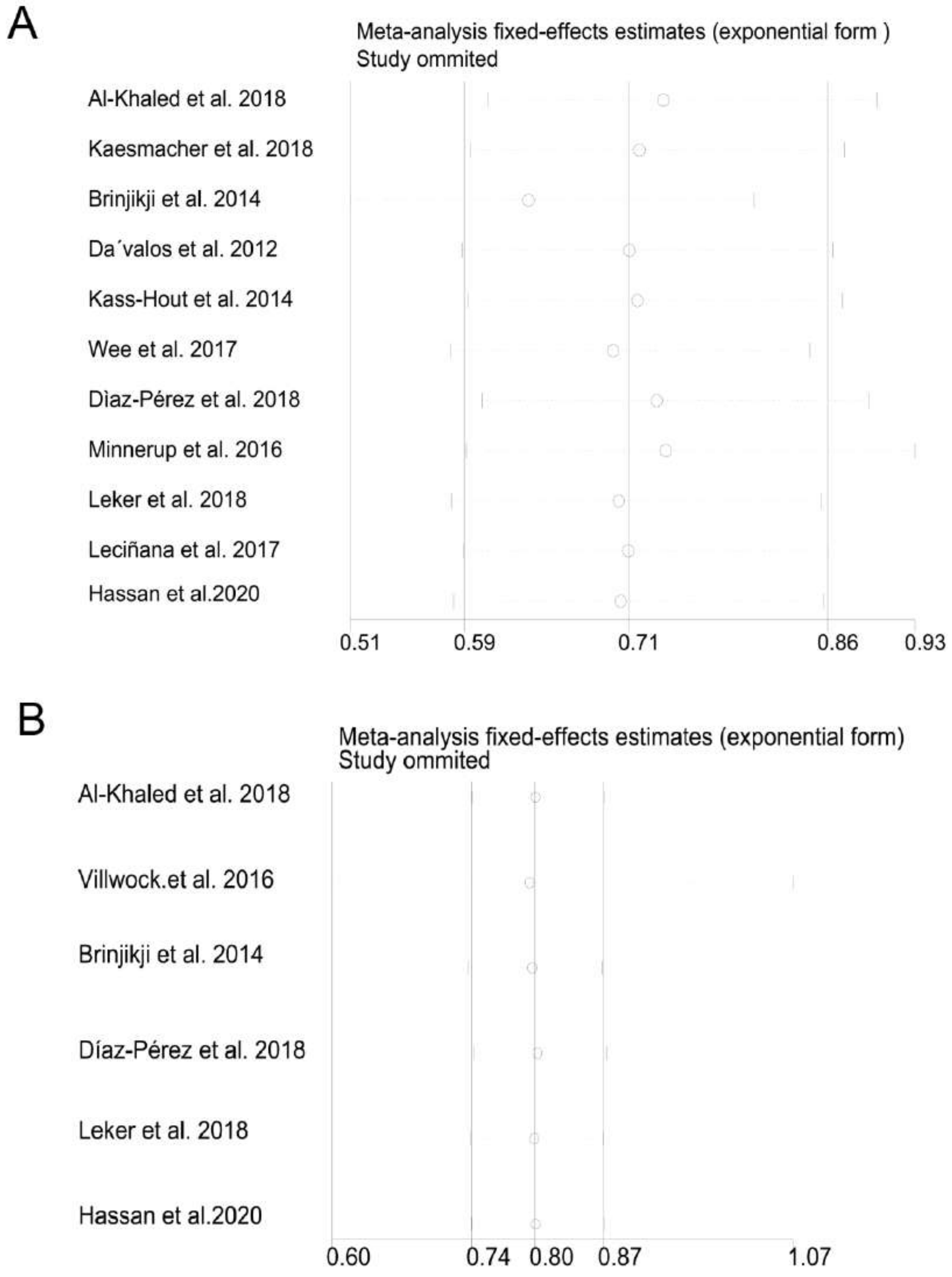

**Supplemental Figure 22.** Forest plots of subgroup analysis assessing mortality within 90 days by location of occluded artery in unadjusted (A) and adjusted (B) analysis. ICA, internal carotid artery. *The acute intracranial vessel occlusion patients with (a) or without (b) concomitant ipsilateral ICA-occlusion or high-grade stenosis were investigated respectively in the study by Bücke et al. 2018.

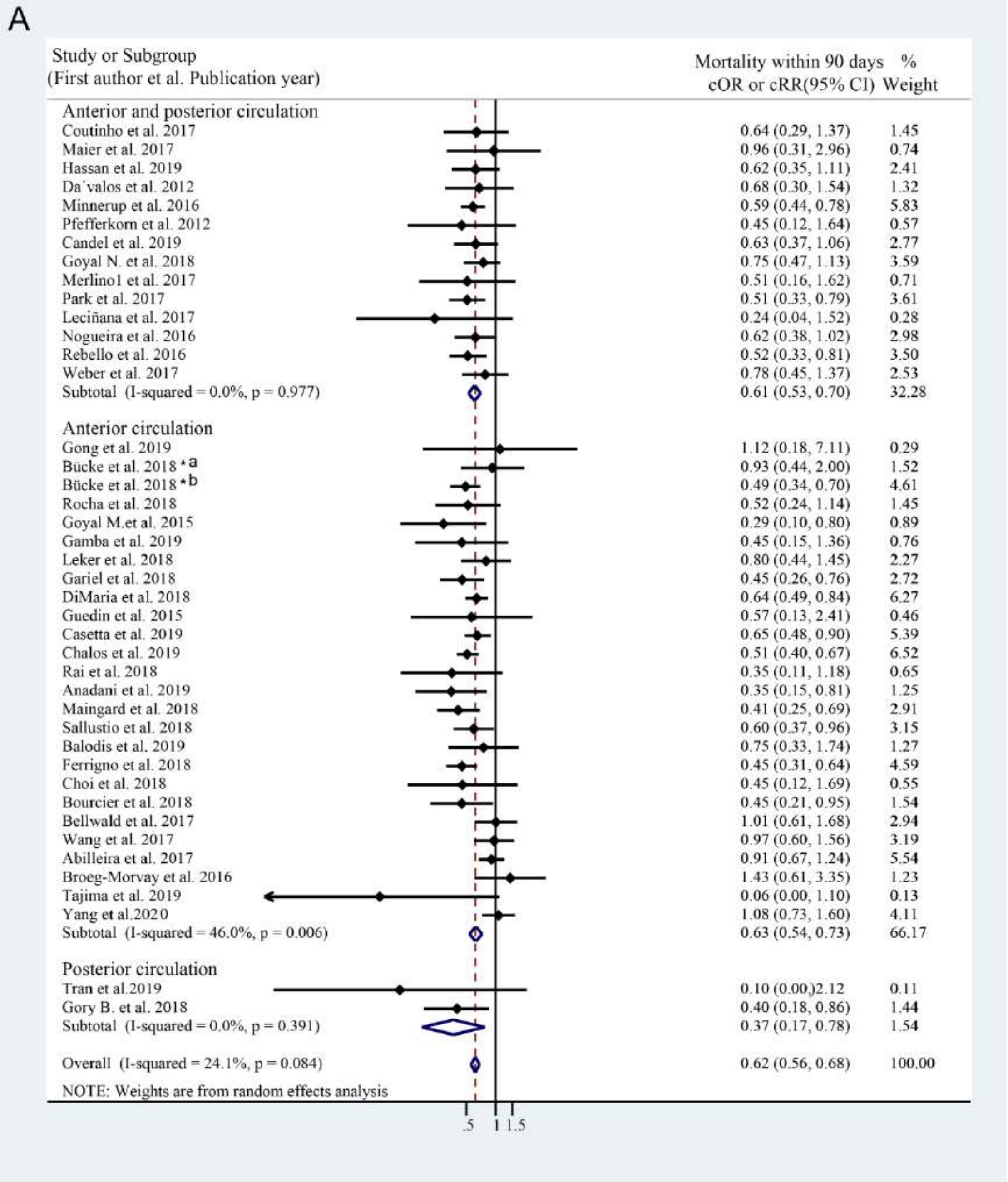

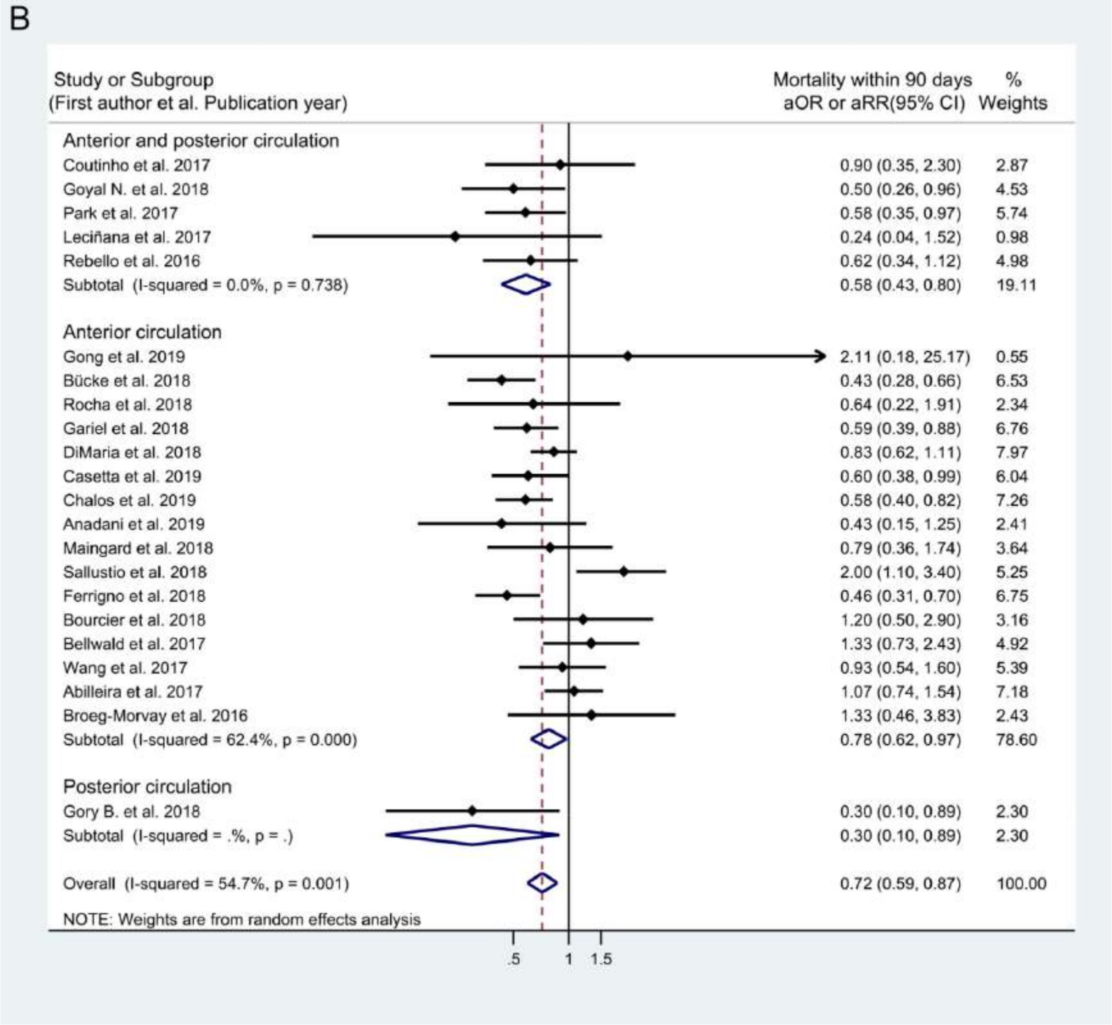
B
Study or Subgroup
(First author et al. Publication year)
Mortality within 90 days
aOR or aRR(95% CI)
%
Weights
Anterior and posterior circulation
Coutinho et al. 2017 0.90 (0.35, 2.30) 2.87
Goyal N. et al. 2018 0.50 (0.26, 0.96) 4.53
Park et al. 2017 0.58 (0.35, 0.97) 5.74
Leciñana et al. 2017 0.24 (0.04, 1.52) 0.98
Rebello et al. 2016 0.62 (0.34, 1.12) 4.98
Subtotal (I-squared = 0.0%, p = 0.738) 0.58 (0.43, 0.80) 19.11
Anterior circulation
Gong et al. 2019 2.11 (0.18, 25.17) 0.55
Bücke et al. 2018 0.43 (0.28, 0.66) 6.53
Rocha et al. 2018 0.64 (0.22, 1.91) 2.34
Gariel et al. 2018 0.59 (0.39, 0.88) 6.76
DiMaria et al. 2018 0.83 (0.62, 1.11) 7.97
Casetta et al. 2019 0.60 (0.38, 0.99) 6.04
Chalos et al. 2019 0.58 (0.40, 0.82) 7.26
Anadani et al. 2019 0.43 (0.15, 1.25) 2.41
Maingard et al. 2018 0.79 (0.36, 1.74) 3.64
Sallustio et al. 2018 2.00 (1.10, 3.40) 5.25
Ferrigno et al. 2018 0.46 (0.31, 0.70) 6.75
Bourcier et al. 2018 1.20 (0.50, 2.90) 3.16
Bellwald et al. 2017 1.33 (0.73, 2.43) 4.92
Wang et al. 2017 0.93 (0.54, 1.60) 5.39
Abilleira et al. 2017 1.07 (0.74, 1.54) 7.18
Broeg-Morvay et al. 2016 1.33 (0.46, 3.83) 2.43
Subtotal (I-squared = 62.4%, p = 0.000) 0.78 (0.62, 0.97) 78.60
Posterior circulation
Gory B. et al. 2018 0.30 (0.10, 0.89) 2.30
Subtotal (I-squared = .%, p = .) 0.30 (0.10, 0.89) 2.30
Overall (I-squared = 54.7%, p = 0.001) 0.72 (0.59, 0.87) 100.00
NOTE: Weights are from random effects analysis
.5
1
1.5

**Supplemental Figure 23.** Forest plots of subgroup analysis assessing mortality within 90 days by ethnicity in unadjusted (A) and adjusted (B) analysis. ICA, internal carotid artery. *The acute intracranial vessel occlusion patients with (a) or without (b) concomitant ipsilateral ICA-occlusion or high-grade stenosis were investigated respectively in the study by Bücke et al. 2018.

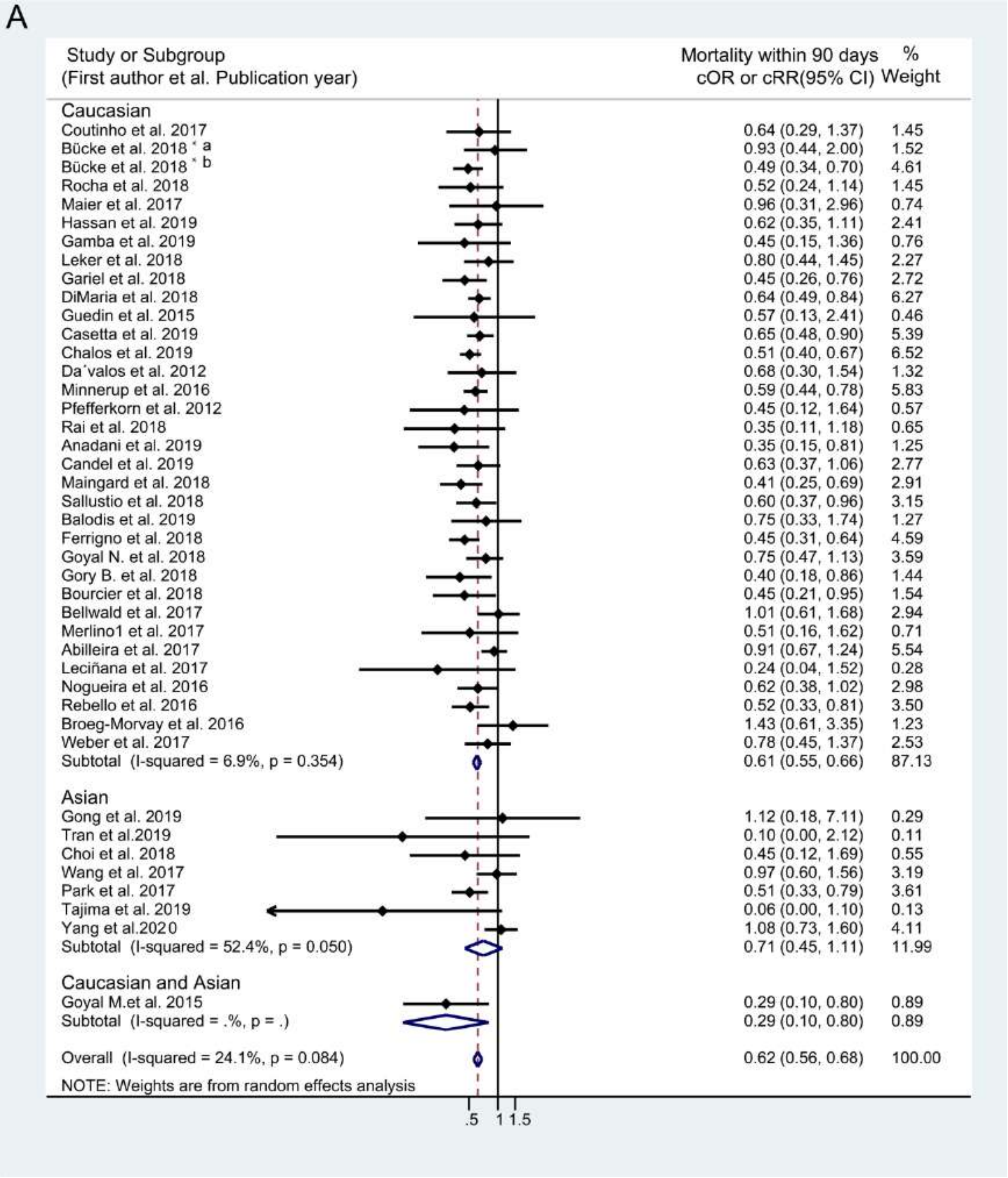

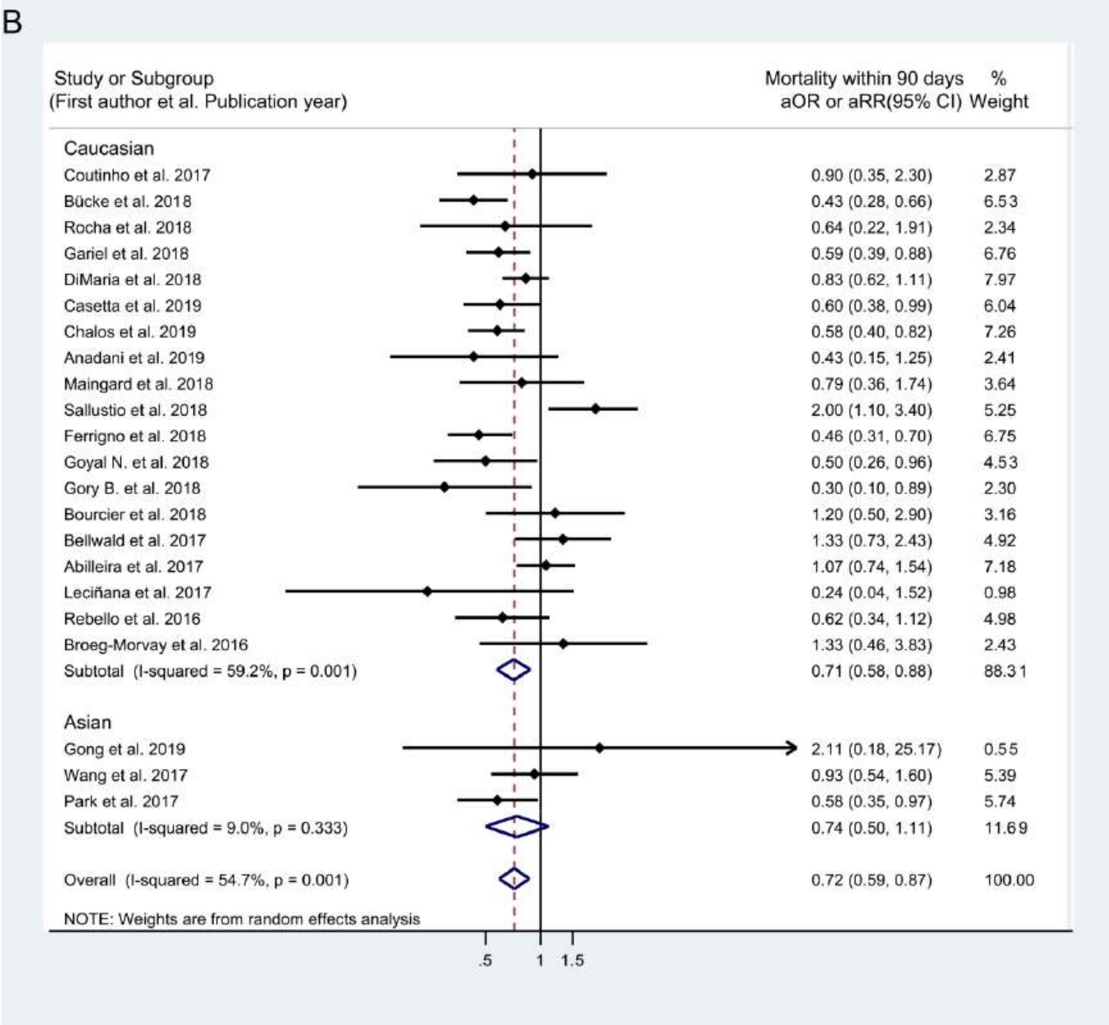
B
Study or Subgroup
(First author et al. Publication year)
Mortality within 90 days
aOR or aRR(95% CI)
%
Weight
Caucasian
Coutinho et al. 2017 0.90 (0.35, 2.30) 2.87
Bücke et al. 2018 0.43 (0.28, 0.66) 6.53
Rocha et al. 2018 0.64 (0.22, 1.91) 2.34
Gariel et al. 2018 0.59 (0.39, 0.88) 6.76
DiMaria et al. 2018 0.83 (0.62, 1.11) 7.97
Casetta et al. 2019 0.60 (0.38, 0.99) 6.04
Chalos et al. 2019 0.58 (0.40, 0.82) 7.26
Anadani et al. 2019 0.43 (0.15, 1.25) 2.41
Maingard et al. 2018 0.79 (0.36, 1.74) 3.64
Sallustio et al. 2018 2.00 (1.10, 3.40) 5.25
Ferrigno et al. 2018 0.46 (0.31, 0.70) 6.75
Goyal N. et al. 2018 0.50 (0.26, 0.96) 4.53
Gory B. et al. 2018 0.30 (0.10, 0.89) 2.30
Bourcier et al. 2018 1.20 (0.50, 2.90) 3.16
Bellwald et al. 2017 1.33 (0.73, 2.43) 4.92
Abilleira et al. 2017 1.07 (0.74, 1.54) 7.18
Leciñana et al. 2017 0.24 (0.04, 1.52) 0.98
Rebello et al. 2016 0.62 (0.34, 1.12) 4.98
Broeg-Morvay et al. 2016 1.33 (0.46, 3.83) 2.43
Subtotal (I-squared = 59.2%, p = 0.001) 0.71 (0.58, 0.88) 88.31
Asian
Gong et al. 2019 2.11 (0.18, 25.17) 0.55
Wang et al. 2017 0.93 (0.54, 1.60) 5.39
Park et al. 2017 0.58 (0.35, 0.97) 5.74
Subtotal (I-squared = 9.0%, p = 0.333) 0.74 (0.50, 1.11) 11.69
Overall (I-squared = 54.7%, p = 0.001) 0.72 (0.59, 0.87) 100.00
NOTE: Weights are from random effects analysis
.5
1
1.5

**Supplemental Figure 24.** Forest plots of studies assessing all mortality in unadjusted (A) and adjusted (B) analysis. ICA, internal carotid artery. *The acute intracranial vessel occlusion patients with (a) or without (b) concomitant ipsilateral ICA-occlusion or high-grade stenosis were investigated respectively in the study by Bücke et al. 2018.

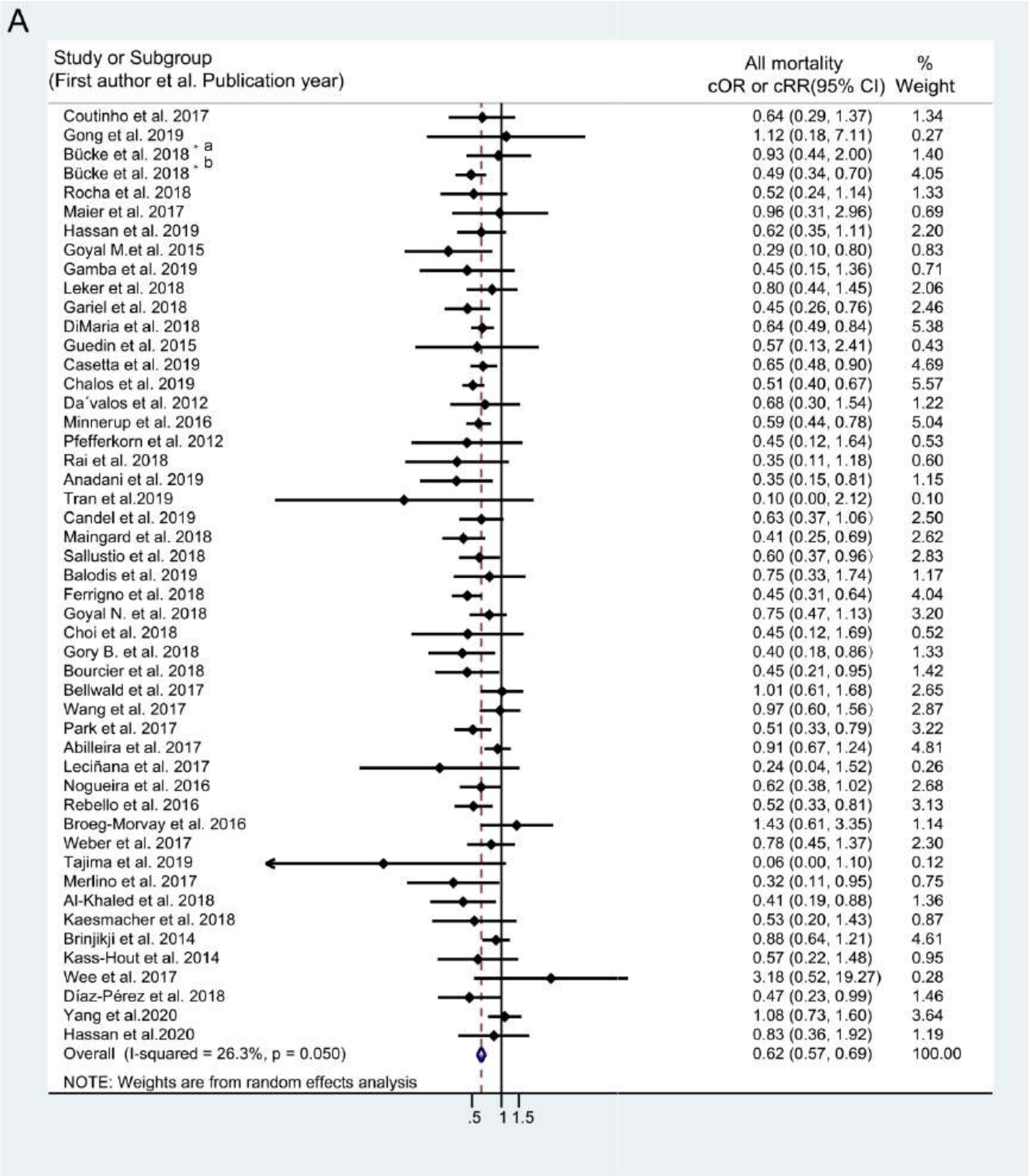

B

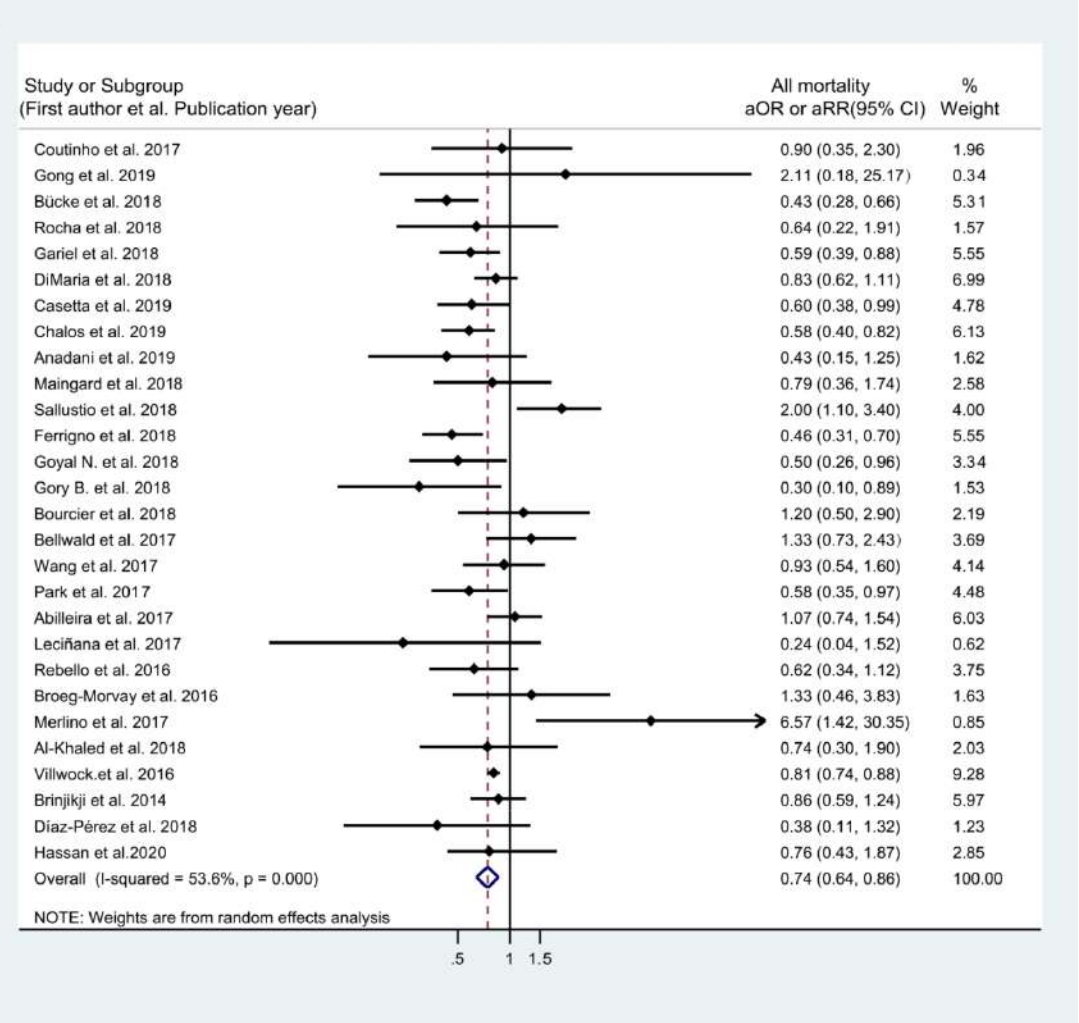
Study or Subgroup
(First author et al. Publication year)
All mortality
aOR or aRR(95% CI)
%
Weight
Coutinho et al. 2017 0.90 (0.35, 2.30) 1.96
Gong et al. 2019 2.11 (0.18, 25.17) 0.34
Bücke et al. 2018 0.43 (0.28, 0.66) 5.31
Rocha et al. 2018 0.64 (0.22, 1.91) 1.57
Gariel et al. 2018 0.59 (0.39, 0.88) 5.55
DiMaria et al. 2018 0.83 (0.62, 1.11) 6.99
Casetta et al. 2019 0.60 (0.38, 0.99) 4.78
Chalos et al. 2019 0.58 (0.40, 0.82) 6.13
Anadani et al. 2019 0.43 (0.15, 1.25) 1.62
Maingard et al. 2018 0.79 (0.36, 1.74) 2.58
Sallustio et al. 2018 2.00 (1.10, 3.40) 4.00
Ferrigno et al. 2018 0.46 (0.31, 0.70) 5.55
Goyal N. et al. 2018 0.50 (0.26, 0.96) 3.34
Gory B. et al. 2018 0.30 (0.10, 0.89) 1.53
Bourcier et al. 2018 1.20 (0.50, 2.90) 2.19
Bellwald et al. 2017 1.33 (0.73, 2.43) 3.69
Wang et al. 2017 0.93 (0.54, 1.60) 4.14
Park et al. 2017 0.58 (0.35, 0.97) 4.48
Abilleira et al. 2017 1.07 (0.74, 1.54) 6.03
Leciñana et al. 2017 0.24 (0.04, 1.52) 0.62
Rebello et al. 2016 0.62 (0.34, 1.12) 3.75
Broeg-Morvay et al. 2016 1.33 (0.46, 3.83) 1.63
Merlino et al. 2017 6.57 (1.42, 30.35) 0.85
Al-Khaled et al. 2018 0.74 (0.30, 1.90) 2.03
Villwock.et al. 2016 0.81 (0.74, 0.88) 9.28
Brinjikji et al. 2014 0.86 (0.59, 1.24) 5.97
Díaz-Pérez et al. 2018 0.38 (0.11, 1.32) 1.23
Hassan et al.2020 0.76 (0.43, 1.87) 2.85
Overall (I-squared = 53.6%, p = 0.000) 0.74 (0.64, 0.86) 100.00
NOTE: Weights are from random effects analysis
.5
1
1.5

**Supplemental Figure 25.** Forest plots of subgroup analysis assessing mortality within 90 days by study type in unadjusted (A) and adjusted (B) analysis. ICA, internal carotid artery. *The acute intracranial vessel occlusion patients with (a) or without (b) concomitant ipsilateral ICA-occlusion or high-grade stenosis were investigated respectively in the study by Bücke et al. 2018.

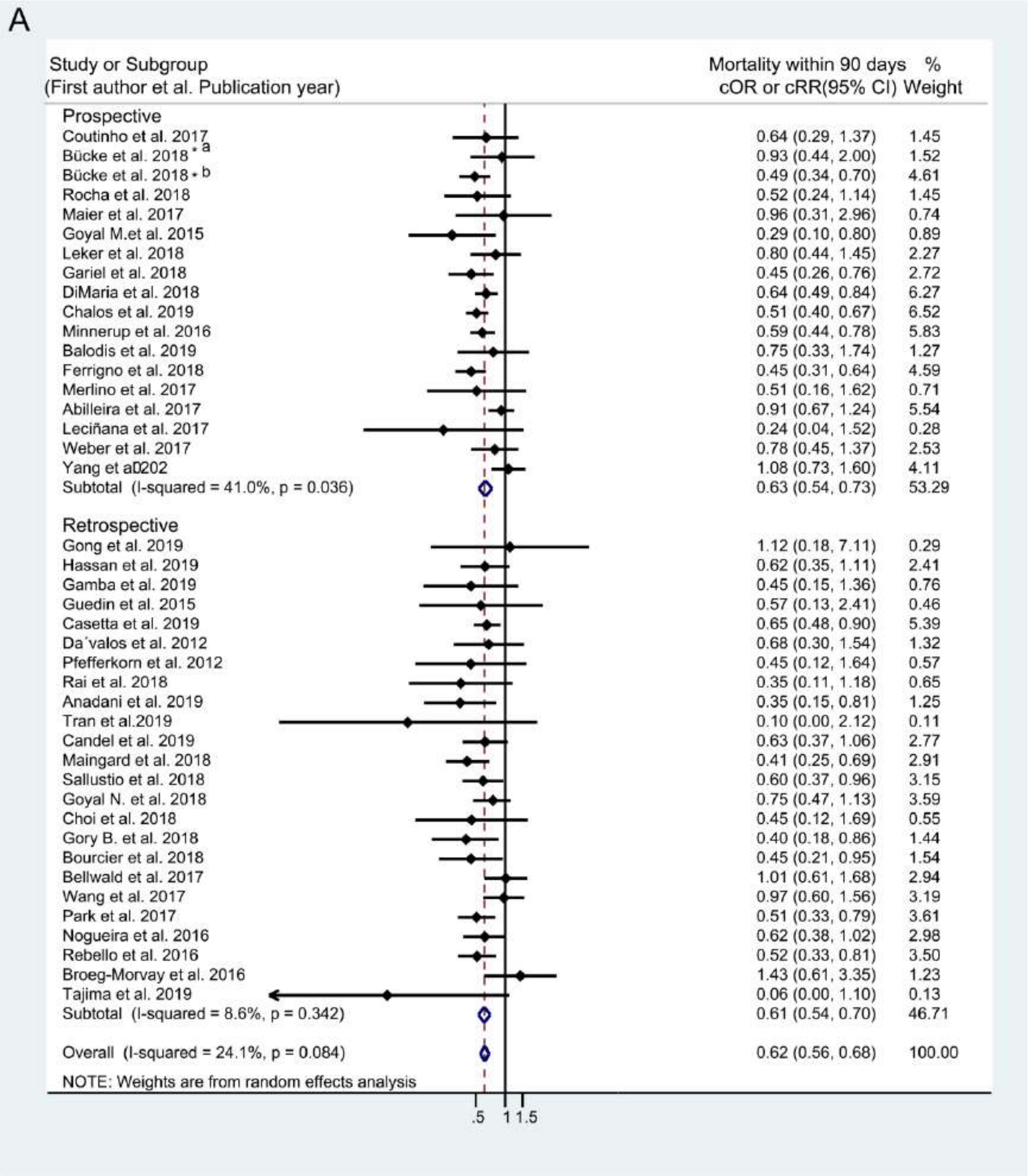

B

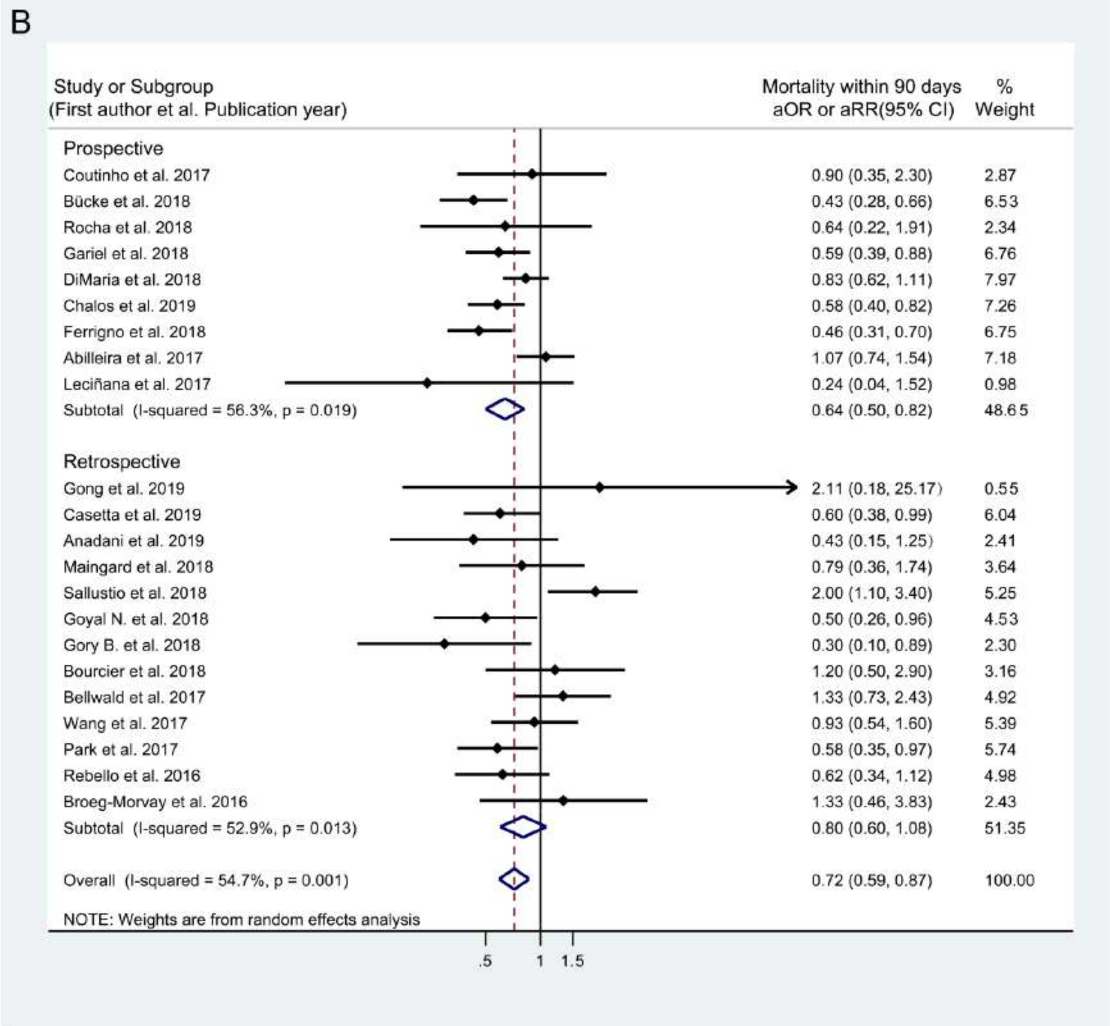

Study or Subgroup
(First author et al. Publication year)
Mortality within 90 days
aOR or aRR(95% CI)
%
Weight
Prospective
Coutinho et al. 2017
0.90 (0.35, 2.30)
2.87
Bücke et al. 2018
0.43 (0.28, 0.66)
6.53
Rocha et al. 2018
0.64 (0.22, 1.91)
2.34
Gariel et al. 2018
0.59 (0.39, 0.88)
6.76
DiMaria et al. 2018
0.83 (0.62, 1.11)
7.97
Chalos et al. 2019
0.58 (0.40, 0.82)
7.26
Ferrigno et al. 2018
0.46 (0.31, 0.70)
6.75
Abilleira et al. 2017
1.07 (0.74, 1.54)
7.18
Leciñana et al. 2017
0.24 (0.04, 1.52)
0.98
Subtotal (I-squared = 56.3%, p = 0.019)
0.64 (0.50, 0.82)
48.65
Retrospective
Gong et al. 2019
2.11 (0.18, 25.17)
0.55
Casetta et al. 2019
0.60 (0.38, 0.99)
6.04
Anadani et al. 2019
0.43 (0.15, 1.25)
2.41
Maingard et al. 2018
0.79 (0.36, 1.74)
3.64
Sallustio et al. 2018
2.00 (1.10, 3.40)
5.25
Goyal N. et al. 2018
0.50 (0.26, 0.96)
4.53
Gory B. et al. 2018
0.30 (0.10, 0.89)
2.30
Bourcier et al. 2018
1.20 (0.50, 2.90)
3.16
Bellwald et al. 2017
1.33 (0.73, 2.43)
4.92
Wang et al. 2017
0.93 (0.54, 1.60)
5.39
Park et al. 2017
0.58 (0.35, 0.97)
5.74
Rebello et al. 2016
0.62 (0.34, 1.12)
4.98
Broeg-Morvay et al. 2016
1.33 (0.46, 3.83)
2.43
Subtotal (I-squared = 52.9%, p = 0.013)
0.80 (0.60, 1.08)
51.35
Overall (I-squared = 54.7%, p = 0.001)
0.72 (0.59, 0.87)
100.00
NOTE: Weights are from random effects analysis
.5
1
1.5

**Supplemental Figure 26.** Forest plots of subgroup analysis assessing in-hospital mortality by location of occluded artery in unadjusted (A) and adjusted (B) analysis.

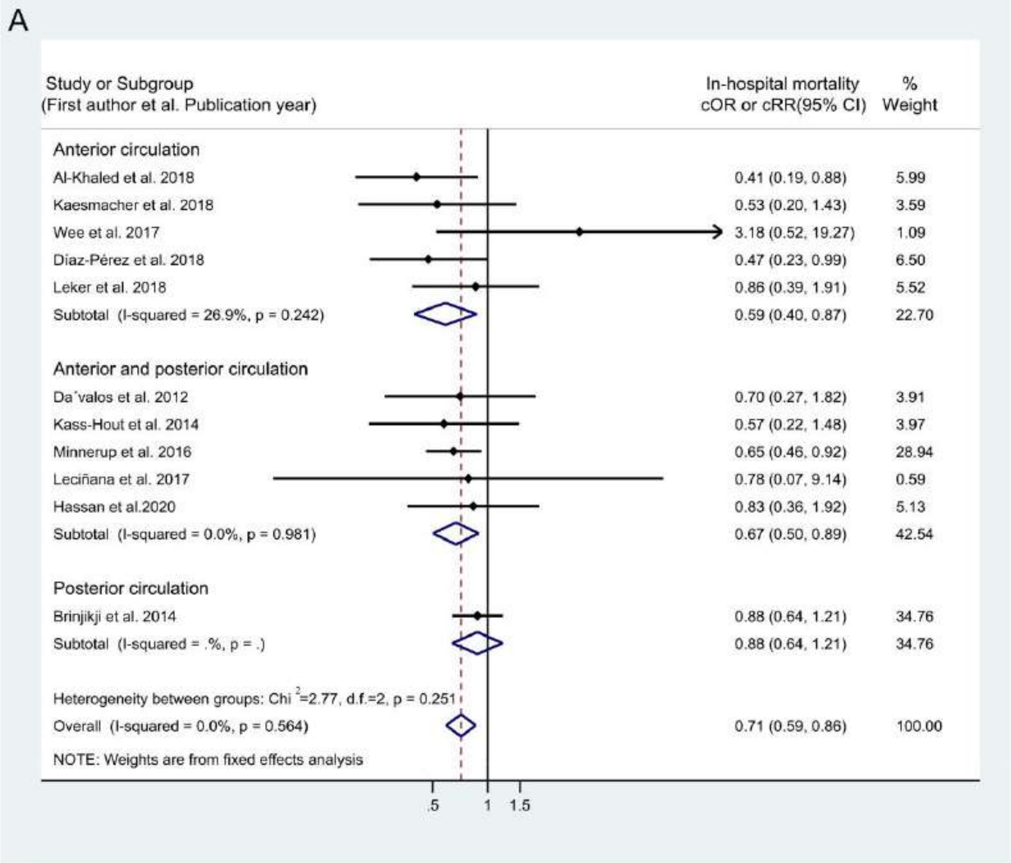


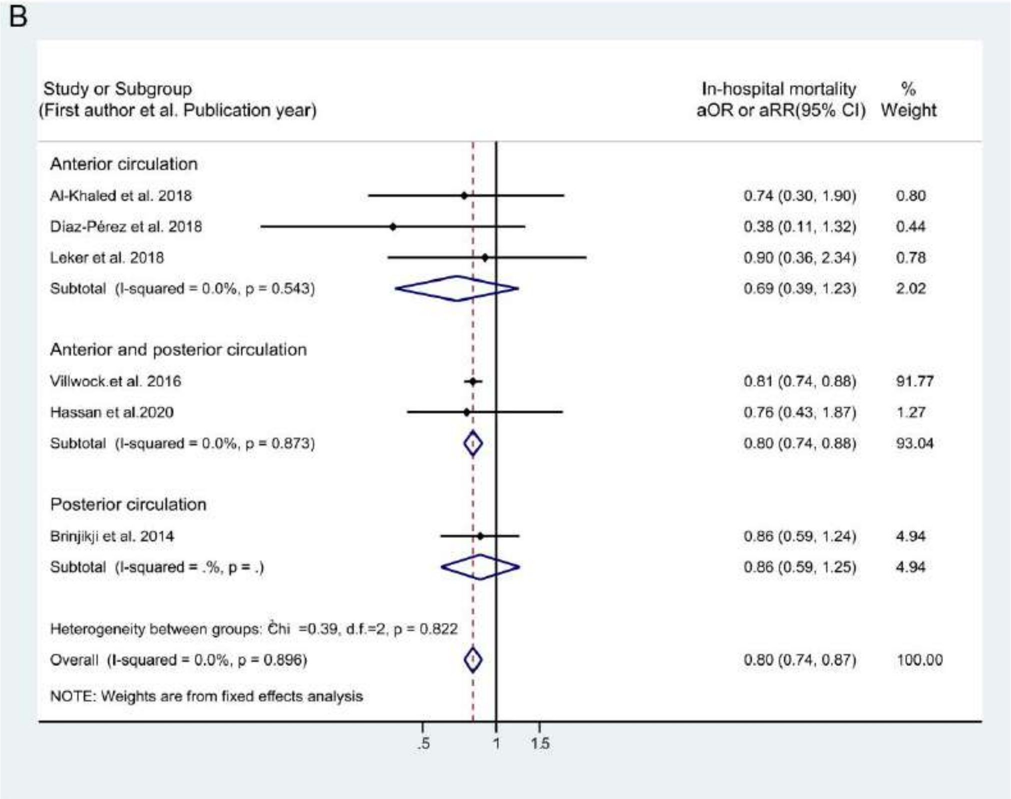

**Supplemental Figure 27.** Forest plots of subgroup analysis assessing in-hospital mortality by study type in unadjusted (A) and adjusted (B) analysis.

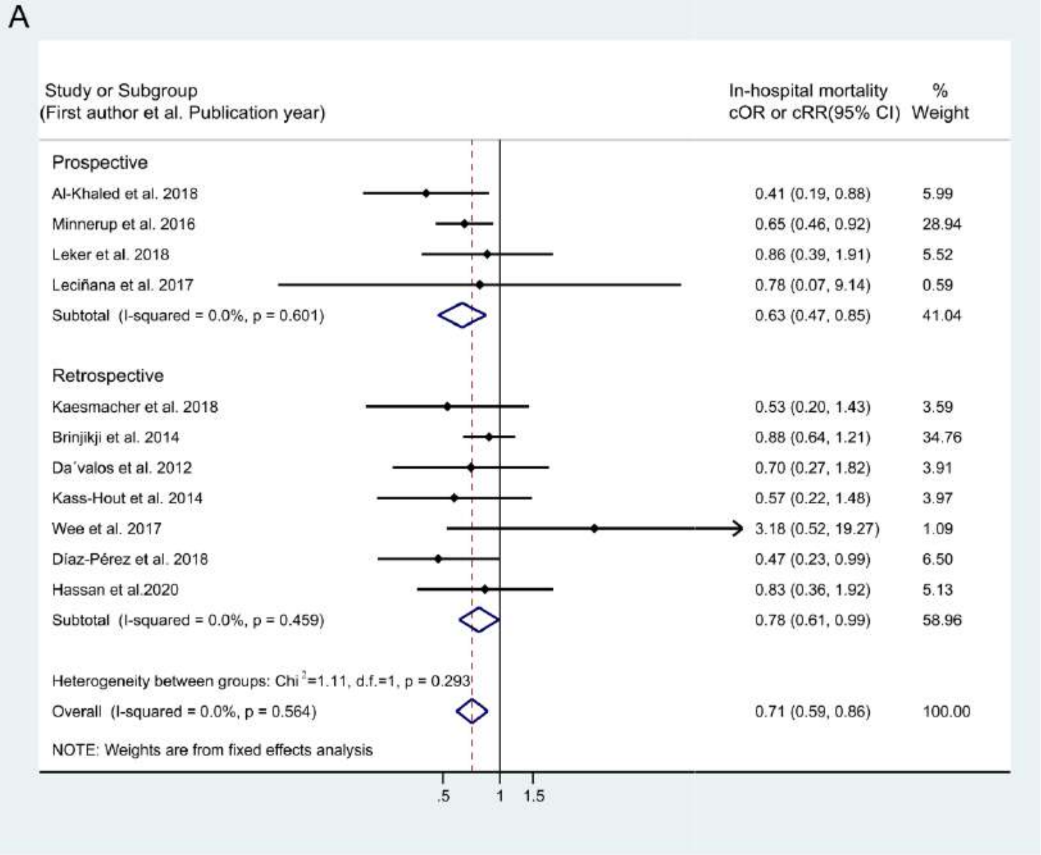


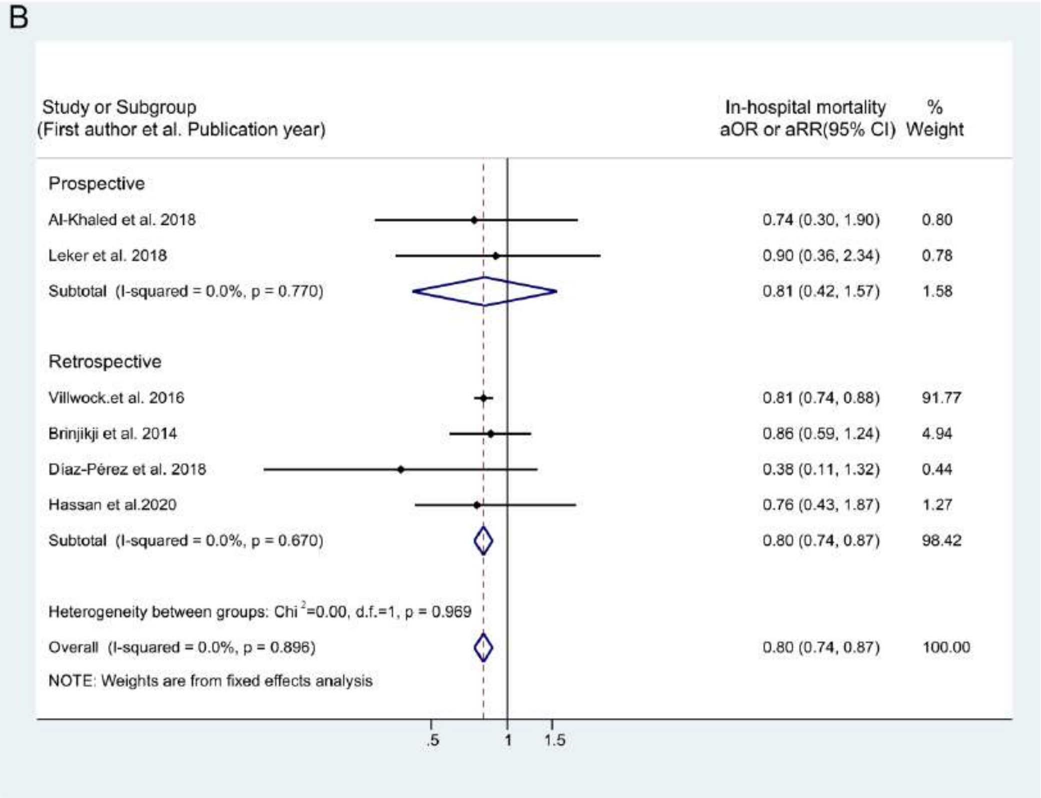

**Supplemental Figure 28.** Forest plots of subgroup analysis assessing all mortality by location of occluded artery in unadjusted (A) and adjusted (B) analysis. ICA, internal carotid artery. *The acute intracranial vessel occlusion patients with (a) or without (b) concomitant ipsilateral ICA-occlusion or high-grade stenosis were investigated respectively in the study by Bücke et al. 2018.

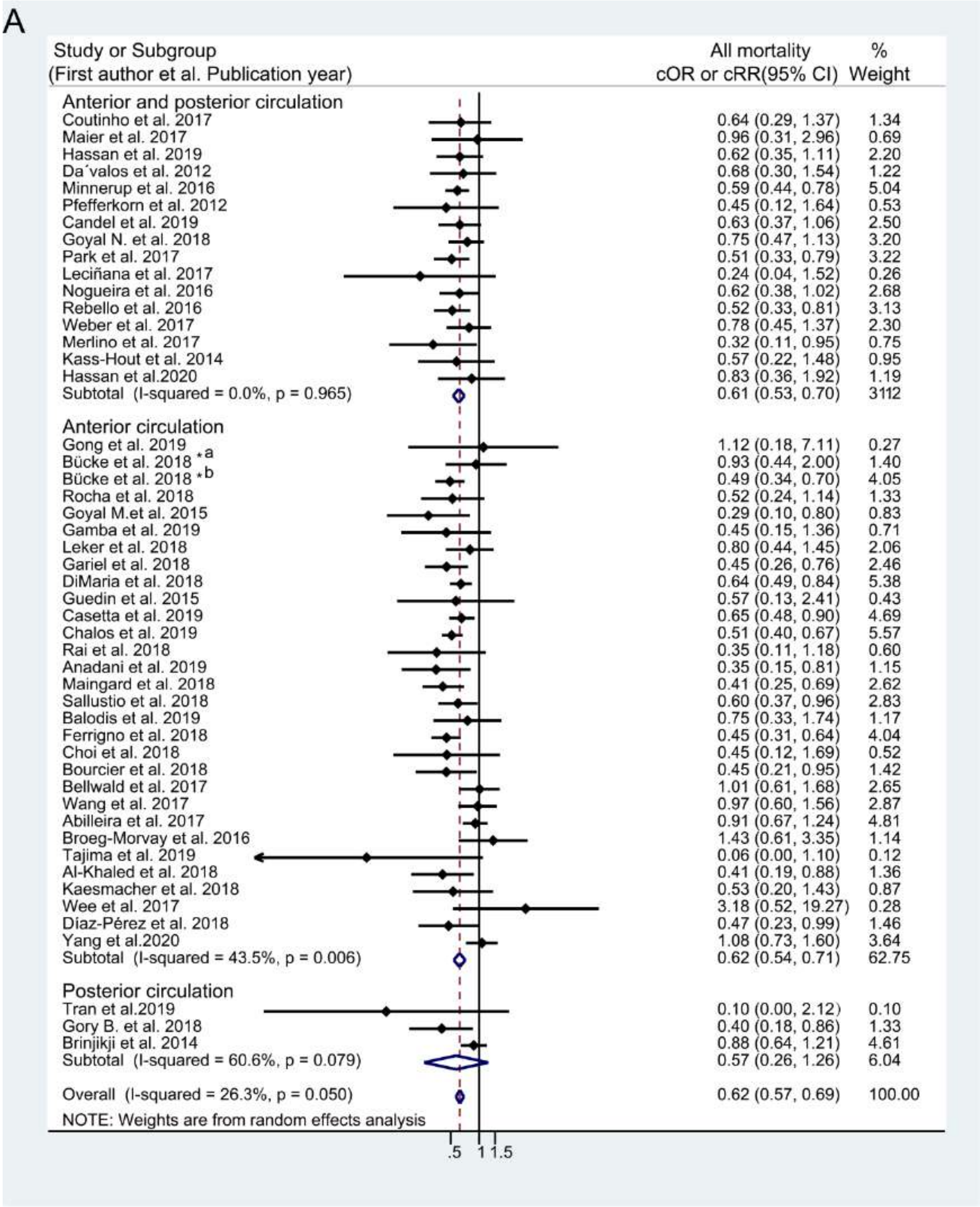

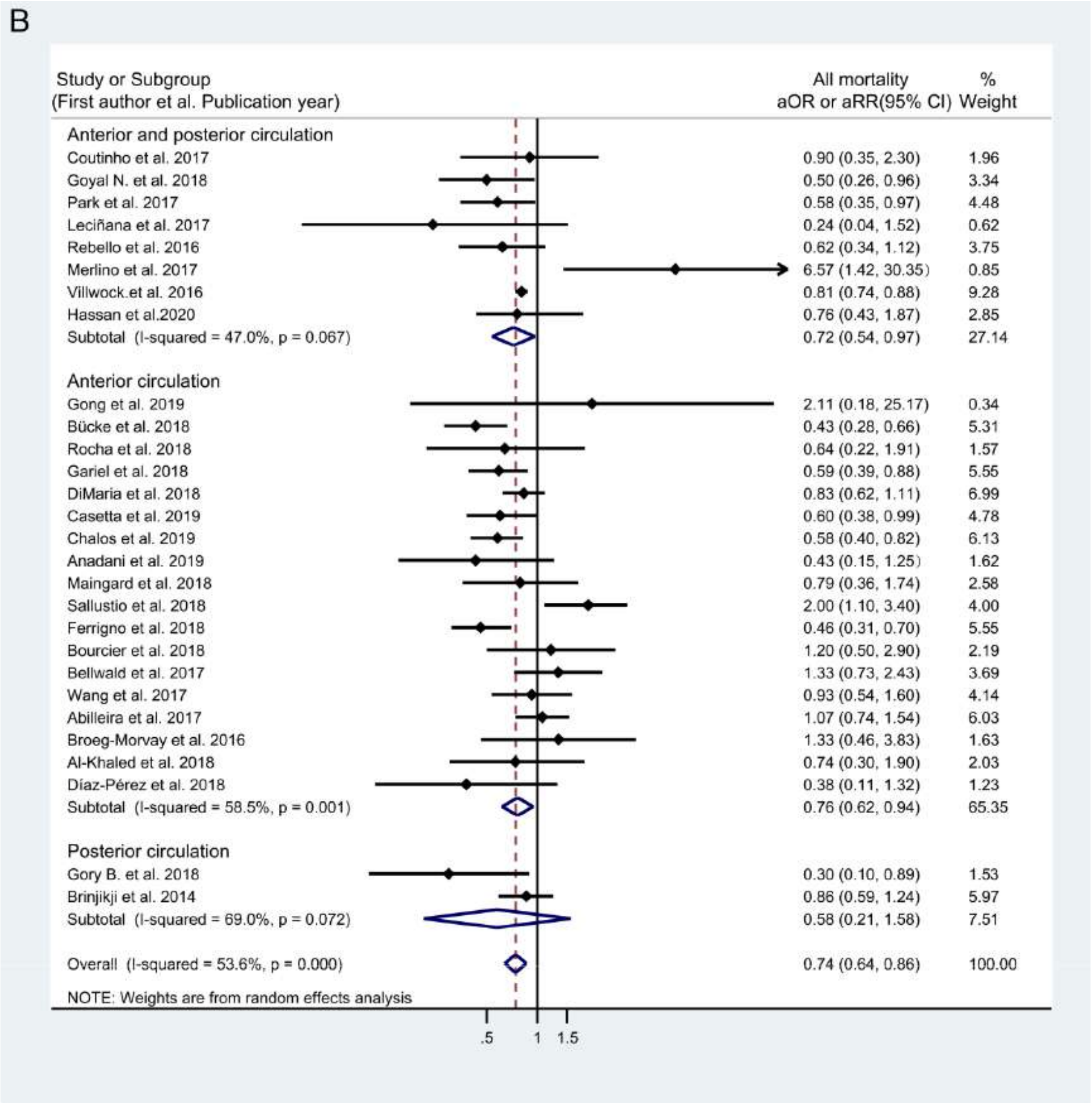
B
Study or Subgroup
(First author et al. Publication year)
All mortality
aOR or aRR(95% CI)
%
Weight
Anterior and posterior circulation
Coutinho et al. 2017 0.90 (0.35, 2.30) 1.96
Goyal N. et al. 2018 0.50 (0.26, 0.96) 3.34
Park et al. 2017 0.58 (0.35, 0.97) 4.48
Leciñana et al. 2017 0.24 (0.04, 1.52) 0.62
Rebello et al. 2016 0.62 (0.34, 1.12) 3.75
Merlino et al. 2017 6.57 (1.42, 30.35) 0.85
Villwock.et al. 2016 0.81 (0.74, 0.88) 9.28
Hassan et al.2020 0.76 (0.43, 1.87) 2.85
Subtotal (I-squared = 47.0%, p = 0.067) 0.72 (0.54, 0.97) 27.14
Anterior circulation
Gong et al. 2019 2.11 (0.18, 25.17) 0.34
Bücke et al. 2018 0.43 (0.28, 0.66) 5.31
Rocha et al. 2018 0.64 (0.22, 1.91) 1.57
Gariel et al. 2018 0.59 (0.39, 0.88) 5.55
DiMaria et al. 2018 0.83 (0.62, 1.11) 6.99
Casetta et al. 2019 0.60 (0.38, 0.99) 4.78
Chalos et al. 2019 0.58 (0.40, 0.82) 6.13
Anadani et al. 2019 0.43 (0.15, 1.25) 1.62
Maingard et al. 2018 0.79 (0.36, 1.74) 2.58
Sallustio et al. 2018 2.00 (1.10, 3.40) 4.00
Ferrigno et al. 2018 0.46 (0.31, 0.70) 5.55
Bourcier et al. 2018 1.20 (0.50, 2.90) 2.19
Bellwald et al. 2017 1.33 (0.73, 2.43) 3.69
Wang et al. 2017 0.93 (0.54, 1.60) 4.14
Abilleira et al. 2017 1.07 (0.74, 1.54) 6.03
Broeg-Morvay et al. 2016 1.33 (0.46, 3.83) 1.63
Al-Khaled et al. 2018 0.74 (0.30, 1.90) 2.03
Díaz-Pérez et al. 2018 0.38 (0.11, 1.32) 1.23
Subtotal (I-squared = 58.5%, p = 0.001) 0.76 (0.62, 0.94) 65.35
Posterior circulation
Gory B. et al. 2018 0.30 (0.10, 0.89) 1.53
Brinjikji et al. 2014 0.86 (0.59, 1.24) 5.97
Subtotal (I-squared = 69.0%, p = 0.072) 0.58 (0.21, 1.58) 7.51
Overall (I-squared = 53.6%, p = 0.000) 0.74 (0.64, 0.86) 100.00
NOTE: Weights are from random effects analysis
.5
1
1.5

**Supplemental Figure 29.** Forest plots of subgroup analysis assessing all mortality by study type in unadjusted (A) and adjusted (B) analysis. ICA, internal carotid artery. [*]The acute intracranial vessel occlusion patients with (a) or without (b) concomitant ipsilateral ICA-occlusion or high-grade stenosis were investigated respectively in the study by Bücke et al. 2018.

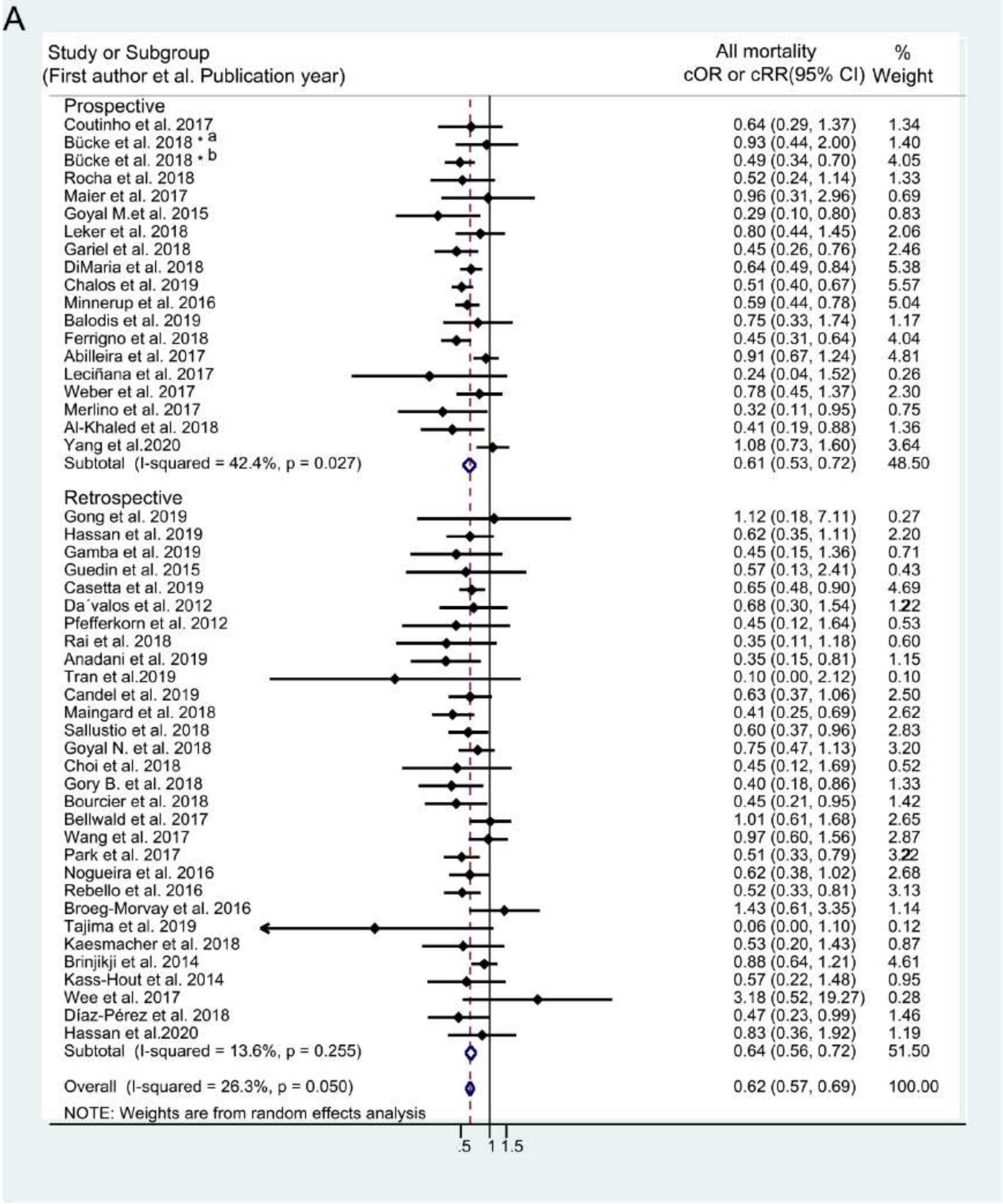

B

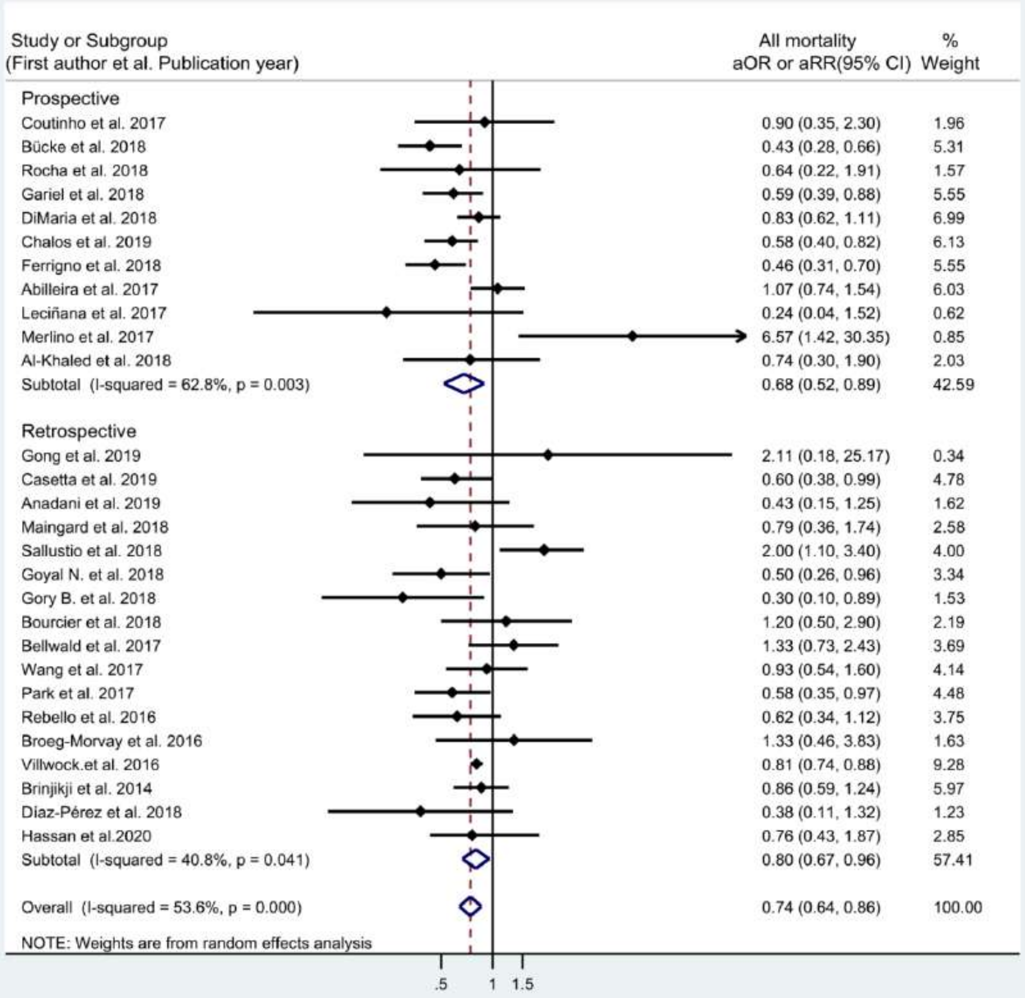
Study or Subgroup
(First author et al. Publication year)
All mortality
aOR or aRR(95% CI)
%
Weight
Prospective
Coutinho et al. 2017 0.90 (0.35, 2.30) 1.96
Bücke et al. 2018 0.43 (0.28, 0.66) 5.31
Rocha et al. 2018 0.64 (0.22, 1.91) 1.57
Gariel et al. 2018 0.59 (0.39, 0.88) 5.55
DiMaria et al. 2018 0.83 (0.62, 1.11) 6.99
Chalos et al. 2019 0.58 (0.40, 0.82) 6.13
Ferrigno et al. 2018 0.46 (0.31, 0.70) 5.55
Abilleira et al. 2017 1.07 (0.74, 1.54) 6.03
Leciñana et al. 2017 0.24 (0.04, 1.52) 0.62
Merlino et al. 2017 6.57 (1.42, 30.35) 0.85
Al-Khaled et al. 2018 0.74 (0.30, 1.90) 2.03
Subtotal (I-squared = 62.8%, p = 0.003) 0.68 (0.52, 0.89) 42.59
Retrospective
Gong et al. 2019 2.11 (0.18, 25.17) 0.34
Casetta et al. 2019 0.60 (0.38, 0.99) 4.78
Anadani et al. 2019 0.43 (0.15, 1.25) 1.62
Maingard et al. 2018 0.79 (0.36, 1.74) 2.58
Sallustio et al. 2018 2.00 (1.10, 3.40) 4.00
Goyal N. et al. 2018 0.50 (0.26, 0.96) 3.34
Gory B. et al. 2018 0.30 (0.10, 0.89) 1.53
Bourcier et al. 2018 1.20 (0.50, 2.90) 2.19
Bellwald et al. 2017 1.33 (0.73, 2.43) 3.69
Wang et al. 2017 0.93 (0.54, 1.60) 4.14
Park et al. 2017 0.58 (0.35, 0.97) 4.48
Rebello et al. 2016 0.62 (0.34, 1.12) 3.75
Broeg-Morvay et al. 2016 1.33 (0.46, 3.83) 1.63
Villwock.et al. 2016 0.81 (0.74, 0.88) 9.28
Brinjikji et al. 2014 0.86 (0.59, 1.24) 5.97
Díaz-Pérez et al. 2018 0.38 (0.11, 1.32) 1.23
Hassan et al.2020 0.76 (0.43, 1.87) 2.85
Subtotal (I-squared = 40.8%, p = 0.041) 0.80 (0.67, 0.96) 57.41
Overall (I-squared = 53.6%, p = 0.000) 0.74 (0.64, 0.86) 100.00
NOTE: Weights are from random effects analysis
.5
1
1.5

**Supplemental Figure 30.** Forest plots of subgroup analysis assessing all mortality by ethnicity in unadjusted (A) and adjusted (B) analysis. ICA, internal carotid artery. [*]The acute intracranial vessel occlusion patients with (a) or without (b) concomitant ipsilateral ICA-occlusion or high-grade stenosis were investigated respectively in the study by Bücke et al. 2018.

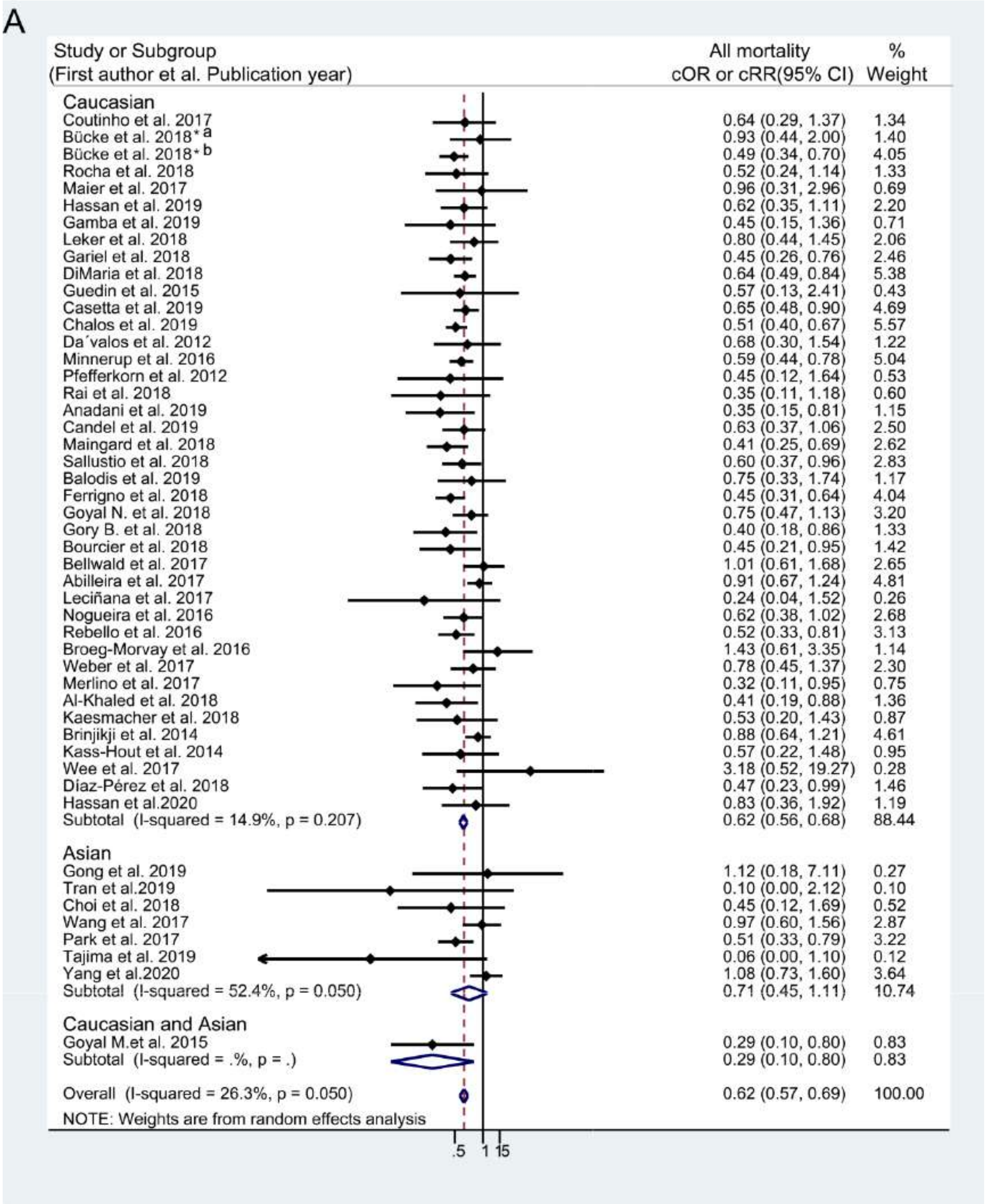

B

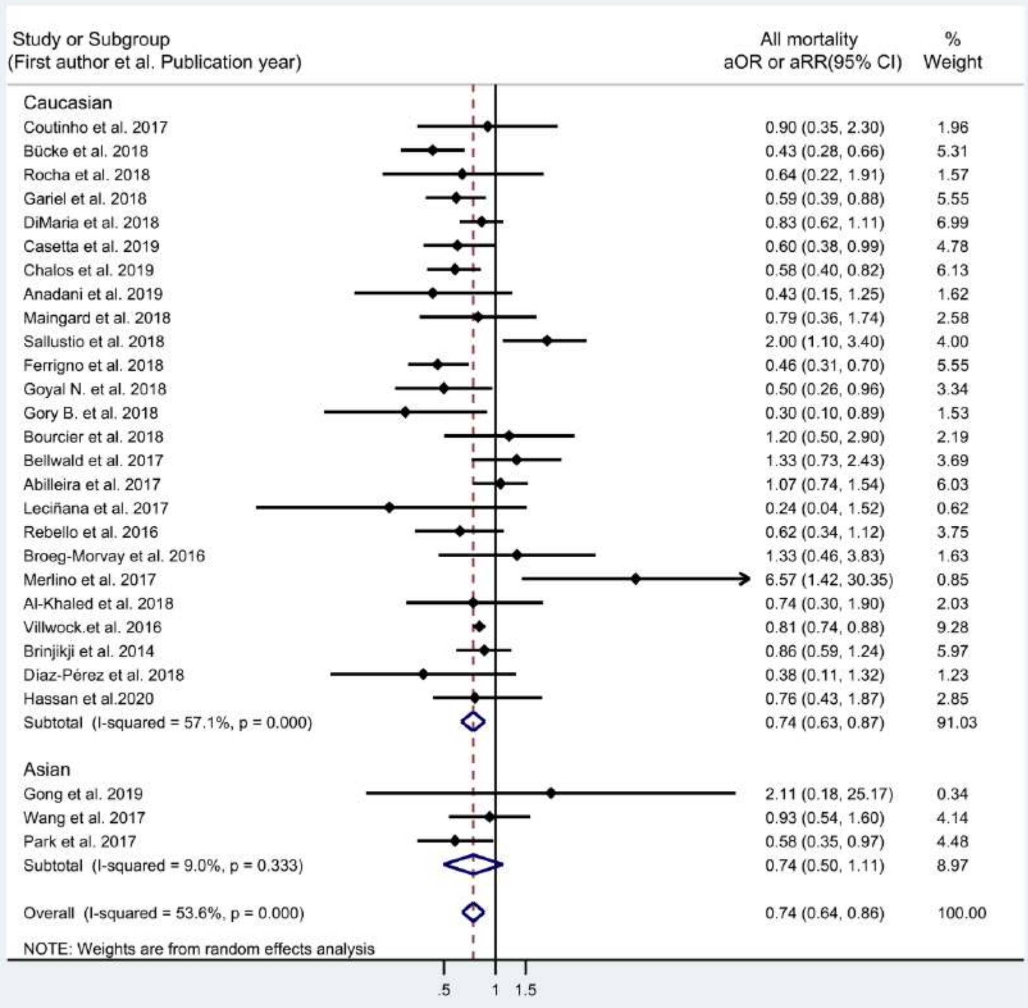
Study or Subgroup
(First author et al. Publication year)
All mortality
aOR or aRR(95% CI)
%
Weight
Caucasian
Coutinho et al. 2017 0.90 (0.35, 2.30) 1.96
Bücke et al. 2018 0.43 (0.28, 0.66) 5.31
Rocha et al. 2018 0.64 (0.22, 1.91) 1.57
Gariel et al. 2018 0.59 (0.39, 0.88) 5.55
DiMaria et al. 2018 0.83 (0.62, 1.11) 6.99
Casetta et al. 2019 0.60 (0.38, 0.99) 4.78
Chalos et al. 2019 0.58 (0.40, 0.82) 6.13
Anadani et al. 2019 0.43 (0.15, 1.25) 1.62
Maingard et al. 2018 0.79 (0.36, 1.74) 2.58
Sallustio et al. 2018 2.00 (1.10, 3.40) 4.00
Ferrigno et al. 2018 0.46 (0.31, 0.70) 5.55
Goyal N. et al. 2018 0.50 (0.26, 0.96) 3.34
Gory B. et al. 2018 0.30 (0.10, 0.89) 1.53
Bourcier et al. 2018 1.20 (0.50, 2.90) 2.19
Bellwald et al. 2017 1.33 (0.73, 2.43) 3.69
Abilleira et al. 2017 1.07 (0.74, 1.54) 6.03
Leciñana et al. 2017 0.24 (0.04, 1.52) 0.62
Rebello et al. 2016 0.62 (0.34, 1.12) 3.75
Broeg-Morvay et al. 2016 1.33 (0.46, 3.83) 1.63
Merlino et al. 2017 6.57 (1.42, 30.35) 0.85
Al-Khaled et al. 2018 0.74 (0.30, 1.90) 2.03
Villwock.et al. 2016 0.81 (0.74, 0.88) 9.28
Brinjikji et al. 2014 0.86 (0.59, 1.24) 5.97
Díaz-Pérez et al. 2018 0.38 (0.11, 1.32) 1.23
Hassan et al.2020 0.76 (0.43, 1.87) 2.85
Subtotal (I-squared = 57.1%, p = 0.000) 0.74 (0.63, 0.87) 91.03
Asian
Gong et al. 2019 2.11 (0.18, 25.17) 0.34
Wang et al. 2017 0.93 (0.54, 1.60) 4.14
Park et al. 2017 0.58 (0.35, 0.97) 4.48
Subtotal (I-squared = 9.0%, p = 0.333) 0.74 (0.50, 1.11) 8.97
Overall (I-squared = 53.6%, p = 0.000) 0.74 (0.64, 0.86) 100.00
NOTE: Weights are from random effects analysis
.5
1
1.5

**Supplemental Figure 31.** Forest plots of studies assessing successful reperfusion in patients with tandem lesion.

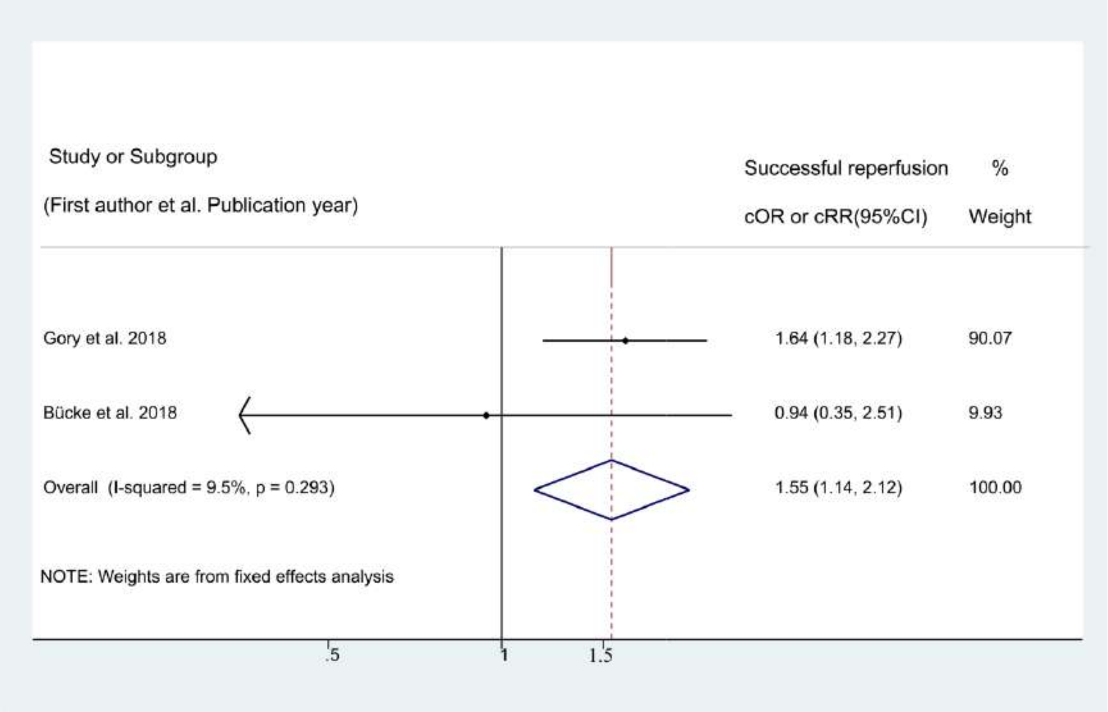

**Supplemental Figure 32.** Forest plots of subgroup analysis assessing successful reperfusion by ethnicity in unadjusted (A) and adjusted (B) analysis. ICA, internal carotid artery. [*]The acute intracranial vessel occlusion patients with (a) or without (b) concomitant ipsilateral ICA-occlusion or high-grade stenosis were investigated respectively in the study by Bücke et al. 2018.

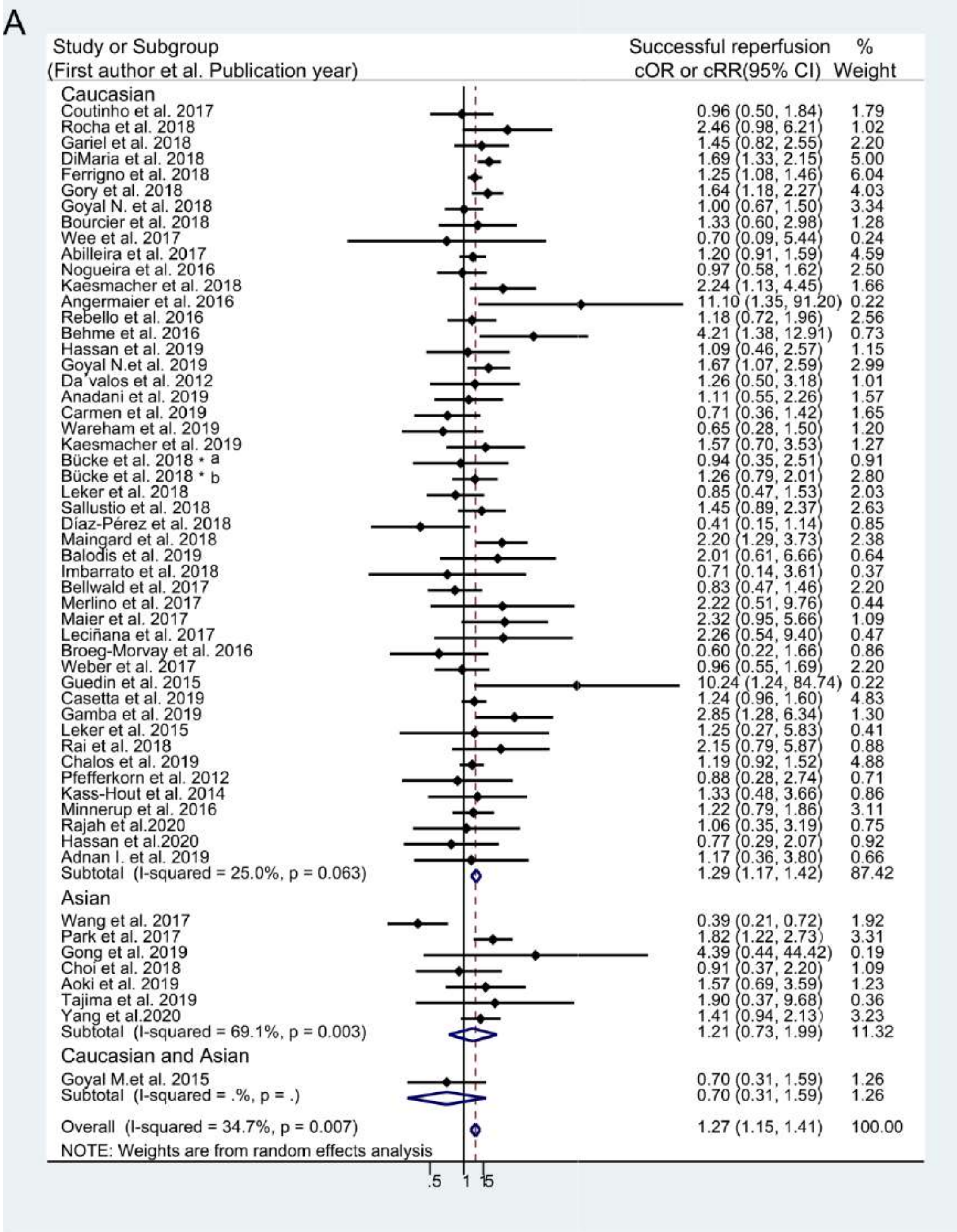

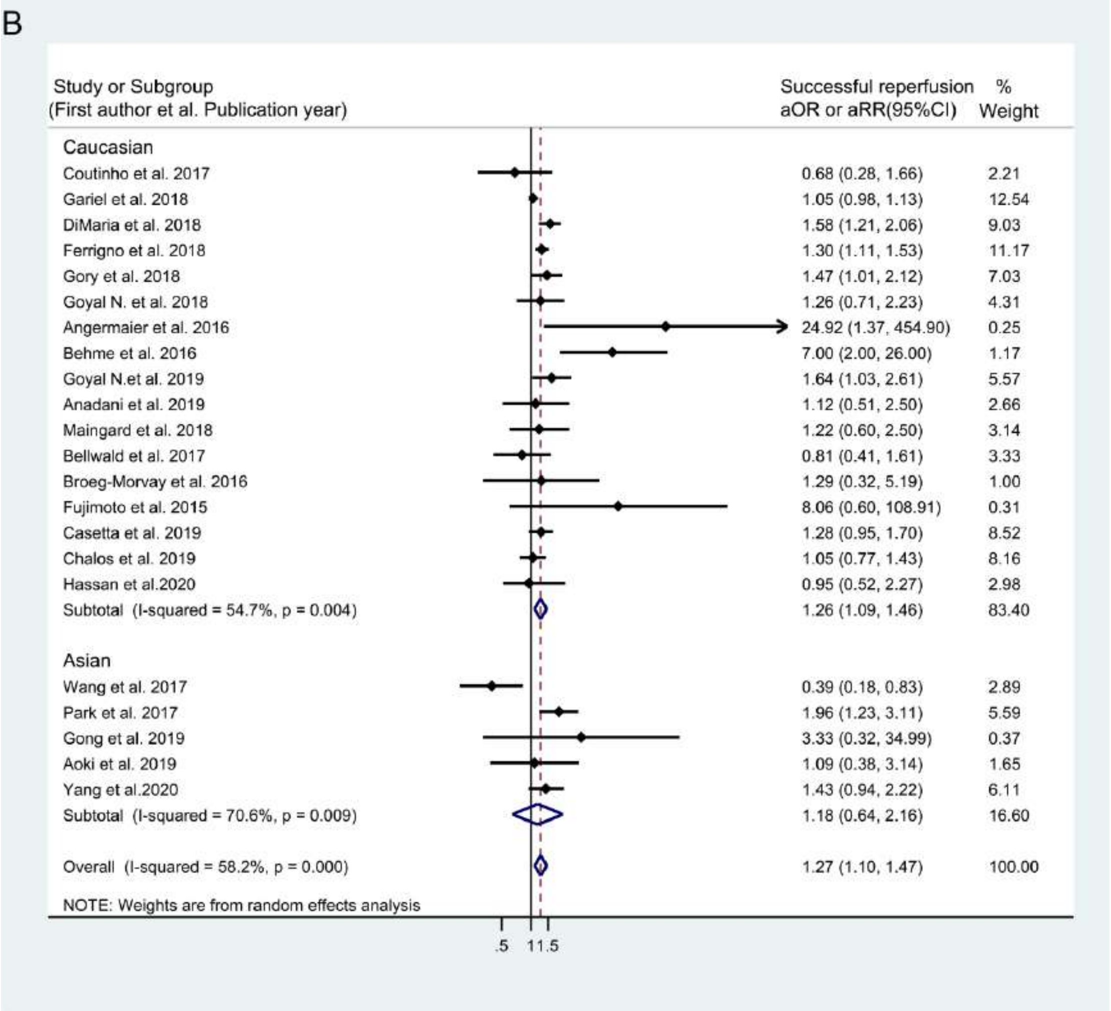
B
Study or Subgroup
(First author et al. Publication year)
Successful reperfusion
aOR or aRR(95%CI)
%
Weight
Caucasian
Coutinho et al. 2017 0.68 (0.28, 1.66) 2.21
Gariel et al. 2018 1.05 (0.98, 1.13) 12.54
DiMaria et al. 2018 1.58 (1.21, 2.06) 9.03
Ferrigno et al. 2018 1.30 (1.11, 1.53) 11.17
Gory et al. 2018 1.47 (1.01, 2.12) 7.03
Goyal N. et al. 2018 1.26 (0.71, 2.23) 4.31
Angermaier et al. 2016 24.92 (1.37, 454.90) 0.25
Behme et al. 2016 7.00 (2.00, 26.00) 1.17
Goyal N.et al. 2019 1.64 (1.03, 2.61) 5.57
Anadani et al. 2019 1.12 (0.51, 2.50) 2.66
Maingard et al. 2018 1.22 (0.60, 2.50) 3.14
Bellwald et al. 2017 0.81 (0.41, 1.61) 3.33
Broeg-Morvay et al. 2016 1.29 (0.32, 5.19) 1.00
Fujimoto et al. 2015 8.06 (0.60, 108.91) 0.31
Casetta et al. 2019 1.28 (0.95, 1.70) 8.52
Chalos et al. 2019 1.05 (0.77, 1.43) 8.16
Hassan et al.2020 0.95 (0.52, 2.27) 2.98
Subtotal (I-squared = 54.7%, p = 0.004) 1.26 (1.09, 1.46) 83.40
Asian
Wang et al. 2017 0.39 (0.18, 0.83) 2.89
Park et al. 2017 1.96 (1.23, 3.11) 5.59
Gong et al. 2019 3.33 (0.32, 34.99) 0.37
Aoki et al. 2019 1.09 (0.38, 3.14) 1.65
Yang et al.2020 1.43 (0.94, 2.22) 6.11
Subtotal (I-squared = 70.6%, p = 0.009) 1.18 (0.64, 2.16) 16.60
Overall (I-squared = 58.2%, p = 0.000) 1.27 (1.10, 1.47) 100.00
NOTE: Weights are from random effects analysis
.5 1 1.5

**Supplemental Figure 33.** Forest plots of studies assessing complete reperfusion in unadjusted (A) and adjusted (B) analysis.

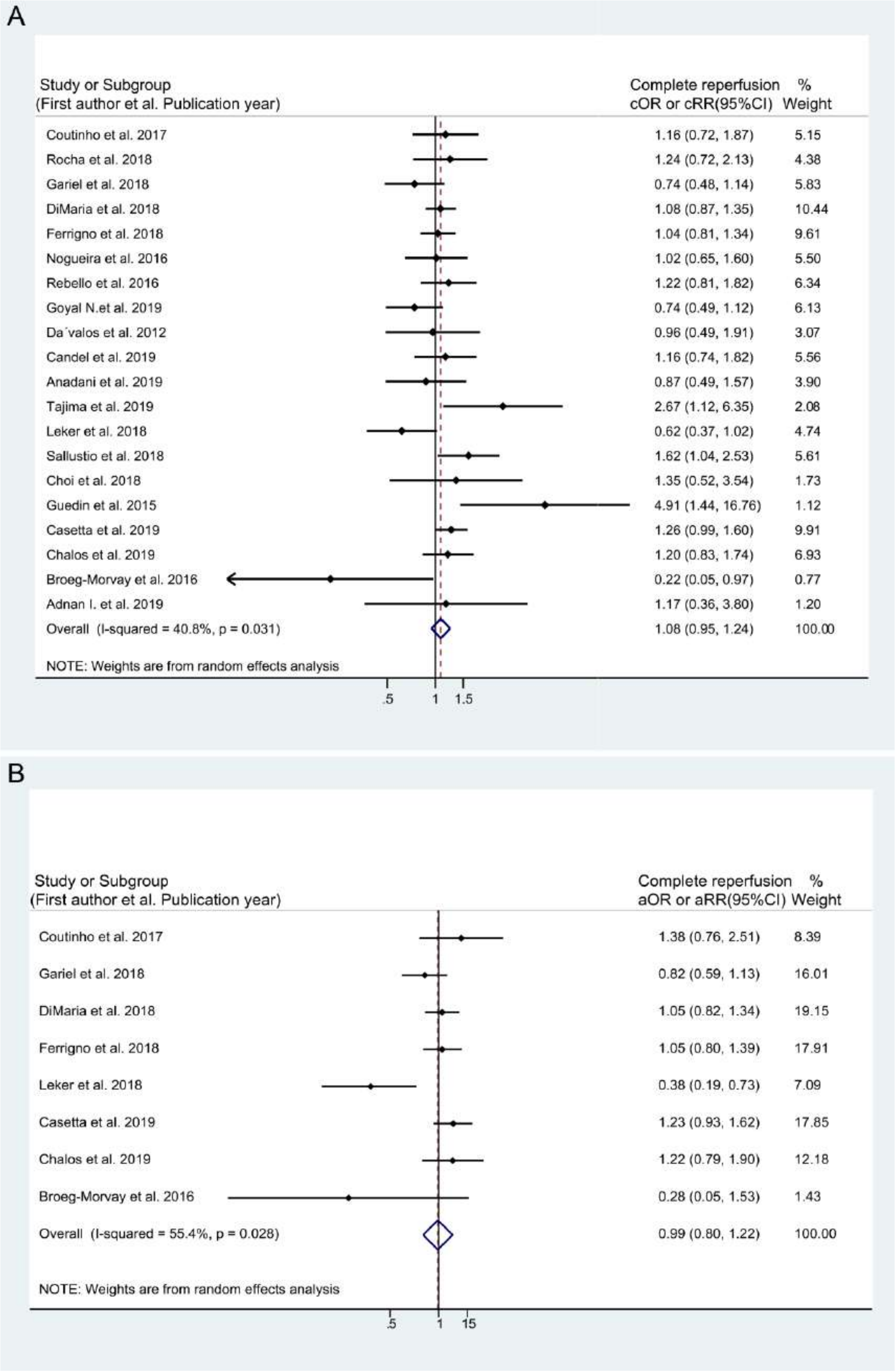

**Supplemental Figure 34.** Forest plots of subgroup analysis assessing successful reperfusion by location of occluded artery in unadjusted (A) and adjusted (B) analysis. ICA, internal carotid artery. [*]The acute intracranial vessel occlusion patients with (a) or without (b) concomitant ipsilateral ICA-occlusion or high-grade stenosis were investigated respectively in the study by Bücke et al. 2018.

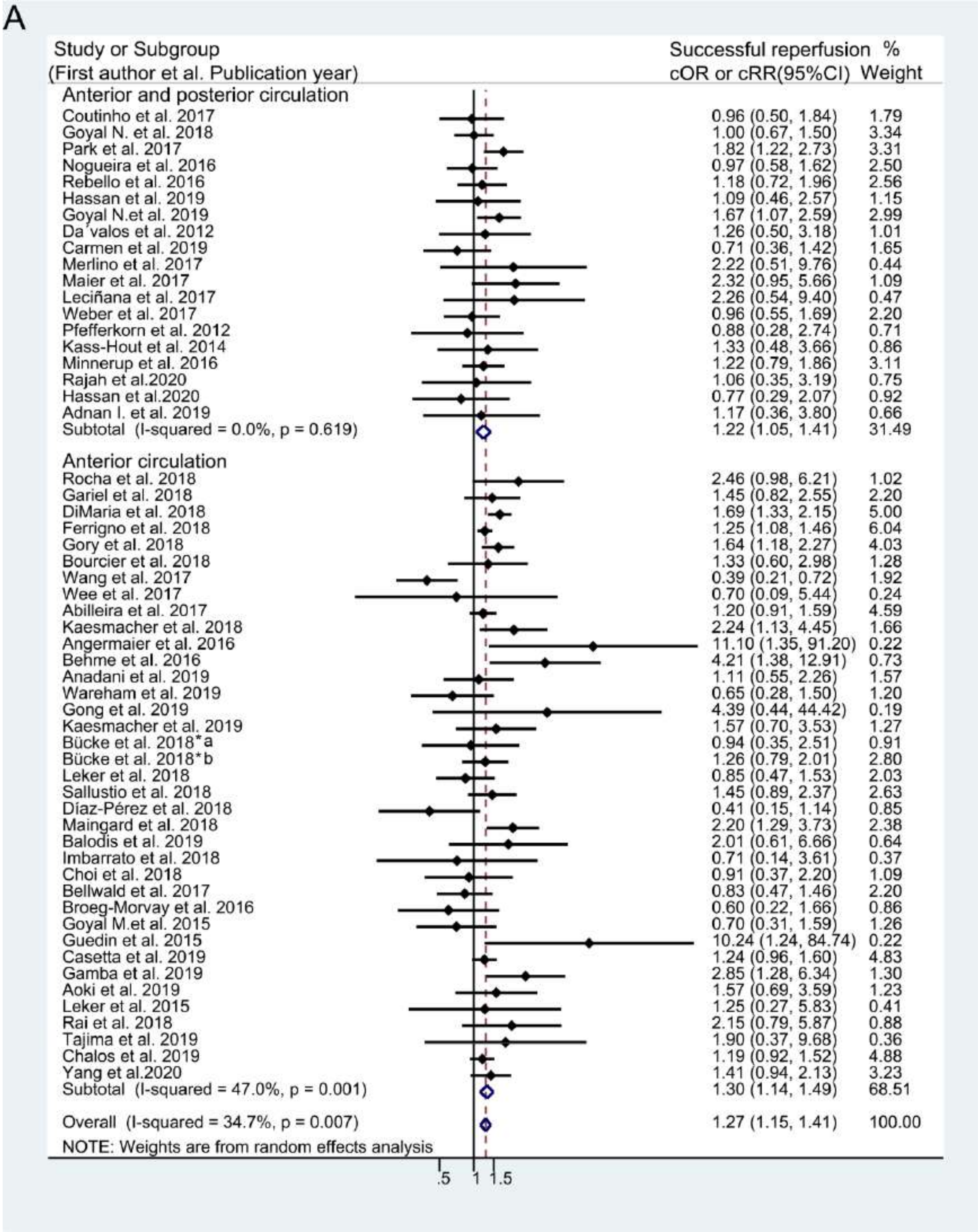

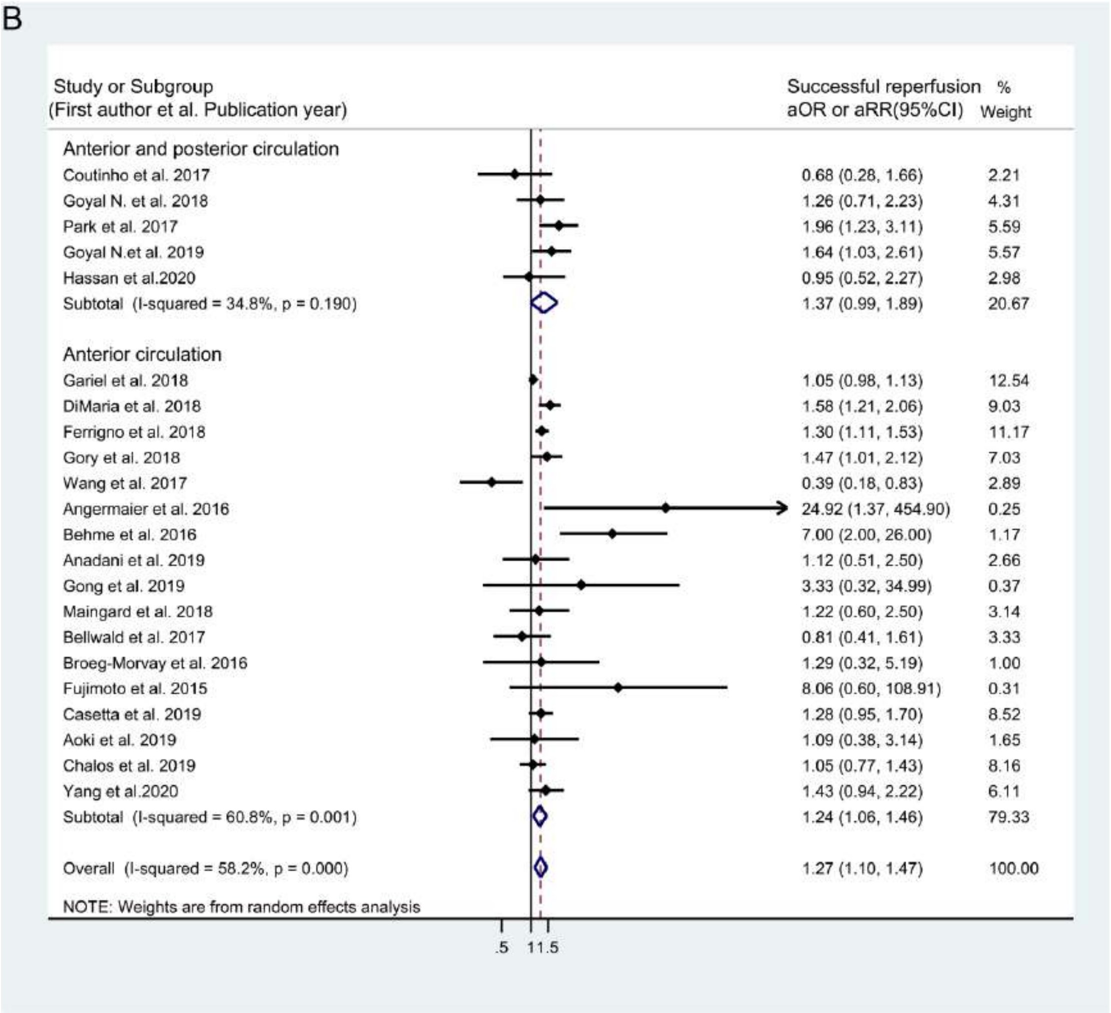
B
Study or Subgroup
(First author et al. Publication year)
Successful reperfusion
aOR or aRR(95%CI)
% Weight
Anterior and posterior circulation
Coutinho et al. 2017 0.68 (0.28, 1.66) 2.21
Goyal N. et al. 2018 1.26 (0.71, 2.23) 4.31
Park et al. 2017 1.96 (1.23, 3.11) 5.59
Goyal N.et al. 2019 1.64 (1.03, 2.61) 5.57
Hassan et al.2020 0.95 (0.52, 2.27) 2.98
Subtotal (I-squared = 34.8%, p = 0.190) 1.37 (0.99, 1.89) 20.67
Anterior circulation
Gariel et al. 2018 1.05 (0.98, 1.13) 12.54
DiMaria et al. 2018 1.58 (1.21, 2.06) 9.03
Ferrigno et al. 2018 1.30 (1.11, 1.53) 11.17
Gory et al. 2018 1.47 (1.01, 2.12) 7.03
Wang et al. 2017 0.39 (0.18, 0.83) 2.89
Angermaier et al. 2016 24.92 (1.37, 454.90) 0.25
Behme et al. 2016 7.00 (2.00, 26.00) 1.17
Anadani et al. 2019 1.12 (0.51, 2.50) 2.66
Gong et al. 2019 3.33 (0.32, 34.99) 0.37
Maingard et al. 2018 1.22 (0.60, 2.50) 3.14
Bellwald et al. 2017 0.81 (0.41, 1.61) 3.33
Broeg-Morvay et al. 2016 1.29 (0.32, 5.19) 1.00
Fujimoto et al. 2015 8.06 (0.60, 108.91) 0.31
Casetta et al. 2019 1.28 (0.95, 1.70) 8.52
Aoki et al. 2019 1.09 (0.38, 3.14) 1.65
Chalos et al. 2019 1.05 (0.77, 1.43) 8.16
Yang et al.2020 1.43 (0.94, 2.22) 6.11
Subtotal (I-squared = 60.8%, p = 0.001) 1.24 (1.06, 1.46) 79.33
Overall (I-squared = 58.2%, p = 0.000) 1.27 (1.10, 1.47) 100.00
NOTE: Weights are from random effects analysis
.5 1 1.5

**Supplemental Figure 35.** Forest plots of subgroup analysis assessing successful reperfusion by study type in unadjusted (A) and adjusted (B) analysis. ICA, internal carotid artery. [*]The acute intracranial vessel occlusion patients with (a) or without (b) concomitant ipsilateral ICA-occlusion or high-grade stenosis were investigated respectively in the study by Bücke et al. 2018.

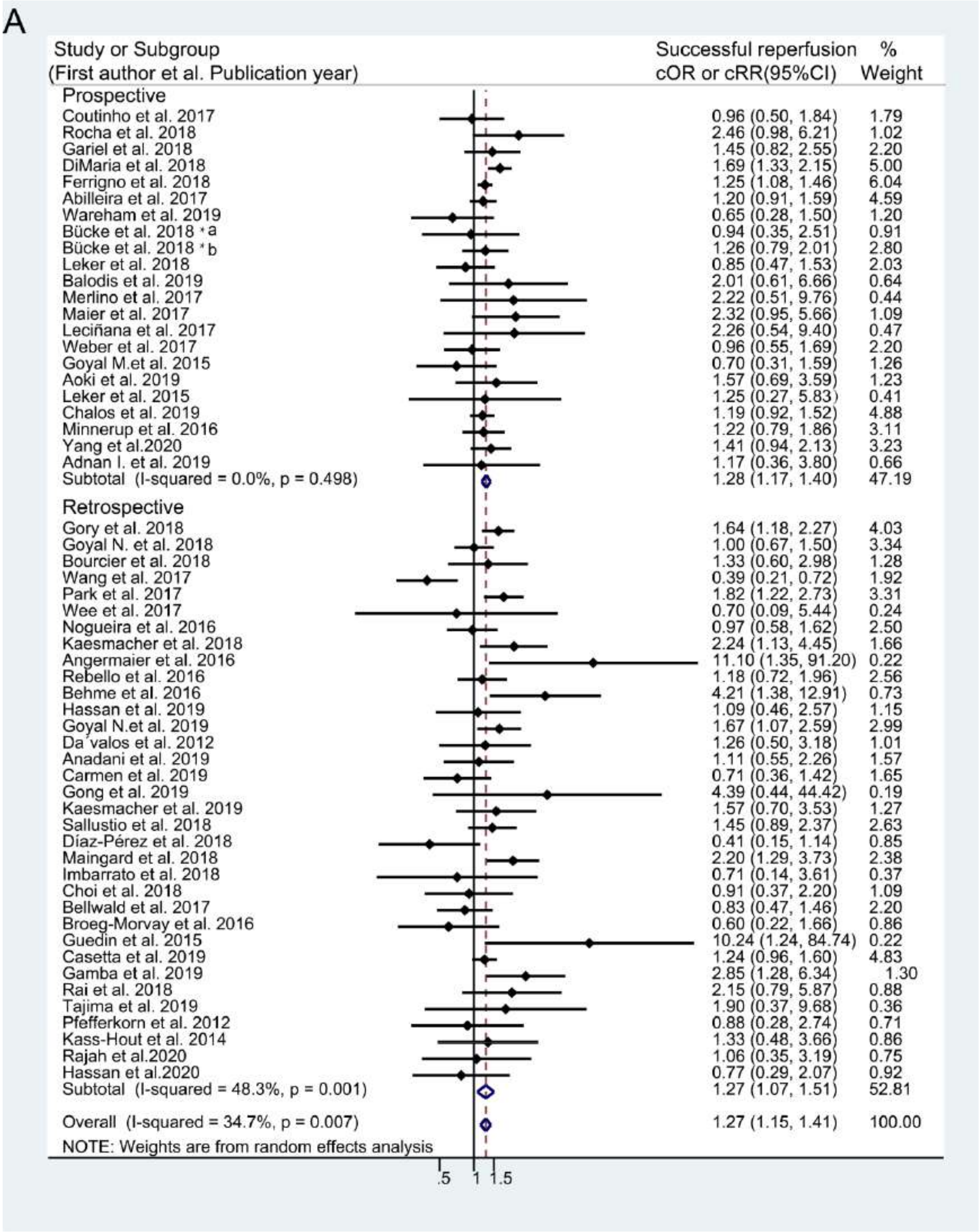

B

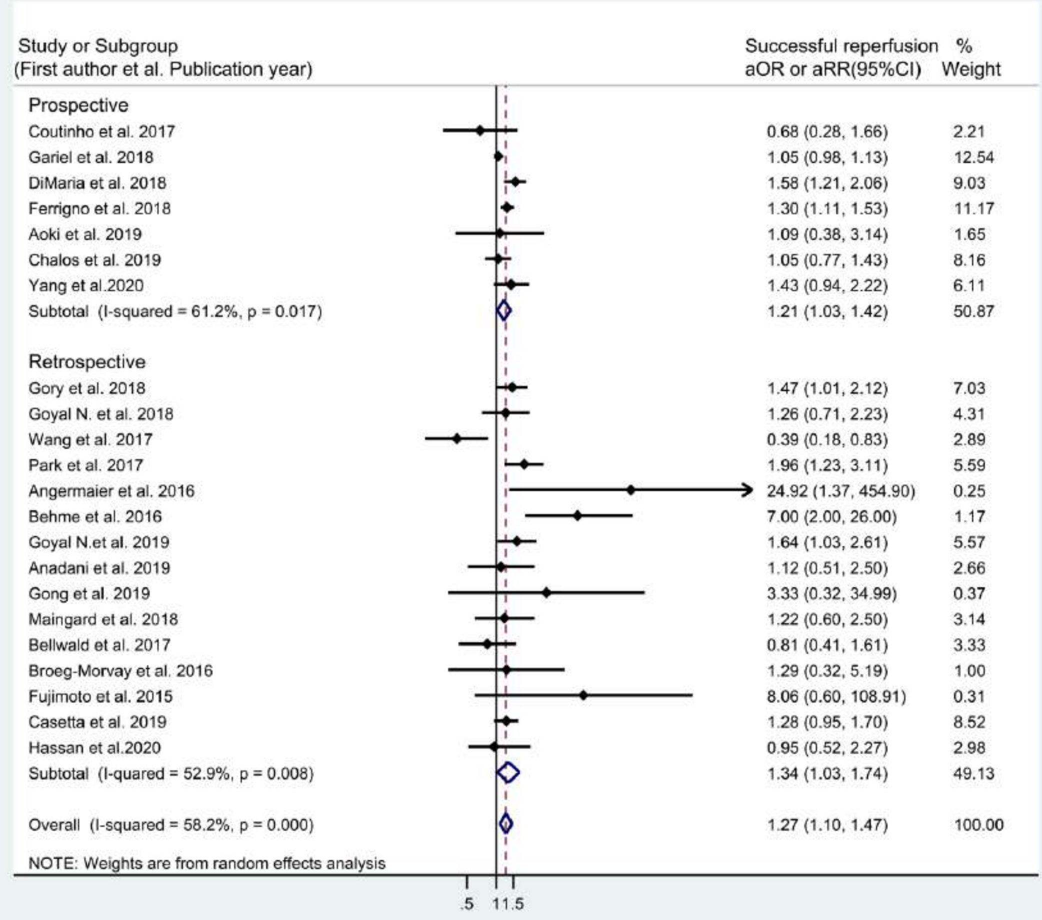

Study or Subgroup
(First author et al. Publication year)
Successful reperfusion
aOR or aRR(95%CI)
%
Weight
Prospective
Coutinho et al. 2017 0.68 (0.28, 1.66) 2.21
Gariel et al. 2018 1.05 (0.98, 1.13) 12.54
DiMaria et al. 2018 1.58 (1.21, 2.06) 9.03
Ferrigno et al. 2018 1.30 (1.11, 1.53) 11.17
Aoki et al. 2019 1.09 (0.38, 3.14) 1.65
Chalos et al. 2019 1.05 (0.77, 1.43) 8.16
Yang et al.2020 1.43 (0.94, 2.22) 6.11
Subtotal (I-squared = 61.2%, p = 0.017) 1.21 (1.03, 1.42) 50.87
Retrospective
Gory et al. 2018 1.47 (1.01, 2.12) 7.03
Goyal N. et al. 2018 1.26 (0.71, 2.23) 4.31
Wang et al. 2017 0.39 (0.18, 0.83) 2.89
Park et al. 2017 1.96 (1.23, 3.11) 5.59
Angermaier et al. 2016 24.92 (1.37, 454.90) 0.25
Behme et al. 2016 7.00 (2.00, 26.00) 1.17
Goyal N.et al. 2019 1.64 (1.03, 2.61) 5.57
Anadani et al. 2019 1.12 (0.51, 2.50) 2.66
Gong et al. 2019 3.33 (0.32, 34.99) 0.37
Maingard et al. 2018 1.22 (0.60, 2.50) 3.14
Bellwald et al. 2017 0.81 (0.41, 1.61) 3.33
Broeg-Morvay et al. 2016 1.29 (0.32, 5.19) 1.00
Fujimoto et al. 2015 8.06 (0.60, 108.91) 0.31
Casetta et al. 2019 1.28 (0.95, 1.70) 8.52
Hassan et al.2020 0.95 (0.52, 2.27) 2.98
Subtotal (I-quared = 52.9%, p = 0.008) 1.34 (1.03, 1.74) 49.13
Overall (I-squared = 58.2%, p = 0.000) 1.27 (1.10, 1.47) 100.00
NOTE: Weights are from random effects analysis
.5
1
1.5

**Supplemental Figure 36.** Forest plots of subgroup analysis assessing complete reperfusion by location of occluded artery in unadjusted (A) and adjusted (B) analysis.

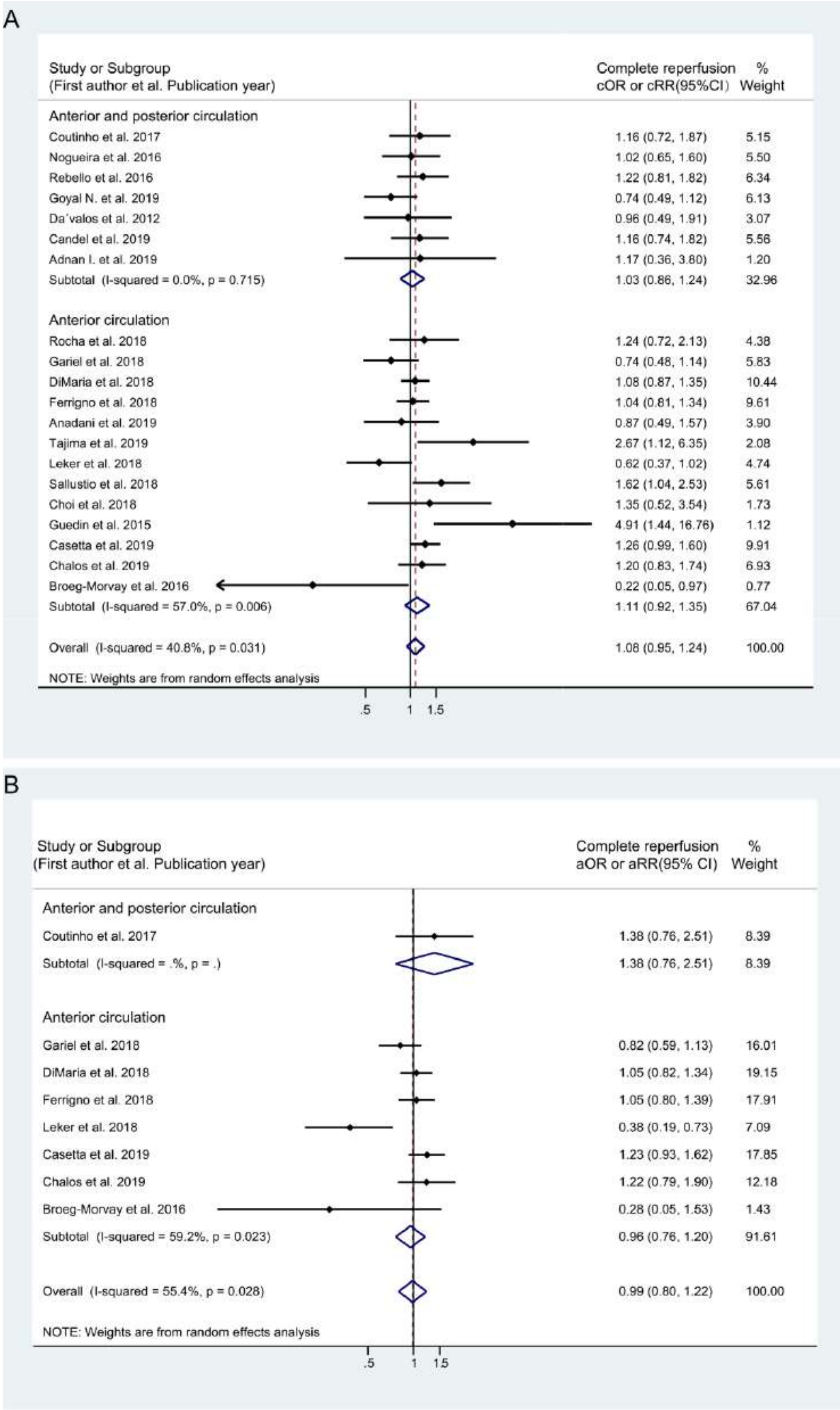

**Supplemental Figure 37.** Forest plots of subgroup analysis assessing complete reperfusion by study type in unadjusted (A) and adjusted (B) analysis.

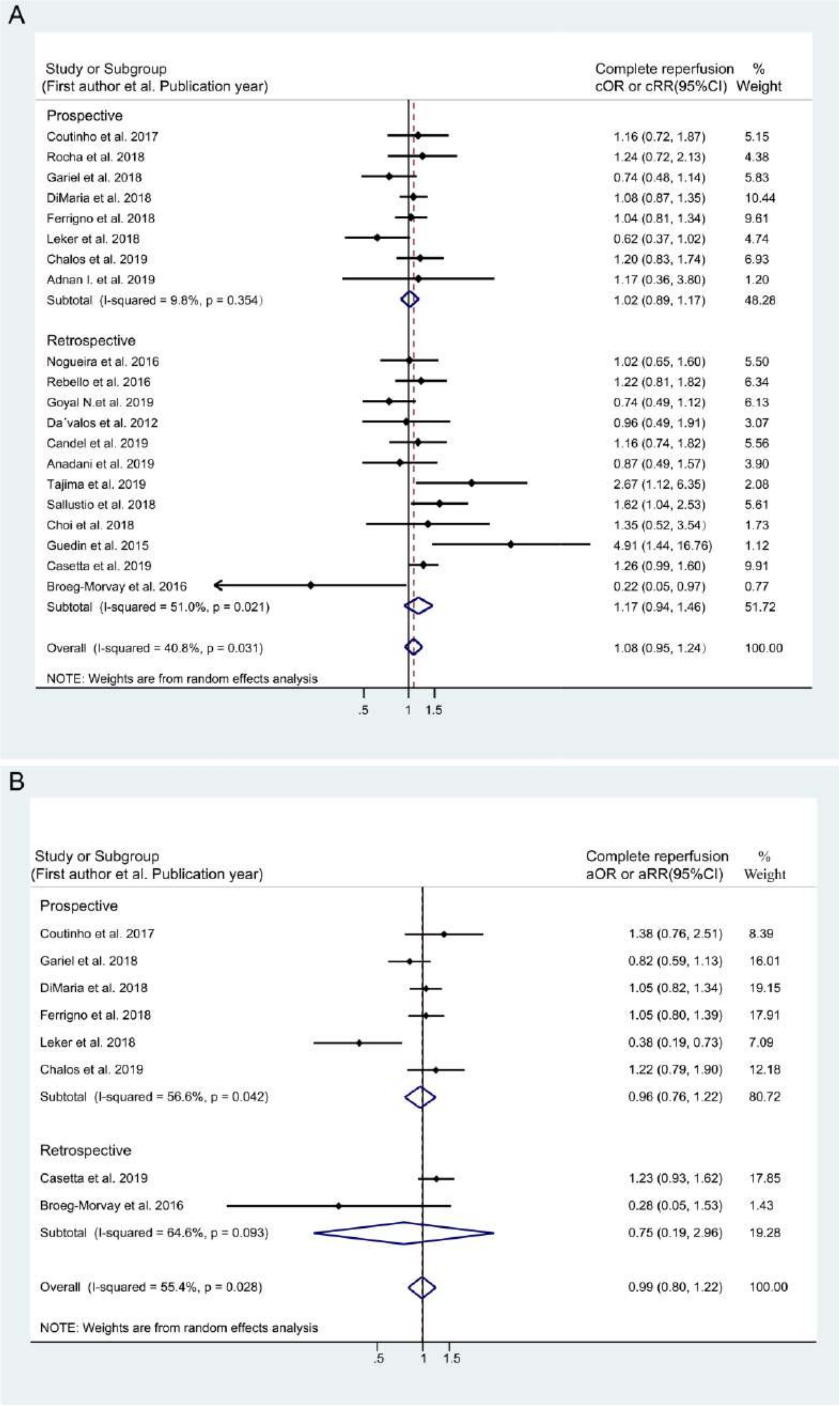

**Supplemental Figure 38.** Forest plots of subgroup analysis assessing complete reperfusion by ethnicity in unadjusted analysis.

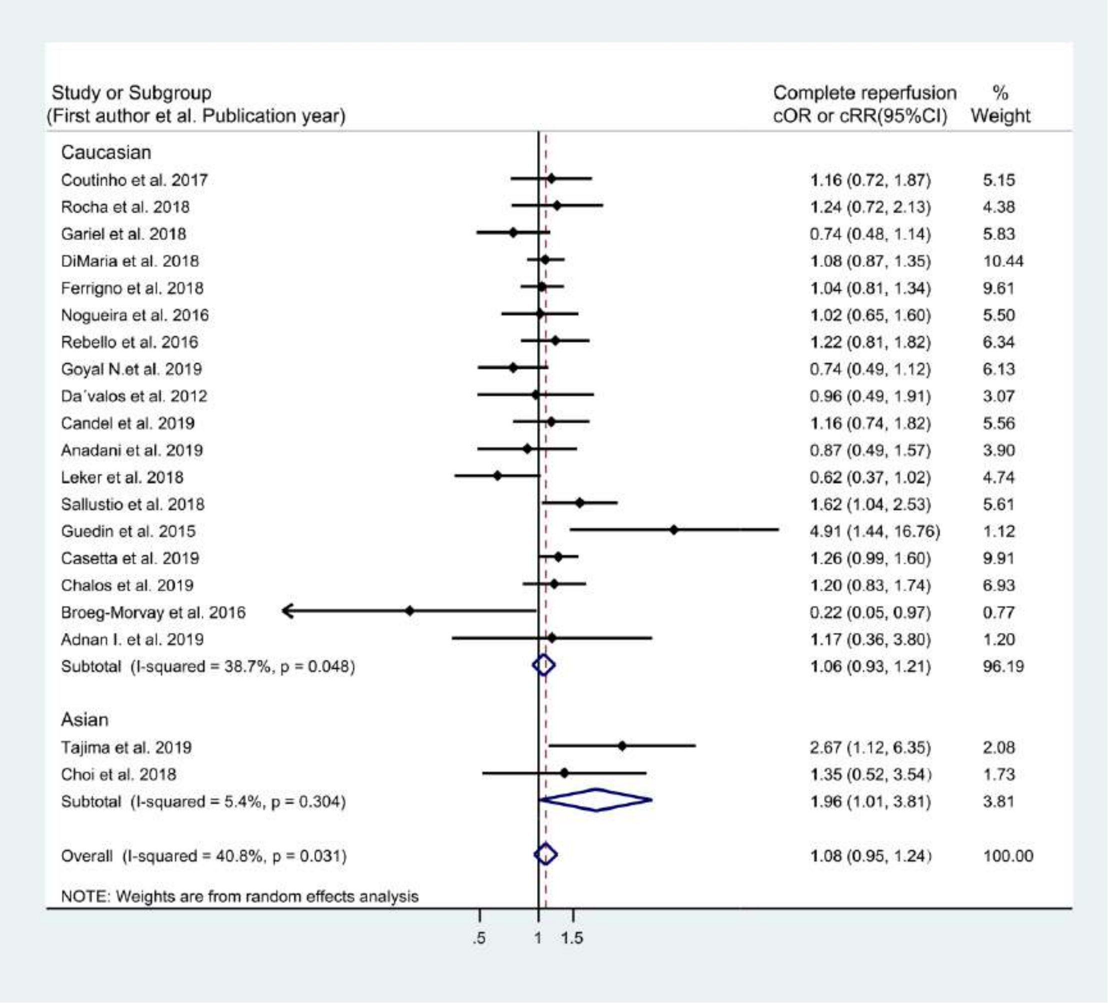

**Supplemental Figure 39.** Forest plots of studies assessing any ICH in unadjusted (A) and adjusted (B) analysis. ICH, intracranial hemorrhage; sICH, symptomatic intracranial hemorrhage; aICH, asymptomatic intracranial hemorrhage; ICA, internal carotid artery. *The acute intracranial vessel occlusion patients with (a) or without (b) concomitant ipsilateral ICA-occlusion or high-grade stenosis were investigated respectively in the study by Bücke et al. 2018. #The outcomes of sICH (c) and aICH (d) were investigated respectively in the studies of Hassan et al. 2019, Balodis et al. 2019, Broeg-Morvay et al. 2016, Bellwald et al. 2017, Wang et al. 2017, Yang et al.2020 and Hassan et al.2020

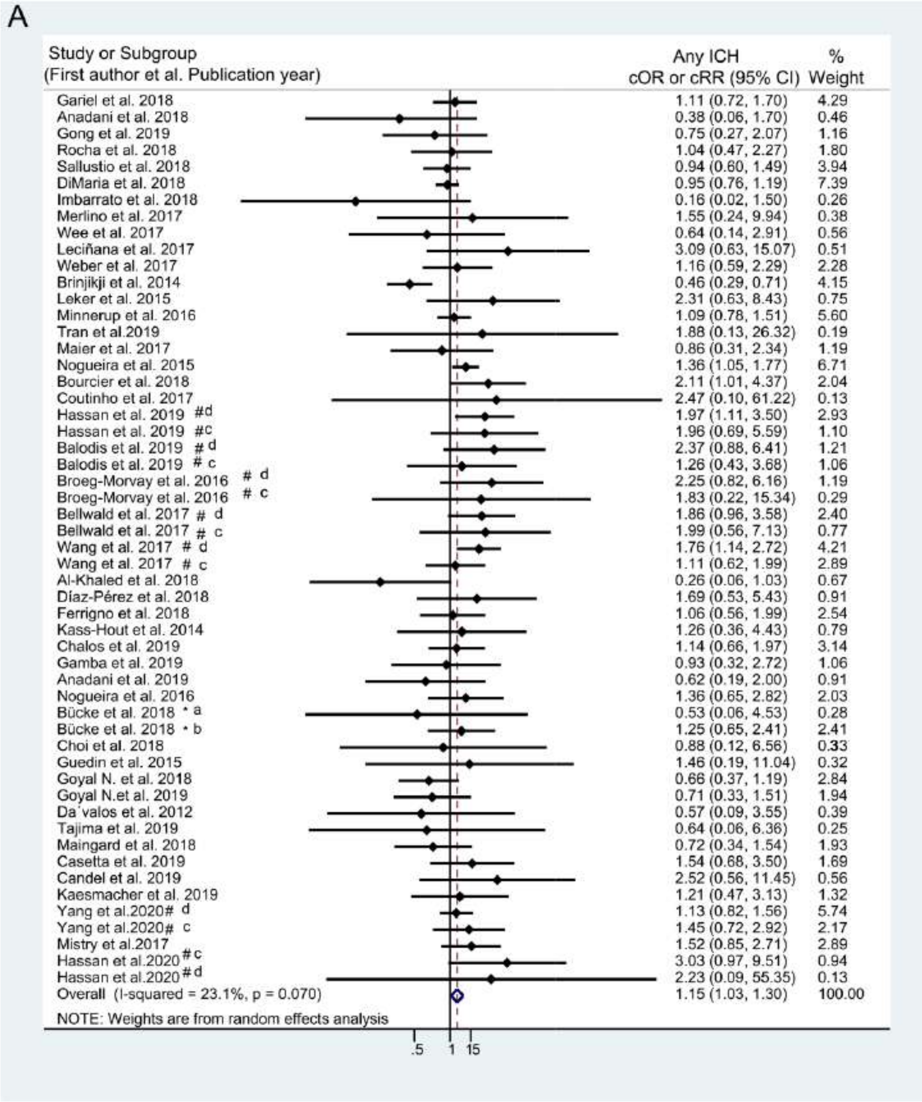

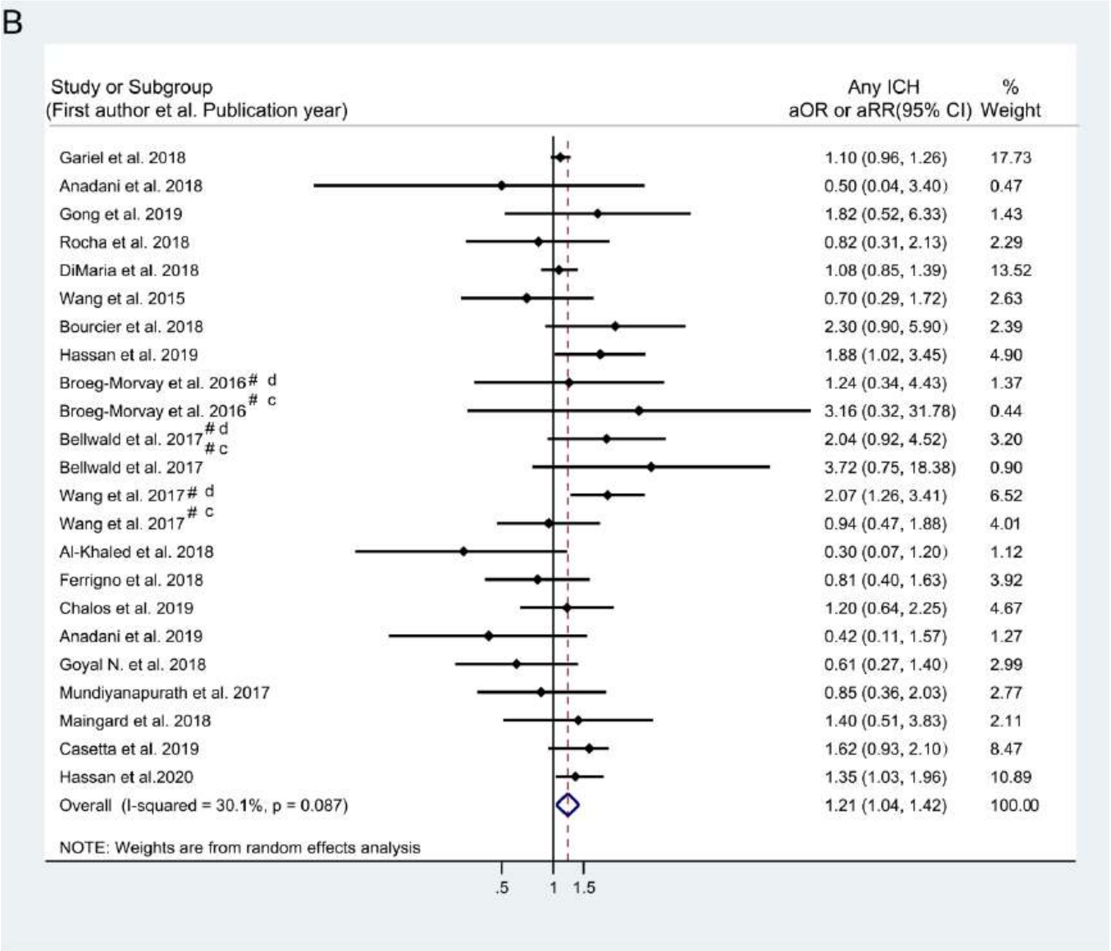
B
Study or Subgroup
(First author et al. Publication year)
Any ICH
aOR or aRR(95% CI)
%
Weight
Gariel et al. 2018 1.10 (0.96, 1.26) 17.73
Anadani et al. 2018 0.50 (0.04, 3.40) 0.47
Gong et al. 2019 1.82 (0.52, 6.33) 1.43
Rocha et al. 2018 0.82 (0.31, 2.13) 2.29
DiMaria et al. 2018 1.08 (0.85, 1.39) 13.52
Wang et al. 2015 0.70 (0.29, 1.72) 2.63
Bourcier et al. 2018 2.30 (0.90, 5.90) 2.39
Hassan et al. 2019 1.88 (1.02, 3.45) 4.90
Broeg-Morvay et al. 2016# d 1.24 (0.34, 4.43) 1.37
Broeg-Morvay et al. 2016# c 3.16 (0.32, 31.78) 0.44
Bellwald et al. 2017# d 2.04 (0.92, 4.52) 3.20
Bellwald et al. 2017# c 3.72 (0.75, 18.38) 0.90
Wang et al. 2017# d 2.07 (1.26, 3.41) 6.52
Wang et al. 2017# c 0.94 (0.47, 1.88) 4.01
Al-Khaled et al. 2018 0.30 (0.07, 1.20) 1.12
Ferrigno et al. 2018 0.81 (0.40, 1.63) 3.92
Chalos et al. 2019 1.20 (0.64, 2.25) 4.67
Anadani et al. 2019 0.42 (0.11, 1.57) 1.27
Goyal N. et al. 2018 0.61 (0.27, 1.40) 2.99
Mundiyanapurath et al. 2017 0.85 (0.36, 2.03) 2.77
Maingard et al. 2018 1.40 (0.51, 3.83) 2.11
Casetta et al. 2019 1.62 (0.93, 2.10) 8.47
Hassan et al.2020 1.35 (1.03, 1.96) 10.89
Overall (I-squared = 30.1%, p = 0.087) 1.21 (1.04, 1.42) 100.00
NOTE: Weights are from random effects analysis
.5
1
1.5

**Supplemental Figure 40.** Forest plots of studies assessing HT in unadjusted (A) and adjusted (B) analysis. HT, hemorrhagic transformation; PH, parenchymal hematoma; HI, hemorrhagic infarction. #The outcomes of PH (a) and HI (b) were investigated respectively in the study of Nogueira et al. 2015; The outcomes of HI-1 (c) and HI-2 (d) were investigated respectively in the studies of Coutinho et al. 2017, Sallustio et al. 2018 and Leciñana et al. 2017; The outcomes of PH-1(e) and PH-2 (f) were investigated respectively in the studies of Sallustio et al. 2018 and Leciñana et al. 2017. The outcome of HI-1 (c), HI-2 (d) and PH2 (f) were investigated respectively in the study of Mulder et al. 2016.

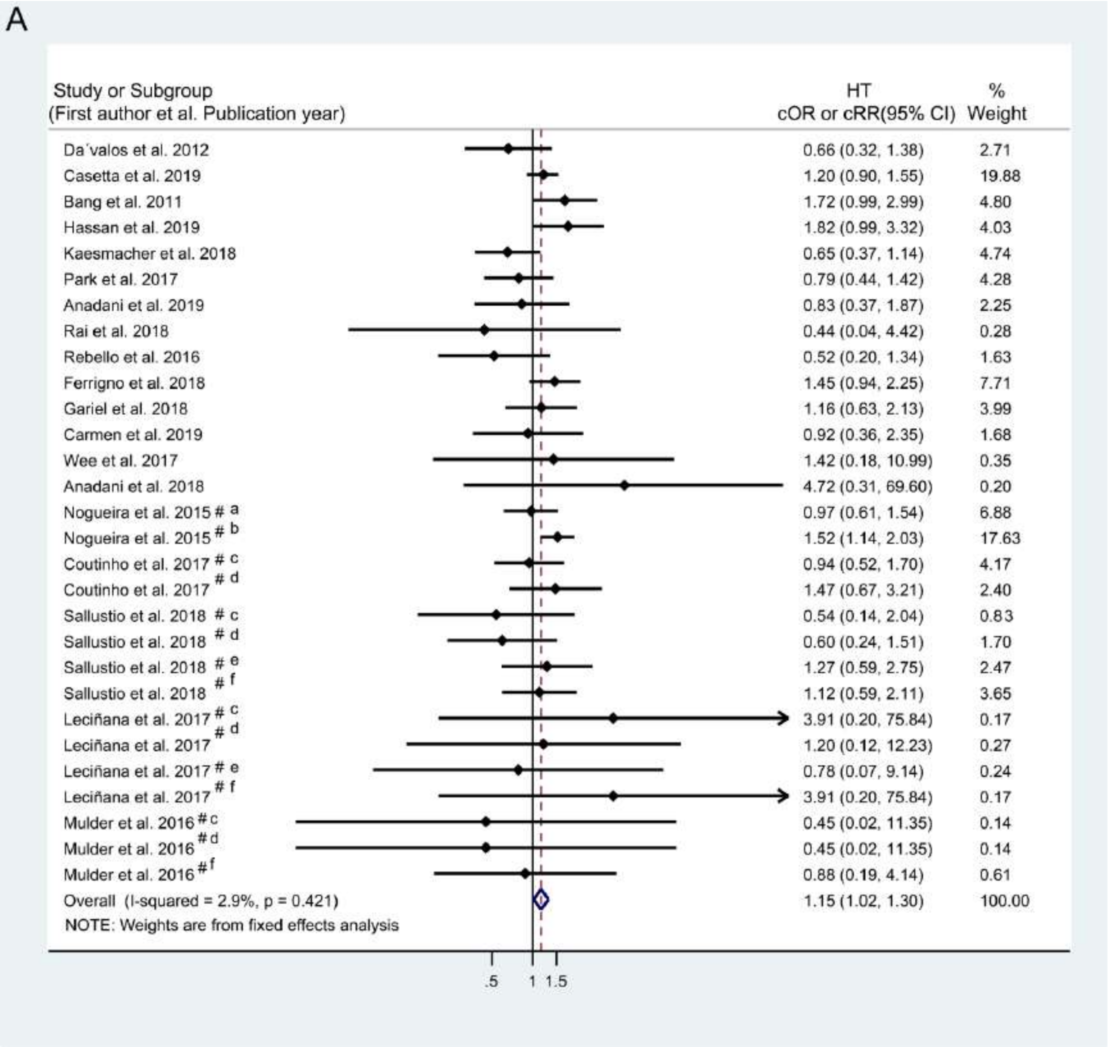

B

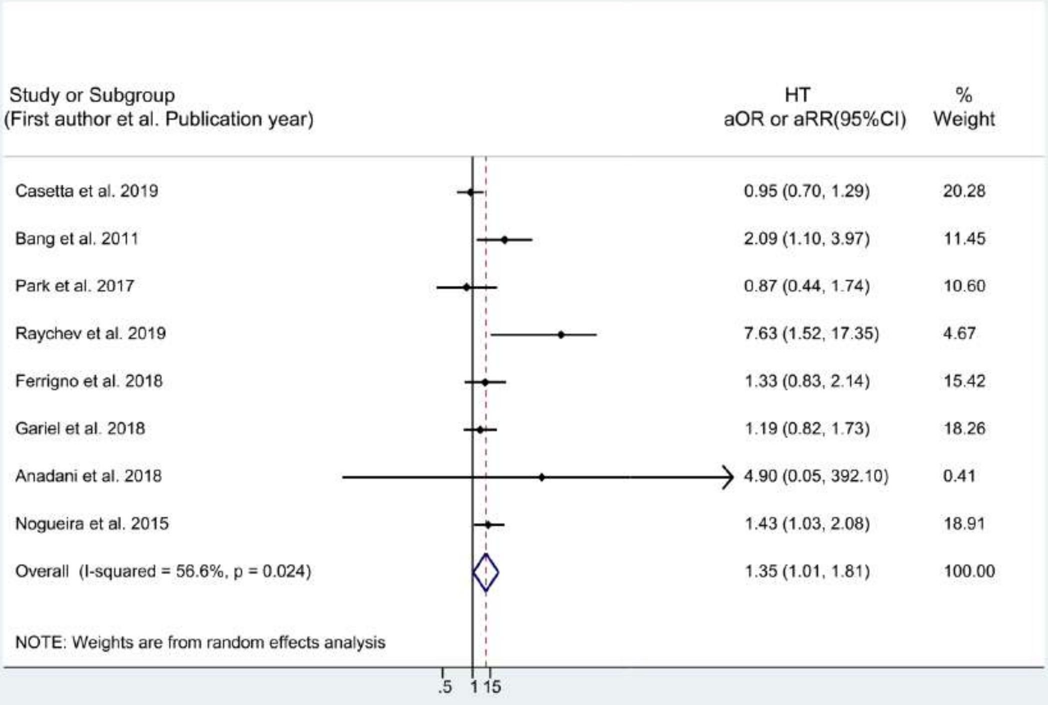
Study or Subgroup
(First author et al. Publication year)
HT
aOR or aRR(95%CI)
%
Weight
Casetta et al. 2019 0.95 (0.70, 1.29) 20.28
Bang et al. 2011 2.09 (1.10, 3.97) 11.45
Park et al. 2017 0.87 (0.44, 1.74) 10.60
Raychev et al. 2019 7.63 (1.52, 17.35) 4.67
Ferrigno et al. 2018 1.33 (0.83, 2.14) 15.42
Gariel et al. 2018 1.19 (0.82, 1.73) 18.26
Anadani et al. 2018 4.90 (0.05, 392.10) 0.41
Nogueira et al. 2015 1.43 (1.03, 2.08) 18.91
Overall (I-squared = 56.6%, p = 0.024) 1.35 (1.01, 1.81) 100.00
NOTE: Weights are from random effects analysis
.5
1
15

**Supplemental Figure 41.** Sensitivity analysis of the effect of individual studies on the cOR (A) and aOR (B) for HT. cOR, crude odds ratio; aOR, adjusted odds ratio. HT, hemorrhagic transformation; PH, parenchymal hematoma; HI, hemorrhagic infarction. #The outcomes of PH (a) and HI (b) were investigated respectively in the study of Nogueira et al. 2015; The outcomes of HI-1 (c) and HI-2 (d) were investigated respectively in the studies of Coutinho et al. 2017, Sallustio et al. 2018 and Leciñana et al. 2017; The outcomes of PH-1(e) and PH-2 (f) were investigated respectively in the studies of Sallustio et al. 2018 and Leciñana et al. 2017. The outcome of HI-1 (c), HI-2 (d) and PH2 (f) were investigated respectively in the study of Mulder et al. 2016.

A

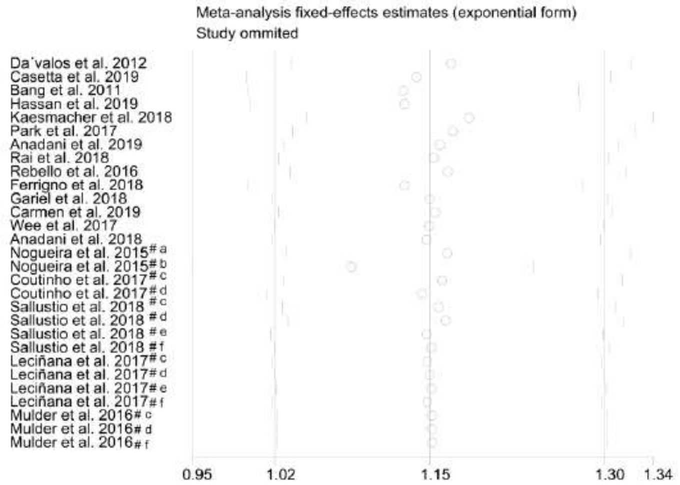


B

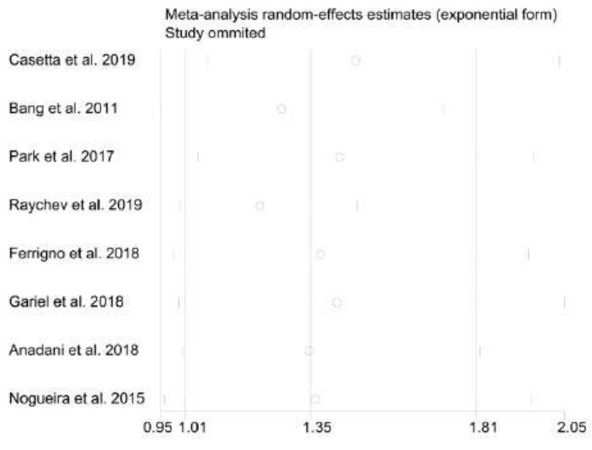

**Supplemental Figure 42.** Forest plots of studies assessing aICH in unadjusted (A) and adjusted (B) analysis. aICH, asymptomatic intracranial hemorrhage.

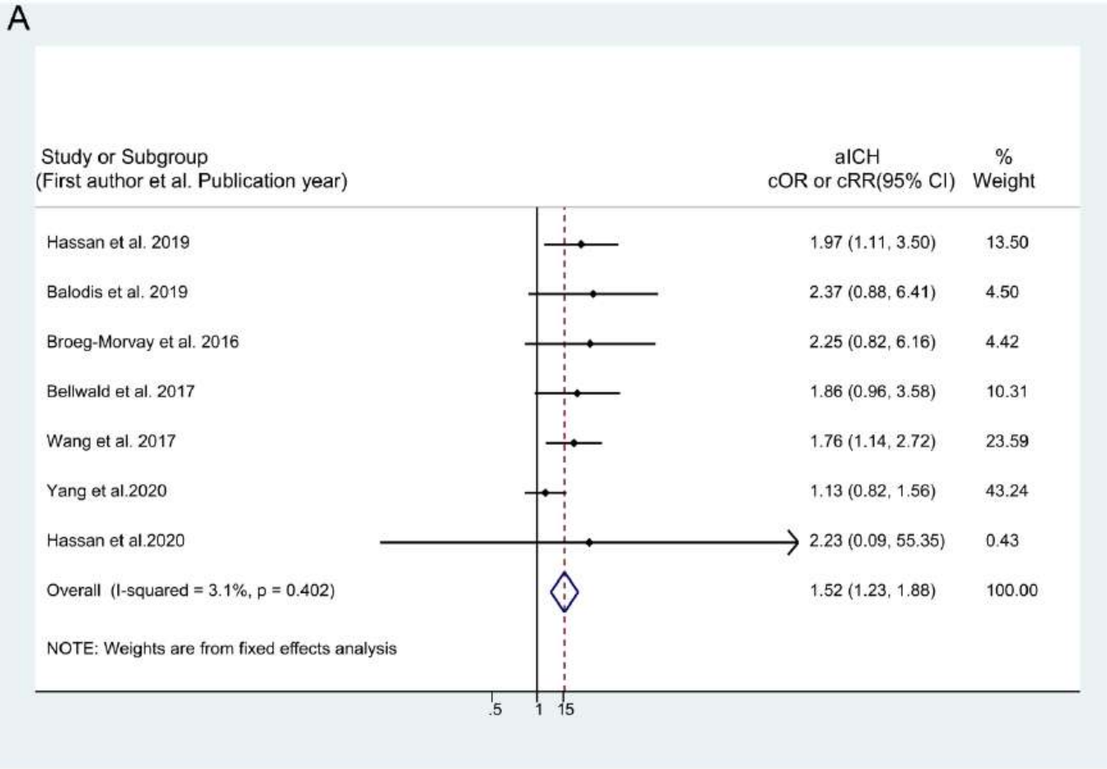


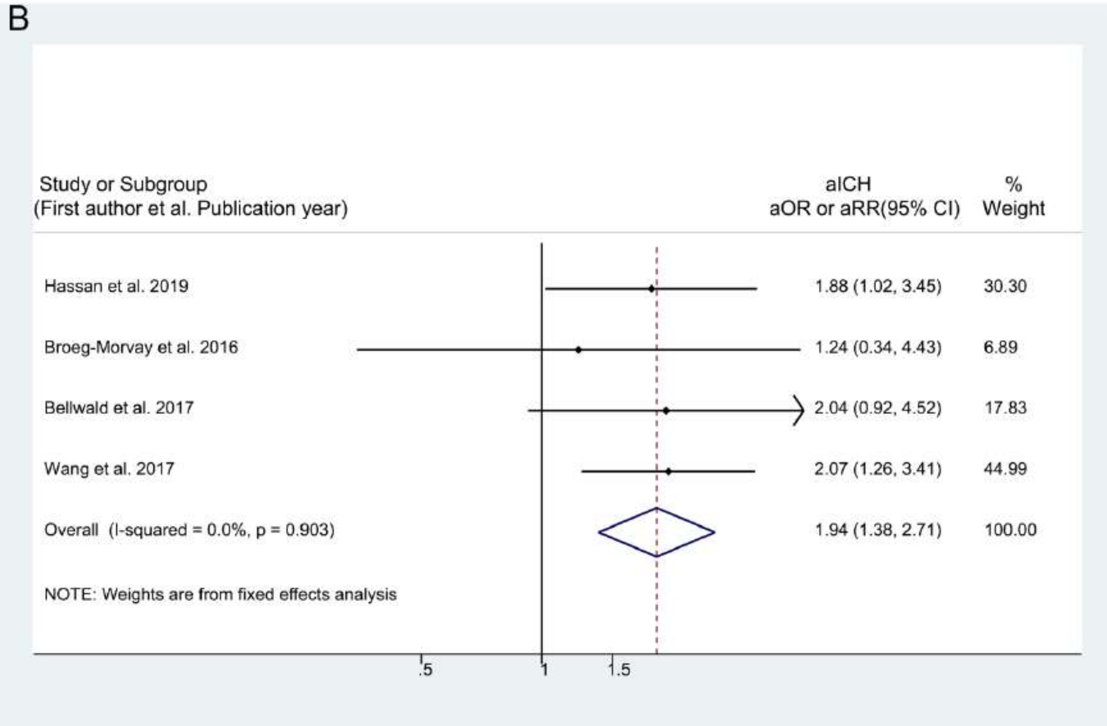

**Supplemental Figure 43.** Forest plots of studies assessing any bleeding in unadjusted (A) and adjusted (B) analysis. ICA, internal carotid artery; sICH, symptomatic intracranial hemorrhage; aICH, asymptomatic intracranial hemorrhage; HT, hemorrhagic transformation; PH, parenchymal hematoma; SAH, subarachnoid hemorrhage. [*]The acute intracranial vessel occlusion patients with (a) or without (b) concomitant ipsilateral ICA-occlusion or high-grade stenosis were investigated respectively in the study by Bücke et al. 2018. [#]The outcomes of severe extracranial hemorrhage (c) and sICH (d) were investigated respectively in the study of Chalos et al. 2019; The outcomes of HT (e) and SAH (f) were investigated respectively in the study of Dávalos et al. 2012. The outcomes of PH (g) and SAH (f) were investigated respectively in the study of Rebello et al. 2016. The outcome of sICH (d) and aICH (h) were investigated respectively in the studies of Wang et al. 2017, Bellwald et al. 2017, Yang et al.2020 and Hassan et al.2020.

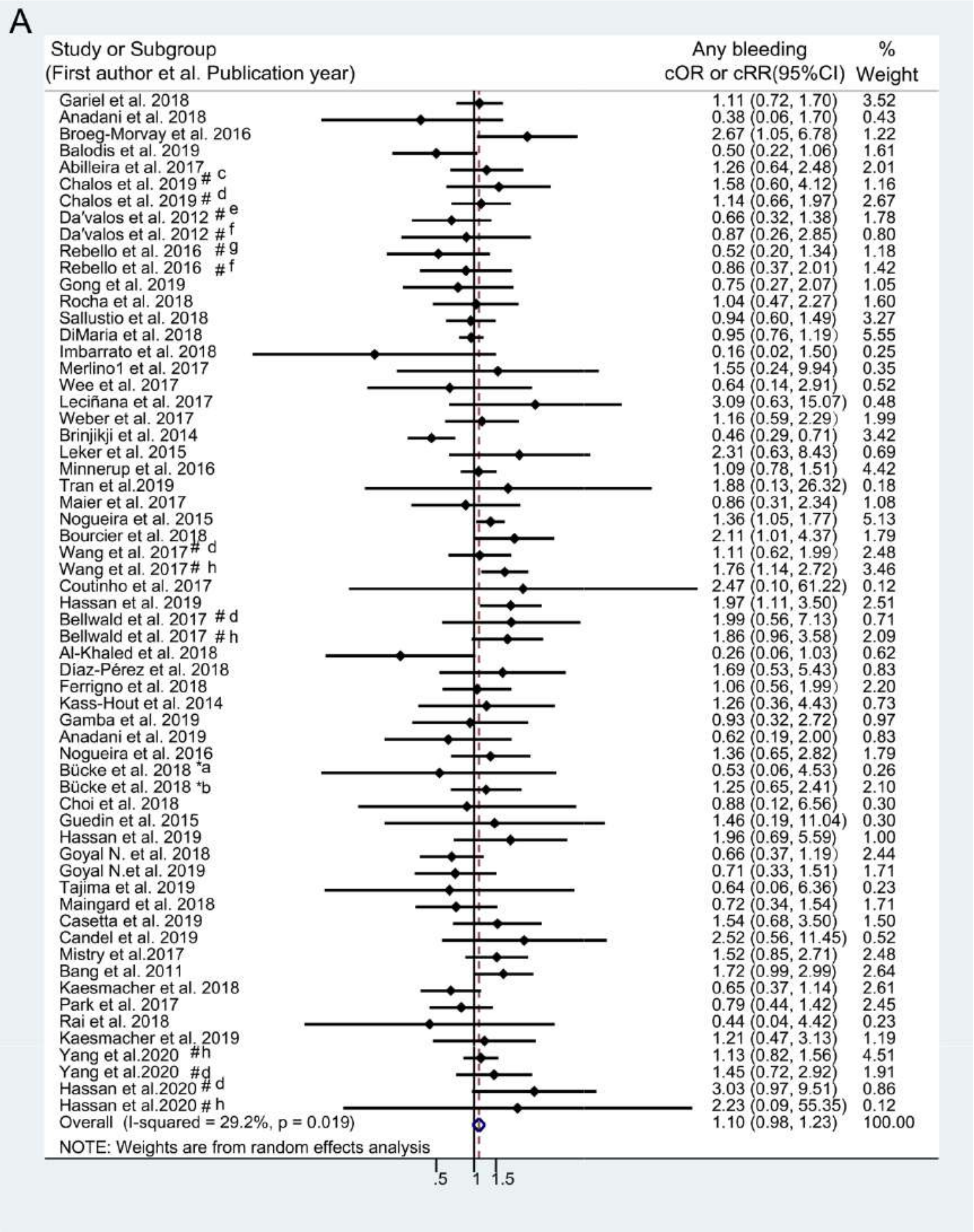
A
Study or Subgroup
(First author et al. Publication year)
Any bleeding
cOR or cRR(95%CI)
% Weight
Gariel et al. 2018 1.11 (0.72, 1.70) 3.52
Anadani et al. 2018 0.38 (0.06, 1.70) 0.43
Broeg-Morvay et al. 2016 2.67 (1.05, 6.78) 1.22
Balodis et al. 2019 0.50 (0.22, 1.06) 1.61
Abilleira et al. 2017 1.26 (0.64, 2.48) 2.01
Chalos et al. 2019 # c 1.58 (0.60, 4.12) 1.16
Chalos et al. 2019 # d 1.14 (0.66, 1.97) 2.67
Da'valos et al. 2012 # e 0.66 (0.32, 1.38) 1.78
Da'valos et al. 2012 # f 0.87 (0.26, 2.85) 0.80
Rebello et al. 2016 # g 0.52 (0.20, 1.34) 1.18
Rebello et al. 2016 # f 0.86 (0.37, 2.01) 1.42
Gong et al. 2019 0.75 (0.27, 2.07) 1.05
Rocha et al. 2018 1.04 (0.47, 2.27) 1.60
Sallustio et al. 2018 0.94 (0.60, 1.49) 3.27
DiMaria et al. 2018 0.95 (0.76, 1.19) 5.55
Imbarrato et al. 2018 0.16 (0.02, 1.50) 0.25
Merlino1 et al. 2017 1.55 (0.24, 9.94) 0.35
Wee et al. 2017 0.64 (0.14, 2.91) 0.52
Leciñana et al. 2017 3.09 (0.63, 15.07) 0.48
Weber et al. 2017 1.16 (0.59, 2.29) 1.99
Brinjikji et al. 2014 0.46 (0.29, 0.71) 3.42
Leker et al. 2015 2.31 (0.63, 8.43) 0.69
Minnerup et al. 2016 1.09 (0.78, 1.51) 4.42
Tran et al.2019 1.88 (0.13, 26.32) 0.18
Maier et al. 2017 0.86 (0.31, 2.34) 1.08
Nogueira et al. 2015 1.36 (1.05, 1.77) 5.13
Bourcier et al. 2018 2.11 (1.01, 4.37) 1.79
Wang et al. 2017# d 1.11 (0.62, 1.99) 2.48
Wang et al. 2017# h 1.76 (1.14, 2.72) 3.46
Coutinho et al. 2017 2.47 (0.10, 61.22) 0.12
Hassan et al. 2019 1.97 (1.11, 3.50) 2.51
Bellwald et al. 2017 # d 1.99 (0.56, 7.13) 0.71
Bellwald et al. 2017 # h 1.86 (0.96, 3.58) 2.09
Al-Khaled et al. 2018 0.26 (0.06, 1.03) 0.62
Díaz-Pérez et al. 2018 1.69 (0.53, 5.43) 0.83
Ferrigno et al. 2018 1.06 (0.56, 1.99) 2.20
Kass-Hout et al. 2014 1.26 (0.36, 4.43) 0.73
Gamba et al. 2019 0.93 (0.32, 2.72) 0.97
Anadani et al. 2019 0.62 (0.19, 2.00) 0.83
Nogueira et al. 2016 1.36 (0.65, 2.82) 1.79
Bücke et al. 2018 *a 0.53 (0.06, 4.53) 0.26
Bücke et al. 2018 *b 1.25 (0.65, 2.41) 2.10
Choi et al. 2018 0.88 (0.12, 6.56) 0.30
Guedin et al. 2015 1.46 (0.19, 11.04) 0.30
Hassan et al. 2019 1.96 (0.69, 5.59) 1.00
Goyal N. et al. 2018 0.66 (0.37, 1.19) 2.44
Goyal N.et al. 2019 0.71 (0.33, 1.51) 1.71
Tajima et al. 2019 0.64 (0.06, 6.36) 0.23
Maingard et al. 2018 0.72 (0.34, 1.54) 1.71
Casetta et al. 2019 1.54 (0.68, 3.50) 1.50
Candel et al. 2019 2.52 (0.56, 11.45) 0.52
Mistry et al.2017 1.52 (0.85, 2.71) 2.48
Bang et al. 2011 1.72 (0.99, 2.99) 2.64
Kaesmacher et al. 2018 0.65 (0.37, 1.14) 2.61
Park et al. 2017 0.79 (0.44, 1.42) 2.45
Rai et al. 2018 0.44 (0.04, 4.42) 0.23
Kaesmacher et al. 2019 1.21 (0.47, 3.13) 1.19
Yang et al.2020 #h 1.13 (0.82, 1.56) 4.51
Yang et al.2020 #d 1.45 (0.72, 2.92) 1.91
Hassan et al.2020 # d 3.03 (0.97, 9.51) 0.86
Hassan et al.2020 # h 2.23 (0.09, 55.35) 0.12
Overall (I-squared = 29.2%, p = 0.019) 1.10 (0.98, 1.23) 100.00
NOTE: Weights are from random effects analysis
.5
1
1.5

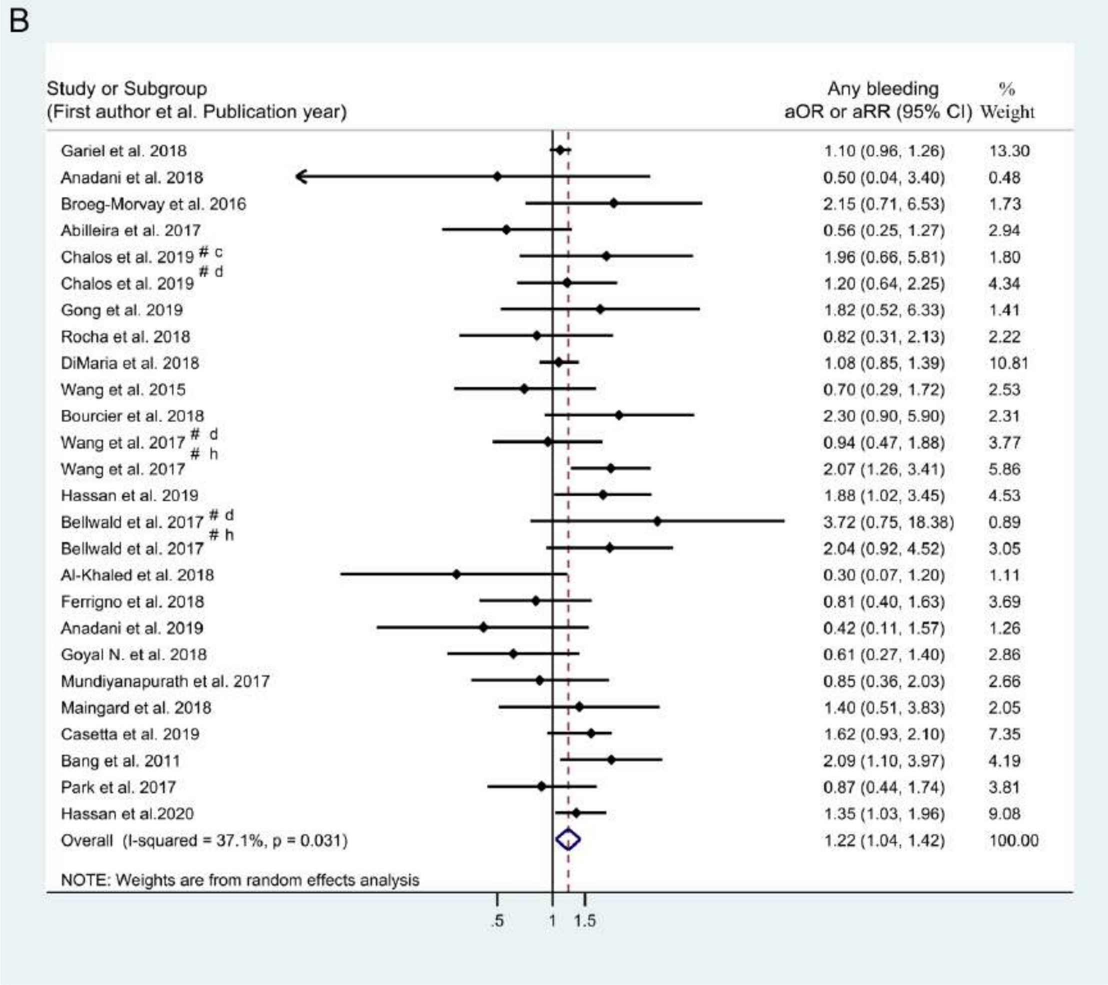
B
Study or Subgroup
(First author et al. Publication year)
Any bleeding
aOR or aRR (95% CI)
%
Weight
Gariel et al. 2018 1.10 (0.96, 1.26) 13.30
Anadani et al. 2018 0.50 (0.04, 3.40) 0.48
Broeg-Morvay et al. 2016 2.15 (0.71, 6.53) 1.73
Abilleira et al. 2017 0.56 (0.25, 1.27) 2.94
Chalos et al. 2019 # c 1.96 (0.66, 5.81) 1.80
Chalos et al. 2019 # d 1.20 (0.64, 2.25) 4.34
Gong et al. 2019 1.82 (0.52, 6.33) 1.41
Rocha et al. 2018 0.82 (0.31, 2.13) 2.22
DiMaria et al. 2018 1.08 (0.85, 1.39) 10.81
Wang et al. 2015 0.70 (0.29, 1.72) 2.53
Bourcier et al. 2018 2.30 (0.90, 5.90) 2.31
Wang et al. 2017 # d 0.94 (0.47, 1.88) 3.77
Wang et al. 2017 # h 2.07 (1.26, 3.41) 5.86
Hassan et al. 2019 1.88 (1.02, 3.45) 4.53
Bellwald et al. 2017 # d 3.72 (0.75, 18.38) 0.89
Bellwald et al. 2017 # h 2.04 (0.92, 4.52) 3.05
Al-Khaled et al. 2018 0.30 (0.07, 1.20) 1.11
Ferrigno et al. 2018 0.81 (0.40, 1.63) 3.69
Anadani et al. 2019 0.42 (0.11, 1.57) 1.26
Goyal N. et al. 2018 0.61 (0.27, 1.40) 2.86
Mundiyanapurath et al. 2017 0.85 (0.36, 2.03) 2.66
Maingard et al. 2018 1.40 (0.51, 3.83) 2.05
Casetta et al. 2019 1.62 (0.93, 2.10) 7.35
Bang et al. 2011 2.09 (1.10, 3.97) 4.19
Park et al. 2017 0.87 (0.44, 1.74) 3.81
Hassan et al.2020 1.35 (1.03, 1.96) 9.08
Overall (I-squared = 37.1%, p = 0.031) 1.22 (1.04, 1.42) 100.00
NOTE: Weights are from random effects analysis
.5
1
1.5

**Supplemental Figure 44.** Forest plots of subgroup analysis assessing any ICH by location of occluded artery in unadjusted (A) and adjusted (B) analysis. ICH, intracranial hemorrhage; sICH, symptomatic intracranial hemorrhage; aICH, asymptomatic intracranial hemorrhage; ICA, internal carotid artery. [*]The acute intracranial vessel occlusion patients with (a) or without (b) concomitant ipsilateral ICA-occlusion or high-grade stenosis were investigated respectively in the study by Bücke et al. 2018. [#]The outcomes of sICH (c) and aICH (d) were investigated respectively in the studies of Hassan et al. 2019, Balodis et al. 2019, Broeg-Morvay et al. 2016, Bellwald et al. 2017, Wang et al. 2017, Yang et al.2020 and Hassan et al.2020.

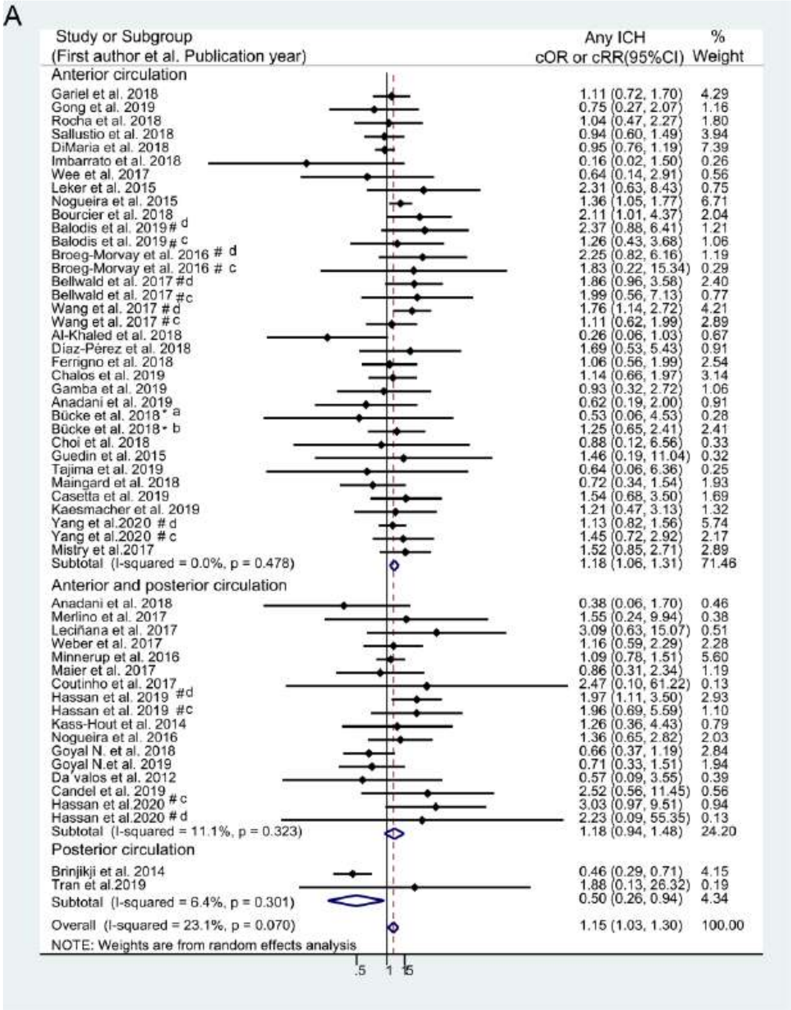

B

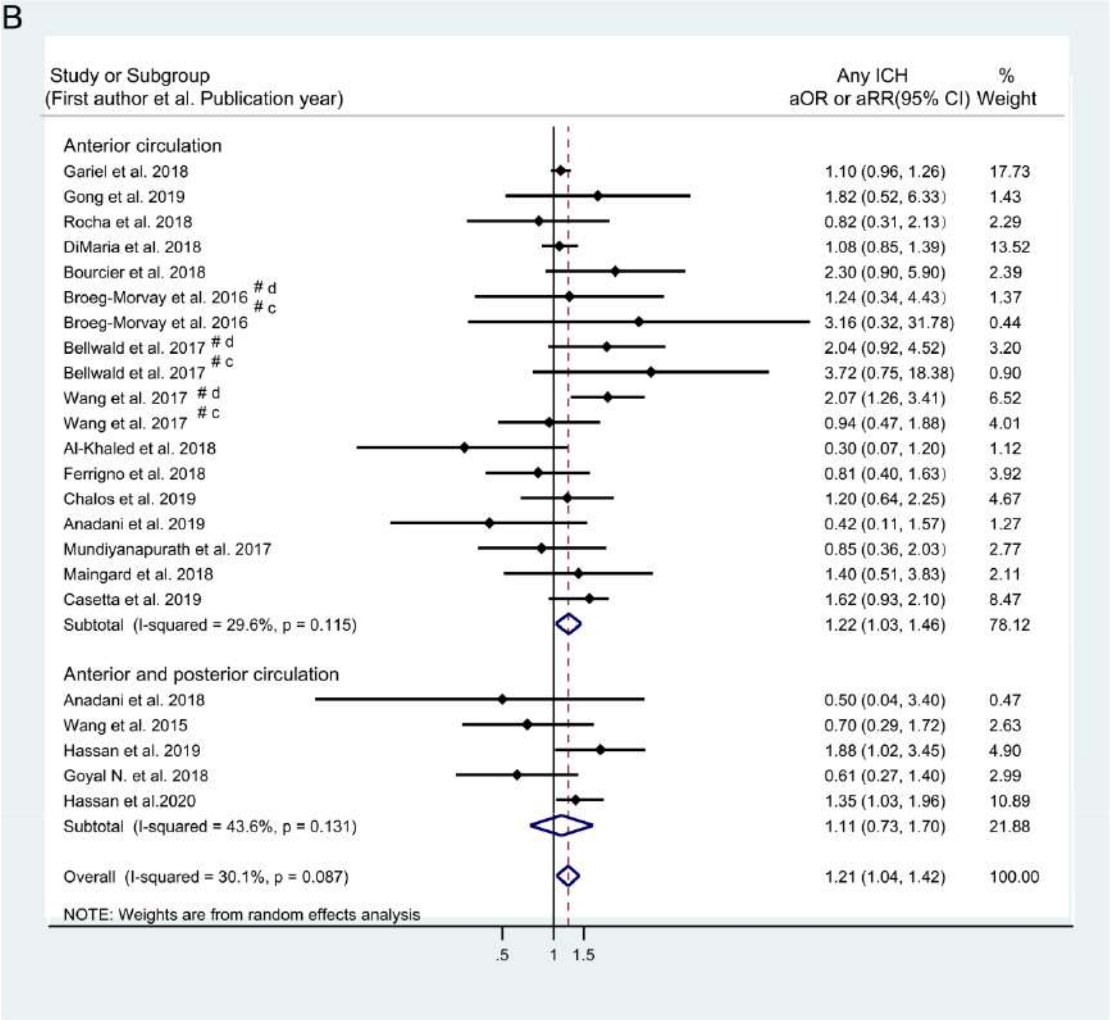

Study or Subgroup
(First author et al. Publication year)
Any ICH
aOR or aRR(95% CI)
% Weight
Anterior circulation
Gariel et al. 2018 1.10 (0.96, 1.26) 17.73
Gong et al. 2019 1.82 (0.52, 6.33) 1.43
Rocha et al. 2018 0.82 (0.31, 2.13) 2.29
DiMaria et al. 2018 1.08 (0.85, 1.39) 13.52
Bourcier et al. 2018 2.30 (0.90, 5.90) 2.39
Broeg-Morvay et al. 2016 # d 1.24 (0.34, 4.43) 1.37
Broeg-Morvay et al. 2016 # c 3.16 (0.32, 31.78) 0.44
Bellwald et al. 2017 # d 2.04 (0.92, 4.52) 3.20
Bellwald et al. 2017 # c 3.72 (0.75, 18.38) 0.90
Wang et al. 2017 # d 2.07 (1.26, 3.41) 6.52
Wang et al. 2017 # c 0.94 (0.47, 1.88) 4.01
Al-Khaled et al. 2018 0.30 (0.07, 1.20) 1.12
Ferrigno et al. 2018 0.81 (0.40, 1.63) 3.92
Chalos et al. 2019 1.20 (0.64, 2.25) 4.67
Anadani et al. 2019 0.42 (0.11, 1.57) 1.27
Mundiyanapurath et al. 2017 0.85 (0.36, 2.03) 2.77
Maingard et al. 2018 1.40 (0.51, 3.83) 2.11
Casetta et al. 2019 1.62 (0.93, 2.10) 8.47
Subtotal (I-squared = 29.6%, p = 0.115) 1.22 (1.03, 1.46) 78.12
Anterior and posterior circulation
Anadani et al. 2018 0.50 (0.04, 3.40) 0.47
Wang et al. 2015 0.70 (0.29, 1.72) 2.63
Hassan et al. 2019 1.88 (1.02, 3.45) 4.90
Goyal N. et al. 2018 0.61 (0.27, 1.40) 2.99
Hassan et al.2020 1.35 (1.03, 1.96) 10.89
Subtotal (I-squared = 43.6%, p = 0.131) 1.11 (0.73, 1.70) 21.88
Overall (I-squared = 30.1%, p = 0.087) 1.21 (1.04, 1.42) 100.00
NOTE: Weights are from random effects analysis
.5
1
1.5

**Supplemental Figure 45.** Forest plots of subgroup analysis assessing any ICH by study type in unadjusted (A) and adjusted (B) analysis. ICH, intracranial hemorrhage; sICH, symptomatic intracranial hemorrhage; aICH, asymptomatic intracranial hemorrhage; ICA, internal carotid artery. *The acute intracranial vessel occlusion patients with (a) or without (b) concomitant ipsilateral ICA-occlusion or high-grade stenosis were investigated respectively in the study by Bücke et al. 2018. #The outcomes of sICH (c) and aICH (d) were investigated respectively in the studies of Hassan et al. 2019, Balodis et al. 2019, Broeg-Morvay et al. 2016, Bellwald et al. 2017, Wang et al. 2017, Yang et al.2020 and Hassan et al.2020

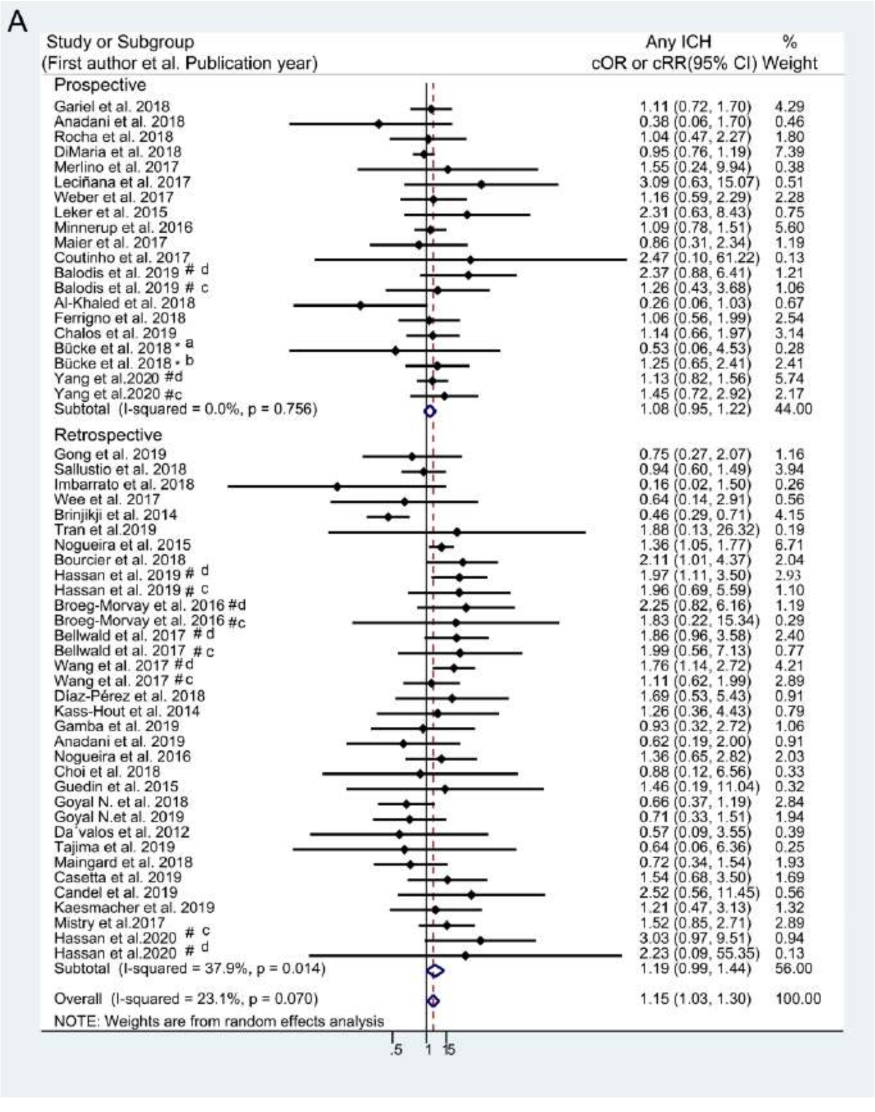

B

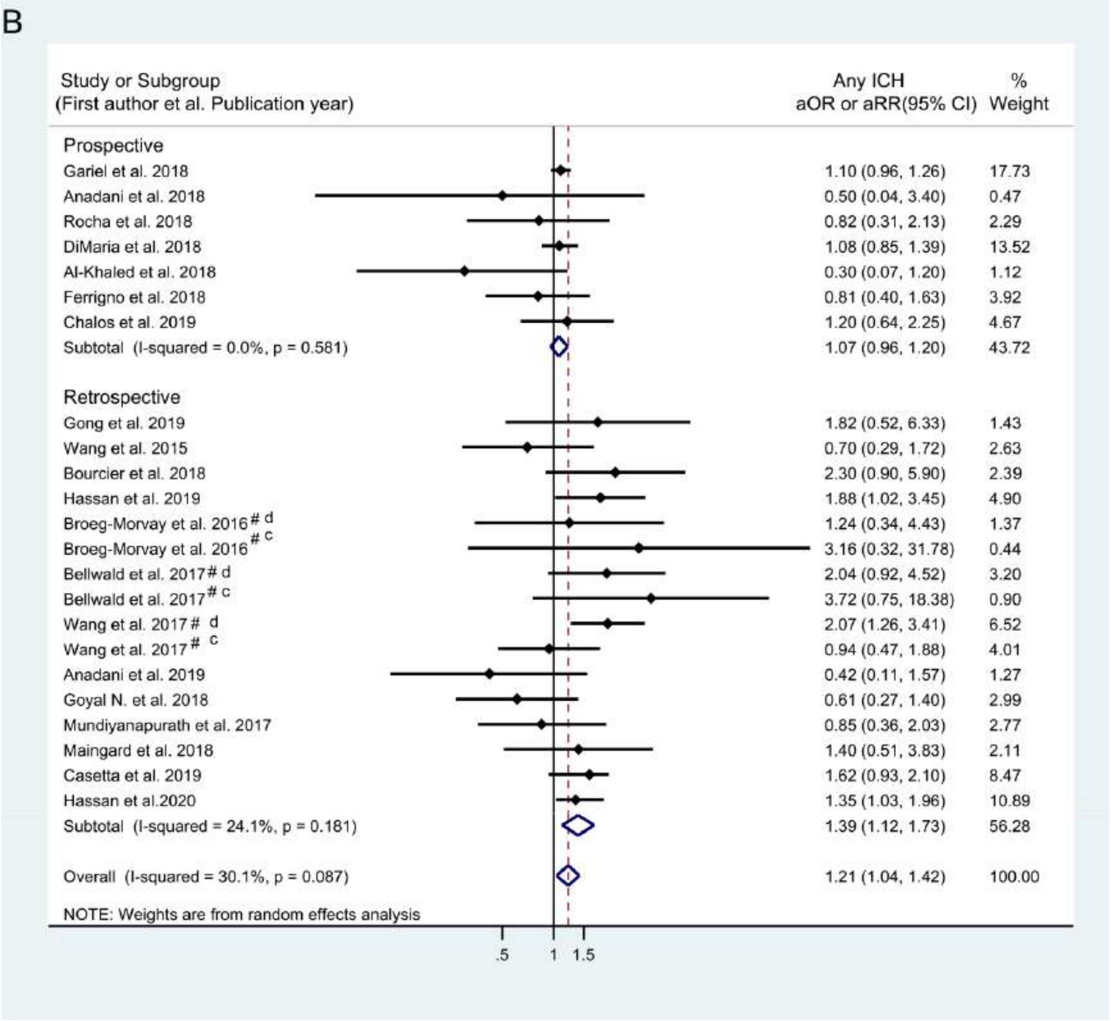
Study or Subgroup
(First author et al. Publication year)
Any ICH
aOR or aRR(95% CI)
%
Weight
Prospective
Gariel et al. 2018 1.10 (0.96, 1.26) 17.73
Anadani et al. 2018 0.50 (0.04, 3.40) 0.47
Rocha et al. 2018 0.82 (0.31, 2.13) 2.29
DiMaria et al. 2018 1.08 (0.85, 1.39) 13.52
Al-Khaled et al. 2018 0.30 (0.07, 1.20) 1.12
Ferrigno et al. 2018 0.81 (0.40, 1.63) 3.92
Chalos et al. 2019 1.20 (0.64, 2.25) 4.67
Subtotal (I-squared = 0.0%, p = 0.581) 1.07 (0.96, 1.20) 43.72
Retrospective
Gong et al. 2019 1.82 (0.52, 6.33) 1.43
Wang et al. 2015 0.70 (0.29, 1.72) 2.63
Bourcier et al. 2018 2.30 (0.90, 5.90) 2.39
Hassan et al. 2019 1.88 (1.02, 3.45) 4.90
Broeg-Morvay et al. 2016# d 1.24 (0.34, 4.43) 1.37
Broeg-Morvay et al. 2016# c 3.16 (0.32, 31.78) 0.44
Bellwald et al. 2017# d 2.04 (0.92, 4.52) 3.20
Bellwald et al. 2017# c 3.72 (0.75, 18.38) 0.90
Wang et al. 2017# d 2.07 (1.26, 3.41) 6.52
Wang et al. 2017# c 0.94 (0.47, 1.88) 4.01
Anadani et al. 2019 0.42 (0.11, 1.57) 1.27
Goyal N. et al. 2018 0.61 (0.27, 1.40) 2.99
Mundiyanapurath et al. 2017 0.85 (0.36, 2.03) 2.77
Maingard et al. 2018 1.40 (0.51, 3.83) 2.11
Casetta et al. 2019 1.62 (0.93, 2.10) 8.47
Hassan et al.2020 1.35 (1.03, 1.96) 10.89
Subtotal (I-squared = 24.1%, p = 0.181) 1.39 (1.12, 1.73) 56.28
Overall (I-squared = 30.1%, p = 0.087) 1.21 (1.04, 1.42) 100.00
NOTE: Weights are from random effects analysis
.5
1
1.5

**Supplemental Figure 46.** Forest plots of subgroup analysis assessing any ICH by ethnicity in unadjusted (A) and adjusted (B) analysis. ICH, intracranial hemorrhage; sICH, symptomatic intracranial hemorrhage; aICH, asymptomatic intracranial hemorrhage; ICA, internal carotid artery. *The acute intracranial vessel occlusion patients with (a) or without (b) concomitant ipsilateral ICA-occlusion or high-grade stenosis were investigated respectively in the study by Bücke et al. 2018. #The outcomes of sICH (c) and aICH (d) were investigated respectively in the studies of Hassan et al. 2019, Balodis et al. 2019, Broeg-Morvay et al. 2016, Bellwald et al. 2017, Wang et al. 2017, Yang et al.2020 and Hassan et al.2020

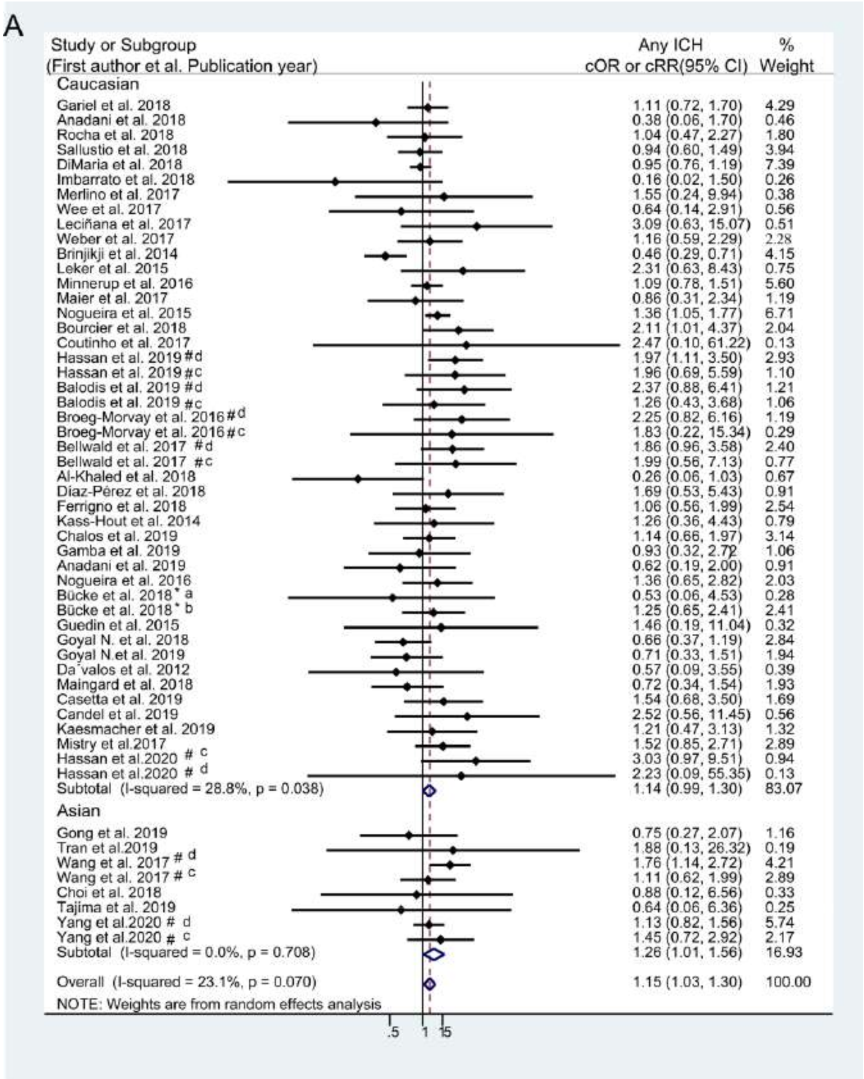

B

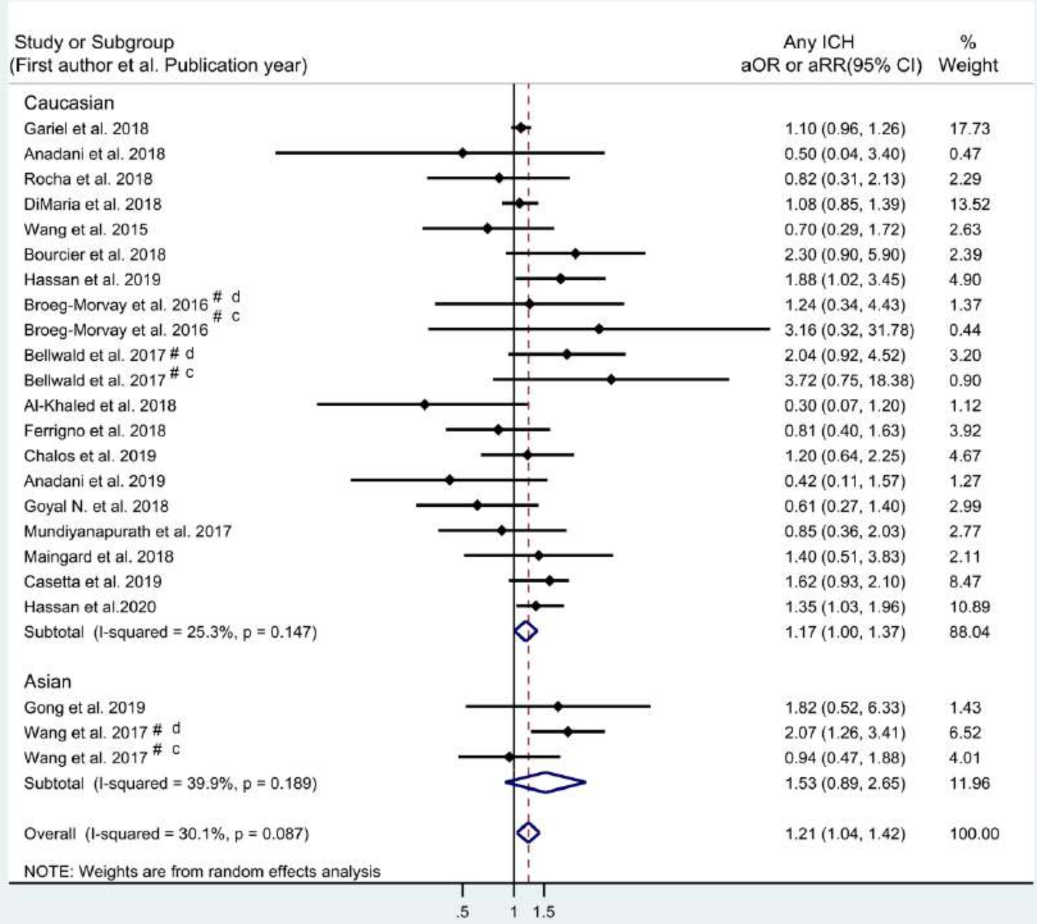

Study or Subgroup
(First author et al. Publication year)
Any ICH
aOR or aRR(95% CI)
%
Weight
Caucasian
Gariel et al. 2018 1.10 (0.96, 1.26) 17.73
Anadani et al. 2018 0.50 (0.04, 3.40) 0.47
Rocha et al. 2018 0.82 (0.31, 2.13) 2.29
DiMaria et al. 2018 1.08 (0.85, 1.39) 13.52
Wang et al. 2015 0.70 (0.29, 1.72) 2.63
Bourcier et al. 2018 2.30 (0.90, 5.90) 2.39
Hassan et al. 2019 1.88 (1.02, 3.45) 4.90
Broeg-Morvay et al. 2016 # d 1.24 (0.34, 4.43) 1.37
Broeg-Morvay et al. 2016 # c 3.16 (0.32, 31.78) 0.44
Bellwald et al. 2017 # d 2.04 (0.92, 4.52) 3.20
Bellwald et al. 2017 # c 3.72 (0.75, 18.38) 0.90
Al-Khaled et al. 2018 0.30 (0.07, 1.20) 1.12
Ferrigno et al. 2018 0.81 (0.40, 1.63) 3.92
Chalos et al. 2019 1.20 (0.64, 2.25) 4.67
Anadani et al. 2019 0.42 (0.11, 1.57) 1.27
Goyal N. et al. 2018 0.61 (0.27, 1.40) 2.99
Mundiyanapurath et al. 2017 0.85 (0.36, 2.03) 2.77
Maingard et al. 2018 1.40 (0.51, 3.83) 2.11
Casetta et al. 2019 1.62 (0.93, 2.10) 8.47
Hassan et al.2020 1.35 (1.03, 1.96) 10.89
Subtotal (I-squared = 25.3%, p = 0.147) 1.17 (1.00, 1.37) 88.04
Asian
Gong et al. 2019 1.82 (0.52, 6.33) 1.43
Wang et al. 2017 # d 2.07 (1.26, 3.41) 6.52
Wang et al. 2017 # c 0.94 (0.47, 1.88) 4.01
Subtotal (I-squared = 39.9%, p = 0.189) 1.53 (0.89, 2.65) 11.96
Overall (I-squared = 30.1%, p = 0.087) 1.21 (1.04, 1.42) 100.00
NOTE: Weights are from random effects analysis
.5
1
1.5

**Supplemental Figure 47.** Forest plots of subgroup analysis assessing sICH by location of occluded artery in unadjusted (A) and adjusted (B) analysis. sICH, symptomatic intracranial hemorrhage; ICA, internal carotid artery. [*]The acute intracranial vessel occlusion patients with (a) or without (b) concomitant ipsilateral ICA-occlusion or high-grade stenosis were investigated respectively in the study by Bücke et al. 2018.

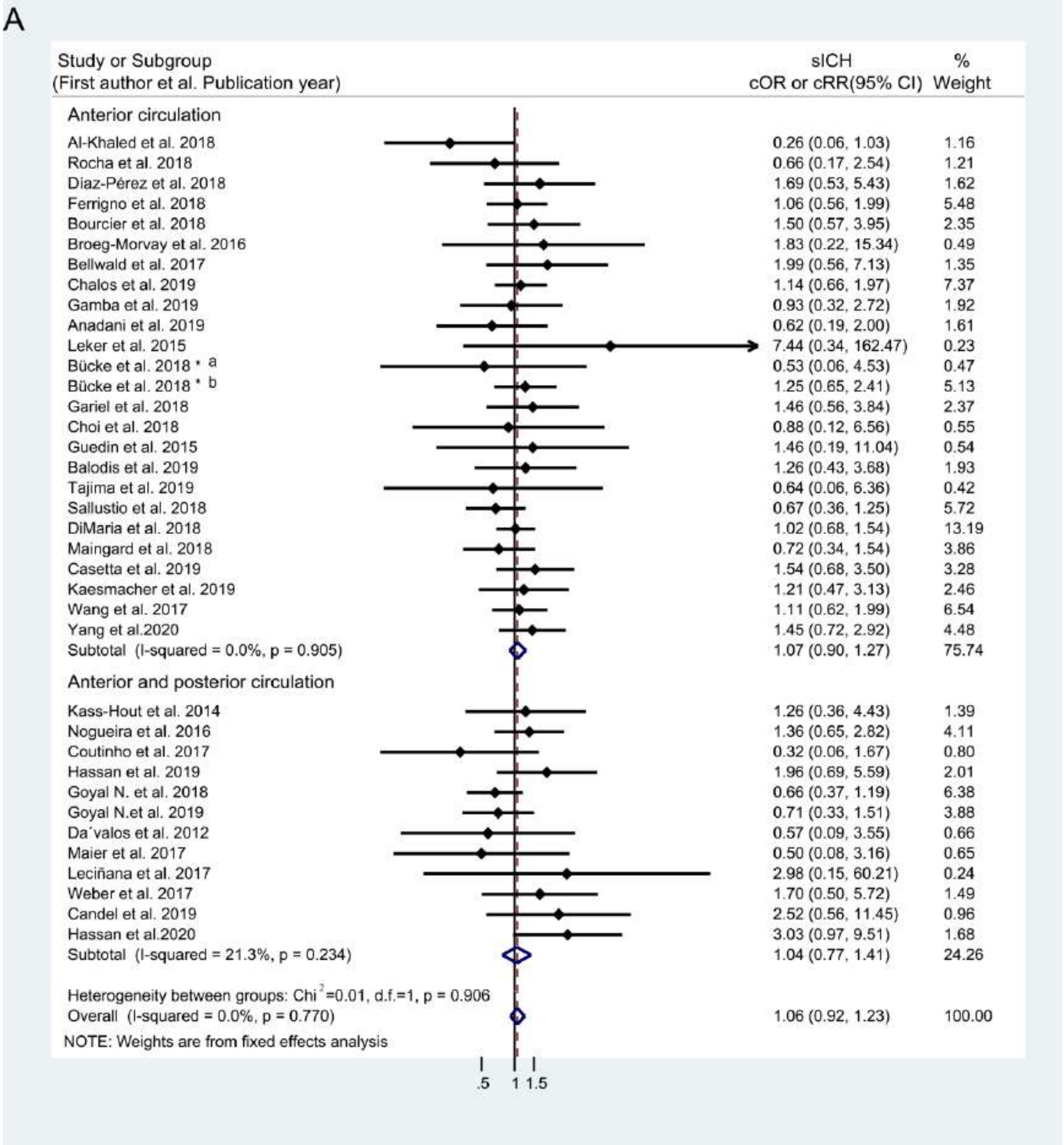

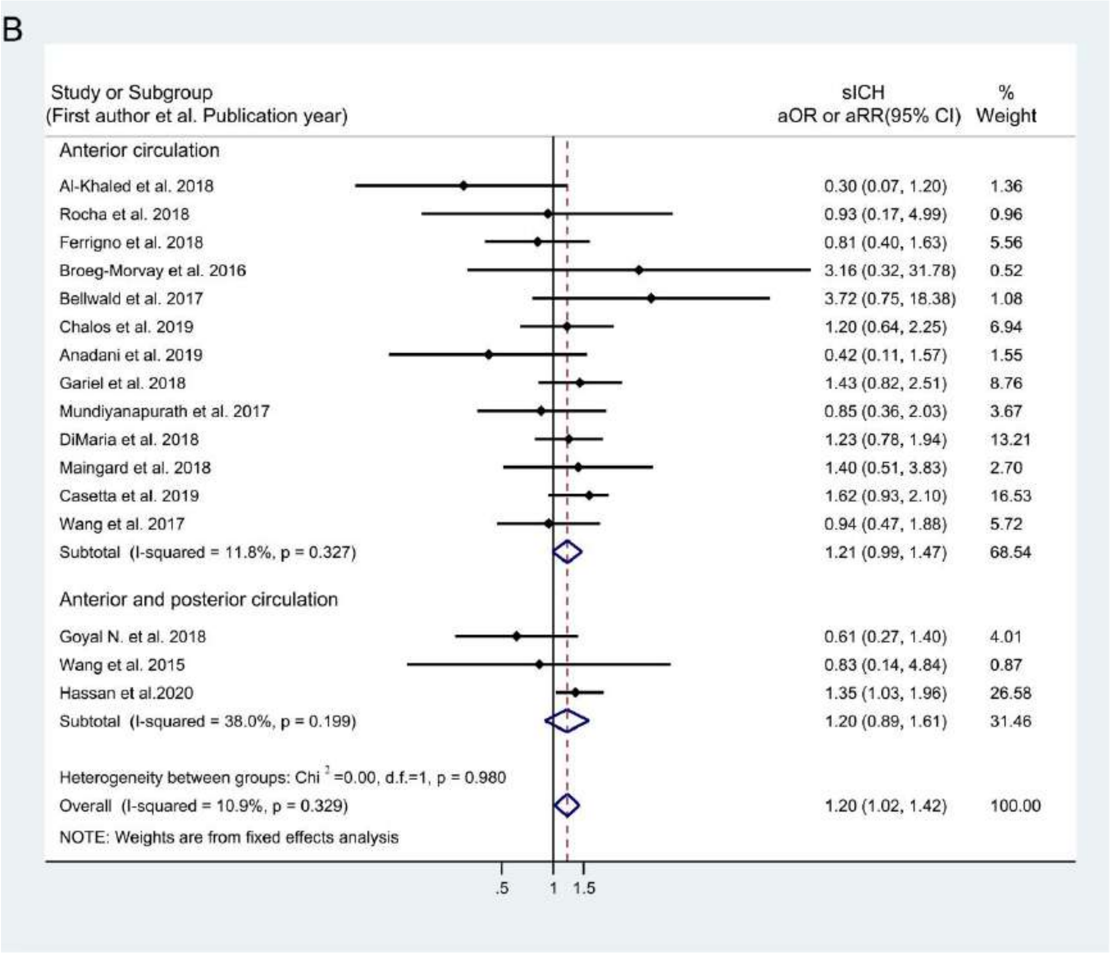
B
Study or Subgroup
(First author et al. Publication year)
sICH
aOR or aRR(95% CI)
% Weight
Anterior circulation
Al-Khaled et al. 2018 0.30 (0.07, 1.20) 1.36
Rocha et al. 2018 0.93 (0.17, 4.99) 0.96
Ferrigno et al. 2018 0.81 (0.40, 1.63) 5.56
Broeg-Morvay et al. 2016 3.16 (0.32, 31.78) 0.52
Bellwald et al. 2017 3.72 (0.75, 18.38) 1.08
Chalos et al. 2019 1.20 (0.64, 2.25) 6.94
Anadani et al. 2019 0.42 (0.11, 1.57) 1.55
Gariel et al. 2018 1.43 (0.82, 2.51) 8.76
Mundiyanapurath et al. 2017 0.85 (0.36, 2.03) 3.67
DiMaria et al. 2018 1.23 (0.78, 1.94) 13.21
Maingard et al. 2018 1.40 (0.51, 3.83) 2.70
Casetta et al. 2019 1.62 (0.93, 2.10) 16.53
Wang et al. 2017 0.94 (0.47, 1.88) 5.72
Subtotal (I-squared = 11.8%, p = 0.327) 1.21 (0.99, 1.47) 68.54
Anterior and posterior circulation
Goyal N. et al. 2018 0.61 (0.27, 1.40) 4.01
Wang et al. 2015 0.83 (0.14, 4.84) 0.87
Hassan et al.2020 1.35 (1.03, 1.96) 26.58
Subtotal (I-squared = 38.0%, p = 0.199) 1.20 (0.89, 1.61) 31.46
Heterogeneity between groups: Chi 2 =0.00, d.f.=1, p = 0.980
Overall (I-squared = 10.9%, p = 0.329) 1.20 (1.02, 1.42) 100.00
NOTE: Weights are from fixed effects analysis
.5
1
1.5

**Supplemental Figure 48.** Forest plots of subgroup analysis assessing sICH by study type in unadjusted (A) and adjusted (B) analysis. sICH, symptomatic intracranial hemorrhage; ICA, internal carotid artery. *The acute intracranial vessel occlusion patients with (a) or without (b) concomitant ipsilateral ICA-occlusion or high-grade stenosis were investigated respectively in the study by Bücke et al. 2018.

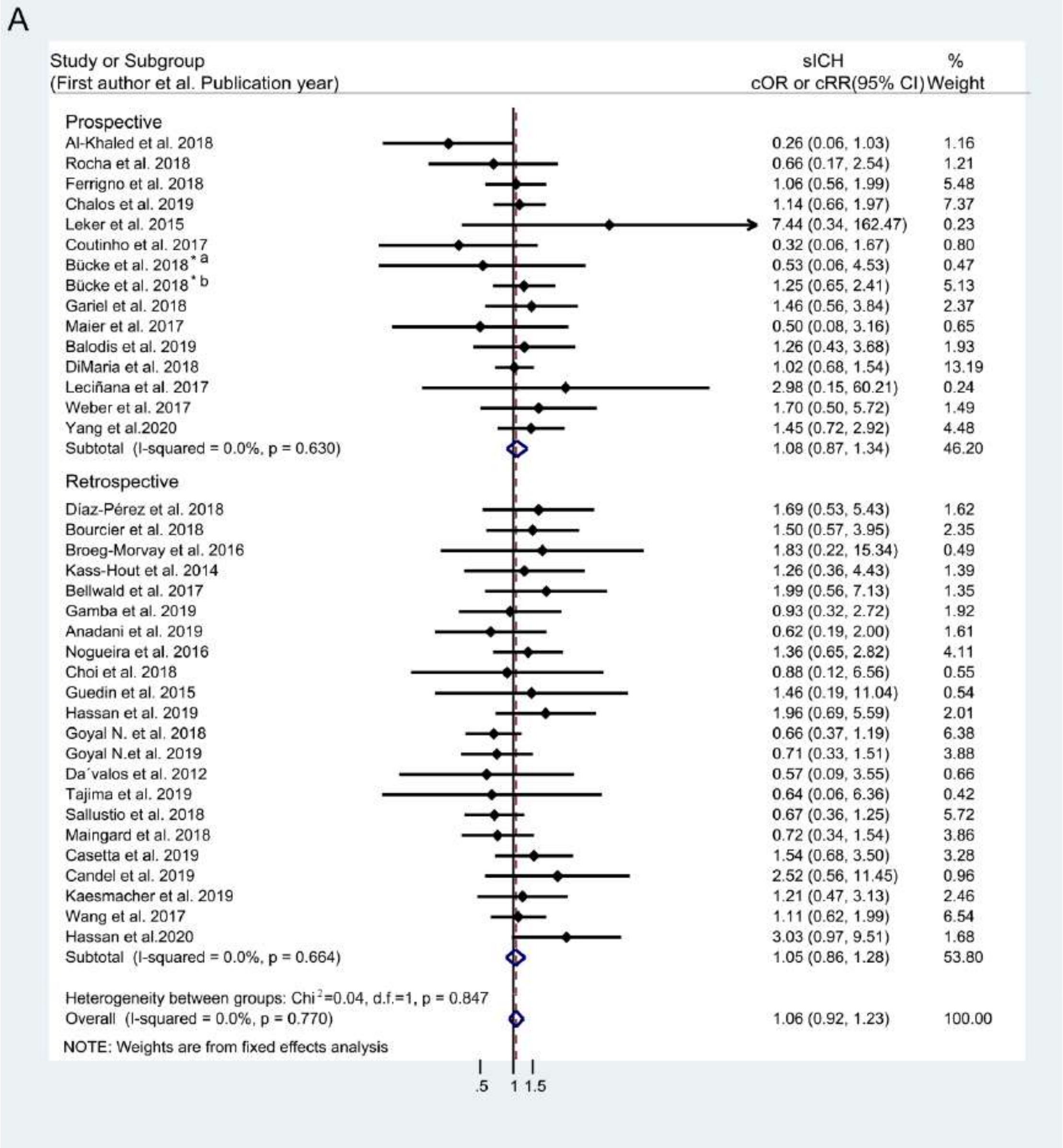

B

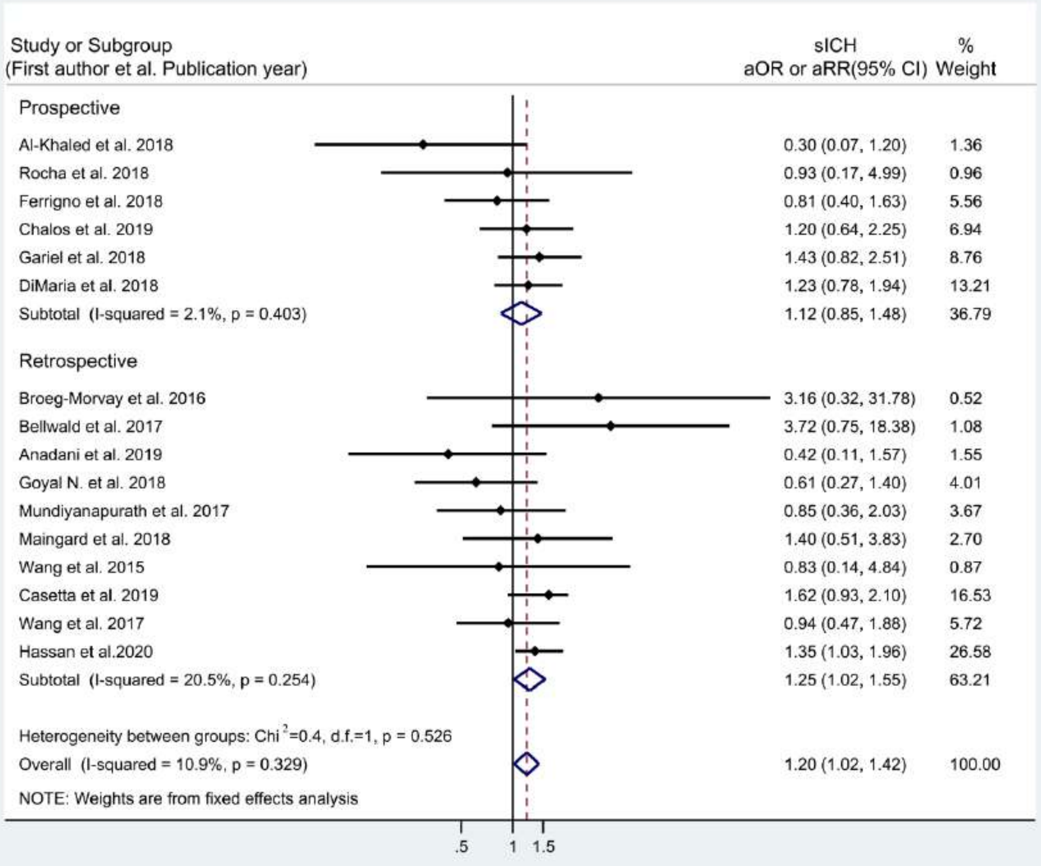

Study or Subgroup
(First author et al. Publication year)
sICH
aOR or aRR(95% CI)
%
Weight
Prospective
Al-Khaled et al. 2018 0.30 (0.07, 1.20) 1.36
Rocha et al. 2018 0.93 (0.17, 4.99) 0.96
Ferrigno et al. 2018 0.81 (0.40, 1.63) 5.56
Chalos et al. 2019 1.20 (0.64, 2.25) 6.94
Gariel et al. 2018 1.43 (0.82, 2.51) 8.76
DiMaria et al. 2018 1.23 (0.78, 1.94) 13.21
Subtotal (I-squared = 2.1%, p = 0.403) 1.12 (0.85, 1.48) 36.79
Retrospective
Broeg-Morvay et al. 2016 3.16 (0.32, 31.78) 0.52
Bellwald et al. 2017 3.72 (0.75, 18.38) 1.08
Anadani et al. 2019 0.42 (0.11, 1.57) 1.55
Goyal N. et al. 2018 0.61 (0.27, 1.40) 4.01
Mundiyanapurath et al. 2017 0.85 (0.36, 2.03) 3.67
Maingard et al. 2018 1.40 (0.51, 3.83) 2.70
Wang et al. 2015 0.83 (0.14, 4.84) 0.87
Casetta et al. 2019 1.62 (0.93, 2.10) 16.53
Wang et al. 2017 0.94 (0.47, 1.88) 5.72
Hassan et al.2020 1.35 (1.03, 1.96) 26.58
Subtotal (I-squared = 20.5%, p = 0.254) 1.25 (1.02, 1.55) 63.21
Heterogeneity between groups: Chi$^2$=0.4, d.f.=1, p = 0.526
Overall (I-squared = 10.9%, p = 0.329) 1.20 (1.02, 1.42) 100.00
NOTE: Weights are from fixed effects analysis
.5
1
1.5

**Supplemental Figure 49.** Forest plots of subgroup analysis assessing sICH by ethnicity in unadjusted (A) and adjusted (B) analysis. sICH, symptomatic intracranial hemorrhage; ICA, internal carotid artery. *The acute intracranial vessel occlusion patients with (a) or without (b) concomitant ipsilateral ICA-occlusion or high-grade stenosis were investigated respectively in the study by Bücke et al. 2018.

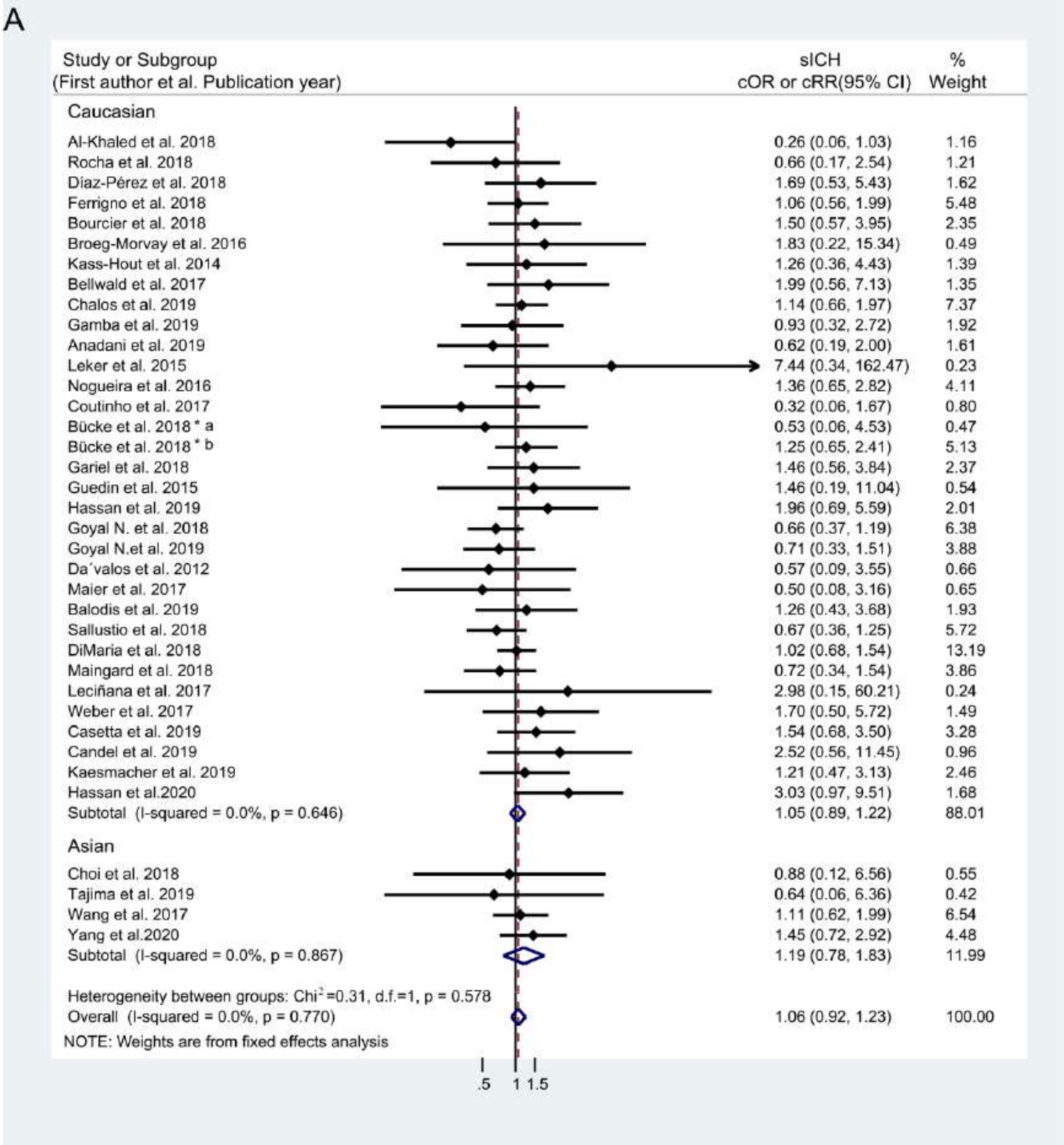

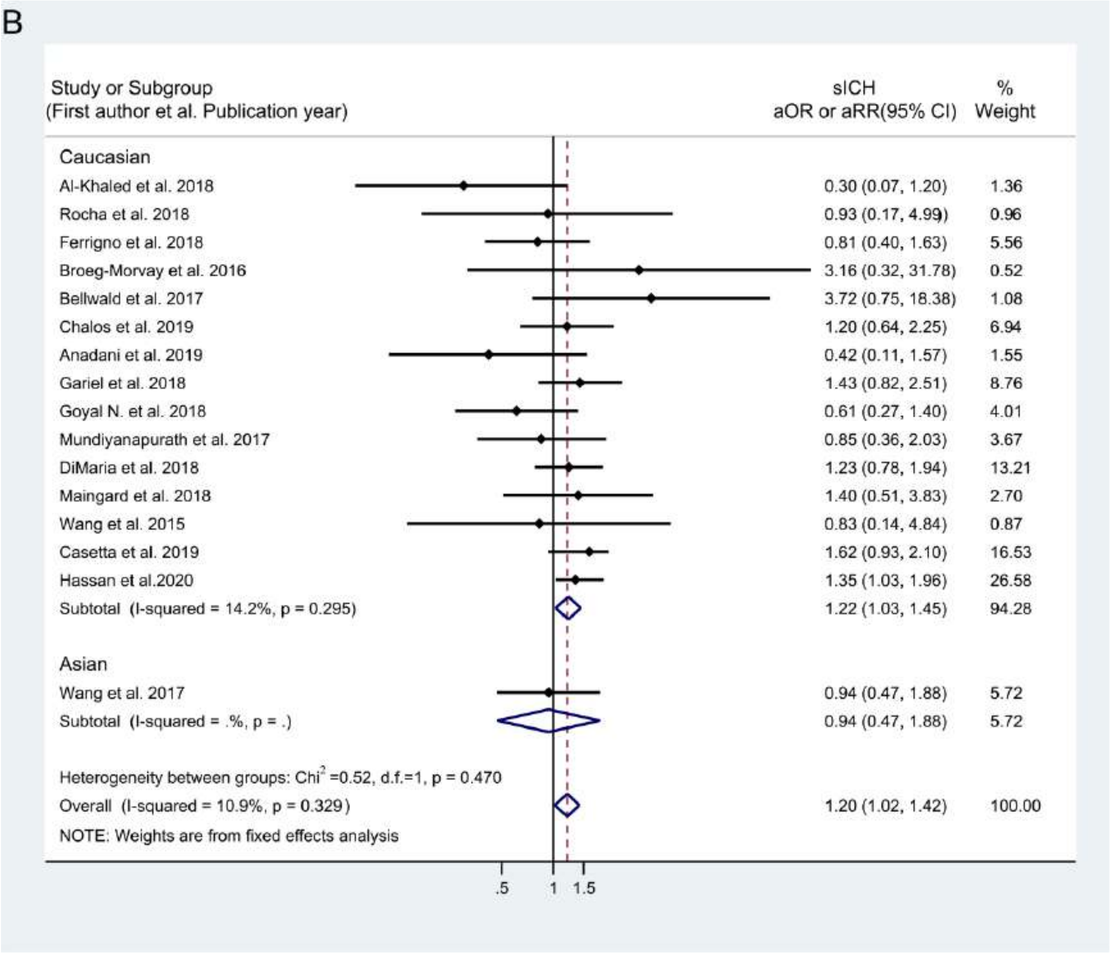
B
Study or Subgroup
(First author et al. Publication year)
sICH
aOR or aRR(95% CI)
%
Weight
Caucasian
Al-Khaled et al. 2018 0.30 (0.07, 1.20) 1.36
Rocha et al. 2018 0.93 (0.17, 4.99) 0.96
Ferrigno et al. 2018 0.81 (0.40, 1.63) 5.56
Broeg-Morvay et al. 2016 3.16 (0.32, 31.78) 0.52
Bellwald et al. 2017 3.72 (0.75, 18.38) 1.08
Chalos et al. 2019 1.20 (0.64, 2.25) 6.94
Anadani et al. 2019 0.42 (0.11, 1.57) 1.55
Gariel et al. 2018 1.43 (0.82, 2.51) 8.76
Goyal N. et al. 2018 0.61 (0.27, 1.40) 4.01
Mundiyanapurath et al. 2017 0.85 (0.36, 2.03) 3.67
DiMaria et al. 2018 1.23 (0.78, 1.94) 13.21
Maingard et al. 2018 1.40 (0.51, 3.83) 2.70
Wang et al. 2015 0.83 (0.14, 4.84) 0.87
Casetta et al. 2019 1.62 (0.93, 2.10) 16.53
Hassan et al.2020 1.35 (1.03, 1.96) 26.58
Subtotal (I-squared = 14.2%, p = 0.295) 1.22 (1.03, 1.45) 94.28
Asian
Wang et al. 2017 0.94 (0.47, 1.88) 5.72
Subtotal (I-squared = .%, p = .) 0.94 (0.47, 1.88) 5.72
Heterogeneity between groups: Chi$^2$ =0.52, d.f.=1, p = 0.470
Overall (I-squared = 10.9%, p = 0.329) 1.20 (1.02, 1.42) 100.00
NOTE: Weights are from fixed effects analysis
.5
1
1.5

**Supplemental Figure 50.** Forest plots of subgroup analysis assessing HT by location of occluded artery in unadjusted (A) and adjusted (B) analysis. HT, hemorrhagic transformation; PH, parenchymal hematoma; HI, hemorrhagic infarction. [#]The outcomes of PH (a) and HI (b) were investigated respectively in the study of Nogueira et al. 2015; The outcomes of HI-1 (c) and HI-2 (d) were investigated respectively in the studies of Coutinho et al. 2017, Sallustio et al. 2018 and Leciñana et al. 2017; The outcomes of PH-1(e) and PH-2 (f) were investigated respectively in the studies of Sallustio et al. 2018 and Leciñana et al. 2017. The outcome of HI-1 (c), HI-2 (d) and PH2 (f) were investigated respectively in the study of Mulder et al. 2016.

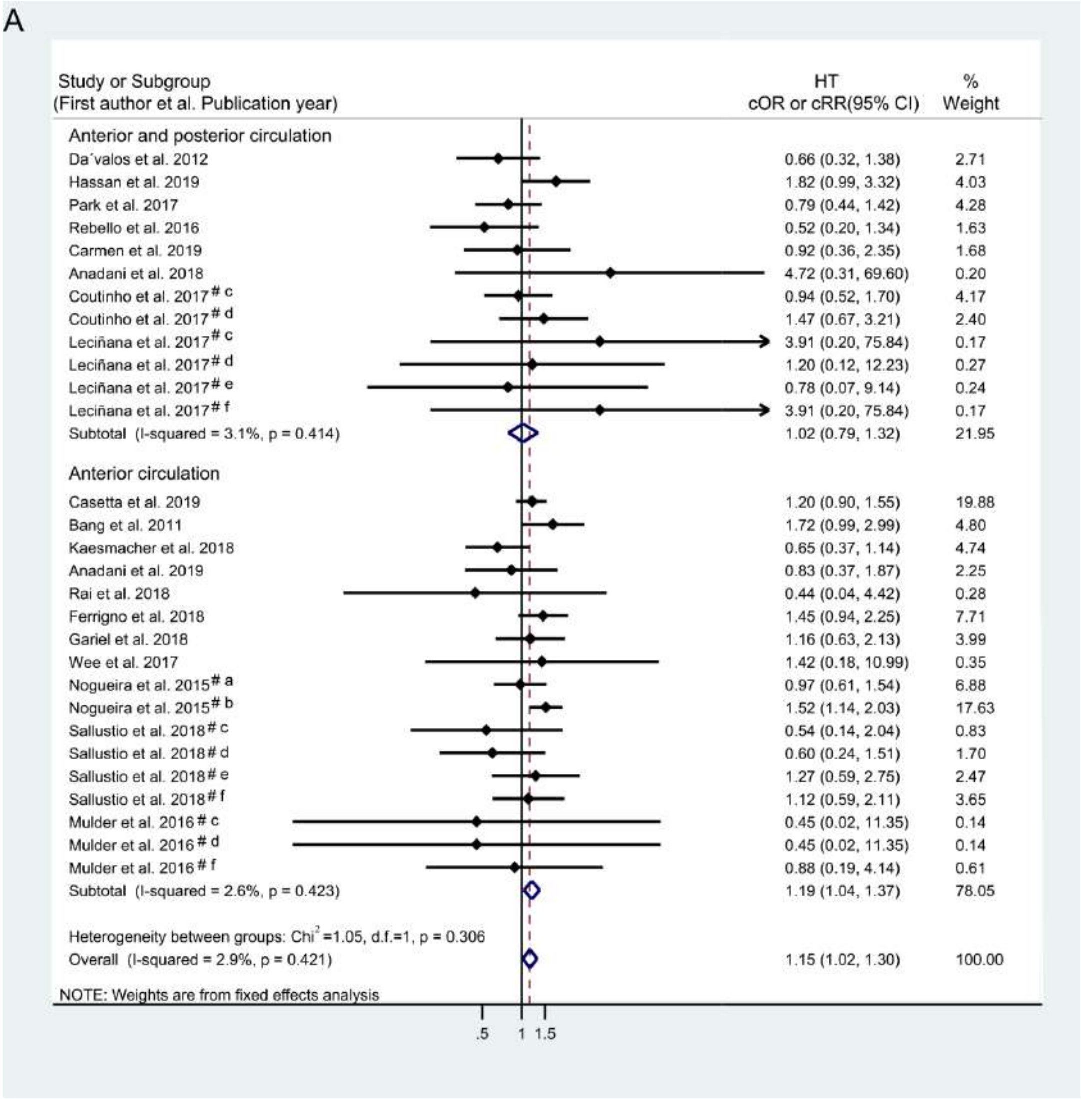

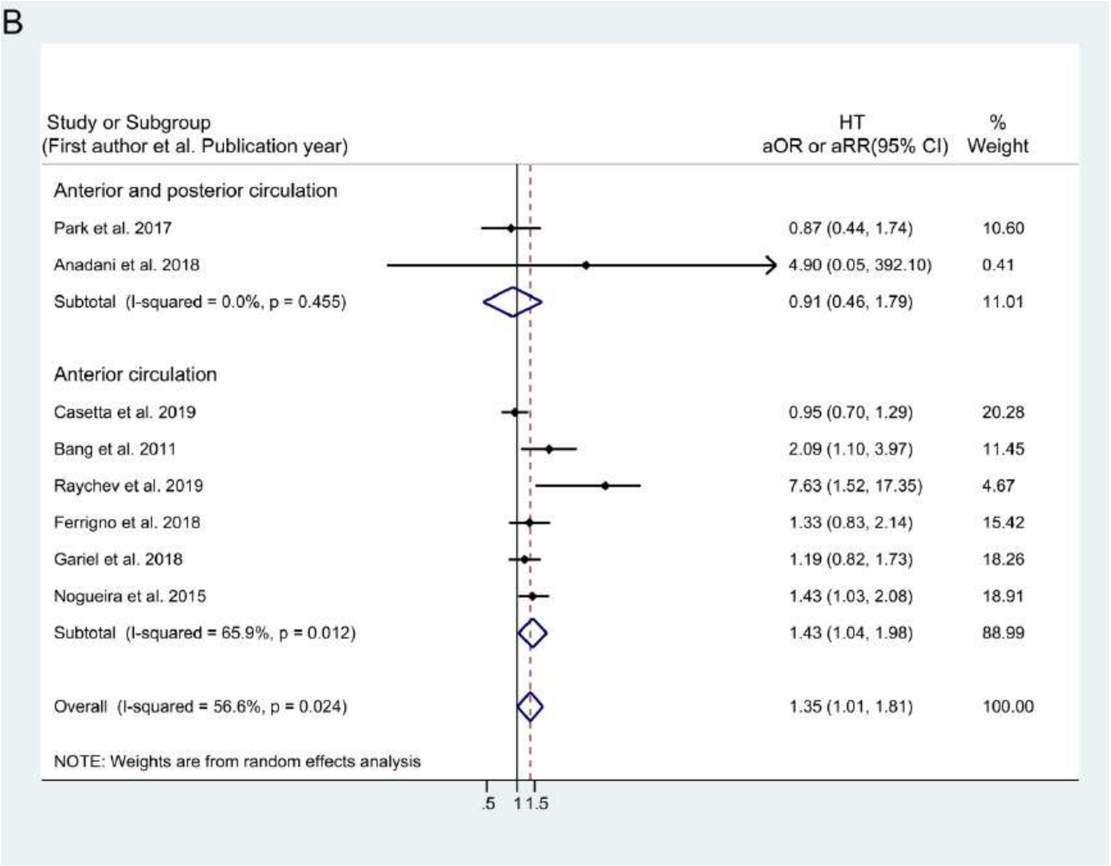
B
Study or Subgroup
(First author et al. Publication year)
HT
aOR or aRR(95% CI)
%
Weight
Anterior and posterior circulation
Park et al. 2017
0.87 (0.44, 1.74)
10.60
Anadani et al. 2018
4.90 (0.05, 392.10)
0.41
Subtotal (I-squared = 0.0%, p = 0.455)
0.91 (0.46, 1.79)
11.01
Anterior circulation
Casetta et al. 2019
0.95 (0.70, 1.29)
20.28
Bang et al. 2011
2.09 (1.10, 3.97)
11.45
Raychev et al. 2019
7.63 (1.52, 17.35)
4.67
Ferrigno et al. 2018
1.33 (0.83, 2.14)
15.42
Gariel et al. 2018
1.19 (0.82, 1.73)
18.26
Nogueira et al. 2015
1.43 (1.03, 2.08)
18.91
Subtotal (I-squared = 65.9%, p = 0.012)
1.43 (1.04, 1.98)
88.99
Overall (I-squared = 56.6%, p = 0.024)
1.35 (1.01, 1.81)
100.00
NOTE: Weights are from random effects analysis
.5
1
1.5

**Supplemental Figure 51.** Forest plots of subgroup analysis assessing HT by study type in unadjusted (A) and adjusted (B) analysis. HT, hemorrhagic transformation; PH, parenchymal hematoma; HI, hemorrhagic infarction. [#]The outcomes of PH (a) and HI (b) were investigated respectively in the study of Nogueira et al. 2015; The outcomes of HI-1 (c) and HI-2 (d) were investigated respectively in the studies of Coutinho et al. 2017, Sallustio et al. 2018 and Leciñana et al. 2017; The outcomes of PH-1(e) and PH-2 (f) were investigated respectively in the studies of Sallustio et al. 2018 and Leciñana et al. 2017. The outcome of HI-1 (c), HI-2 (d) and PH2 (f) were investigated respectively in the study of Mulder et al. 2016.

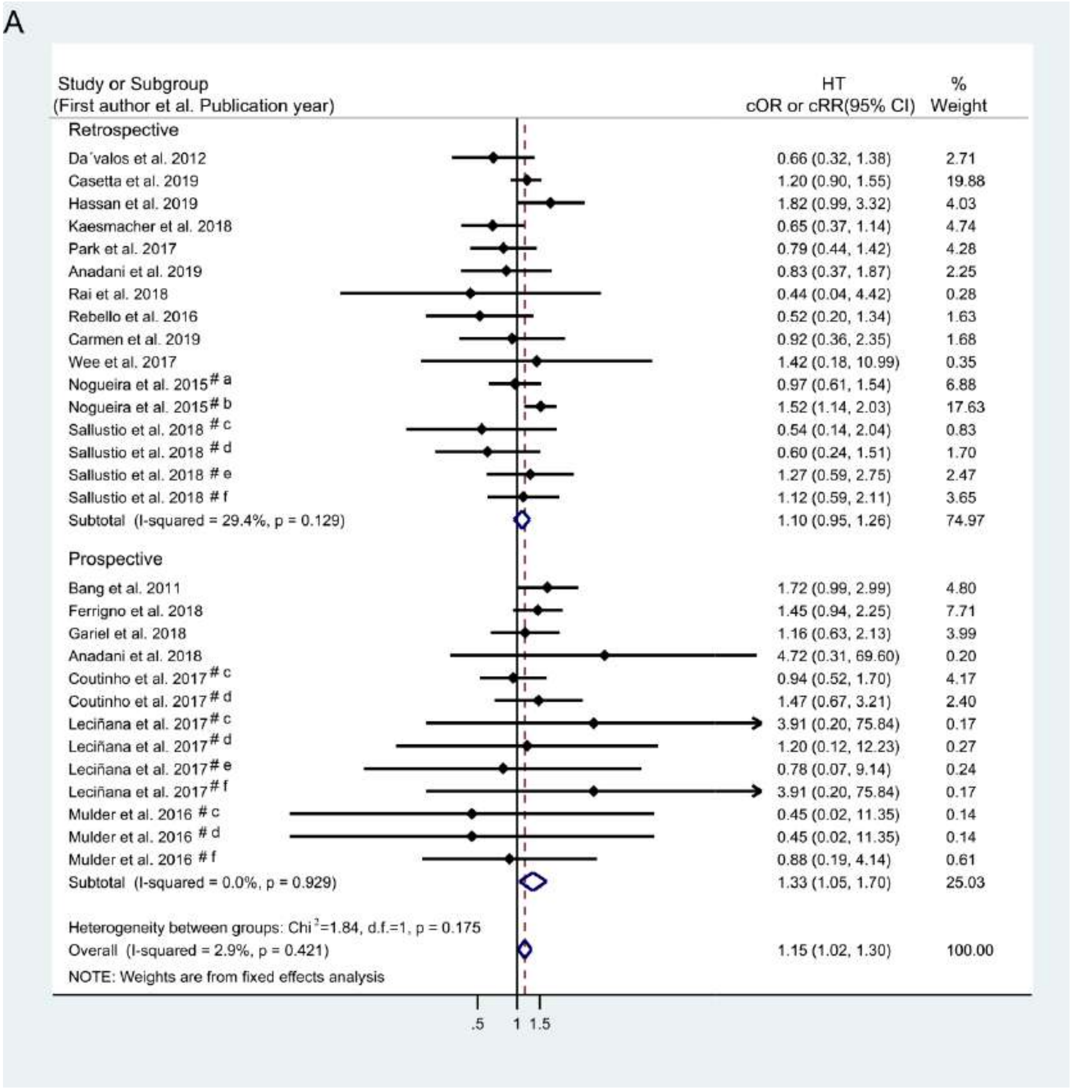

B

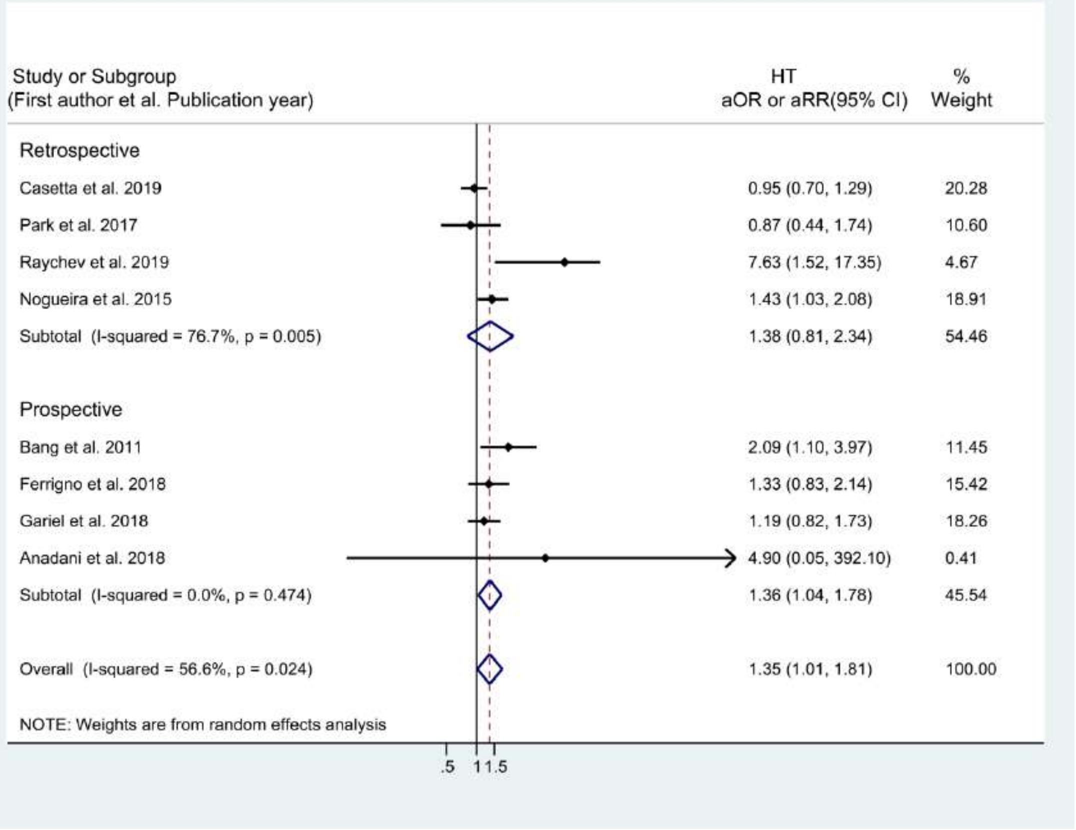

Study or Subgroup
(First author et al. Publication year)
HT
aOR or aRR(95% CI)
%
Weight
Retrospective
Casetta et al. 2019
0.95 (0.70, 1.29)
20.28
Park et al. 2017
0.87 (0.44, 1.74)
10.60
Raychev et al. 2019
7.63 (1.52, 17.35)
4.67
Nogueira et al. 2015
1.43 (1.03, 2.08)
18.91
Subtotal (I-squared = 76.7%, p = 0.005)
1.38 (0.81, 2.34)
54.46
Prospective
Bang et al. 2011
2.09 (1.10, 3.97)
11.45
Ferrigno et al. 2018
1.33 (0.83, 2.14)
15.42
Gariel et al. 2018
1.19 (0.82, 1.73)
18.26
Anadani et al. 2018
4.90 (0.05, 392.10)
0.41
Subtotal (I-squared = 0.0%, p = 0.474)
1.36 (1.04, 1.78)
45.54
Overall (I-squared = 56.6%, p = 0.024)
1.35 (1.01, 1.81)
100.00
NOTE: Weights are from random effects analysis
.5
1
1.5

**Supplemental Figure 52.** Forest plots of subgroup analysis assessing HT by ethnicity in unadjusted (A) and adjusted (B) analysis. HT, hemorrhagic transformation; PH, parenchymal hematoma; HI, hemorrhagic infarction. #The outcomes of PH (a) and HI (b) were investigated respectively in the study of Nogueira et al. 2015; The outcomes of HI-1 (c) and HI-2 (d) were investigated respectively in the studies of Coutinho et al. 2017, Sallustio et al. 2018 and Leciñana et al. 2017; The outcomes of PH-1(e) and PH-2 (f) were investigated respectively in the studies of Sallustio et al. 2018 and Leciñana et al. 2017. The outcome of HI-1 (c), HI-2 (d) and PH2 (f) were investigated respectively in the study of Mulder et al. 2016.

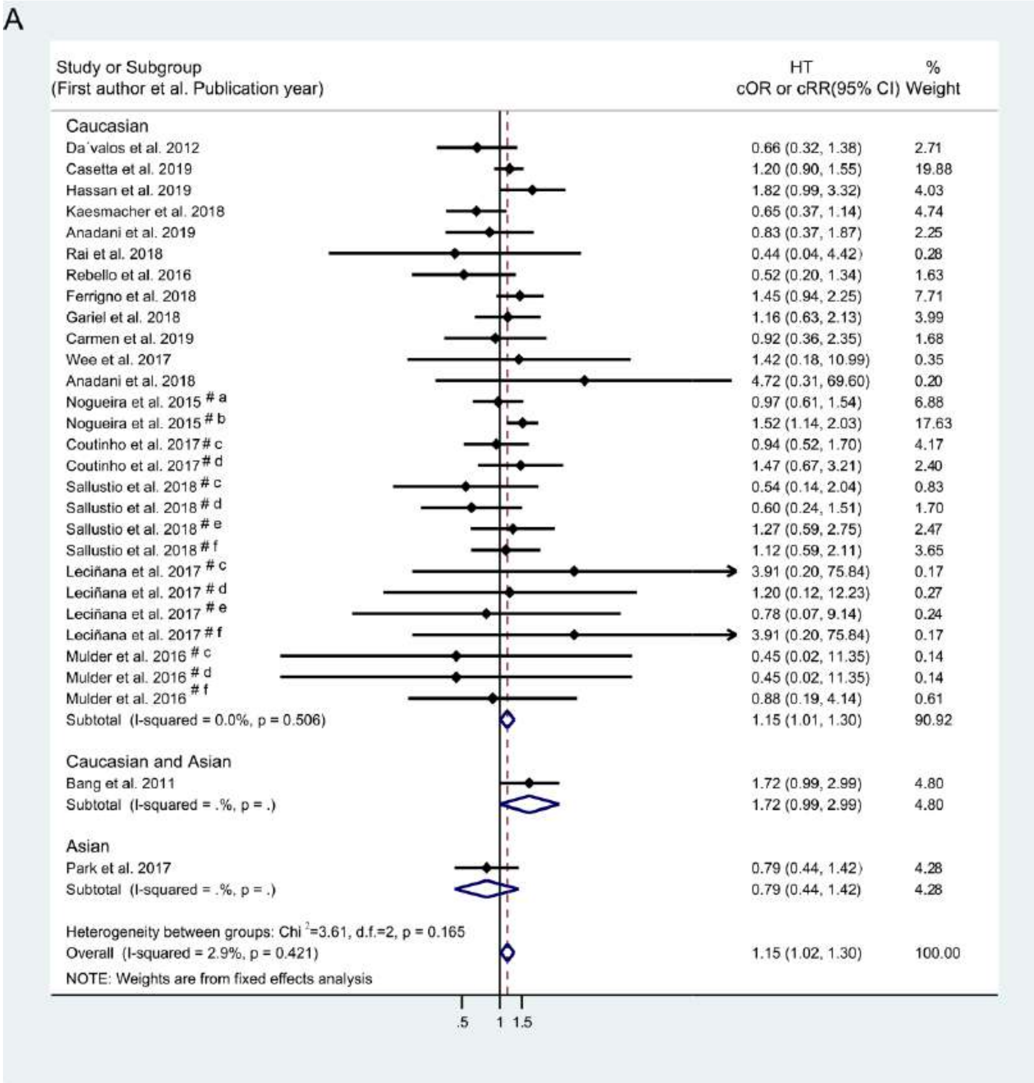

B

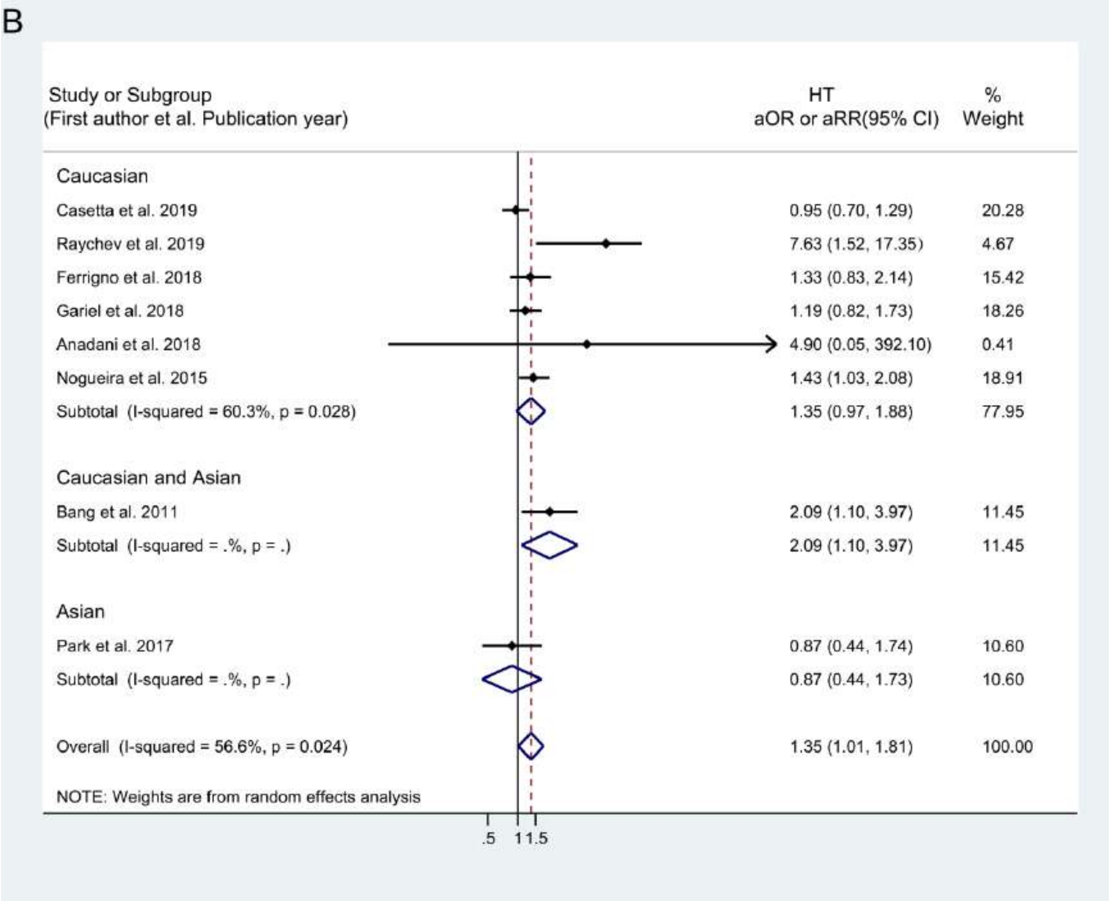
Study or Subgroup
(First author et al. Publication year)
HT
aOR or aRR(95% CI)
%
Weight
Caucasian
Casetta et al. 2019
0.95 (0.70, 1.29)
20.28
Raychev et al. 2019
7.63 (1.52, 17.35)
4.67
Ferrigno et al. 2018
1.33 (0.83, 2.14)
15.42
Gariel et al. 2018
1.19 (0.82, 1.73)
18.26
Anadani et al. 2018
4.90 (0.05, 392.10)
0.41
Nogueira et al. 2015
1.43 (1.03, 2.08)
18.91
Subtotal (I-squared = 60.3%, p = 0.028)
1.35 (0.97, 1.88)
77.95
Caucasian and Asian
Bang et al. 2011
2.09 (1.10, 3.97)
11.45
Subtotal (I-squared = .%, p = .)
2.09 (1.10, 3.97)
11.45
Asian
Park et al. 2017
0.87 (0.44, 1.74)
10.60
Subtotal (I-squared = .%, p = .)
0.87 (0.44, 1.73)
10.60
Overall (I-squared = 56.6%, p = 0.024)
1.35 (1.01, 1.81)
100.00
NOTE: Weights are from random effects analysis
.5
1
1.5

**Supplemental Figure 53.** Forest plots of subgroup analysis assessing aICH by location of occluded artery in unadjusted (A) and adjusted (B) analysis. aICH, asymptomatic intracranial hemorrhage.

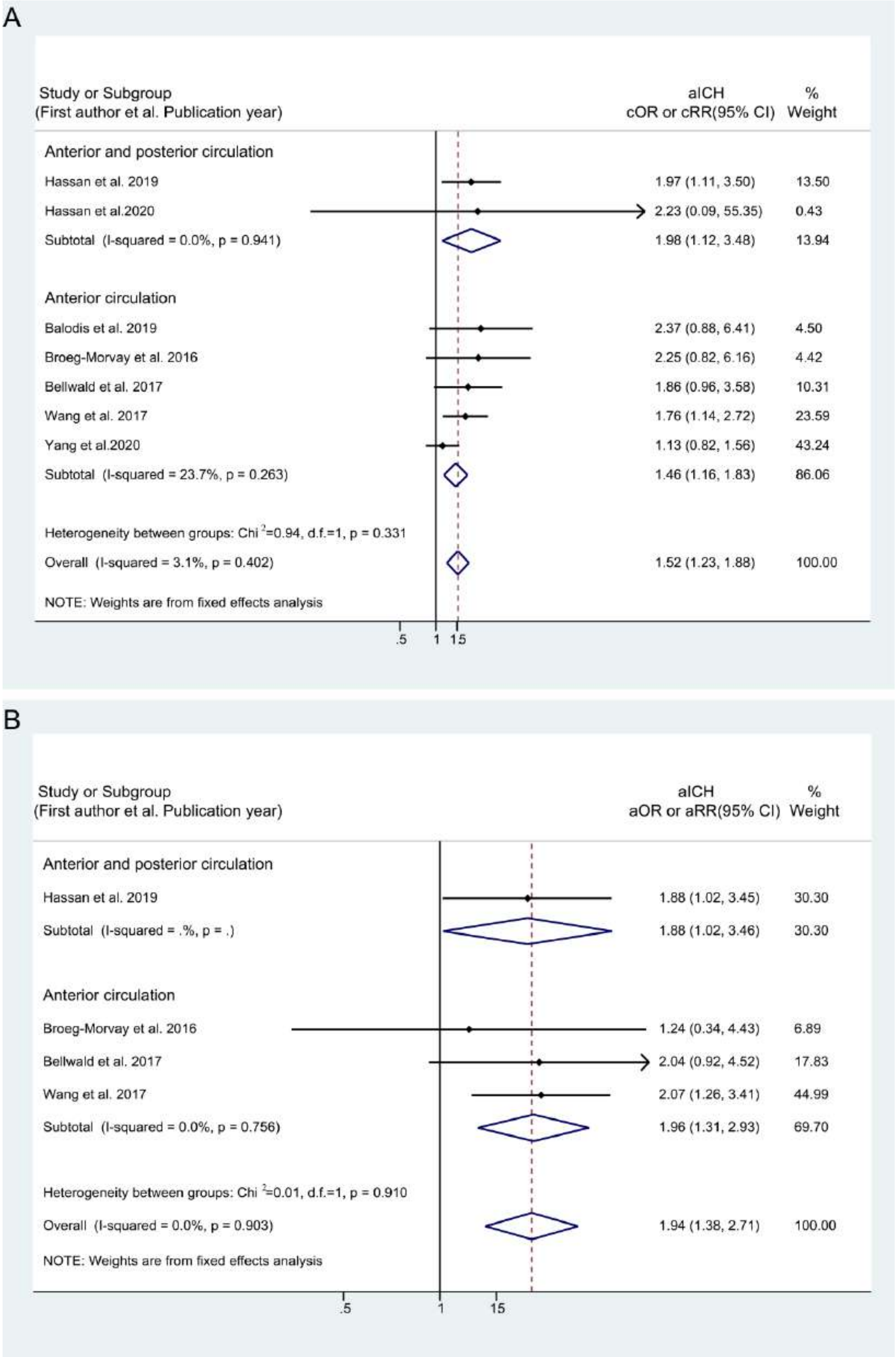

**Supplemental Figure 54.** Forest plots of subgroup analysis assessing aICH by study type in unadjusted analysis. aICH, asymptomatic intracranial hemorrhage.

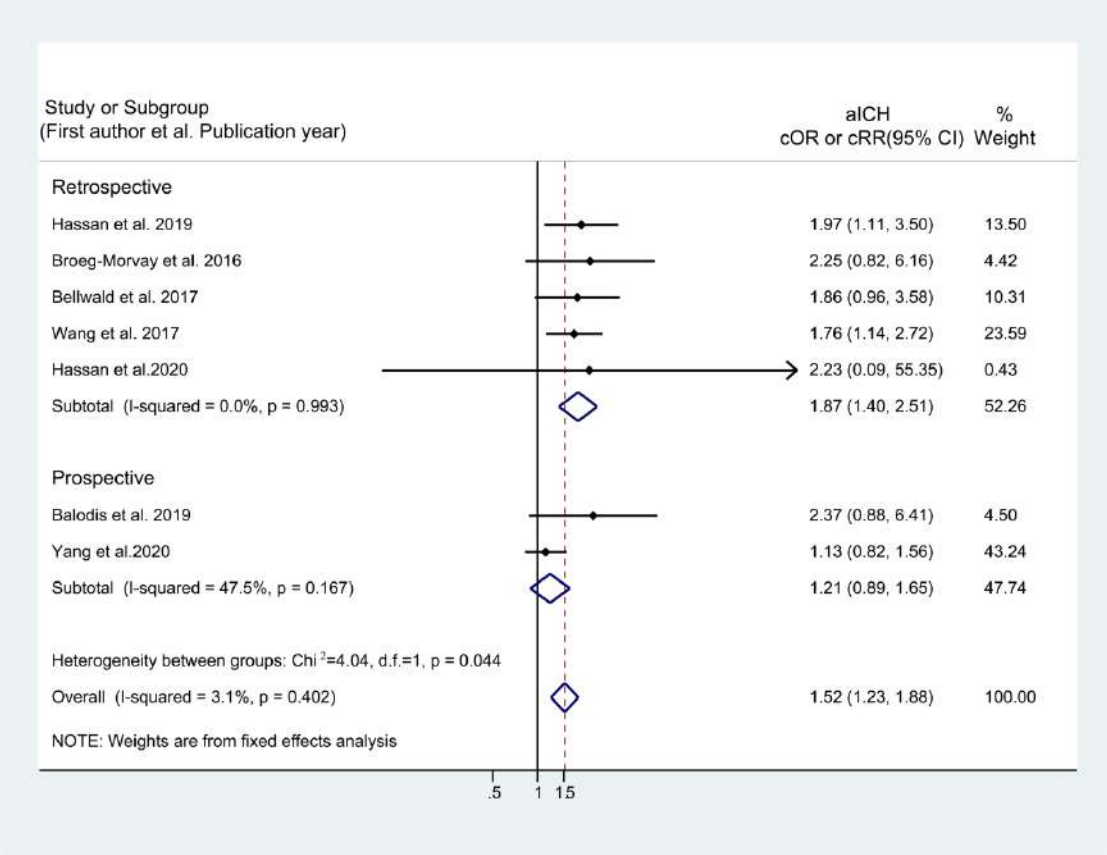

**Supplemental Figure 55.** Forest plots of subgroup analysis assessing aICH by ethnicity in unadjusted (A) and adjusted (B) analysis. aICH, asymptomatic intracranial hemorrhage.

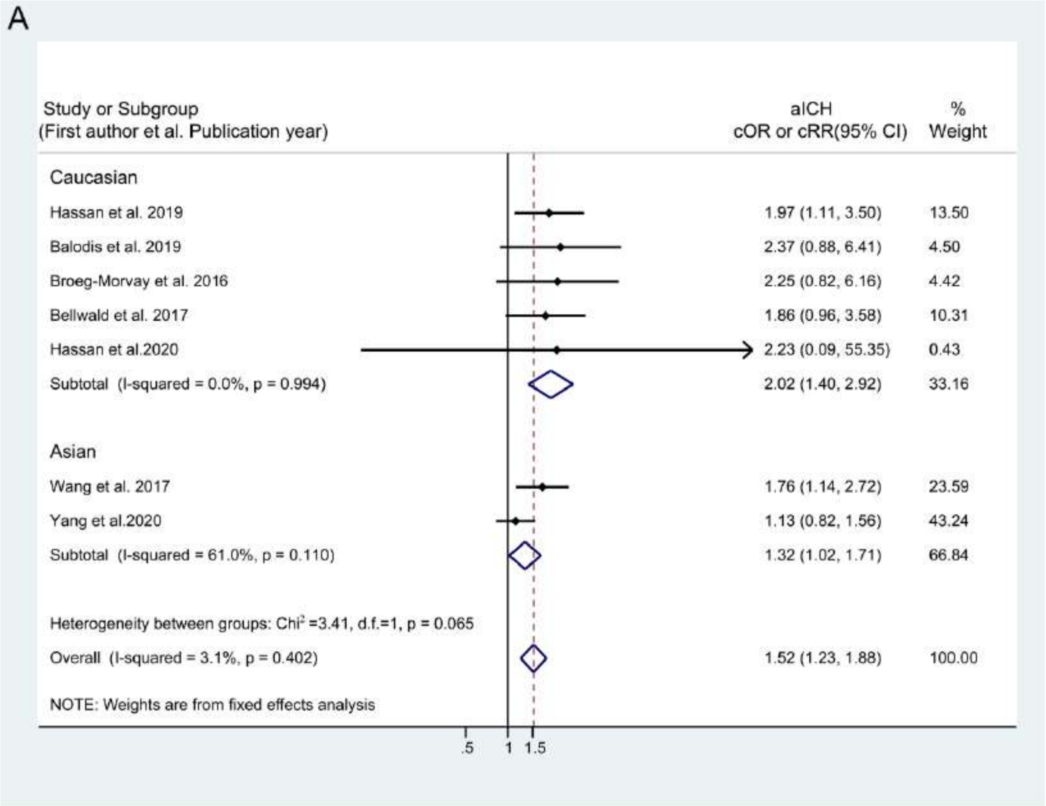


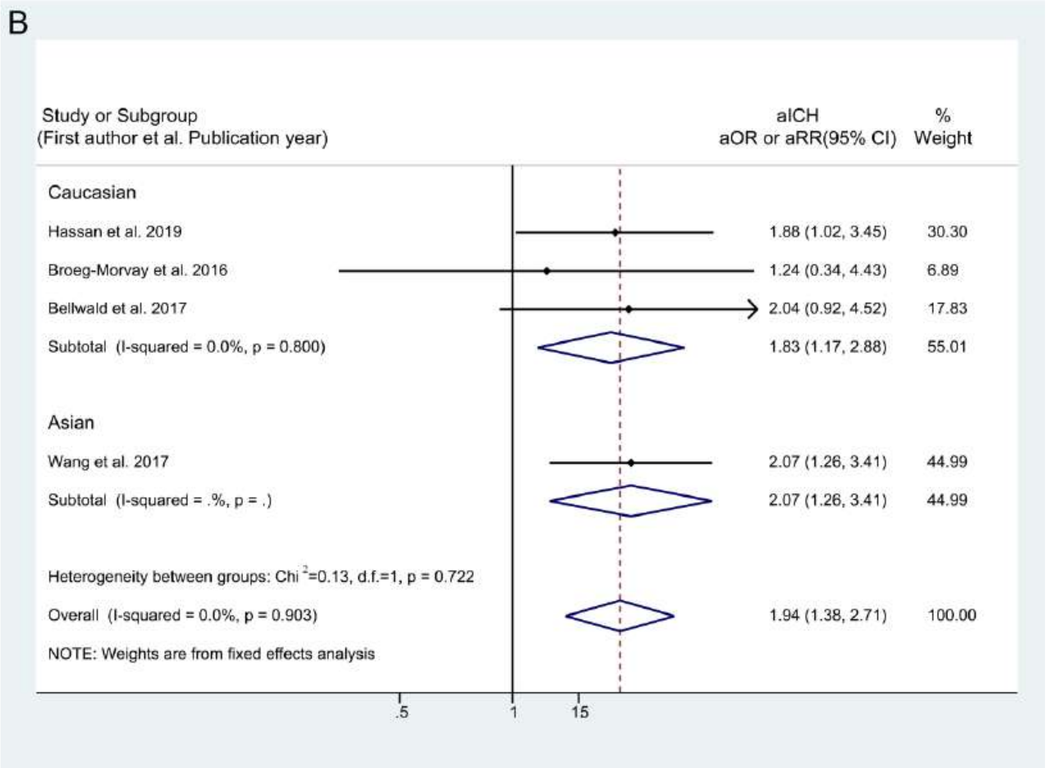

**Supplemental Figure 56.** Forest plots of subgroup analysis assessing any bleeding by location of occluded artery in unadjusted (A) and adjusted (B) analysis. ICA, internal carotid artery; sICH, symptomatic intracranial hemorrhage; aICH, asymptomatic intracranial hemorrhage; HT, hemorrhagic transformation; PH, parenchymal hematoma; SAH, subarachnoid hemorrhage. *The acute intracranial vessel occlusion patients with (a) or without (b) concomitant ipsilateral ICA-occlusion or high-grade stenosis were investigated respectively in the study by Bücke et al. 2018. #The outcomes of severe extracranial hemorrhage (c) and sICH (d) were investigated respectively in the study of Chalos et al. 2019; The outcomes of HT (e) and SAH (f) were investigated respectively in the study of Dávalos et al. 2012. The outcomes of PH (g) and SAH (f) were investigated respectively in the study of Rebello et al. 2016. The outcome of sICH (d) and aICH (h) were investigated respectively in the studies of Wang et al. 2017, Bellwald et al. 2017, Yang et al.2020 and Hassan et al.2020.

A

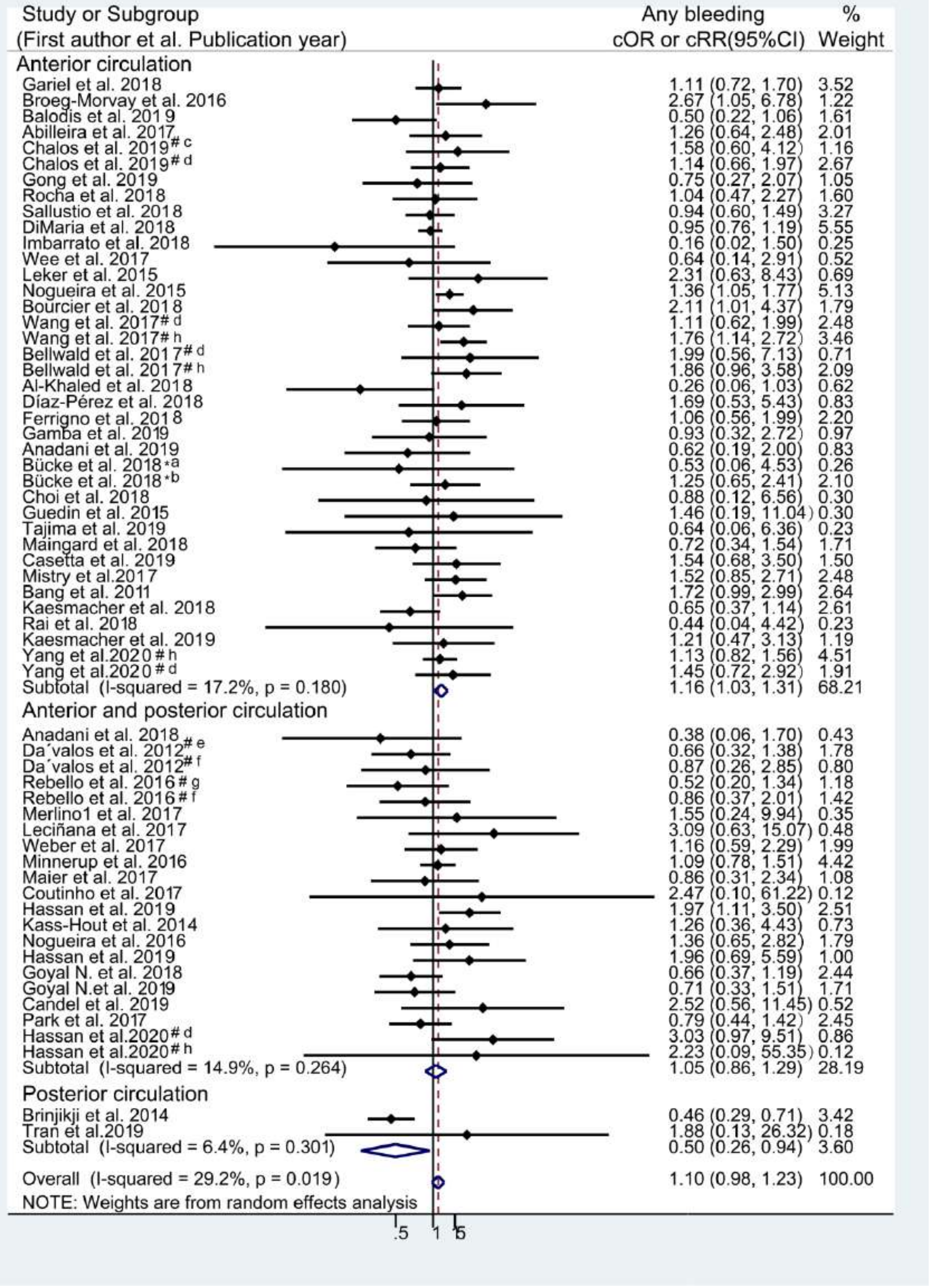
Study or Subgroup
(First author et al. Publication year)
Any bleeding
cOR or cRR(95%CI)
%
Weight
Anterior circulation
Gariel et al. 2018 1.11 (0.72, 1.70) 3.52
Broeg-Morvay et al. 2016 2.67 (1.05, 6.78) 1.22
Balodis et al. 2019 0.50 (0.22, 1.06) 1.61
Abilleira et al. 2017 1.26 (0.64, 2.48) 2.01
Chalos et al. 2019# c 1.58 (0.60, 4.12) 1.16
Chalos et al. 2019# d 1.14 (0.66, 1.97) 2.67
Gong et al. 2019 0.75 (0.27, 2.07) 1.05
Rocha et al. 2018 1.04 (0.47, 2.27) 1.60
Sallustio et al. 2018 0.94 (0.60, 1.49) 3.27
DiMaria et al. 2018 0.95 (0.76, 1.19) 5.55
Imbarrato et al. 2018 0.16 (0.02, 1.50) 0.25
Wee et al. 2017 0.64 (0.14, 2.91) 0.52
Leker et al. 2015 2.31 (0.63, 8.43) 0.69
Nogueira et al. 2015 1.36 (1.05, 1.77) 5.13
Bourcier et al. 2018 2.11 (1.01, 4.37) 1.79
Wang et al. 2017# d 1.11 (0.62, 1.99) 2.48
Wang et al. 2017# h 1.76 (1.14, 2.72) 3.46
Bellwald et al. 2017# d 1.99 (0.56, 7.13) 0.71
Bellwald et al. 2017# h 1.86 (0.96, 3.58) 2.09
Al-Khaled et al. 2018 0.26 (0.06, 1.03) 0.62
Díaz-Pérez et al. 2018 1.69 (0.53, 5.43) 0.83
Ferrigno et al. 2018 1.06 (0.56, 1.99) 2.20
Gamba et al. 2019 0.93 (0.32, 2.72) 0.97
Anadani et al. 2019 0.62 (0.19, 2.00) 0.83
Bücke et al. 2018*a 0.53 (0.06, 4.53) 0.26
Bücke et al. 2018*b 1.25 (0.65, 2.41) 2.10
Choi et al. 2018 0.88 (0.12, 6.56) 0.30
Guedin et al. 2015 1.46 (0.19, 11.04) 0.30
Tajima et al. 2019 0.64 (0.06, 6.36) 0.23
Maingard et al. 2018 0.72 (0.34, 1.54) 1.71
Casetta et al. 2019 1.54 (0.68, 3.50) 1.50
Mistry et al.2017 1.52 (0.85, 2.71) 2.48
Bang et al. 2011 1.72 (0.99, 2.99) 2.64
Kaesmacher et al. 2018 0.65 (0.37, 1.14) 2.61
Rai et al. 2018 0.44 (0.04, 4.42) 0.23
Kaesmacher et al. 2019 1.21 (0.47, 3.13) 1.19
Yang et al.2020 # h 1.13 (0.82, 1.56) 4.51
Yang et al.2020 # d 1.45 (0.72, 2.92) 1.91
Subtotal (I-squared = 17.2%, p = 0.180) 1.16 (1.03, 1.31) 68.21
Anterior and posterior circulation
Anadani et al. 2018 0.38 (0.06, 1.70) 0.43
Da´valos et al. 2012# e 0.66 (0.32, 1.38) 1.78
Da´valos et al. 2012# f 0.87 (0.26, 2.85) 0.80
Rebello et al. 2016# g 0.52 (0.20, 1.34) 1.18
Rebello et al. 2016# f 0.86 (0.37, 2.01) 1.42
Merlino1 et al. 2017 1.55 (0.24, 9.94) 0.35
Leciñana et al. 2017 3.09 (0.63, 15.07) 0.48
Weber et al. 2017 1.16 (0.59, 2.29) 1.99
Minnerup et al. 2016 1.09 (0.78, 1.51) 4.42
Maier et al. 2017 0.86 (0.31, 2.34) 1.08
Coutinho et al. 2017 2.47 (0.10, 61.22) 0.12
Hassan et al. 2019 1.97 (1.11, 3.50) 2.51
Kass-Hout et al. 2014 1.26 (0.36, 4.43) 0.73
Nogueira et al. 2016 1.36 (0.65, 2.82) 1.79
Hassan et al. 2019 1.96 (0.69, 5.59) 1.00
Goyal N. et al. 2018 0.66 (0.37, 1.19) 2.44
Goyal N.et al. 2019 0.71 (0.33, 1.51) 1.71
Candel et al. 2019 2.52 (0.56, 11.45) 0.52
Park et al. 2017 0.79 (0.44, 1.42) 2.45
Hassan et al.2020# d 3.03 (0.97, 9.51) 0.86
Hassan et al.2020# h 2.23 (0.09, 55.35) 0.12
Subtotal (I-squared = 14.9%, p = 0.264) 1.05 (0.86, 1.29) 28.19
Posterior circulation
Brinjikji et al. 2014 0.46 (0.29, 0.71) 3.42
Tran et al.2019 1.88 (0.13, 26.32) 0.18
Subtotal (I-squared = 6.4%, p = 0.301) 0.50 (0.26, 0.94) 3.60
Overall (I-squared = 29.2%, p = 0.019) 1.10 (0.98, 1.23) 100.00
NOTE: Weights are from random effects analysis
.5
1
5

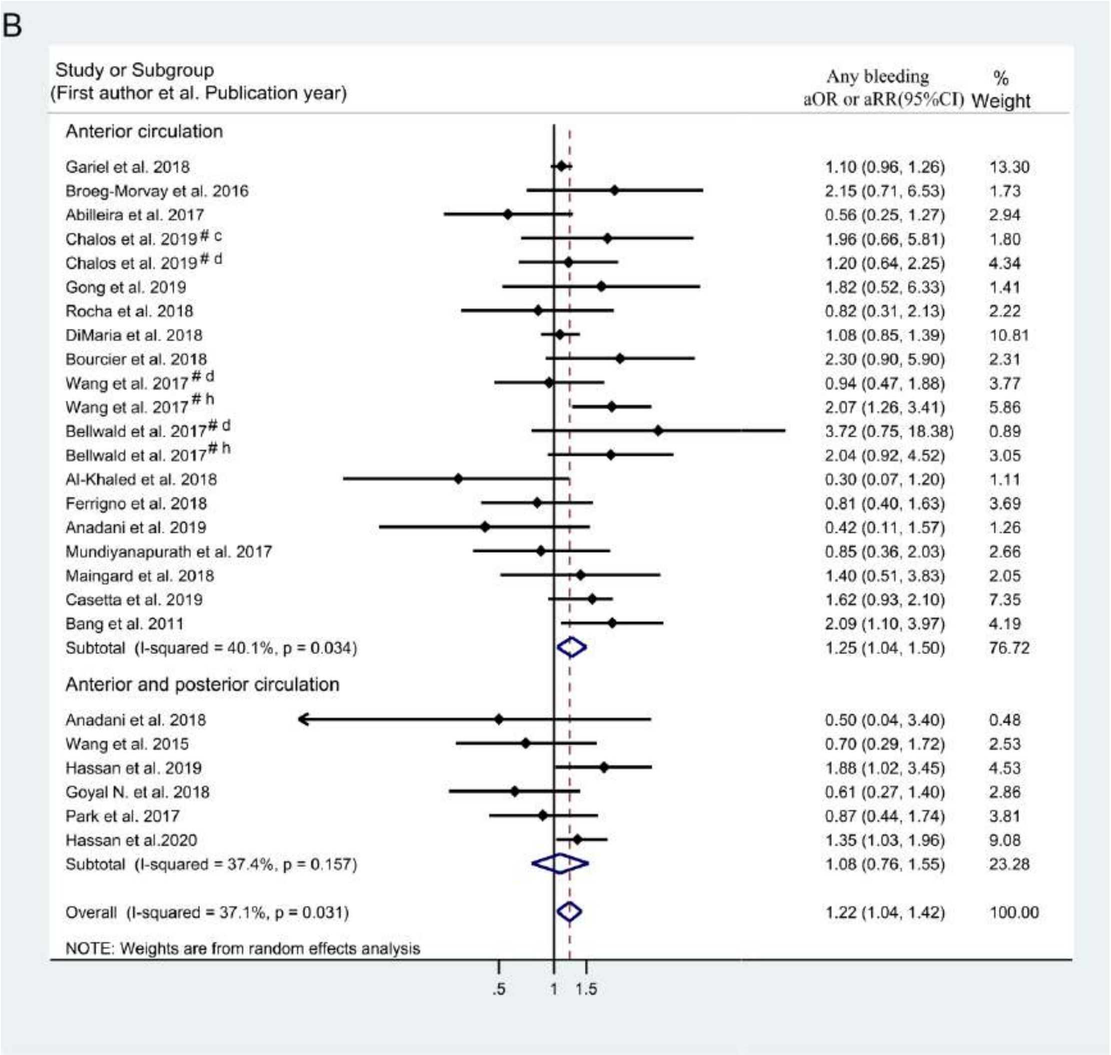
B
Study or Subgroup
(First author et al. Publication year)
Any bleeding
aOR or aRR(95%CI)
% Weight
Anterior circulation
Gariel et al. 2018 1.10 (0.96, 1.26) 13.30
Broeg-Morvay et al. 2016 2.15 (0.71, 6.53) 1.73
Abilleira et al. 2017 0.56 (0.25, 1.27) 2.94
Chalos et al. 2019# c 1.96 (0.66, 5.81) 1.80
Chalos et al. 2019# d 1.20 (0.64, 2.25) 4.34
Gong et al. 2019 1.82 (0.52, 6.33) 1.41
Rocha et al. 2018 0.82 (0.31, 2.13) 2.22
DiMaria et al. 2018 1.08 (0.85, 1.39) 10.81
Bourcier et al. 2018 2.30 (0.90, 5.90) 2.31
Wang et al. 2017# d 0.94 (0.47, 1.88) 3.77
Wang et al. 2017# h 2.07 (1.26, 3.41) 5.86
Bellwald et al. 2017# d 3.72 (0.75, 18.38) 0.89
Bellwald et al. 2017# h 2.04 (0.92, 4.52) 3.05
Al-Khaled et al. 2018 0.30 (0.07, 1.20) 1.11
Ferrigno et al. 2018 0.81 (0.40, 1.63) 3.69
Anadani et al. 2019 0.42 (0.11, 1.57) 1.26
Mundiyanapurath et al. 2017 0.85 (0.36, 2.03) 2.66
Maingard et al. 2018 1.40 (0.51, 3.83) 2.05
Casetta et al. 2019 1.62 (0.93, 2.10) 7.35
Bang et al. 2011 2.09 (1.10, 3.97) 4.19
Subtotal (I-squared = 40.1%, p = 0.034) 1.25 (1.04, 1.50) 76.72
Anterior and posterior circulation
Anadani et al. 2018 0.50 (0.04, 3.40) 0.48
Wang et al. 2015 0.70 (0.29, 1.72) 2.53
Hassan et al. 2019 1.88 (1.02, 3.45) 4.53
Goyal N. et al. 2018 0.61 (0.27, 1.40) 2.86
Park et al. 2017 0.87 (0.44, 1.74) 3.81
Hassan et al.2020 1.35 (1.03, 1.96) 9.08
Subtotal (I-squared = 37.4%, p = 0.157) 1.08 (0.76, 1.55) 23.28
Overall (I-squared = 37.1%, p = 0.031) 1.22 (1.04, 1.42) 100.00
NOTE: Weights are from random effects analysis
.5
1
1.5

**Supplemental Figure 57.** Forest plots of subgroup analysis assessing any bleeding by study type in unadjusted (A) and adjusted (B) analysis. ICA, internal carotid artery; sICH, symptomatic intracranial hemorrhage; aICH, asymptomatic intracranial hemorrhage; HT, hemorrhagic transformation; PH, parenchymal hematoma; SAH, subarachnoid hemorrhage. [*]The acute intracranial vessel occlusion patients with (a) or without (b) concomitant ipsilateral ICA-occlusion or high-grade stenosis were investigated respectively in the study by Bücke et al. 2018. [#]The outcomes of severe extracranial hemorrhage (c) and sICH (d) were investigated respectively in the study of Chalos et al. 2019; The outcomes of HT (e) and SAH (f) were investigated respectively in the study of Dávalos et al. 2012. The outcomes of PH (g) and SAH (f) were investigated respectively in the study of Rebello et al. 2016. The outcome of sICH (d) and aICH (h) were investigated respectively in the studies of Wang et al. 2017, Bellwald et al. 2017, Yang et al.2020 and Hassan et al.2020.

A

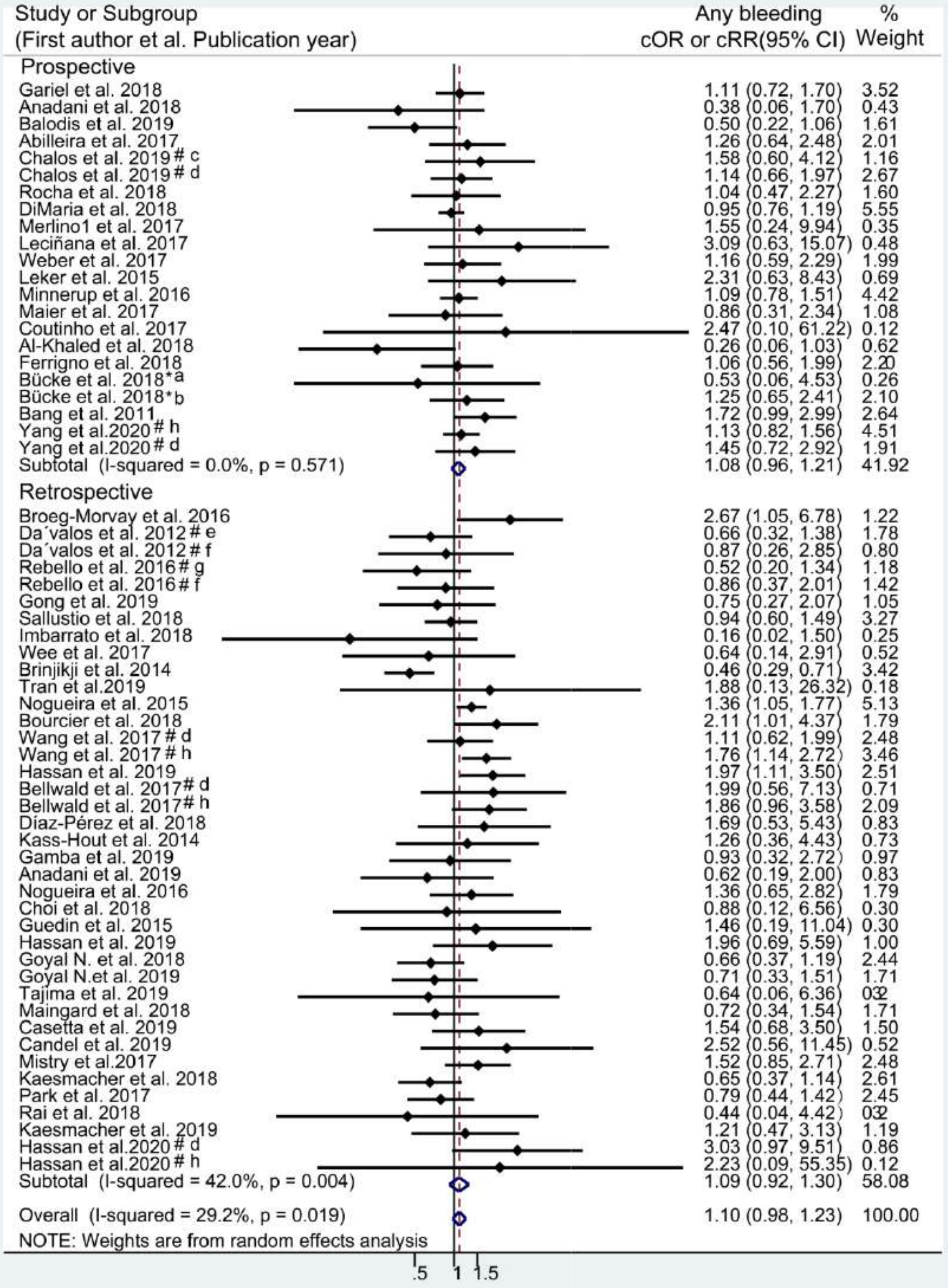

Study or Subgroup
(First author et al. Publication year)
Any bleeding cOR or cRR(95% CI)
% Weight
Prospective
Gariel et al. 2018 1.11 (0.72, 1.70) 3.52
Anadani et al. 2018 0.38 (0.06, 1.70) 0.43
Balodis et al. 2019 0.50 (0.22, 1.06) 1.61
Abilleira et al. 2017 1.26 (0.64, 2.48) 2.01
Chalos et al. 2019 # c 1.58 (0.60, 4.12) 1.16
Chalos et al. 2019 # d 1.14 (0.66, 1.97) 2.67
Rocha et al. 2018 1.04 (0.47, 2.27) 1.60
DiMaria et al. 2018 0.95 (0.76, 1.19) 5.55
Merlino1 et al. 2017 1.55 (0.24, 9.94) 0.35
Leciñana et al. 2017 3.09 (0.63, 15.07) 0.48
Weber et al. 2017 1.16 (0.59, 2.29) 1.99
Leker et al. 2015 2.31 (0.63, 8.43) 0.69
Minnerup et al. 2016 1.09 (0.78, 1.51) 4.42
Maier et al. 2017 0.86 (0.31, 2.34) 1.08
Coutinho et al. 2017 2.47 (0.10, 61.22) 0.12
Al-Khaled et al. 2018 0.26 (0.06, 1.03) 0.62
Ferrigno et al. 2018 1.06 (0.56, 1.99) 2.20
Bücke et al. 2018*a 0.53 (0.06, 4.53) 0.26
Bücke et al. 2018*b 1.25 (0.65, 2.41) 2.10
Bang et al. 2011 1.72 (0.99, 2.99) 2.64
Yang et al.2020 # h 1.13 (0.82, 1.56) 4.51
Yang et al.2020 # d 1.45 (0.72, 2.92) 1.91
Subtotal (I-squared = 0.0%, p = 0.571) 1.08 (0.96, 1.21) 41.92
Retrospective
Broeg-Morvay et al. 2016 2.67 (1.05, 6.78) 1.22
Da´valos et al. 2012 # e 0.66 (0.32, 1.38) 1.78
Da´valos et al. 2012 # f 0.87 (0.26, 2.85) 0.80
Rebello et al. 2016 # g 0.52 (0.20, 1.34) 1.18
Rebello et al. 2016 # f 0.86 (0.37, 2.01) 1.42
Gong et al. 2019 0.75 (0.27, 2.07) 1.05
Sallustio et al. 2018 0.94 (0.60, 1.49) 3.27
Imbarrato et al. 2018 0.16 (0.02, 1.50) 0.25
Wee et al. 2017 0.64 (0.14, 2.91) 0.52
Brinjikji et al. 2014 0.46 (0.29, 0.71) 3.42
Tran et al.2019 1.88 (0.13, 26.32) 0.18
Nogueira et al. 2015 1.36 (1.05, 1.77) 5.13
Bourcier et al. 2018 2.11 (1.01, 4.37) 1.79
Wang et al. 2017 # d 1.11 (0.62, 1.99) 2.48
Wang et al. 2017 # h 1.76 (1.14, 2.72) 3.46
Hassan et al. 2019 1.97 (1.11, 3.50) 2.51
Bellwald et al. 2017 # d 1.99 (0.56, 7.13) 0.71
Bellwald et al. 2017 # h 1.86 (0.96, 3.58) 2.09
Díaz-Pérez et al. 2018 1.69 (0.53, 5.43) 0.83
Kass-Hout et al. 2014 1.26 (0.36, 4.43) 0.73
Gamba et al. 2019 0.93 (0.32, 2.72) 0.97
Anadani et al. 2019 0.62 (0.19, 2.00) 0.83
Nogueira et al. 2016 1.36 (0.65, 2.82) 1.79
Choi et al. 2018 0.88 (0.12, 6.56) 0.30
Guedin et al. 2015 1.46 (0.19, 11.04) 0.30
Hassan et al. 2019 1.96 (0.69, 5.59) 1.00
Goyal N. et al. 2018 0.66 (0.37, 1.19) 2.44
Goyal N.et al. 2019 0.71 (0.33, 1.51) 1.71
Tajima et al. 2019 0.64 (0.06, 6.36) [illegible]
Maingard et al. 2018 0.72 (0.34, 1.54) 1.71
Casetta et al. 2019 1.54 (0.68, 3.50) 1.50
Candel et al. 2019 2.52 (0.56, 11.45) 0.52
Mistry et al.2017 1.52 (0.85, 2.71) 2.48
Kaesmacher et al. 2018 0.65 (0.37, 1.14) 2.61
Park et al. 2017 0.79 (0.44, 1.42) 2.45
Rai et al. 2018 0.44 (0.04, 4.42) [illegible]
Kaesmacher et al. 2019 1.21 (0.47, 3.13) 1.19
Hassan et al.2020 # d 3.03 (0.97, 9.51) 0.86
Hassan et al.2020 # h 2.23 (0.09, 55.35) 0.12
Subtotal (I-squared = 42.0%, p = 0.004) 1.09 (0.92, 1.30) 58.08
Overall (I-squared = 29.2%, p = 0.019) 1.10 (0.98, 1.23) 100.00
NOTE: Weights are from random effects analysis
.5
1
1.5

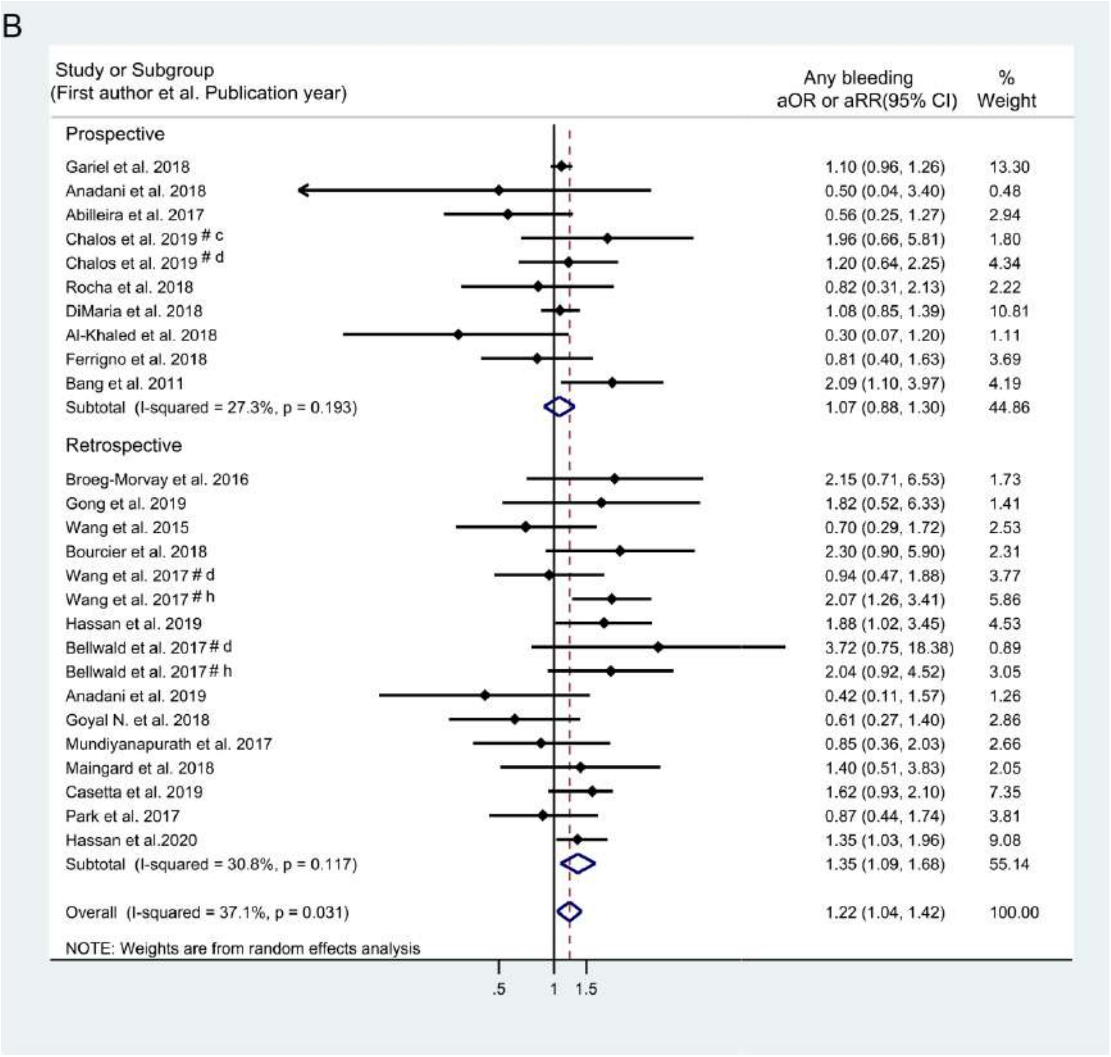
B
Study or Subgroup
(First author et al. Publication year)
Any bleeding
aOR or aRR(95% CI)
%
Weight
Prospective
Gariel et al. 2018 1.10 (0.96, 1.26) 13.30
Anadani et al. 2018 0.50 (0.04, 3.40) 0.48
Abilleira et al. 2017 0.56 (0.25, 1.27) 2.94
Chalos et al. 2019 # c 1.96 (0.66, 5.81) 1.80
Chalos et al. 2019 # d 1.20 (0.64, 2.25) 4.34
Rocha et al. 2018 0.82 (0.31, 2.13) 2.22
DiMaria et al. 2018 1.08 (0.85, 1.39) 10.81
Al-Khaled et al. 2018 0.30 (0.07, 1.20) 1.11
Ferrigno et al. 2018 0.81 (0.40, 1.63) 3.69
Bang et al. 2011 2.09 (1.10, 3.97) 4.19
Subtotal (I-squared = 27.3%, p = 0.193) 1.07 (0.88, 1.30) 44.86
Retrospective
Broeg-Morvay et al. 2016 2.15 (0.71, 6.53) 1.73
Gong et al. 2019 1.82 (0.52, 6.33) 1.41
Wang et al. 2015 0.70 (0.29, 1.72) 2.53
Bourcier et al. 2018 2.30 (0.90, 5.90) 2.31
Wang et al. 2017 # d 0.94 (0.47, 1.88) 3.77
Wang et al. 2017 # h 2.07 (1.26, 3.41) 5.86
Hassan et al. 2019 1.88 (1.02, 3.45) 4.53
Bellwald et al. 2017 # d 3.72 (0.75, 18.38) 0.89
Bellwald et al. 2017 # h 2.04 (0.92, 4.52) 3.05
Anadani et al. 2019 0.42 (0.11, 1.57) 1.26
Goyal N. et al. 2018 0.61 (0.27, 1.40) 2.86
Mundiyanapurath et al. 2017 0.85 (0.36, 2.03) 2.66
Maingard et al. 2018 1.40 (0.51, 3.83) 2.05
Casetta et al. 2019 1.62 (0.93, 2.10) 7.35
Park et al. 2017 0.87 (0.44, 1.74) 3.81
Hassan et al.2020 1.35 (1.03, 1.96) 9.08
Subtotal (I-squared = 30.8%, p = 0.117) 1.35 (1.09, 1.68) 55.14
Overall (I-squared = 37.1%, p = 0.031) 1.22 (1.04, 1.42) 100.00
NOTE: Weights are from random effects analysis
.5
1
1.5

**Supplemental Figure 58.** Forest plots of subgroup analysis assessing any bleeding by ethnicity in unadjusted (A) and adjusted (B) analysis. ICA, internal carotid artery; sICH, symptomatic intracranial hemorrhage; aICH, asymptomatic intracranial hemorrhage; HT, hemorrhagic transformation; PH, parenchymal hematoma; SAH, subarachnoid hemorrhage. *The acute intracranial vessel occlusion patients with (a) or without (b) concomitant ipsilateral ICA-occlusion or high-grade stenosis were investigated respectively in the study by Bücke et al. 2018. #The outcomes of severe extracranial hemorrhage (c) and sICH (d) were investigated respectively in the study of Chalos et al. 2019; The outcomes of HT (e) and SAH (f) were investigated respectively in the study of Dávalos et al. 2012. The outcomes of PH (g) and SAH (f) were investigated respectively in the study of Rebello et al. 2016. The outcome of sICH (d) and aICH (h) were investigated respectively in the studies of Wang et al. 2017, Bellwald et al. 2017, Yang et al.2020 and Hassan et al.2020.

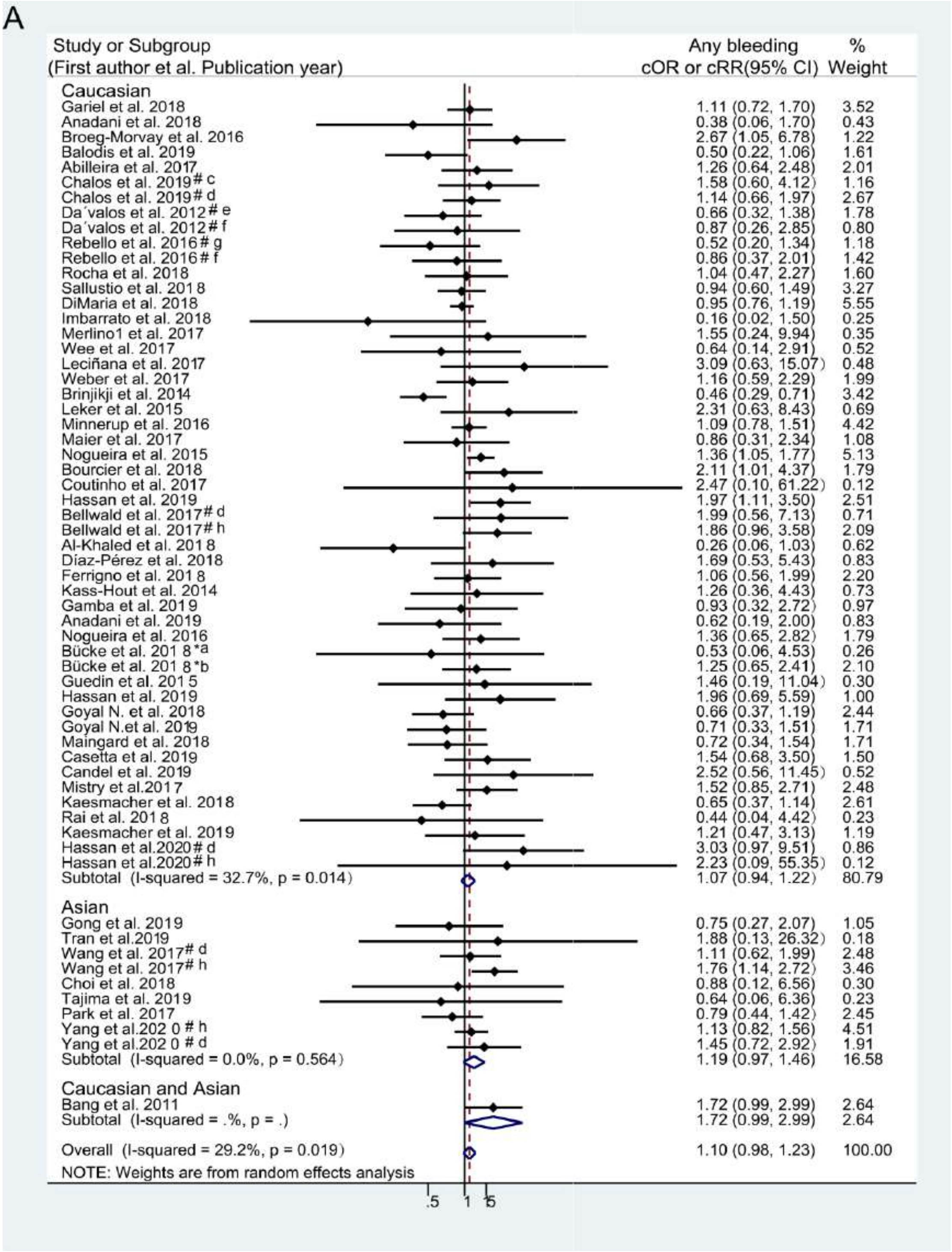
A
Study or Subgroup
(First author et al. Publication year)
Any bleeding
cOR or cRR(95% CI)
%
Weight
Caucasian
Gariel et al. 2018 1.11 (0.72, 1.70) 3.52
Anadani et al. 2018 0.38 (0.06, 1.70) 0.43
Broeg-Morvay et al. 2016 2.67 (1.05, 6.78) 1.22
Balodis et al. 2019 0.50 (0.22, 1.06) 1.61
Abilleira et al. 2017 1.26 (0.64, 2.48) 2.01
Chalos et al. 2019# c 1.58 (0.60, 4.12) 1.16
Chalos et al. 2019# d 1.14 (0.66, 1.97) 2.67
Da´valos et al. 2012# e 0.66 (0.32, 1.38) 1.78
Da´valos et al. 2012# f 0.87 (0.26, 2.85) 0.80
Rebello et al. 2016# g 0.52 (0.20, 1.34) 1.18
Rebello et al. 2016# f 0.86 (0.37, 2.01) 1.42
Rocha et al. 2018 1.04 (0.47, 2.27) 1.60
Sallustio et al. 2018 0.94 (0.60, 1.49) 3.27
DiMaria et al. 2018 0.95 (0.76, 1.19) 5.55
Imbarrato et al. 2018 0.16 (0.02, 1.50) 0.25
Merlino1 et al. 2017 1.55 (0.24, 9.94) 0.35
Wee et al. 2017 0.64 (0.14, 2.91) 0.52
Leciñana et al. 2017 3.09 (0.63, 15.07) 0.48
Weber et al. 2017 1.16 (0.59, 2.29) 1.99
Brinjikji et al. 2014 0.46 (0.29, 0.71) 3.42
Leker et al. 2015 2.31 (0.63, 8.43) 0.69
Minnerup et al. 2016 1.09 (0.78, 1.51) 4.42
Maier et al. 2017 0.86 (0.31, 2.34) 1.08
Nogueira et al. 2015 1.36 (1.05, 1.77) 5.13
Bourcier et al. 2018 2.11 (1.01, 4.37) 1.79
Coutinho et al. 2017 2.47 (0.10, 61.22) 0.12
Hassan et al. 2019 1.97 (1.11, 3.50) 2.51
Bellwald et al. 2017# d 1.99 (0.56, 7.13) 0.71
Bellwald et al. 2017# h 1.86 (0.96, 3.58) 2.09
Al-Khaled et al. 2018 0.26 (0.06, 1.03) 0.62
Díaz-Pérez et al. 2018 1.69 (0.53, 5.43) 0.83
Ferrigno et al. 2018 1.06 (0.56, 1.99) 2.20
Kass-Hout et al. 2014 1.26 (0.36, 4.43) 0.73
Gamba et al. 2019 0.93 (0.32, 2.72) 0.97
Anadani et al. 2019 0.62 (0.19, 2.00) 0.83
Nogueira et al. 2016 1.36 (0.65, 2.82) 1.79
Bücke et al. 2018*a 0.53 (0.06, 4.53) 0.26
Bücke et al. 2018*b 1.25 (0.65, 2.41) 2.10
Guedin et al. 2015 1.46 (0.19, 11.04) 0.30
Hassan et al. 2019 1.96 (0.69, 5.59) 1.00
Goyal N. et al. 2018 0.66 (0.37, 1.19) 2.44
Goyal N.et al. 2019 0.71 (0.33, 1.51) 1.71
Maingard et al. 2018 0.72 (0.34, 1.54) 1.71
Casetta et al. 2019 1.54 (0.68, 3.50) 1.50
Candel et al. 2019 2.52 (0.56, 11.45) 0.52
Mistry et al.2017 1.52 (0.85, 2.71) 2.48
Kaesmacher et al. 2018 0.65 (0.37, 1.14) 2.61
Rai et al. 2018 0.44 (0.04, 4.42) 0.23
Kaesmacher et al. 2019 1.21 (0.47, 3.13) 1.19
Hassan et al.2020# d 3.03 (0.97, 9.51) 0.86
Hassan et al.2020# h 2.23 (0.09, 55.35) 0.12
Subtotal (I-squared = 32.7%, p = 0.014) 1.07 (0.94, 1.22) 80.79
Asian
Gong et al. 2019 0.75 (0.27, 2.07) 1.05
Tran et al.2019 1.88 (0.13, 26.32) 0.18
Wang et al. 2017# d 1.11 (0.62, 1.99) 2.48
Wang et al. 2017# h 1.76 (1.14, 2.72) 3.46
Choi et al. 2018 0.88 (0.12, 6.56) 0.30
Tajima et al. 2019 0.64 (0.06, 6.36) 0.23
Park et al. 2017 0.79 (0.44, 1.42) 2.45
Yang et al.2020 # h 1.13 (0.82, 1.56) 4.51
Yang et al.2020 # d 1.45 (0.72, 2.92) 1.91
Subtotal (I-squared = 0.0%, p = 0.564) 1.19 (0.97, 1.46) 16.58
Caucasian and Asian
Bang et al. 2011 1.72 (0.99, 2.99) 2.64
Subtotal (I-squared = .%, p = .) 1.72 (0.99, 2.99) 2.64
Overall (I-squared = 29.2%, p = 0.019) 1.10 (0.98, 1.23) 100.00
NOTE: Weights are from random effects analysis
.5
1
5

B

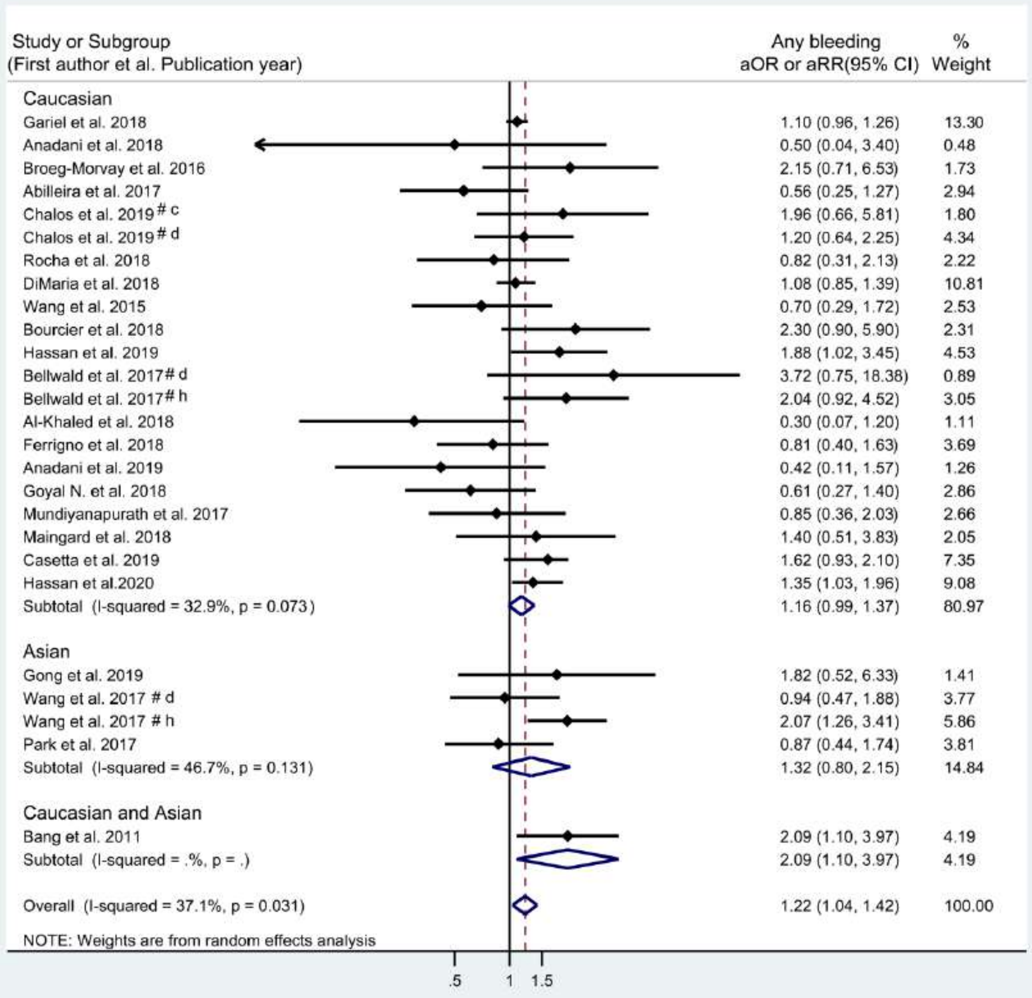

Study or Subgroup
(First author et al. Publication year)
Any bleeding
aOR or aRR(95% CI)
% Weight
Caucasian
Gariel et al. 2018 1.10 (0.96, 1.26) 13.30
Anadani et al. 2018 0.50 (0.04, 3.40) 0.48
Broeg-Morvay et al. 2016 2.15 (0.71, 6.53) 1.73
Abilleira et al. 2017 0.56 (0.25, 1.27) 2.94
Chalos et al. 2019 # c 1.96 (0.66, 5.81) 1.80
Chalos et al. 2019 # d 1.20 (0.64, 2.25) 4.34
Rocha et al. 2018 0.82 (0.31, 2.13) 2.22
DiMaria et al. 2018 1.08 (0.85, 1.39) 10.81
Wang et al. 2015 0.70 (0.29, 1.72) 2.53
Bourcier et al. 2018 2.30 (0.90, 5.90) 2.31
Hassan et al. 2019 1.88 (1.02, 3.45) 4.53
Bellwald et al. 2017# d 3.72 (0.75, 18.38) 0.89
Bellwald et al. 2017# h 2.04 (0.92, 4.52) 3.05
Al-Khaled et al. 2018 0.30 (0.07, 1.20) 1.11
Ferrigno et al. 2018 0.81 (0.40, 1.63) 3.69
Anadani et al. 2019 0.42 (0.11, 1.57) 1.26
Goyal N. et al. 2018 0.61 (0.27, 1.40) 2.86
Mundiyanapurath et al. 2017 0.85 (0.36, 2.03) 2.66
Maingard et al. 2018 1.40 (0.51, 3.83) 2.05
Casetta et al. 2019 1.62 (0.93, 2.10) 7.35
Hassan et al.2020 1.35 (1.03, 1.96) 9.08
Subtotal (I-squared = 32.9%, p = 0.073) 1.16 (0.99, 1.37) 80.97
Asian
Gong et al. 2019 1.82 (0.52, 6.33) 1.41
Wang et al. 2017 # d 0.94 (0.47, 1.88) 3.77
Wang et al. 2017 # h 2.07 (1.26, 3.41) 5.86
Park et al. 2017 0.87 (0.44, 1.74) 3.81
Subtotal (I-squared = 46.7%, p = 0.131) 1.32 (0.80, 2.15) 14.84
Caucasian and Asian
Bang et al. 2011 2.09 (1.10, 3.97) 4.19
Subtotal (I-squared = .%, p = .) 2.09 (1.10, 3.97) 4.19
Overall (I-squared = 37.1%, p = 0.031) 1.22 (1.04, 1.42) 100.00
NOTE: Weights are from random effects analysis
.5
1
1.5

**Supplemental Figure 59.** Forest plots of studies assessing SAH in unadjusted analysis. SAH, subarachnoid hemorrhage.

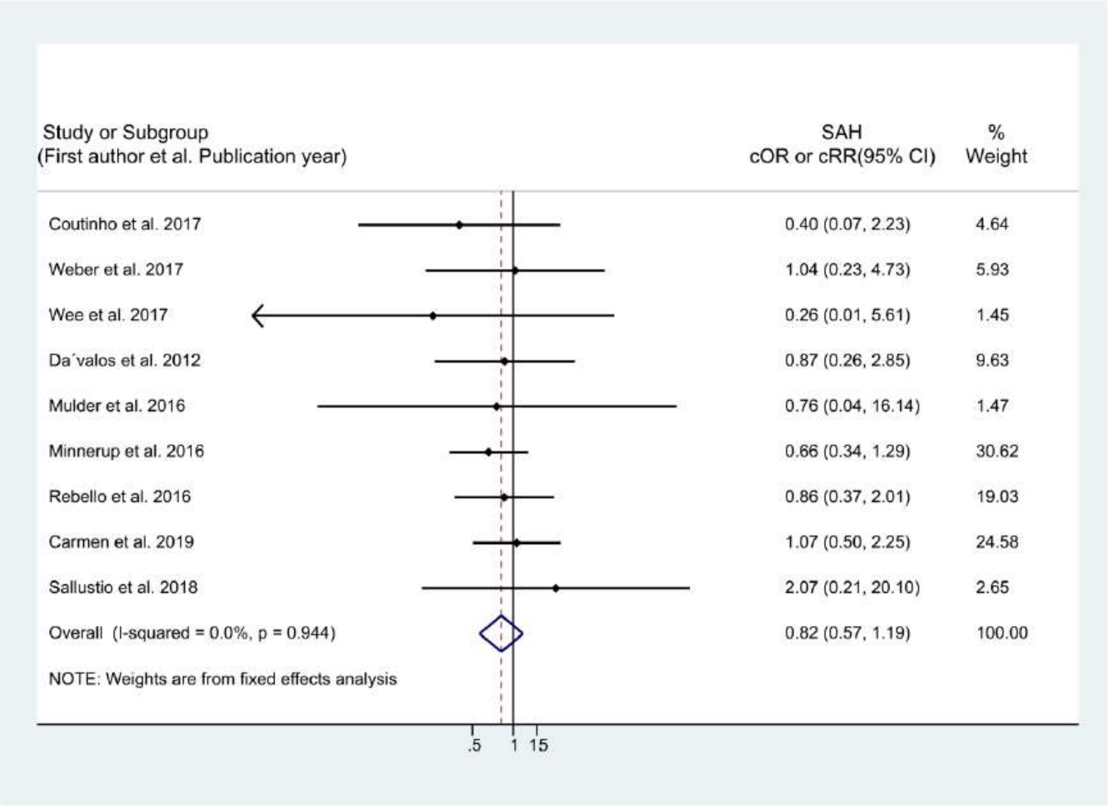

**Supplemental Figure 60.** Forest plots of subgroup analysis assessing SAH by location of occluded artery (A) and study type (B). SAH, subarachnoid hemorrhage.

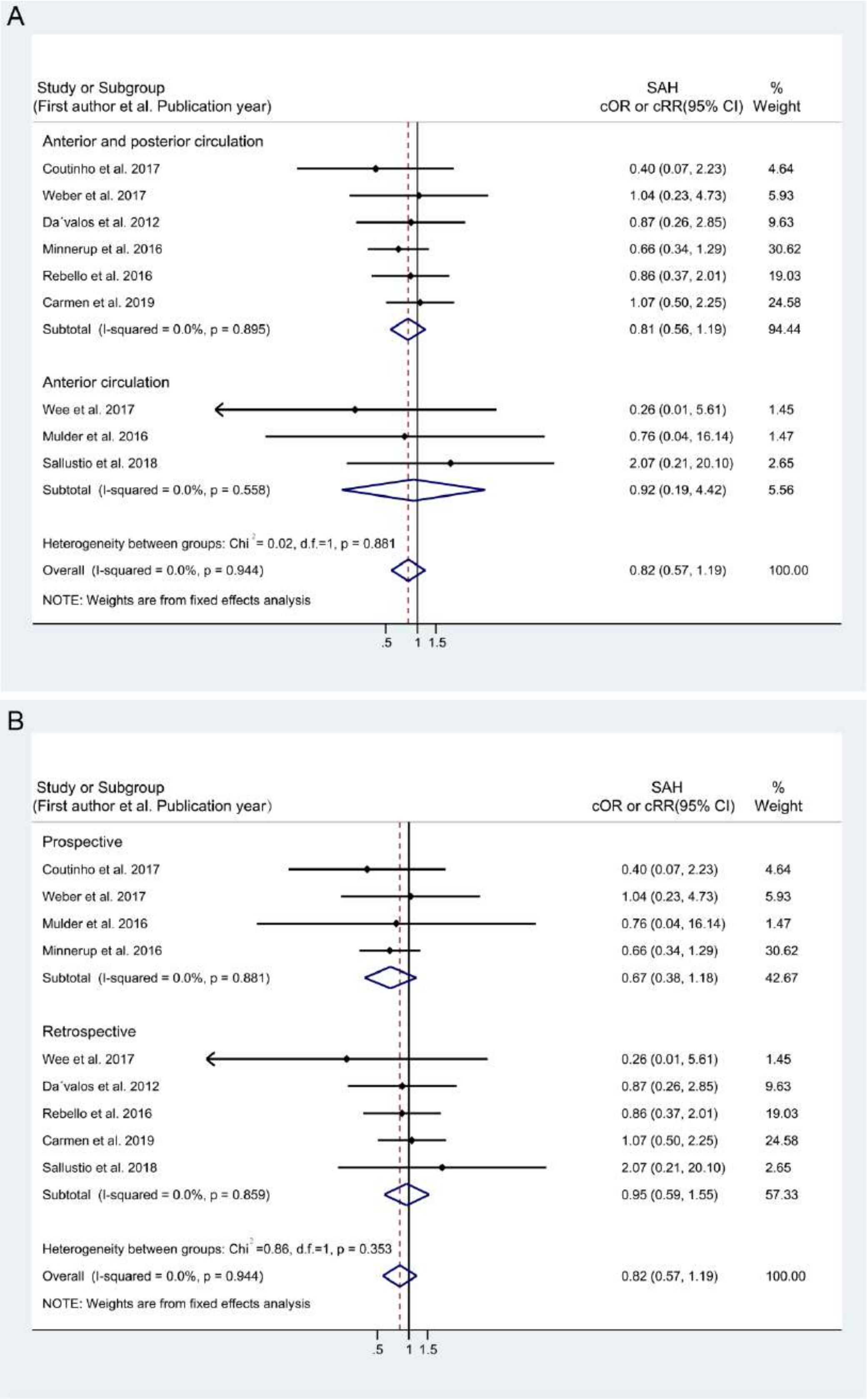

**Supplemental Figure 61.** Forest plots of studies assessing HI (A), HI-1 and HI-2 (B) in unadjusted analysis. HI, hemorrhagic infarction. [#]The outcomes of HI-1(a) and HI-2 (b) were investigated respectively in the studies of Coutinho et al. 2017, Sallustio et al. 2018, Leciñana et al. 2017, Da´valos et al. 2012 and Mulder et al. 2016.

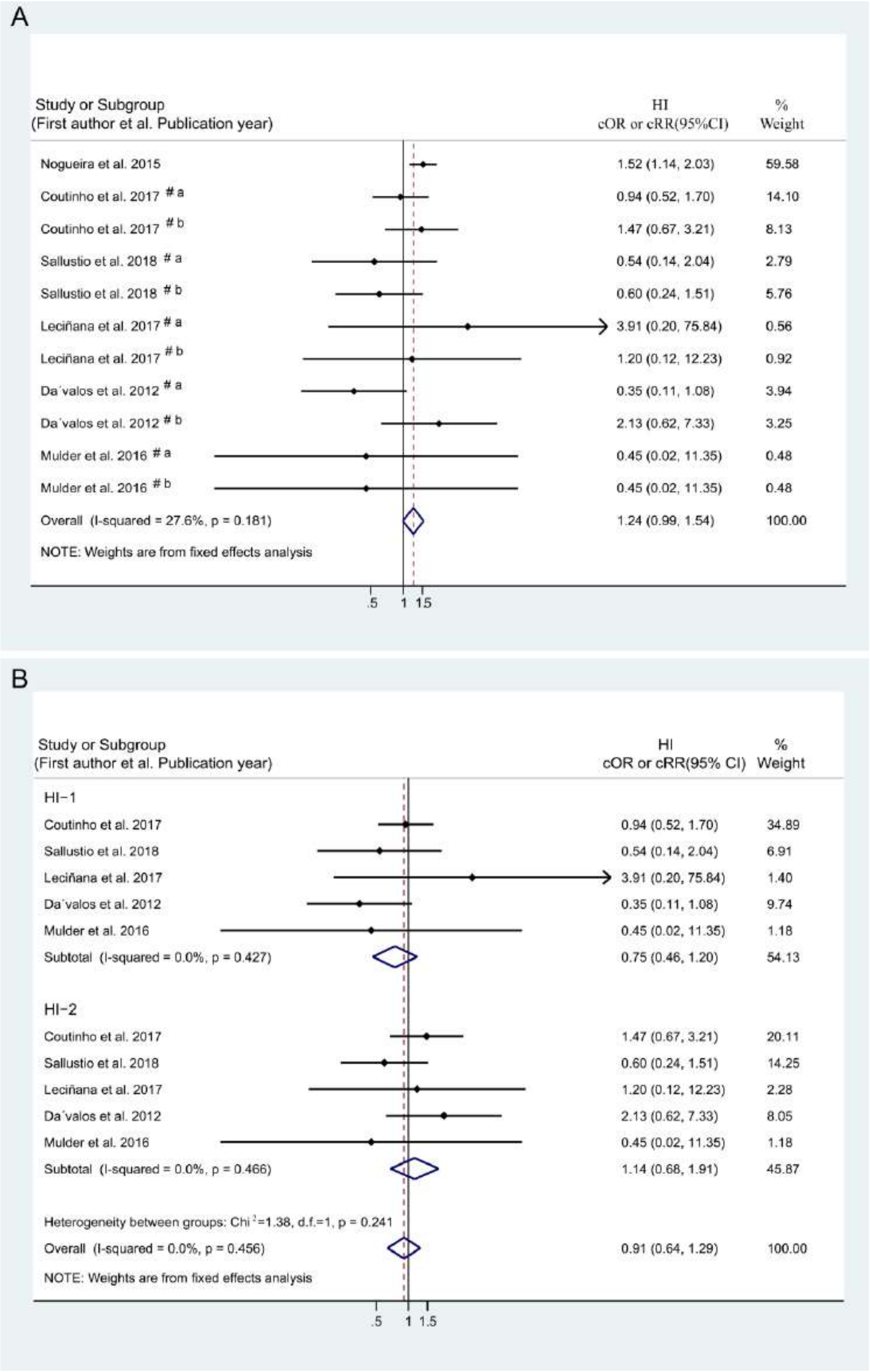

**Supplemental Figure 62.** Forest plots of subgroup analysis assessing HI by location of occluded artery (A) and study type (B). HI, hemorrhagic infarction. #The outcomes of HI-1(a) and HI-2 (b) were investigated respectively in the studies of Coutinho et al. 2017, Sallustio et al. 2018, Leciñana et al. 2017, Dávalos et al. 2012 and Mulder et al. 2016.

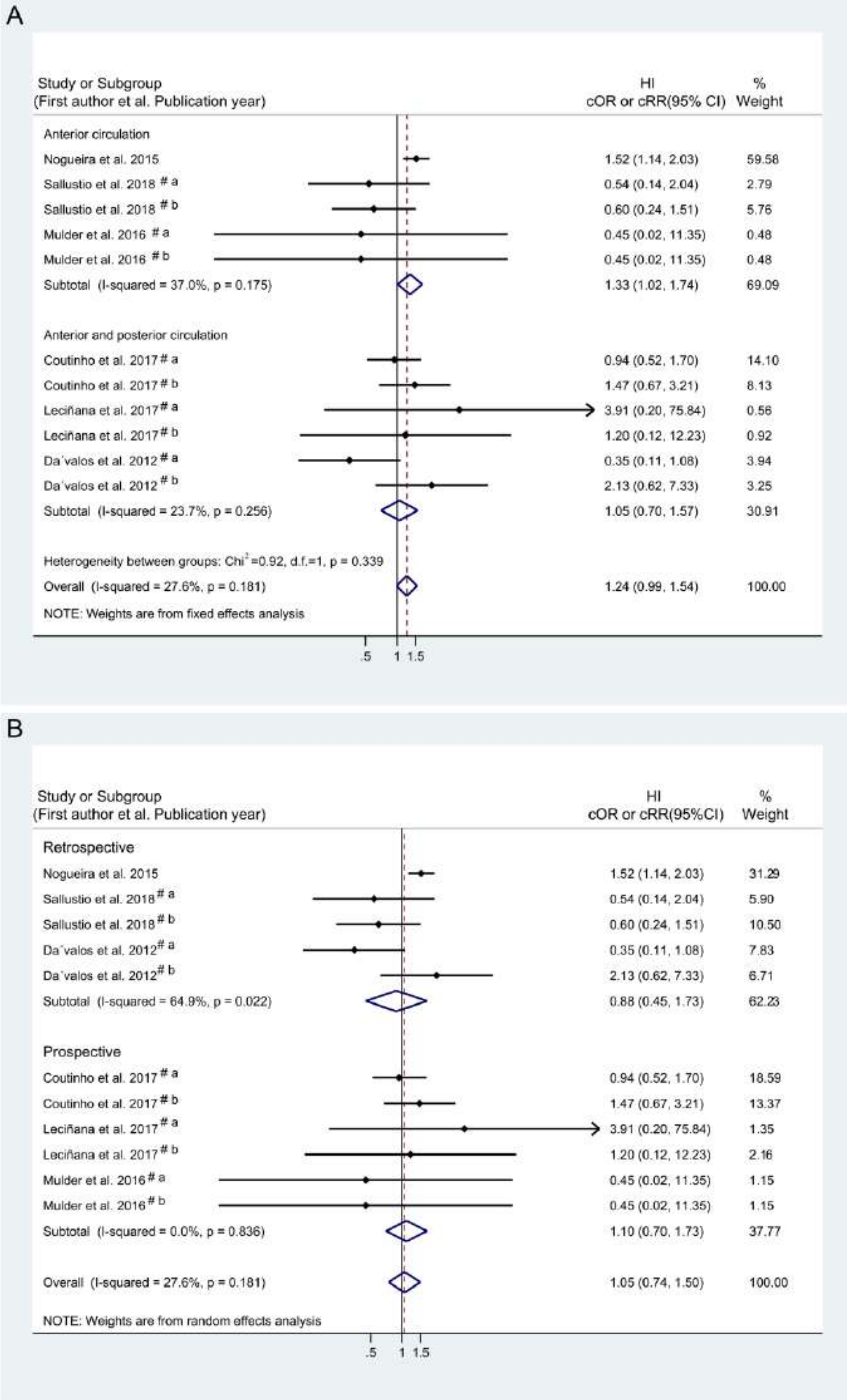

**Supplemental Figure 63.** Forest plots of studies assessing PH in unadjusted (A) and adjusted analysis (B). PH, parenchymal hematoma. [#]The outcomes of PH-1(a) and PH-2 (b) were investigated respectively in the studies of Leciñana et al. 2017, Casetta et al. 2019, Sallustio et al. 2018 and Da´valos et al. 2012.

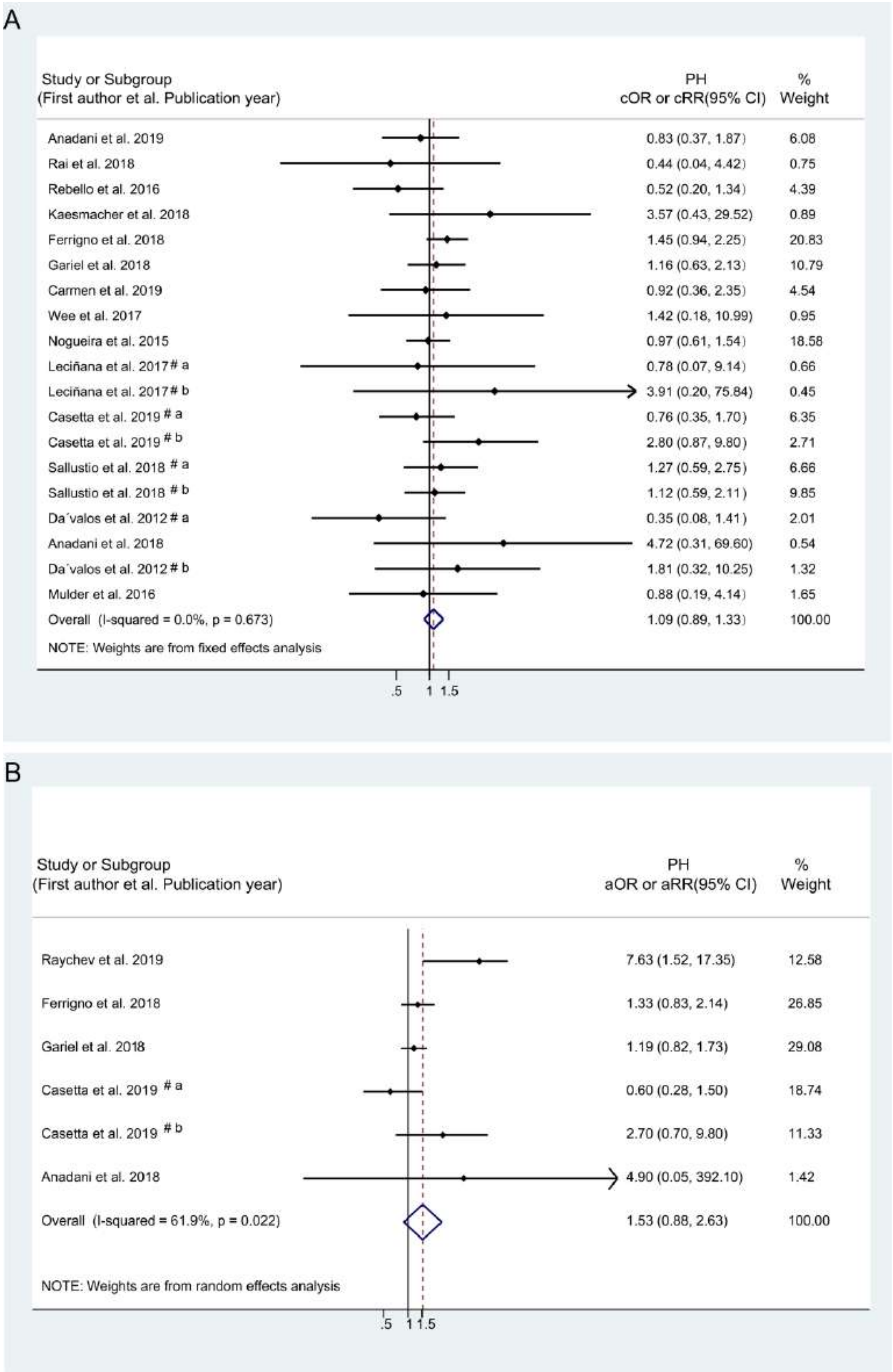

**Supplemental Figure 64.** Forest plots of subgroup analysis assessing PH by location of occluded artery in unadjusted (A) and adjusted analysis (B). PH, parenchymal hematoma. [#]The outcomes of PH-1(a) and PH-2 (b) were investigated respectively in the studies of Leciñana et al. 2017, Casetta et al. 2019, Sallustio et al. 2018 and Dávalos et al. 2012.

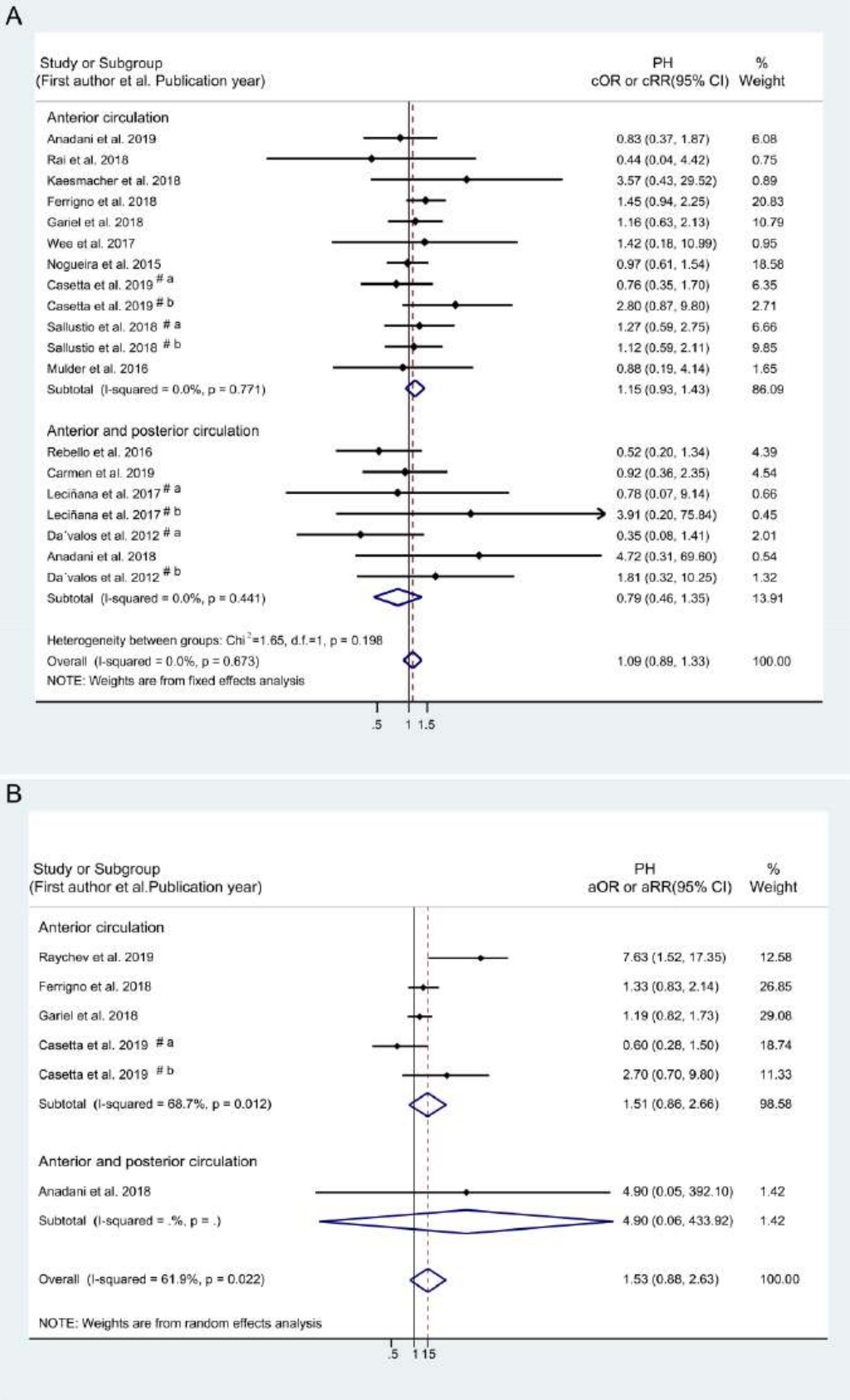

**Supplemental Figure 65.** Forest plots of subgroup analysis assessing PH by study type in unadjusted (A) and adjusted analysis (B). PH, parenchymal hematoma. [#]The outcomes of PH-1 (a) and PH-2 (b) were investigated respectively in the studies of Leciñana et al. 2017, Casetta et al. 2019, Sallustio et al. 2018 and Dávalos et al. 2012.

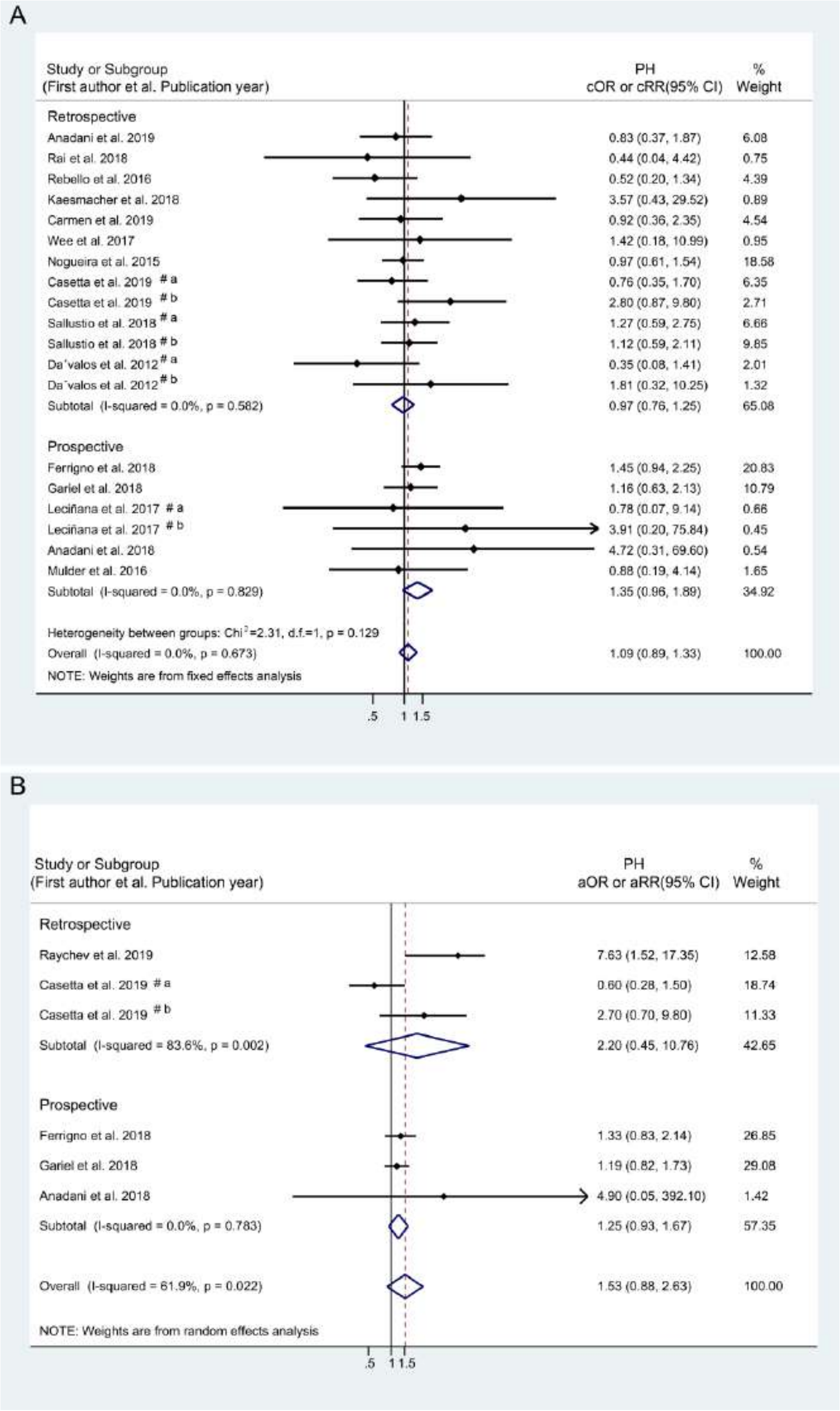

**Supplemental Figure 66.** Forest plots of studies assessing PH-1 in unadjusted analysis (A), and PH-2 in unadjusted (A) and adjusted analysis (B). PH, parenchymal hematoma.

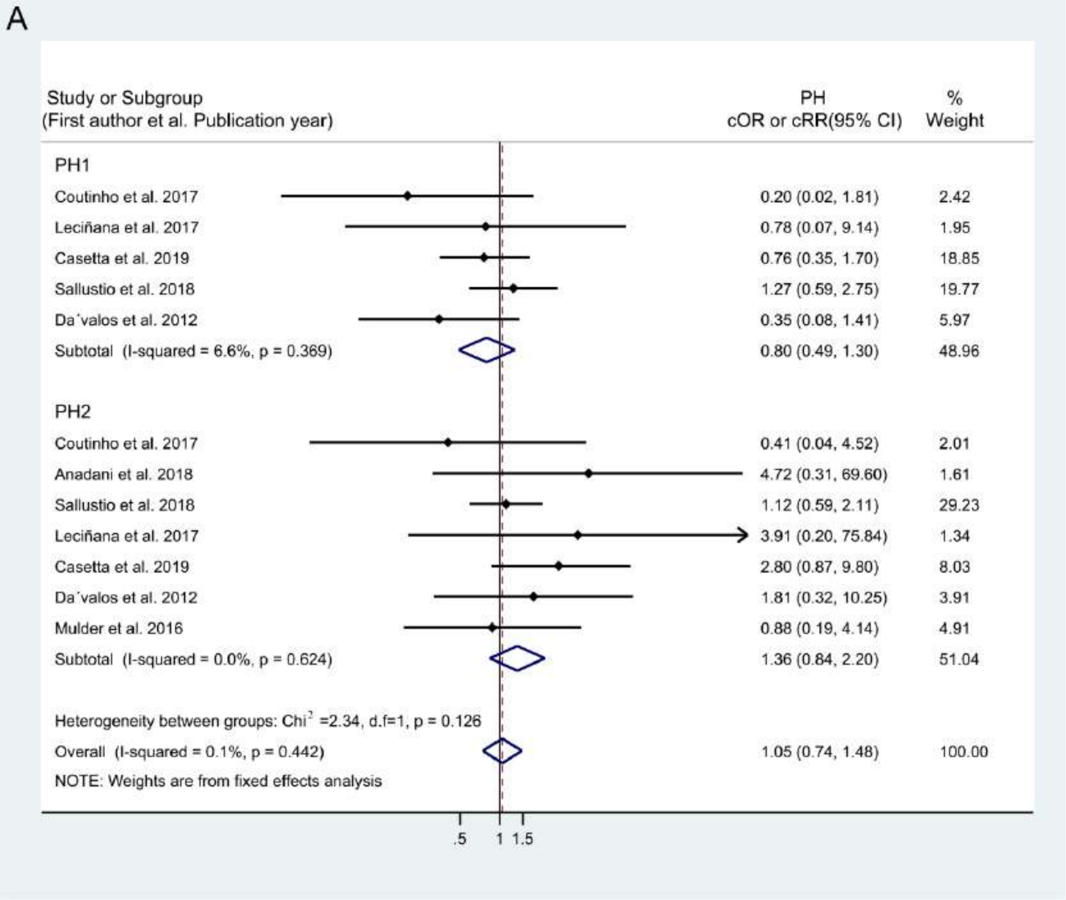


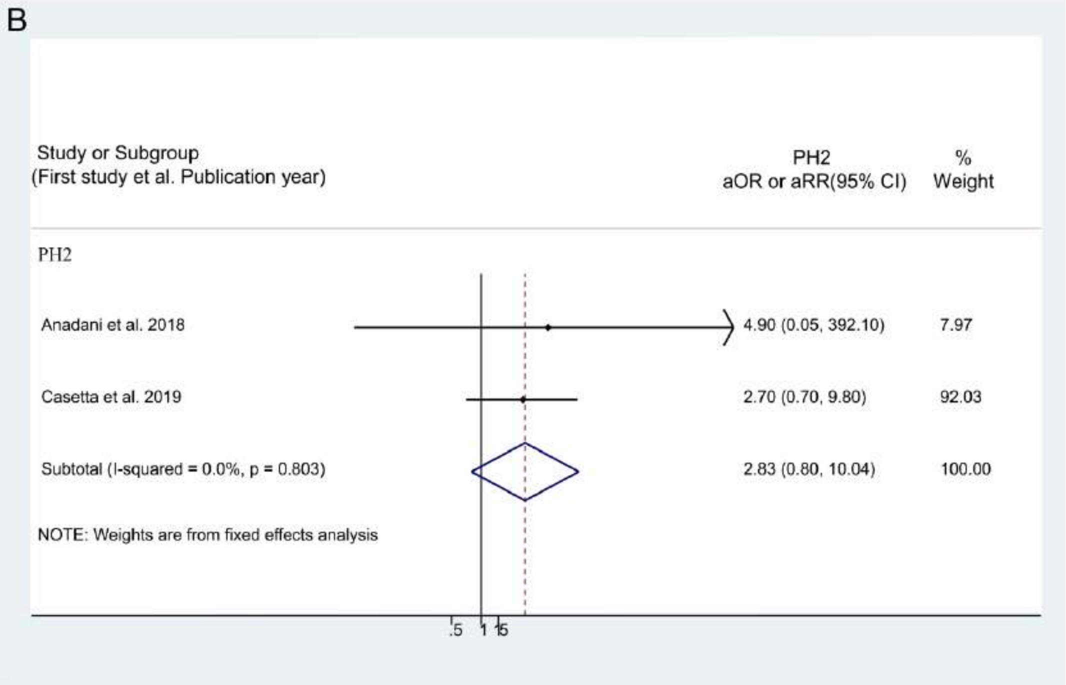

**Supplemental Figure 67.** Forest plots of studies assessing dramatic improvement at discharge/7days in unadjusted analysis.

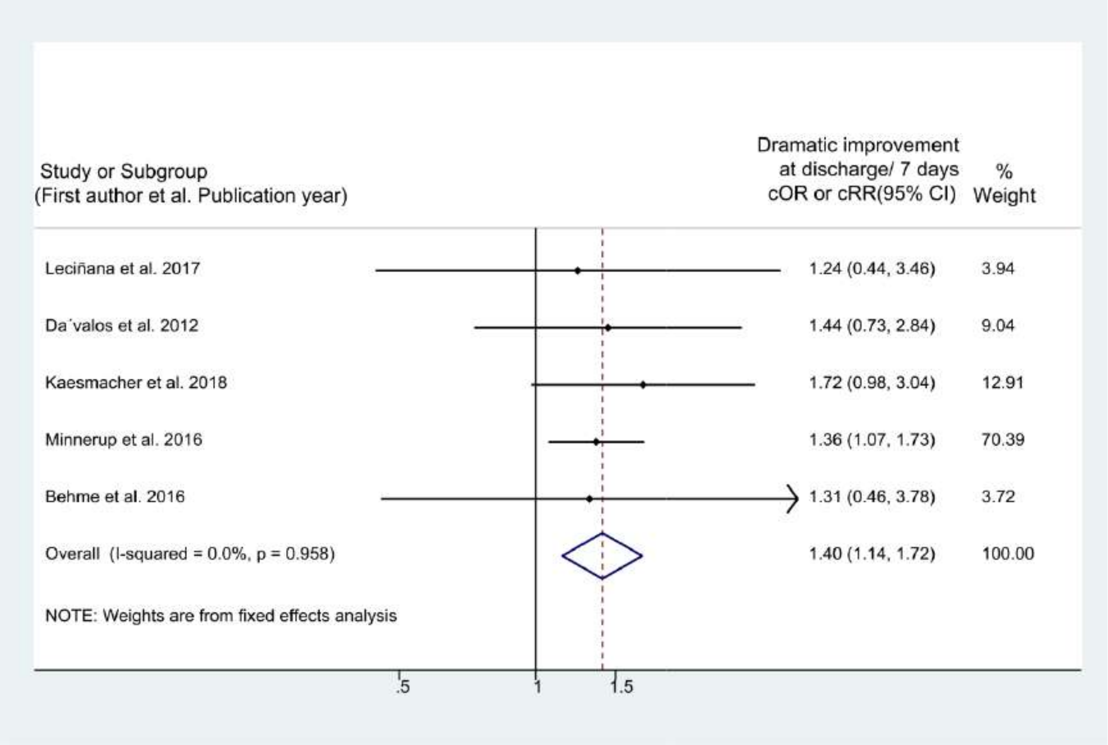

**Supplemental Figure 68.** Forest plots of studies assessing good improvement at discharge/7 days (A) and at 3 months (B) in unadjusted analysis.

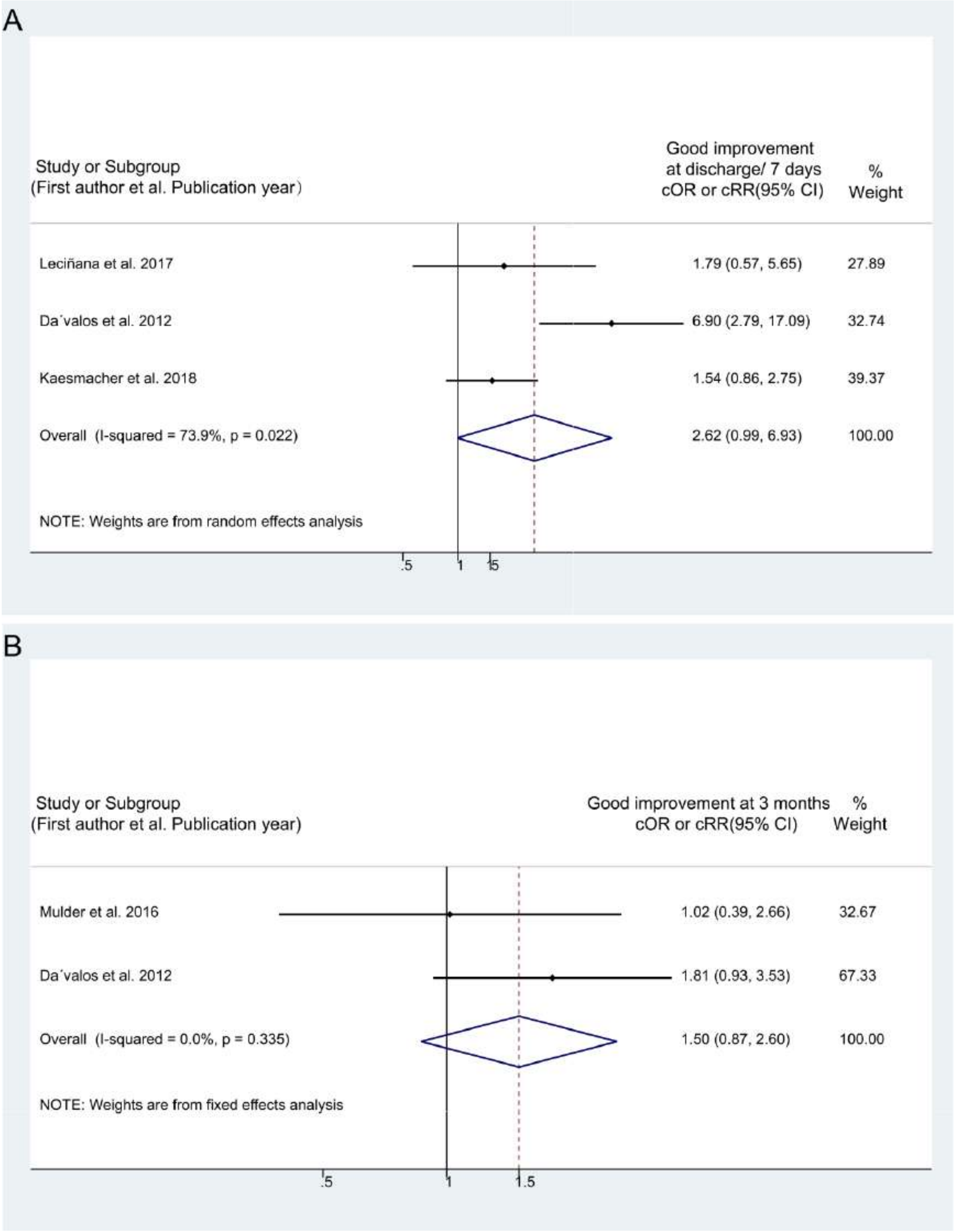

**Supplemental Figure 69.** Forest plots of subgroup analysis assessing dramatic improvement at discharge/7days by location of occluded artery (A) and study type (B) in unadjusted analysis.

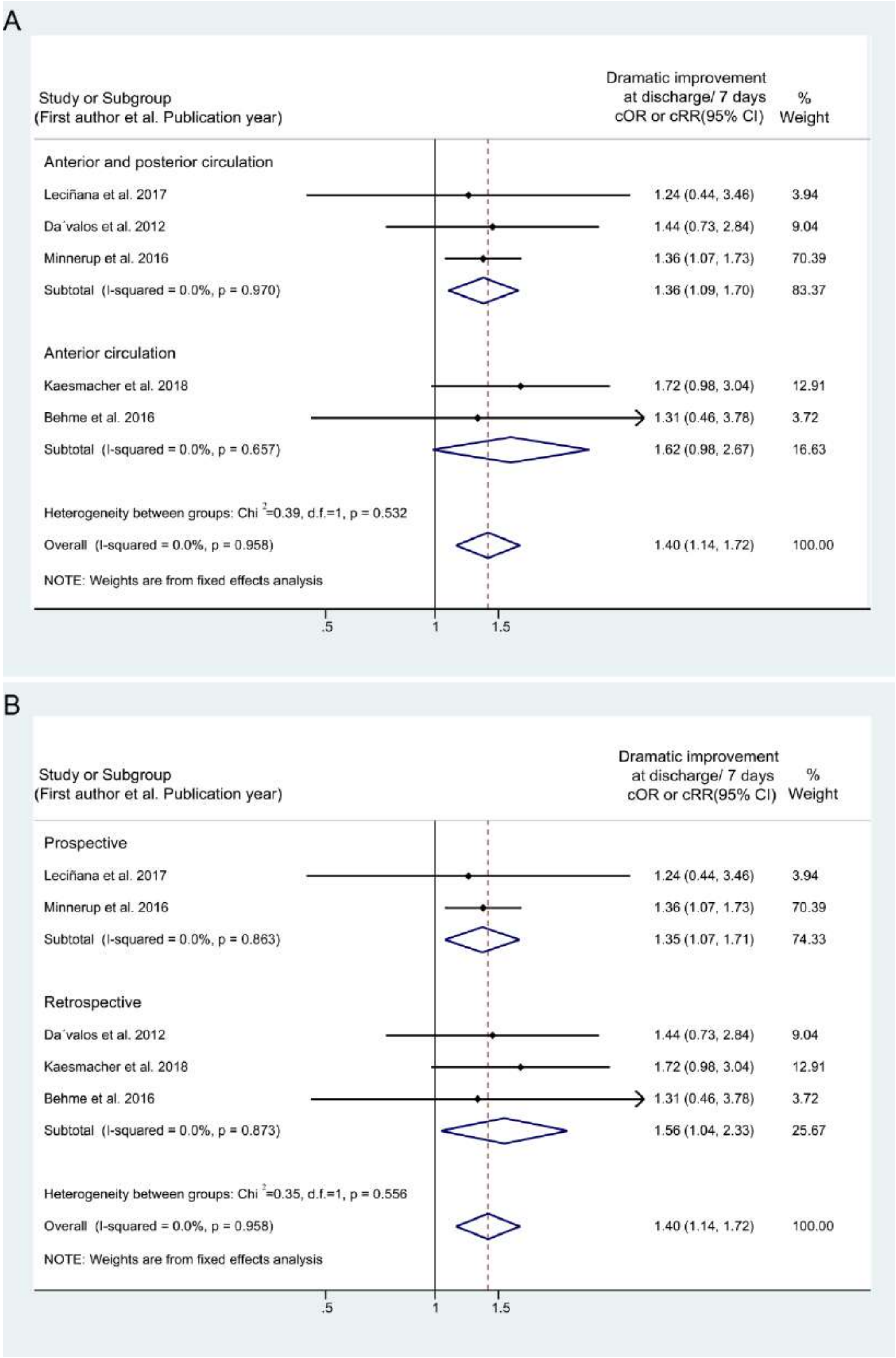

**Supplemental Figure 70.** Forest plots of subgroup analysis assessing early recovery at 24h by ethnicity in unadjusted (A) and adjusted analysis (B).

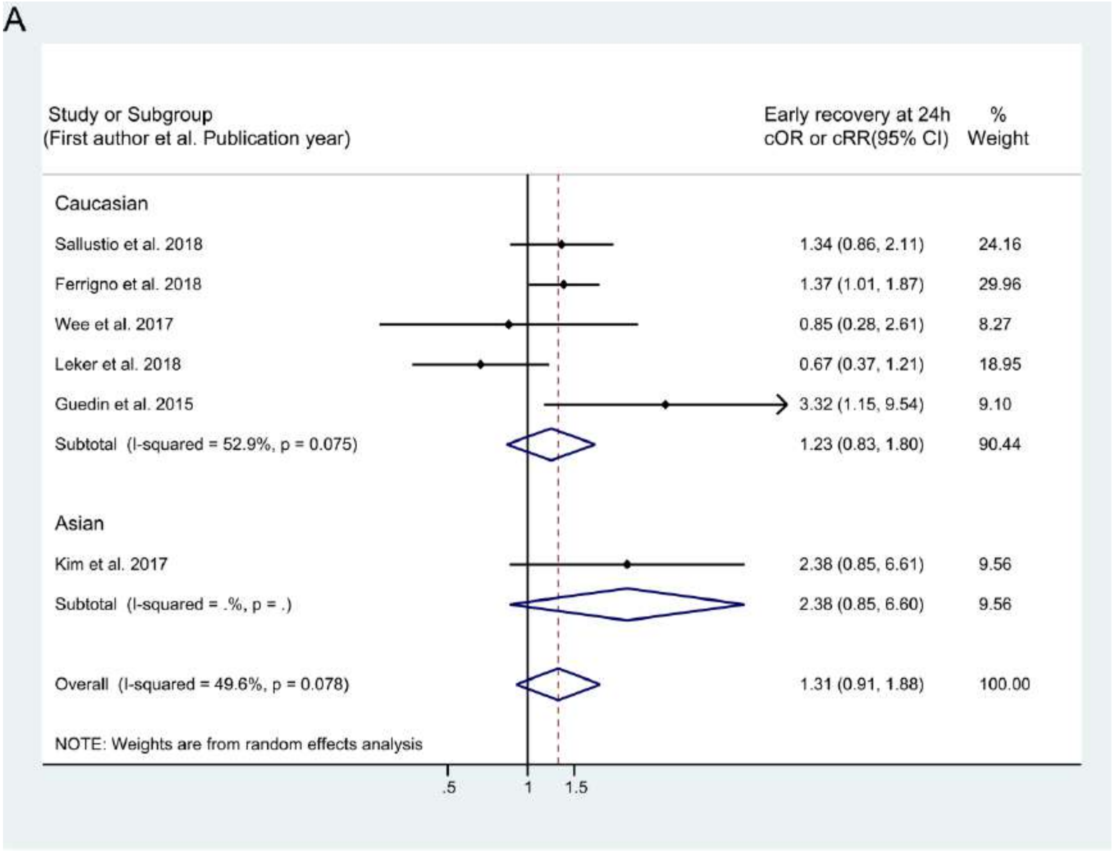


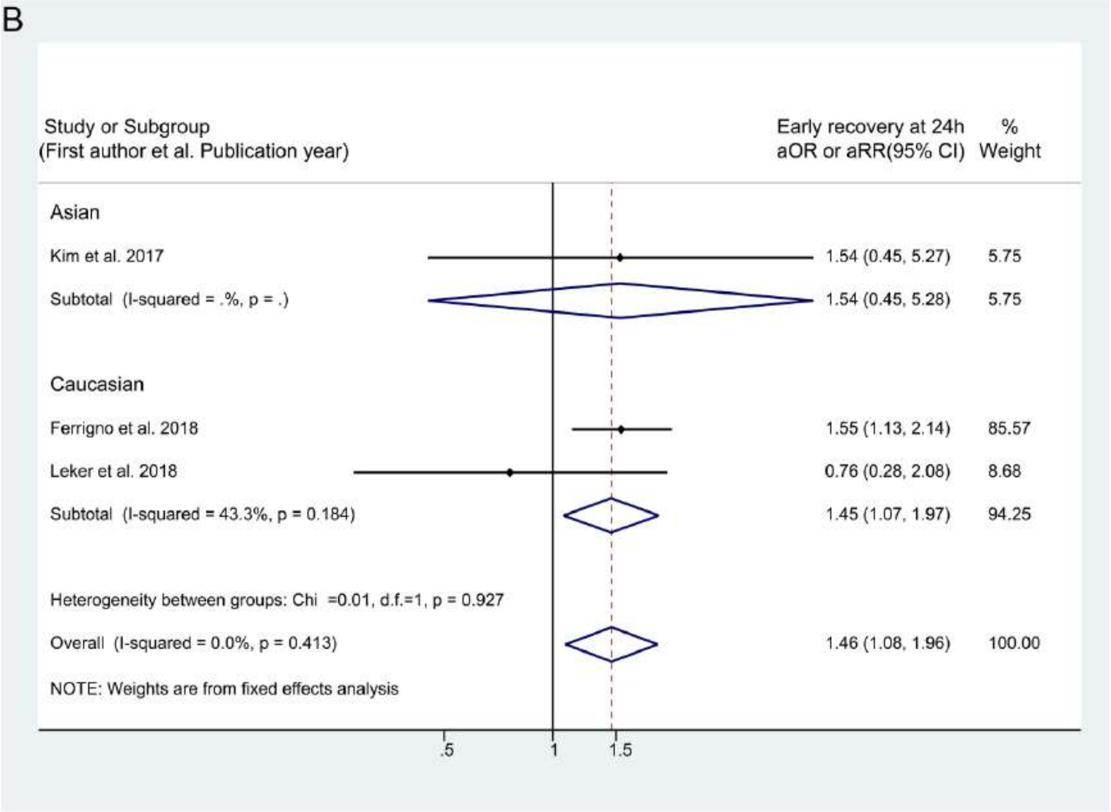

**Supplemental Figure 71.** Forest plots of subgroup analysis assessing early recovery at 24h by study type in unadjusted (A) and adjusted analysis (B).

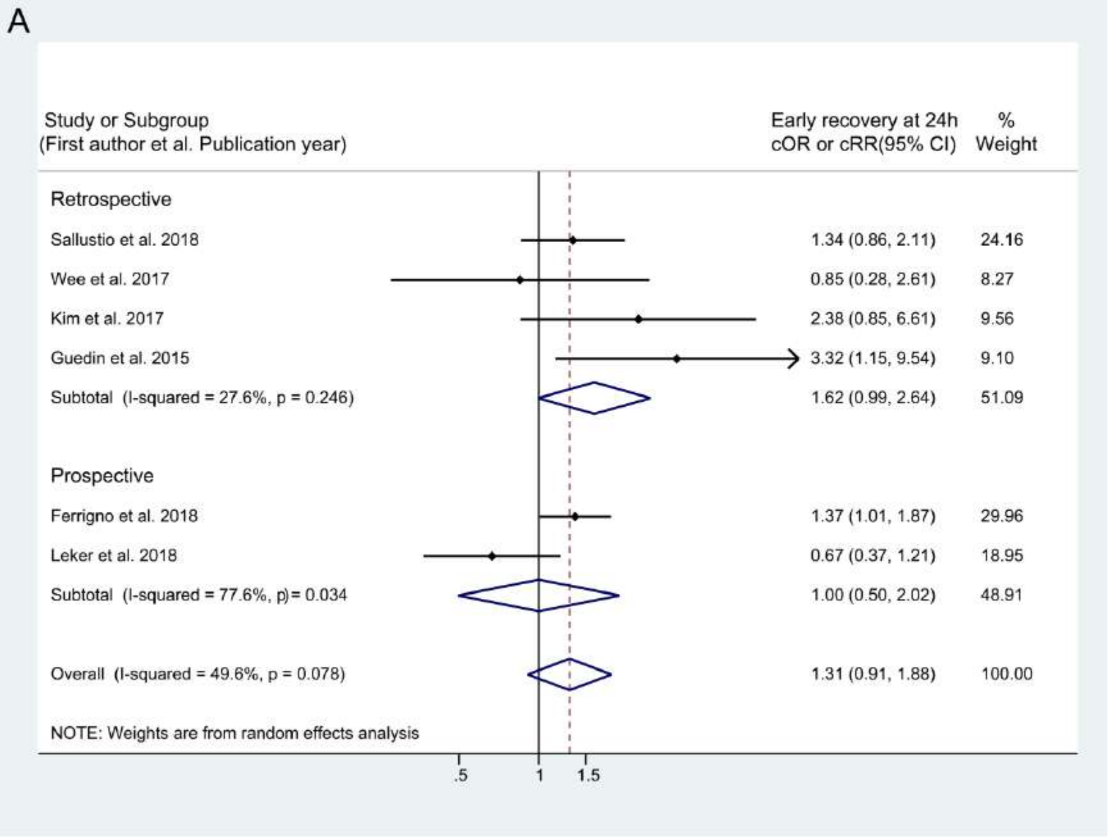


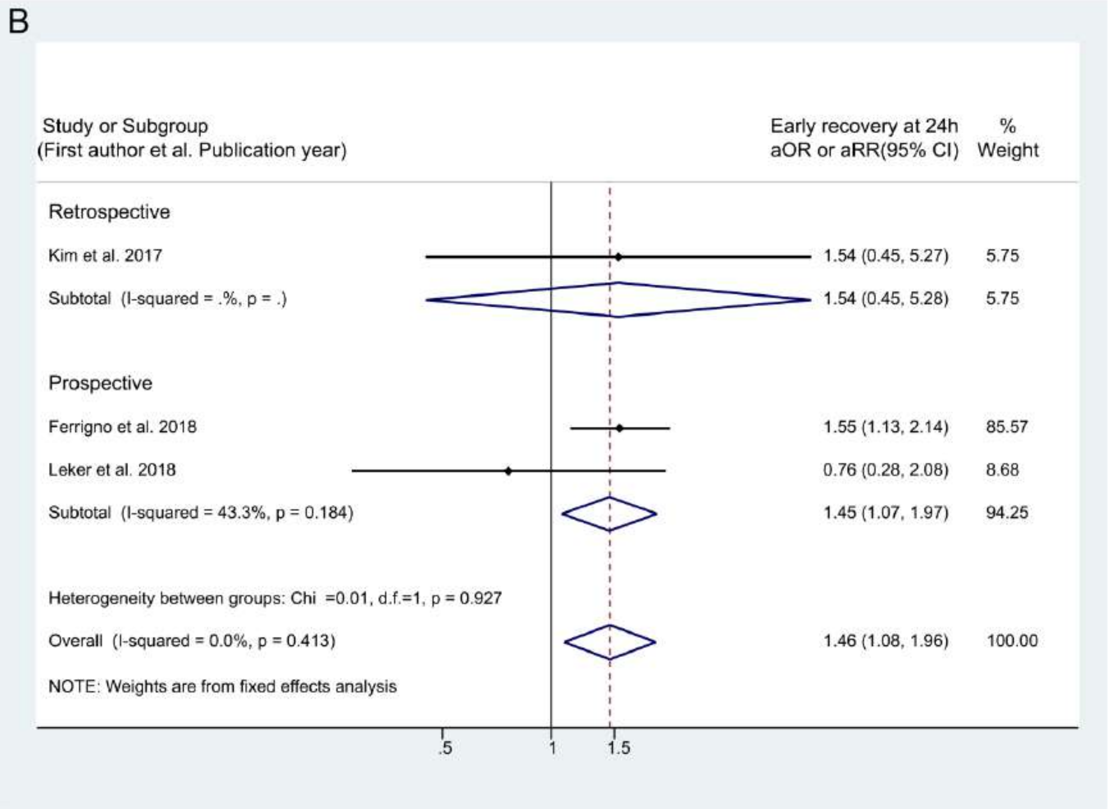

**Supplemental Figure 72.** Forest plots of subgroup analysis assessing good improvement at discharge/7 days by location of occluded artery (A) and study type (B) in unadjusted analysis.

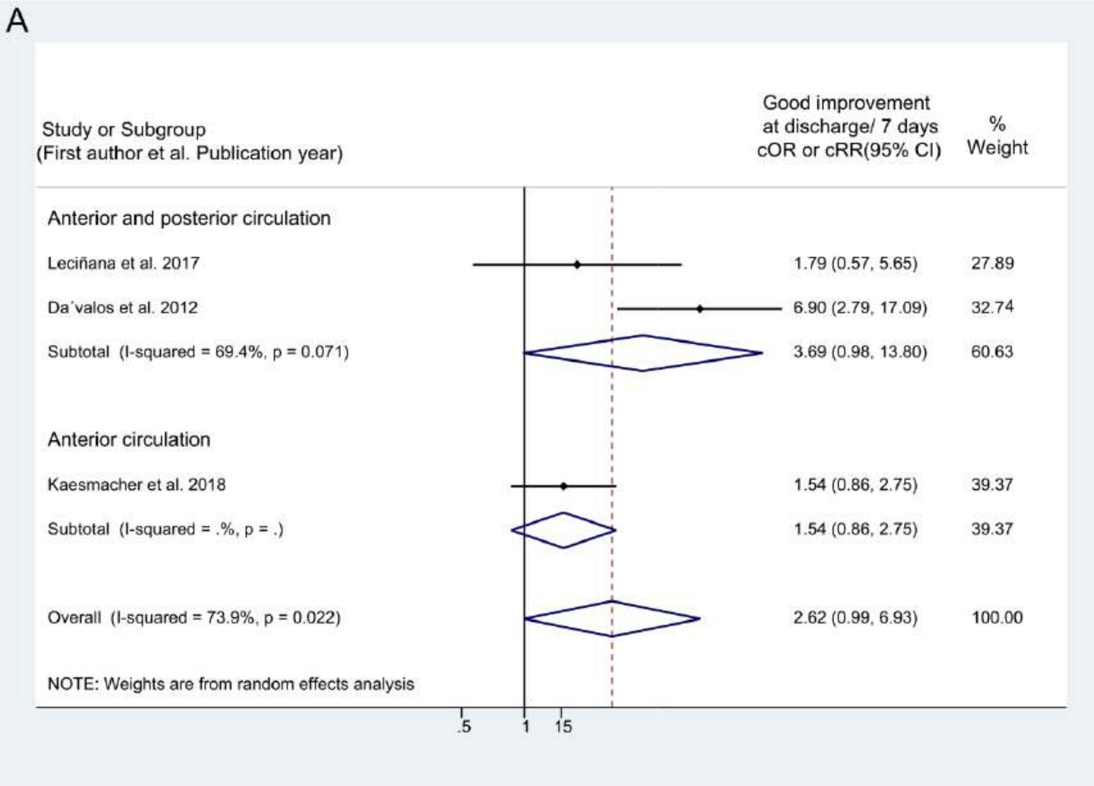


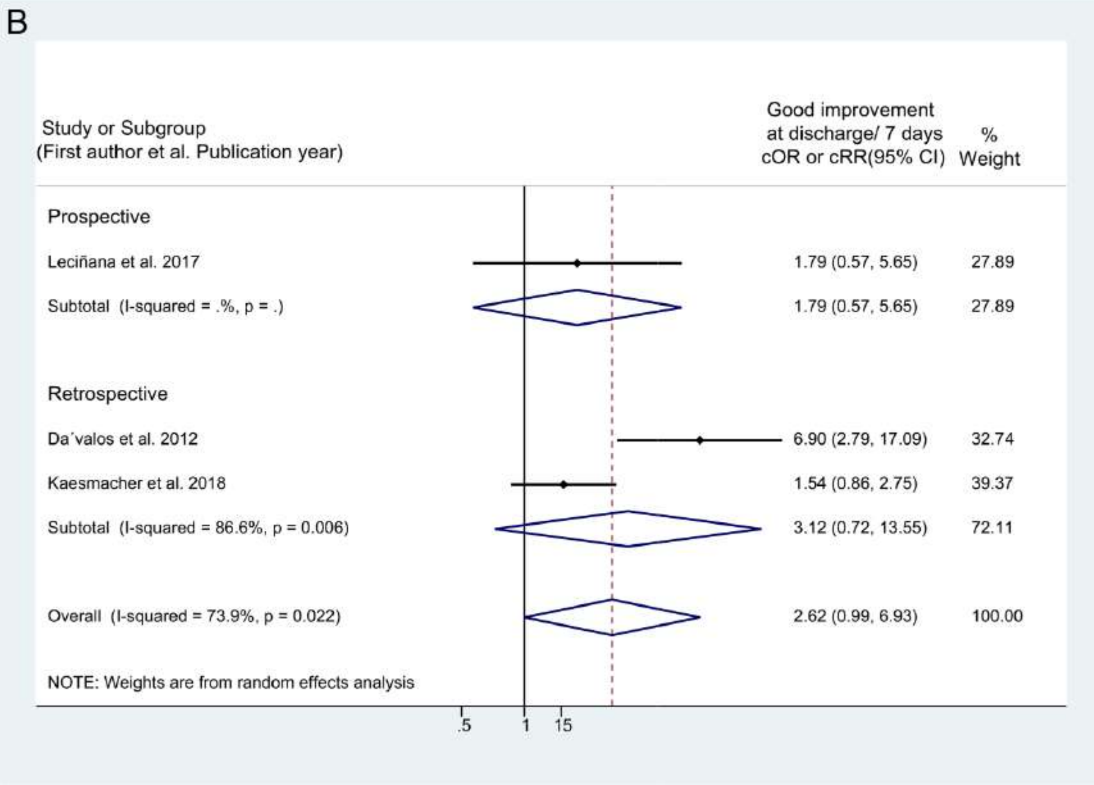

**Supplemental Figure 73.** Forest plots of studies assessing passes number of the thrombectomy device=1 (A) in unadjusted analysis and subgroup analysis by study type (B).

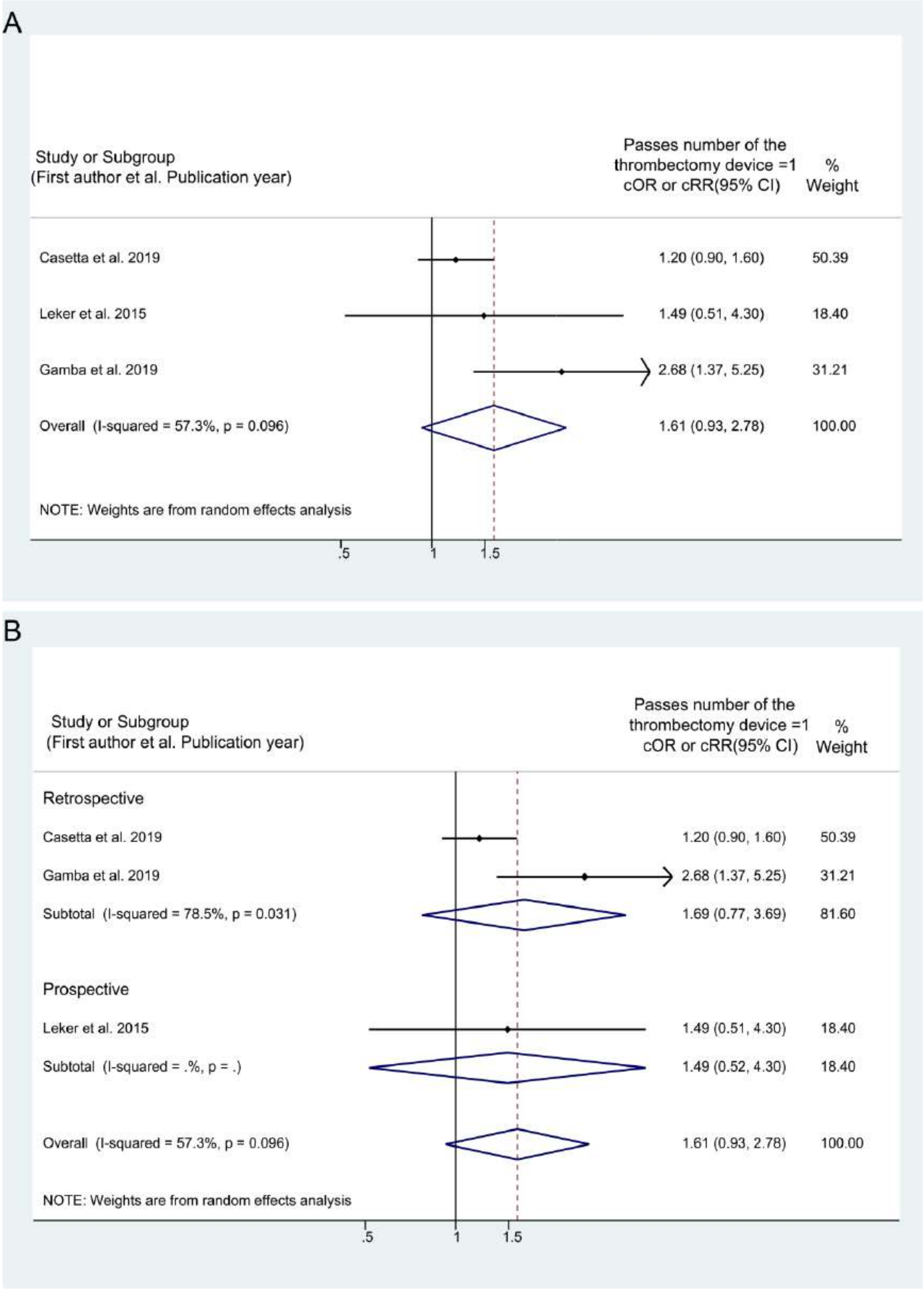

**Supplemental Figure 74.** Forest plots of subgroup analysis assessing passes number of the thrombectomy device⩽2 by location of occluded artery in unadjusted (A) and adjusted analysis (B).

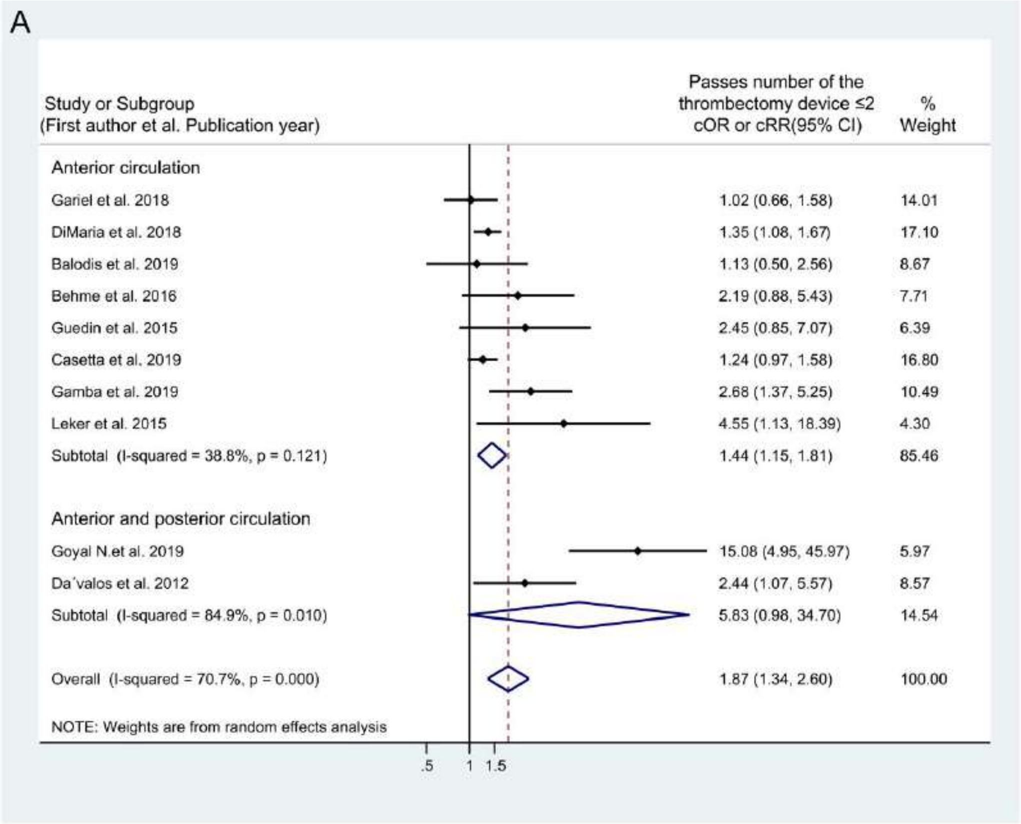


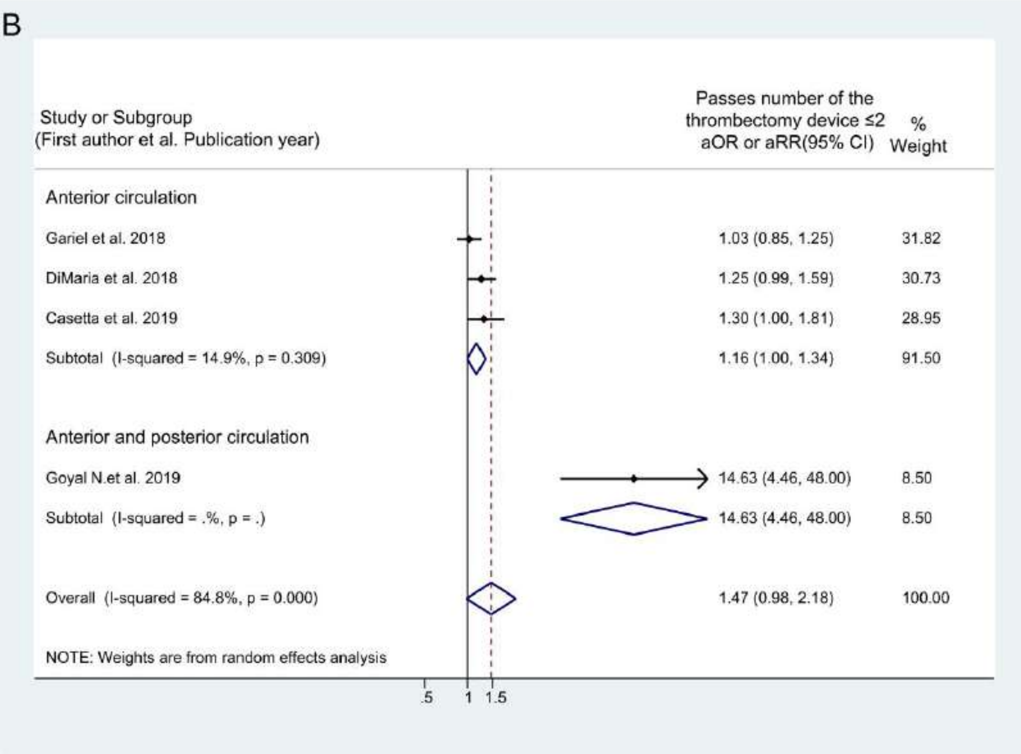

**Supplemental Figure 75.** Forest plots of subgroup analysis assessing passes number of the thrombectomy device≤2 by study type in unadjusted (A) and adjusted analysis (B).

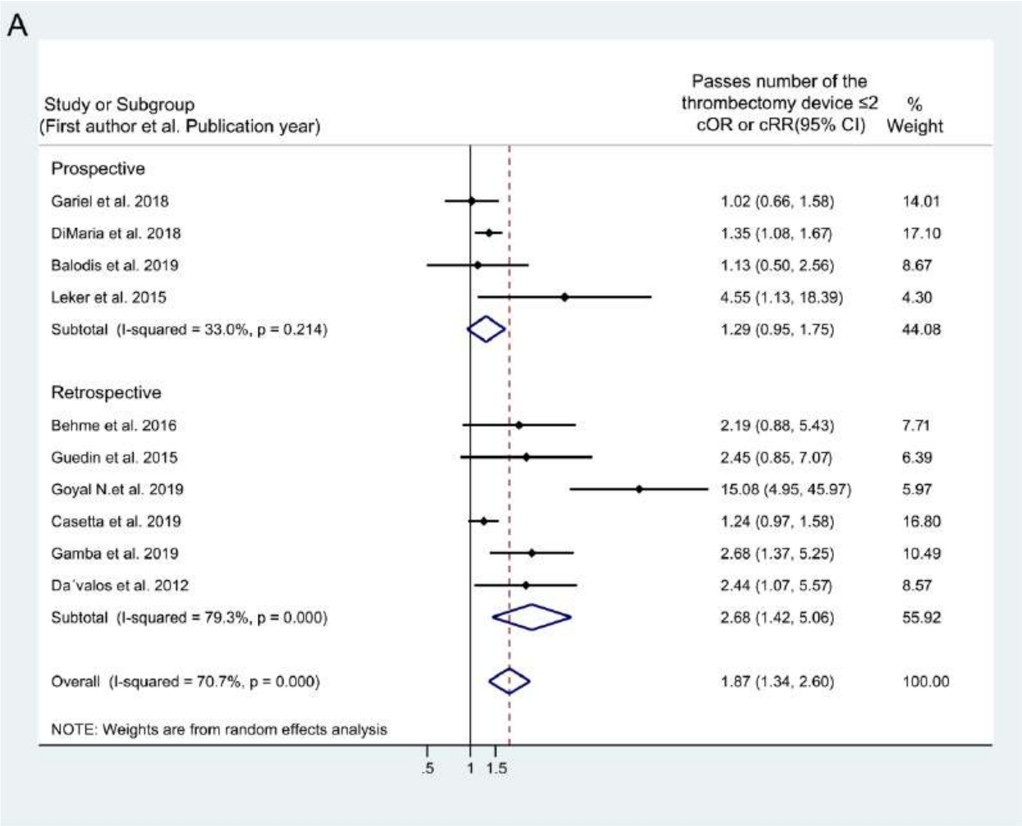


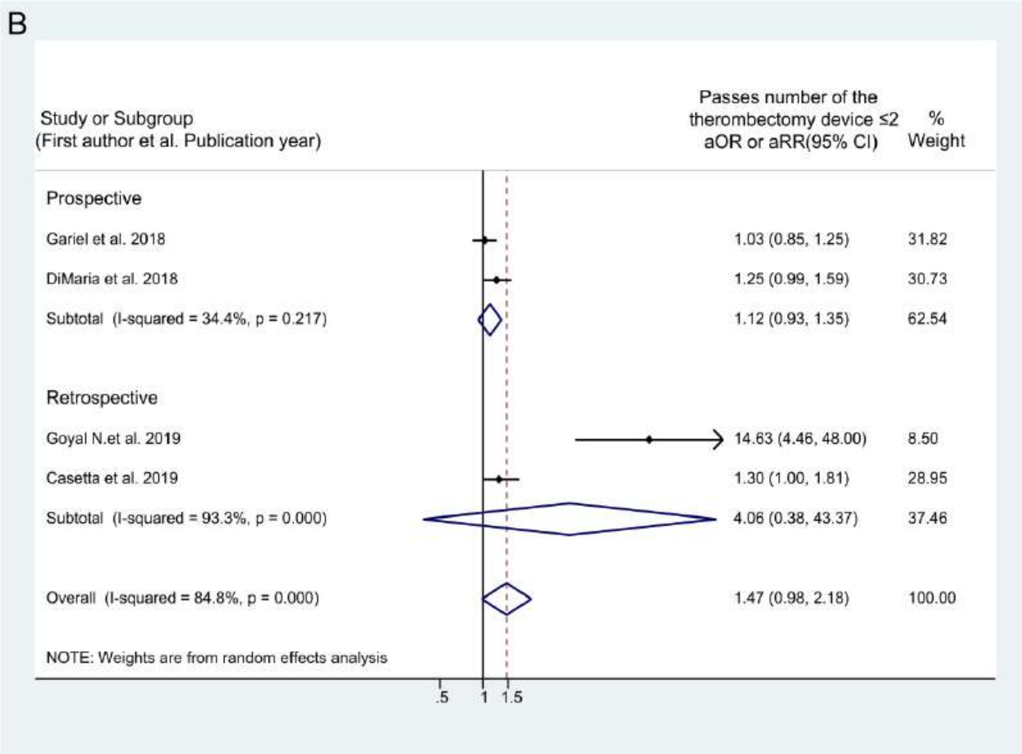

**Supplemental Figure 76.** Forest plots of studies assessing any complications in unadjusted (A) and adjusted analysis (B). [#]The outcomes of procedural complications (a), rescue therapy (b) and clot migration (c) were investigated respectively in the study of Gariel et al. 2018; The outcomes of vasospasm (d), vessel perforation (e), clot migration (c) and rescue therapy (b) were investigated respectively in the study of DiMaria et al. 2018; The outcomes of vessel perforation (e), arterial dissection (f), device failure (g) and clot migration (c) were investigated respectively in the study of Minnerup et al. 2016; The outcomes of vessel pseudoaneurysm (h), groin haematoma (i) and clot migration (c) were investigated respectively in the study of Wee et al. 2017; The outcomes of vessel perforation (e), vasospasm (d), clot migration (c) and groin haematoma (i) were investigated respectively in the study of Coutinho et al. 2017; The outcomes of groin haematoma (i) and clot migration (c) were investigated respectively in the study of Weber et al. 2017; The outcomes of arterial dissection (f) and clot migration (c) were investigated respectively in the study of Casetta et al. 2019;

A

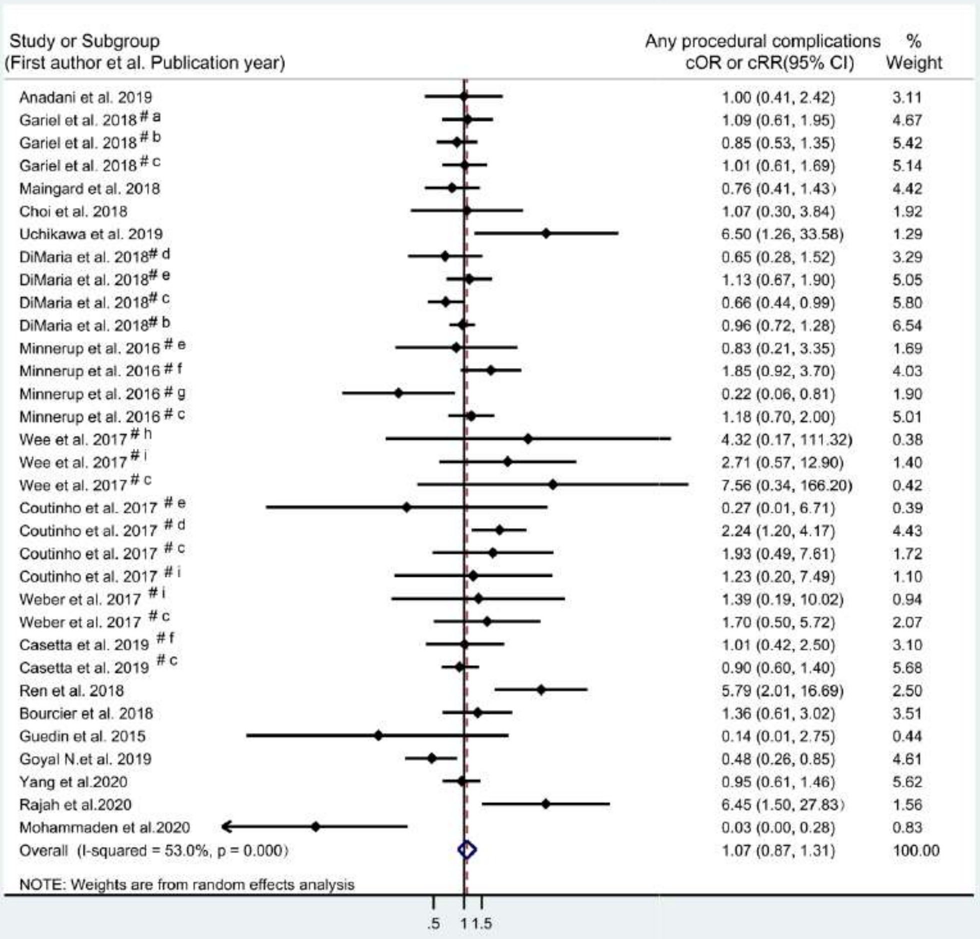

Study or Subgroup
(First author et al. Publication year)
Any procedural complications
cOR or cRR(95% CI)
%
Weight
Anadani et al. 2019 1.00 (0.41, 2.42) 3.11
Gariel et al. 2018 # a 1.09 (0.61, 1.95) 4.67
Gariel et al. 2018 # b 0.85 (0.53, 1.35) 5.42
Gariel et al. 2018 # c 1.01 (0.61, 1.69) 5.14
Maingard et al. 2018 0.76 (0.41, 1.43) 4.42
Choi et al. 2018 1.07 (0.30, 3.84) 1.92
Uchikawa et al. 2019 6.50 (1.26, 33.58) 1.29
DiMaria et al. 2018# d 0.65 (0.28, 1.52) 3.29
DiMaria et al. 2018# e 1.13 (0.67, 1.90) 5.05
DiMaria et al. 2018# c 0.66 (0.44, 0.99) 5.80
DiMaria et al. 2018# b 0.96 (0.72, 1.28) 6.54
Minnerup et al. 2016 # e 0.83 (0.21, 3.35) 1.69
Minnerup et al. 2016 # f 1.85 (0.92, 3.70) 4.03
Minnerup et al. 2016 # g 0.22 (0.06, 0.81) 1.90
Minnerup et al. 2016 # c 1.18 (0.70, 2.00) 5.01
Wee et al. 2017 # h 4.32 (0.17, 111.32) 0.38
Wee et al. 2017 # i 2.71 (0.57, 12.90) 1.40
Wee et al. 2017 # c 7.56 (0.34, 166.20) 0.42
Coutinho et al. 2017 # e 0.27 (0.01, 6.71) 0.39
Coutinho et al. 2017 # d 2.24 (1.20, 4.17) 4.43
Coutinho et al. 2017 # c 1.93 (0.49, 7.61) 1.72
Coutinho et al. 2017 # i 1.23 (0.20, 7.49) 1.10
Weber et al. 2017 # i 1.39 (0.19, 10.02) 0.94
Weber et al. 2017 # c 1.70 (0.50, 5.72) 2.07
Casetta et al. 2019 # f 1.01 (0.42, 2.50) 3.10
Casetta et al. 2019 # c 0.90 (0.60, 1.40) 5.68
Ren et al. 2018 5.79 (2.01, 16.69) 2.50
Bourcier et al. 2018 1.36 (0.61, 3.02) 3.51
Guedin et al. 2015 0.14 (0.01, 2.75) 0.44
Goyal N.et al. 2019 0.48 (0.26, 0.85) 4.61
Yang et al.2020 0.95 (0.61, 1.46) 5.62
Rajah et al.2020 6.45 (1.50, 27.83) 1.56
Mohammaden et al.2020 0.03 (0.00, 0.28) 0.83
Overall (I-squared = 53.0%, p = 0.000) 1.07 (0.87, 1.31) 100.00
NOTE: Weights are from random effects analysis
.5
1
1.5


B

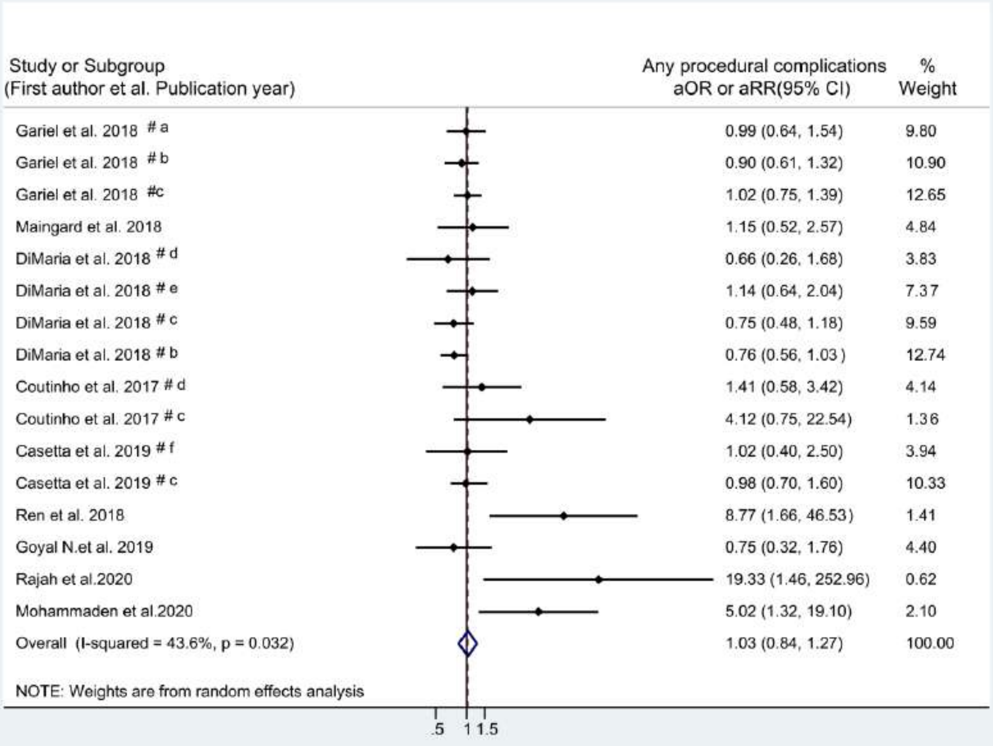

Study or Subgroup
(First author et al. Publication year)
Any procedural complications
aOR or aRR(95% CI)
%
Weight
Gariel et al. 2018 # a 0.99 (0.64, 1.54) 9.80
Gariel et al. 2018 # b 0.90 (0.61, 1.32) 10.90
Gariel et al. 2018 #c 1.02 (0.75, 1.39) 12.65
Maingard et al. 2018 1.15 (0.52, 2.57) 4.84
DiMaria et al. 2018 # d 0.66 (0.26, 1.68) 3.83
DiMaria et al. 2018 # e 1.14 (0.64, 2.04) 7.37
DiMaria et al. 2018 # c 0.75 (0.48, 1.18) 9.59
DiMaria et al. 2018 # b 0.76 (0.56, 1.03 ) 12.74
Coutinho et al. 2017 # d 1.41 (0.58, 3.42) 4.14
Coutinho et al. 2017 # c 4.12 (0.75, 22.54) 1.36
Casetta et al. 2019 # f 1.02 (0.40, 2.50) 3.94
Casetta et al. 2019 # c 0.98 (0.70, 1.60) 10.33
Ren et al. 2018 8.77 (1.66, 46.53) 1.41
Goyal N.et al. 2019 0.75 (0.32, 1.76) 4.40
Rajah et al.2020 19.33 (1.46, 252.96) 0.62
Mohammaden et al.2020 5.02 (1.32, 19.10) 2.10
Overall (I-squared = 43.6%, p = 0.032) 1.03 (0.84, 1.27) 100.00
NOTE: Weights are from random effects analysis
.5
1
1.5

**Supplemental Figure 77.** Forest plots of subgroup analysis assessing any complications by location of occluded artery in unadjusted (A) and adjusted analysis (B). #The outcomes of procedural complications (a), rescue therapy (b) and clot migration (c) were investigated respectively in the study of Gariel et al. 2018; The outcomes of vasospasm (d), vessel perforation (e), clot migration (c) and rescue therapy (b) were investigated respectively in the study of DiMaria et al. 2018; The outcomes of vessel perforation (e), arterial dissection (f), device failure (g) and clot migration (c) were investigated respectively in the study of Minnerup et al. 2016; The outcomes of vessel pseudoaneurysm (h), groin haematoma (i) and clot migration (c) were investigated respectively in the study of Wee et al. 2017; The outcomes of vessel perforation (e), vasospasm (d), clot migration (c) and groin haematoma (i) were investigated respectively in the study of Coutinho et al. 2017; The outcomes of groin haematoma (i) and clot migration (c) were investigated respectively in the study of Weber et al. 2017; The outcomes of arterial dissection (f) and clot migration (c) were investigated respectively in the study of Casetta et al. 2019;

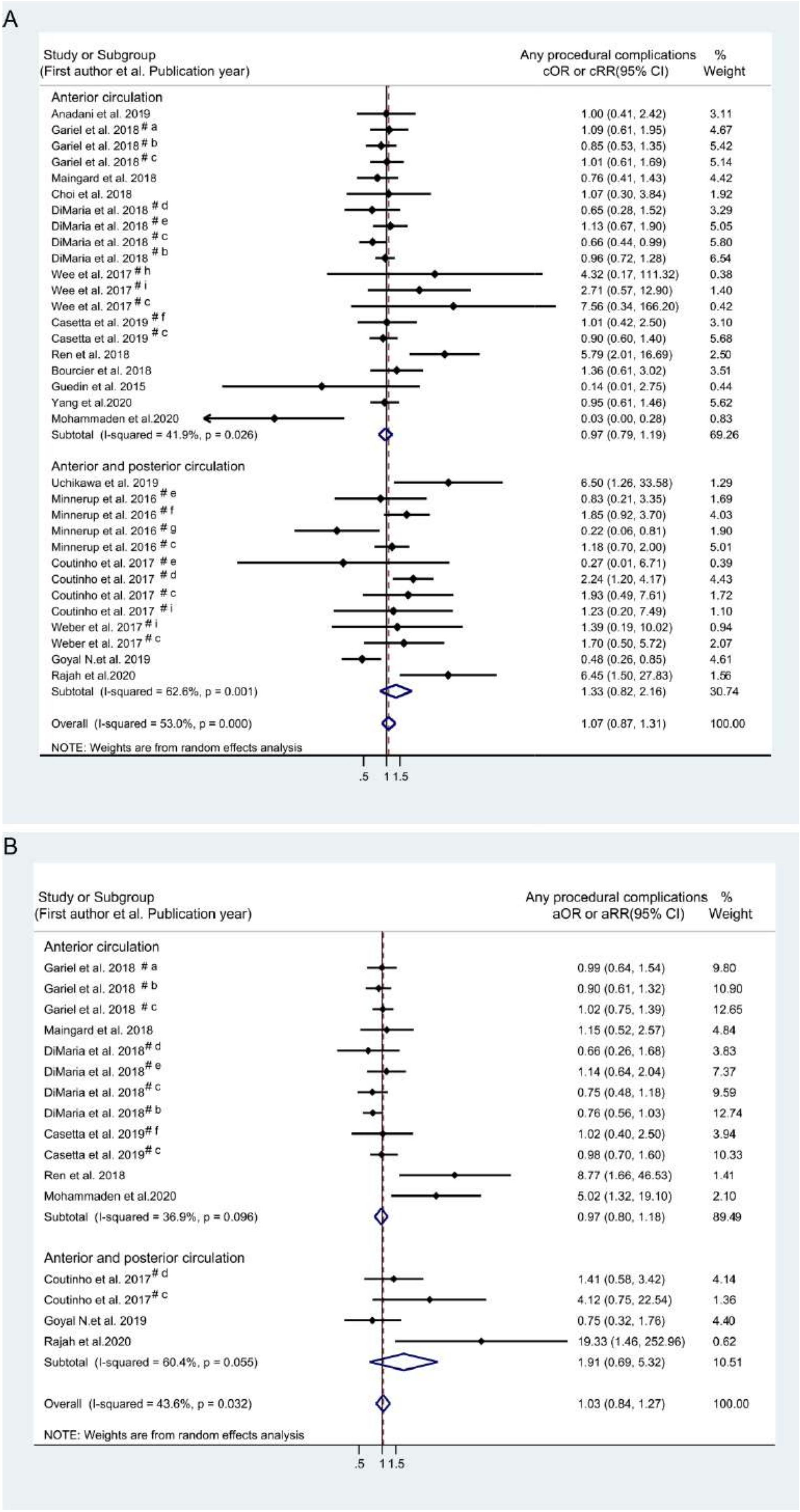
A
Study or Subgroup
(First author et al. Publication year)
Any procedural complications
cOR or cRR(95% CI)
% Weight
Anterior circulation
Anadani et al. 2019 1.00 (0.41, 2.42) 3.11
Gariel et al. 2018 # a 1.09 (0.61, 1.95) 4.67
Gariel et al. 2018 # b 0.85 (0.53, 1.35) 5.42
Gariel et al. 2018 # c 1.01 (0.61, 1.69) 5.14
Maingard et al. 2018 0.76 (0.41, 1.43) 4.42
Choi et al. 2018 1.07 (0.30, 3.84) 1.92
DiMaria et al. 2018 # d 0.65 (0.28, 1.52) 3.29
DiMaria et al. 2018 # e 1.13 (0.67, 1.90) 5.05
DiMaria et al. 2018 # c 0.66 (0.44, 0.99) 5.80
DiMaria et al. 2018 # b 0.96 (0.72, 1.28) 6.54
Wee et al. 2017 # h 4.32 (0.17, 111.32) 0.38
Wee et al. 2017 # i 2.71 (0.57, 12.90) 1.40
Wee et al. 2017 # c 7.56 (0.34, 166.20) 0.42
Casetta et al. 2019 # f 1.01 (0.42, 2.50) 3.10
Casetta et al. 2019 # c 0.90 (0.60, 1.40) 5.68
Ren et al. 2018 5.79 (2.01, 16.69) 2.50
Bourcier et al. 2018 1.36 (0.61, 3.02) 3.51
Guedin et al. 2015 0.14 (0.01, 2.75) 0.44
Yang et al.2020 0.95 (0.61, 1.46) 5.62
Mohammaden et al.2020 0.03 (0.00, 0.28) 0.83
Subtotal (I-squared = 41.9%, p = 0.026) 0.97 (0.79, 1.19) 69.26
Anterior and posterior circulation
Uchikawa et al. 2019 6.50 (1.26, 33.58) 1.29
Minnerup et al. 2016 # e 0.83 (0.21, 3.35) 1.69
Minnerup et al. 2016 # f 1.85 (0.92, 3.70) 4.03
Minnerup et al. 2016 # g 0.22 (0.06, 0.81) 1.90
Minnerup et al. 2016 # c 1.18 (0.70, 2.00) 5.01
Coutinho et al. 2017 # e 0.27 (0.01, 6.71) 0.39
Coutinho et al. 2017 # d 2.24 (1.20, 4.17) 4.43
Coutinho et al. 2017 # c 1.93 (0.49, 7.61) 1.72
Coutinho et al. 2017 # i 1.23 (0.20, 7.49) 1.10
Weber et al. 2017 # i 1.39 (0.19, 10.02) 0.94
Weber et al. 2017 # c 1.70 (0.50, 5.72) 2.07
Goyal N.et al. 2019 0.48 (0.26, 0.85) 4.61
Rajah et al.2020 6.45 (1.50, 27.83) 1.56
Subtotal (I-squared = 62.6%, p = 0.001) 1.33 (0.82, 2.16) 30.74
Overall (I-squared = 53.0%, p = 0.000) 1.07 (0.87, 1.31) 100.00
NOTE: Weights are from random effects analysis
.5 1 1.5
B
Study or Subgroup
(First author et al. Publication year)
Any procedural complications
aOR or aRR(95% CI)
% Weight
Anterior circulation
Gariel et al. 2018 # a 0.99 (0.64, 1.54) 9.80
Gariel et al. 2018 # b 0.90 (0.61, 1.32) 10.90
Gariel et al. 2018 # c 1.02 (0.75, 1.39) 12.65
Maingard et al. 2018 1.15 (0.52, 2.57) 4.84
DiMaria et al. 2018 # d 0.66 (0.26, 1.68) 3.83
DiMaria et al. 2018 # e 1.14 (0.64, 2.04) 7.37
DiMaria et al. 2018 # c 0.75 (0.48, 1.18) 9.59
DiMaria et al. 2018 # b 0.76 (0.56, 1.03) 12.74
Casetta et al. 2019 # f 1.02 (0.40, 2.50) 3.94
Casetta et al. 2019 # c 0.98 (0.70, 1.60) 10.33
Ren et al. 2018 8.77 (1.66, 46.53) 1.41
Mohammaden et al.2020 5.02 (1.32, 19.10) 2.10
Subtotal (I-squared = 36.9%, p = 0.096) 0.97 (0.80, 1.18) 89.49
Anterior and posterior circulation
Coutinho et al. 2017 # d 1.41 (0.58, 3.42) 4.14
Coutinho et al. 2017 # c 4.12 (0.75, 22.54) 1.36
Goyal N.et al. 2019 0.75 (0.32, 1.76) 4.40
Rajah et al.2020 19.33 (1.46, 252.96) 0.62
Subtotal (I-squared = 60.4%, p = 0.055) 1.91 (0.69, 5.32) 10.51
Overall (I-squared = 43.6%, p = 0.032) 1.03 (0.84, 1.27) 100.00
NOTE: Weights are from random effects analysis
.5 1 1.5

**Supplemental Figure 78.** Forest plots of subgroup analysis assessing any complications by study type in unadjusted (A) and adjusted analysis (B). [#]The outcomes of procedural complications (a), rescue therapy (b) and clot migration (c) were investigated respectively in the study of Gariel et al. 2018; The outcomes of vasospasm (d), vessel perforation (e), clot migration (c) and rescue therapy (b) were investigated respectively in the study of DiMaria et al. 2018; The outcomes of vessel perforation (e), arterial dissection (f), device failure (g) and clot migration (c) were investigated respectively in the study of Minnerup et al. 2016; The outcomes of vessel pseudoaneurysm (h), groin haematoma (i) and clot migration (c) were investigated respectively in the study of Wee et al. 2017; The outcomes of vessel perforation (e), vasospasm (d), clot migration (c) and groin haematoma (i) were investigated respectively in the study of Coutinho et al. 2017; The outcomes of groin haematoma (i) and clot migration (c) were investigated respectively in the study of Weber et al. 2017; The outcomes of arterial dissection (f) and clot migration (c) were investigated respectively in the study of Casetta et al. 2019;

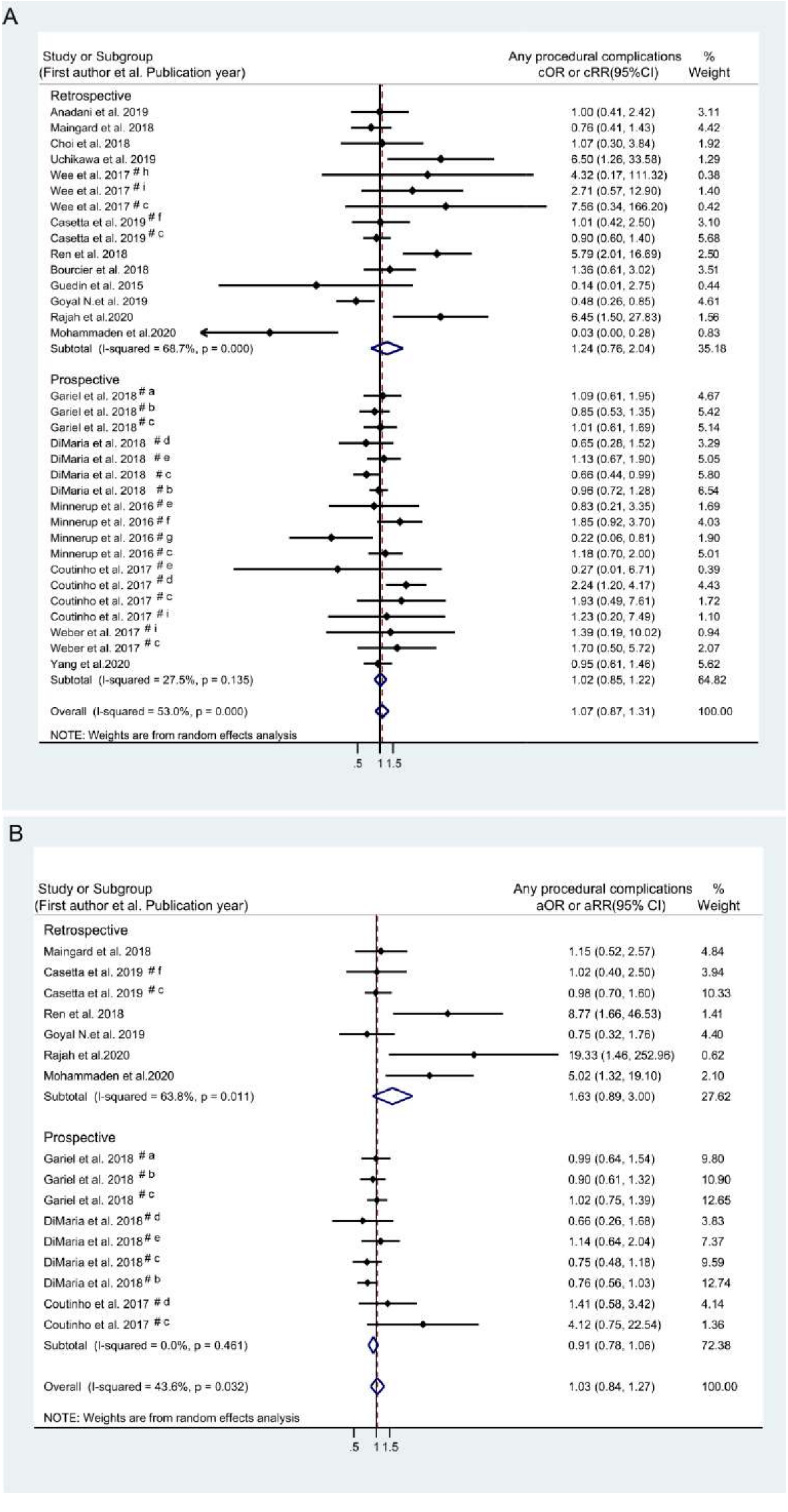
A
Study or Subgroup
(First author et al. Publication year)
Any procedural complications
cOR or cRR(95%CI)
%
Weight
Retrospective
Anadani et al. 2019 1.00 (0.41, 2.42) 3.11
Maingard et al. 2018 0.76 (0.41, 1.43) 4.42
Choi et al. 2018 1.07 (0.30, 3.84) 1.92
Uchikawa et al. 2019 6.50 (1.26, 33.58) 1.29
Wee et al. 2017 # h 4.32 (0.17, 111.32) 0.38
Wee et al. 2017 # i 2.71 (0.57, 12.90) 1.40
Wee et al. 2017 # c 7.56 (0.34, 166.20) 0.42
Casetta et al. 2019 # f 1.01 (0.42, 2.50) 3.10
Casetta et al. 2019 # c 0.90 (0.60, 1.40) 5.68
Ren et al. 2018 5.79 (2.01, 16.69) 2.50
Bourcier et al. 2018 1.36 (0.61, 3.02) 3.51
Guedin et al. 2015 0.14 (0.01, 2.75) 0.44
Goyal N.et al. 2019 0.48 (0.26, 0.85) 4.61
Rajah et al.2020 6.45 (1.50, 27.83) 1.56
Mohammaden et al.2020 0.03 (0.00, 0.28) 0.83
Subtotal (I-squared = 68.7%, p = 0.000) 1.24 (0.76, 2.04) 35.18
Prospective
Gariel et al. 2018 # a 1.09 (0.61, 1.95) 4.67
Gariel et al. 2018 # b 0.85 (0.53, 1.35) 5.42
Gariel et al. 2018 # c 1.01 (0.61, 1.69) 5.14
DiMaria et al. 2018 # d 0.65 (0.28, 1.52) 3.29
DiMaria et al. 2018 # e 1.13 (0.67, 1.90) 5.05
DiMaria et al. 2018 # c 0.66 (0.44, 0.99) 5.80
DiMaria et al. 2018 # b 0.96 (0.72, 1.28) 6.54
Minnerup et al. 2016 # e 0.83 (0.21, 3.35) 1.69
Minnerup et al. 2016 # f 1.85 (0.92, 3.70) 4.03
Minnerup et al. 2016 # g 0.22 (0.06, 0.81) 1.90
Minnerup et al. 2016 # c 1.18 (0.70, 2.00) 5.01
Coutinho et al. 2017 # e 0.27 (0.01, 6.71) 0.39
Coutinho et al. 2017 # d 2.24 (1.20, 4.17) 4.43
Coutinho et al. 2017 # c 1.93 (0.49, 7.61) 1.72
Coutinho et al. 2017 # i 1.23 (0.20, 7.49) 1.10
Weber et al. 2017 # i 1.39 (0.19, 10.02) 0.94
Weber et al. 2017 # c 1.70 (0.50, 5.72) 2.07
Yang et al.2020 0.95 (0.61, 1.46) 5.62
Subtotal (I-squared = 27.5%, p = 0.135) 1.02 (0.85, 1.22) 64.82
Overall (I-squared = 53.0%, p = 0.000) 1.07 (0.87, 1.31) 100.00
NOTE: Weights are from random effects analysis
.5 1 1.5
B
Study or Subgroup
(First author et al. Publication year)
Any procedural complications
aOR or aRR(95% CI)
%
Weight
Retrospective
Maingard et al. 2018 1.15 (0.52, 2.57) 4.84
Casetta et al. 2019 # f 1.02 (0.40, 2.50) 3.94
Casetta et al. 2019 # c 0.98 (0.70, 1.60) 10.33
Ren et al. 2018 8.77 (1.66, 46.53) 1.41
Goyal N.et al. 2019 0.75 (0.32, 1.76) 4.40
Rajah et al.2020 19.33 (1.46, 252.96) 0.62
Mohammaden et al.2020 5.02 (1.32, 19.10) 2.10
Subtotal (I-squared = 63.8%, p = 0.011) 1.63 (0.89, 3.00) 27.62
Prospective
Gariel et al. 2018 # a 0.99 (0.64, 1.54) 9.80
Gariel et al. 2018 # b 0.90 (0.61, 1.32) 10.90
Gariel et al. 2018 # c 1.02 (0.75, 1.39) 12.65
DiMaria et al. 2018 # d 0.66 (0.26, 1.68) 3.83
DiMaria et al. 2018 # e 1.14 (0.64, 2.04) 7.37
DiMaria et al. 2018 # c 0.75 (0.48, 1.18) 9.59
DiMaria et al. 2018 # b 0.76 (0.56, 1.03) 12.74
Coutinho et al. 2017 # d 1.41 (0.58, 3.42) 4.14
Coutinho et al. 2017 # c 4.12 (0.75, 22.54) 1.36
Subtotal (I-squared = 0.0%, p = 0.461) 0.91 (0.78, 1.06) 72.38
Overall (I-squared = 43.6%, p = 0.032) 1.03 (0.84, 1.27) 100.00
NOTE: Weights are from random effects analysis
.5 1 1.5

**Supplemental Figure 79.** Forest plots of subgroup analysis assessing any complications by ethnicity in unadjusted analysis. #The outcomes of procedural complications (a), rescue therapy (b) and clot migration (c) were investigated respectively in the study of Gariel et al. 2018; The outcomes of vasospasm (d), vessel perforation (e), clot migration (c) and rescue therapy (b) were investigated respectively in the study of DiMaria et al. 2018; The outcomes of vessel perforation (e), arterial dissection (f), device failure (g) and clot migration (c) were investigated respectively in the study of Minnerup et al. 2016; The outcomes of vessel pseudoaneurysm (h), groin haematoma (i) and clot migration (c) were investigated respectively in the study of Wee et al. 2017; The outcomes of vessel perforation (e), vasospasm (d), clot migration (c) and groin haematoma (i) were investigated respectively in the study of Coutinho et al. 2017; The outcomes of groin haematoma (i) and clot migration (c) were investigated respectively in the study of Weber et al. 2017; The outcomes of arterial dissection (f) and clot migration (c) were investigated respectively in the study of Casetta et al. 2019;

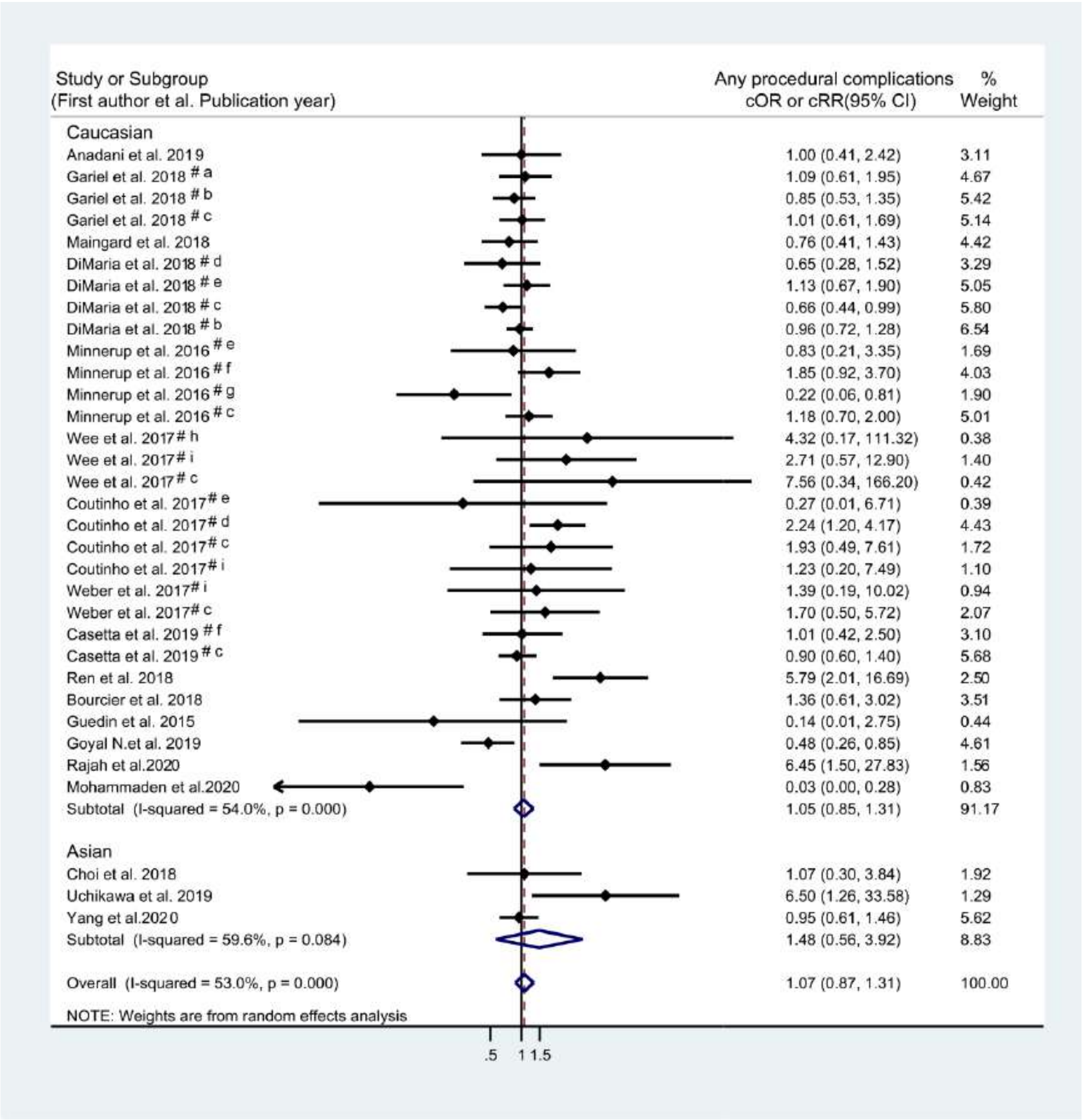
Study or Subgroup
(First author et al. Publication year)
Any procedural complications
cOR or cRR(95% CI)
%
Weight
Caucasian
Anadani et al. 2019 | 1.00 (0.41, 2.42) | 3.11
Gariel et al. 2018 # a | 1.09 (0.61, 1.95) | 4.67
Gariel et al. 2018 # b | 0.85 (0.53, 1.35) | 5.42
Gariel et al. 2018 # c | 1.01 (0.61, 1.69) | 5.14
Maingard et al. 2018 | 0.76 (0.41, 1.43) | 4.42
DiMaria et al. 2018 # d | 0.65 (0.28, 1.52) | 3.29
DiMaria et al. 2018 # e | 1.13 (0.67, 1.90) | 5.05
DiMaria et al. 2018 # c | 0.66 (0.44, 0.99) | 5.80
DiMaria et al. 2018 # b | 0.96 (0.72, 1.28) | 6.54
Minnerup et al. 2016 # e | 0.83 (0.21, 3.35) | 1.69
Minnerup et al. 2016 # f | 1.85 (0.92, 3.70) | 4.03
Minnerup et al. 2016 # g | 0.22 (0.06, 0.81) | 1.90
Minnerup et al. 2016 # c | 1.18 (0.70, 2.00) | 5.01
Wee et al. 2017 # h | 4.32 (0.17, 111.32) | 0.38
Wee et al. 2017 # i | 2.71 (0.57, 12.90) | 1.40
Wee et al. 2017 # c | 7.56 (0.34, 166.20) | 0.42
Coutinho et al. 2017 # e | 0.27 (0.01, 6.71) | 0.39
Coutinho et al. 2017 # d | 2.24 (1.20, 4.17) | 4.43
Coutinho et al. 2017 # c | 1.93 (0.49, 7.61) | 1.72
Coutinho et al. 2017 # i | 1.23 (0.20, 7.49) | 1.10
Weber et al. 2017 # i | 1.39 (0.19, 10.02) | 0.94
Weber et al. 2017 # c | 1.70 (0.50, 5.72) | 2.07
Casetta et al. 2019 # f | 1.01 (0.42, 2.50) | 3.10
Casetta et al. 2019 # c | 0.90 (0.60, 1.40) | 5.68
Ren et al. 2018 | 5.79 (2.01, 16.69) | 2.50
Bourcier et al. 2018 | 1.36 (0.61, 3.02) | 3.51
Guedin et al. 2015 | 0.14 (0.01, 2.75) | 0.44
Goyal N.et al. 2019 | 0.48 (0.26, 0.85) | 4.61
Rajah et al.2020 | 6.45 (1.50, 27.83) | 1.56
Mohammaden et al.2020 | 0.03 (0.00, 0.28) | 0.83
Subtotal (I-squared = 54.0%, p = 0.000) | 1.05 (0.85, 1.31) | 91.17
Asian
Choi et al. 2018 | 1.07 (0.30, 3.84) | 1.92
Uchikawa et al. 2019 | 6.50 (1.26, 33.58) | 1.29
Yang et al.2020 | 0.95 (0.61, 1.46) | 5.62
Subtotal (I-squared = 59.6%, p = 0.084) | 1.48 (0.56, 3.92) | 8.83
Overall (I-squared = 53.0%, p = 0.000) | 1.07 (0.87, 1.31) | 100.00
NOTE: Weights are from random effects analysis
.5
1
1.5

**Supplemental Figure 80.** Forest plots of studies assessing clot migration in unadjusted (A) and adjusted analysis (B).

A

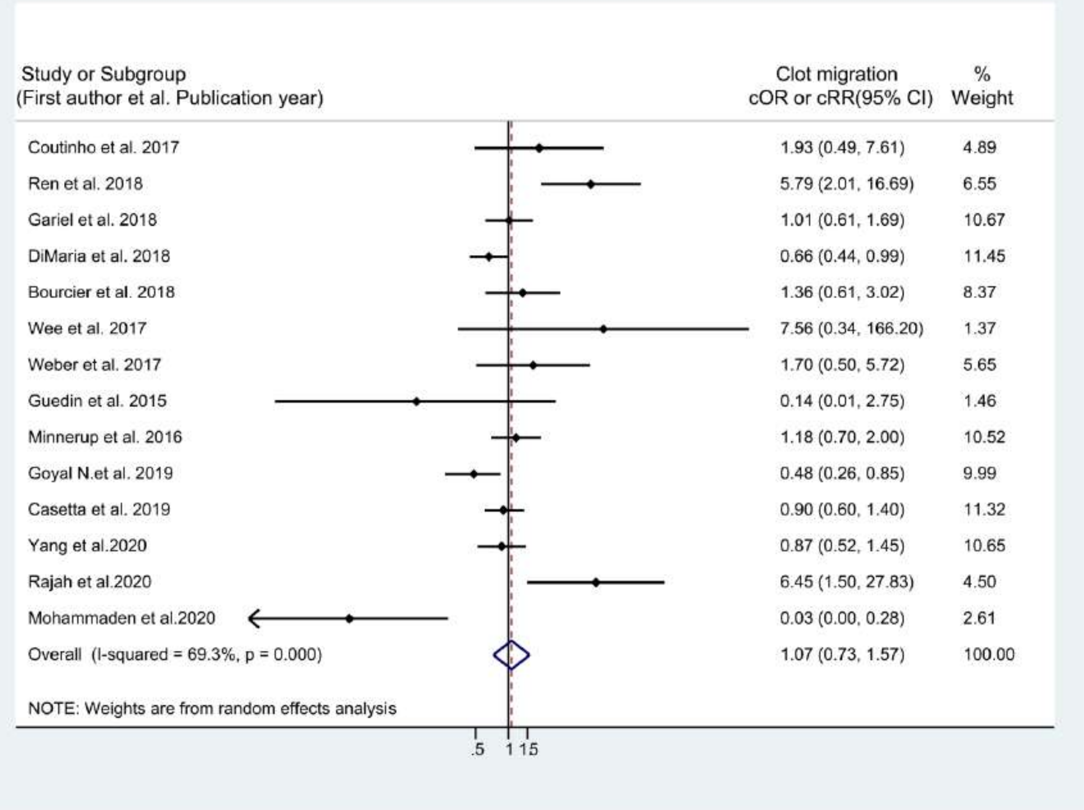


B

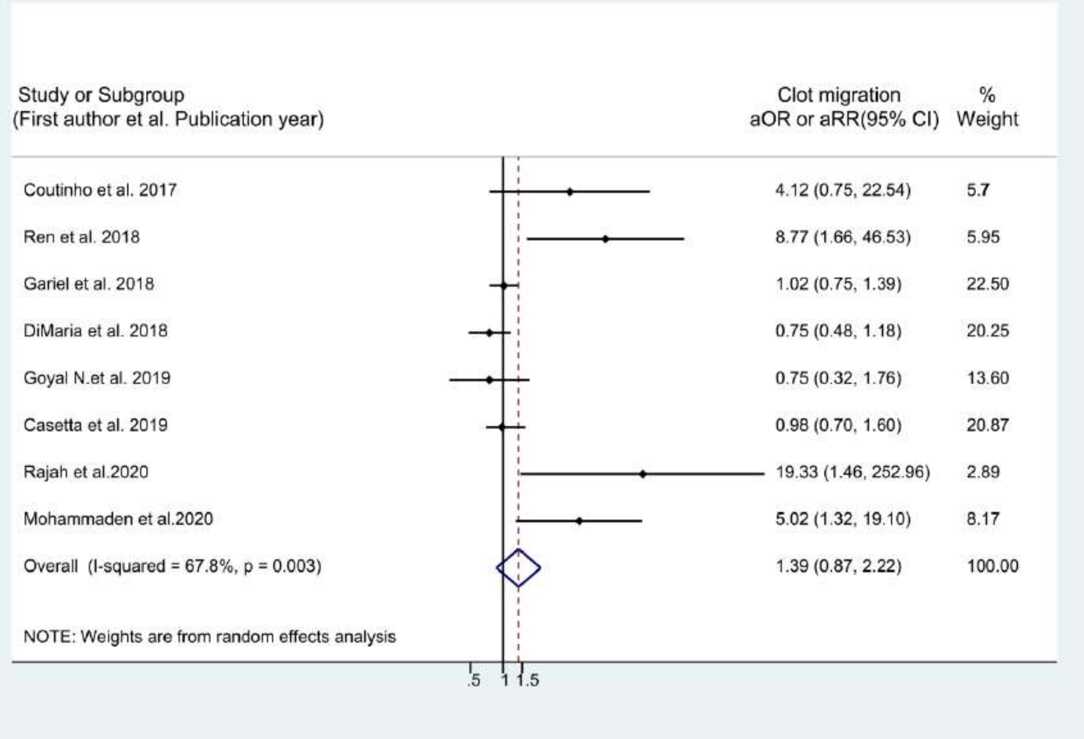

**Supplemental Figure 81.** Forest plots of subgroup analysis assessing clot migration by location of occluded artery in unadjusted (A) and adjusted analysis (B).

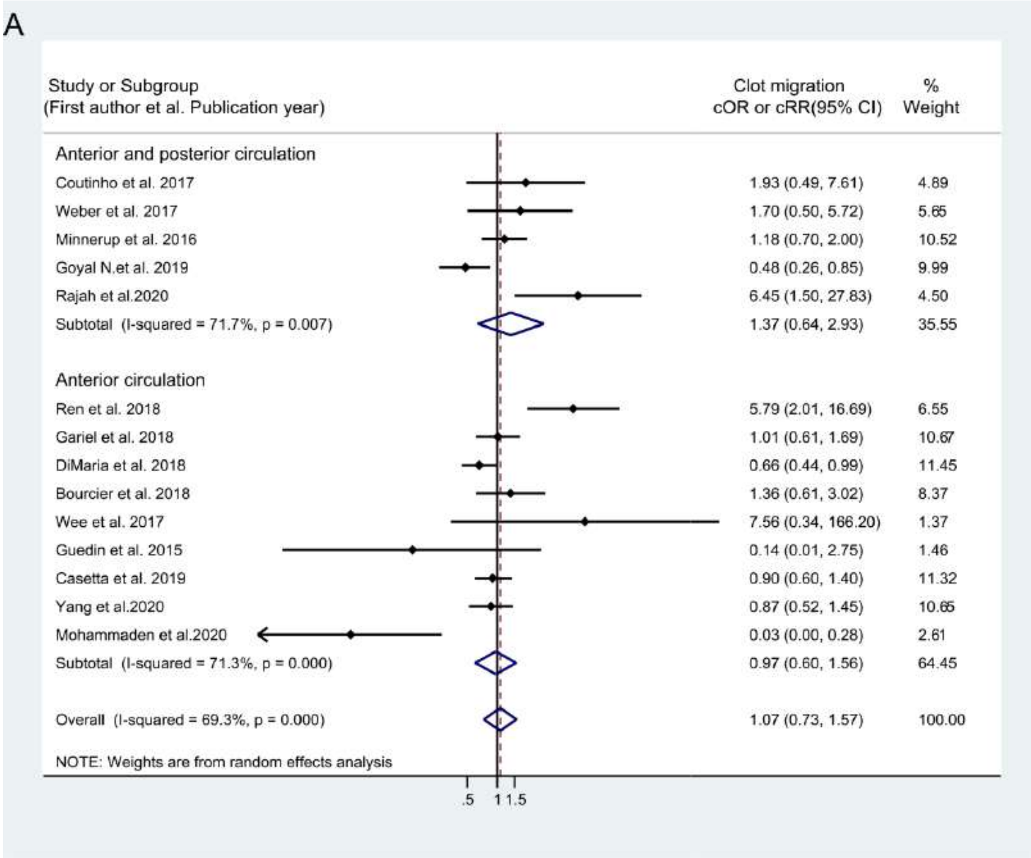


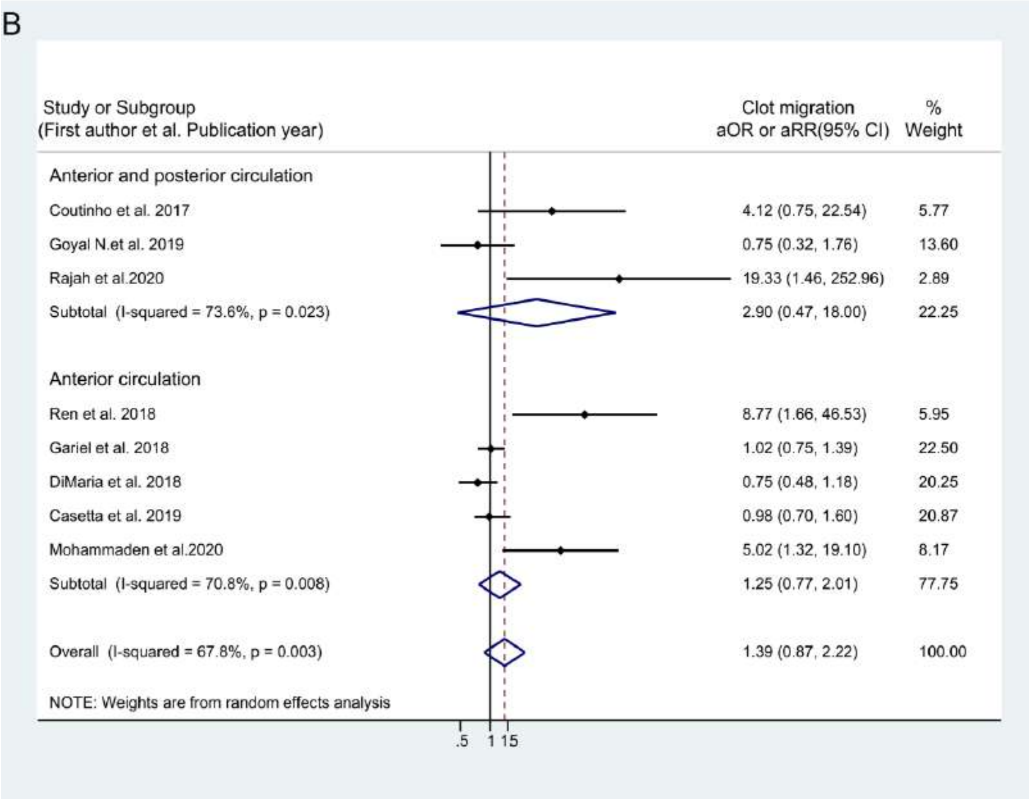

**Supplemental Figure 82.** Forest plots of subgroup analysis assessing clot migration by study type in unadjusted (A) and adjusted analysis (B).

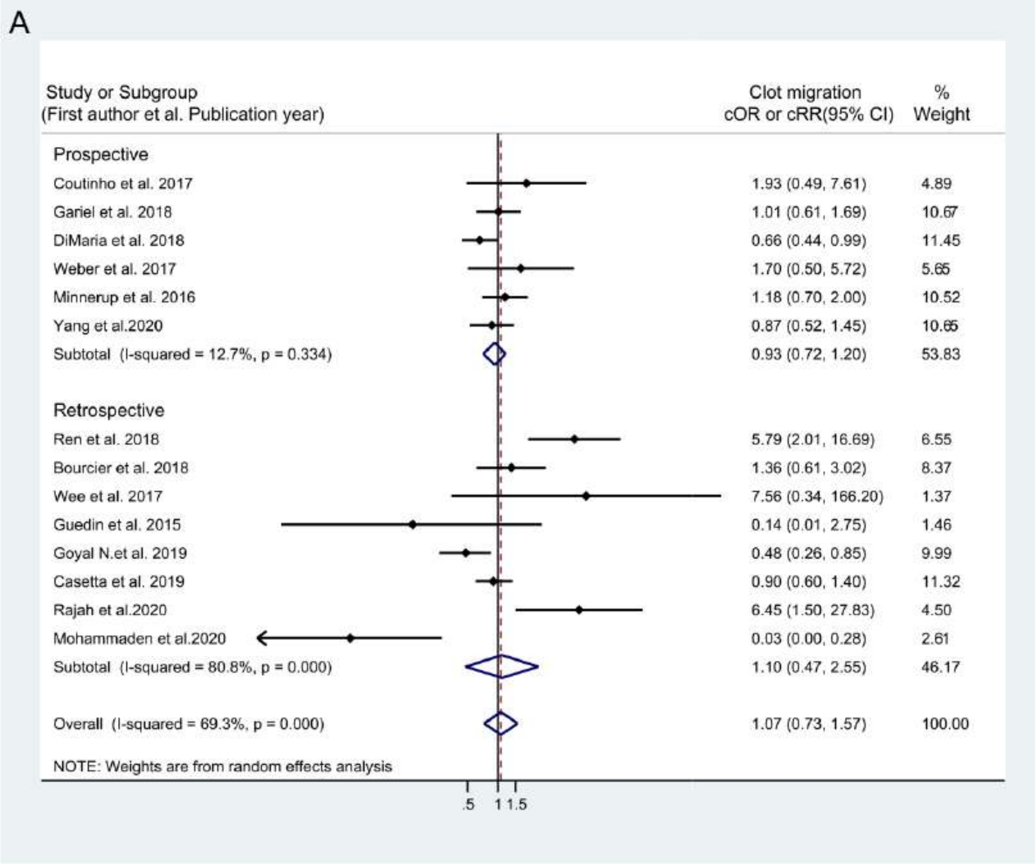


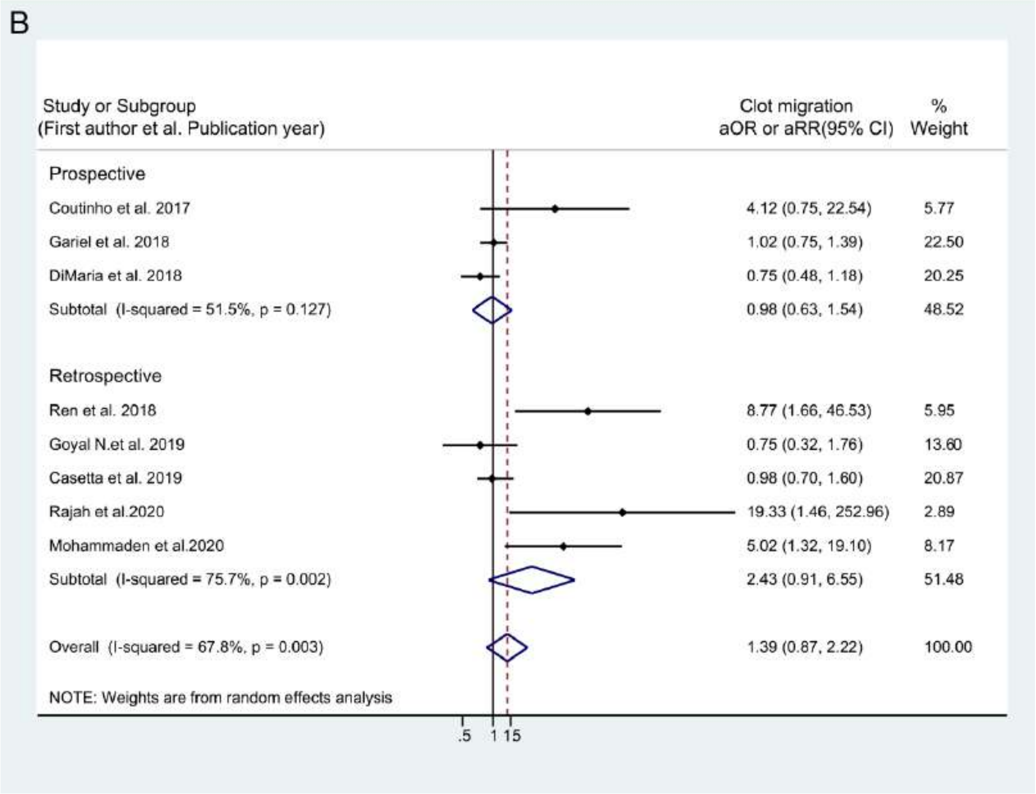

**Supplemental Figure 83.** Forest plots of subgroup analysis assessing clot migration by ethnicity in unadjusted (A) and adjusted analysis (B).

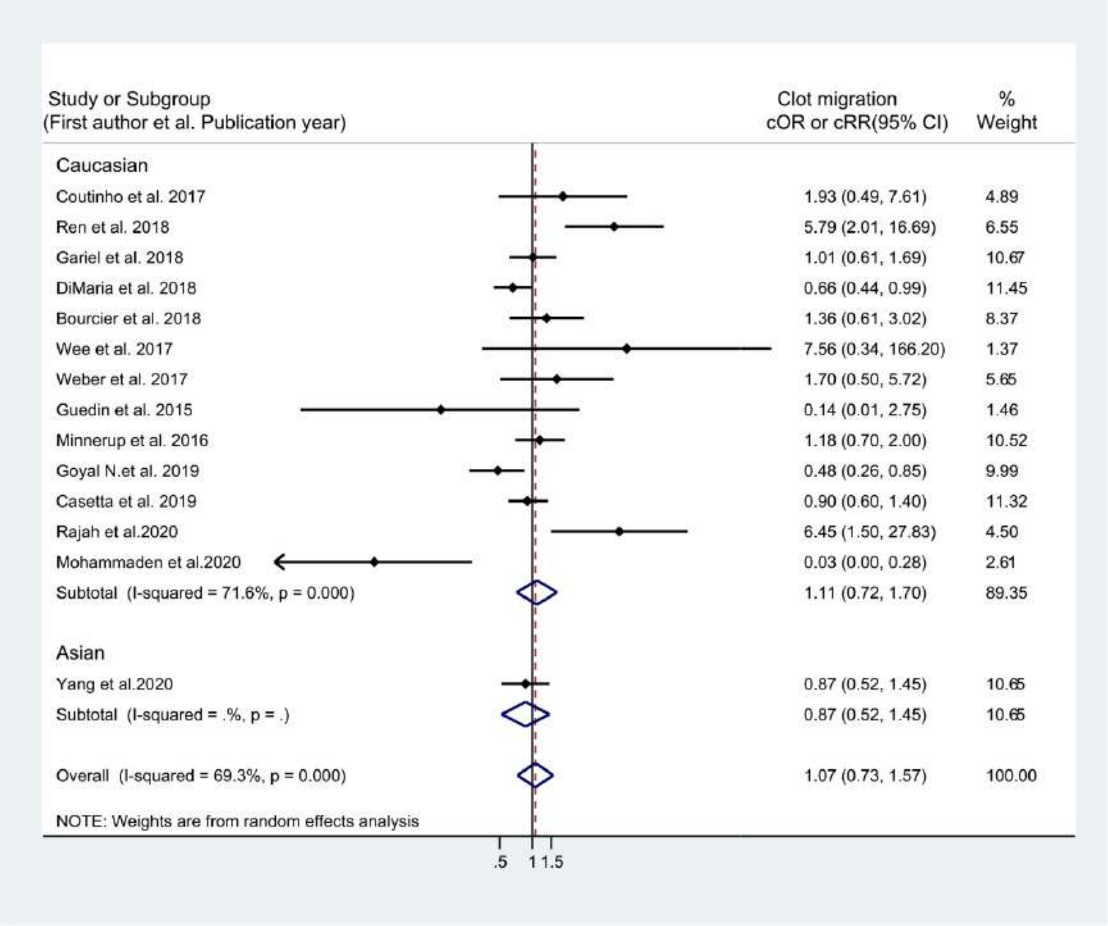

**Supplemental Figure 84.** Forest plots of studies assessing groin hematoma (A) and subgroup analysis by location of occluded artery (B) in unadjusted analysis.

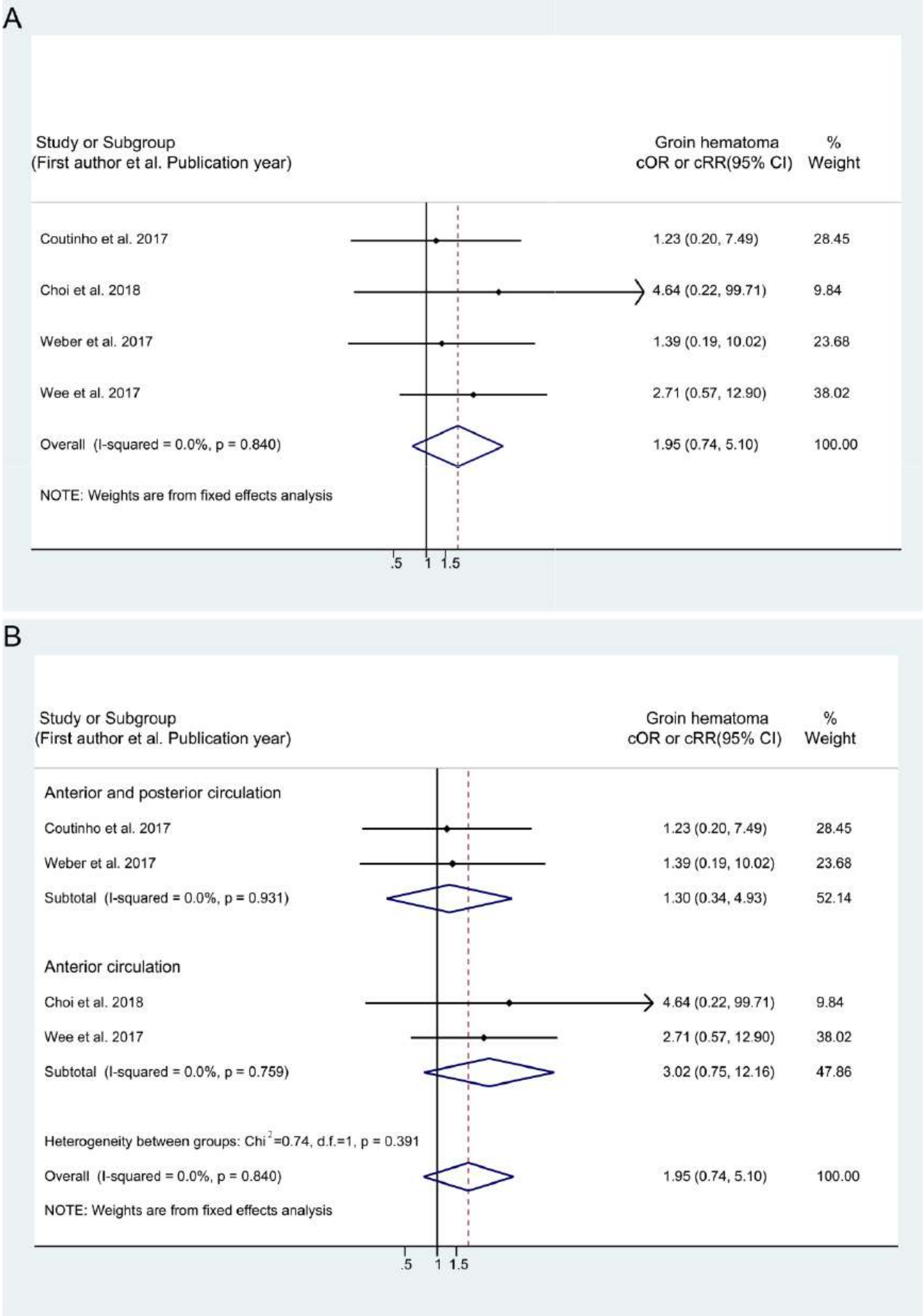

**Supplemental Figure 85.** Forest plots of subgroup analysis assessing groin hematoma by study type (A) and ethnicity (B) in unadjusted analysis.

A

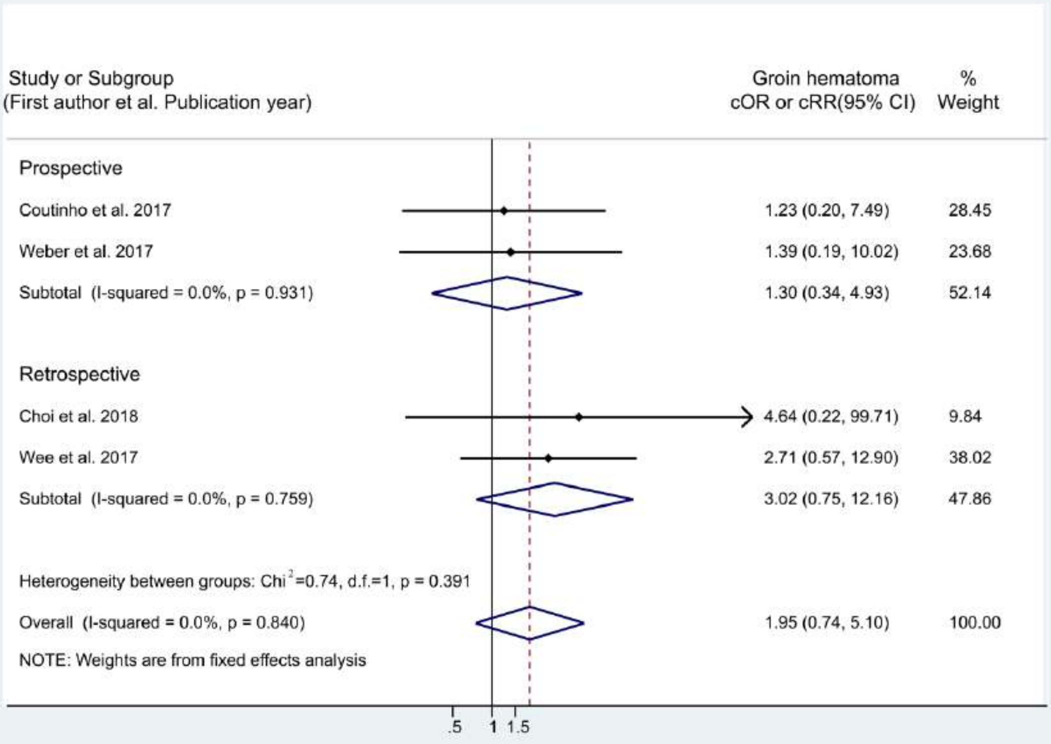


B

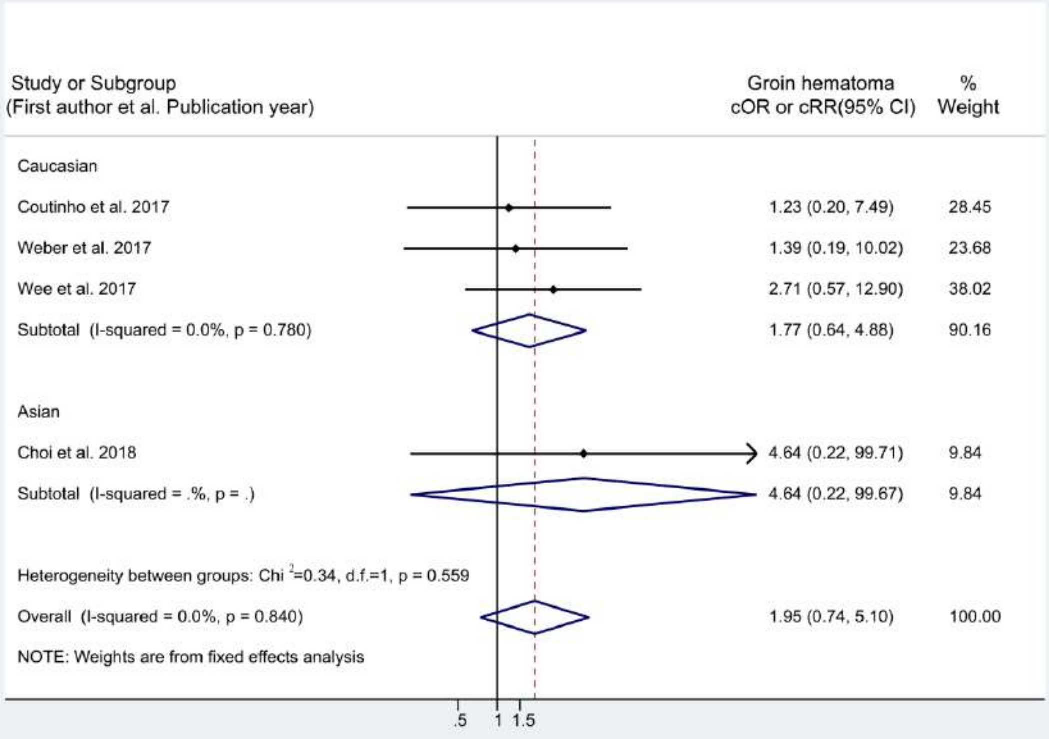

**Supplemental Figure 86.** Forest plots of studies assessing rescue therapy in unadjusted (A) and adjusted (B) analysis.

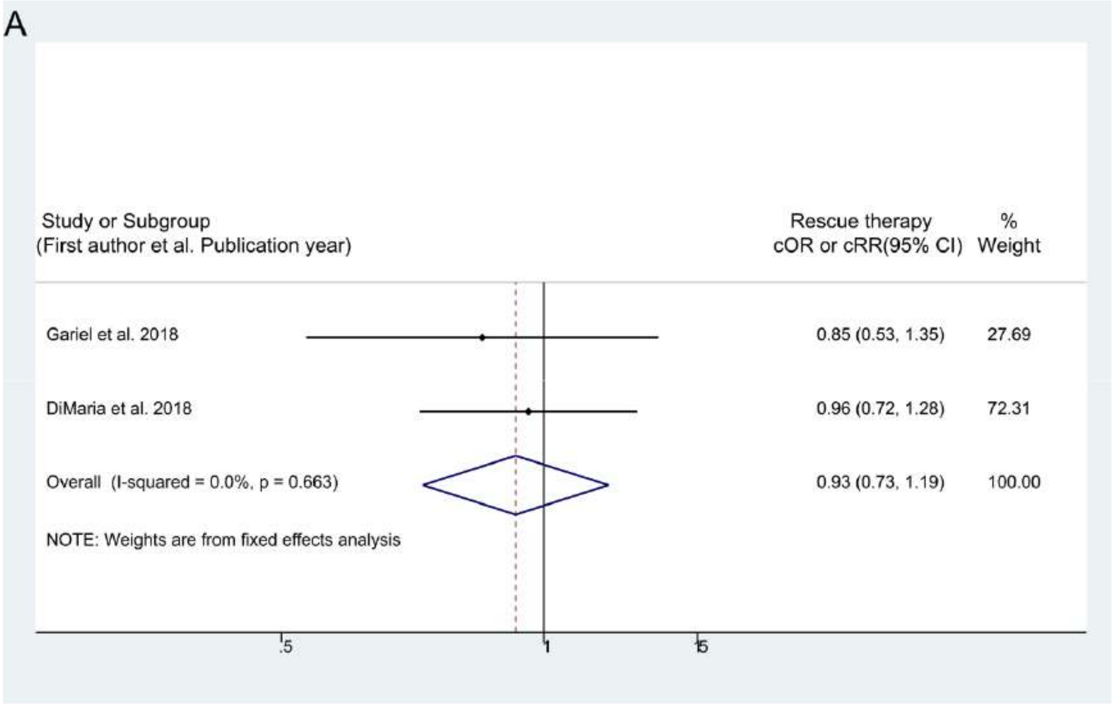


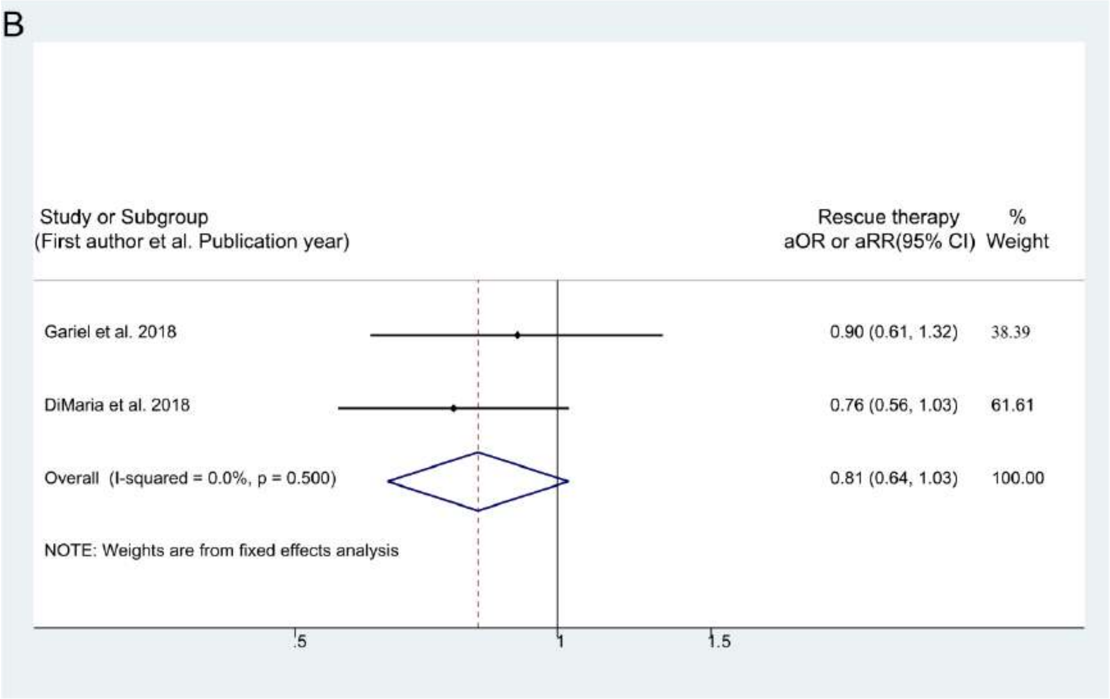

**Supplemental Figure 87.** Forest plots of studies assessing vasospasm in unadjusted (A) and adjusted (B) analysis.

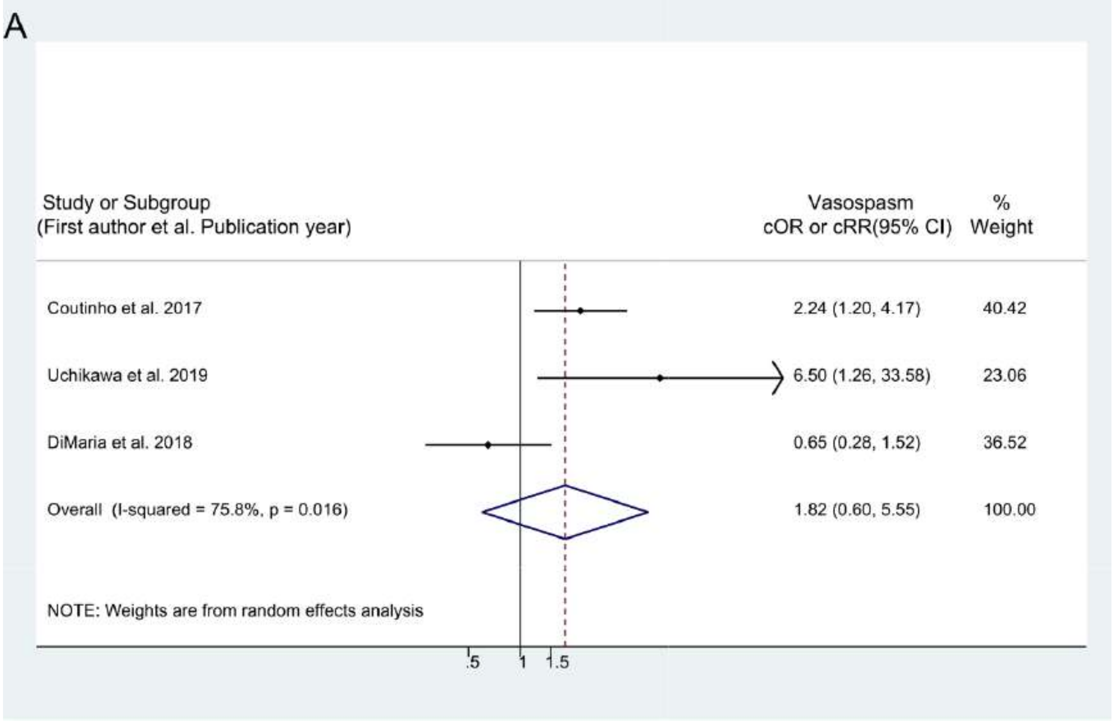


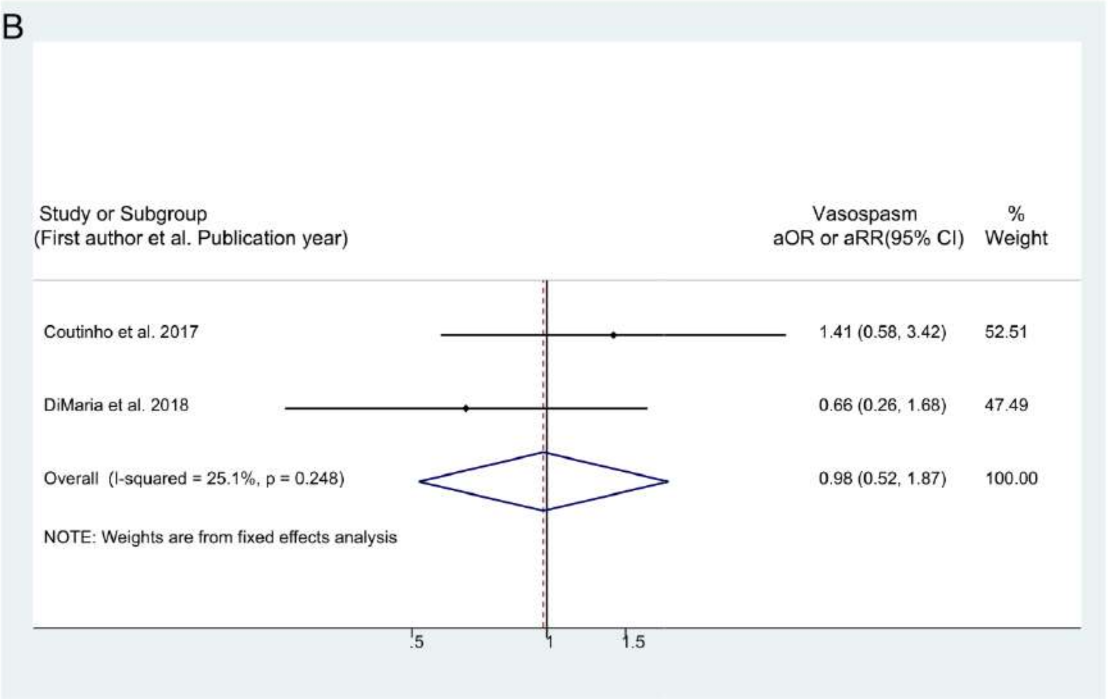

**Supplemental Figure 88.** Forest plots of subgroup analysis assessing vasospasm by location of occluded artery (A) and study type (B) in unadjusted analysis.

A

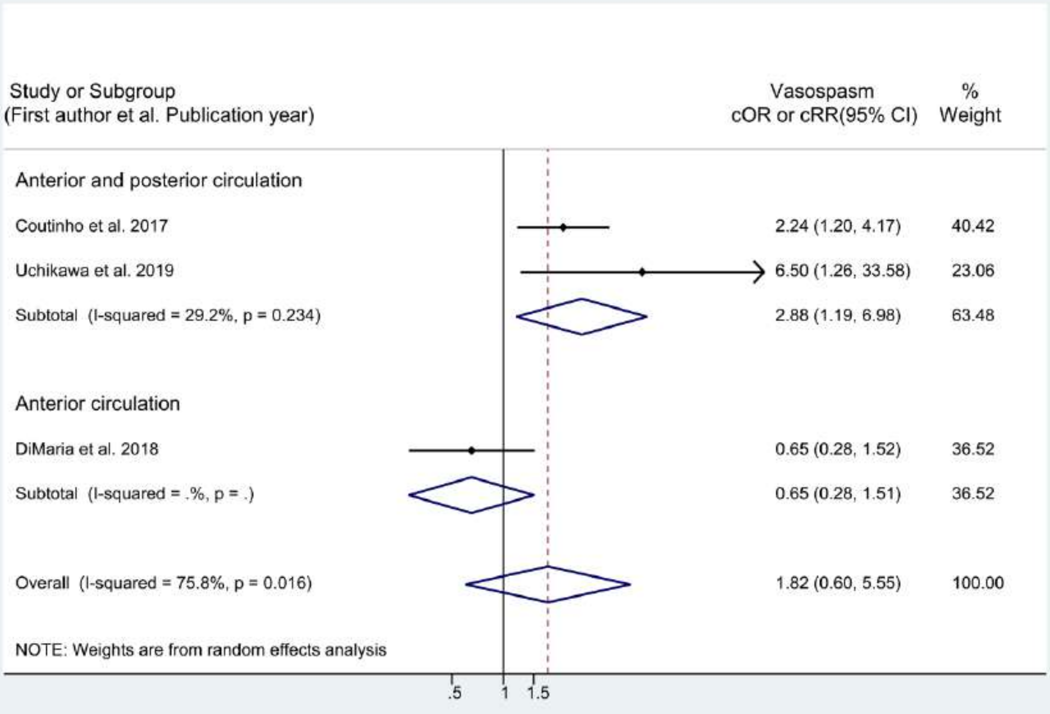


B

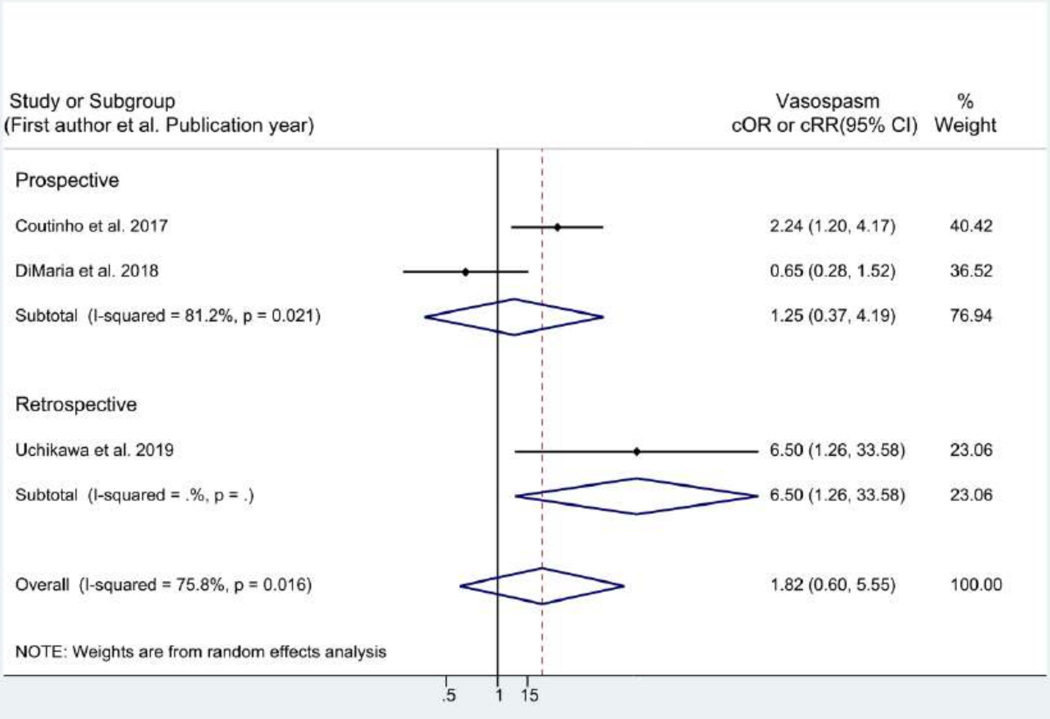

**Supplemental Figure 89.** Forest plots of subgroup analysis assessing vasospasm by ethnicity in unadjusted analysis.

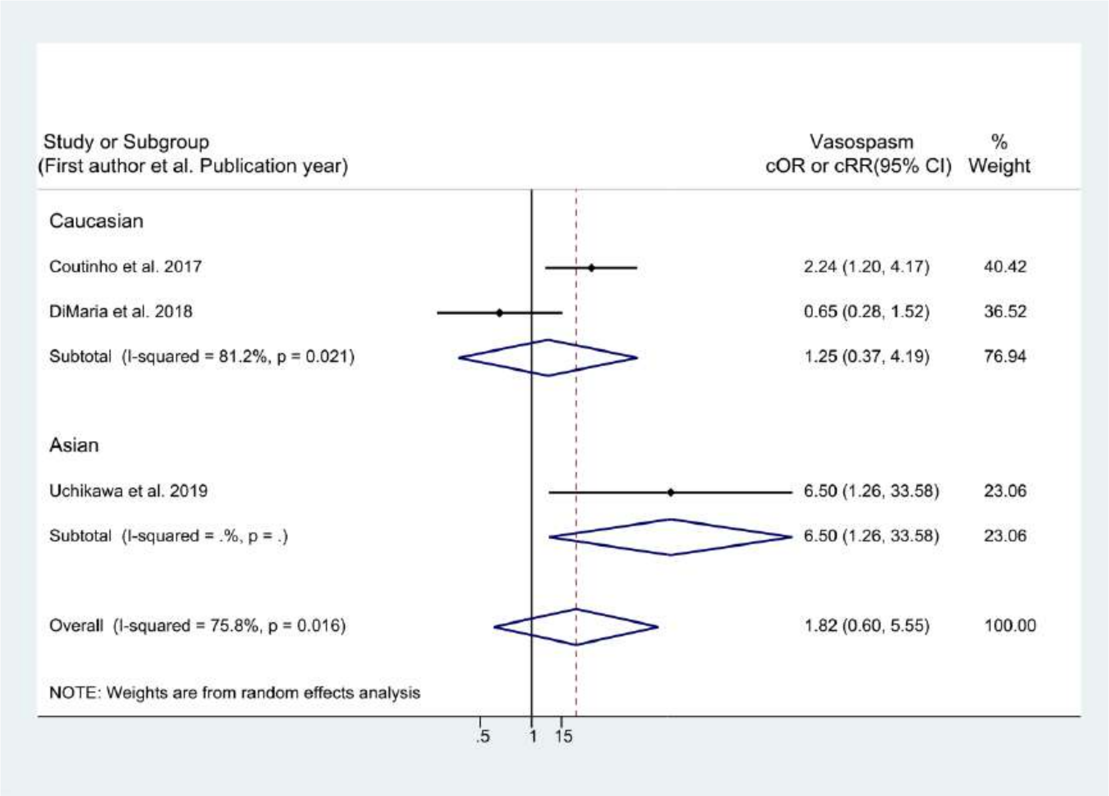

**Supplemental Figure 90.** Forest plots of studies assessing vessel dissection (A) and vessel perforation (B) in unadjusted analysis.

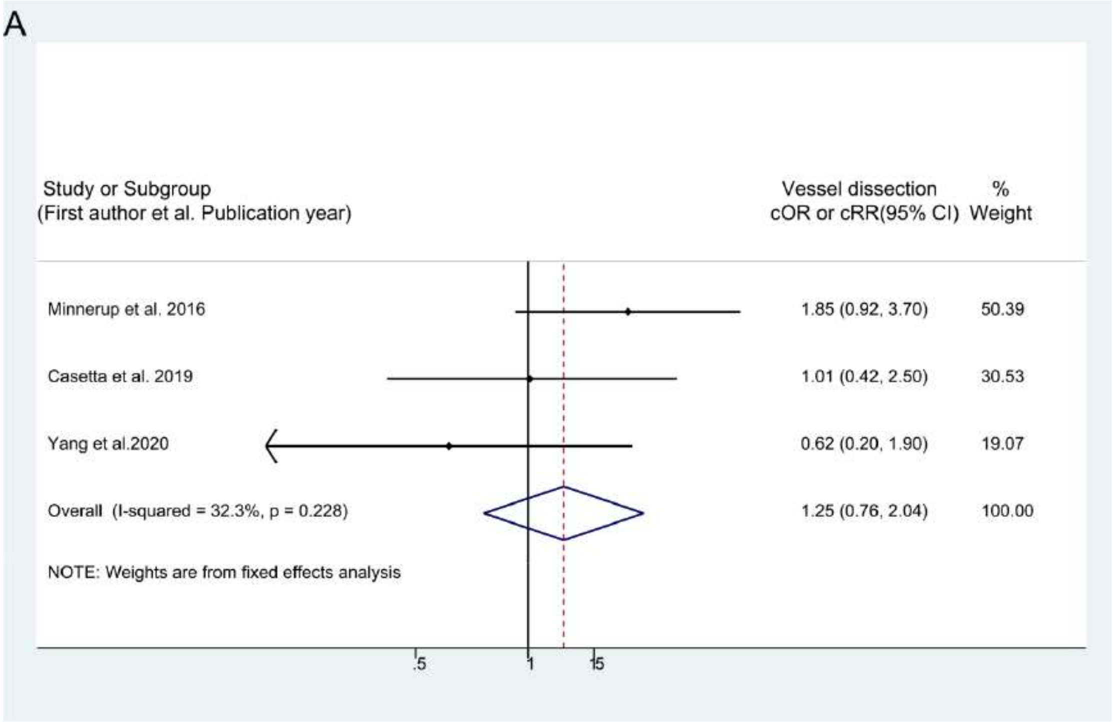


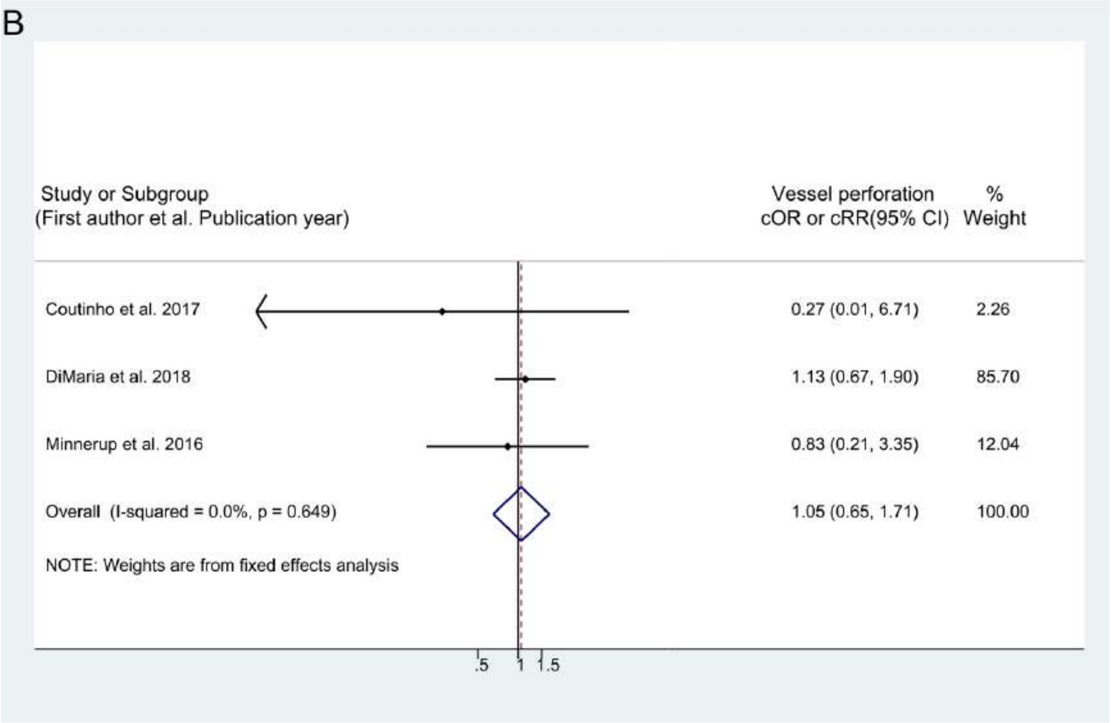

**Supplemental Figure 91.** Forest plots of subgroup analysis assessing vessel dissection (A) and vessel perforation (B) by location of occluded artery in unadjusted analysis.

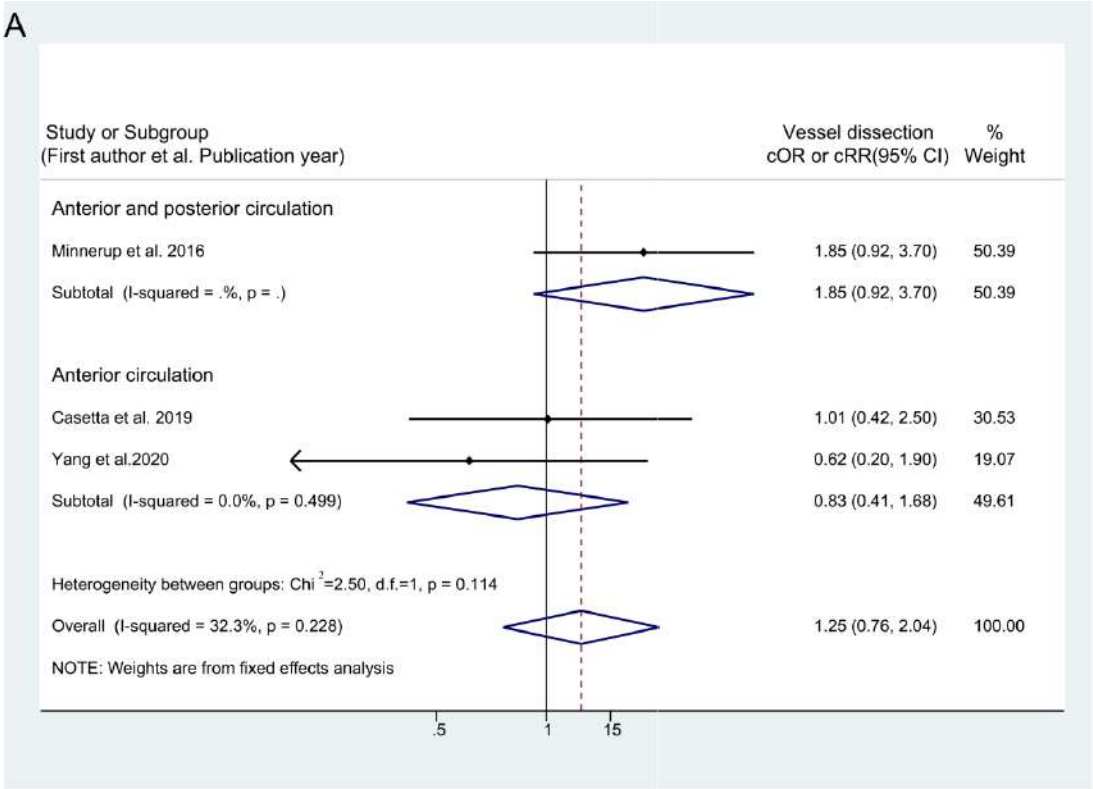


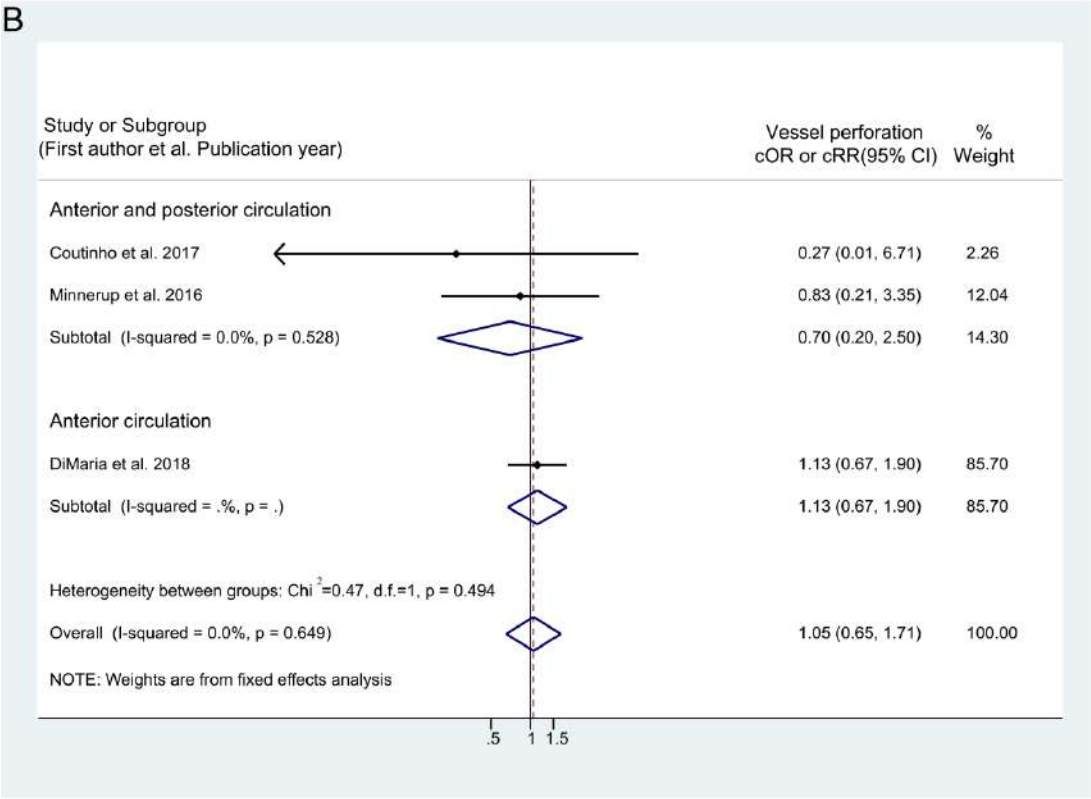

**Supplemental Figure 92.** Forest plots of subgroup analysis assessing vessel dissection by study type (A) and ethnicity (B) in unadjusted analysis.

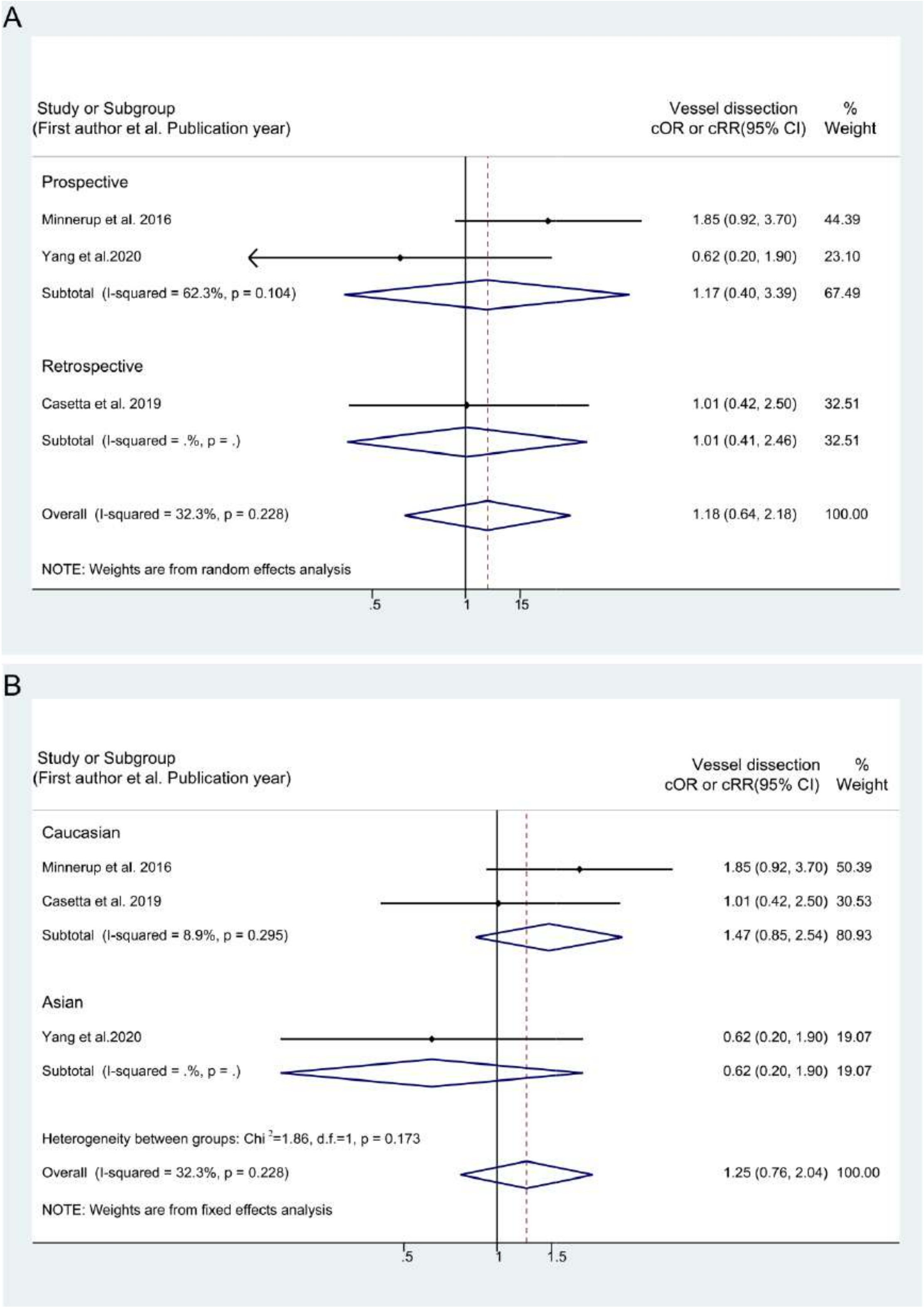

**Supplemental Figure 93.** Forest plots of studies assessing pneumonia (A) and recurrent stroke (B) in unadjusted analysis.

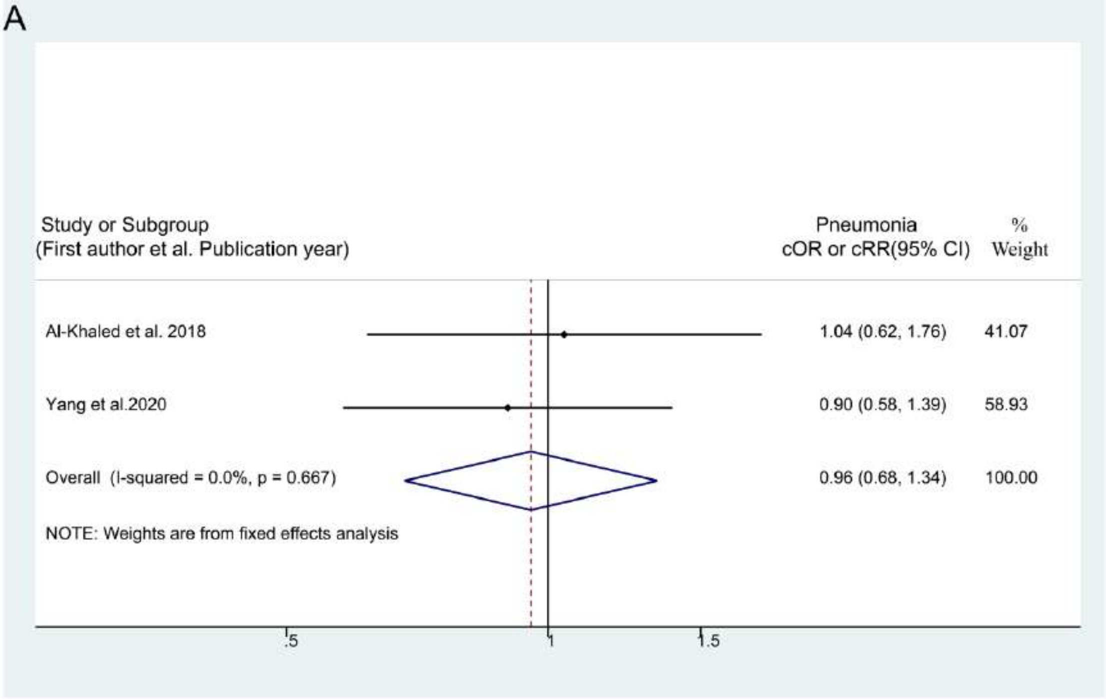


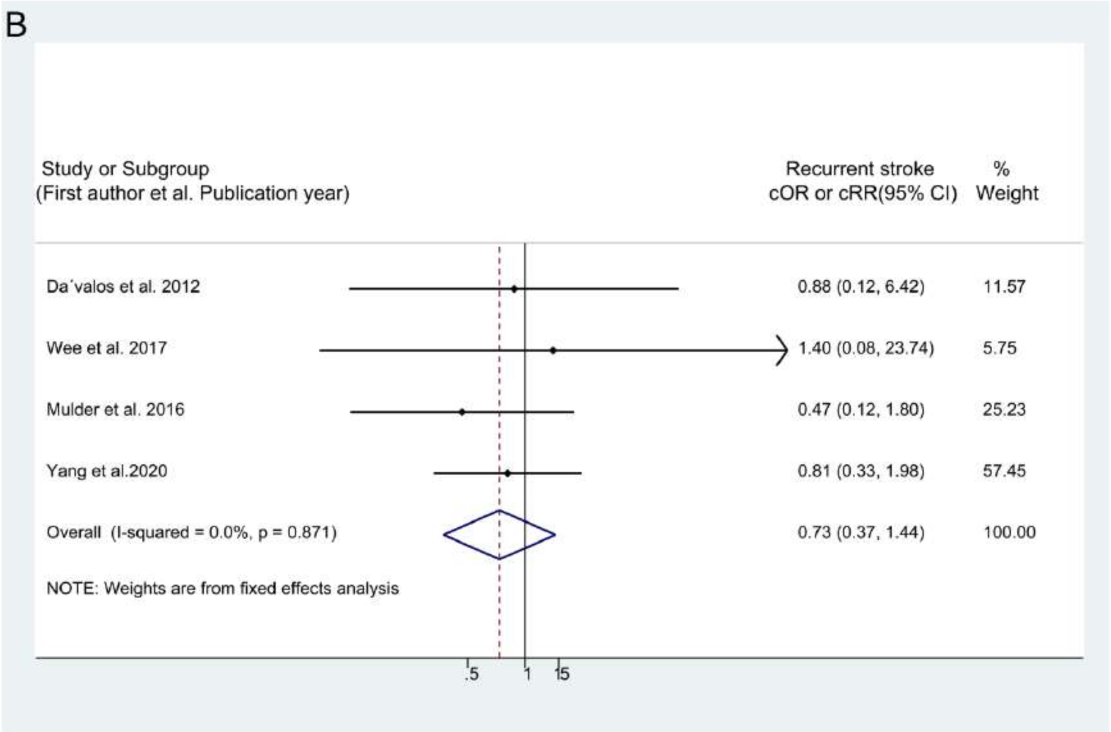

**Supplemental Figure 94.** Forest plots of subgroup analysis assessing recurrent stroke by location of occluded artery (A) and study type (B) in unadjusted analysis.

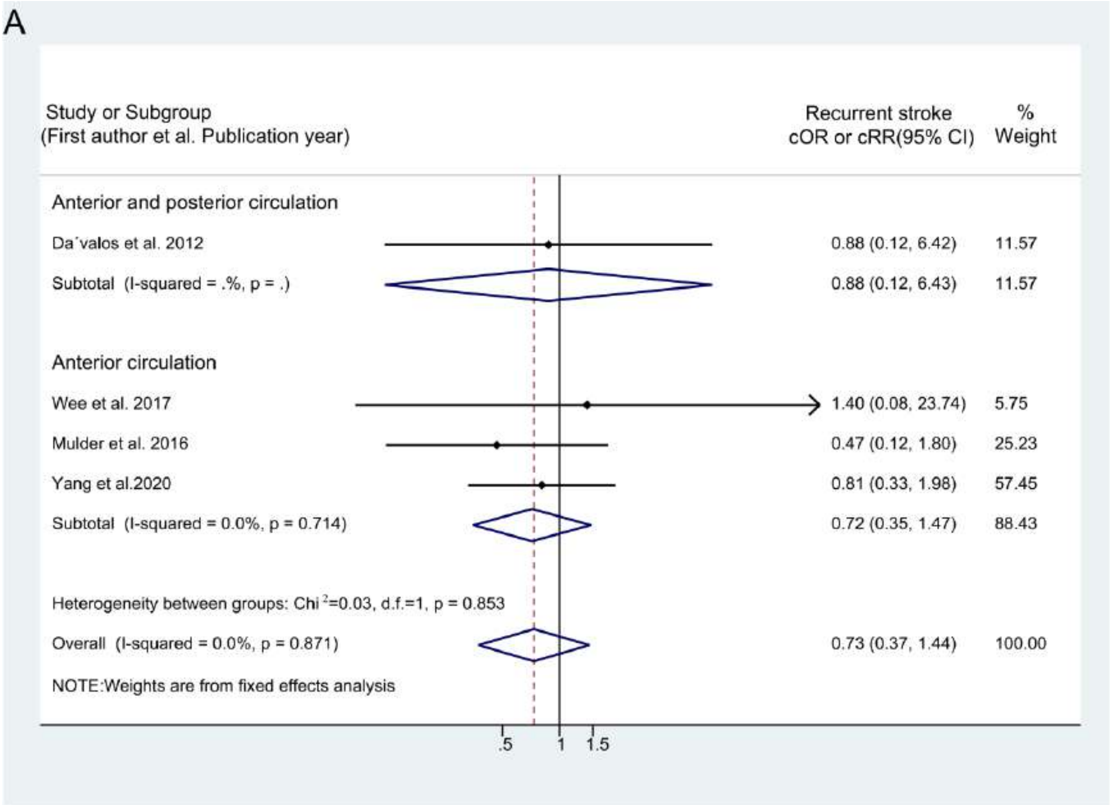


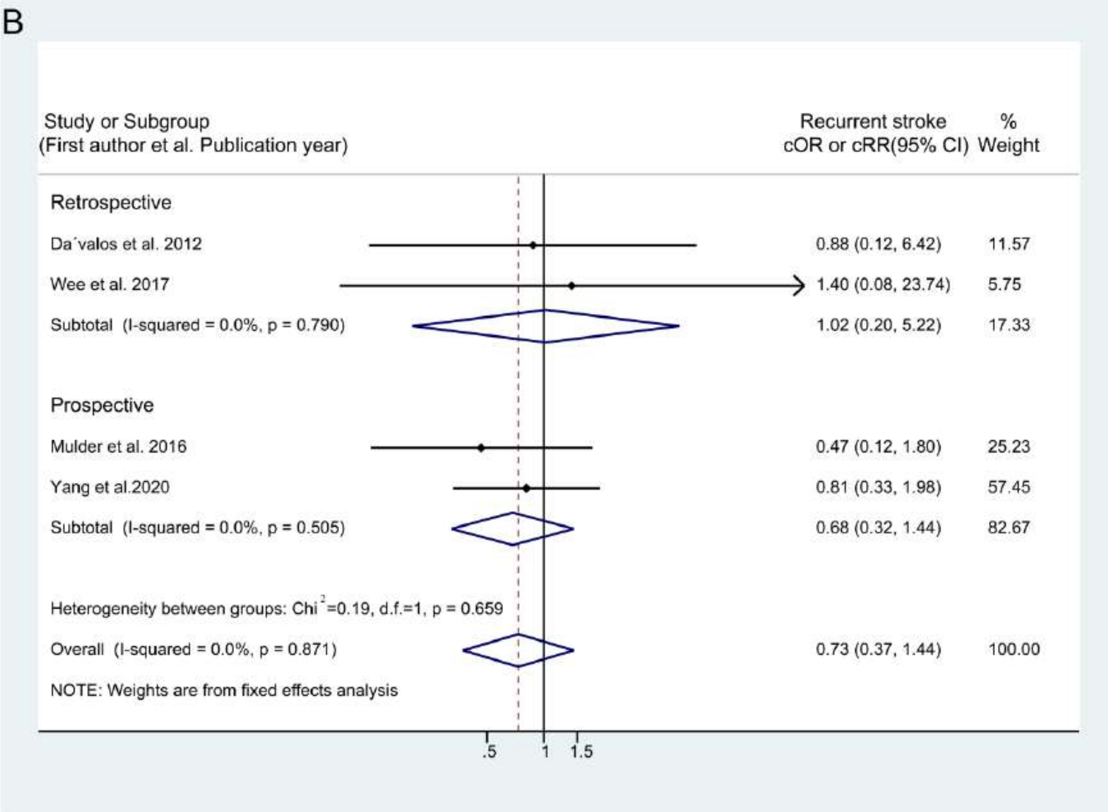

**Supplemental Figure 95.** Forest plots of subgroup analysis assessing recurrent stroke by ethnicity in unadjusted analysis.

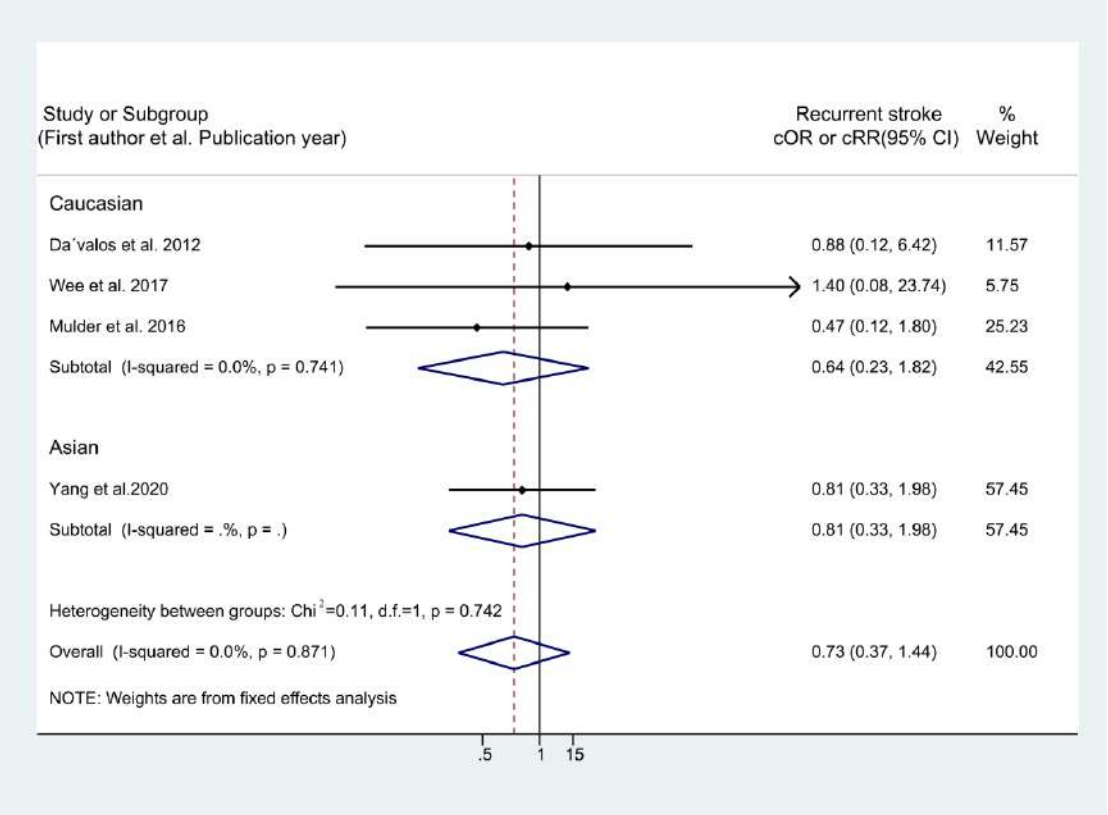

**Supplemental Figure 96.** Sensitivity analysis of the effect of individual studies on the cOR (A) and aOR (B) for good outcome at 90 days. cOR, crude odds ratio; aOR, adjusted odds ratio; ICA, internal carotid artery. [*]The acute intracranial vessel occlusion patients with (a) or without (b) concomitant ipsilateral ICA-occlusion or high-grade stenosis were investigated respectively in the study by Bücke et al. 2018.

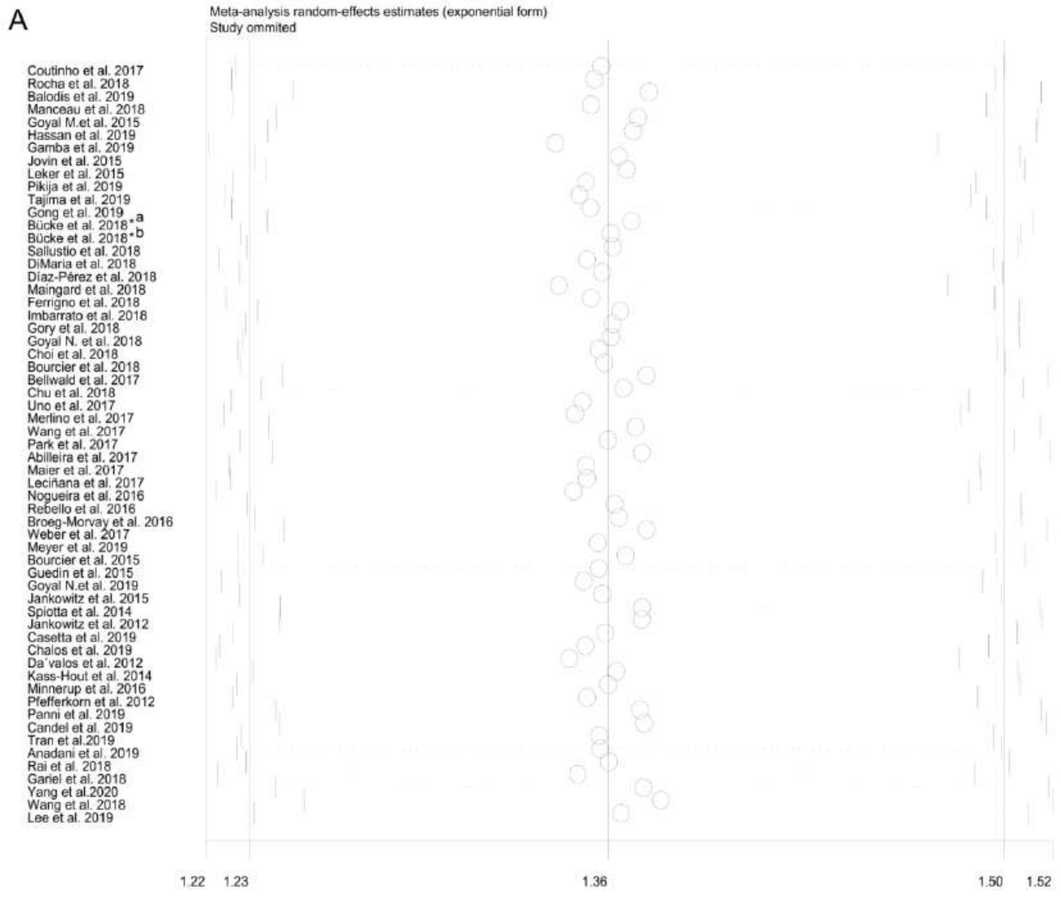

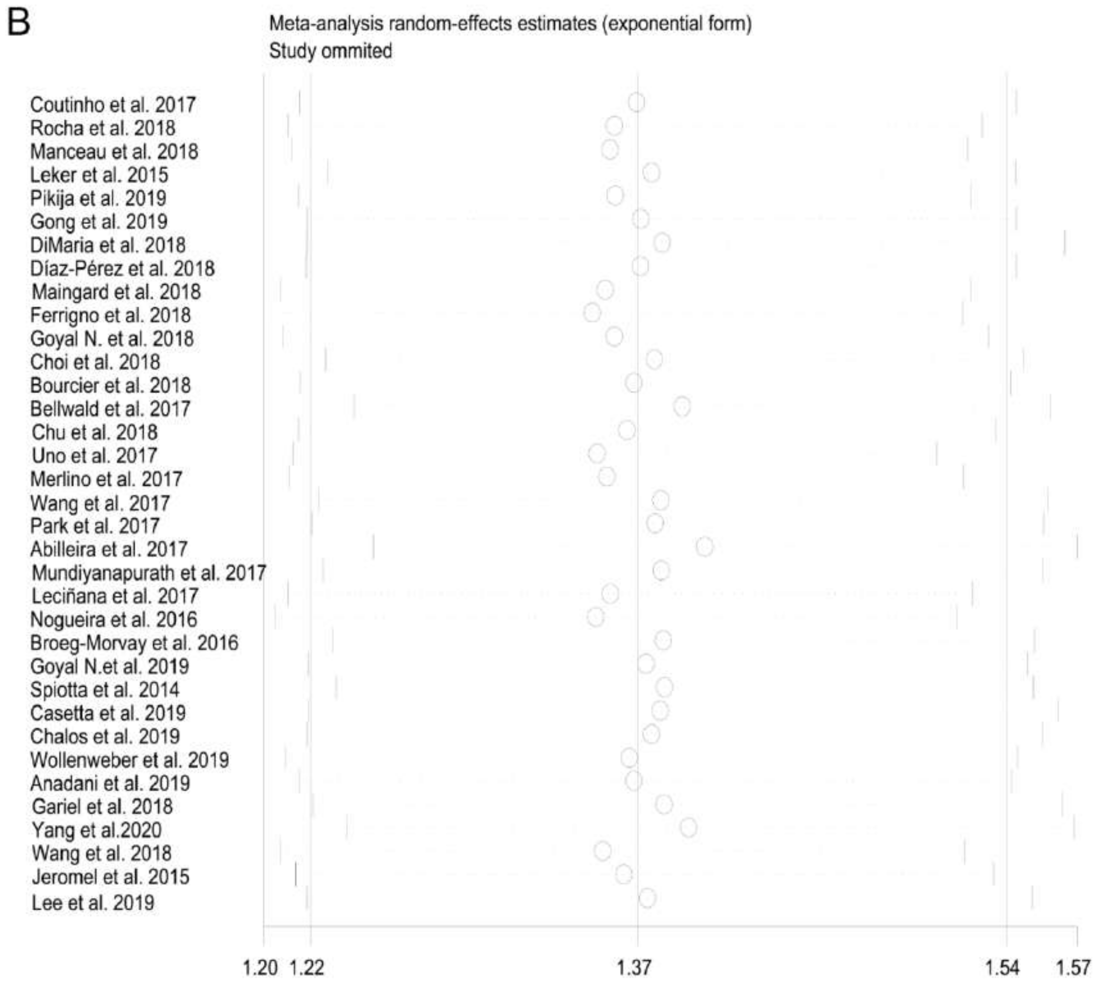
B
Meta-analysis random-effects estimates (exponential form)
Study ommited
Coutinho et al. 2017
Rocha et al. 2018
Manceau et al. 2018
Leker et al. 2015
Pikija et al. 2019
Gong et al. 2019
DiMaria et al. 2018
Díaz-Pérez et al. 2018
Maingard et al. 2018
Ferrigno et al. 2018
Goyal N. et al. 2018
Choi et al. 2018
Bourcier et al. 2018
Bellwald et al. 2017
Chu et al. 2018
Uno et al. 2017
Merlino et al. 2017
Wang et al. 2017
Park et al. 2017
Abilleira et al. 2017
Mundiyanapurath et al. 2017
Leciñana et al. 2017
Nogueira et al. 2016
Broeg-Morvay et al. 2016
Goyal N.et al. 2019
Spiotta et al. 2014
Casetta et al. 2019
Chalos et al. 2019
Wollenweber et al. 2019
Anadani et al. 2019
Gariel et al. 2018
Yang et al.2020
Wang et al. 2018
Jeromel et al. 2015
Lee et al. 2019
1.20 1.22
1.37
1.54 1.57

**Supplemental Figure 97.** Sensitivity analysis of the effect of individual studies on the cOR (A) and aOR (B) for excellent outcome at 90 days. cOR, crude odds ratio; aOR, adjusted odds ratio.

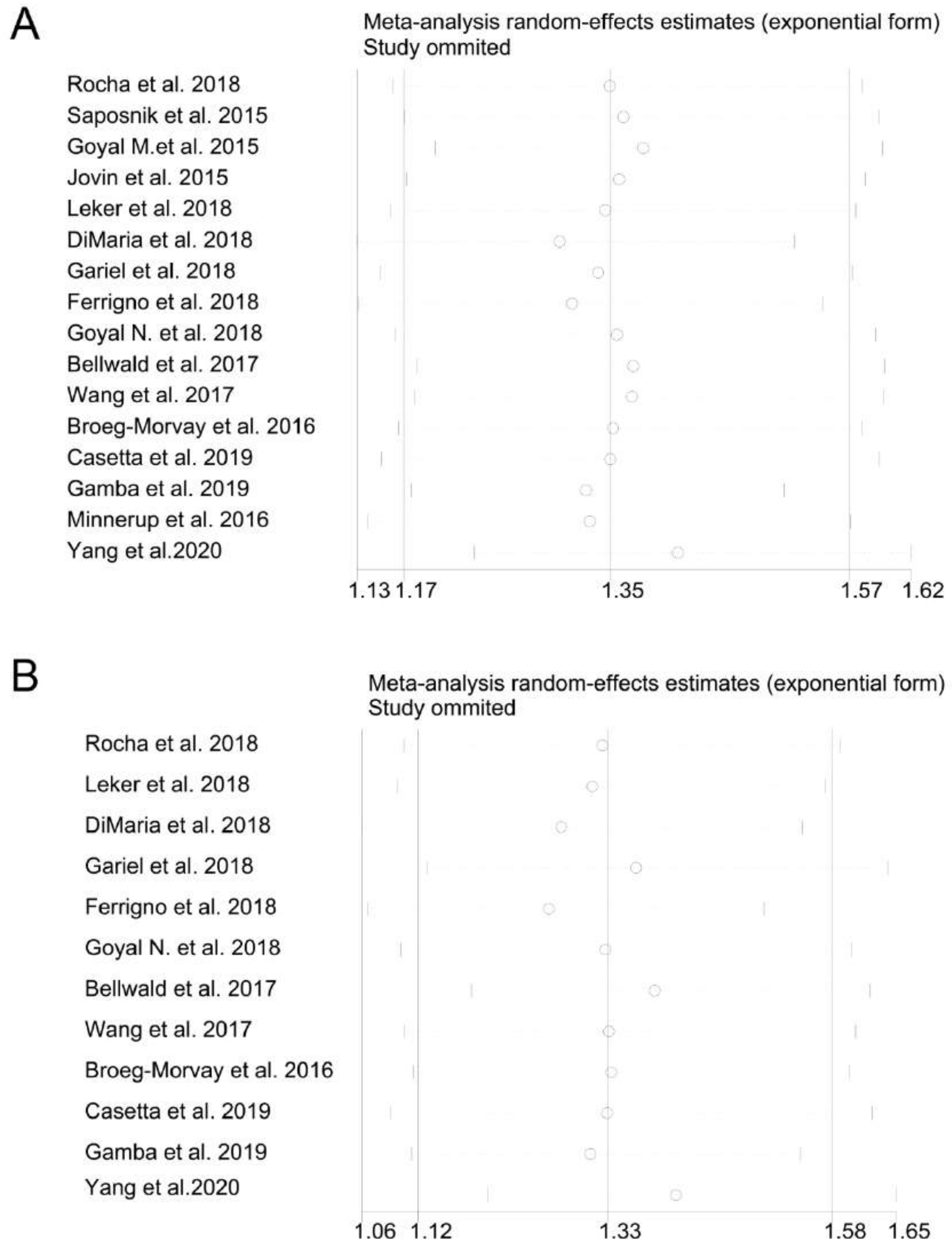

**Supplemental Figure 98.** Sensitivity analysis of the effect of individual studies on the cOR (A) and aOR (B) for favorable outcome at 90 days. cOR, crude odds ratio; aOR, adjusted odds ratio.

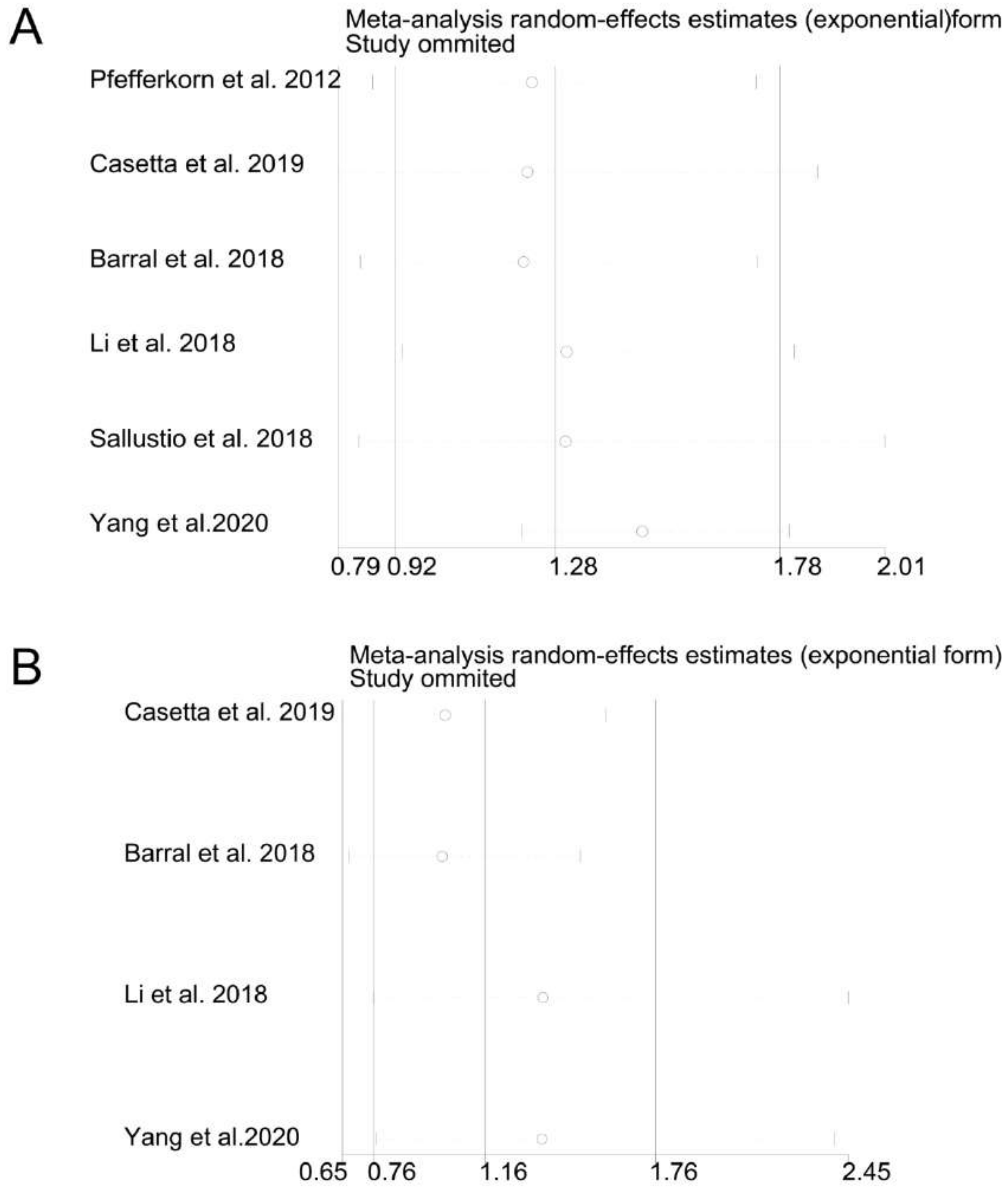

**Supplemental Figure 99.** Sensitivity analysis of the effect of individual studies on the cOR (A) and aOR (B) for mortality within 90 days. cOR, crude odds ratio; aOR, adjusted odds ratio. ICA, internal carotid artery. *The acute intracranial vessel occlusion patients with (a) or without (b) concomitant ipsilateral ICA-occlusion or high-grade stenosis were investigated respectively in the study by Bücke et al. 2018.

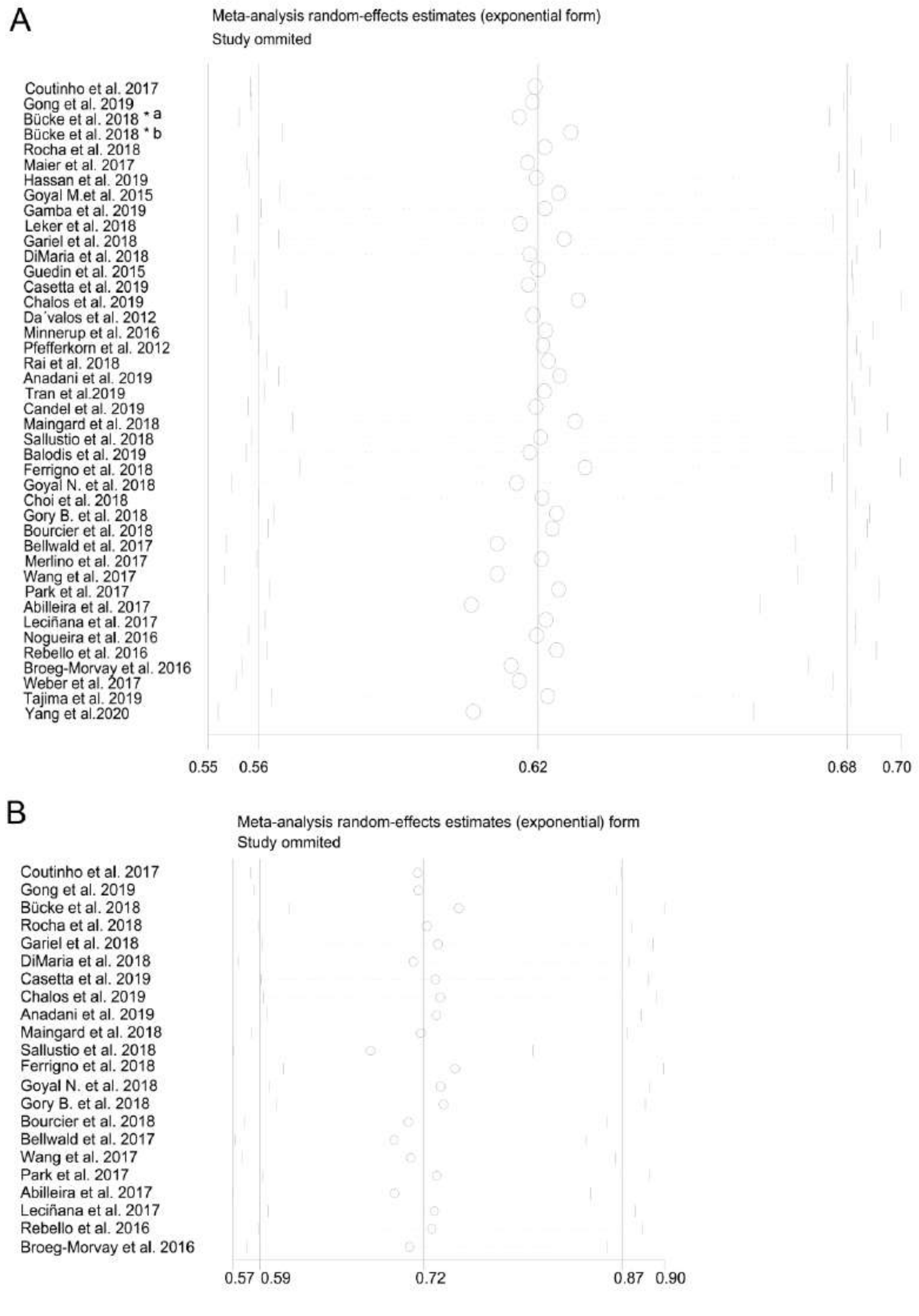

**Supplemental Figure 100.** Sensitivity analysis of the effect of individual studies on the cOR (A) and aOR (B) for all mortality. cOR, crude odds ratio; aOR, adjusted odds ratio. ICA, internal carotid artery. [*]The acute intracranial vessel occlusion patients with (a) or without (b) concomitant ipsilateral ICA-occlusion or high-grade stenosis were investigated respectively in the study by Bücke et al. 2018.

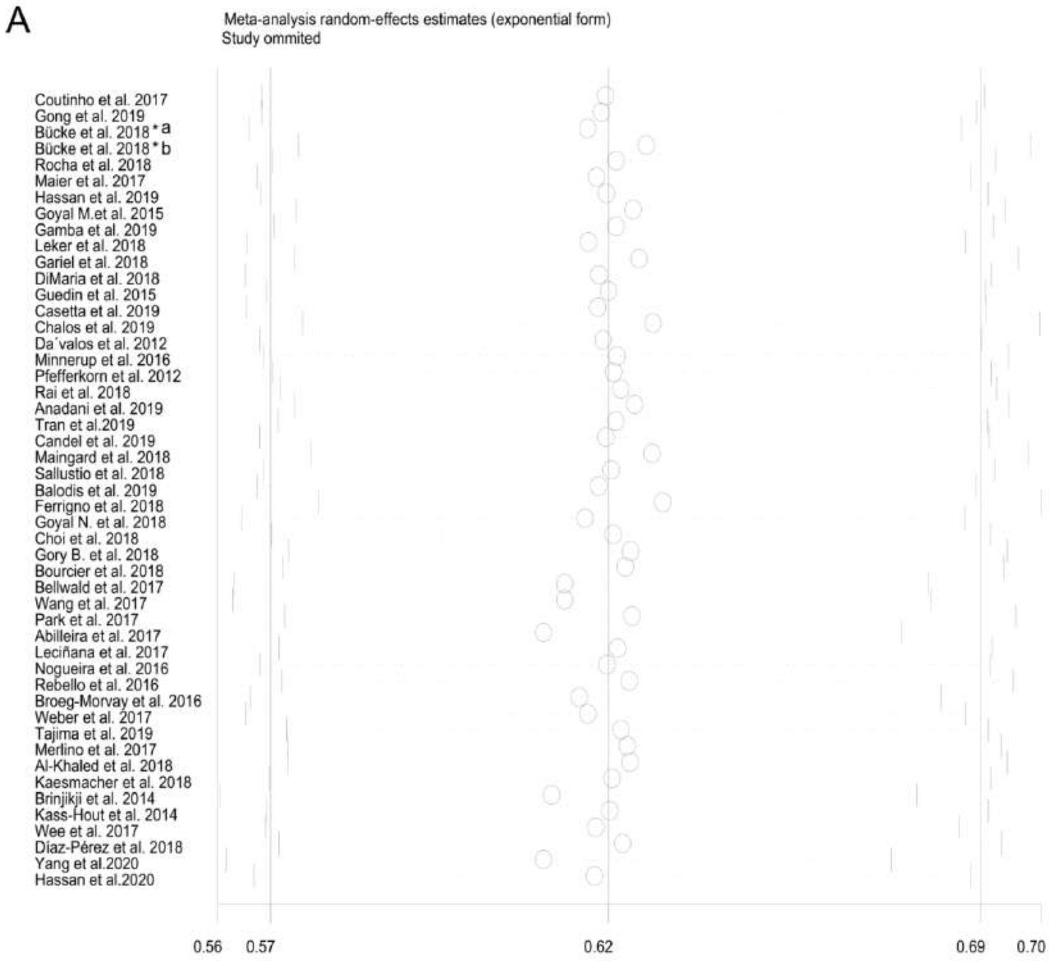

B

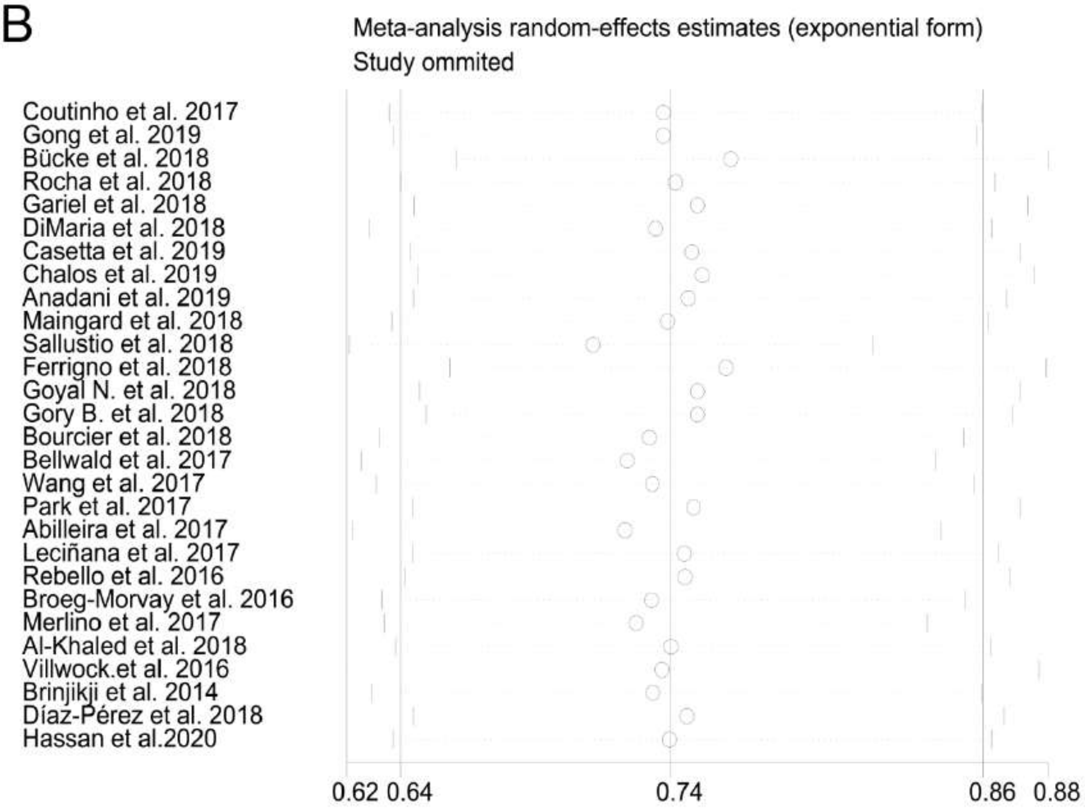
Meta-analysis random-effects estimates (exponential form)
Study ommited
Coutinho et al. 2017
Gong et al. 2019
Bücke et al. 2018
Rocha et al. 2018
Gariel et al. 2018
DiMaria et al. 2018
Casetta et al. 2019
Chalos et al. 2019
Anadani et al. 2019
Maingard et al. 2018
Sallustio et al. 2018
Ferrigno et al. 2018
Goyal N. et al. 2018
Gory B. et al. 2018
Bourcier et al. 2018
Bellwald et al. 2017
Wang et al. 2017
Park et al. 2017
Abilleira et al. 2017
Leciñana et al. 2017
Rebello et al. 2016
Broeg-Morvay et al. 2016
Merlino et al. 2017
Al-Khaled et al. 2018
Villwock.et al. 2016
Brinjikji et al. 2014
Díaz-Pérez et al. 2018
Hassan et al.2020
0.62
0.64
0.74
0.86
0.88

**Supplemental Figure 101.** Sensitivity analysis of the effect of individual studies on the cOR (A) and aOR (B) for successful reperfusion. cOR, crude odds ratio; aOR, adjusted odds ratio. ICA, internal carotid artery. [*]The acute intracranial vessel occlusion patients with (a) or without (b) concomitant ipsilateral ICA-occlusion or high-grade stenosis were investigated respectively in the study by Bücke et al. 2018.

A

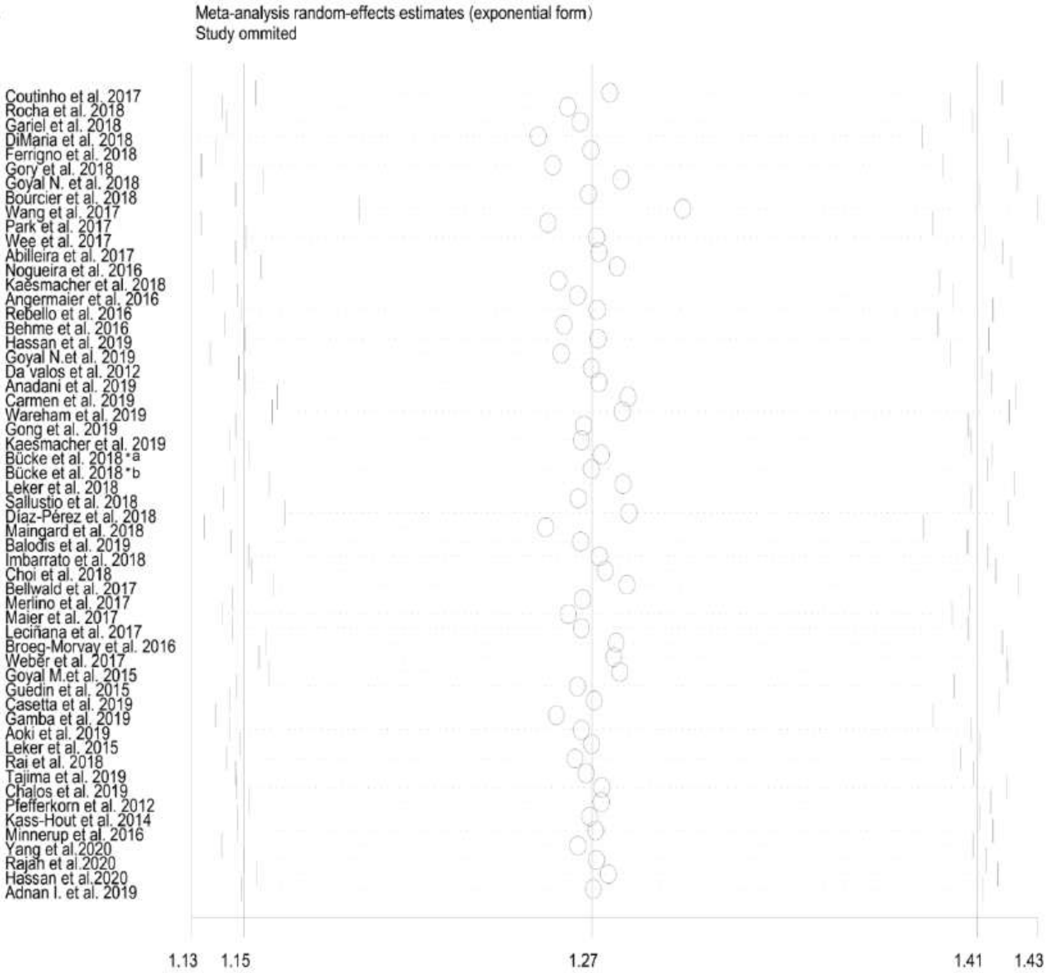

B

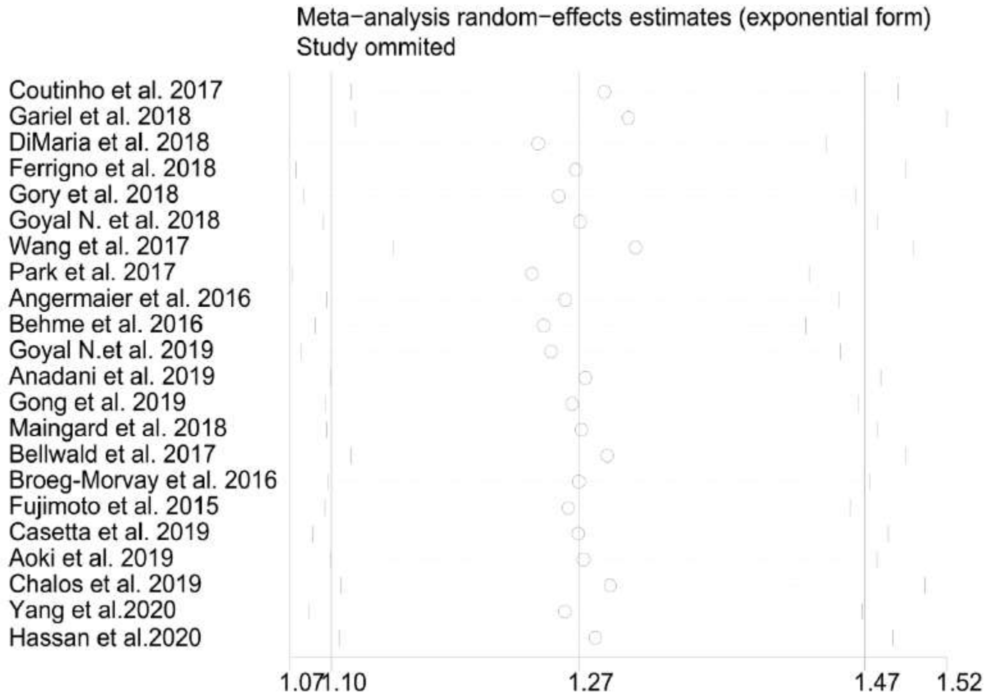

Meta−analysis random−effects estimates (exponential form)
Study ommited
Coutinho et al. 2017
Gariel et al. 2018
DiMaria et al. 2018
Ferrigno et al. 2018
Gory et al. 2018
Goyal N. et al. 2018
Wang et al. 2017
Park et al. 2017
Angermaier et al. 2016
Behme et al. 2016
Goyal N.et al. 2019
Anadani et al. 2019
Gong et al. 2019
Maingard et al. 2018
Bellwald et al. 2017
Broeg-Morvay et al. 2016
Fujimoto et al. 2015
Casetta et al. 2019
Aoki et al. 2019
Chalos et al. 2019
Yang et al.2020
Hassan et al.2020
1.07
1.10
1.27
1.47
1.52

**Supplemental Figure 102.** Sensitivity analysis of the effect of individual studies on the cOR (A) and aOR (B) for complete reperfusion. cOR, crude odds ratio; aOR, adjusted odds ratio.

A

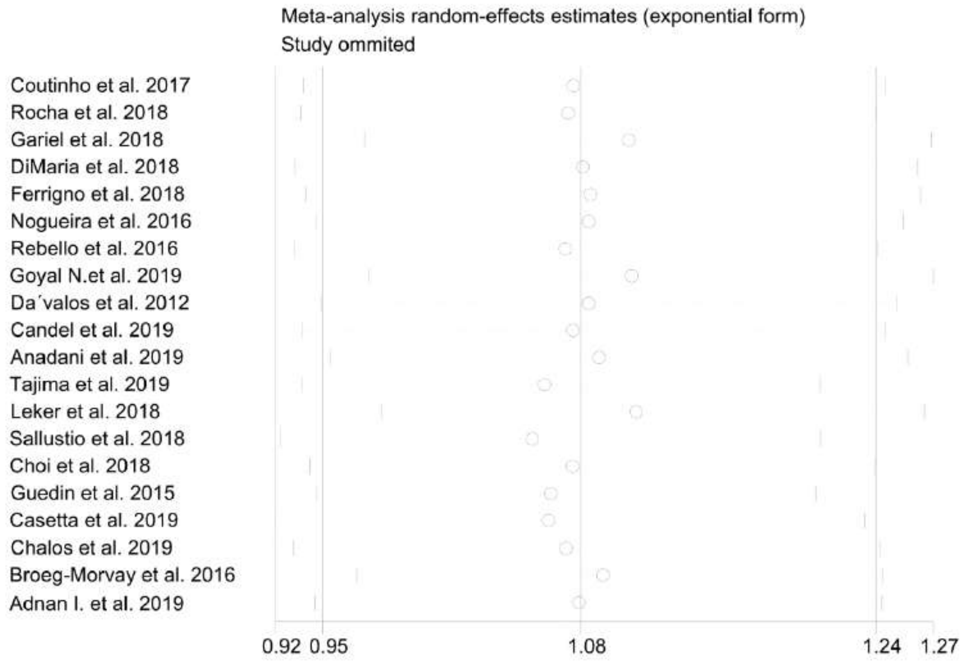


B

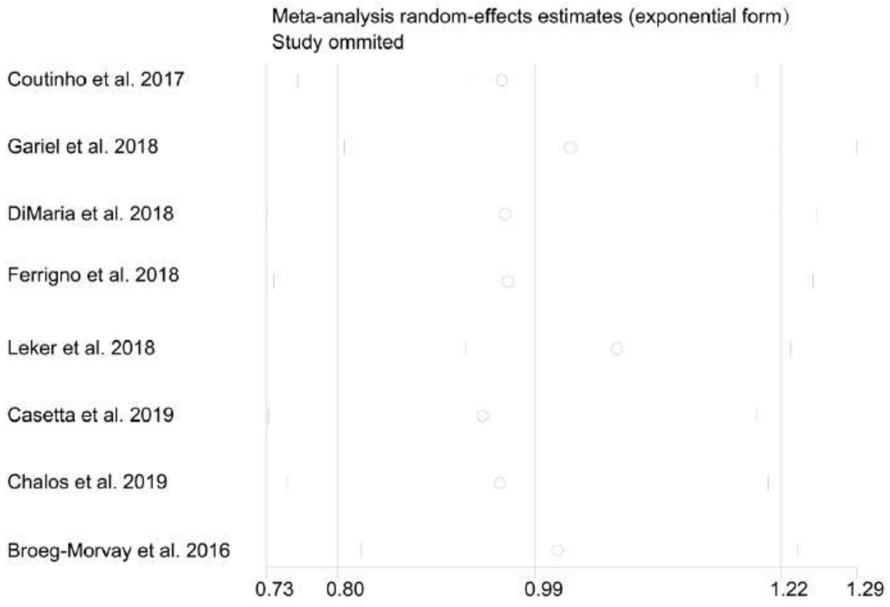

**Supplemental Figure 103.** Sensitivity analysis of the effect of individual studies on the cOR (A) and aOR (B) for any ICH. cOR, crude odds ratio; aOR, adjusted odds ratio. ICH, intracranial hemorrhage; sICH, symptomatic intracranial hemorrhage; aICH, asymptomatic intracranial hemorrhage; ICA, internal carotid artery. *The acute intracranial vessel occlusion patients with (a) or without (b) concomitant ipsilateral ICA-occlusion or high-grade stenosis were investigated respectively in the study by Bücke et al. 2018. #The outcomes of sICH (c) and aICH (d) were investigated respectively in the studies of Hassan et al. 2019, Balodis et al. 2019, Broeg-Morvay et al. 2016, Bellwald et al. 2017, Wang et al. 2017, Yang et al.2020 and Hassan et al.2020

A

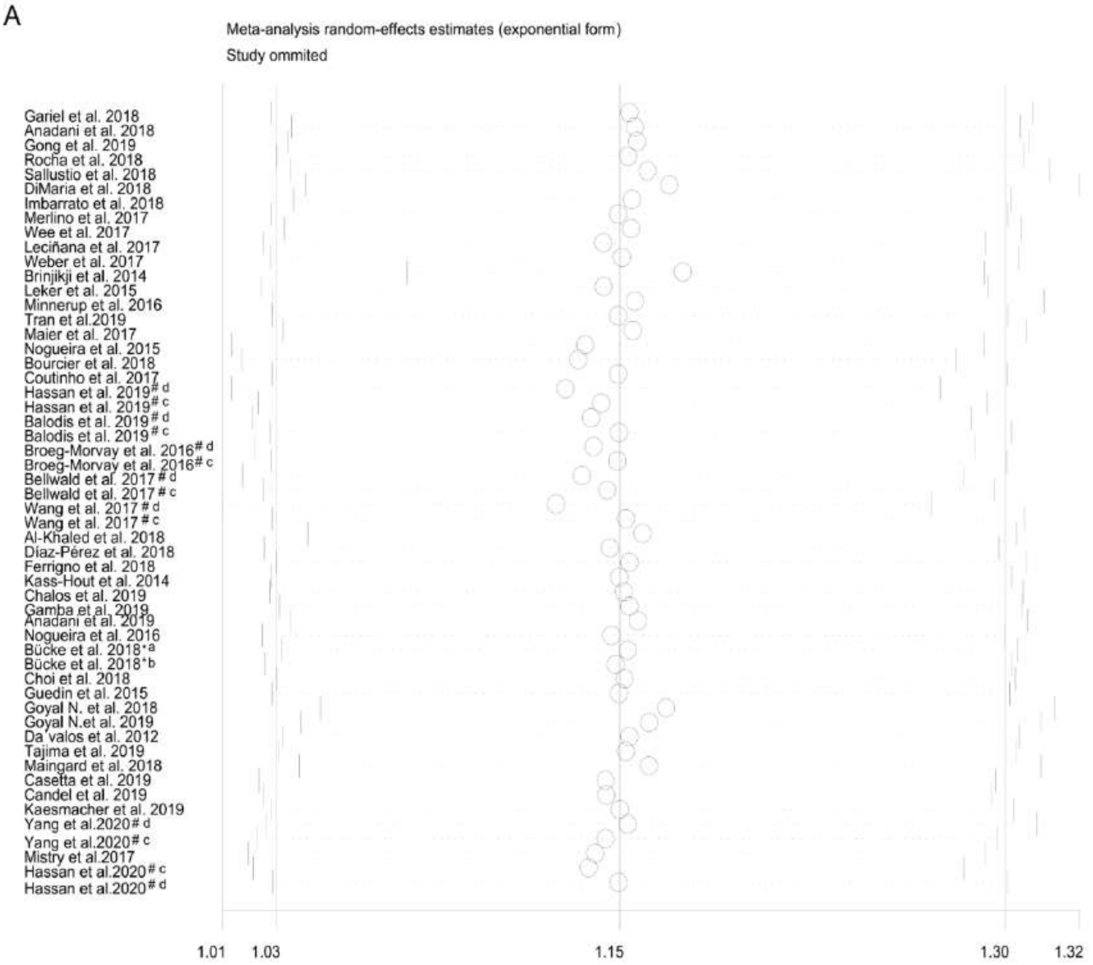

Meta-analysis random-effects estimates (exponential form)
Study ommited
Gariel et al. 2018
Anadani et al. 2018
Gong et al. 2019
Rocha et al. 2018
Sallustio et al. 2018
DiMaria et al. 2018
Imbarrato et al. 2018
Merlino et al. 2017
Wee et al. 2017
Leciñana et al. 2017
Weber et al. 2017
Brinjikji et al. 2014
Leker et al. 2015
Minnerup et al. 2016
Tran et al.2019
Maier et al. 2017
Nogueira et al. 2015
Bourcier et al. 2018
Coutinho et al. 2017
Hassan et al. 2019# d
Hassan et al. 2019# c
Balodis et al. 2019# d
Balodis et al. 2019# c
Broeg-Morvay et al. 2016# d
Broeg-Morvay et al. 2016# c
Bellwald et al. 2017 # d
Bellwald et al. 2017 # c
Wang et al. 2017 # d
Wang et al. 2017 # c
Al-Khaled et al. 2018
Díaz-Pérez et al. 2018
Ferrigno et al. 2018
Kass-Hout et al. 2014
Chalos et al. 2019
Gamba et al. 2019
Anadani et al. 2019
Nogueira et al. 2016
Bücke et al. 2018*a
Bücke et al. 2018*b
Choi et al. 2018
Guedin et al. 2015
Goyal N. et al. 2018
Goyal N.et al. 2019
Da valos et al. 2012
Tajima et al. 2019
Maingard et al. 2018
Casetta et al. 2019
Candel et al. 2019
Kaesmacher et al. 2019
Yang et al.2020# d
Yang et al.2020# c
Mistry et al.2017
Hassan et al.2020# c
Hassan et al.2020# d
1.01
1.03
1.15
1.30
1.32


B

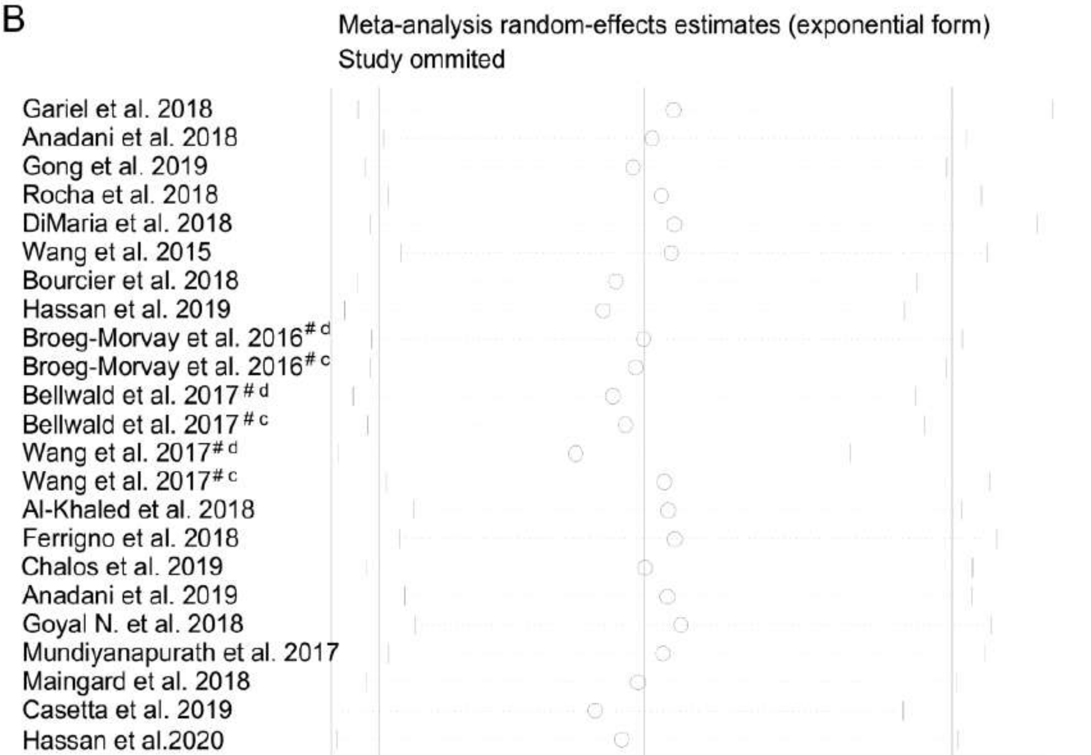

Meta-analysis random-effects estimates (exponential form)
Study ommited
Gariel et al. 2018
Anadani et al. 2018
Gong et al. 2019
Rocha et al. 2018
DiMaria et al. 2018
Wang et al. 2015
Bourcier et al. 2018
Hassan et al. 2019
Broeg-Morvay et al. 2016# d
Broeg-Morvay et al. 2016# c
Bellwald et al. 2017 # d
Bellwald et al. 2017 # c
Wang et al. 2017# d
Wang et al. 2017# c
Al-Khaled et al. 2018
Ferrigno et al. 2018
Chalos et al. 2019
Anadani et al. 2019
Goyal N. et al. 2018
Mundiyanapurath et al. 2017
Maingard et al. 2018
Casetta et al. 2019
Hassan et al.2020
1.011.04
1.21
1.42
1.48

**Supplemental Figure 104.** Sensitivity analysis of the effect of individual studies on the cOR for sICH. cOR, crude odds ratio; sICH, symptomatic intracranial hemorrhage; ICA, internal carotid artery. [*]The acute intracranial vessel occlusion patients with (a) or without (b) concomitant ipsilateral ICA-occlusion or high-grade stenosis were investigated respectively in the study by Bücke et al. 2018.

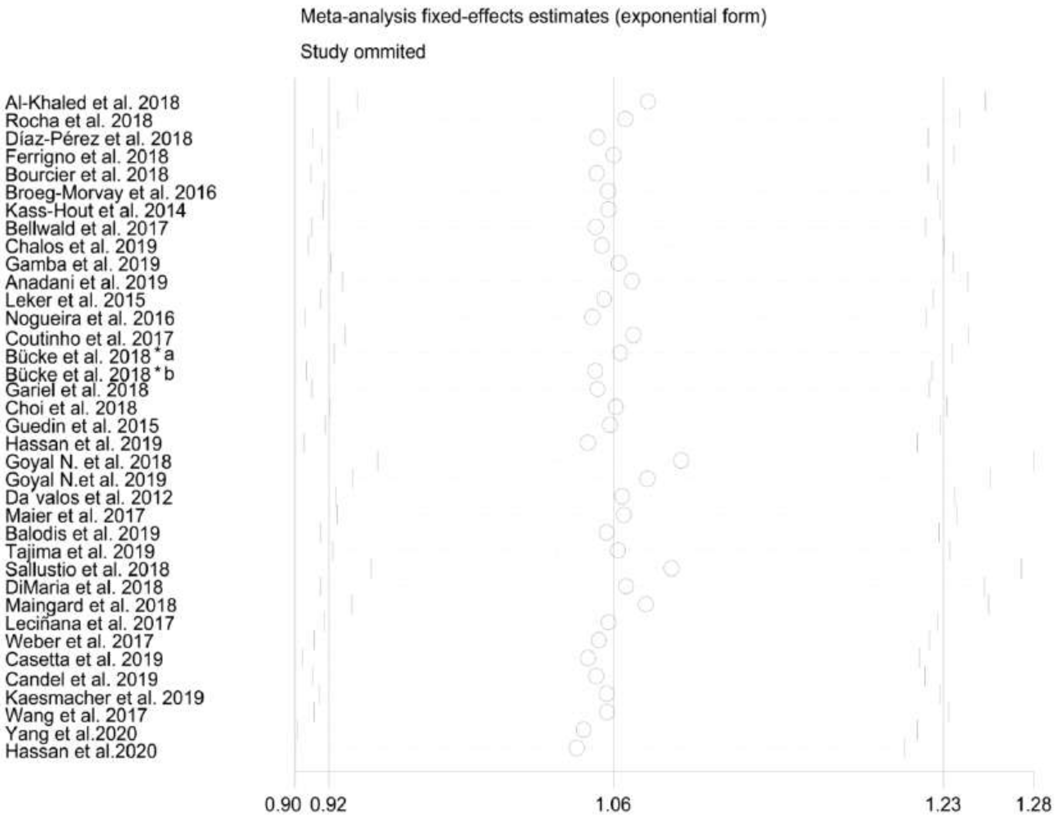

**Supplemental Figure 105.** Sensitivity analysis of the effect of individual studies on the cOR (A) and aOR (B) for aICH. cOR, crude odds ratio; aOR, adjusted odds ratio; aICH, asymptomatic intracranial hemorrhage.

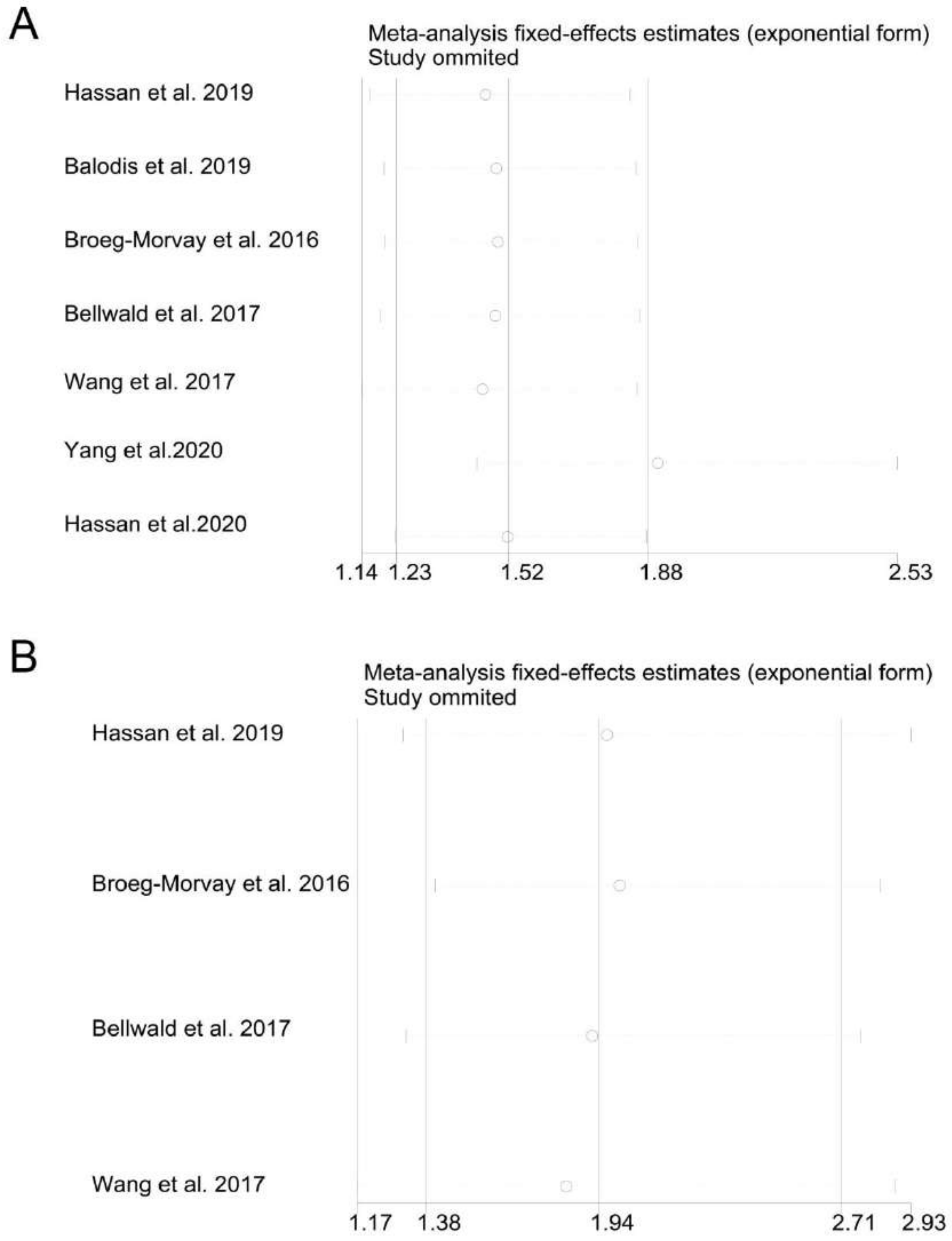

**Supplemental Figure 106.** Sensitivity analysis of the effect of individual studies on the cOR (A) and aOR (B) for any bleeding. cOR, crude odds ratio; aOR, adjusted odds ratio; ICA, internal carotid artery; sICH, symptomatic intracranial hemorrhage; aICH, asymptomatic intracranial hemorrhage; HT, hemorrhagic transformation; PH, parenchymal hematoma; SAH, subarachnoid hemorrhage. *The acute intracranial vessel occlusion patients with (a) or without (b) concomitant ipsilateral ICA-occlusion or high-grade stenosis were investigated respectively in the study by Bücke et al. 2018. #The outcomes of severe extracranial hemorrhage (c) and sICH (d) were investigated respectively in the study of Chalos et al. 2019; The outcomes of HT (e) and SAH (f) were investigated respectively in the study of Dávalos et al. 2012. The outcomes of PH (g) and SAH (f) were investigated respectively in the study of Rebello et al. 2016. The outcome of sICH (d) and aICH (h) were investigated respectively in the studies of Wang et al. 2017, Bellwald et al. 2017, Yang et al.2020 and Hassan et al.2020.

A

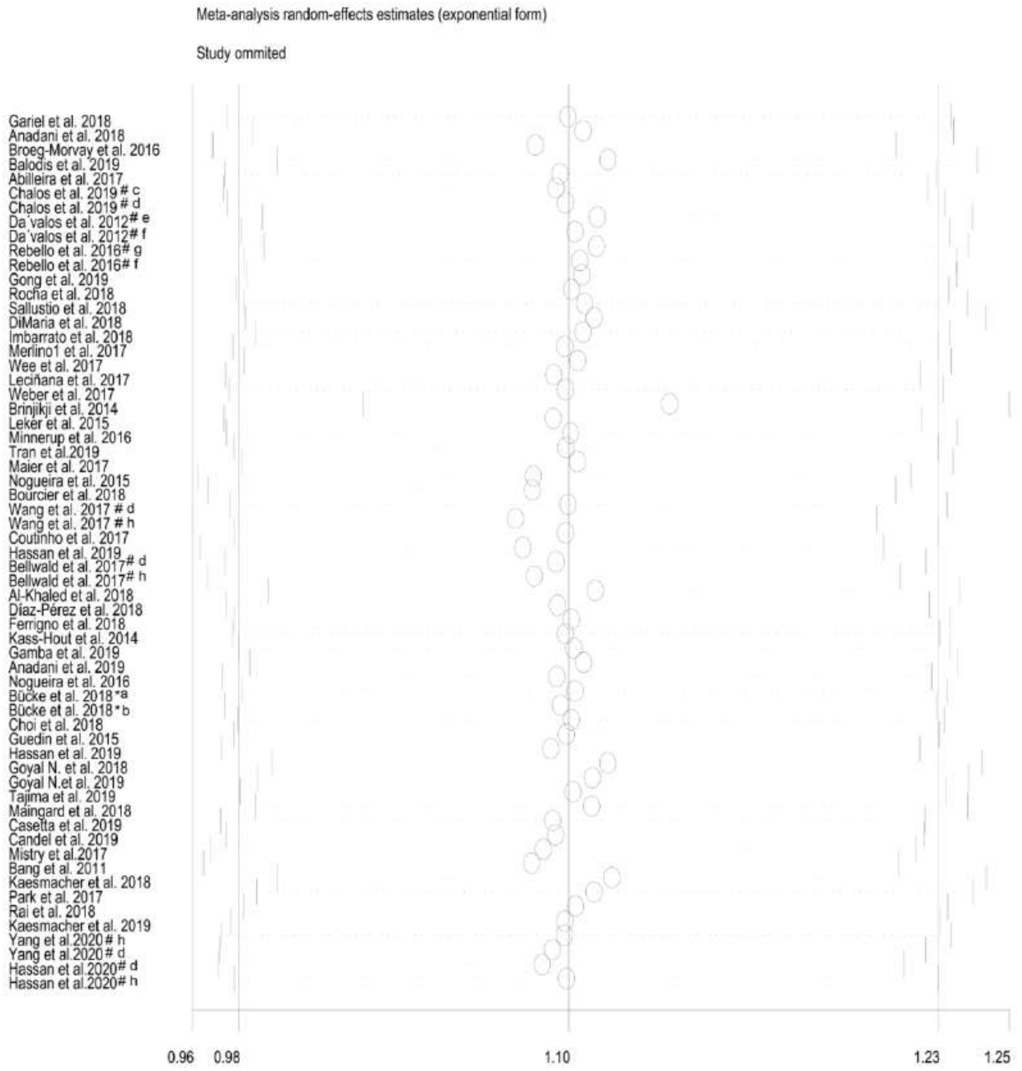

Meta-analysis random-effects estimates (exponential form)
Study ommited
Gariel et al. 2018
Anadani et al. 2018
Broeg-Morvay et al. 2016
Balodis et al. 2019
Abilleira et al. 2017
Chalos et al. 2019 # c
Chalos et al. 2019 # d
Da`valos et al. 2012# e
Da`valos et al. 2012# f
Rebello et al. 2016# g
Rebello et al. 2016# f
Gong et al. 2019
Rocha et al. 2018
Sallustio et al. 2018
DiMaria et al. 2018
Imbarrato et al. 2018
Merlino1 et al. 2017
Wee et al. 2017
Leciñana et al. 2017
Weber et al. 2017
Brinjikji et al. 2014
Leker et al. 2015
Minnerup et al. 2016
Tran et al.2019
Maier et al. 2017
Nogueira et al. 2015
Bourcier et al. 2018
Wang et al. 2017 # d
Wang et al. 2017 # h
Coutinho et al. 2017
Hassan et al. 2019
Bellwald et al. 2017# d
Bellwald et al. 2017# h
Al-Khaled et al. 2018
Díaz-Pérez et al. 2018
Ferrigno et al. 2018
Kass-Hout et al. 2014
Gamba et al. 2019
Anadani et al. 2019
Nogueira et al. 2016
Bücke et al. 2018 *a
Bücke et al. 2018 *b
Choi et al. 2018
Guedin et al. 2015
Hassan et al. 2019
Goyal N. et al. 2018
Goyal N.et al. 2019
Tajima et al. 2019
Maingard et al. 2018
Casetta et al. 2019
Candel et al. 2019
Mistry et al.2017
Bang et al. 2011
Kaesmacher et al. 2018
Park et al. 2017
Rai et al. 2018
Kaesmacher et al. 2019
Yang et al.2020# h
Yang et al.2020# d
Hassan et al.2020# d
Hassan et al.2020# h
0.96
0.98
1.10
1.23
1.25

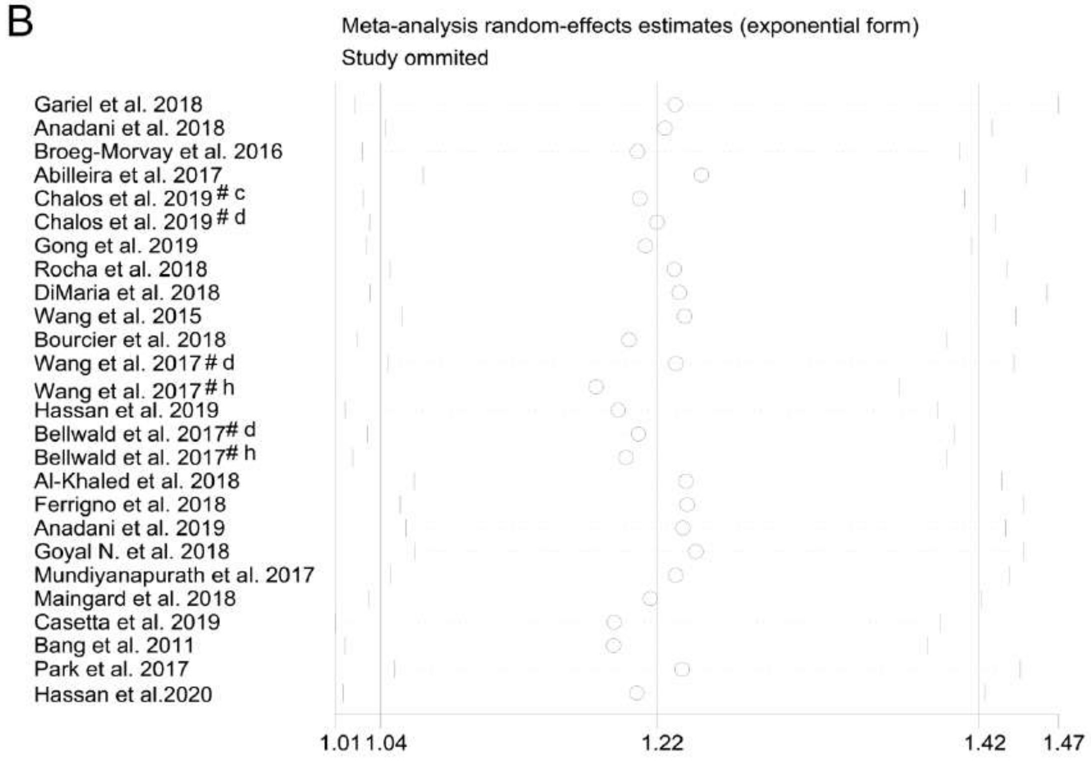
B
Meta-analysis random-effects estimates (exponential form)
Study ommited
Gariel et al. 2018
Anadani et al. 2018
Broeg-Morvay et al. 2016
Abilleira et al. 2017
Chalos et al. 2019 # c
Chalos et al. 2019 # d
Gong et al. 2019
Rocha et al. 2018
DiMaria et al. 2018
Wang et al. 2015
Bourcier et al. 2018
Wang et al. 2017 # d
Wang et al. 2017 # h
Hassan et al. 2019
Bellwald et al. 2017 # d
Bellwald et al. 2017 # h
Al-Khaled et al. 2018
Ferrigno et al. 2018
Anadani et al. 2019
Goyal N. et al. 2018
Mundiyanapurath et al. 2017
Maingard et al. 2018
Casetta et al. 2019
Bang et al. 2011
Park et al. 2017
Hassan et al.2020
1.01
1.04
1.22
1.42
1.47

**Supplemental Figure 107.** Sensitivity analysis of the effect of individual studies on the cOR for SAH (A) and HI (B). cOR, crude odds ratio; SAH, subarachnoid hemorrhage; HI, hemorrhagic infarction. #The outcomes of HI-1 (a) and HI-2 (b) were investigated respectively in the studies of Coutinho et al. 2017, Sallustio et al. 2018, Leciñana et al. 2017, Dávalos et al. 2012 and Mulder et al. 2016.

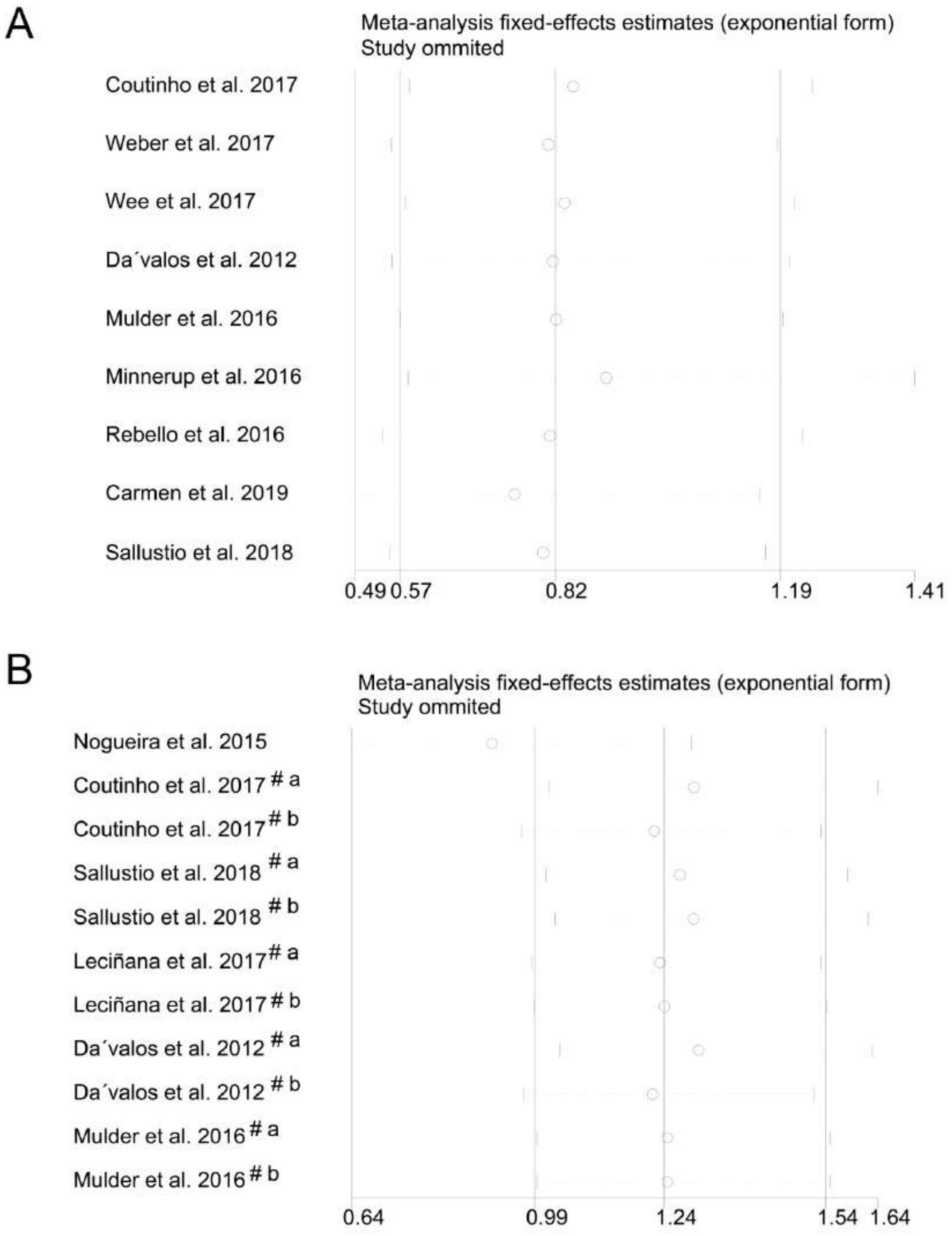

**Supplemental Figure 108.** Sensitivity analysis of the effect of individual studies on the cOR (A) and aOR (B) for PH. cOR, crude odds ratio; aOR, adjusted odds ratio; PH, parenchymal hematoma. [#]The outcomes of PH-1(a) and PH-2 (b) were investigated respectively in the studies of Leciñana et al. 2017, Casetta et al. 2019, Sallustio et al. 2018 and Da ́valos et al. 2012.

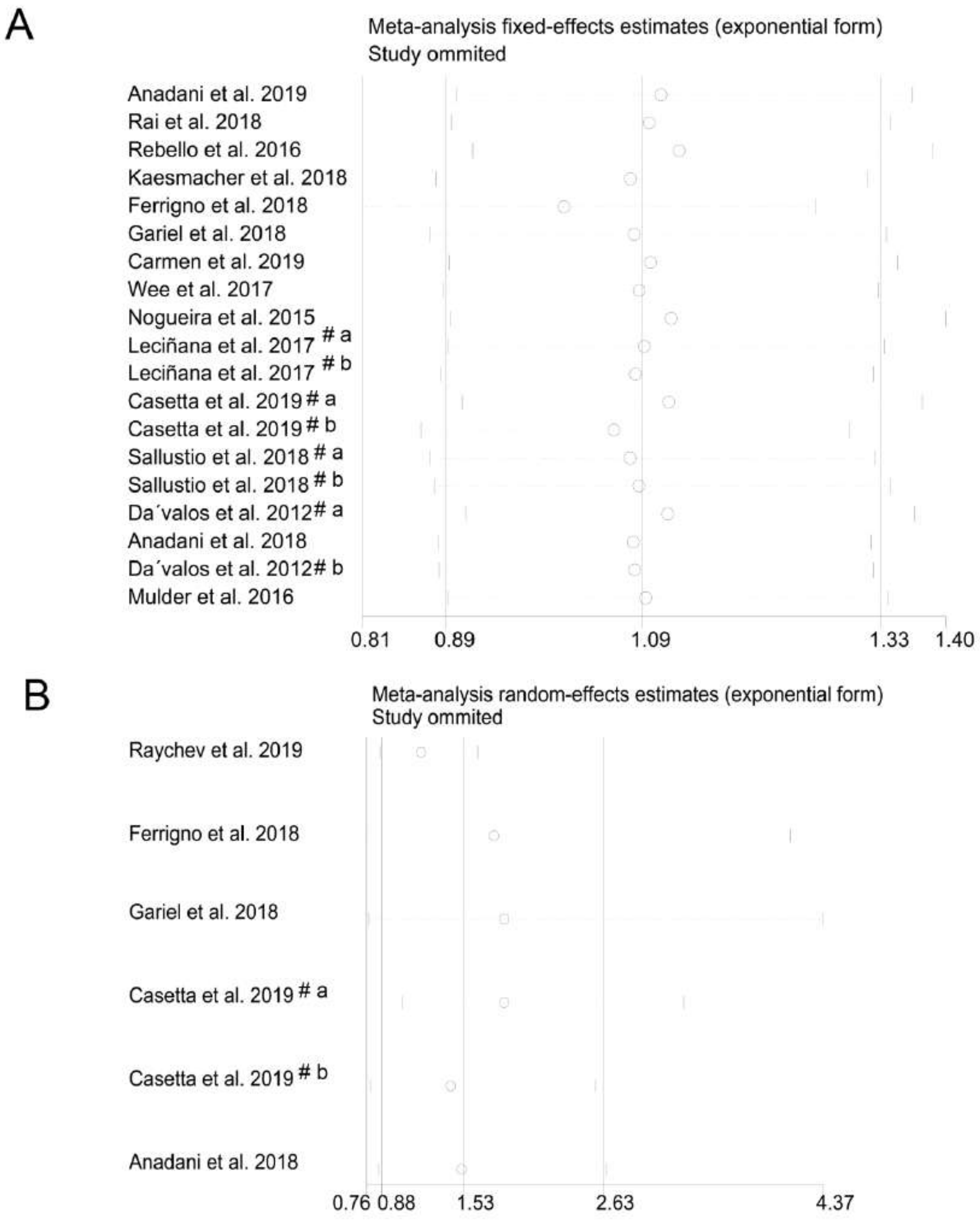

**Supplemental Figure 109.** Sensitivity analysis of the effect of individual studies on the cOR for dramatic improvement at discharge/7days (A), early recovery at 24h (B) and good improvement at discharge/7 days (C). cOR, crude odds ratio.

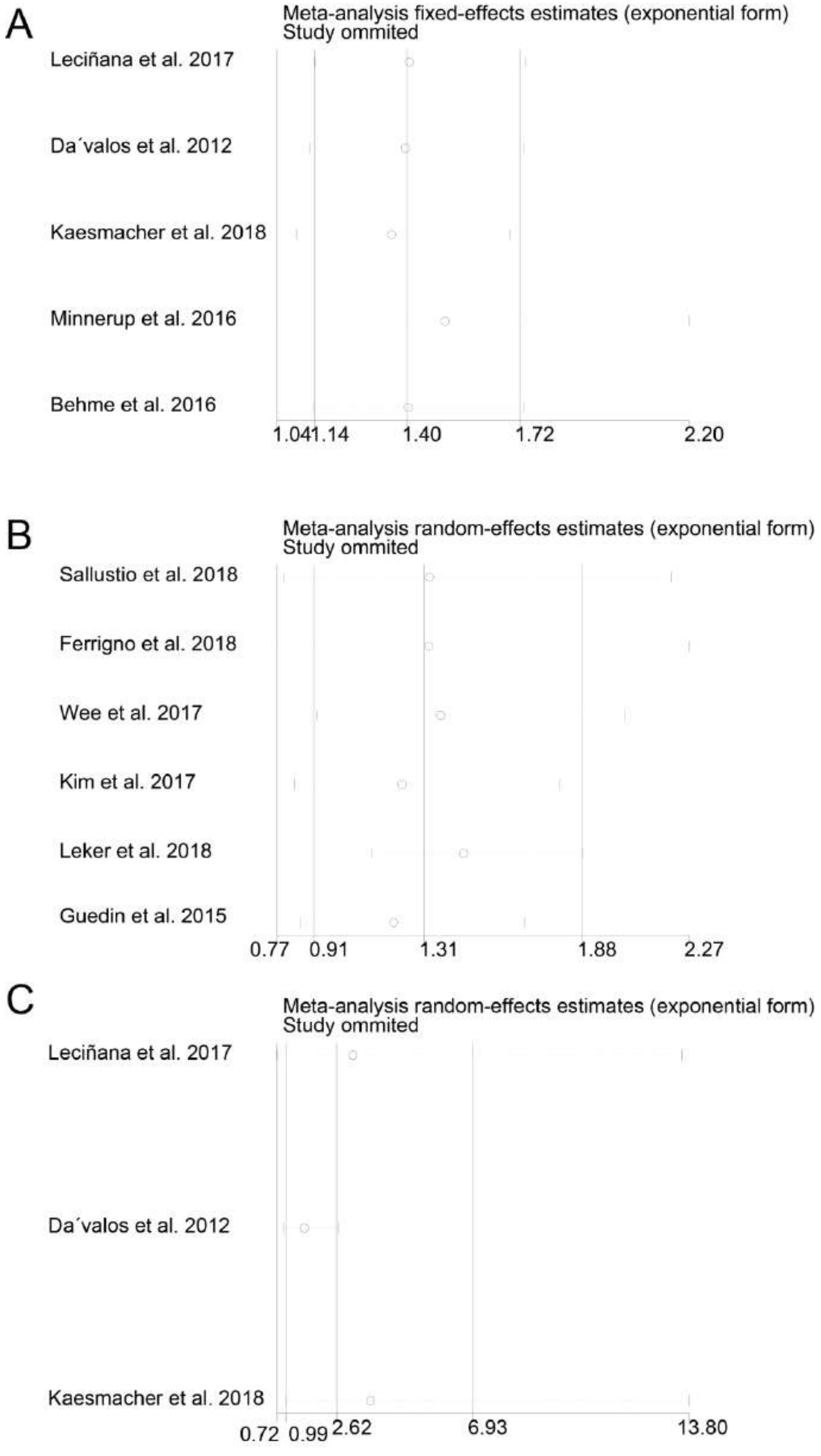

**Supplemental Figure 110.** Sensitivity analysis of the effect of individual studies on the cOR (A) and aOR (B) for passes number of thrombectomy device⩽2, and good the cOR for passes number of thrombectomy device=1 (C). cOR, crude odds ratio; aOR, adjusted odds ratio.

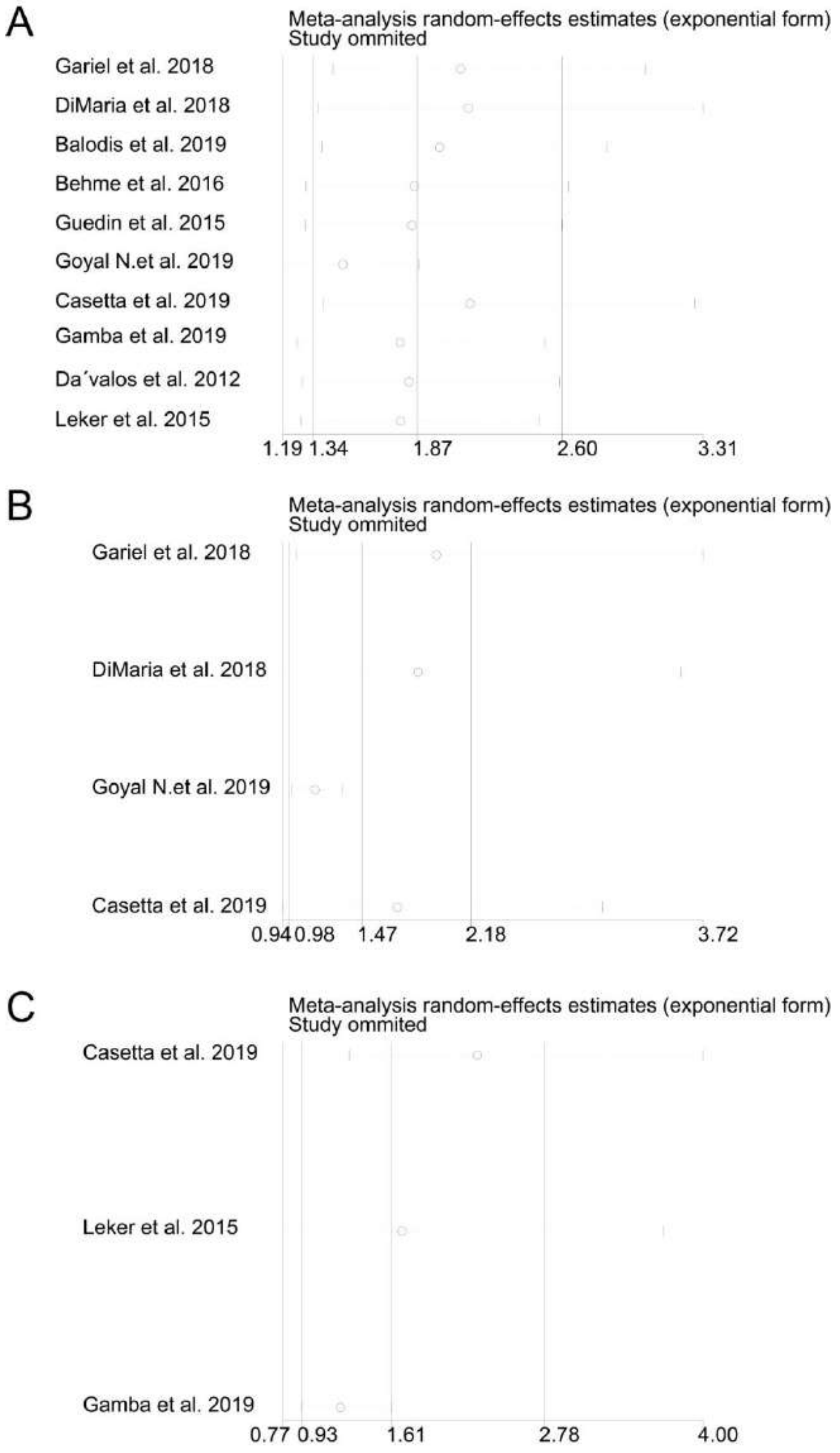

**Supplemental Figure 111.** Sensitivity analysis of the effect of individual studies on the cOR (A) and aOR (B) for any complications. cOR, crude odds ratio; aOR, adjusted odds ratio. [#]The outcomes of procedural complications (a), rescue therapy (b) and clot migration (c) were investigated respectively in the study of Gariel et al. 2018; The outcomes of vasospasm (d), vessel perforation (e), clot migration (c) and rescue therapy (b) were investigated respectively in the study of DiMaria et al. 2018; The outcomes of vessel perforation (e), arterial dissection (f), device failure (g) and clot migration (c) were investigated respectively in the study of Minnerup et al. 2016; The outcomes of vessel pseudoaneurysm (h), groin haematoma (i) and clot migration (c) were investigated respectively in the study of Wee et al. 2017; The outcomes of vessel perforation (e), vasospasm (d), clot migration (c) and groin haematoma (i) were investigated respectively in the study of Coutinho et al. 2017; The outcomes of groin haematoma (i) and clot migration (c) were investigated respectively in the study of Weber et al. 2017; The outcomes of arterial dissection (f) and clot migration (c) were investigated respectively in the study of Casetta et al. 2019;

A

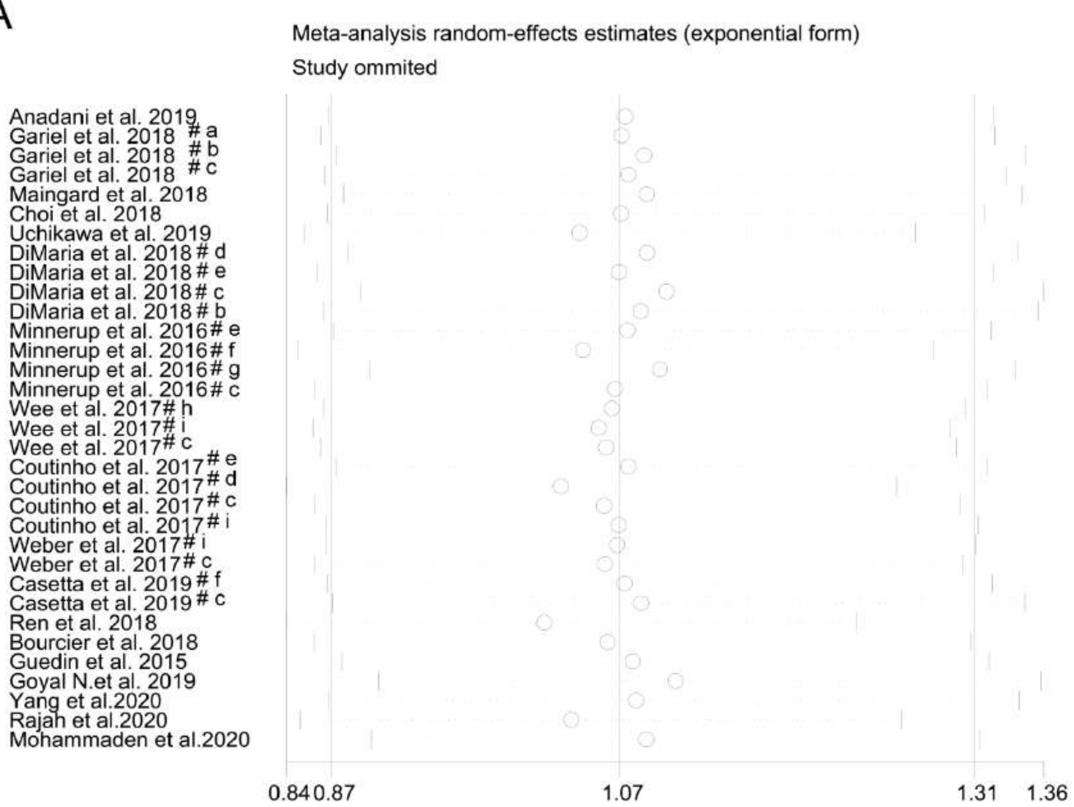

Meta-analysis random-effects estimates (exponential form)
Study ommited
Anadani et al. 2019
Gariel et al. 2018 # a
Gariel et al. 2018 # b
Gariel et al. 2018 # c
Maingard et al. 2018
Choi et al. 2018
Uchikawa et al. 2019
DiMaria et al. 2018 # d
DiMaria et al. 2018 # e
DiMaria et al. 2018 # c
DiMaria et al. 2018 # b
Minnerup et al. 2016 # e
Minnerup et al. 2016 # f
Minnerup et al. 2016 # g
Minnerup et al. 2016 # c
Wee et al. 2017 # h
Wee et al. 2017 # i
Wee et al. 2017 # c
Coutinho et al. 2017 # e
Coutinho et al. 2017 # d
Coutinho et al. 2017 # c
Coutinho et al. 2017 # i
Weber et al. 2017 # i
Weber et al. 2017 # c
Casetta et al. 2019 # f
Casetta et al. 2019 # c
Ren et al. 2018
Bourcier et al. 2018
Guedin et al. 2015
Goyal N.et al. 2019
Yang et al.2020
Rajah et al.2020
Mohammaden et al.2020
0.84
0.87
1.07
1.31
1.36


B

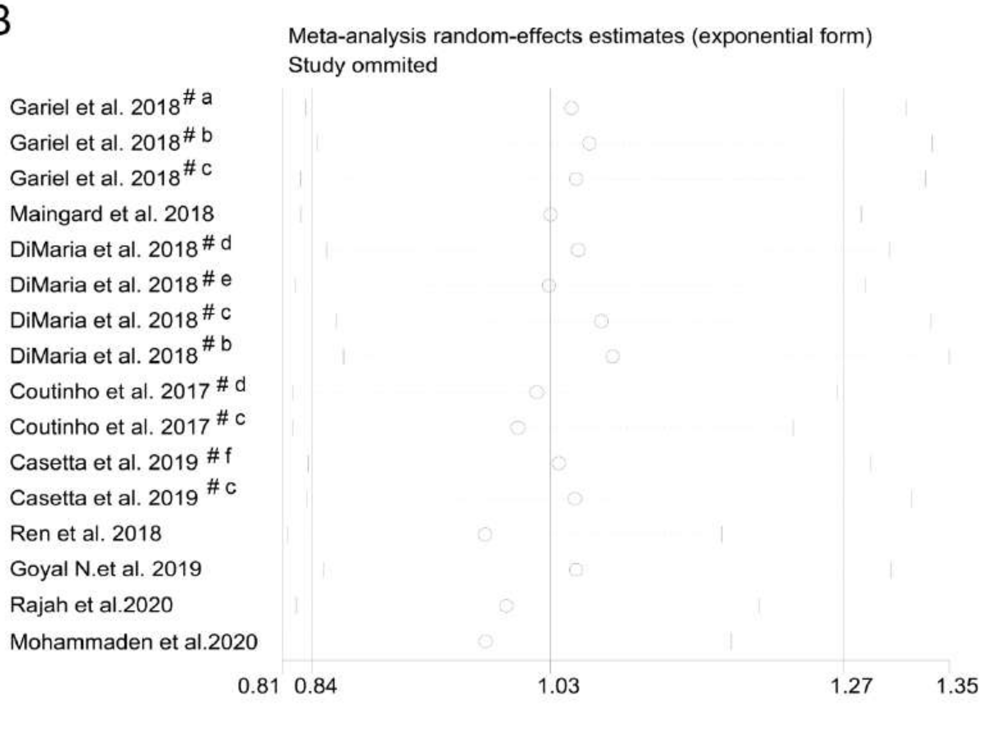

Meta-analysis random-effects estimates (exponential form)
Study ommited
Gariel et al. 2018 # a
Gariel et al. 2018 # b
Gariel et al. 2018 # c
Maingard et al. 2018
DiMaria et al. 2018 # d
DiMaria et al. 2018 # e
DiMaria et al. 2018 # c
DiMaria et al. 2018 # b
Coutinho et al. 2017 # d
Coutinho et al. 2017 # c
Casetta et al. 2019 # f
Casetta et al. 2019 # c
Ren et al. 2018
Goyal N.et al. 2019
Rajah et al.2020
Mohammaden et al.2020
0.81
0.84
1.03
1.27
1.35

**Supplemental Figure 112.** Sensitivity analysis of the effect of individual studies on the cOR (A) and aOR (B) for clot migration. cOR, crude odds ratio; aOR, adjusted odds ratio.

A

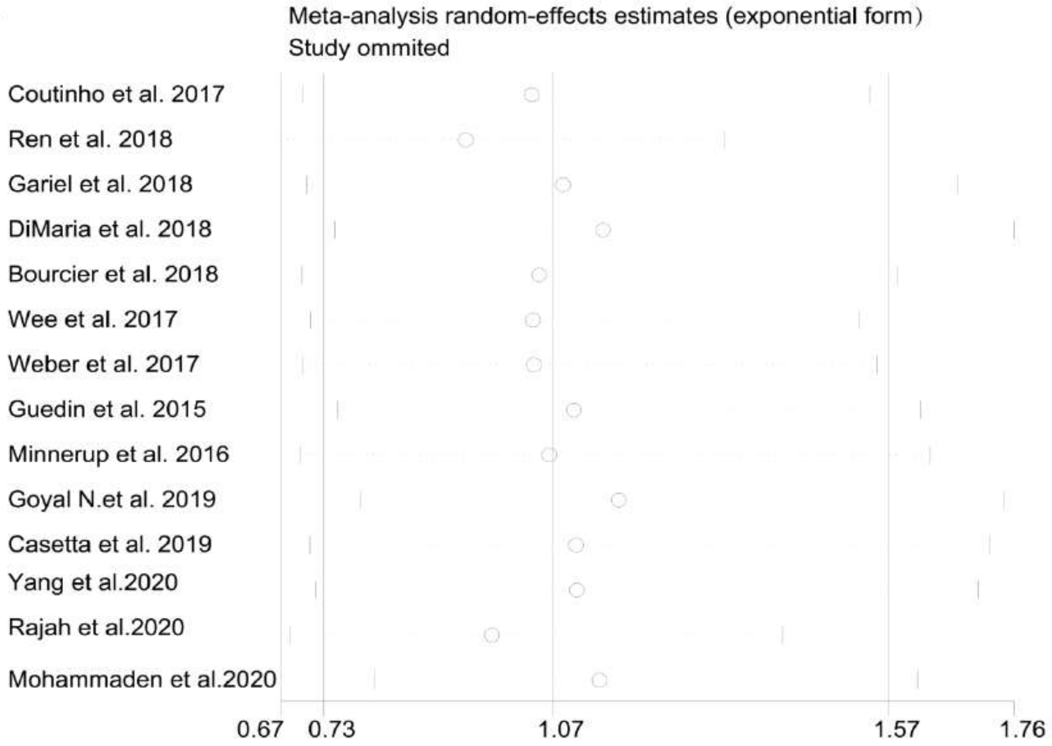


B

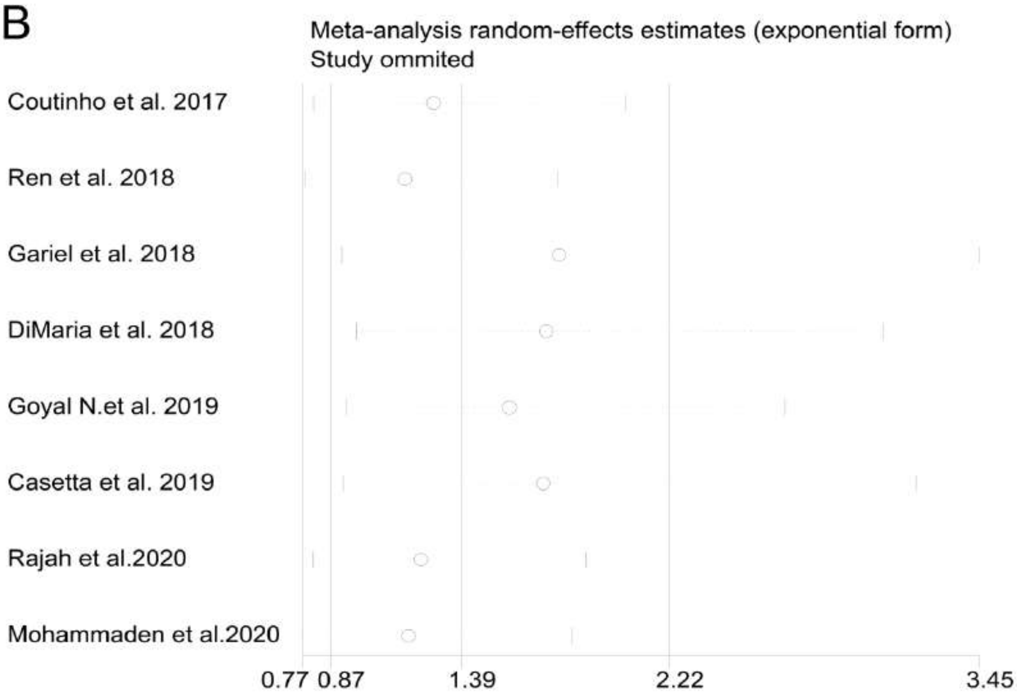

**Supplemental Figure 113.** Sensitivity analysis of the effect of individual studies on the cOR for groin hematoma (A) and recurrent stroke (B). cOR, crude odds ratio.

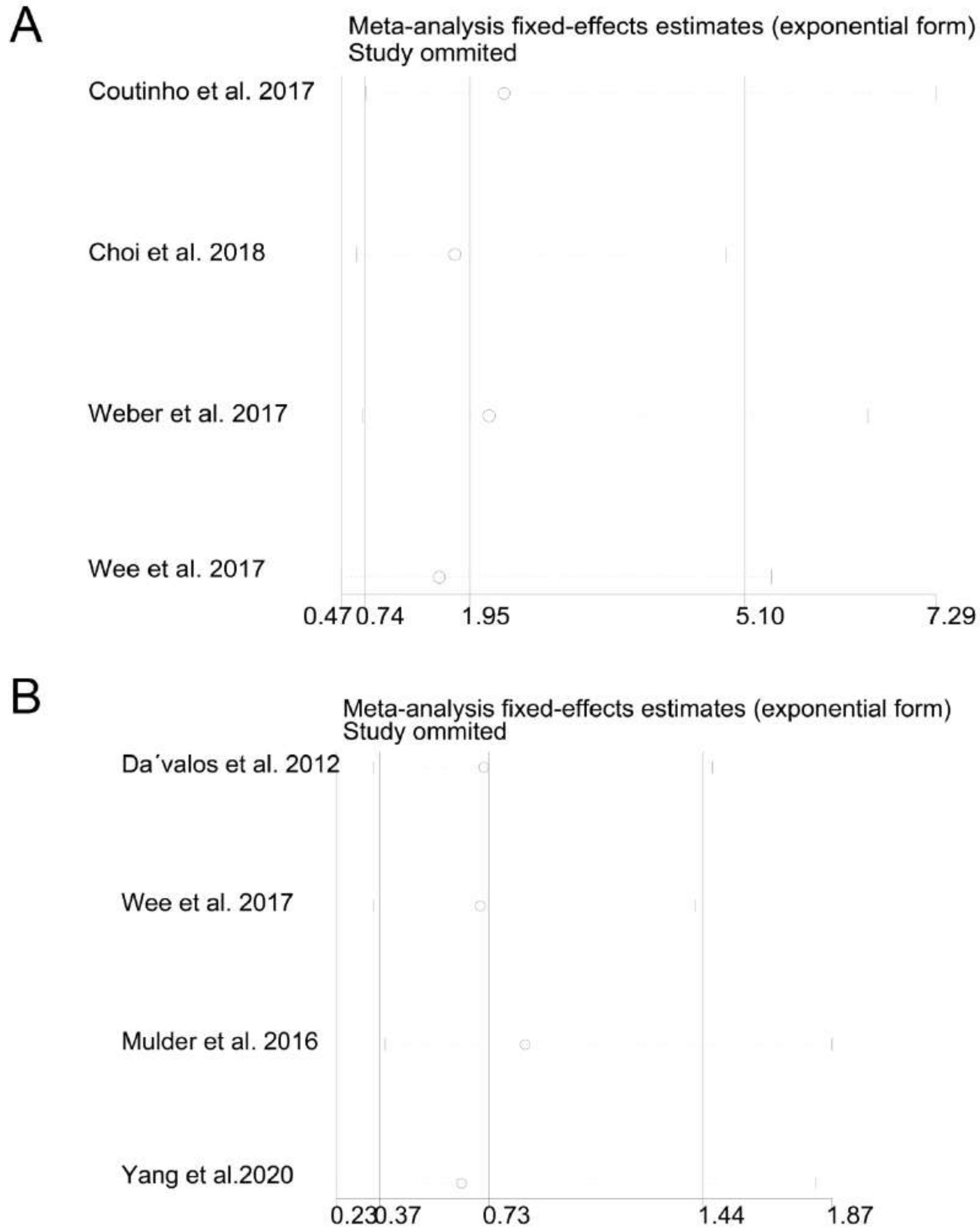

**Supplemental Figure 114.** Sensitivity analysis of the effect of individual studies on the cOR for vasospasm. cOR, crude odds ratio.

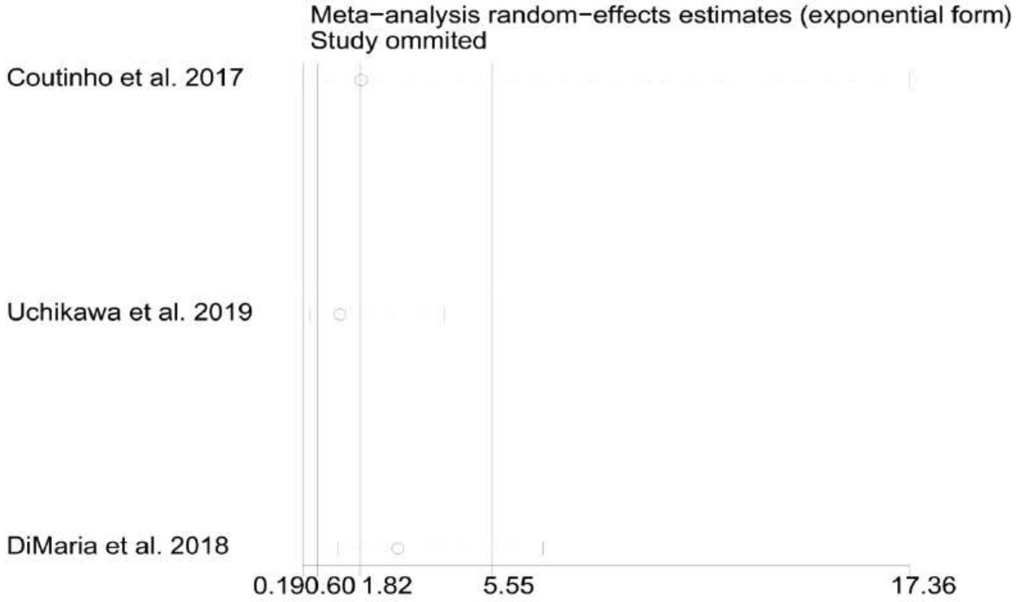

**Supplemental Figure 115.** Sensitivity analysis of the effect of individual studies on the cOR for vessel dissection (A) and vessel perforation (B). cOR, crude odds ratio.

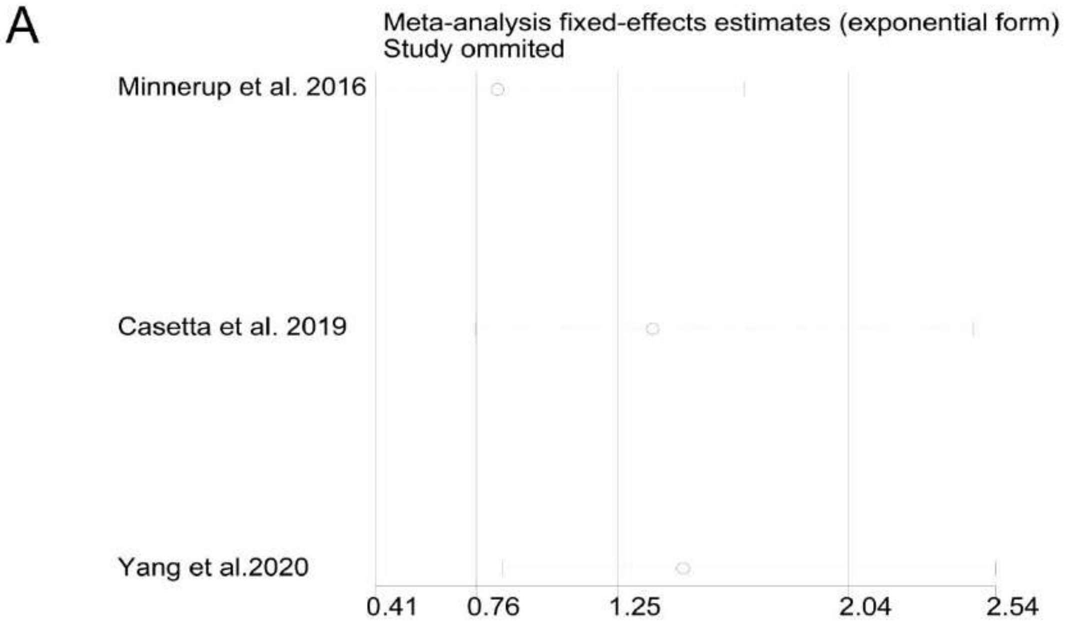


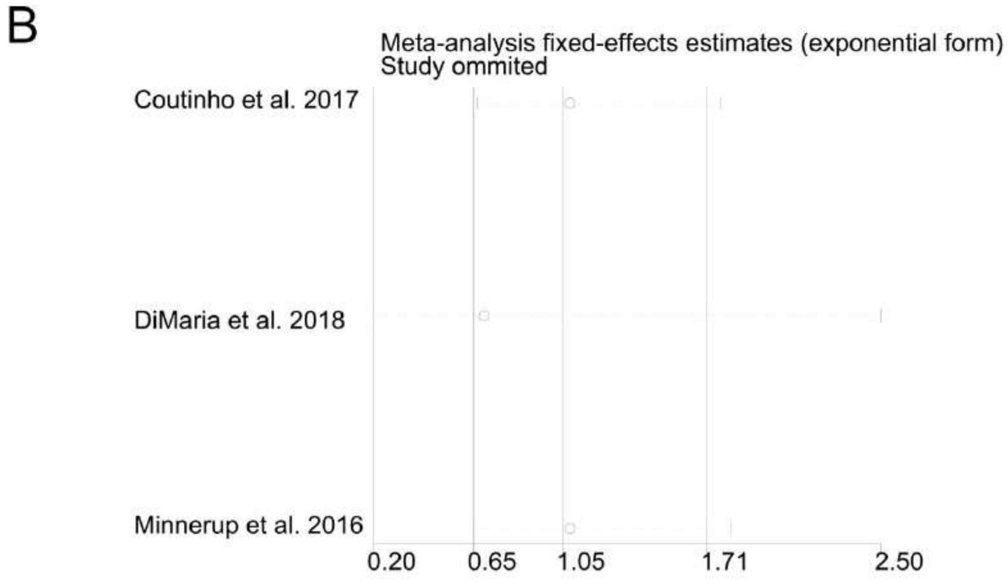

**Supplemental Figure 116.** Publication bias analysis by Begg's funnel plots of cOR (A) and aOR (B) for good outcome at 90 days, and by the "Trim and fill" funnel plots of aOR (C) for good outcome at 90 days. cOR, crude odds ratio; aOR, adjusted odds ratio.

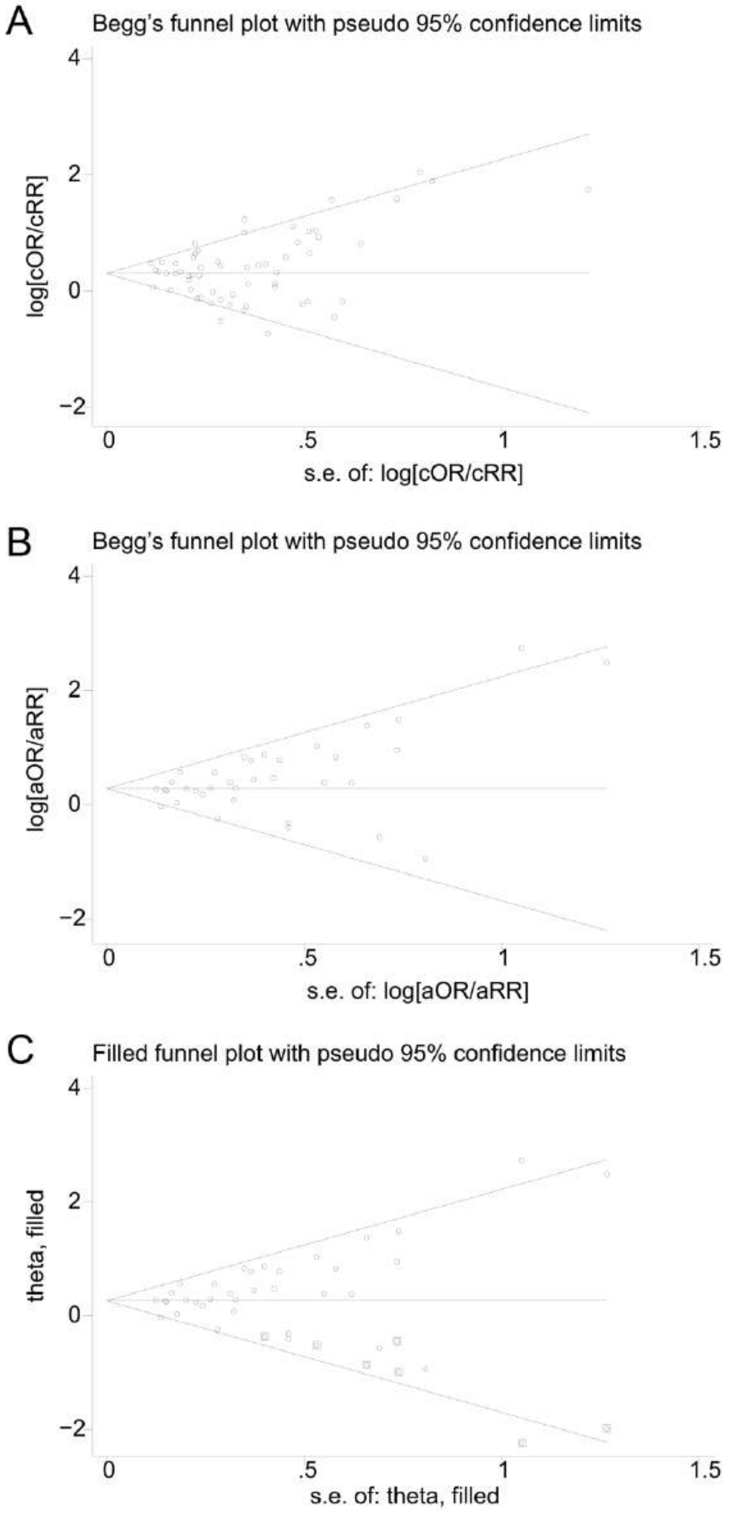

**Supplemental Figure 117.** Publication bias analysis by Begg's funnel plots of cOR (A) and aOR (B) for good outcome at discharge (vertical axis), and the standard error for cOR or aOR (horizontal axis). cOR, crude odds ratio; aOR, adjusted odds ratio.

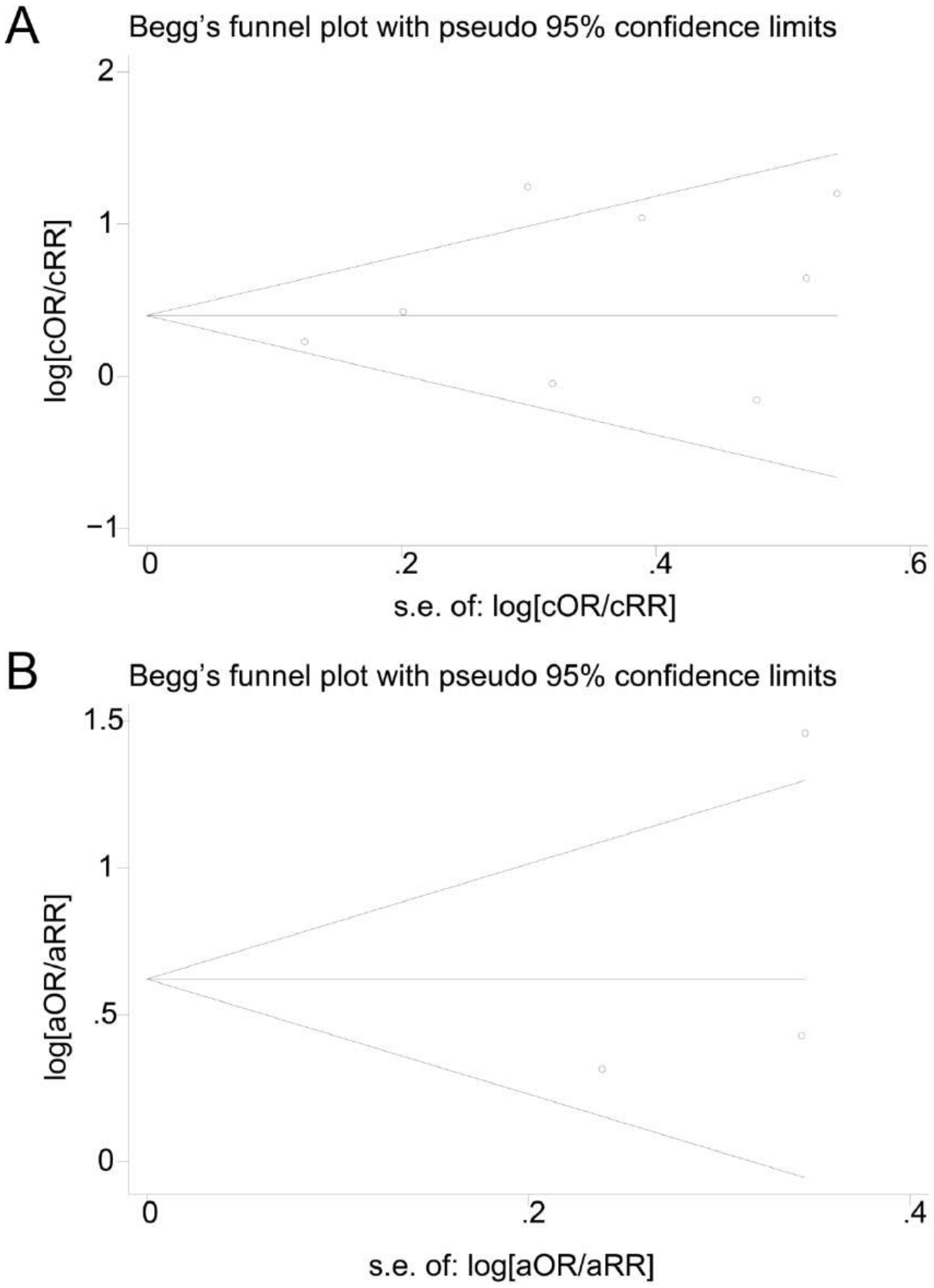

**Supplemental Figure 118.** Publication bias analysis by Begg's funnel plots of cOR (A) and aOR (B) for excellent outcome at 90 days (vertical axis), and the standard error for cOR or aOR (horizontal axis). cOR, crude odds ratio; aOR, adjusted odds ratio.

A

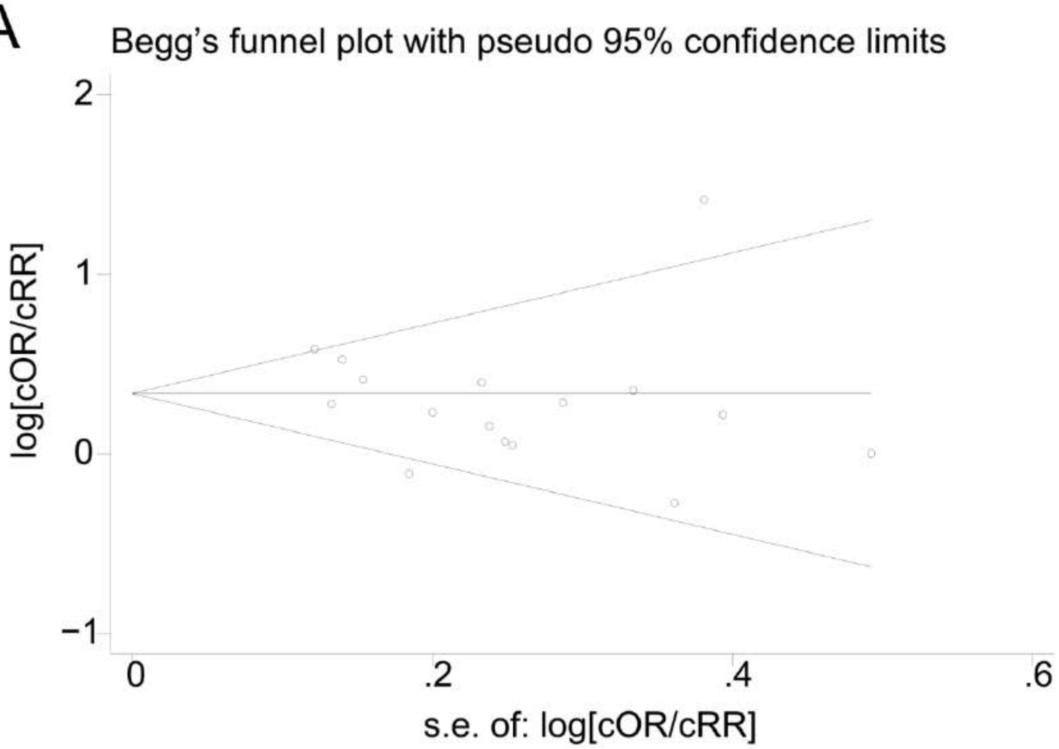


B

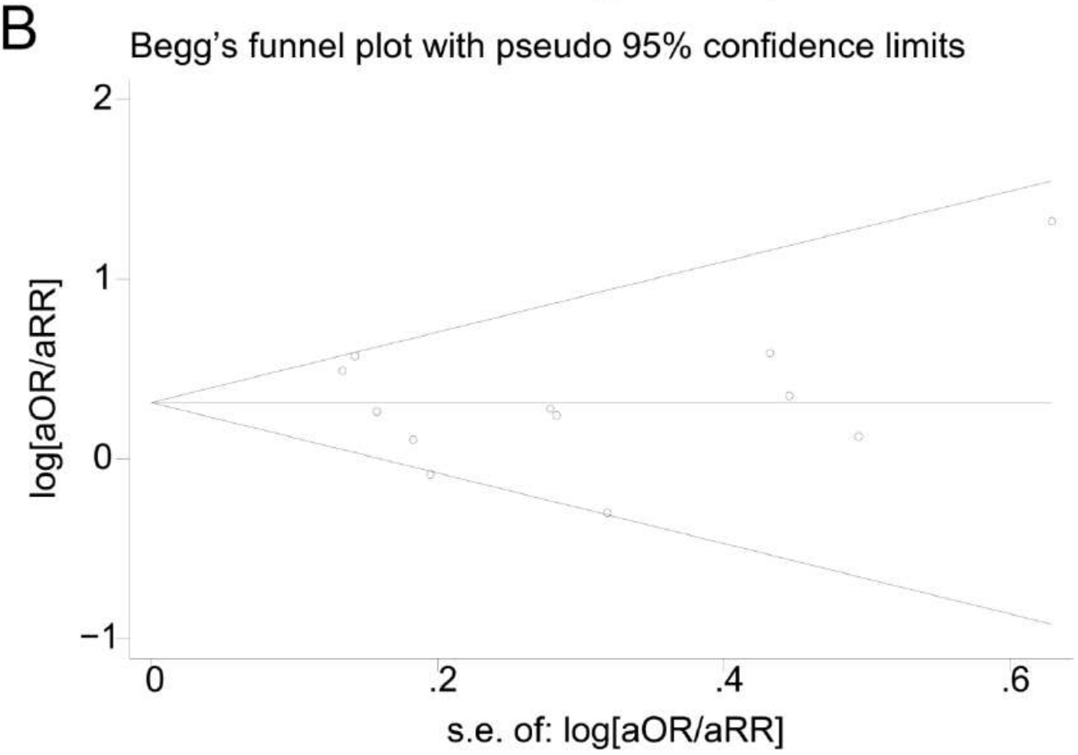

**Supplemental Figure 119.** Publication bias analysis by Begg's funnel plots of cOR (A) and aOR (B) for favorable outcome at 90 days (vertical axis), and the standard error for cOR or aOR (horizontal axis). cOR, crude odds ratio; aOR, adjusted odds ratio.

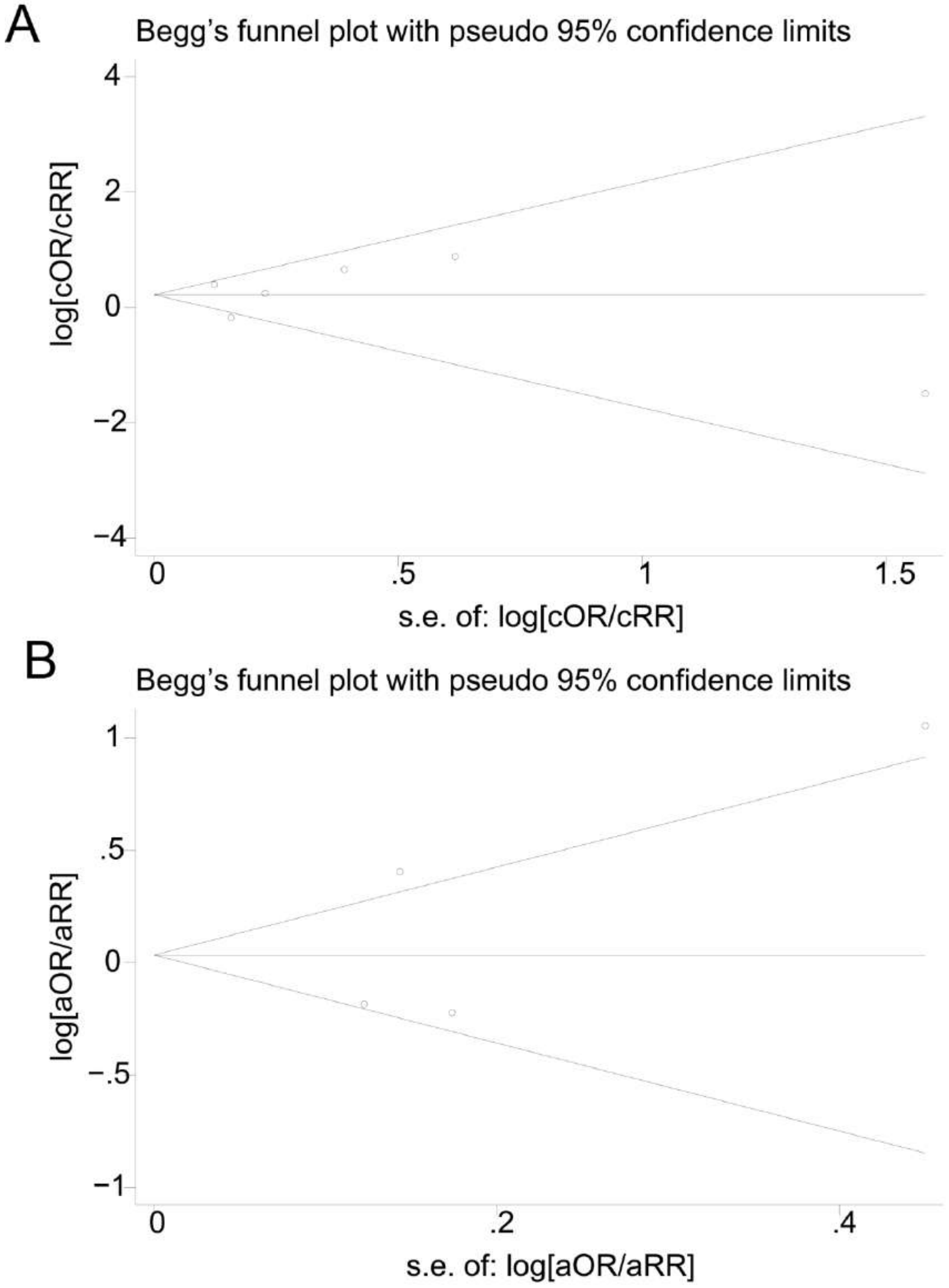

**Supplemental Figure 120.** Publication bias analysis by Begg's funnel plots of cOR (A) and aOR (B) for mortality within 90 days (vertical axis), and the standard error for cOR or aOR (horizontal axis). cOR, crude odds ratio; aOR, adjusted odds ratio.

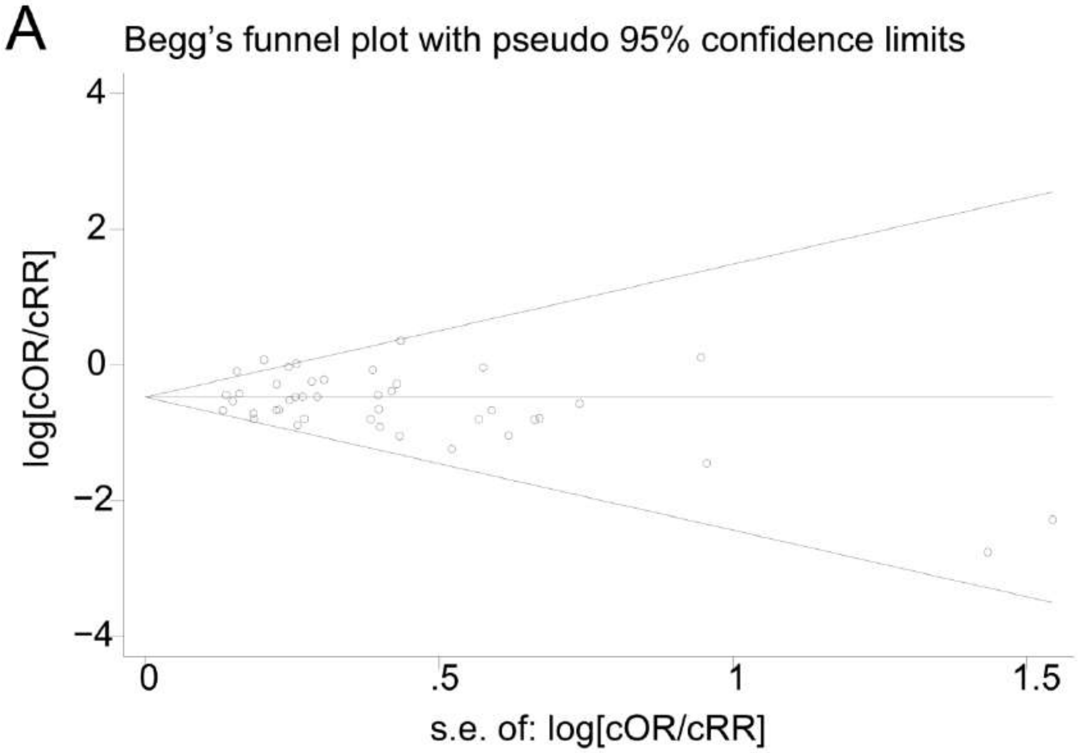


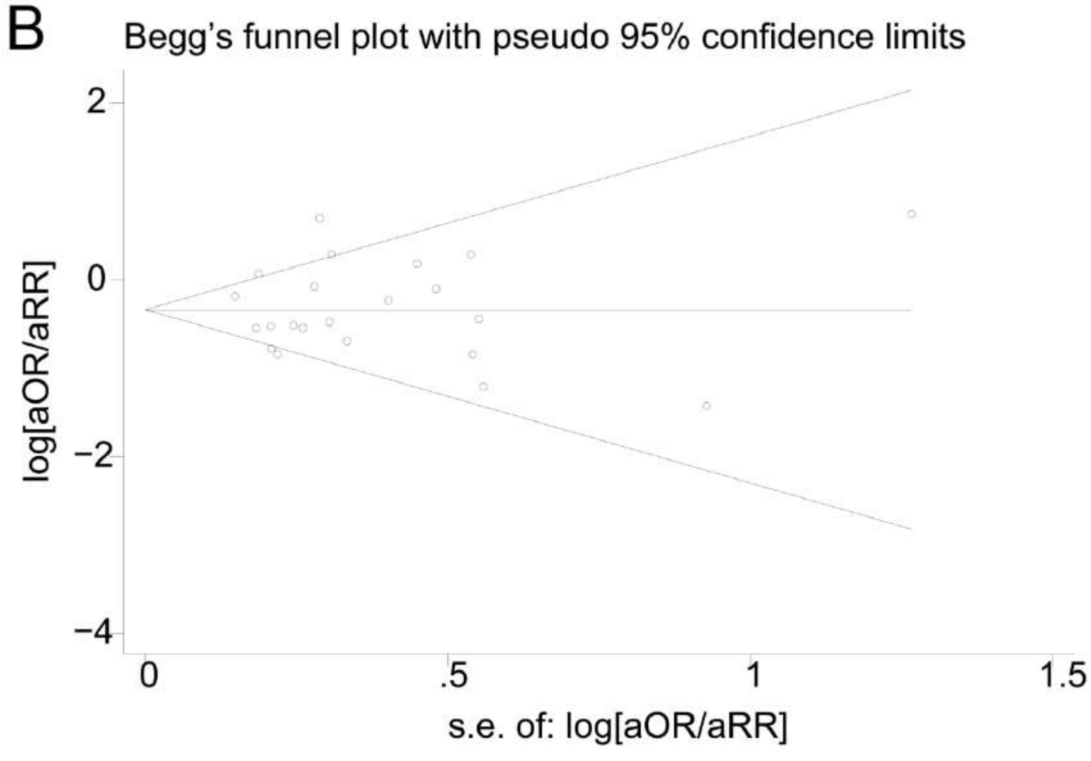

**Supplemental Figure 121.** Publication bias analysis by Begg's funnel plots of cOR (A) and aOR (B) for all mortality (vertical axis), and the standard error for cOR or aOR (horizontal axis). cOR, crude odds ratio; aOR, adjusted odds ratio.

A

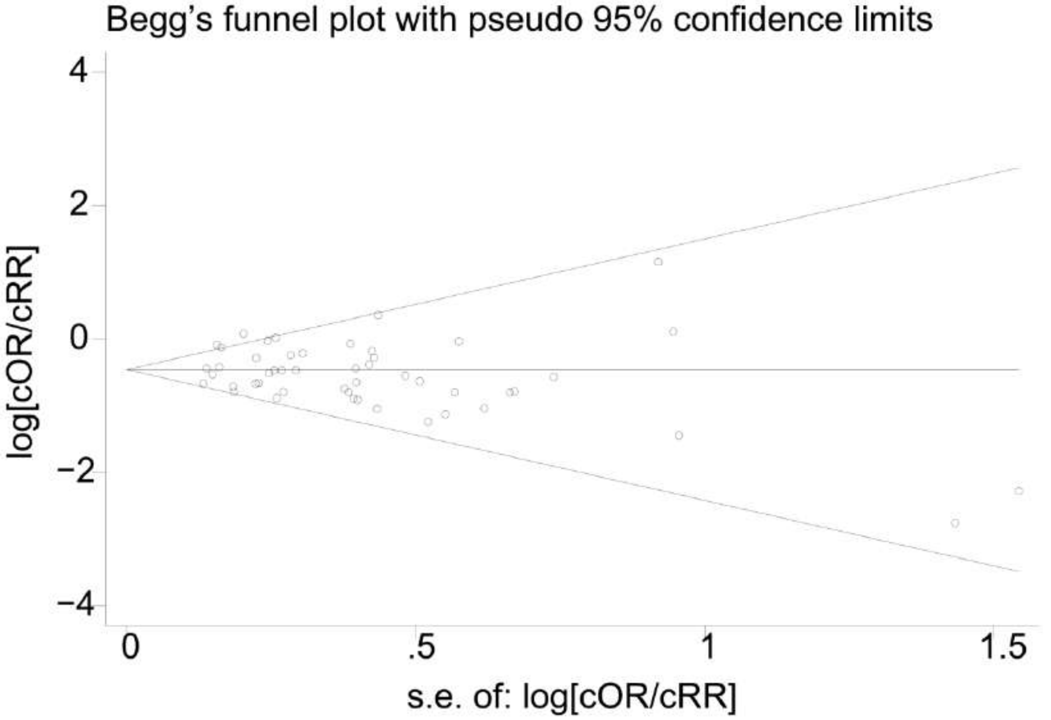


B

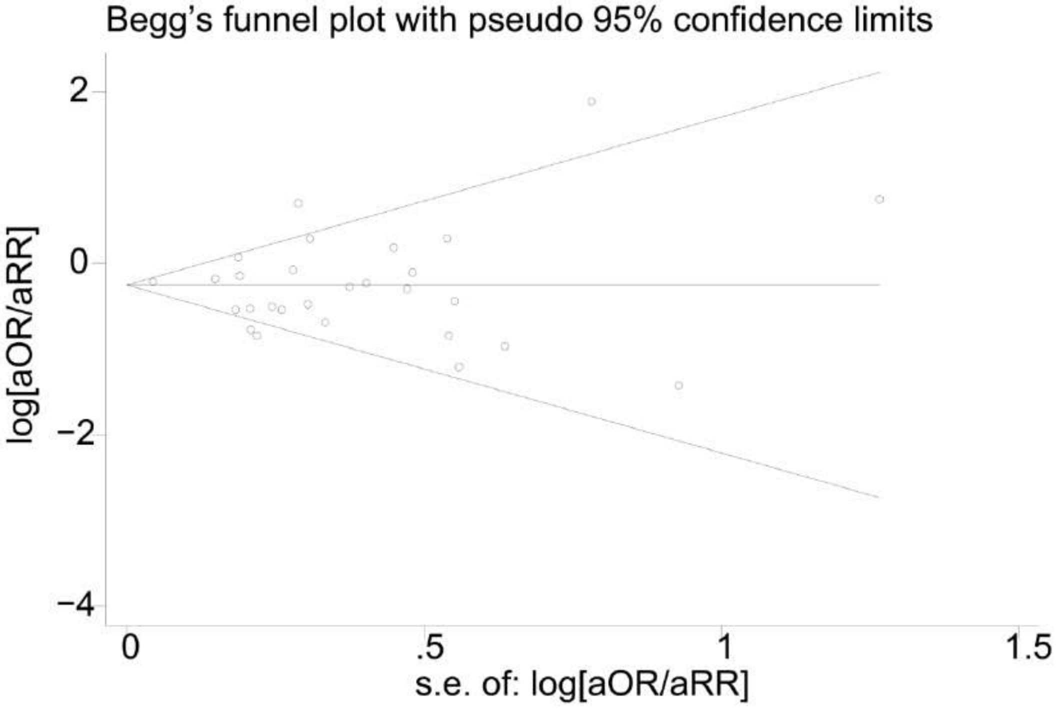

**Supplemental Figure 122.** Publication bias analysis by Begg's funnel plots of cOR (A) and aOR (B) for in-hospital mortality (vertical axis), and the standard error for cOR or aOR (horizontal axis). cOR, crude odds ratio; aOR, adjusted odds ratio.

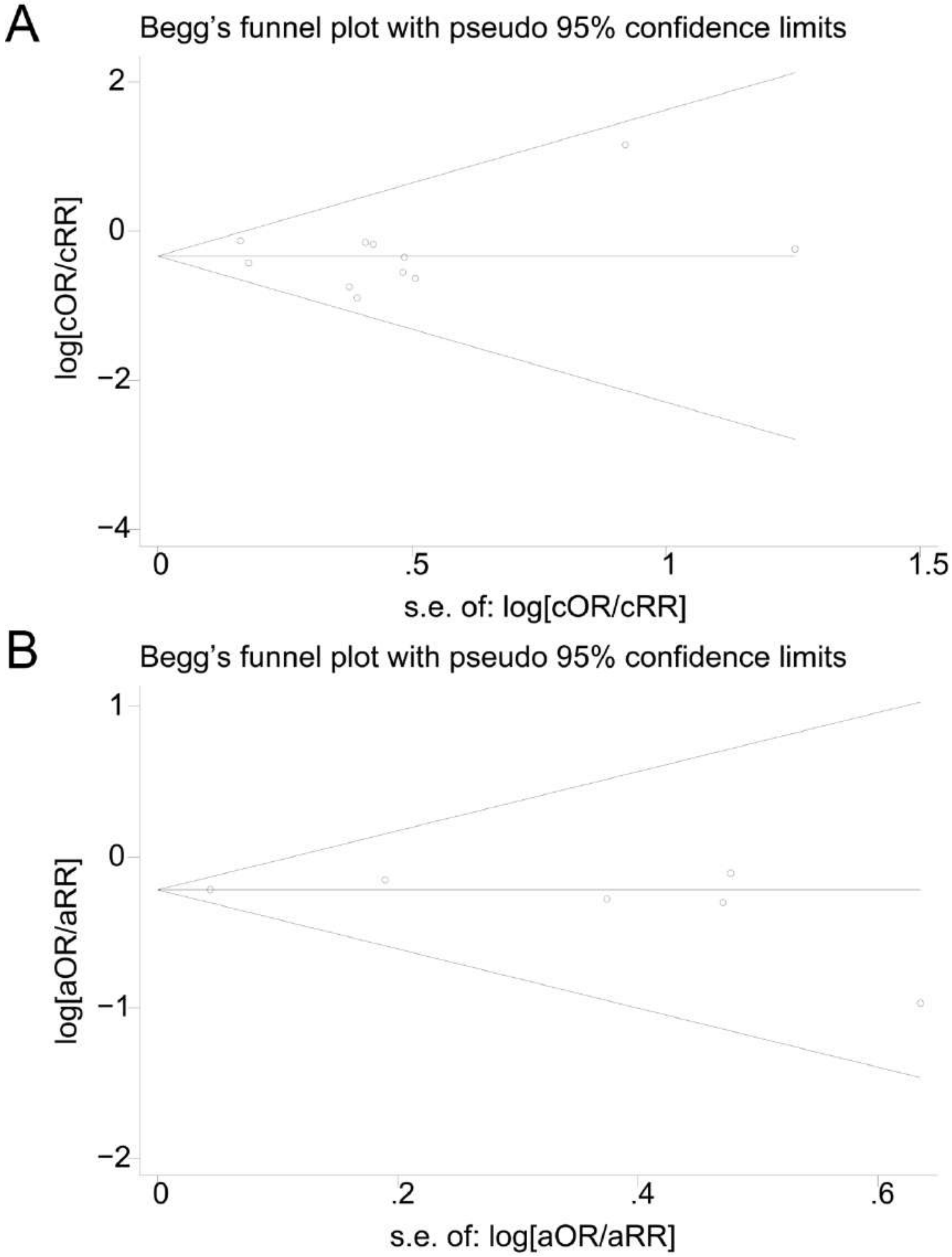

**Supplemental Figure 123.** Publication bias analysis by Begg's funnel plots of cOR (A) and aOR (B) for successful reperfusion, and by the "Trim and fill" funnel plots of aOR (C) for successful reperfusion. cOR, crude odds ratio; aOR, adjusted odds ratio.

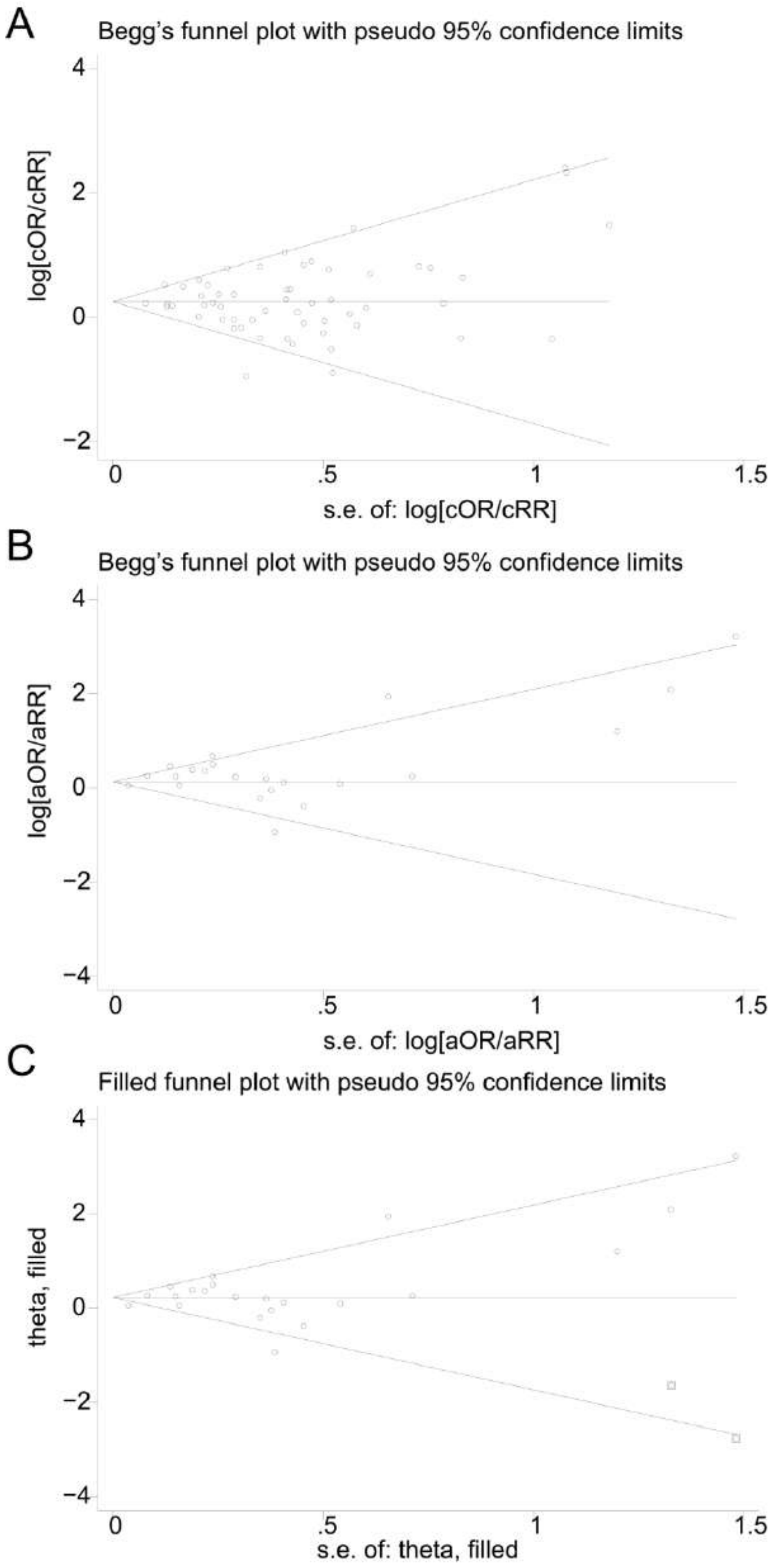

**Supplemental Figure 124.** Publication bias analysis by Begg's funnel plots of cOR (A) and aOR (B) for complete reperfusion (vertical axis), and the standard error for cOR or aOR (horizontal axis). cOR, crude odds ratio; aOR, adjusted odds ratio.

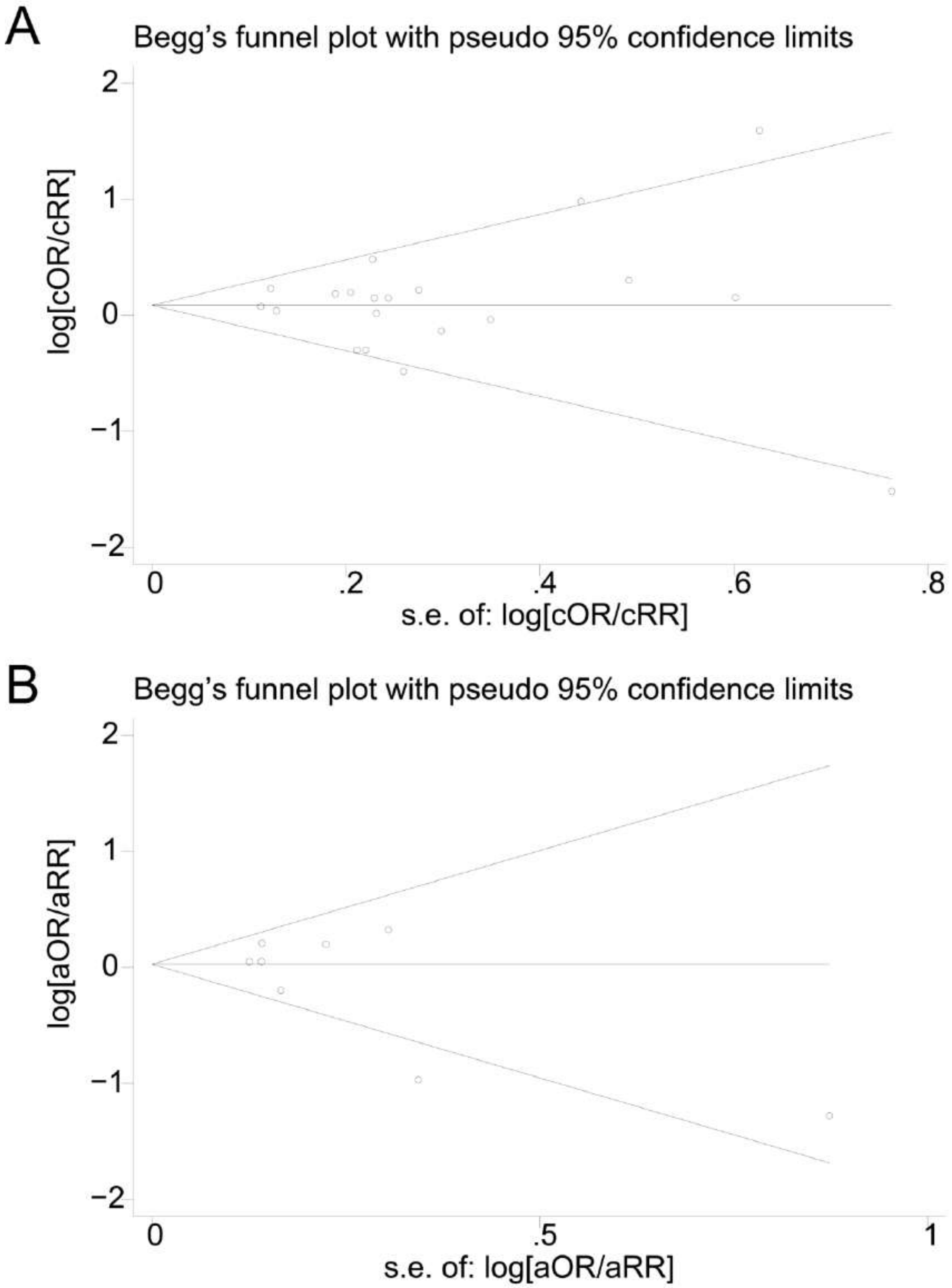

**Supplemental Figure 125.** Publication bias analysis by Begg's funnel plots of cOR (A) and aOR (B) for any ICH (vertical axis), and the standard error for cOR or aOR (horizontal axis). cOR, crude odds ratio; aOR, adjusted odds ratio; ICH, intracranial hemorrhage.

A

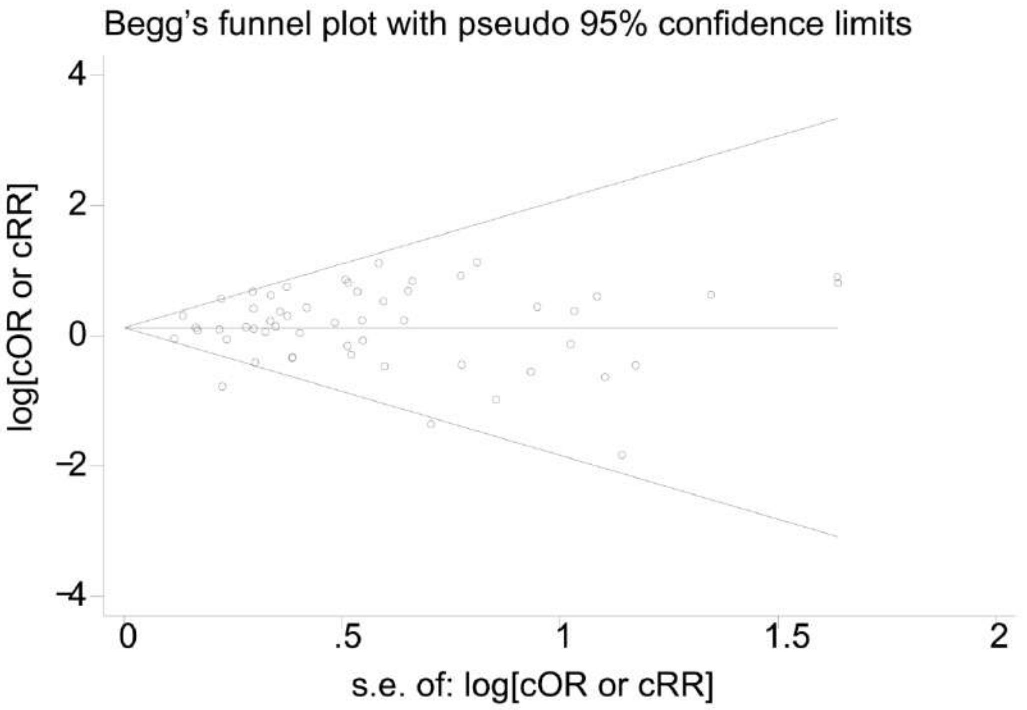


B

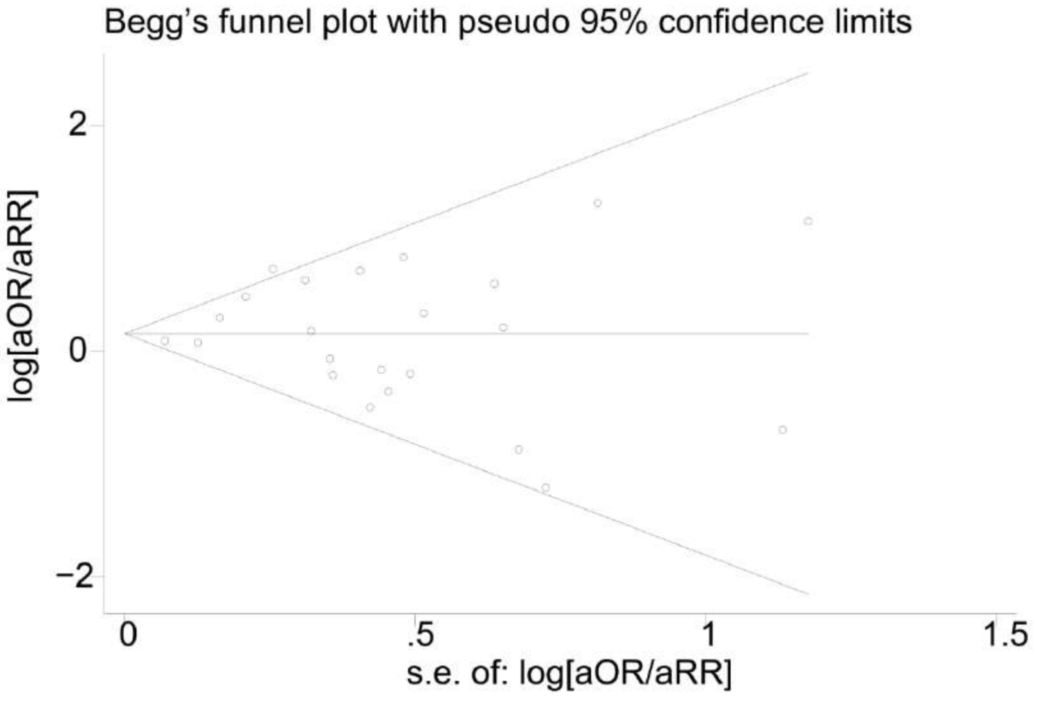

**Supplemental Figure 126.** Publication bias analysis by Begg's funnel plots of cOR (A) and aOR (B) for sICH (vertical axis), and the standard error for cOR or aOR (horizontal axis). cOR, crude odds ratio; aOR, adjusted odds ratio; sICH, symptomatic intracranial hemorrhage.

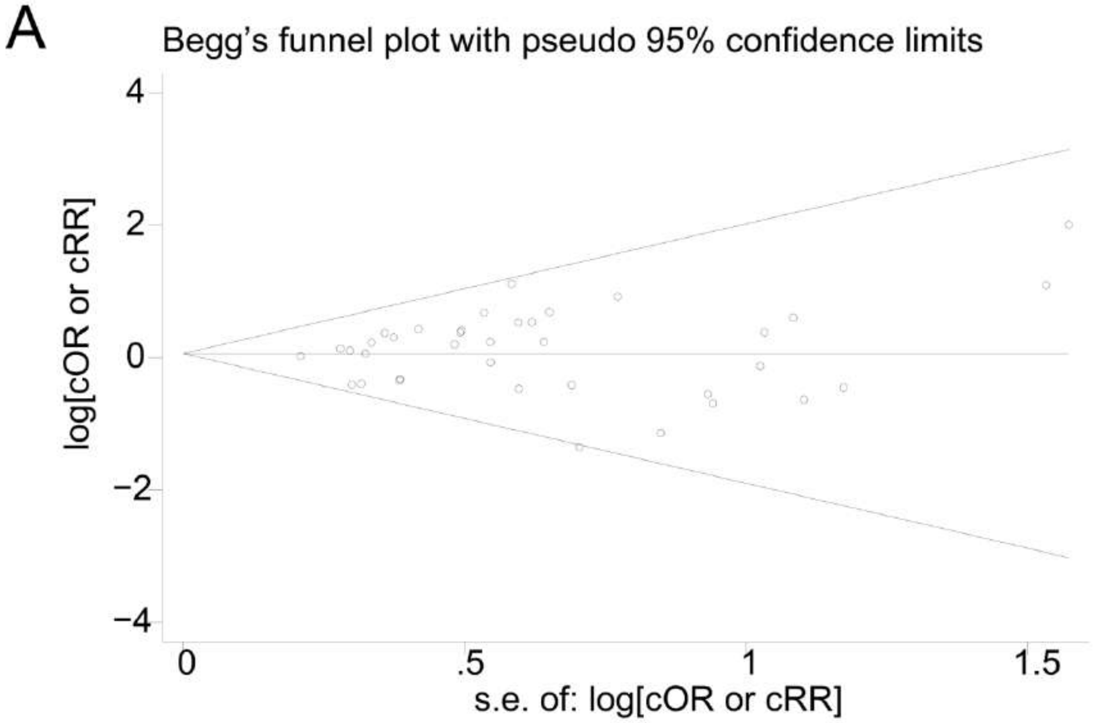


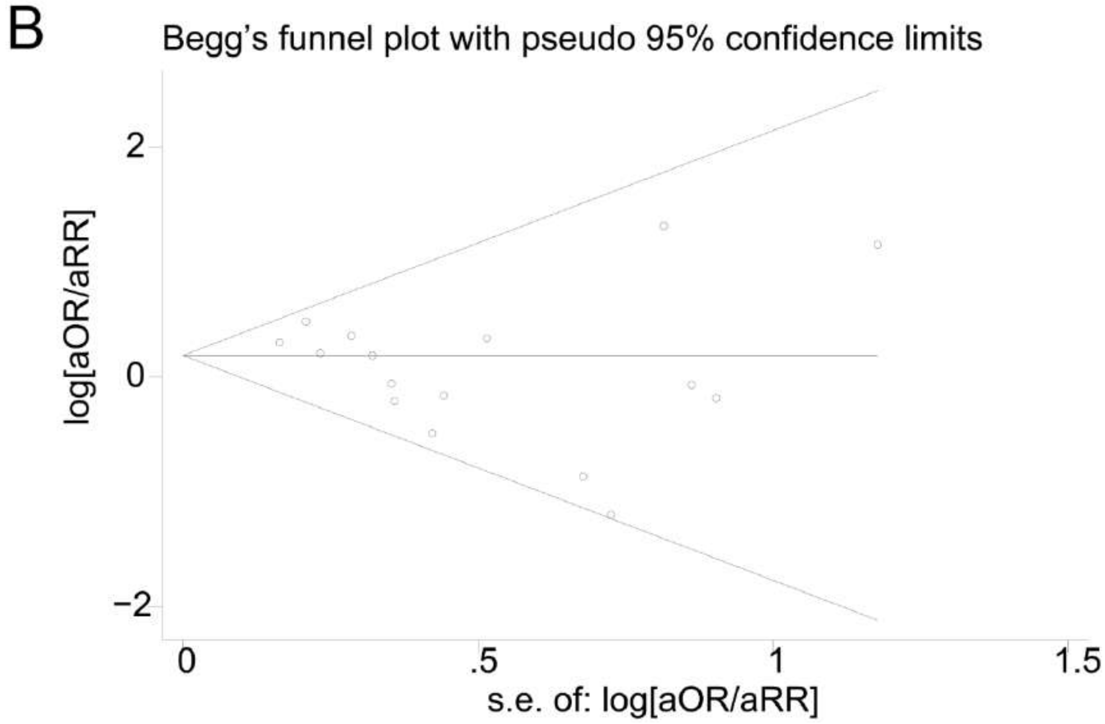

**Supplemental Figure 127.** Publication bias analysis by Begg's funnel plots of cOR (A) and aOR (B) for aICH, and by the "Trim and fill" funnel plots of cOR (C) for aICH. cOR, crude odds ratio; aOR, adjusted odds ratio; aICH, asymptomatic intracranial hemorrhage.

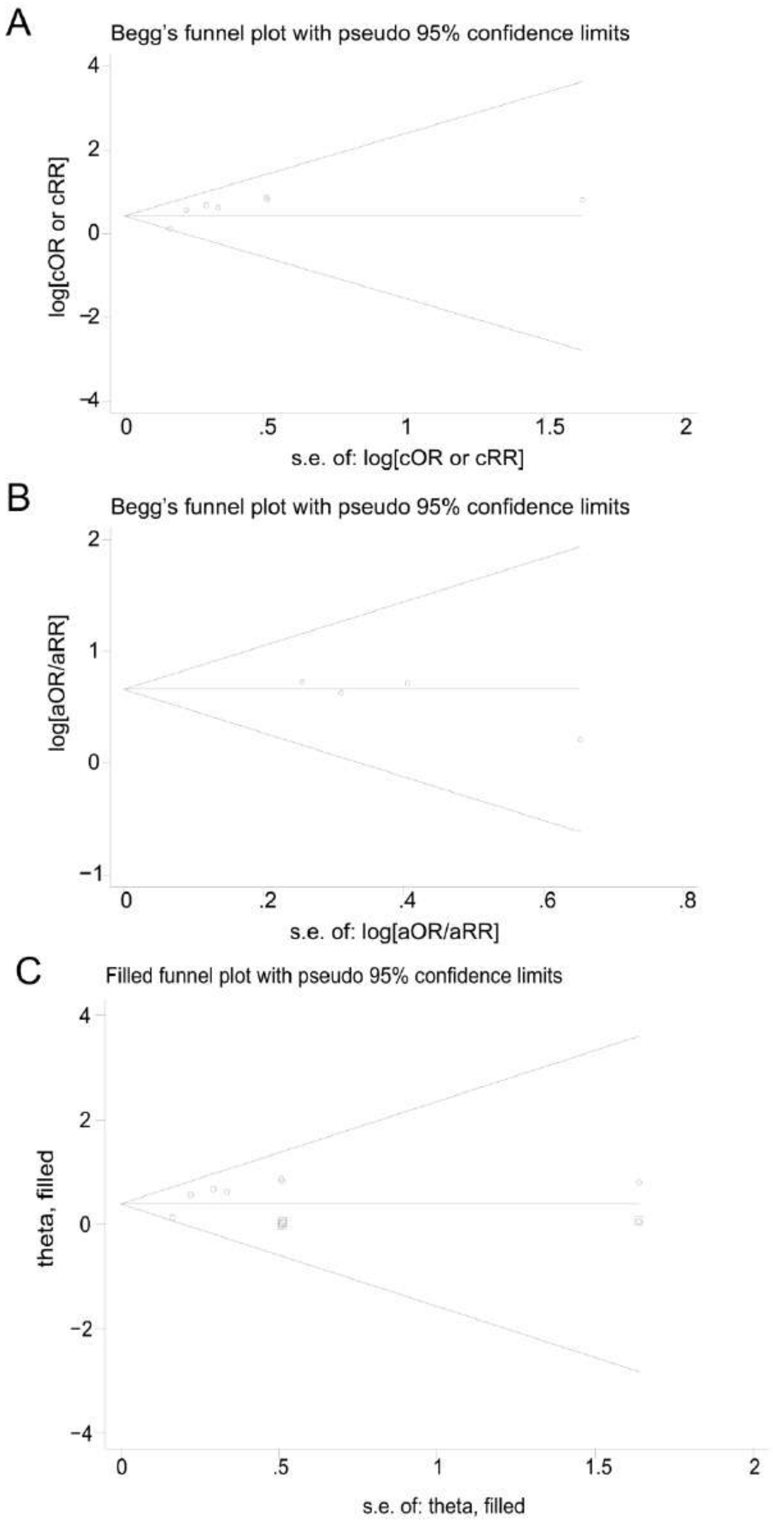

**Supplemental Figure 128.** Publication bias analysis by Begg's funnel plots of cOR (A) and aOR (B) for HT (vertical axis), and the standard error for cOR or aOR (horizontal axis). cOR, crude odds ratio; aOR, adjusted odds ratio; HT, hemorrhagic transformation.

A

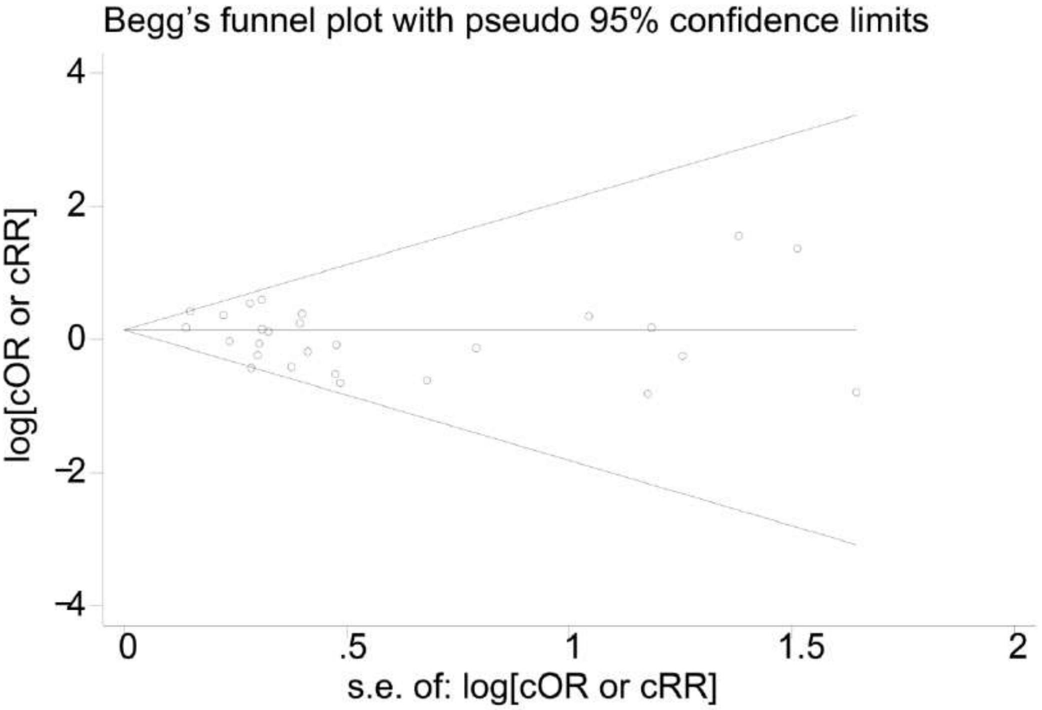


B

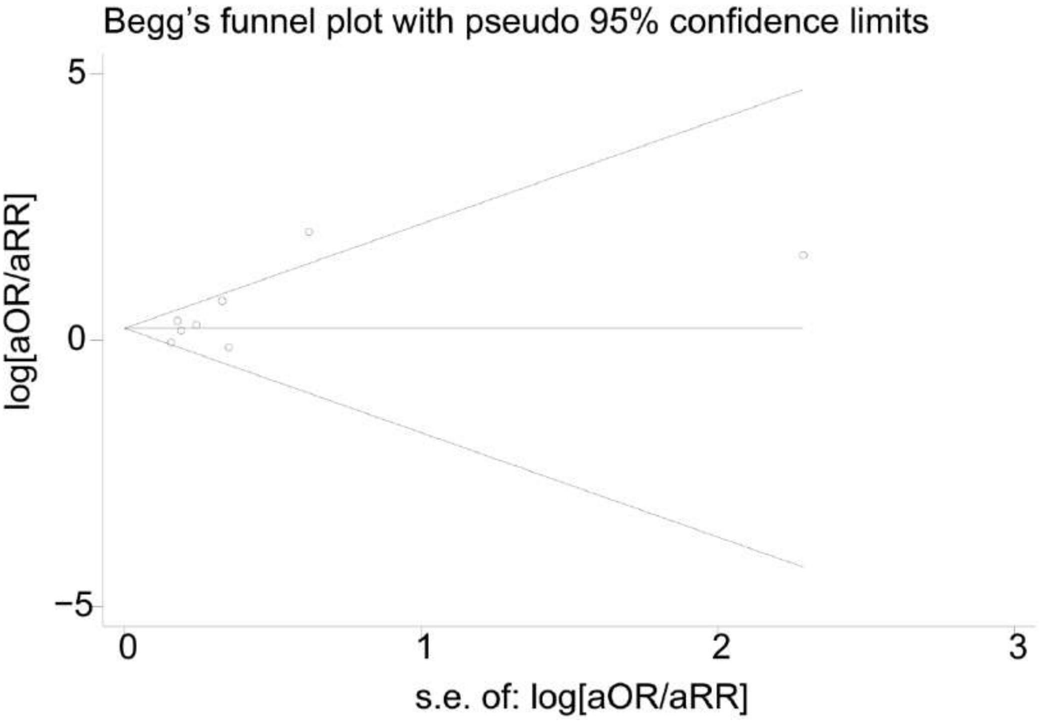

**Supplemental Figure 129.** Publication bias analysis by Begg's funnel plots of cOR (A) and aOR (B) for any bleeding (vertical axis), and the standard error for cOR or aOR (horizontal axis). cOR, crude odds ratio; aOR, adjusted odds ratio.

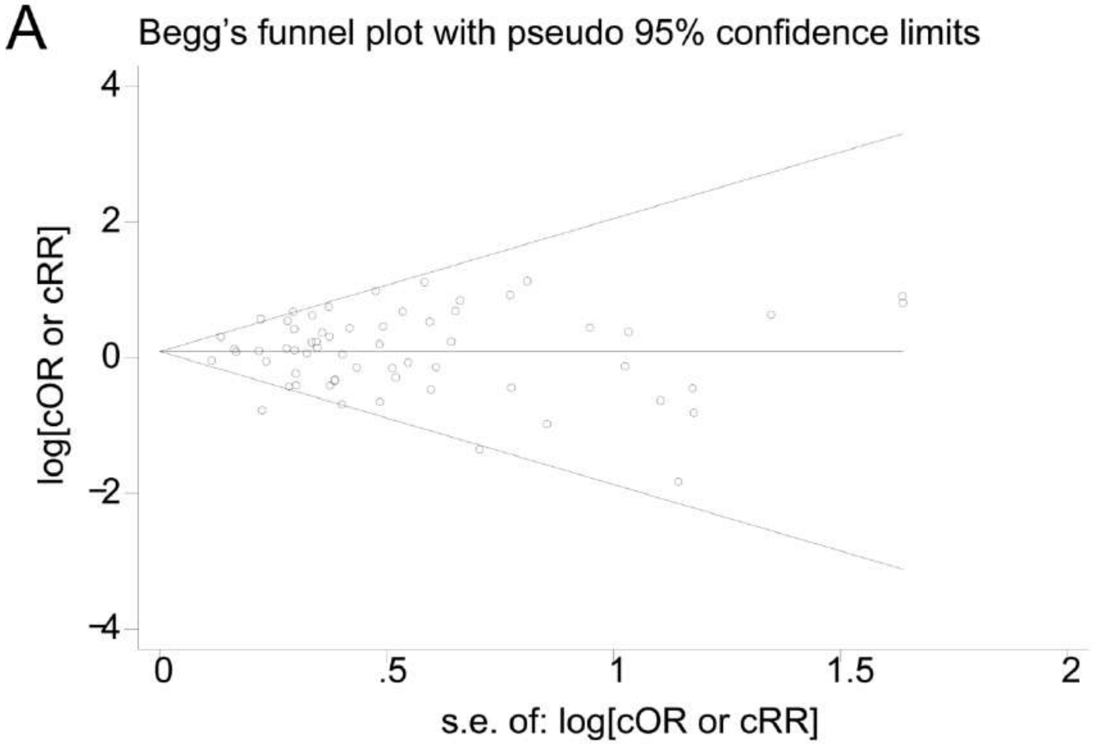


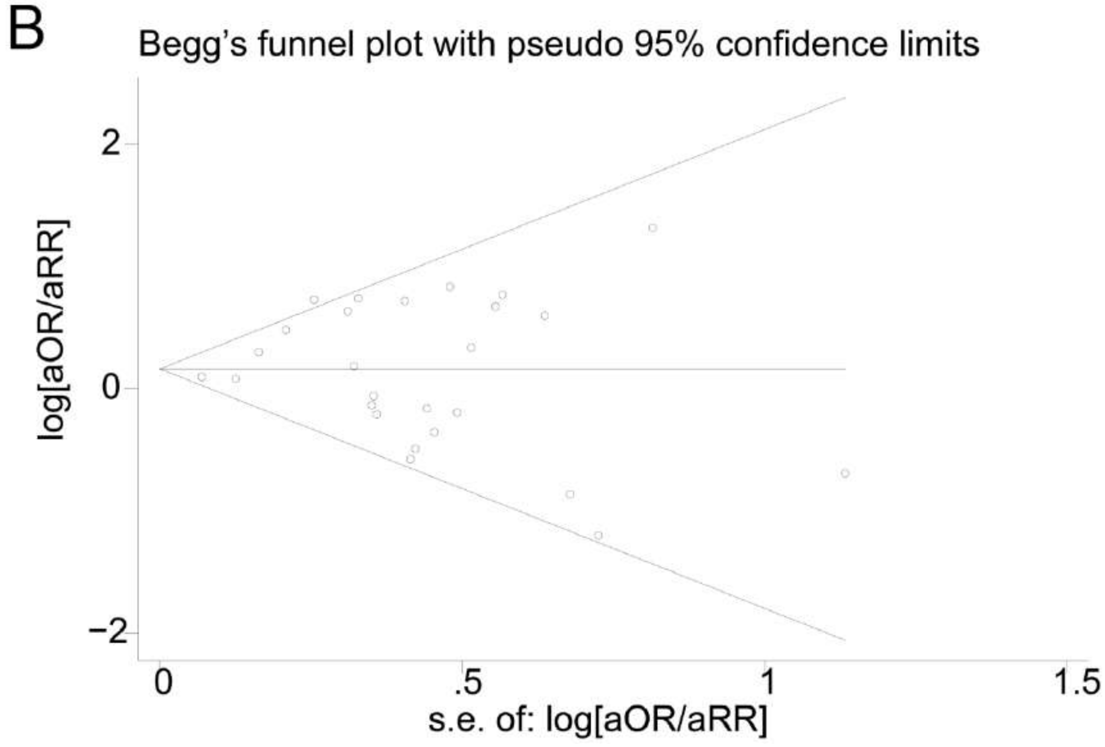

**Supplemental Figure 130.** Publication bias analysis by Begg's funnel plots of cOR for SAH (A) and HI (B) (vertical axis), and the standard error for cOR (horizontal axis). cOR, crude odds ratio; SAH, subarachnoid hemorrhage. HI, hemorrhagic infarction.

A

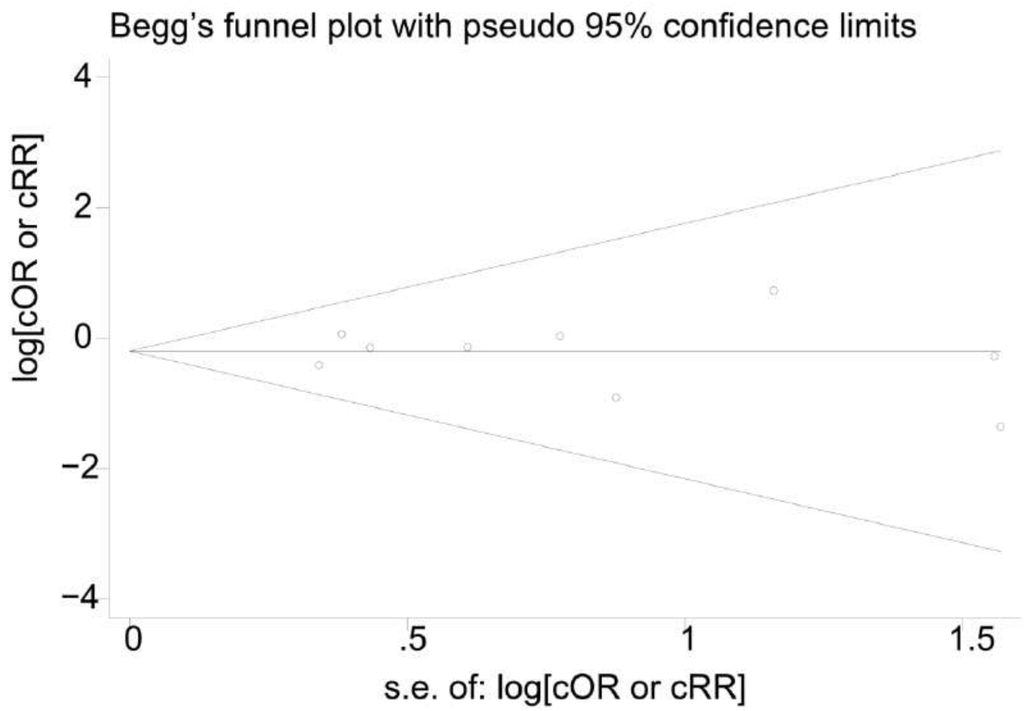


B

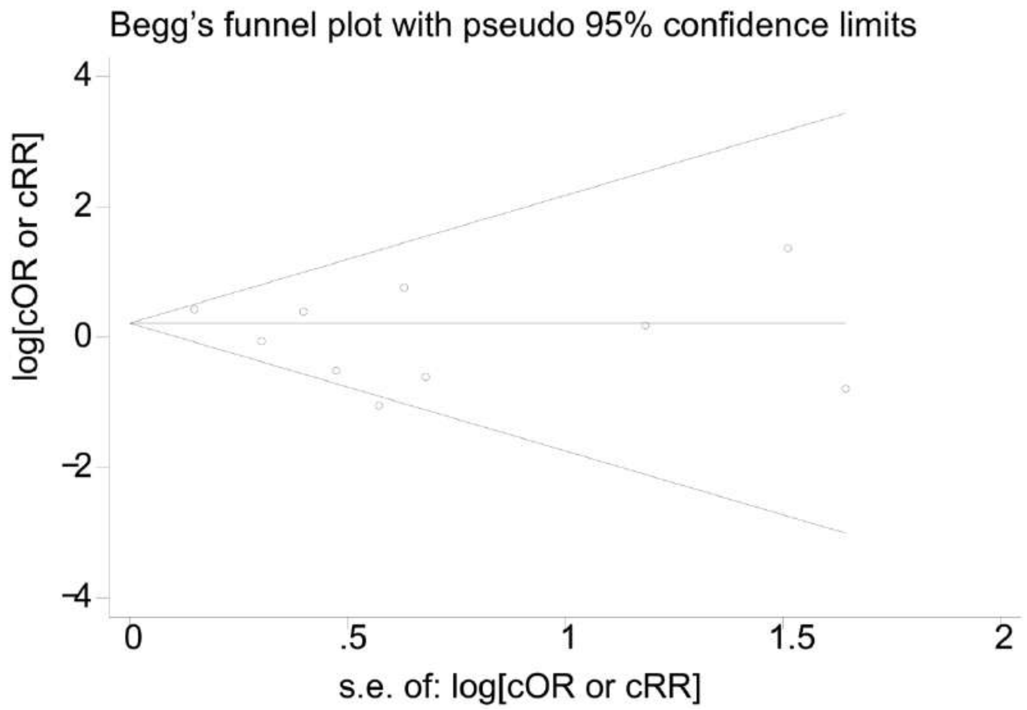

**Supplemental Figure 131.** Publication bias analysis by Begg's funnel plots of cOR (A) and aOR (B) for PH (vertical axis), and the standard error for cOR or aOR (horizontal axis). cOR, crude odds ratio; aOR, adjusted odds ratio. PH, parenchymal hematoma.

A

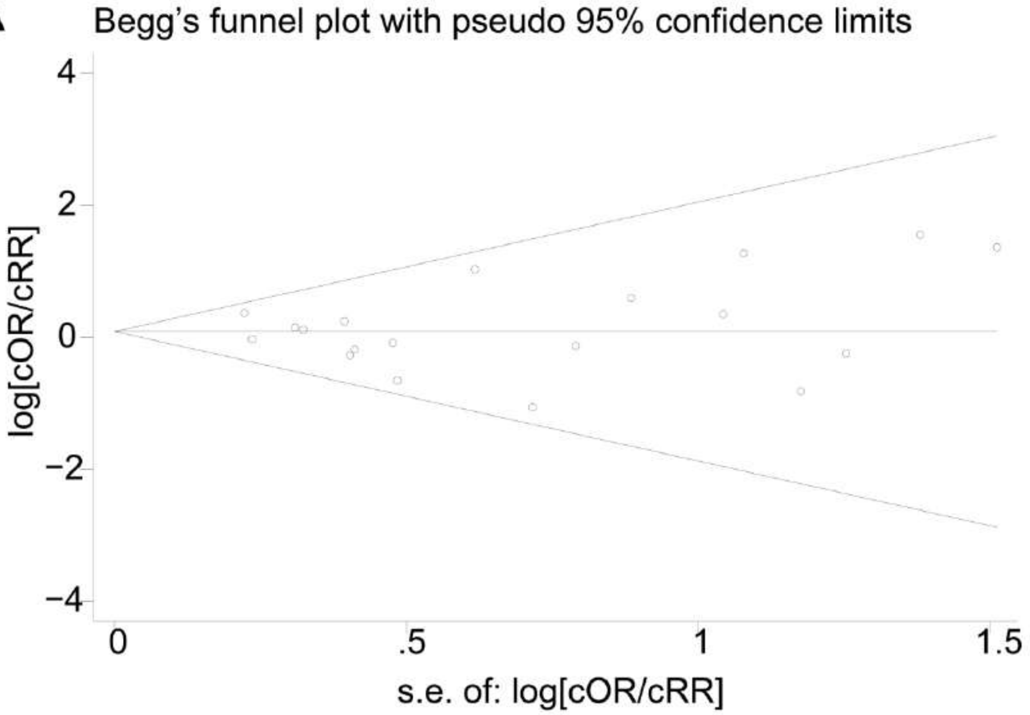


B

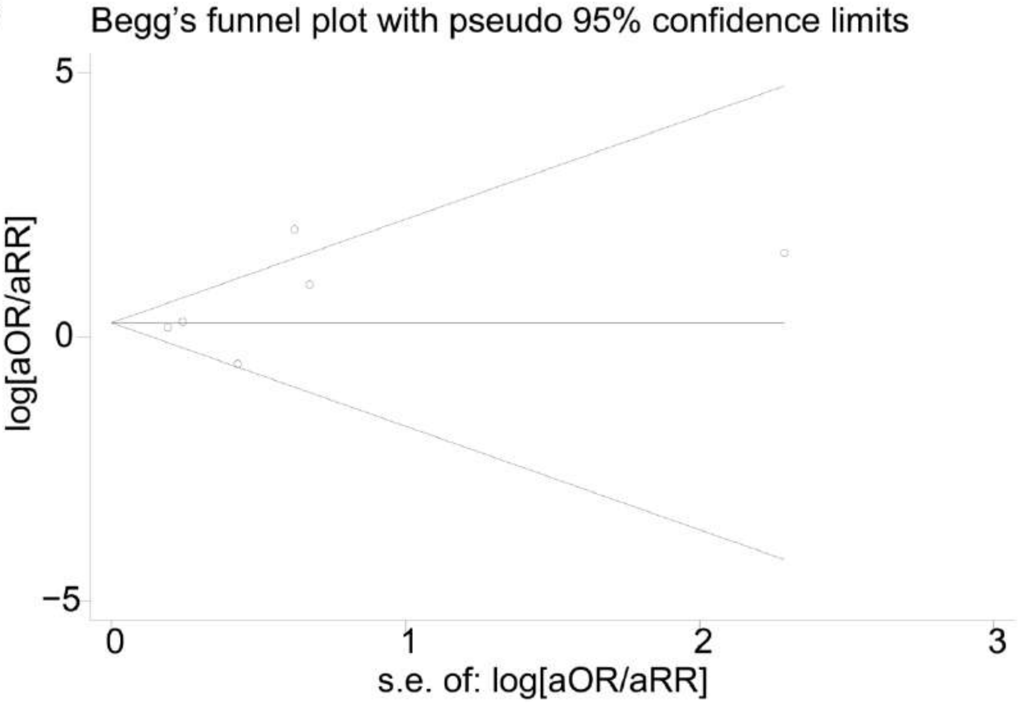

**Supplemental Figure 132.** Publication bias analysis by Begg's funnel plots of cOR (A) and aOR (B) for early recovery at 24h (vertical axis), and the standard error for cOR or aOR (horizontal axis). cOR, crude odds ratio; aOR, adjusted odds ratio.

A

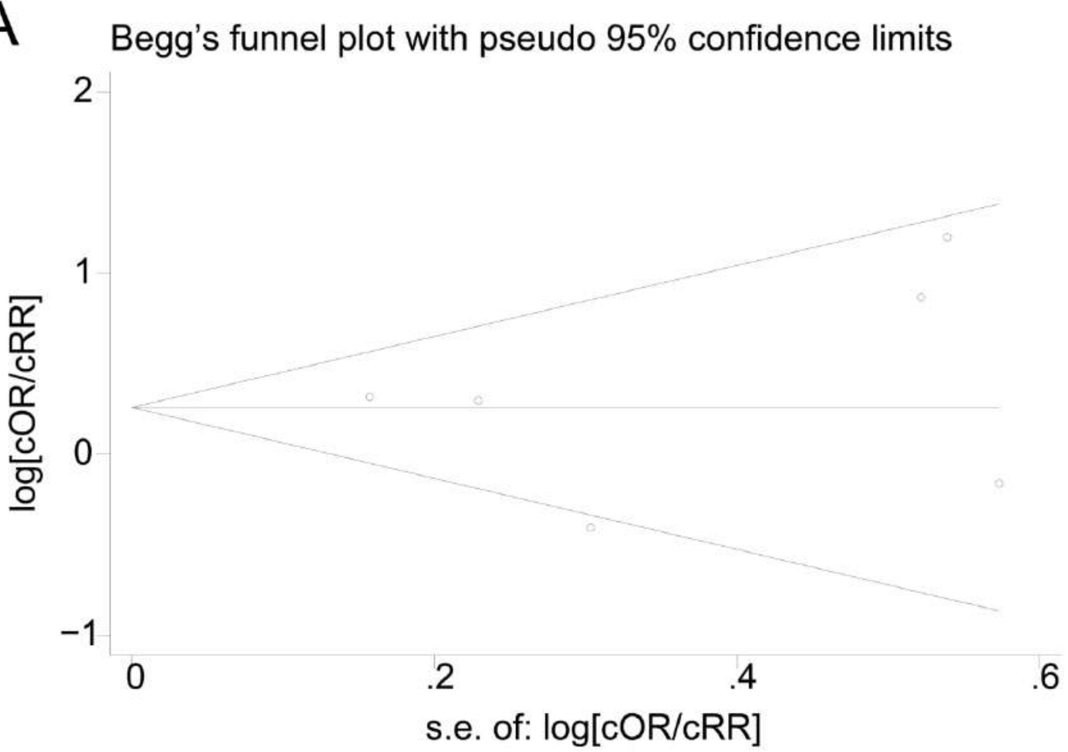


B

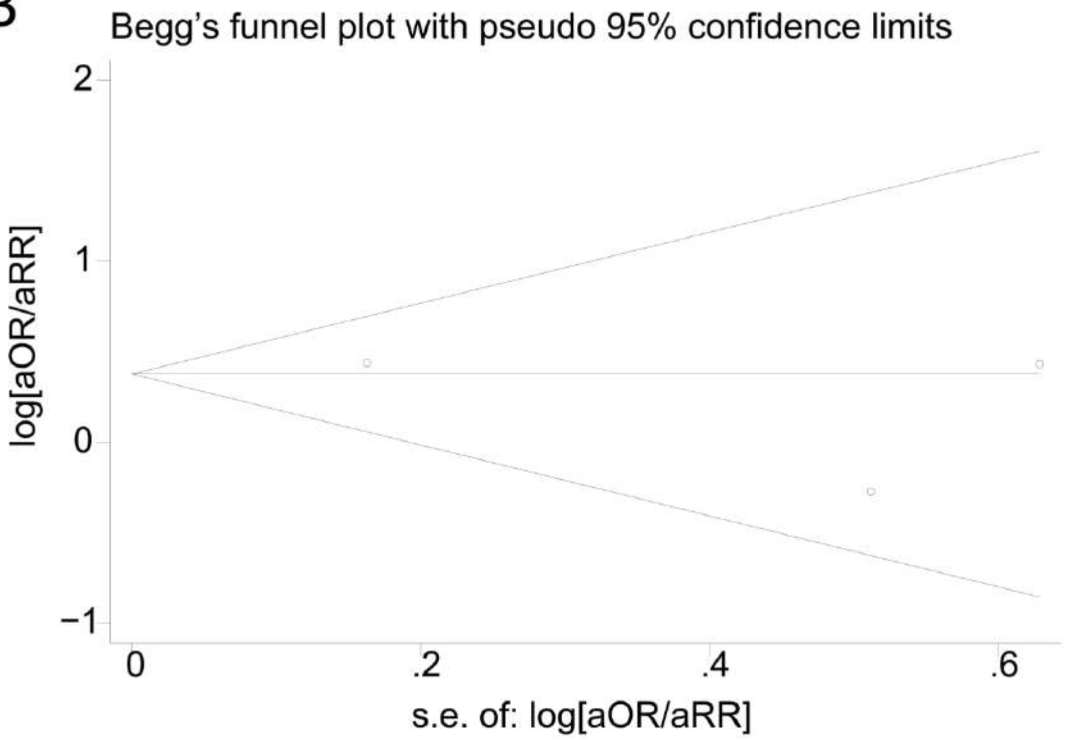

**Supplemental Figure 133.** Publication bias analysis by Begg's funnel plots of cOR for dramatic improvement at discharge/7days (A) and good improvement at discharge/7days (B) (vertical axis), and the standard error for cOR (horizontal axis). cOR, crude odds ratio.

A

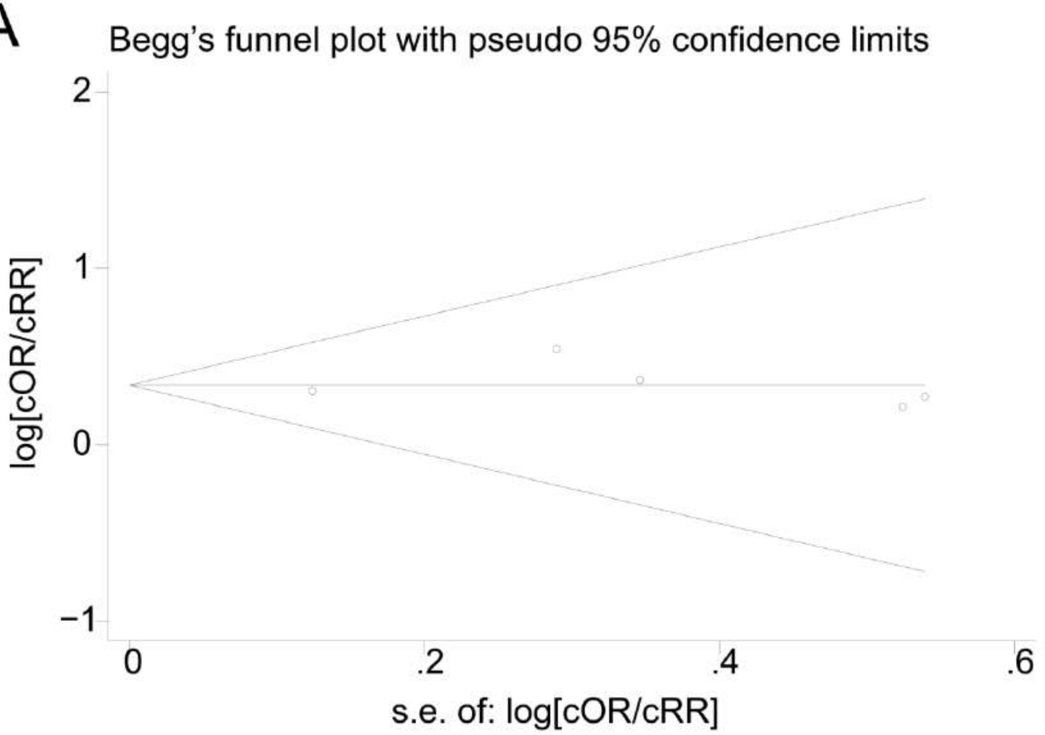


B

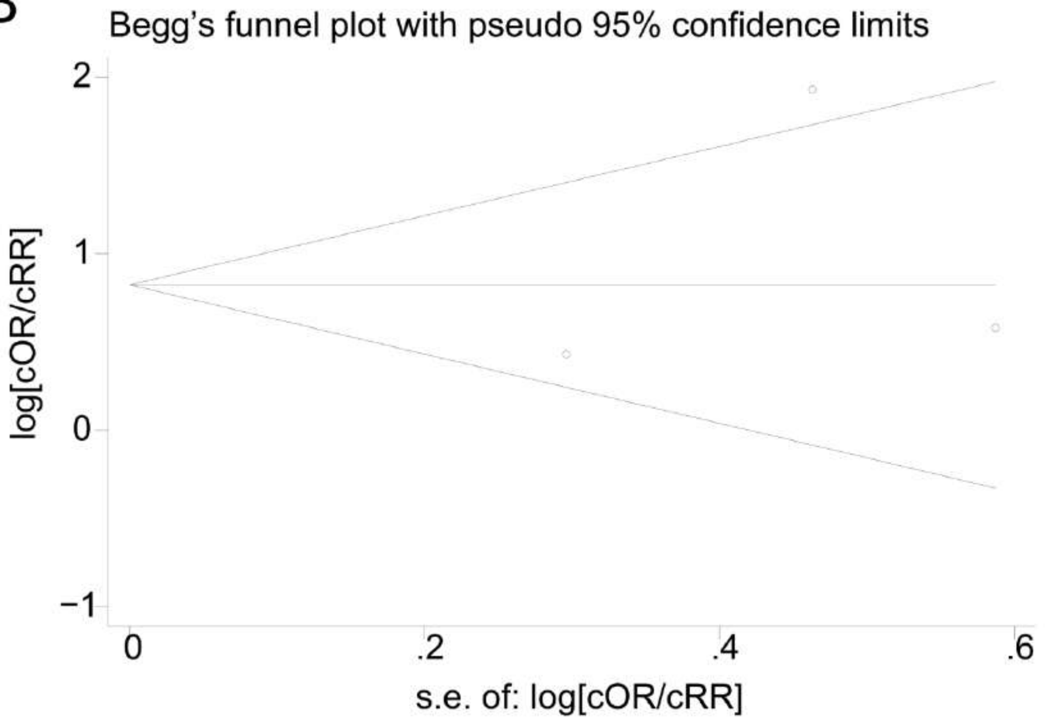

**Supplemental Figure 134.** Publication bias analysis by Begg's funnel plots (A) and "Trim and fill" funnel plots of (B) of aOR for passes number of thrombectomy device ≤2, and Begg's funnel plots of aOR for passes number of thrombectomy device=1 (C). aOR, adjusted odds ratio.

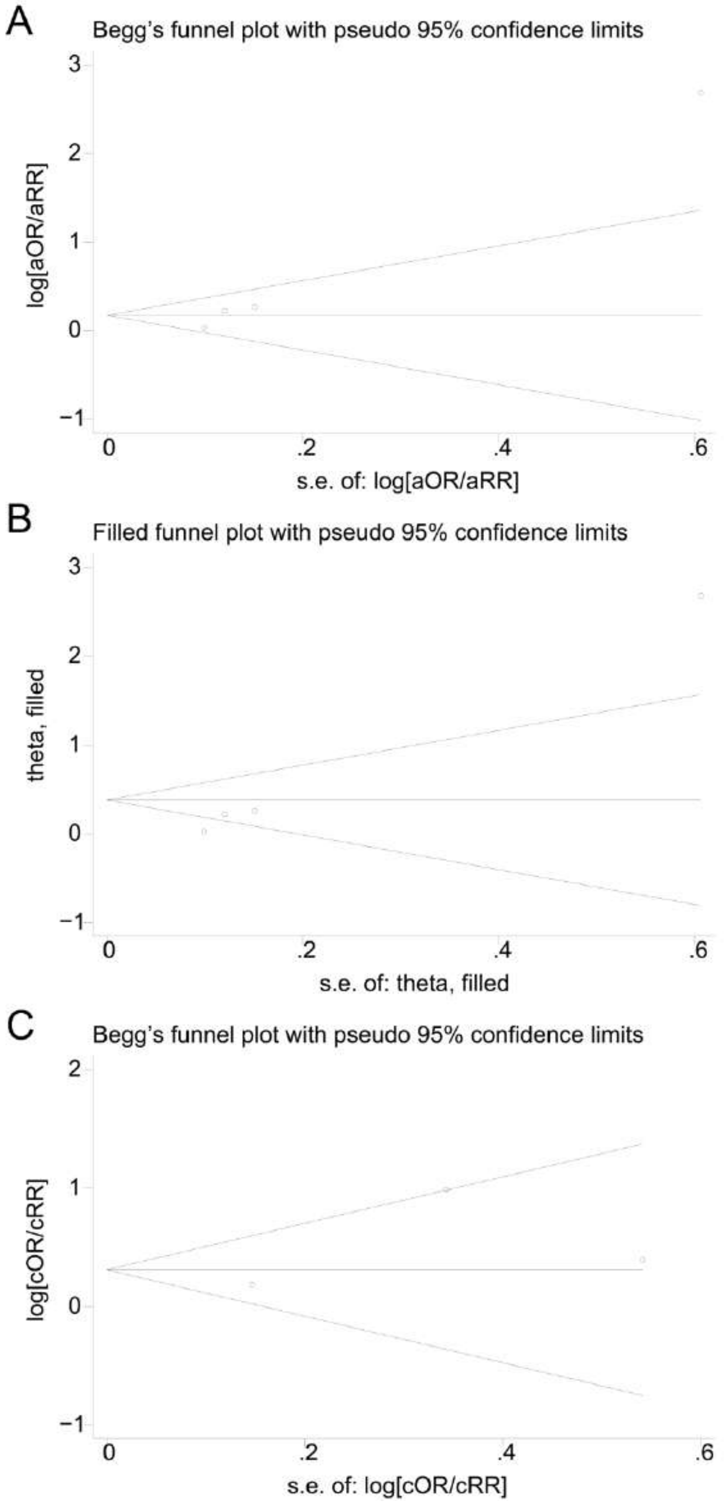

**Supplemental Figure 135.** Publication bias analysis by Begg's funnel plots of cOR (A) and aOR (B) for any complications, and by the "Trim and fill" funnel plots of aOR (C) for any complications. cOR, crude odds ratio; aOR, adjusted odds ratio.

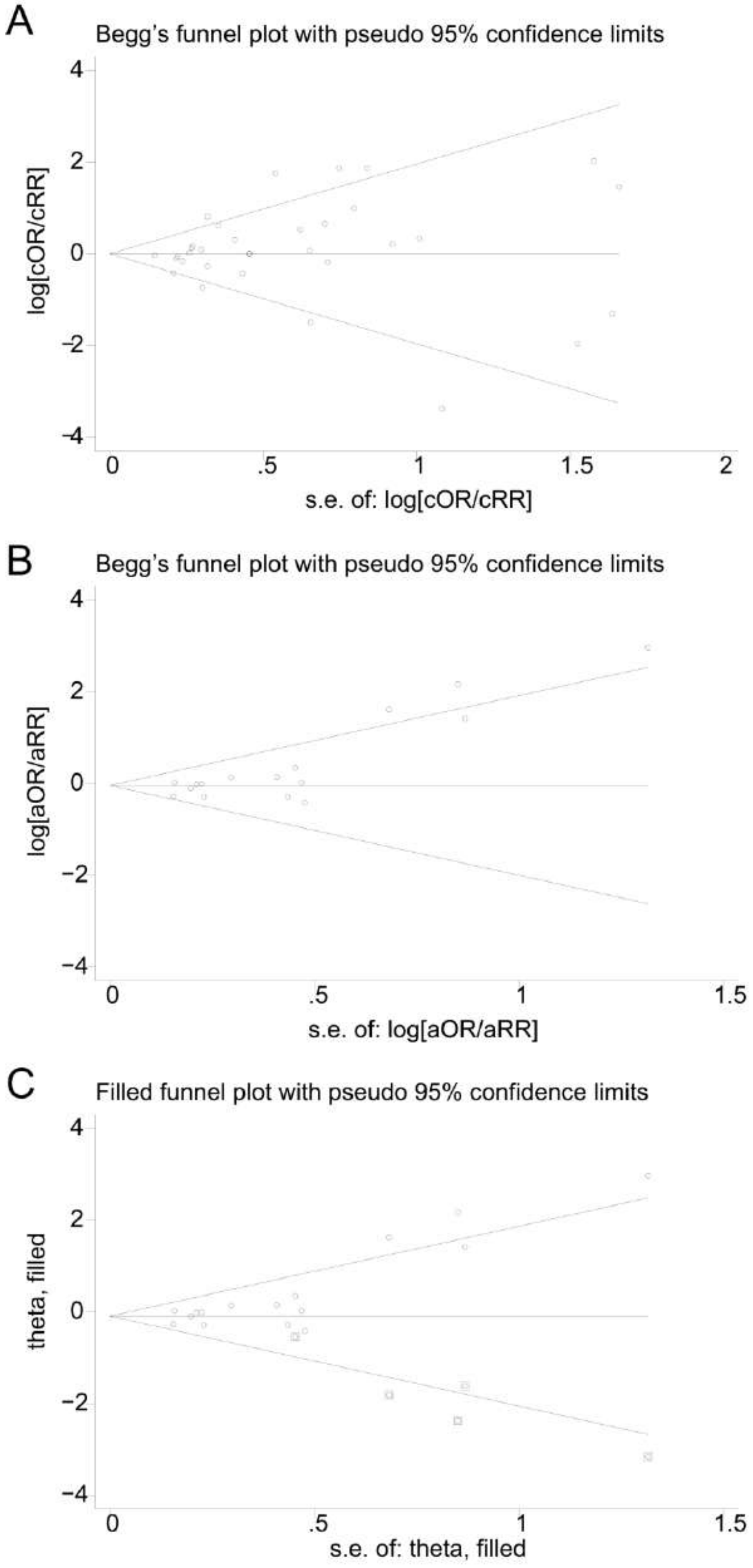

**Supplemental Figure 136.** Publication bias analysis by Begg's funnel plots of cOR (A) and aOR (B) for clot migration, and by the "Trim and fill" funnel plots of aOR (C) for clot migration. cOR, crude odds ratio; aOR, adjusted odds ratio.

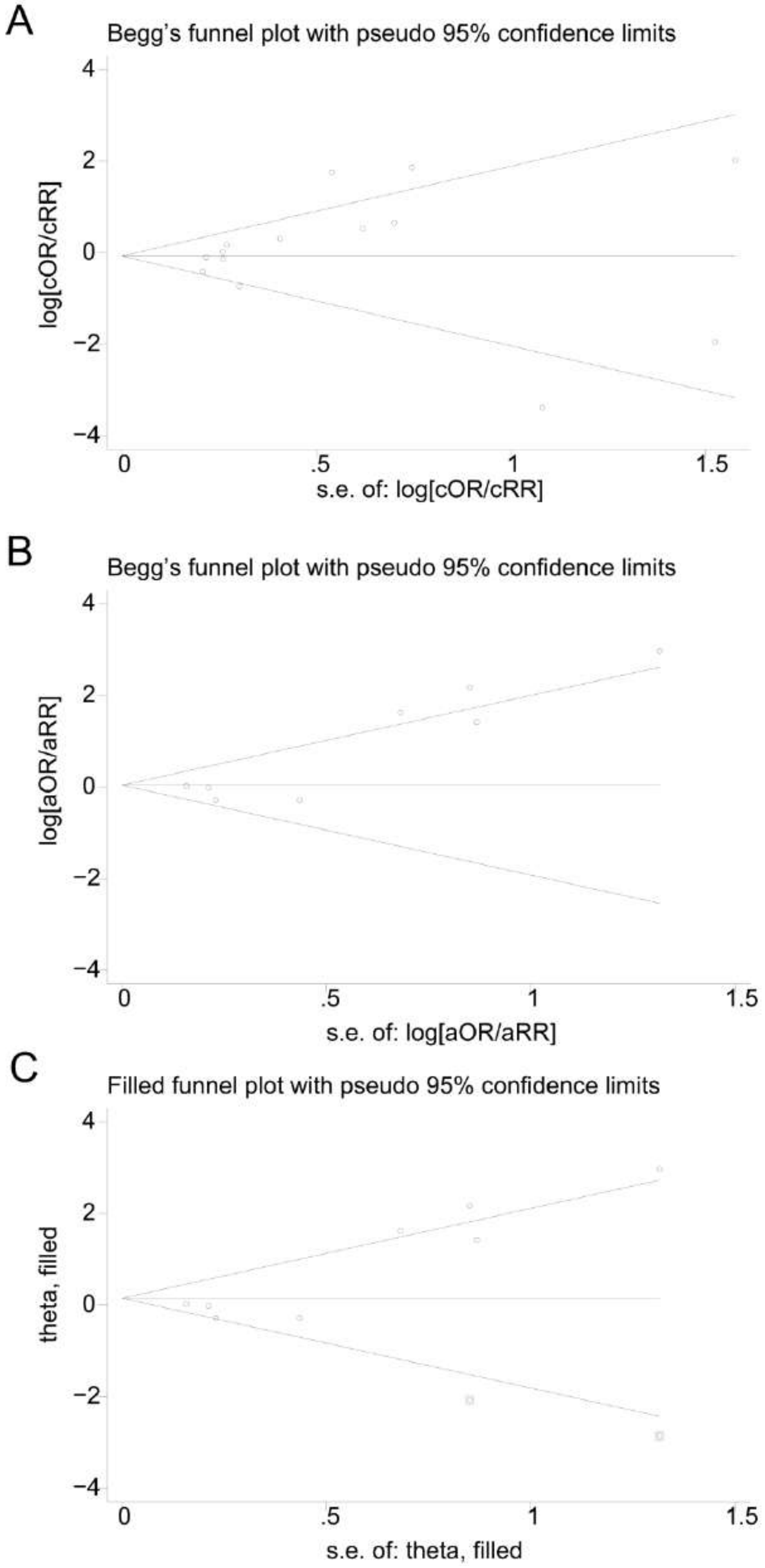

**Supplemental Figure 137.** Publication bias analysis by Begg's funnel plots of cOR for groin hematoma (A), recurrent stroke (B) and vasospasm (C). cOR, crude odds ratio.

A

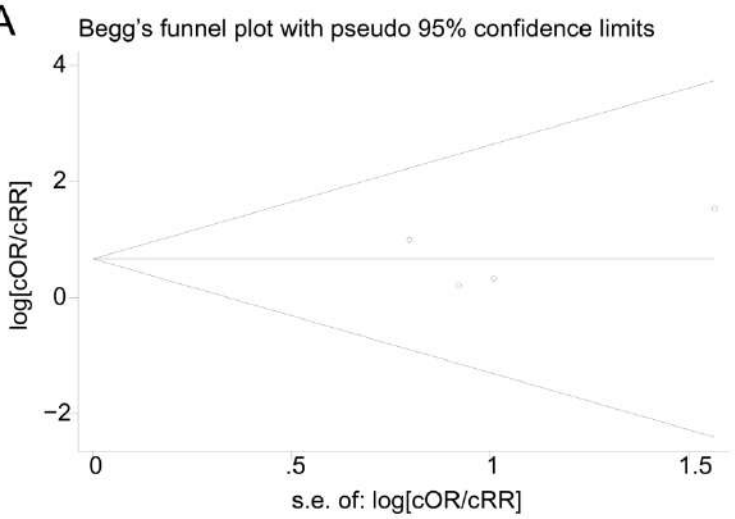


B

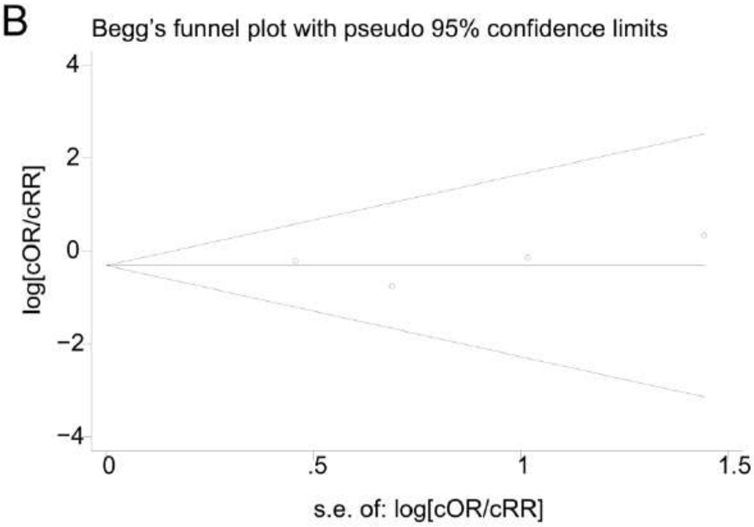


C

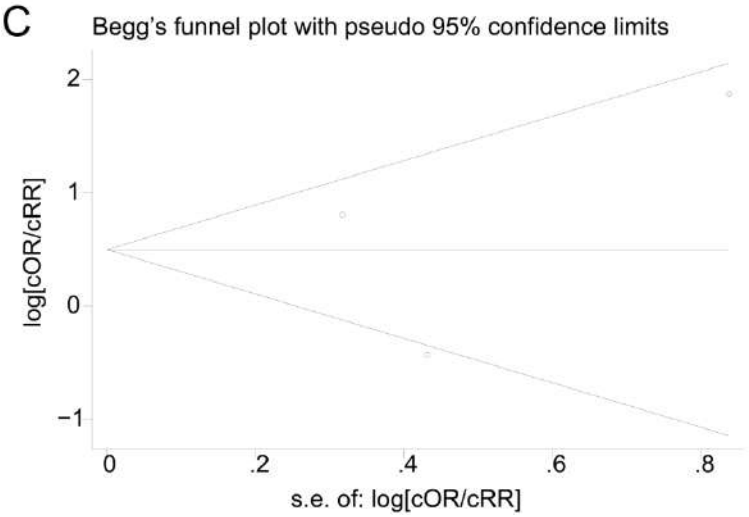

**Supplemental Figure 138.** Publication bias analysis by Begg's funnel plots (A) and "Trim and fill" funnel plots (B) of cOR for vessel dissection, and Begg's funnel plots of cOR for vessel perforation (C). cOR, crude odds ratio.

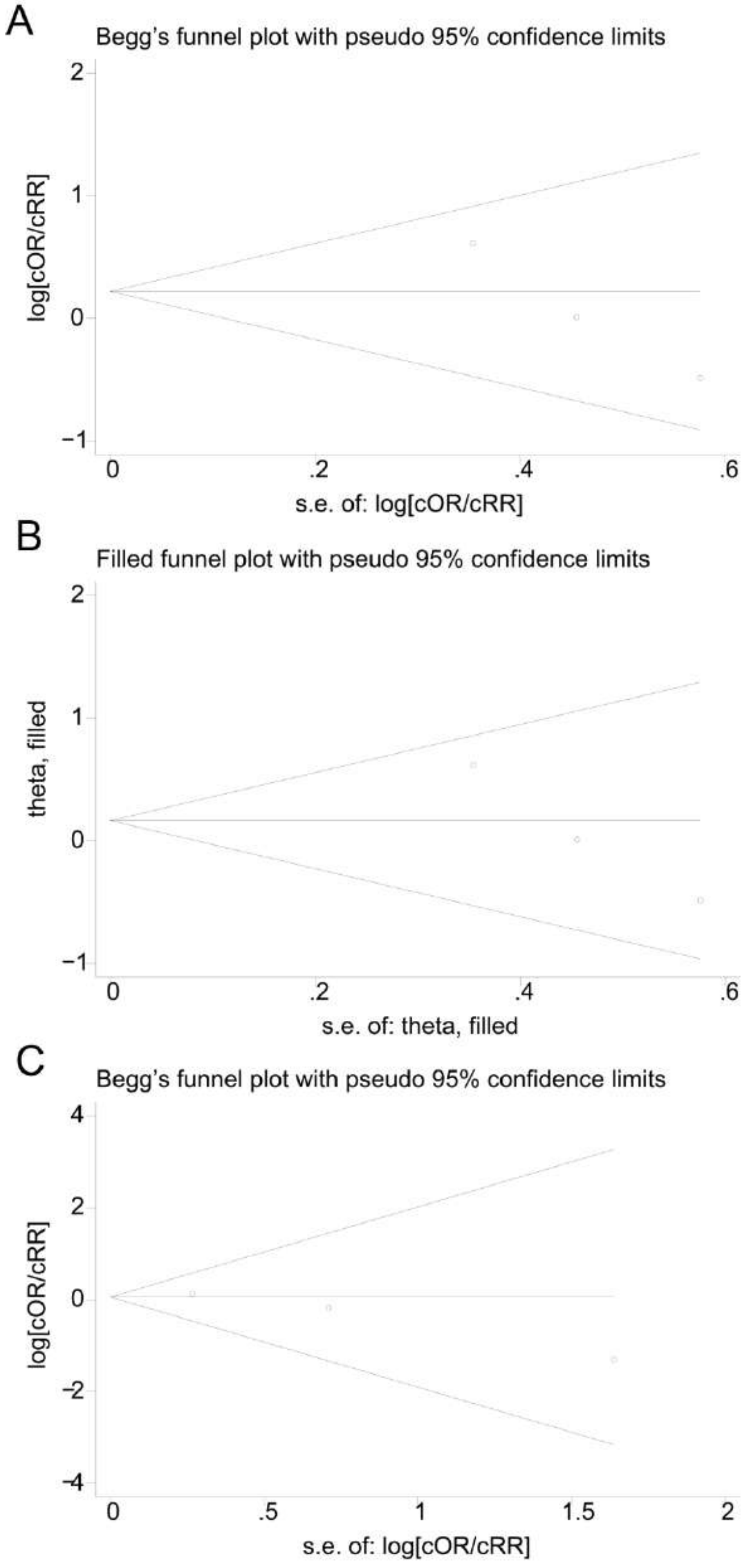

**Supplemental Figure 139.** Forest plots of RCT studies assessing good outcome at 90 days in unadjusted (A) and adjusted (B) analysis.

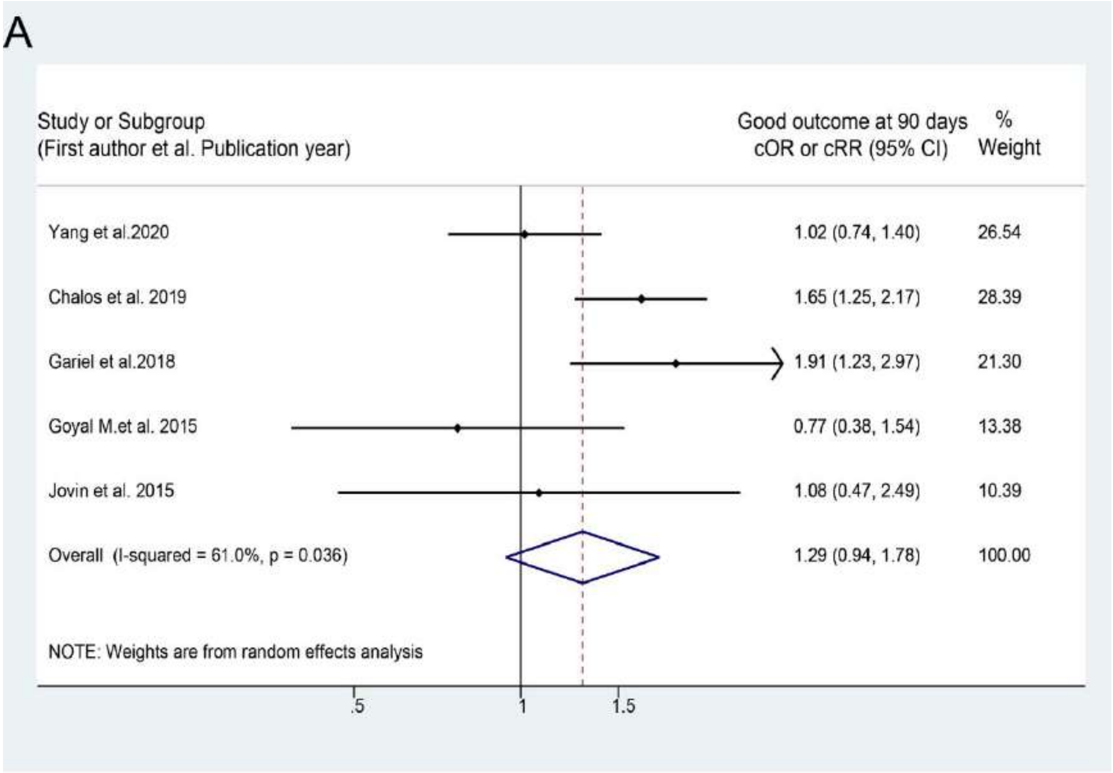


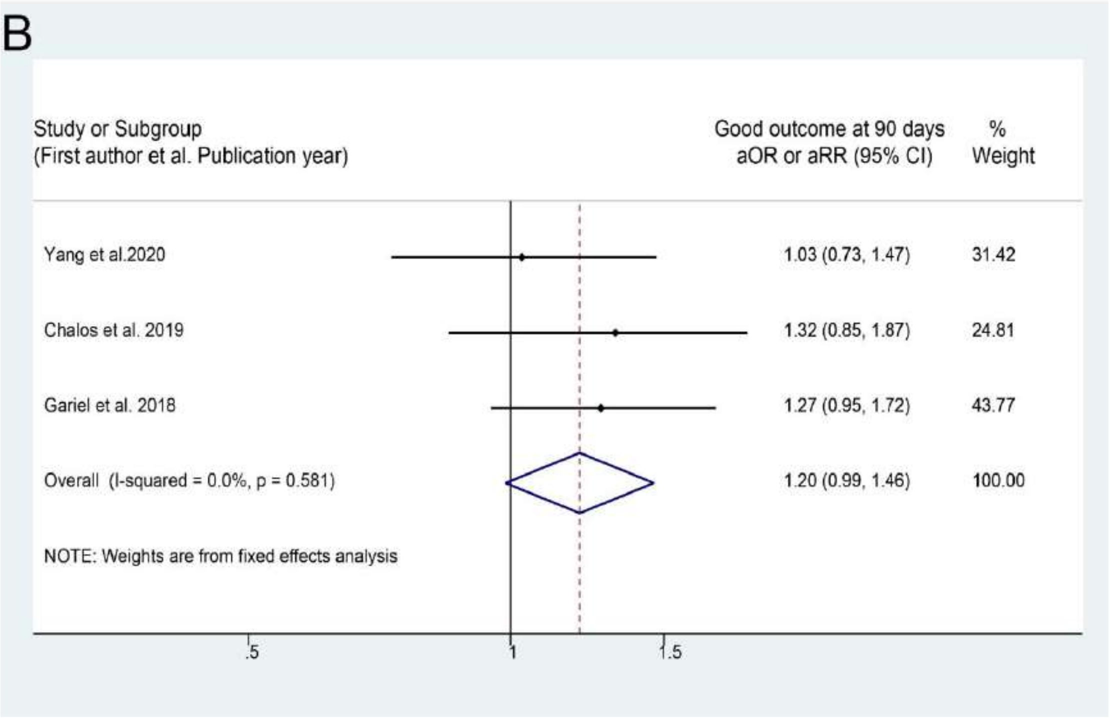

**Supplemental Figure 140.** Sensitivity analysis of the effect of individual RCT studies on the cOR (A) and aOR (B) for good outcome at 90 days, and the publication bias analysis by Begg's funnel plots of cOR (C) and aOR (D).

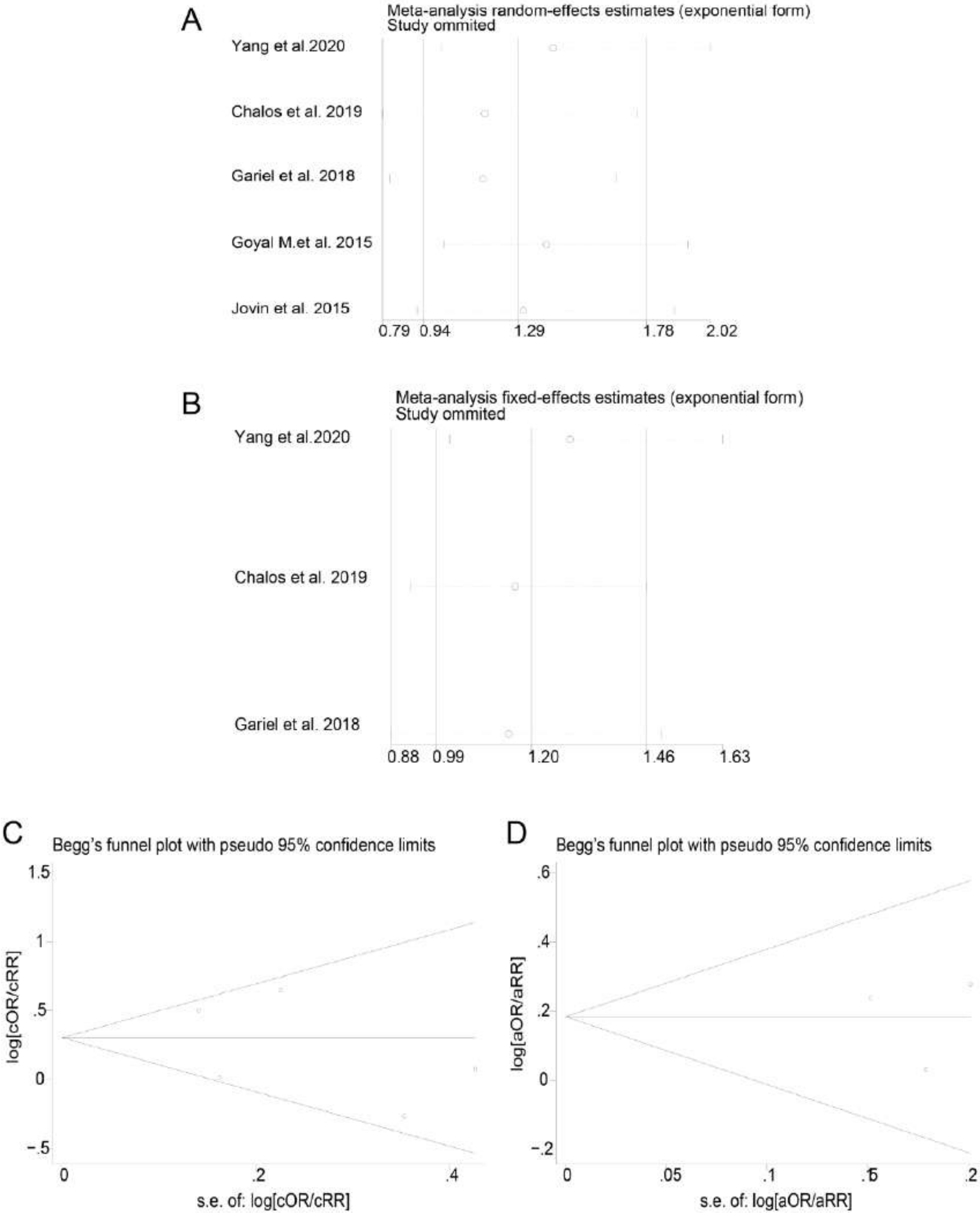

**Supplemental Figure 141.** Forest plots of RCT studies assessing excellent outcome at 90 days in unadjusted (A) and adjusted (B) analysis.

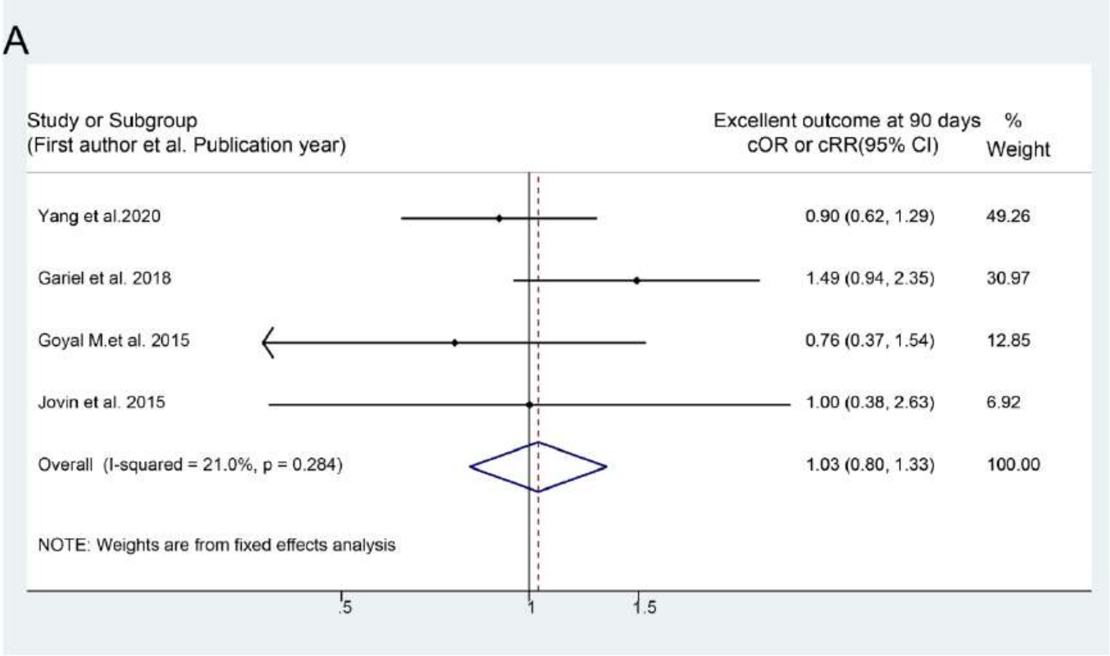


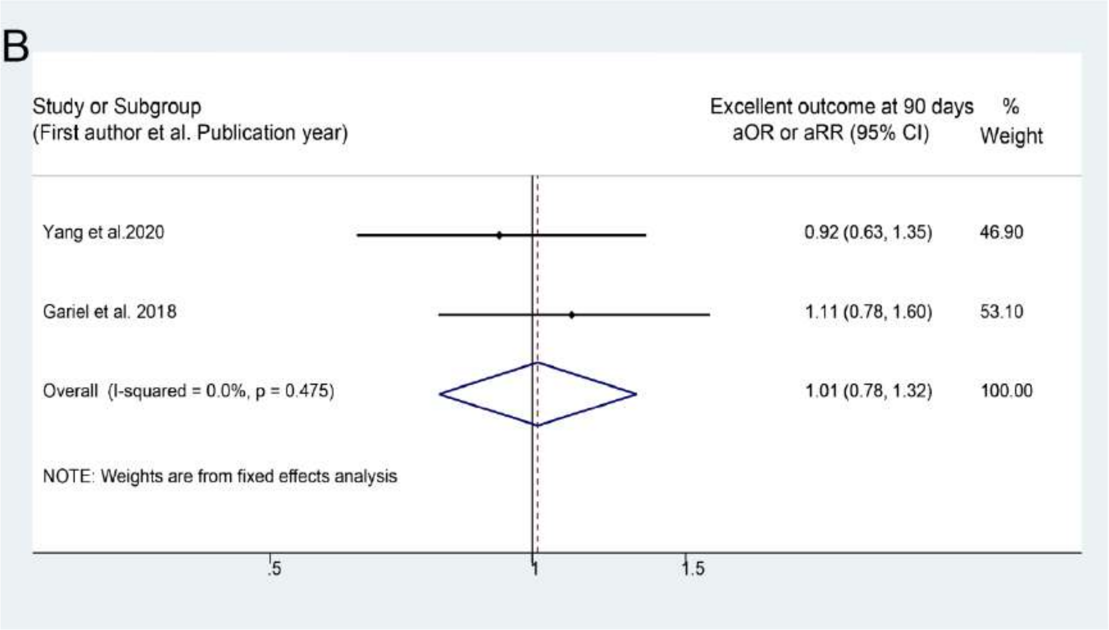

**Supplemental Figure 142.** Sensitivity analysis of the effect of individual RCT studies on the cOR (A) and aOR (B) for excellent outcome at 90 days, and the publication bias analysis by Begg's funnel plots of cOR (C) and aOR (D).

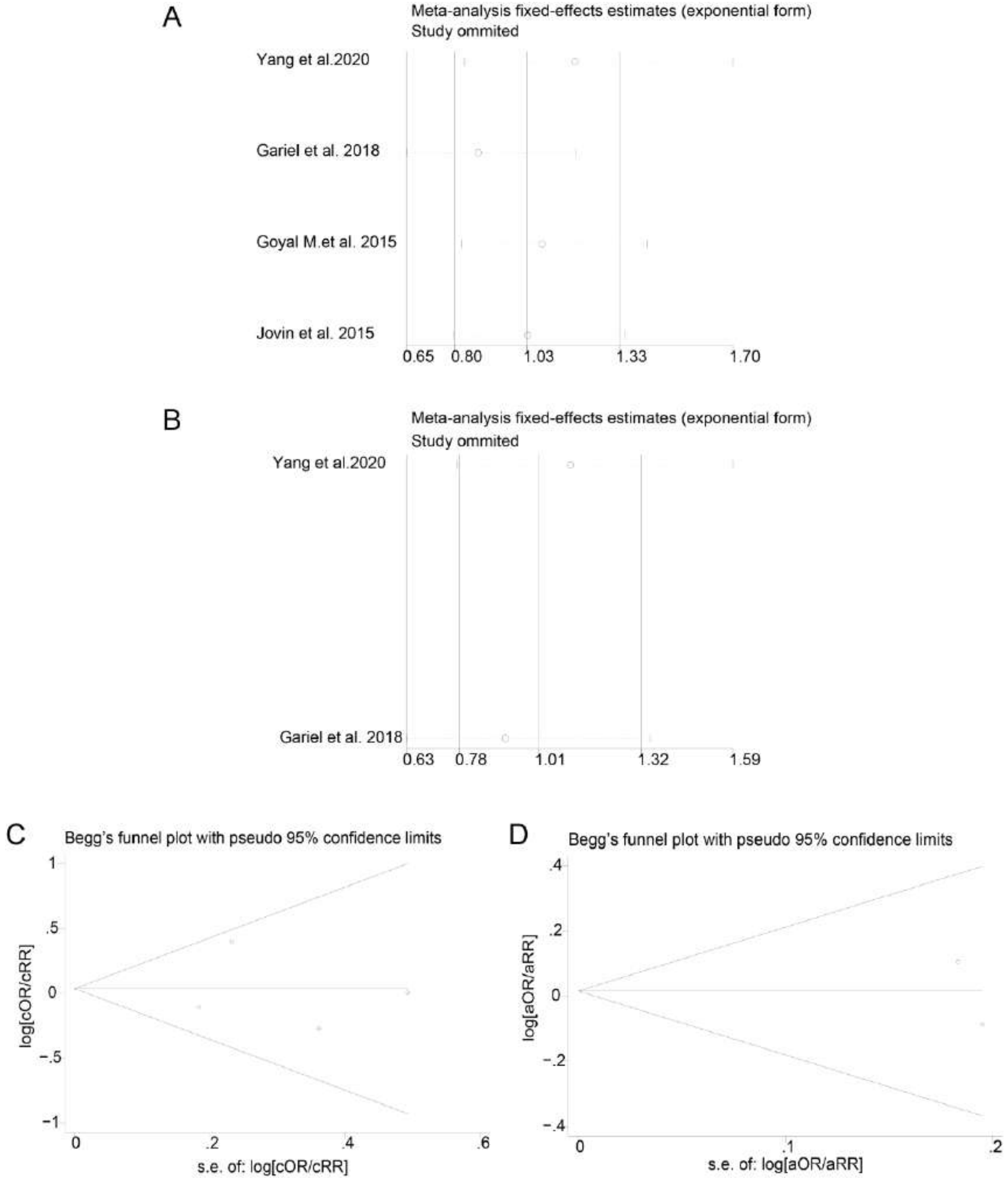

**Supplemental Figure 143**. Forest plots of RCT studies assessing mortality at 90 days in unadjusted (A) and adjusted (B) analysis.

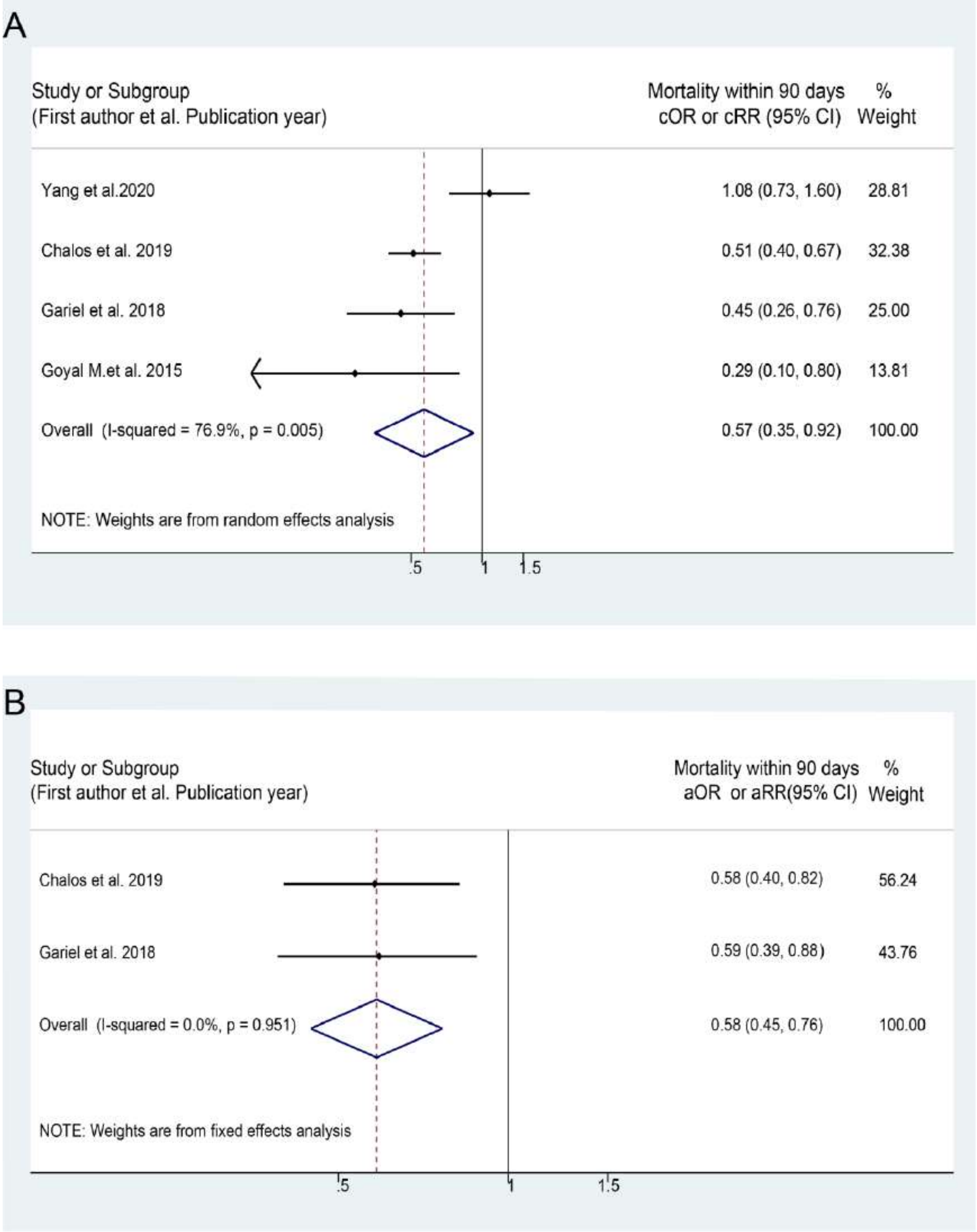

**Supplemental Figure 144.** Sensitivity analysis of the effect of individual RCT studies on the cOR (A) and aOR (B) for mortality at 90 days, and the publication bias analysis by Begg's funnel plots of cOR (C) and aOR (D).

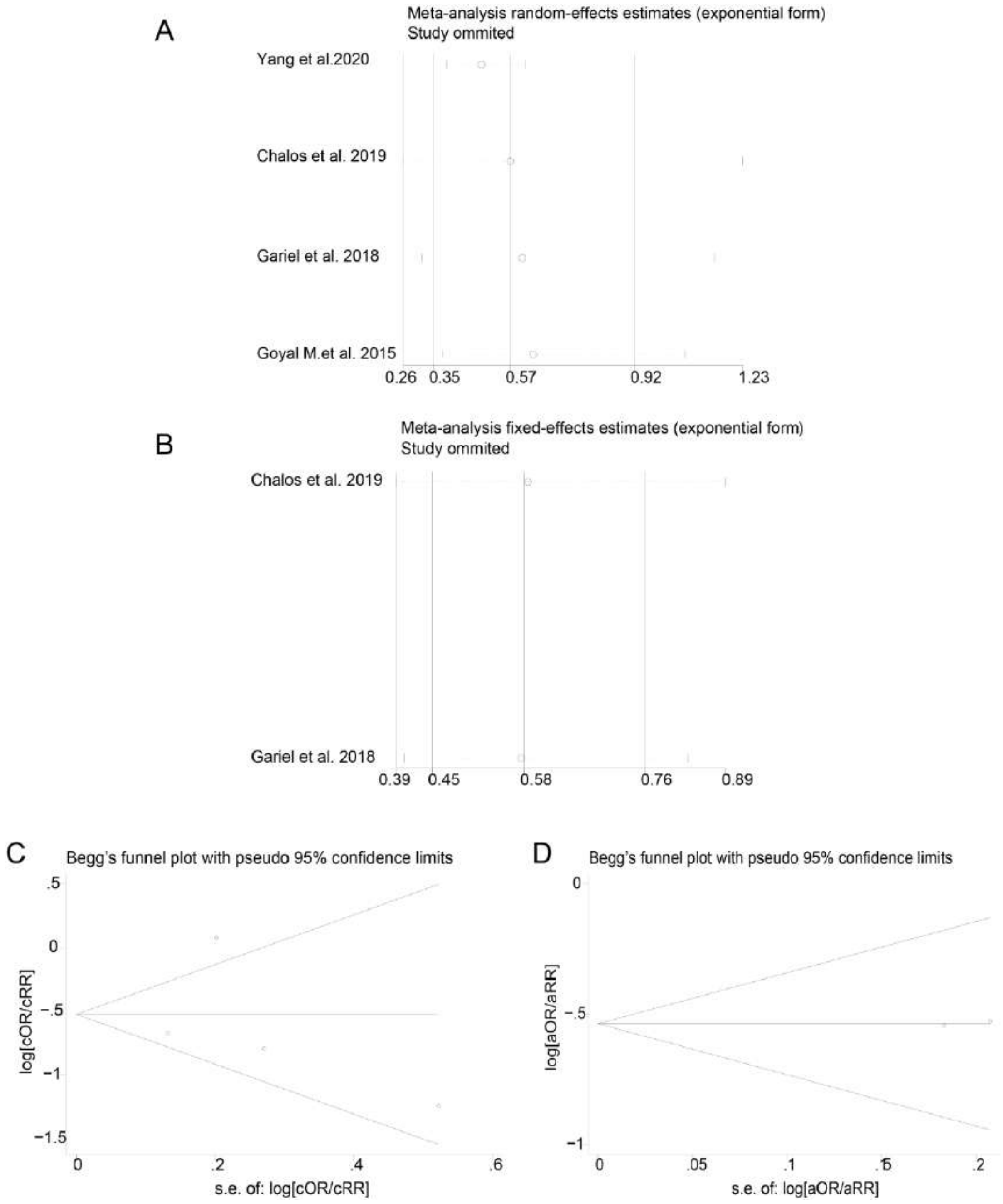

**Supplemental Figure 145.** Forest plots of RCT studies assessing successful reperfusion in unadjusted (A) and adjusted (B) analysis.

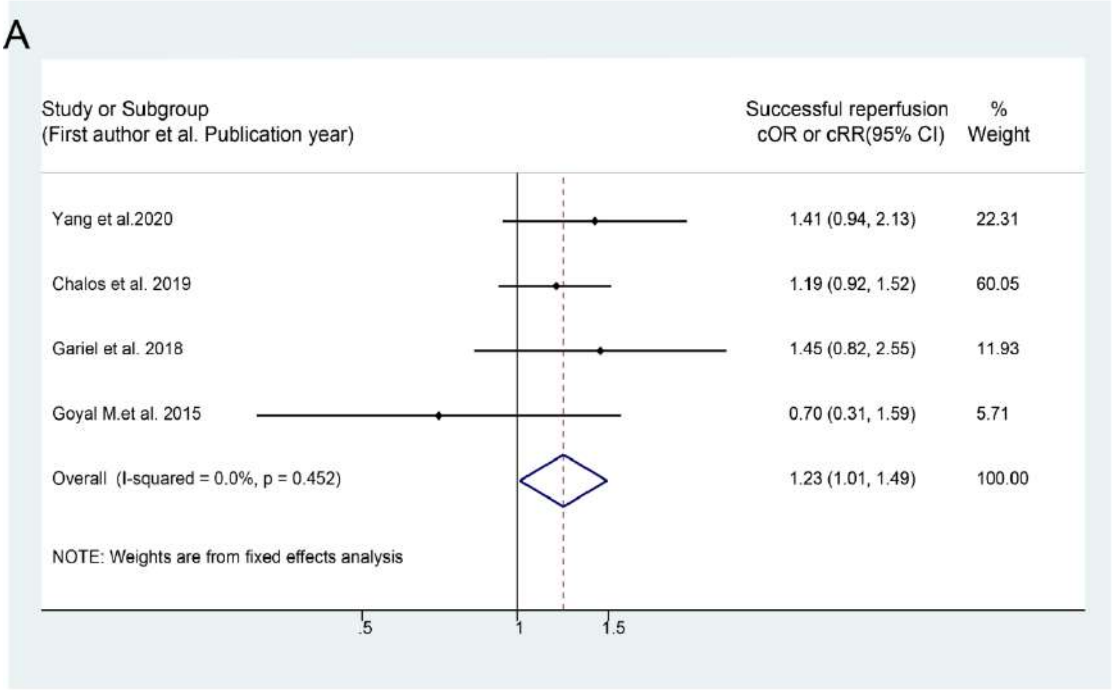


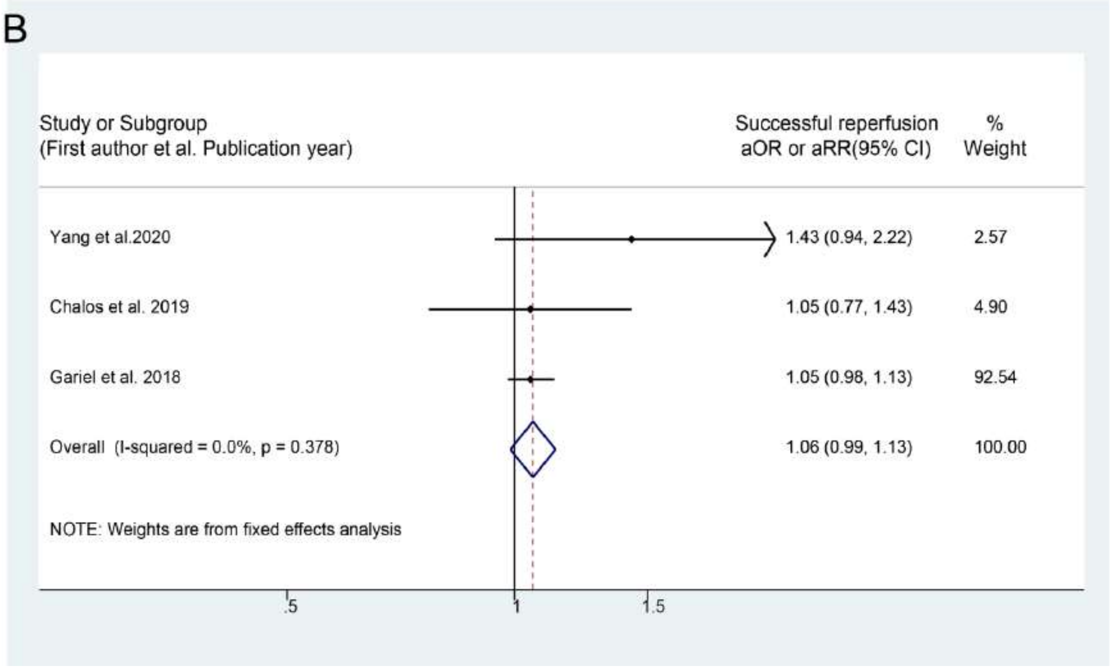

**Supplemental Figure 146.** Sensitivity analysis of the effect of individual RCT studies on the cOR (A) and aOR (B) for successful reperfusion, and the publication bias analysis by Begg's funnel plots of cOR (C) and aOR (D).

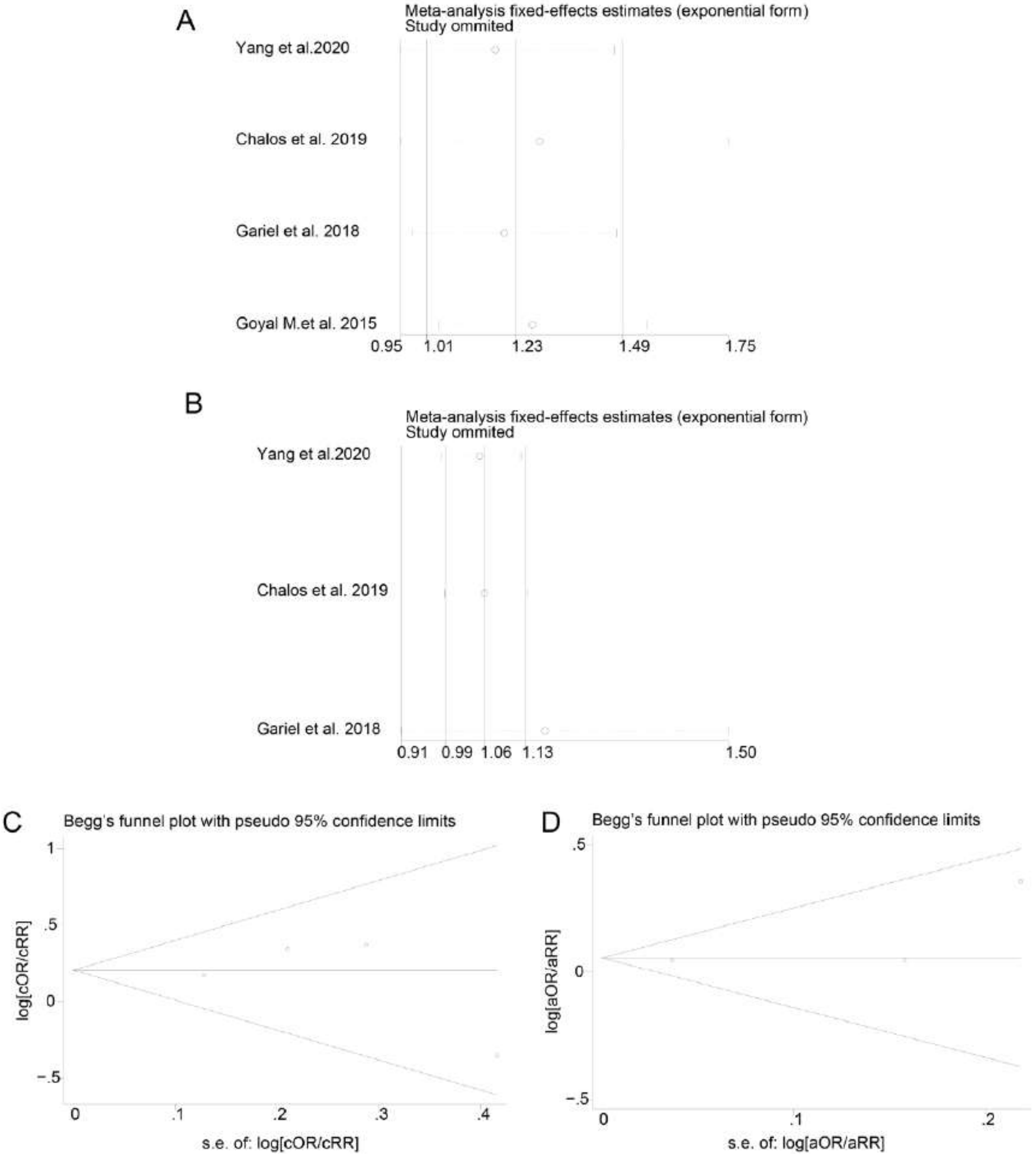

**Supplemental Figure 147.** Forest plots of RCT studies assessing Sich in unadjusted (A) and adjusted (B) analysis. sICH, symptomatic intracranial hemorrhage.

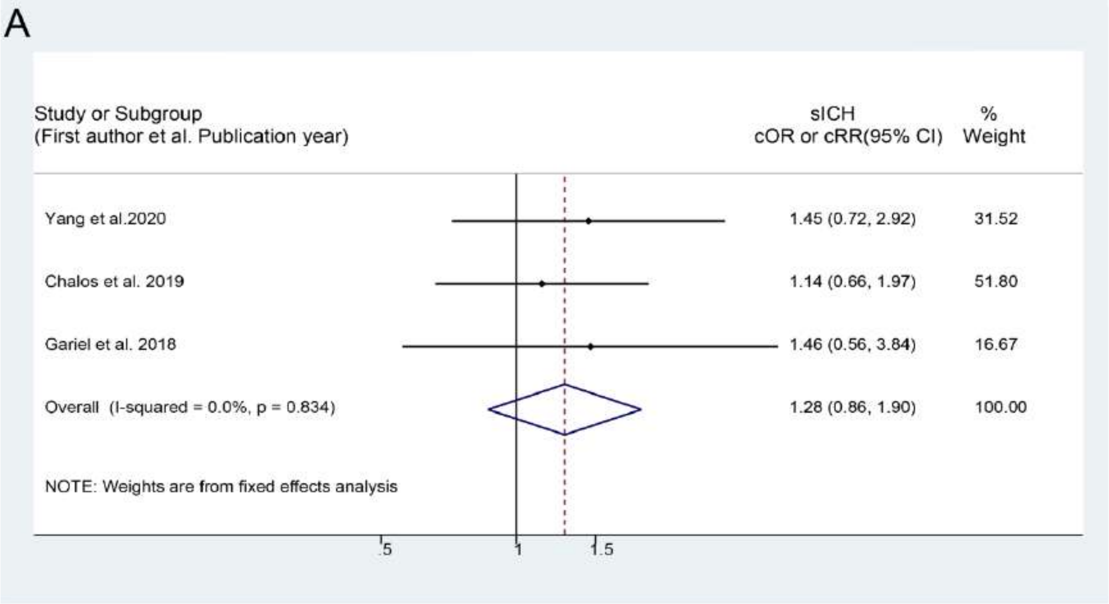


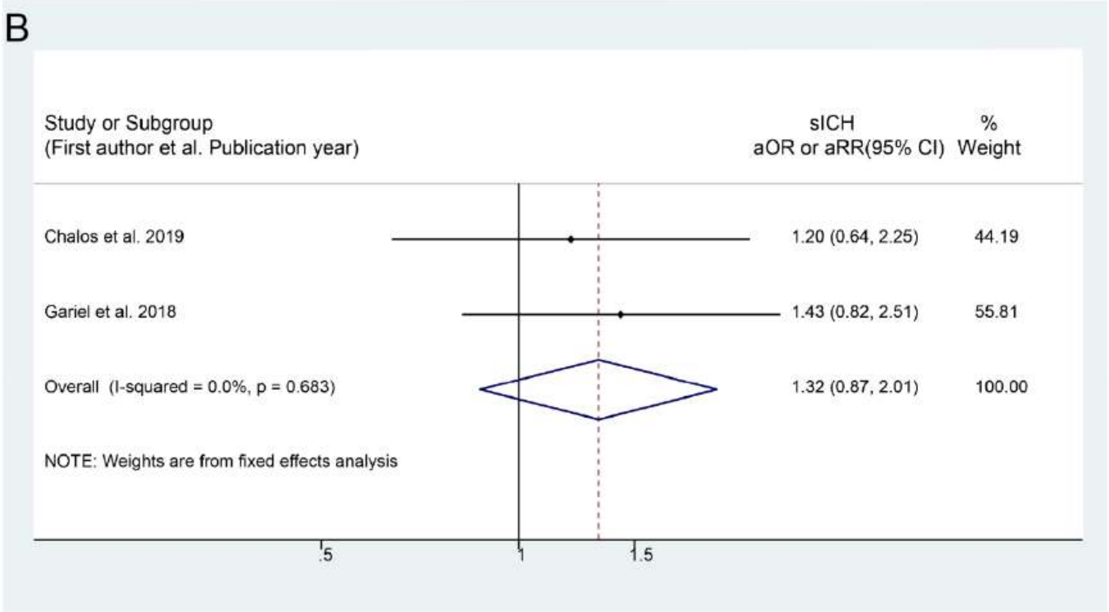

**Supplemental Figure 148.** Sensitivity analysis of the effect of individual RCT studies on the cOR (A) and aOR (B) for sICH, and the publication bias analysis by Begg's funnel plots of cOR (C) and aOR (D). sICH, symptomatic intracranial hemorrhage.

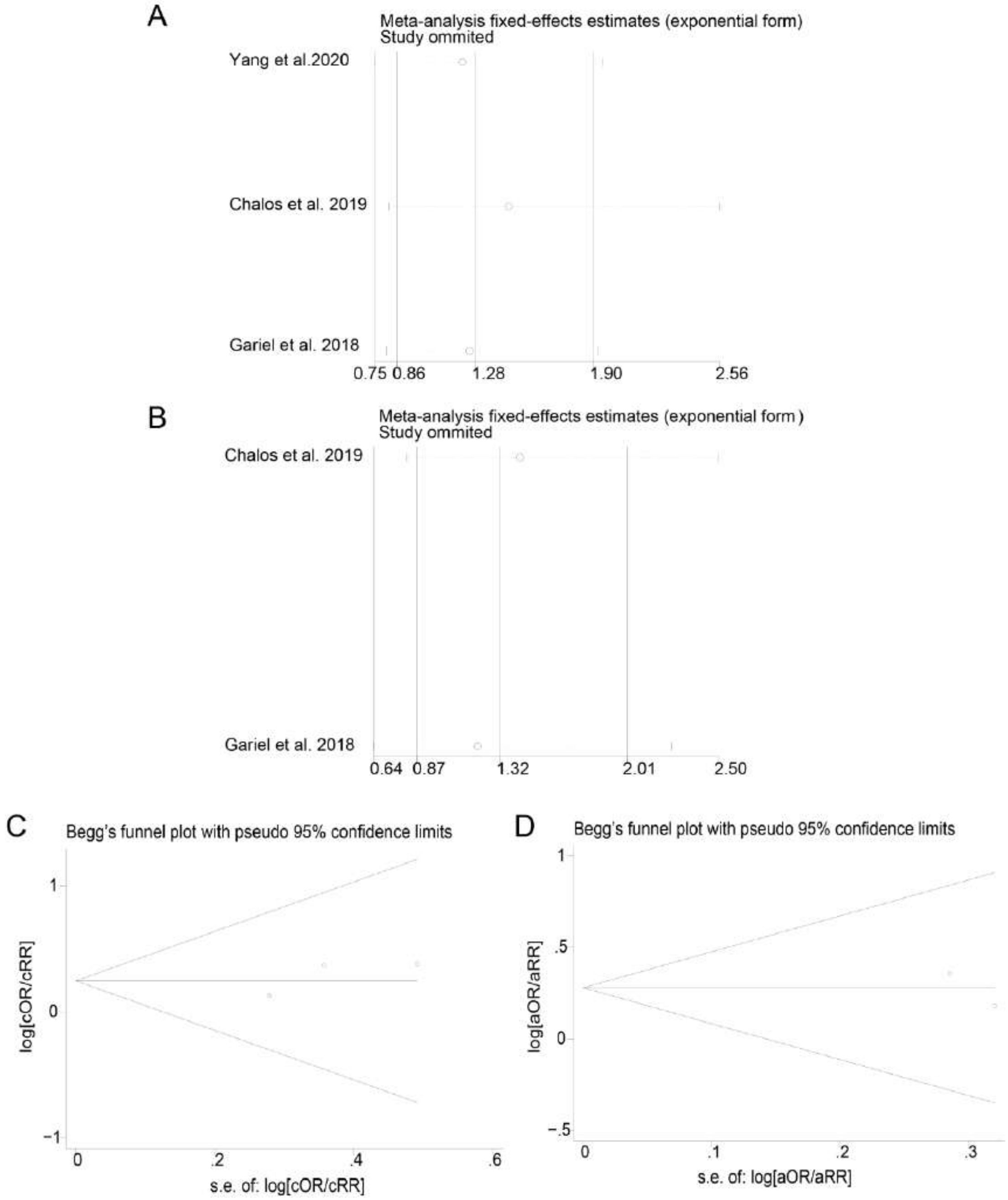

Supplement: Supplementary file 1 [file Data_Sheet_1.PDF]
