# Supplementary material for: Endovascular Treatment With and Without Intravenous Thrombolysis in Large Vessel Occlusions Stroke: A Systematic Review and Meta-Analysis
Source: Front Neurol. 2021 Aug 30;12:697478. doi: 10.3389/fneur.2021.697478 (PMC8437100; doi:10.3389/fneur.2021.697478)
Supplement: Supplementary file 2 [file Data_Sheet_2.docx]

**SUPPLEMENTAL TABLES**

**Supplemental Table 1.** The outcomes and related OR/RR value of each study in this meta-analysis

| **Study** | **Reference**  **group** | **Criteria** | **Outcome** | **Statistical method** | **adjusted OR/RR (95%CI),**  ***P* value** | **unadjusted OR/RR (95%CI), *P* value** |
| --- | --- | --- | --- | --- | --- | --- |
| Yang et al.2020[[1](#_ENREF_1)] | MT | mRS score 0-1 | mRS score 0-1 | LRA and Calculate^*^ | 0.917 (0.629-1.351) | 0.896 (0.624-1.286) |
|  |  | mRS score 0-2 | mRS score 0-2 |  | 1.031 (0.730-1.471) | 1.017 (0.740-1.398) |
|  |  | mRS score 0-3 | mRS score 0-3 |  | 0.8 (0.568-1.123) | 0.841 (0.618-1.144) |
|  |  | mRS score 3-6 | mRS score 3-6 | Calculate^*^ | NA | 0.984 (0.716-1.352) |
|  |  | NA | Death |  | NA | 1.077 (0.725-1.600) |
|  |  | eTICI score 2b-3 | Successful reperfusion | LRA and Calculate^*^ | 1.429 (0.943-2.128) | 1.413 (0.936-2.133) |
|  |  | Heidelberg Bleeding Classification | aICH | Calculate^*^ | NA | 1.133 (0.822-1.563) |
|  |  |  | sICH |  | NA | 1.447 (0.718-2.917) |
|  |  | NA | Infarction in new territory at 5–7 days |  | NA | 0.808 (0.330-1.976) |
|  |  | NA | Pneumonia, aspiration or other |  | NA | 0.899 (0.581-1.392) |
|  |  | NA | Any procedural complication |  | NA | 0.946 (0.613-1.458) |
|  |  | NA | Vessel dissection |  | NA | 0.615 (0.199-1.901) |
|  |  | NA | Embolization into a new territory |  | NA | 0.868 (0.521-1.445) |
| Rajah et al.2020[[2](#_ENREF_2)] | MT | NA | Clot migration | LRA and Calculate^*^ | 19.33 (1.46-252.96), *P* =0.02 | 6.451 (1.495-27.829) |
|  |  | TICI score 2b-3 | Successful recanalization | Calculate^*^ | NA | 1.057 (0.350-3.192) |
| Mohammaden et al.2020[[3](#_ENREF_3)] | MT | NA | Distal clot migration (DCM) | LRA | 5.019 (1.319-19.102), *P* =0.018 | 0.034 (0.004-0.277) |
| Hassan et al.2020[[4](#_ENREF_4)] | EVT | NA | Mortality | LRA and Calculate^*^ | 0.758 (0.432-1.873), *P*=0.520 | 0.835 (0.364-1.918), *P* =0.671 |
|  |  | mRS score 0-2 | Good outcome at discharge |  | 1.536 (0.820-3.145), *P* =0.365 | 0.955 (0.511-1.784), *P* =0.443 |
|  |  | TICI score 2b-3 | Good TICI score |  | 0.954 (0.518-2.269), *P* =0.511 | 0.775 (0.291-2.066), *P* =0.610 |
|  |  | Hemorrhage within 24h associated with NIHSS score ≥4 or leading to death | sICH |  | 1.346 (1.031-1.96), *P*=0.039 | 3.032 (0.966-9.51), *P*=0.048 |
|  |  | Hemorrhage not associated with a decrease of neurological condition | aICH | Calculate^*^ | NA | 2.226 (0.090-55.351) |
|  |  | mRS score 3–6 | Poor outcome at discharge |  | NA | 1.047 (0.560-1.956) |
| Uchikawa et al. 2019[[5](#_ENREF_5)] | MT | Reversible 80% stenosis of the normal vessel diameter after stent retriever use | Vasospasm | Calculate^*^ | NA | 6.50 (1.258-33.578) |
| Wareham et al. 2019[[6](#_ENREF_6)] | MT | TICI score 2b-3 | Recanalisation | BLRA | NA | 0.650 (0.281-1.501), *P*=0.313 |
| Pikija et al. 2019[[7](#_ENREF_7)] | MT | mRS score 0-2 at 3 months | Good outcome | MLRA and Calculate^*^ | 12.045 (1.004-144.392), *P*=0.049 | 6.632 (1.318-33.369) |
| Tajima et al. 2019[[8](#_ENREF_8)] | MT | TICI score 2b-3 | Successful revascularization | Calculate^*^ | NA | 1.898 (0.372-9.682) |
|  |  | TICI score 3 | TICI score 3 |  | NA | 2.668 (1.121-6.352) |
|  |  | ECASS Ⅲ criteria | sICH |  | NA | 0.636 (0.064-6.360) |
|  |  | mRS score 0-2 after 90 d | Good clinical outcome |  | NA | 3.059 (1.209-7.736) |
|  |  | mRS score 6 after 90 d | Death |  | NA | 0.063 (0.004-1.102) |
| Gong et al. 2019[[9](#_ENREF_9)] | MT | TICI score 2b-3 | Successful reperfusion | MLRA with PS and calculate^*^ | 3.333 (0.318-34.989) | 4.393 (0.435-44.416) |
|  |  | NA | ICH |  | 1.818 (0.522-6.331) | 0.745 (0.269-2.066) |
|  |  | mRS score 0-2 at 3 months | Good functional outcome |  | 1.467 (0.434-4.951) | 2.328 (0.901-6.019) |
|  |  | NA | Mortality |  | 2.105 (0.176-25.170) | 1.115 (0.175-7.112) |
| Lee et al.2019[[10](#_ENREF_10)] | EVT | 3-month mRS score of 3-6 despite TICI score 2b-3 | Futile reperfusion | MLRA | 0.75 (0.45-1.26) | 0.83 (0.55-1.25） |
| Kaesmacher  et al. 2019[[11](#_ENREF_11)] | MT | TICI score 2b-3 | Reperfusion success | Calculate^*^ | NA | 1.568(0.697-3.527) |
|  |  | ECASS II criteria | Non-hemorrhagic neurological worsening |  | NA | 0.824(0.320-2.123) |
| Balodis et al. 2019[[12](#_ENREF_12)] | MT | TICI score 2b or greater | Successful reperfusion | Calculate^*^ | NA | 2.011 (0.607-6.664) |
|  |  | NA | Thrombectomy attempts >2 |  | NA | 0.887 (0.391-2.011) |
|  |  | NA | Mortality at 90 days |  | NA | 0.754 (0.326-1.744) |
|  |  | ECASS Ⅱ criteria | sICH |  | NA | 1.261 (0.433-3.677) |
|  |  | Hemorrhagic imbibition with no mass effect and no increase in NIHSS score | aICH |  | NA | 2.368 (0.875-6.412) |
|  |  | mRS score 0-2 | Functional independence | BLRA | NA | 0.48 (0.216-1.07), *P* =0.14 |
|  |  | sICH and aICH | Any bleeding |  | NA | 0.5 (0.221-1.062), *P* =0.05 |
| Meyer et al. 2019[[13](#_ENREF_13)] | MT | mRS score≤2 at 90-day | Good functional outcome | ULRA | NA | 2.27 (0.64-7.98), *P* =0.199 |
| Hassan et al. 2019[[14](#_ENREF_14)] | MT | NA | aICH | BLRA and Calculate^*^ | 1.88 (1.02-3.45), *P*=0.04 | 1.969 (1.108-3.499) |
|  |  | NA | HT | Calculate^*^ | NA | 1.817 (0.993-3.323) |
|  |  | at least 4 points in the NIHSS score worsening within 24 hours post-procedure | sICH |  | NA | 1.961 (0.688-5.592) |
|  |  | mRS score 0-2 | Functional independence |  | NA | 0.989 (0.584-1.675) |
|  |  | mTICI score 2b-3 | Recanalization |  | NA | 1.089 (0.461-2.572) |
|  |  | NA | Death |  | NA | 0.624 (0.352-1.107) |
| Goyal N.et al. 2019[[15](#_ENREF_15)] | MT | mTICI score 2b-3 | SR | ULRA and MLRA | 1.64 (1.03-2.61) , *P*=0.036 | 1.67 (1.07-2.59), *P*=0.023 |
|  |  | mRS score 0-2 at 3-month | Functional independence |  | 1.33 (0.70-2.52), *P*=0.377 | 1.79 (1.16 -2.75), *P*= 0.008 |
|  |  | NA | ≤2 direct pass in patients with SR |  | 14.63 (4.46-48.00), *P*<0.001 | 15.08 (4.95-45.97),  *P* <0.001 |
|  |  | SITS-MOST criteria | sICH | Calculate^*^ | NA | 0.709 (0.334-1.507) |
|  |  | mTICI score 3 | mTICI score 3 |  | NA | 0.739 (0.489-1.118) |
| Casetta et al. 2019[[16](#_ENREF_16)] | MT | mRS score 0-1 at 90 days | mRS score 0-1 at 90 days | MLRA and PS with IPW | 1.3 (0.97-1.8) | 1.32 (1.01-1.7) |
|  |  | mRS score 0-2 at 90 days | mRS score 0-2 at 90 days |  | 1.3 (0.98-1.75) | 1.43 (1.12-1.82) |
|  |  | mRS score 0-3 at 90 days | mRS score 0-3 at 90 days |  | 1.5 (1.14-2) | 1.48 (1.16-1.88) |
|  |  | NA | Death at 90 days |  | 0.6 (0.38-0.99) | 0.65 (0.48-0.9) |
|  |  | TICI score 2b-3 | Successful reperfusion |  | 1.28 (0.95-1.7) | 1.24 (0.96-1.6) |
|  |  | TICI score 3 | TICI score 3 |  | 1.23 (0.93-1.62) | 1.26 (0.99-1.6) |
|  |  | First pass | First pass | MLRA | 1.2 (0.76-1.66) | 1.2 (0.9-1.6) |
|  |  | 1-2 passes | 1-2 passes |  | 1.3 (1.002-1.81) | 1.24 (0.97-1.58) |
|  |  | ECASS II criteria | sICH | MLRA and PS with IPW | 1.62 (0.93-2.1) | 1.54 (0.68-3.5) |
|  |  |  | any HT |  | 0.95 (0.7-1.29) | 1.2 (0.9-1.55) |
|  |  | ECASS I criteria | PH1 | MLRA | 0.6 (0.28-1.5) | 0.76 (0.35-1.7) |
|  |  |  | PH2 |  | 2.7 (0.7-9.8) | 2.8 (0.87-9.8) |
|  |  | NA | Distal embolization |  | 0.98 (0.7-1.6) | 0.9 (0.6-1.4) |
|  |  | NA | Arterial dissection |  | 1.02 (0.4-2.5) | 1.01 (0.42-2.5) |
| Chalos et al. 2019[[17](#_ENREF_17)] | EVT | mRS score 0–2 at 90 d | mRS score 0-2 at 90 d | MLRA | 1.32 (0.85-1.87) | 1.65 (1.25–2.17) |
|  |  | Single pass/use of the device as first line of EVT, complete reperfusion of the large vessel occlusion and its downstream territory (eTICI score 3) and no use of rescue therapy after use of the device | First-pass effect |  | 1.22 (0.79-1.9) | 1.20 (0.83-1.74) |
|  |  | eTICI score ≥2b post-EVT | Successful reperfusion |  | 1.05 (0.77-1.43) | 1.19 (0.92-1.52) |
|  |  | NA | Mortality at 90 d |  | 0.58 (0.40-0.82) | 0.51 (0.40-0.67) |
|  |  | NA (ie, requiring surgery or blood transfusion) | Severe extracranial hemorrhage |  | 1.96 (0.66-5.81) | 1.58 (0.60-4.12) |
|  |  | Heidelberg Bleeding Classification | sICH |  | 1.20 (0.64-2.25) | 1.14 (0.66-1.97) |
| Gamba et al. 2019[[18](#_ENREF_18)] | MT | mRS score 0-1 at 90 days | Good clinical outcome | MLRA and Calculate^*^ | 3.75 (1.09-12.85), *P* =0.03 | 4.115 (1.951-8.680) |
|  |  | mRS score of 0-2 | mRS score of 0-2 |  | NA | 3.43 (1.731-6.797) |
|  |  | NA | Mortality |  | NA | 0.448 (0.147-1.361) |
|  |  | ECASS II criteria | sICH |  | NA | 0.931 (0.319-2.716) |
|  |  | NA | Rates of first-pass success |  | NA | 2.684 (1.371-5.254) |
|  |  | TICI score 2b-3 | Good recanalization |  | NA | 2.846 (1.278-6.336) |
| Aoki et al. 2019[[19](#_ENREF_19)] | EVT | TICI score 2b-3 | Successful recanalization | MLRA | 1.090 (0.378-3.143), *P*=0.874 | 1.572 (0.688-3.589) |
| Goyal N. et al. 2019[[20](#_ENREF_20)] | MT | All infarcts located outside the immediate territory of the vessel implicated in the presenting stroke | Infarct in new territory (INT) | MLRA | 0.75 (0.32-1.76), *P*=0.506 | 0.48 (0.26-0.85), *P*= 0.012 |
|  |  |  |  | ULRA | NA | 0.72 (0.34 -1.50) ,P=0.383 |
|  |  |  |  |  | NA | 0.35 (0.09 -1.26) ,P=0.109 |
| Raychev et al. 2019[[21](#_ENREF_21)] | MT | ECASS III criteria | PH | MLRA | 7.63 (1.52-17.35 ), *P* =0.013 | NA |
| Anadani et al. 2019[[22](#_ENREF_22)] | MT | ECASS II criteria | sICH | MLRA and Calculate^*^ | 0.42 (0.11 - 1.57), *P* =0.196 | 0.622 (0.193-2.000) |
|  |  |  | PH1/2 |  | NA | 0.832 (0.371-1.865) |
|  |  | NA | 90-d mortality |  | 0.43 (0.15 - 1.25), *P* =0.121 | 0.348 (0.149-0.812) |
|  |  | mTICI score 2b-3 | Successful reperfusion |  | 1.12 (0.51 - 2.5), *P* =0.771 | 1.109 (0.545-2.256) |
|  |  | mTICI score 3 | Complete reperfusion |  | NA | 0.874 (0.487-1.567) |
|  |  | mRS score 0-2 at 90 day | Favorable outcome |  | 1.56 (0.75 - 3.23), *P* =0.235 | 1.551 (0.880-2.733) |
|  |  | NA | Procedural complications |  | NA | 0.995 (0.409-2.420) |
| Qureshi et al. 2019[[23](#_ENREF_23)] | MT | Post-treatment TIMI score 3 which is equivalent to TICI score 3 or by Qureshi grade of 0 or an improvement of 1 grade or more on either the TIMI score or Qureshi grading scale) | Partial or complete recanalization | Calculate^*^ | NA | 1.167 (0.359-3.797) |
|  |  | mRS score 0-2 at discharge | Favorable outcome |  | NA | 1.905 (0.690-5.263) |
| Wollenweber et al. 2019[[24](#_ENREF_24)] | MT | mRS score 0-2 at 90 day | Good outcome | MLRA | 1.49(1.08-2.06) | NA |
| Panni et al. 2019[[25](#_ENREF_25)] | MT | mRS score 0-2 at 90 day | Good Outcome | Calculate^*^ | NA | 0.864 (0.490-1.522) |
| Tran et al. 2019[[26](#_ENREF_26)] | MT | mRS score 0-2 at 90 day | Favorable outcome | Calculate^*^ | NA | 5.714 (0.521-62.659) |
|  |  | NA | Death at 90 day |  | NA | 0.102 (0.005-2.123) |
|  |  | ECASS II criteria | ICH |  | NA | 1.875 (0.134-26.320) |
| Candel et al. 2019[[27](#_ENREF_27)] | MT | ECASS II criteria | PH | Calculate^*^ | NA | 0.922 (0.362-2.349) |
|  |  |  | sICH |  | NA | 2.520 (0.555-11.446) |
|  |  |  | SAH |  | NA | 1.065 (0.505-2.246) |
|  |  | mRS score 0-2 at 90 days | Good clinical outcome |  | NA | 0.892 (0.558-1.425) |
|  |  | mRS score >2 at 90 days | mRS score >2 at 90 days |  | NA | 1.080 (0.677-1.724) |
|  |  | NA | Mortality at 90 days |  | NA | 0.625 (0.370-1.056) |
|  |  | mTICI score 2b-3 | Successful or sufficient recanalization |  | NA | 0.715 (0.360-1.418) |
|  |  | mTICI score 3 | mTICI score 3 |  | NA | 1.159 (0.740-1.816) |
| Bücke et al. 2018[[28](#_ENREF_28)] | MT+  extracranial stenting | mRS score 0-2 at day 90 | Good functional outcome | ULRA | NA | 0.94 (0.50-1.75), *P*=0.875 |
|  |  | mRS score 6 | All-cause mortality |  | NA | 0.93 (0.44-2.00), *P*=1 |
|  |  | ECASS Ⅱ criteria | sICH |  | NA | 0.53 (0.06-4.53), *P*=1 |
|  |  | TICI score 2b-3 | TICI score 2b-3 |  | NA | 0.94 (0.35-2.51), *P*=1 |
|  | MT | mRS score 0-2 at day 90 | Good functional outcome |  | NA | 1.36 (1.01-1.83), *P*=0.05 |
|  |  | ECASS Ⅱ criteria | sICH |  | NA | 1.25 (0.65-2.41), *P*=0.495 |
|  |  | TICI score 2b-3 | TICI score 2b-3 |  | NA | 1.26 (0.79-2.01), *P*=0.359 |
|  |  | mRS score 6 | All-cause mortality | MLRA | 0.43 (0.28-0.66) | 0.49 (0.34-0.70), *P*<0.001 |
| Ren et al. 2018[[29](#_ENREF_29)] | MT | Clot having moved from 1 category to another | Clot migration | LRA | NA | 5.79 (2.01-16.69), *P*=0.001 |
|  |  |  | Clot migrated beyond the reach of EVT devices |  | 8.77 (1.66-46.53), *P*=0.011 | NA |
| Anadani et al. 2018[[30](#_ENREF_30)] | MT+IAT | HT and SAH | Any hemorrhage | OR | 0.5 (0.04-3.40) | 0.375 (0.06-1.70) |
|  |  |  |  |  |  |  |
|  |  |  |  |  |  |  |
|  |  |  |  |  |  |  |
|  |  | ECASS Ⅱ criteria | PH2 |  | 4.9 (0.05-392.1) | 4.72 (0.31-69.6) |
| Rocha et al. 2018[[31](#_ENREF_31)] | MT | mRS score 0-2 | Good outcome | MLRA | 2.19 (0.93-5.19) | 1.67 (0.96-2.90) |
|  |  | mRS score 0-1 | Excellent outcome |  | 1.42 (0.59-3.39) | 1.33 (0.76-2.34) |
|  |  | ECASS Ⅲ criteria | Any ICH |  | 0.82 (0.31-2.13) | 1.04 (0.47-2.27) |
|  |  |  | sICH |  | 0.93 (0.17-4.99) | 0.66 (0.17-2.54) |
|  |  | NA | Mortality |  | 0.64 (0.22-1.91) | 0.52 (0.24-1.14) |
|  |  | mTICI score 2b-3 | Successful recanalization | Calculate^*^ | NA | 2.462(0.975-6.213) |
|  |  | mTICI score 3 | mTICI score 3 |  | NA | 1.241(0.724-2.127) |
| Leker et al. 2018[[32](#_ENREF_32)] | EVT | NA | Mortality at 90 days | Calculate^*^ | NA | 0.8 (0.441-1.453) |
|  |  | TICI score 2b-3 | TICI score 2b-3 |  | NA | 0.845 (0.465-1.533) |
|  |  | Excellent outcome was defined as an mRS score 0-1 at discharge and 3 months | mRS score 0-1 at 3 months | MLRA and Calculate^*^ | 1.8 (0.79-4.3) | 1.424 (0.740-2.740) |
|  |  |  | Excellent outcome at hospital discharge |  | 0.7 (0.3-1.5) | 0.709 (0.382-1.314) |
|  |  | 8-point drop in NIHSS score or NIHSS score 0-1 on day 1 following procedure | Major early recovery |  | 0.76 (0.28-2.08) | 0.665 (0.367-1.206) |
|  |  | NA | In-hospital mortality |  | 0.9 (0.36- 2.34) | 0.859 (0.386-1.915) |
|  |  | TICI score 3 at the end of EVT | Favorable target vessel recanalization |  | 0.38 (0.19-0.73) | 0.616 (0.371-1.025) |
| Gariel et al. 2018[[33](#_ENREF_33)] | MT | NA | Use of rescue therapy | Generalized estimating equations models (Poisson distribution, loglink function) | 0.90 (0.61-1.32), *P*=0.58 | 0.850 (0.534-1.353) |
|  |  | mTICI score 3 | Reperfusion at end of procedure |  | 0.82 (0.59-1.13), *P*=0.23 | 0.740 (0.481-1.141) |
|  |  | mTICI score 2b-3 | Reperfusion at end of procedure |  | 1.05 (0.98-1.13), *P*=0.16 | 1.449 (0.825-2.545) |
|  |  | No. of passes >2 | No. of passes >2 |  | 0.97 (0.80-1.18), *P*=0.77 | 0.976 (0.632-1.509) |
|  |  | Arterial perforation and dissection, embolization in a new vascular territory, SAH, and vasospasm | Procedural complications |  | 0.99 (0.64-1.54), *P*=0.96 | 1.089 (0.608-1.949) |
|  |  | NA | Emboli |  | 1.02 (0.75-1.39), *P*=0.91 | 1.015 (0.611-1.688) |
|  |  | mRS score 0-2 at 90-day | Favorable outcome |  | 1.27 (0.95-1.72), *P*=0.11 | 1.908 (1.226-2.969) |
|  |  | mRS score 0-1 at 90-day | Excellent outcome |  | 1.11 (0.78-1.60), *P*=0.55 | 1.490 (0.944-2.350) |
|  |  | NA | 90-d mortality |  | 0.59 (0.39-0.88), *P*=0.009 | 0.451 (0.265-0.765) |
|  |  | Hemorrhagic complication rates (any, PH, or sICH) | Any ICH |  | 1.10 (0.96-1.26), *P*=0.18 | 1.106 (0.721-1.696) |
|  |  | ECASS Ⅲ criteria | PH |  | 1.19 (0.82-1.73), *P*=0.37 | 1.159 (0.632-2.125) |
|  |  | ECASS Ⅲ criteria | sICH |  | 1.43 (0.82-2.51), *P*=0.21 | 1.463 (0.558-3.835) |
| Sallustio et al. 2018[[34](#_ENREF_34)] | MT | TICI score 2b-3 | Successful reperfusion | Calculate^*^ | NA | 1.450 (0.886-2.371) |
|  |  | TICI score 3 | Complete reperfusion |  | NA | 1.621 (1.038-2.532) |
|  |  | Reduction of at least 4 points in the NIHSS score or NIHSS score of 0 | 24-h clinical improvement |  | NA | 1.344 (0.858-2.107) |
|  |  | ECASS Ⅰ criteria | Any ICH |  | NA | 0.943 (0.595-1.493) |
|  |  |  | SAH |  | NA | 2.068 (0.213-20.103) |
|  |  |  | HI1 |  | NA | 0.538 (0.142-2.041) |
|  |  |  | HI2 |  | NA | 0.597 (0.236-1.511) |
|  |  |  | PH1 |  | NA | 1.272 (0.588-2.751) |
|  |  |  | PH2 |  | NA | 1.120 (0.594-2.113) |
|  |  | Hemorrhage associated with NIHSS ≥4 | sICH |  | NA | 0.673 (0.362-1.252) |
|  |  | 3-month mRS score 0-2 | Functional independence |  | NA | 1.311 (0.827-2.079) |
|  |  | 3-month mRS score 0-3 | 3-month mRS score 0-3 |  | NA | 1.276 (0.817-1.991) |
|  |  | NA | 3-month mortality | MLRA | 2 (1.1-3.4), *P*=0.009 | 0.596 (0.368-0.963) |
| DiMaria et al. 2018[[35](#_ENREF_35)] | MT | 90-day mRS score 0-2, or equal to pre-stroke mRS score | Favorable outcome | MLRA with PS | 1.31 (1.02-1.68), *P*=0.036 | 1.61 (1.29-2.01), *P* <0.0001 |
|  |  | 90-day mRS score 0-1, or equal to pre-stroke mRS score | Excellent outcome |  | 1.63 (1.25-2.11), *P* =0.0003 | 1.79 (1.41-2.27), *P* <0.0001 |
|  |  | NA | 90-day mortality |  | 0.83 (0.62,1.11), *P* =0.21 | 0.64 (0.49-0.84), *P*=0.001 |
|  |  | NA | Any ICH |  | 1.08 (0.85,1.39), *P*=0.52 | 0.95 (0.76-1.19), *P*=0.65 |
|  |  | Hemorrhage associated with NIHSS score ≥4 | sICH |  | 1.23 (0.78,1.94), *P* =0.38 | 1.02 (0.68-1.54), *P*=0.91 |
|  |  | mTICI score 2b-3 | Successful reperfusion |  | 1.58 (1.21-2.06), *P*=0.0007 | 1.69 (1.33-2.15), *P* <0.0001 |
|  |  | mTICI score 3 | Excellent reperfusion |  | 1.05 (0.82-1.34), *P* =0.7 | 1.08 (0.87-1.35), *P*=0.47 |
|  |  | NA | Rescue therapy |  | 0.76 (0.56-1.03), *P* =0.08 | 0.96 (0.72-1.28), *P*=0.78 |
|  |  | NA | Emboli to uninvolved territory |  | 0.75 (0.48-1.18), *P* =0.22 | 0.66 (0.44-0.99), *P*=0.044 |
|  |  | NA | Vessel perforation |  | 1.14 (0.64-2.04), *P* =0.65 | 1.13 (0.67-1.9), *P*=0.66 |
|  |  | NA | Number of passes (>2) |  | 0.80 (0.63-1.01), *P* =0.059 | 0.74 (0.60-0.93), *P*=0.008 |
|  |  | NA | Vasospasme |  | 0.66 (0.26-1.68), *P* =0.38 | 0.65 (0.28-1.52), *P*=0.33 |
| Díaz-Pérez et al. 2018[[36](#_ENREF_36)] | MT | NA | sICH | Calculate^*^ | NA | 1.694 (0.528-5.434) |
|  |  | TICI score 2b-3 | Successful reperfusion |  | NA | 0.410 (0.147-1.142) |
|  |  | NA | Intrahospital mortality | MLRA | 0.379 (0.109-1.316), *P* =0.127 | 0.473 (0.226,0.989),  *P* =0.044 |
|  |  | mRS score 0-2 at 3 months | Favorable outcome |  | 1.47 (0.50-4.34), *P* =0.485 | 1.57 (0.74-3.34), *P* =0.244 |
| Maingard et al. 2018[[37](#_ENREF_37)] | MT | mRS score 0-2 at 3 months | Favourable functional outcome | MLRA | 2.17 (1.06-4.44), *P* =0.033 | 2.28 (1.47-3.54), *P* <0.001 |
|  |  | NA | Mortality at 3 months |  | 0.79 (0.36-1.74), *P* =0.551 | 0.41 (0.25-0.69), *P* =0.001 |
|  |  | TICI score 2b or greater | Successful reperfusion |  | 1.22 (0.60-2.50), *P* =0.580 | 2.20 (1.29-3.73), *P* =0.004 |
|  |  | NA | Major complication |  | 1.15 (0.52-2.57), *P* =0.730 | 0.76 (0.41-1.43), *P* =0.42 |
|  |  | Hemorrhage associated with NIHSS score≥4 | sICH |  | 1.40 (0.51-3.83), *P* =0.512 | 0.72 (0.34-1.54), *P* =0.434 |
| Al-Khaled et al. 2018[[38](#_ENREF_38)] | MT | mRS score ≤2 at discharge | Favorable functional outcome | LRA | 4.3 (2.2-8.5), *P* <0.001 | 3.476 (1.934-6.248) |
|  |  | NA | In-hospital mortality |  | 0.74 (0.3-1.9), *P* =0.760 | 0.408 (0.189-0.880) |
|  |  | ECASS III criteria | SICH |  | 0.3 (0.07-1.2), *P* =0.090 | 0.258 (0.065-1.026) |
|  |  | NA | Pneumonia during hospitalization | Calculate^*^ | NA | 1.044 (0.619-1.763) |
| Ferrigno et al. 2018[[39](#_ENREF_39)] | MT | 90-day mRS score 0-2, or equal to pre-stroke mRS score | Favorable outcome | MLRA with PS | 1.76 (1.23-2.55), *P* =0.002 | 1.61 (1.14-2.28), *P* =0.007 |
|  |  | 90-day mRS score 0-1, or equal to pre-stroke mRS score | Excellent outcome |  | 1.77 (1.34-2.34), *P* <0.0001 | 1.69 (1.29-2.23), *P*=0.0001 |
|  |  | NIHSS score 0 at 24 hours or a decrease of ≥8 points at 24 hours | Early neurological improvement (ENI) |  | 1.55 (1.13-2.14), *P* =0.007 | 1.37 (1.01-1.87), *P* =0.044 |
|  |  | NA | 90-d all-cause mortality |  | 0.46 (0.31-0.70), *P* =0.0003 | 0.45 (0.31-0.64), *P* <0.0001 |
|  |  | ECASS Ⅱ criteria | PH |  | 1.33 (0.83-2.14), *P* =0.24 | 1.45 (0.94-2.25), *P* =0.097 |
|  |  |  | sICH |  | 0.81 (0.40-1.63), *P* =0.56 | 1.06 (0.56-1.99), *P* =0.87 |
|  |  | mTICI score 2b-3 | Successful reperfusion |  | 1.30 (1.11-1.53), *P* =0.001 | 1.25 (1.08-1.46), *P* =0.003 |
|  |  | mTICI score 3 | Complete reperfusion |  | 1.05 (0.80-1.39), *P* =0.720 | 1.04 (0.81-1.34), *P* =0.75 |
| Imbarrato et al. 2018[[40](#_ENREF_40)] | MT | TICI score 2b-3 | Successful recanalization | Chi‑square *t* and Fisher’s exact test | NA | 0.713 (0.141–3.612) |
|  |  | mRS score 0-2 at 90 days | Good functional outcome |  | NA | 0.84 (0.26–2.70), *P* =0.8 |
|  |  | NA | ICH |  | NA | 0.16 (0.017–1.5), *P* =0.07 |
| Li et al. 2018[[41](#_ENREF_41)] | EVT | mRS score 0-3 assessed at 3-month | Favorable functional outcome | MLRA | 0.831 (0.682-1.102), *P*=0.006 | 0.225 (0.010-4.875) |
| Wang et al. 2018[[42](#_ENREF_42)] | EVT | mRS score 3-6 | Poor outcome | MLRA | 0.42 (0.19-0.91) | 1.667 (0.944-2.943) |
| Gory et al. 2018[[43](#_ENREF_43)] | MT | mTICI score 2b-3 | Successful reperfusion | MLRA | 1.47 (1.01–2.12),p=0.042 | 1.64 (1.18-2.27), *P* =0.003 |
|  |  | mRS score 0-2 at 90 days | 90-day good outcome | ULRA | NA | 1.32 (0.86-2.010), *P* =0.190 |
| Goyal N. et al. 2018[[44](#_ENREF_44)] | MT | SITS-MOST criteria | sICH | Calculate^*^ | 0.610 (0.267-1.396) | 0.663 (0.368-1.192) |
|  |  | mTICI score 2b-3 | Complete recanalization |  | 1.26 (0.711-2.232) | 1.005 (0.674-1.498) |
|  |  | mRS score 0-1 | Favorable functional outcome |  | 1.320 (0.764-2.280) | 1.259 (0.850-1.863) |
|  |  | mRS score 0-2 | Functional independence | MLRA with PS | 1.75 (1.02-2.99), *P* =0.042 | 1.35 (0.96-1.90), *P* =0.080 |
|  |  | NA | Mortality at 3 months |  | 0.50 (0.26-0.96), *P* =0.037 | 0.75 (0.47-1.13), *P* =0.173 |
| Barral et al. 2018[[45](#_ENREF_45)] | MT | mRS score 0-3 at 3 months | Favorable outcome | MLRA and Calculate^*^ | 2.87 (1.19-6.94), *P* =0.019 | 1.929 (0.900-4.134) |
| Choi et al. 2018[[46](#_ENREF_46)] | MT | mRS score 0-2 at 3 months | Good Functional Outcome | MLRA and Calculate^*^ | 0.567 (0.146-2.197), *P*=0.411 | 1.80 (0.738-4.373) |
|  |  | TICI score 2b-3 | TICI score 2b-3 reperfusions | Calculate^*^ | NA | 0.906 (0.372-2.203) |
|  |  | TICI score 3 | TICI score 3 reperfusion |  | NA | 1.352 (0.516-3.542) |
|  |  | NA | Mortality at 90 days |  | NA | 0.454 (0.122-1.693) |
|  |  | ECASS Ⅱ criteria | sICH |  | NA | 0.878 (0.118-6.556) |
|  |  | Hematoma, Pain, Blood oozing | Groin complications |  | NA | 1.070 (0.299-3.835) |
|  |  | NA | Groin Hematoma |  | NA | 4.639 (0.216-99.711) |
| Gory B. et al. 2018[[47](#_ENREF_47)] | MT | NA | 90-day all-cause mortality | MLRA | 0.298 (0.1-0.893), *P*=0.03 | 0.40 (0.18-0.86), *P* =0.019 |
| Bourcier et al. 2018[[48](#_ENREF_48)] | MT | mRS score 0-2 at 3 months | Favourable outcome | MLRA, PS with IPW and Calculate^*^ | 1.6 (0.7-3.7), *P* =0.29 | 1.501 (0.746-3.023) |
|  |  | NA | Mortality |  | 1.2 (0.5-2.9), *P* =0.66 | 0.448 (0.211-0.949) |
|  |  | NA | ICH |  | 2.3 (0.9-5.9), *P* =0.09 | 2.107 (1.015-4.374) |
|  |  | mTICI score 2b-3 | Successful recanalization | Calculate^*^ | NA | 1.333 (0.596-2.983) |
|  |  | NA | Distal embolism |  | NA | 1.360 (0.613-3.020) |
|  |  | PROACT II criteria | sICH |  | NA | 1.500 (0.569-3.951) |
|  |  | mRS score 3-6 | Poor outcome |  | NA | 0.666 (0.331-1.341) |
| Chu et al. 2018[[49](#_ENREF_49)] | EVT | mRS score 0-2 at 90 days | Favorable outcome | MRA | 2.59 (0.61-10.93), *P* =0.200 | 0.835 (0.307-2.271) |
| Manceau et al. 2018[[50](#_ENREF_50)] | MT | mRS score 0-2 | Good neurological outcome | MLRA | 4.43 (1.04-18.94), *P* =0.04 | 2.521 (0.879-7.228) |
| Kaesmacher et al. 2018[[51](#_ENREF_51)] | MT | mTICI score 2b/3 | Successful recanalization | Calculate^*^ | NA | 2.244 (1.132-4.446) |
|  |  | NA | Inhospital mortality |  | NA | 0.529 (0.196-1.428) |
|  |  | NIHSS score <5 on discharge | Good neurological outcome |  | NA | 1.540 (0.862-2.752) |
|  |  | ΔNIHSS (difference between admission and discharge) score ≥8 | ΔNIHSS score ≥8 |  | NA | 1.723 (0.977-3.039) |
|  |  | ECASS I criteria  ECASS I criteria | HT |  | NA | 0.652 (0.374-1.139) |
|  |  |  | PH1/PH2 |  | NA | 3.569 (0.431-29.522) |
| Rai et al. 2018[[52](#_ENREF_52)] | EVT | TICI score 2b-3 | Recanalization | Calculate^*^ | NA | 2.151 (0.788-5.872) |
|  |  | mRS score 0-2 at 90 days | Favorable outcome |  | NA | 1.375 (0.592-3.194) |
|  |  | ECASS II criteria | Hemorrhage (PH1/PH2) |  | NA | 0.441 (0.044-4.417) |
|  |  | NA | Mortality |  | NA | 0.353 (0.105-1.185) |
| Coutinho et al. 2017[[53](#_ENREF_53)] | MT | mTICI score 2b-3 | Successful reperfusion | MLRA | 0.68 (0.28-1.66) | 0.96 (0.50-1.84) |
|  |  | mTICI score 3 | mTICI score 3 |  | 1.38 (0.76-2.51) | 1.16 (0.72-1.87) |
|  |  | NA | >3 Passes with stent retriever |  | 0.90 (0.44-1.85) | 0.88 (0.49-1.58) |
|  |  | ECASS II criteria | sICH |  | 0.03 (0.00-1.28) | 0.32 (0.06-1.67) |
|  |  | NA | Emboli to uninvolved territory |  | 4.12 (0.75-22.54) | 1.93 (0.49-7.61) |
|  |  | NA | Vasospasm |  | 1.41 (0.58-3.42) | 2.24 (1.20-4.17) |
|  |  | mRS score 0-2 | Good outcomes |  | 1.48 (0.80-2.74) | 1.50 (0.94-2.40) |
|  |  | NA | Mortality |  | 0.90 (0.35-2.30) | 0.64 (0.29-1.37) |
|  |  | NA | SAH | Calculate^*^ | NA | 0.402 (0.072-2.23) |
|  |  | NA | PH1 |  | NA | 0.200 (0.022-1.809) |
|  |  | NA | PH2 |  | NA | 0.406 (0.036-4.524) |
|  |  | NA | HI1 |  | NA | 0.939 (0.519-1.700) |
|  |  | NA | HI2 |  | NA | 1.470 (0.673-3.212) |
|  |  | NA | Romote ICH |  | NA | 2.473 (0.100-61.221) |
|  |  | NA | Vessel perforation |  | NA | 0.271 (0.011-6.709) |
|  |  | NA | Groin hematoma |  | NA | 1.233 (0.203-7.488) |
| Bellwald et al. 2017[[54](#_ENREF_54)] | MT | TICI score 2b-3 | Complete reperfusion | MRA | 0.812 (0.409-1.614) | 0.834 (0.475-1.464) |
|  |  | TICI score 0-1 | TICI score 0-1 |  | 1.686 (0.532-5.340) | 1.458 (0.566-3.758) |
|  |  | ECASS III criteria | sICH |  | 3.723 (0.754-18.378) | 1.990 (0.556-7.129) |
|  |  |  | aICH |  | 2.043 (0.923-4.520) | 1.856 (0.961-3.584) |
|  |  | mRS score 0-2 at 3 months | Functional independence/good outcomes |  | 0.788 (0.453-1.370) | 0.878 (0.559-1.379) |
|  |  | mRS score 0-1 at 3 months | Excellent outcomes |  | 0.739 (0.396-1.380) | 1.050 (0.639-1.726) |
|  |  | NA | Mortality |  | 1.327 (0.726-2.426) | 1.014 (0.613-1.678) |
| Kim et al. 2017[[55](#_ENREF_55)] | EVT | An improvement of 8 points on the NIHSS score, or an NIHSS score of 0 or 1 at 24 h | Early dramatic recovery (EDR) | MLRA | 1.54 (0.449-5.275), *P* =0.493 | 2.375 (0.854-6.605) |
| Uno et al. 2017[[56](#_ENREF_56)] | MT | mRS score 0-2 at 90 days | Favorable outcome | MLRA | 15.45 (2.53-156.35), *P*=0.007 | 7.70 (1.79-40.09),  *P* =0.009 |
| Merlino et al. 2017[[57](#_ENREF_57)] | MT | mRS score 0–2 at 90 day | Favorable outcome at 90 day | MLRA and Calculate^*^ | 3.97 (1.09-14.43), *P* =0.040 | 4.781 (1.564-14.616) |
|  |  | mRS score 0–2 at 1 year | Favorable outcome at 1 year |  | 8.04(1.68-38.40), *P* =0.009 | 5.95 (1.845-19.193) |
|  |  | NA | Mortality at 90-days |  | NA | 0.511 (0.161-1.622) |
|  |  | NA | Mortality at 1-year |  | 6.57 (1.42-30.35), *P* =0.010 | 0.323 (0.110-0.951) |
|  |  | TICI score 2b-3 | Successful recanalization | Calculate^*^ | NA | 2.222 (0.506-9.764) |
|  |  | ECASS Ⅰ and III criteria | ICH |  | NA | 1.55 (0.242-9.940) |
| Wang et al. 2017[[58](#_ENREF_58)] | MT | mTICI score 2b-3 | Successful reperfusion | LRA with PS and Calculate^*^ | 0.392(0.184-0.831) | 0.389(0.209-0.723) |
|  |  | mRS score 0-1 at 90 days | Excellent functional outcome |  | 1.271(0.729-2.213) | 1.070(0.658-1.740) |
|  |  | mRS score 0-2 at 90 days | Good functional outcome |  | 1.195(0.741-1.926) | 1.032(0.679-1.568) |
|  |  | Heidelberg Bleeding Classification | aICH |  | 2.071(1.256-3.414) | 1.762(1.140-2.721) |
|  |  |  | sICH |  | 0.940(0.470-1.878) | 1.111(0.622-1.985) |
|  |  | NA | Mortality |  | 0.925(0.535-1.600) | 0.968(0.601-1.559) |
| Park et al. 2017[[59](#_ENREF_59)] | EVT | mRS score 0-2 at 3 months | Functional independence | MLRA | 1.27 (0.82-1.99), *P* =0.290 | 1.39 (0.96-2.00), *P* = 0.08 |
|  |  | mTICI score 2b-3 | Successful recanalization |  | 1.96 (1.23-3.11), *P* <0.01 | 1.82 (1.22-2.73), *P* <0.01 |
|  |  | mRS score 0-2 at discharge | Functional independence |  | 1.37 (0.86-2.19), *P* =0.190 | 1.53 (1.03-2.27), *P* =0.04 |
|  |  | ECASS III criteria | sHT |  | 0.87 (0.44-1.74), *P* =0.700 | 0.79 (0.44-1.42), *P* =0.42 |
|  |  | NA | Mortality at 3 months |  | 0.58 (0.35-0.97), *P* =0.040 | 0.51(0.33-0.79), *P* <0.01 |
| Wee et al. 2017[[60](#_ENREF_60)] | MT | mTICI score 2b-3 | Complete reperfusion | Calculate^*^ | NA | 0.704 (0.091-5.444) |
|  |  | NIHSS score ≥8 or 0-2 at 24 h | Significant clinical improvement |  | NA | 0.849 (0.276-2.612) |
|  |  | NA | Groin haematoma |  | NA | 2.708 (0.569-12.901) |
|  |  | NA | Pseudoaneurysm |  | NA | 4.317 (0.167-111.322) |
|  |  | ECASS Ⅰ criteria | ICH |  | NA | 0.639 (0.140-2.912) |
|  |  |  | SAH |  | NA | 0.256 (0.012-5.613) |
|  |  |  | PH |  | NA | 1.421 (0.184-10.994) |
|  |  | NA | Clot fragmentation and distal embolization |  | NA | 7.564 (0.344-166.200) |
|  |  | NA | Recurrent stroke |  | NA | 1.400 (0.083-23.737) |
|  |  | NA | Inpatient demise |  | NA | 3.177 (0.524-19.266) |
| Abilleira et al. 2017[[61](#_ENREF_61)] | MT | mRS score 0-2 at 90 Days | Good outcome | LRA with PS, Mantel-  Haenzsel test and Calculate^*^ | 0.97 (0.74-1.27) | 1.077 (0.856-1.356) |
|  |  | Mortality at 90 Days | Mortality at 90 Days |  | 1.07 (0.74-1.54) | 0.913 (0.672-1.239) |
|  |  | NA | Symptomatic Bleedings at 24-36 h |  | 0.56 (0.25-1.27) | 1.263 (0.643-2.482) |
|  |  | mTICI score 2b-3 | Successful recanalization | Calculate^*^ | NA | 1.204 (0.914-1.587） |
| Maier et al. 2017[[62](#_ENREF_62)] | MT | TICI score 2b-3 | Recanalization successful | Calculate^*^ | NA | 2.324 (0.955-5.657) |
|  |  | Hemorrhage within 48 h associated with NIHSS score ≥4 | sICH |  | NA | 0.500 (0.079-3.159) |
|  |  | NA | Any post-interventional ICH |  | NA | 0.857 (0.314-2.337) |
|  |  | mRS score 0-2 on discharge | mRS score 0-2 on discharge |  | NA | 3.328 (1.149-9.634) |
|  |  | mRS score 0-2 90 d | mRS score 0-2 90 d |  | NA | 2.790 (1.022-7.614) |
|  |  | NA | Death (all cause) |  | NA | 0.961 (0.312-2.963) |
| Mundiyanapurath et al. 2017[[63](#_ENREF_63)] | EVT | ECASS Ⅱ or SITS-MOST or NINDS criteria or Heidelberg Bleeding Classification | sICH | MLRA | 0.85 (0.36-2.03), *P* =0.716 | NA |
|  |  | mRS score 0-2 at 90 day | Favorable outcome |  | 1.08 (0.57-2.02), *P* =0.820 | NA |
| Leciñana et al. 2017[[64](#_ENREF_64)] | MT | mRS score 0-2 at 3 months | Independence | MLRA and Calculate^*^ | 2.8 (0.99 -7.98), *P* =0.05 | 2.824 (1.000-7.981) |
|  |  | NA | Mortality at 3 months |  | 0.24 (0.04-1.52), *P* =0.12 | 0.235 (0.036-1.524) |
|  |  | NA | Death or dependence at 3 months | Calculate^*^ | NA | 0.354 (0.125-1.001) |
|  |  | HI1, HI2, PH1 and PH2 | Any ICH |  | NA | 3.088 (0.632-15.075) |
|  |  | SITS-MOST criteria | HI1 |  | NA | 3.909 (0.202-75.837) |
|  |  |  | HI2 |  | NA | 1.200 (0.118-12.233) |
|  |  |  | PH1 |  | NA | 0.784 (0.067-9.139) |
|  |  |  | PH2 |  | NA | 3.909 (0.202-75.837) |
|  |  | significant improvement of >10 points in the NIHSS score at 7 days | Significant improvement |  | NA | 1.238 (0.443-3.457) |
|  |  | NIHSS score 0-1 at 7 days | NIHSS score 0-1 7 days |  | NA | 1.788 (0.566-5.650) |
|  |  | NA | Mortality within 7 days |  | NA | 0.784 (0.067-9.139) |
|  |  | Parenchymal hemorrhage associated with NIHSS score ≥4 or leading to death | sICH |  | NA | 2.980 (0.148-60.211) |
|  |  | TIBI score 4-5 or TICI score 2b-3 | Successful recanalization |  | NA | 2.259 (0.543-9.405) |
| Weber et al. 2017[[65](#_ENREF_65)] | MT | TICI score 2b-3 | Successful recanalization | Calculate^*^ | NA | 0.964 (0.549-1.694) |
|  |  | NA | Embolus in further vessel territory |  | NA | 1.697 (0.504-5.716) |
|  |  | NA | Subarachnoidal hemorrhage |  | NA | 1.037 (0.227-4.733) |
|  |  | NA | Groin hematoma |  | NA | 1.388 (0.192-10.018) |
|  |  | ECASS Ⅲcriteria | sICH |  | NA | 1.697 (0.504-5.716) |
|  |  | sICH and aICH | Any ICH |  | NA | 1.157 (0.586-2.285) |
|  |  | mRS score 0-2 at 3 months | Favorable outcome |  | NA | 0.816 (0.485-1.373) |
|  |  | Mortality at 3 months | Mortality |  | NA | 0.783 (0.449-1.365) |
| Mistry et al.2017[[66](#_ENREF_66)] | MT | Intraparenchymal, subarachnoid, and/or intraventricular hemorrhages within 48 hours after MT | Intracranial hemorrhagic complications | Ordinal regression | NA | 1.52 (0.85-2.71), *P* =0.16 |
| Nogueira et al. 2016[[67](#_ENREF_67)] | MT | mRS score 0-2 at 90-day | Good functional outcome | MLRA and Calculate^*^ | 2.3 (1.2-4.7), *P* =0.02 | 2.002 (1.269-3.158) |
|  |  | mTICI score 2b-3 | mTICI score 2b-3 | Calculate^*^ | NA | 0.969 (0.581-1.617) |
|  |  | mTICI score 3 | mTICI score 3 |  | NA | 1.016 (0.646-1.599) |
|  |  | Hemorrhage within 24 h associated with NIHSS score ≥4 | sICH |  | NA | 1.358 (0.653-2.824) |
|  |  | NA | 90-day mortality |  | NA | 0.620 (0.376-1.021) |
| Angermaier et al. 2016[[68](#_ENREF_68)] | MT | mTICI score 2b or 3 | Revascularization | MLRA and ULRA | 24.92 (1.37-454.9), *P* =0.03 | 11.1 (1.35-91.2), *P* =0.025 |
| Rebello et al. 2016[[69](#_ENREF_69)] | MT | NA | 90-day mortality | MLRA | 0.62 (0.34-1.12) | 0.517 (0.331-0.809) |
|  |  | mRS score 0-2 at 3 month | Good outcome | Calculate^*^ | NA | 1.298 (0.865-1.947) |
|  |  | mTICI score 2b-3 | Successful reperfusion |  | NA | 1.185 (0.717-1.958) |
|  |  | mTICI score 3 | mTICI score 3 |  | NA | 1.216 (0.814-1.817) |
|  |  | ECASS Ⅱ criteria | Any PH |  | NA | 0.519 (0.201-1.344) |
|  |  | NA | SAH |  | NA | 0.861 (0.369-2.012) |
| Broeg-  Morvay et al. 2016[[70](#_ENREF_70)] | MT | TICI score 2b-3 | Immediately complete reperfusion | MLRA with PS and Calculate^*^ | 1.286 (0.319-5.186) | 0.600 (0.217-1.661) |
|  |  | 24 h TIMI score 3 | Complete recanalization |  | 0.278 (0.050-1.531) | 0.219 (0.049-0.974) |
|  |  | PROACT II criteria | sICH |  | 3.162 (0.315-31.775) | 1.832 (0.219-15.337) |
|  |  | PROACT II criteria | aICH |  | 1.235 (0.344-4.431) | 2.254 (0.825-6.164) |
|  |  | NA | Any bleeding |  | 2.149 (0.708-6.530) | 2.673 (1.054-6.780) |
|  |  | mRS score 0-2 after 3 months | Functional independence |  | 0.729 (0.295-1.797) | 1.130 (0.560-2.280) |
|  |  | mRS score 0-1 after 3 months | Excellent clinical outcome |  | 1.130 (0.429-2.978) | 1.244 (0.575-2.689) |
|  |  | NA | Mortality after 3 months |  | 1.333 (0.464-3.828) | 1.426 (0.608-3.346) |
| Behme et al. 2016[[71](#_ENREF_71)] | MT | mTICI score 2b-3 | Successful recanalizations | LRA and Calculate^*^ | 7 (2-26), *P* =0.004 | 4.214 (1.375-12.915) |
|  |  | NA | No. of stent-retriever maneuvers 2 or less | Calculate^*^ | NA | 2.188 (0.881-5.433) |
|  |  | Improvement in NIHSS score (>10 points) | Favorable functional outcome | Calculate^*^ | NA | 1.313 (0.456-3.776) |
| Villwock.et al. 2016[[72](#_ENREF_72)] | MT | NA | Inhospital mortality | BLRA | 0.805 (0.739-0.878), *P* <0.001 | NA |
| Minnerup et al. 2016[[73](#_ENREF_73)] | MT | NA | Death in-hospital/at discharge | Calculate^*^ | NA | 0.651(0.459-0.924) |
|  |  | mRS score 0-1 in-hospital/at discharge | mRS score 0-1 in-hospital/at discharge |  | NA | 1.239 (0.940-1.634) |
|  |  | mRS score 0-2 in-hospital/at discharge | mRS score 0-2 in-hospital/at discharge |  | NA | 1.255 (0.984-1.600) |
|  |  | >8-point NIHSS score improvement from baseline in-hospital/At discharge | >8-point NIHSS score improvement from baseline in-hospital/at discharge |  | NA | 1.359 (1.066-1.733) |
|  |  | NA | Complete or partial recanalization in-hospital/at discharge |  | NA | 1.216 (0.794-1.864) |
|  |  | NA | Death at 3 months |  | NA | 0.586 (0.438-0.784) |
|  |  | mRS score 0-1 at 3 months | mRS score 0-1 at 3 months |  | NA | 1.512 (1.119-2.043) |
|  |  | mRS score 0-2 at 3 months | mRS score 0-2 at 3 months |  | NA | 1.394 (1.076-1.806) |
|  |  | NA | Periprocedural intracerebral hemorrhage |  | NA | 2.237 (0.982-5.095) |
|  |  | NA | Periprocedural subarachnoid hemorrhage |  | NA | 0.660 (0.338-1.287) |
|  |  | NA | Device failure |  | NA | 0.224 (0.062-0.807) |
|  |  | NA | Vessel perforation |  | NA | 0.835 (0.208-3.355) |
|  |  | NA | Intramural arterial dissection |  | NA | 1.848 (0.923-3.700) |
|  |  | NA | Embolization to previously uninvolved territory |  | NA | 1.181 (0.697-2.001) |
|  |  | NA | Intracerebral hemorrhage |  | NA | 1.087 (0.781-1.511) |
| Mulder et al.2016[[74](#_ENREF_74)] | EVT | ECASS Ⅰ criteria | PH2 | Calculate^*^ | NA | 0.880 (0.187-4.138) |
|  |  | ECASS Ⅰ criteria | HI1 |  | NA | 0.452 (0.018-11.345) |
|  |  | ECASS Ⅰ criteria | HI2 |  | NA | 0.452 (0.018-11.345) |
|  |  | NA | SAH |  | NA | 0.757 (0.036-16.144) |
|  |  | NA | Recurrent ischemic stroke |  | NA | 0.466 (0.121-1.802) |
|  |  | Neurological deterioration with an increase of two or more points on the NIHSS, follow-up CT or MRI brain compatible with diagnosis of ischemia, and no other obvious cause for neurological deterioration | Progression of ischemic stroke |  | NA | 0.982 (0.376-2.561) |
| Wang et al. 2015[[75](#_ENREF_75)] | EVT | ECASS II criteria | ICH | BLRA and MLRA | 0.70 (0.29-1.72), *P*=0.44 | NA |
|  |  |  | SICH |  | 0.83 (0.14-4.84), *P*=0.84 | NA |
| Bourcier et al. 2015[[76](#_ENREF_76)] | MT | mRS score≤2 at 3 month | Good outcome | ULRA | NA | 0.8 (0.3-2.1), *P* =0.69 |
| Saposnik et al. 2015[[77](#_ENREF_77)] | MT | mRS score 0-2 | Favorable outcome | Calculate^*^ | NA | 1.159 (0.697-1.927) |
|  |  | mRS score 0-1 | mRS score 0-1 |  |  | 1.167 (0.732-1.861) |
|  |  | mRS score 3-6 | mRS score 3-6 |  |  | 0.895 (0.537-1.491) |
|  |  | NA | Death |  |  | 0.812 (0.256-2.579) |
| Goyal M. et al. 2015[[78](#_ENREF_78)] | MT | mRS score 0-1 | mRS score 0-1 | Calculate^*^ | NA | 0.760 (0.374-1.541) |
|  |  | mRS score 0-2 | mRS score 0-2 |  | NA | 0.769 (0.385-1.536) |
|  |  | mRS score 3-6 | mRS score 3-6 |  | NA | 1.392 (0.697-2.780) |
|  |  | NA | Death |  | NA | 0.288 (0.104-0.803) |
|  |  | TICI score 2b-3 | Successful reperfusion |  | NA | 0.704 (0.312-1.589) |
| Jeromel et al. 2015[[79](#_ENREF_79)] | EVT | mRS score >2 after the procedure | Worse clinical outcome | MLRA | 0.437 (0.140-1.364), *P* =0.154 | NA |
| Guedin et al. 2015[[80](#_ENREF_80)] | MT | Angiographic occlusion in a previously unaffected vascular territory observed on the angiogram after clot removal | Erratic emboli | Calculate^*^ | NA | 0.142 (0.007-2.753) |
|  |  | TICI score 2b-3 | Successful recanalization |  | NA | 10.241 (1.238-84.740) |
|  |  | TICI score 0-2b | No recanalization |  | NA | 0.098 (0.012-0.808) |
|  |  | TICI score 3 | Complete recanalization |  | NA | 4.909 (1.438-16.765) |
|  |  | NIHSS score of 0-1 at 24 hours or a decrease of at least 4 points in the NIHSS score | Early neurologic improvement (NI) |  | NA | 3.316 (1.153-9.540) |
|  |  | mRS score 0-2 at 90-day | Favorable functional outcome |  | NA | 1.910 (0.698-5.229) |
|  |  | SITS-MOST criteria | sICH |  | NA | 1.462 (0.193-11.045） |
|  |  | NA | Number of passes of the thrombectomy device ≤2 |  | NA | 2.455 (0.852-7.072) |
|  |  | NA | 90-Day mortality |  | NA | 0.566 (0.133-2.409) |
| Fujimoto et al. 2015[[81](#_ENREF_81)] | EVT | TICI score 2b-3 | Successful recanalization | MLRA | 8.06 (0.6-108.91), *P* =0.116 | NA |
| Nogueira et al. 2015[[82](#_ENREF_82)] | EVT | ECASS I criteria | HI | BLRA | 1.43 (1.03-2.08), *P* =0.037 | 1.524 (1.142-2.034) |
|  |  |  | PH |  | NA | 0.971 (0.612-1.542) |
|  |  | CT/MRI on the 24-36 h | Any ICH | Calculate^*^ | NA | 1.360 (1.046-1.768) |
| Jankowitz et al. 2015[[83](#_ENREF_83)] | MT | mRS score 0-2 at 90 days | Good outcome | ULRA | NA | 1.6 (0.75 -3.63), *P* =0.2 |
| Jovin et al. 2015[[84](#_ENREF_84)] | MT | mRS score 0-2 | mRS score 0-2 | Calculate^*^ | NA | 1.079 (0.468-2.489) |
|  |  | mRS score 0-1 | mRS score 0-1 |  | NA | 1.002 (0.382-2.632) |
|  |  | mRS score 3-6 | mRS score 3-6 |  | NA | 0.927 (0.402-2.139) |
| Leker et al. 2015[[85](#_ENREF_85)] | MT | mRS score 0-2 | Good outcome | MLRA and Calculate^*^ | 0.39 (0.08-1.9), *P* =0.25 | 0.642 (0.207-1.989) |
|  |  | mRS score 3-6 | mRS 3-6 | Calculate^*^ | NA | 1.558 (0.503-4.830) |
|  |  | NA | Number of passes of the thrombectomy device ≤2 |  | NA | 4.550 (1.126-18.392) |
|  |  | NA | Number of passes of the thrombectomy device 1 |  | NA | 1.488 (0.515-4.296) |
|  |  | TICI score 2b-3 at end of stentriever-based endovascular reperfusion | TICI score 2b-3 at end of stentriever-based endovascular reperfusion (SER) |  | NA | 1.250 (0.268-5.826) |
|  |  | NA | Any ICH |  | NA | 2.306 (0.631-8.429) |
|  |  | NA | sICH |  | NA | 7.444 (0.341-162.466) |
| Brinjikji et al. 2014[[86](#_ENREF_86)] | MT | NA | In-hospital mortality | MLRA and Calculate^*^ | 0.86 (0.59-1.24),  *P* =0.42 | 0.879 (0.639-1.210) |
|  |  | ICD-9 (code 430-432.9) | ICH | Calculate^*^ | NA | 0.457 (0.294-0.709) |
| Spiotta.et al.2014[[87](#_ENREF_87)] | MT | mRS score 0-2 at 90 days | Good outcome | MLRA | 0.67(0.27-1.64), *P* =0.38 | 0.72(0.36 -1.41), *P* =0.33 |
| Mourand et al. 2014[[88](#_ENREF_88)] | MT | mRS score 0-2 | Favorable outcome | ULRA | NA | 0.646 (0.144-2.899),  *P* =0.71 |
| Kass-Hout et al. 2014[[89](#_ENREF_89)] | EVT | TIMI score 2-3 | Successful revascularization | Calculate^*^ | NA | 1.327 (0.480-3.664) |
|  |  | ECASS III criteria | sICH |  | NA | 1.261 (0.359-4.435) |
|  |  | mRS score 0-2 at discharge | Good outcome at discharge |  | NA | 0.855 (0.334-2.185) |
|  |  | Mortality at discharge | Mortality at discharge |  | NA | 0.575 (0.224-1.481) |
|  |  | mRS score 0-2 at 3 months | Good outcome at 3 months |  | NA | 1.140 (0.493-2.636) |
|  |  | mRS score 3-6 at 3 months | Poor outcome at 3 months |  | NA | 0.812 (0.350-1.886) |
| Jankowitz et al. 2012[[90](#_ENREF_90)] | MT | mRS score 0-2 at 90-day | Favorable outcome | ULRA | NA | 0.79 (0.43-1.46), *P* =0.450 |
| Da´valos et al. 2012[[91](#_ENREF_91)] | MT | mTICI score 2b-3 | Complete revascularization | Calculate^*^ | NA | 1.257 (0.497-3.181) |
|  |  | mTICI score 3 | mTICI score 3 |  | NA | 0.965 (0.487-1.914) |
|  |  | NA | No. of retrievals per patient ≤2 |  | NA | 2.437 (1.066-5.573) |
|  |  | NIHSS score ≥ 10-point improvement at discharge | Dramatic improvement at discharge |  | NA | 1.442 (0.732-2.840) |
|  |  | NIHSS score 0-1 at discharge | NIHSS score 0-1 at discharge |  | NA | 6.899 (2.785-17.090) |
|  |  | Death at discharge | Death at discharge |  | NA | 0.705 (0.272-1.824) |
|  |  | mRS score 0–2 at discharge | Favorable functional outcome at discharge |  | NA | 2.832 (1.321-6.073) |
|  |  | NIHSS score ≥ 4-point improvement at 3 months | Good improvement at 3 months |  | NA | 1.809 (0.927-3.531) |
|  |  | NA | Death at 3 months |  | NA | 0.679 (0.299-1.544) |
|  |  | mRS score 0-2 at 3 months | Favorable functional outcomeat 3 months |  | NA | 2.730 (1.378-5.410) |
|  |  | ECASS II criteria | HT |  | NA | 0.661 (0.317-1.380) |
|  |  |  | HI 1 |  | NA | 0.350 (0.114-1.077) |
|  |  |  | HI 2 |  | NA | 2.132 (0.620-7.331) |
|  |  |  | PH 1 |  | NA | 0.347 (0.085-1.410) |
|  |  |  | PH 2 |  | NA | 1.807 (0.319-10.245) |
|  |  |  | SAH |  | NA | 0.867 (0.263-2.852) |
|  |  | SITS-MOST criteria | sICH |  | NA | 0.573 (0.092-3.554) |
|  |  | NA | New ischemic stroke |  | NA | 0.875 (0.119-6.417) |
| Pfefferkorn et al. 2012[[92](#_ENREF_92)] | MT | TIMI score 2-3 | Successful recanalizationwas | Calculate^*^ | NA | 0.879 (0.282-2.738) |
|  |  | mRS score 0-2 at 3 months | mRS score 0-2 at 3 months |  | NA | 4.889 (1.157-20.665) |
|  |  | mRS score 0-3 at 3 months | mRS score 0-3 at 3 months |  | NA | 2.429 (0.725-8.133) |
|  |  | NA | Mortality at 3 months |  | NA | 0.446 (0.122-1.637) |
| Bang et al. 2011[[93](#_ENREF_93)] | EVT | ECASS I criteria | HT | LRA | 2.085 (1.096–3.969), *P*=0.025 | 1.719 (0.989-2.988) |

Abbreviations: NA, not available or not applicable; MT, mechanical thrombectomy; EVT, endovascular treatment; IVT, intravenous thrombolysis; MLRA, multivariate logistic regression analysis; BLRA, Binary logistic regression analysis; ULRA, univariate logistic regression analysis; MRA, multivariate regression analysis; LRA, logistic regression analysis; PS, propensity score matched; IPW, inverse probability of treatment weighting; ICH, intracranial hemorrhage; OR, odds ratio; HR, hazard ratio; 95% CI, 95% confidence interval; NIHSS, National Institutes of Health Stroke Scale; mRS, modified Rankin Scale; TICI, Thrombolysis in Cerebral Infarction; mTICI, modified Thrombolysis in Cerebral Infarction; eTICI, expanded Thrombolysis in Cerebral Infarction; TIMI, Thrombolysis in Myocardial Infarction; ICH, intracranial hemorrhage; SAH, subarachnoid hemorrhage; sICH, symptomatic intracranial hemorrhage; aICH, asymptomatic intracranial hemorrhage; HT, hemorrhagic transformation; PH, parenchymal hematoma; HI, hemorrhagic infarction; cOR, crude odds ratio; cHR, crude hazard ratio; PROACT II, Prolyse in Acute Cerebral Thromboembolism II sStudy; ECASS, European Cooperative Acute Stroke Study; SITS-MOST, Safe Implementation of Thrombolysis in Stroke-Monitoring Study; NINDS, National Institute of Neurological Disease and Stroke (any new ICH on follow-up imaging with any clinical deterioration within the first 7 days for the diagnosis of sICH); ICD-9, International Classification of Diseases 9th edition.

^*^The undajusted OR/RR (95%CI) was calculated by the Review Manager 5.3 Software Package (Nordic Cochrane Centre, Cochrane Collaboration, Copenhagen, Denmark) with the raw data in the enrolled study.

**Supplemental Table 2.** Characteristics of enrolled studies in this meta-analysis (1)

| **Study** | **Registry name/ data source** | **Study type** | **Total Sample Size** | **BT Sample Size** | **Age (Mean±SD/ Median (IQR))** | **Ethnicity** | **NIHSS on admission (Mean±SD/ Median (IQR))** |
| --- | --- | --- | --- | --- | --- | --- | --- |
| Yang et al.2020[[1](#_ENREF_1)] | DIRECT-MT | MC, PC, RCT | 656 | 329 | 69 (61-76)for BT 69 (61-76) for MT | Asian | 17 (14-22) for BT 17 (12-21) for MT |
| Rajah et al.2020[[2](#_ENREF_2)] | Local database | SC, RC | 80 | 26 | 61 (55-66)for BT 66.5 (63-69)for MT mean (95%CI) | Caucasian | 16.96 (13.77-20.16) for BT 14.59 (12.60-16.59) for MT |
| Mohammaden et al.2020[[3](#_ENREF_3)] | NA | SC, RC | 65 | 41 | 70.9 ± 11.7 for DCM^¶^ 65.4 ± 15.7 for non-DCM^¶^ | Caucasian | 19.7 ± 5.8 for DCM^¶^ 17.2 ± 6.6 for non-DCM^¶^ |
| Hassan et al.2020[[4](#_ENREF_4)] | Local database | SC, RC | 189 | 109 | 68.17 ± 14.28 for BT 73.54 ± 9.84 for EVT | Caucasian | 18.26 ± 6.88 for BT 17.67 ± 8.37 for EVT |
| Uchikawa et al.2019[[5](#_ENREF_5)] | NA | SC, RC | 29 | 12 | 78.8 ± 9.6 | Asian | NA |
| Wareham et al.2019[[6](#_ENREF_6)] | NA | SC, PC | 200 | 105 | about 70.5 (mean) | Caucasian | 19 (7.5) |
| Pikija et al. 2019[[7](#_ENREF_7)] | NA | Two Centers, RC | 53 | 37 | 75.9 (64.6-82.6) | Caucasian | 20 (16-23) |
| Tajima et al. 2019[[8](#_ENREF_8)] | NA | SC, RC | 100 | 34 | 74.3 ± 8.6 for BT 75.7 ± 10.6 for MT | Asian | 19.8 ± 4.1 for BT 19.6 ± 6.2 for MT |
| Gong et al. 2019[[9](#_ENREF_9)] | NA | SC, RC | 73 | 42 | 69 ± 9 for BT 71 ± 10 for MT | Asian | 13 (6-21)for BT  15 (6-22)for MT median (range) |
| Lee et al.2019[[10](#_ENREF_10)] | CRCS-5 | MC,PC | 440 | 308 | 67.3 ± 12.3 | Asian | about 14 (10-18) mean (IQR) |
| Kaesmacher et al. 2019[[11](#_ENREF_11)] | BEYOND-SWIFT registry | MC, RC | 193^*^ | 77 | 66.7 ± 14.6 | Caucasian | 5 (4-6) |
|  |  |  | 152^*^ | 64 | NA |  | NA |
| Balodis et al. 2019[[12](#_ENREF_12)] | NA | SC, PC | 146 | 84 | 72 ± 12.5 for BT 72 ± 9.9 for EVT | Caucasian | 15 (12-18) for BT 16.5 (14-20) for MT |
| Meyer et al. 2019[[13](#_ENREF_13)] | NA | MC, PC | 79 | 47 | 92 (90-93) | Caucasian | 17 (13-21) |
| Hassan et al. 2019[[14](#_ENREF_14)] | Local database | SC, RC | 254 | 96 | 69.8 ± 13.29 for BT 71.2 ± 12.63 for MT | Caucasian | 19 (0-36) for BT 16 (0-36) for MT median (range) |
| Goyal N.et al. 2019[[15](#_ENREF_15)] | Local database | SC, RC | 419 | 287 | 63.7 ± 15.1 for BT 64.3 ± 13.9 for MT | Caucasian | 16 (11-20) for BT 16 (10-21) for MT |
| Casetta et al. 2019[[16](#_ENREF_16)] | The Italian Registry of Endovascular Stroke Treatments | MC, RC | 1148 | 635 | 67.6 ± 14.6 for BT 68.8 ± 13.1 for MT | Caucasian | 18 (14-21) for BT 18 (14-22) for MT |
| Chalos et al. 2019[[17](#_ENREF_17)] | MR CLEAN | MC, PC, RCT | 1485 | 1161 | 70 (59-79) for BT 72 (63-80) for EVT | Caucasian | 16 (11-20) for BT 17 (13-20) for EVT |
| Gamba et al. 2019[[18](#_ENREF_18)] | NA | SC, RC | 145 | 70 | 71.9 ± 10.6 for BT 69.1 ± 13.2 for MT | Caucasian | 18 (15-21) for BT 19 (15-20) for MT |
| Aoki et al. 2019[[19](#_ENREF_19)] | NA | SC, PC | 183 | 58 | 76 (68-83) | Asian | 19 (14-24) |
| Goyal N.et al. 2019[[20](#_ENREF_20)] | Local database | SC, RC | 408^†^ | 280 | 63.4 ± 15.1 for no INT^‡^ 66.1 ± 12.6 for INT^‡^ | Caucasian | 16 (11-20) for no INT^‡^ 16 (10-21) for INT^‡^ |
|  |  |  | 339^†^ | NA |  |  |  |
|  |  |  | 69^†^ | NA |  |  |  |
| Raychev et al. 2019[[21](#_ENREF_21)] | STAR,SWIFT,SWIFT PRIME | MC, RC | 389 | 258 | 67.1 ± 12.6 | Caucasian | 16.8 ± 4.7 |
| Anadani et al. 2019[[22](#_ENREF_22)] | TITAN registry | MC, RC | 205 | 125 | 65.5 ± 10 for BT 68.1 ± 11 for MT | Caucasian | 15.6 ± 6 for BT 15.1 ± 7 for MT |
| Qureshi et al. 2019[[23](#_ENREF_23)] | NA | SC, PC | 63 | 27 | 62.8 ± 15.2 for BT 65.9 ± 15.4 for MT | Caucasian | 0-42 (range) |
| Wollenweber et al. 2019[[24](#_ENREF_24)] | GSR-ET | MC, PC | 2637 | 1457 | 75 (64-82) | Caucasian | 15 (10-19) |
| Panni et al. 2019[[25](#_ENREF_25)] | ETIS | MC, RC | 216 | 126 | 65.7 ± 14.8 | Caucasian | 20 (16-23) |
| Tran et al.2019[[26](#_ENREF_26)] | NA | SC, RC | 22 | 5 | 62.3 ± 16.6 | Asian | 17.5 ± 5.4 |
| Candel et al. 2019[[27](#_ENREF_27)] | NA | SC, RC | 348 | 122 | 75.1 ± 12.3 for BT 75.4 ± 12.6 for MT | Caucasian | 17 (12-21) for BT 18 (10-21) for MT |
| Bücke et al. 2018[[28](#_ENREF_28)] | NA | SC, PC | 222^#^ | 53 | 67.8 ± 12.8 | Caucasian | 14.8 ± 8.1 |
|  |  |  | 849^#^ | 269 | 73.1 ± 12.9 |  | 16.5 ± 7.6 |
| Ren et al. 2018[[29](#_ENREF_29)] | NA | SC, RC | 314 | 209 | 71 (60-79) for BT 69 (56-77) for MT | Caucasian | 14 (9-19) for BT 14 (8-19) for MT |
| Anadani et al. 2018[[30](#_ENREF_30)] | NA | SC, PC | 67 | 13 | 66.1 ± 15.0 | Caucasian | 15.2 ± 7.6 |
| Rocha et al. 2018[[31](#_ENREF_31)] | NA | SC, PC | 234 | 152 | 70.90 ± 12.60 for BT 71.93 ± 13.67 for MT | Caucasian | 16.28 ± 4.98 for BT 15.67 ± 4.48 for MT |
| Leker et al. 2018[[32](#_ENREF_32)] | NASIS-REVASC | MC, PC | 270 | 159 | 68.1 ± 1 for BT 67.4 ± 15.5 for MT | Caucasian | 16 (13-19) for BT 16 (12-20) for MT |
| Gariel et al. 2018[[33](#_ENREF_33)] | ASTER | MC, PC, RCT | 381 | 250 | 68.7 ± 14.8 for BT  72.2 ± 13.0 for MT | Caucasian | 17.0 (12.5-20.0) for BT 18.0 (11.0-21.0) for MT |
| Sallustio et al. 2018[[34](#_ENREF_34)] | NA | SC, RC | 325 | 193 | 71.8 ± 14.2 for BT 70.3 ± 12.9 for MT | Caucasian | 19 (2-26) for BT 19 (3-25) for MT median (range) |
| DiMaria et al. 2018[[35](#_ENREF_35)] | ETIS registry | MC, PC | 1,507 | 976 | 67.2 ± 15.0 for BT 67.6 ± 15.1 for MT | Caucasian | 17 (11-20) for BT 16 (11-21) for MT |
| Díaz-Pérez et al. 2018[[36](#_ENREF_36)] | NA | SC, RC | 153 | 76 | 71 (29) | Caucasian | 19 (7) for BT 17.5 (7) for MT |
| Maingard et al. 2018[[37](#_ENREF_37)] | ESCAPE and others | MC, RC | 355 | 210 | 67 ± 14 | Caucasian | 17 ± 5 for BT 17 ± 6 for MT |
| Al-Khaled et al. 2018[[38](#_ENREF_38)] | NA | SC, PC | 236 | 144 | 69.0 ± 13 | Caucasian | 13 (10-17) |
| Ferrigno et al. 2018[[39](#_ENREF_39)] | NA | SC, PC | 485 | 348 | 68 (median) | Caucasian | 16.2 ± 5.9 for BT 16.1 ± 5.1 for MT |
| Imbarrato et al. 2018[[40](#_ENREF_40)] | NA | Two Centers, RC | 46 | 23 | 69.2 ± 14.7 for BT 71.1 ± 16.2 for EVT | Caucasian | 17.6 for BT 16.6 for MT (mean) |
| Li et al. 2018[[41](#_ENREF_41)] | Local database | SC, RC | 68 | 66 | 57.9 ± 11.8 | Asian | 24.5 (15-30) |
| Wang et al. 2018[[42](#_ENREF_42)] | ACTUAL registry | MC, RC | 199 | 85 | 64 (55-72) | Asian | 16 (13-21) |
| Gory et al. 2018[[43](#_ENREF_43)] | TITAN study | MC, RC | 395 | 251 | 63.4 ± 12.3 | Caucasian | 15.8 ± 5.9 |
| Goyal N. et al. 2018[[44](#_ENREF_44)] | Local database | MC, RC | 569 | 292 | 62.5 ± 17.0 for BT 61.0 ± 19.8 for MT | Caucasian | 17 (13-21) for BT 16 (12-21) for MT |
| Barral et al. 2018[[45](#_ENREF_45)] | NA | Two centers, RC | 169 | 130 | 82 (82-86) | Caucasian | 18 (13-21) |
| Choi et al. 2018[[46](#_ENREF_46)] | NA | SC, RC | 81 | 43 | 68.9 ± 12.8 for BT 72.6 ± 14.1 for MT | Asian | 13 (10-16) for BT 15 (11-17) for MT |
| Gory B. et al. 2018[[47](#_ENREF_47)] | ETIS registry | MC, RC | 117 | 54 | 62.9 (16.2) for alive 67.7 (12.9) for dead | Caucasian | 12 (8-21) for alive 22 (14-41) for dead |
| Bourcier et al. 2018[[48](#_ENREF_48)] | NA | MC, RC | 141 | 85 | 68 ± 15 for BT 73 ± 15 for MT | Caucasian | 18 (14-22) for BT 18 (16-23) for MT |
| Chu et al. 2018[[49](#_ENREF_49)] | NTUH stroke registry | SC, RC | 65 | 33 | 71.9 ±12.4 | Asian | 19 (15-26) |
| Manceau et al. 2018[[50](#_ENREF_50)] | NA | SC, RC | 82 | 54 | 64.6 ± 14.4 | Caucasian | 18.4 ± 5.4 |
| Kaesmacher et al. 2018[[51](#_ENREF_51)] | NA | SC, RC | 239 | 160 | 69.8 ± 15.5 for BT 73.3 ± 12.4 for MT | Caucasian | 15 (11-17) for BT 15 (11-18) for MT |
| Rai et al. 2018[[52](#_ENREF_52)] | NA | SC, RC | 90 | 38 | 63 ± 19 for BT 69 ± 18 for EVT | Caucasian | 18 (13-23) for BT 16 (10-22) EVT |
| Coutinho et al.2017[[53](#_ENREF_53)] | SWIFT and STAR studies | MC, PC | 291 | 160 | 67 ± 13 for BT  69 ± 12 for MT | Caucasian | 17 (13-20) |
| Bellwald et al. 2017[[54](#_ENREF_54)] | Essen and Bernese stroke registries | MC, RC | 360 | 249 | 73 ± 14 for BT 75 ± 15 for MT | Caucasian | 16 (1-36) for BT 15 (0-38) for MT median (range) |
| Kim et al. 2017[[55](#_ENREF_55)] | NA | SC, RC | 102 | 77 | 64.3 ± 10.4 | Asian | 14 (10-17) |
| Uno et al. 2017[[56](#_ENREF_56)] | NA | SC, PC | 34 | 18 | 72 (66-77) | Asian | 29 (14-33) |
| Merlino et al. 2017[[57](#_ENREF_57)] | NA | SC, PC | 66 | 33 | 69.6 ± 12.7 for BT 70.8 ± 12.2 for MT | Caucasian | 17.5 (14-20) for BT 20 (14-23) for MT |
| Wang et al. 2017[[58](#_ENREF_58)] | ACTUAL | MC, RC | 263 | 160 | 67 (59-73) for BT 66 (57-75) for MT | Asian | 17 (13.25-21.75) for BT 16 (12-21) for MT |
| Park et al. 2017[[59](#_ENREF_59)] | CRCS-5 registry | MC, RC | 639 | 458 | 68 ± 12 for BT 69 ± 12 for EVT | Asian | 15 (11-19) for BT 15 (10-18.5) for EVT |
| Wee et al. 2017[[60](#_ENREF_60)] | NA | SC, RC | 50 | 21 | 73 ± 16 for BT 71 ± 14 for MT | Caucasian | 15 ± 5 for BT 15 ± 7 for MT |
| Abilleira et al. 2017[[61](#_ENREF_61)] | SONIIA registry | MC, PC | 1166 | 567 | 68.6 ± 12.8 for BT 68.1 ± 13.5 for MT | Caucasian | 17 (13–20) for BT 17 (12-20) for MT |
| Maier et al. 2017[[62](#_ENREF_62)] | NA | SC, PC | 109 | 81 | 75 (62-80) for BT 76 (62.8-79.8) for MT | Caucasian | 17 (10.5-20.5) for BT 12.5 (8.3-18) for MT |
| Mundiyanapurath et al. 2017[[63](#_ENREF_63)] | Local database | SC, RC | 435 | NA | 72 (62-79) | Caucasian | 17 (14-20) |
| Leciñana et al. 2017[[64](#_ENREF_64)] | FUN-TPA | MC, PC | 131 | 53 | 64 (51-73) for BT 74 (66-78) for MT | Caucasian | 17 (14-22) for BT 19 (13-22) for MT |
| Weber et al. 2017[[65](#_ENREF_65)] | NA | SC, PC | 250 | 105 | 70.2 ± 12.6 for BT 69.3 ± 14.9 for MT | Caucasian | 15.5 (12-20) for BT 15 (9-19) for MT |
| Mistry et al.2017[[66](#_ENREF_66)] | NA | MC, RC | 228 | 119 | 65.8 ± 14.3 | Caucasian | 16.3 ± 7.1 |
| Nogueira et al. 2016[[67](#_ENREF_67)] | NASA registry | MC, RC | 315 | 136 | 67.3 ± 16 for BT 67.7 ± 14 for MT | Caucasian | 18.5 ± 7 for BT 17.8 ± 6 for MT |
| Angermaier et al. 2016[[68](#_ENREF_68)] | NA | SC, RC | 63 | NA | 73 (62-77) | Caucasian | 13 (9-19) |
| Rebello et al. 2016[[69](#_ENREF_69)] | Local database | SC, RC | 422 | 253 | 66 (53-78) for BT 72 (57-79) for MT | Caucasian | 20 (15-23) for BT 19 (15-24) for MT |
| Broeg-Morvay et al. 2016[[70](#_ENREF_70)] | Local database | SC, RC | 196 | 156 | 73 ± 14 for BT 77 ± 14 for MT | Caucasian | 15 (2-36) for BT 17 (4-38) for MT median (range) |
| Behme et al. 2016[[71](#_ENREF_71)] | Local databases | Two centers, RC | 93 | 66 | 74 (32-91) for BT 74 (48-91) for MT median (range) | Caucasian | 16 ± 6 for BT 17 ± 8 for MT |
| Villwock.et al. 2016[[72](#_ENREF_72)] | Database (NIS,HCUP, Agency for Healthcare Research and Quality) | MC, RC | 16307 | 8941 | 68 (median) | Caucasian | NA |
| Minnerup et al. 2016[[73](#_ENREF_73)] | REVASK registry | MC, PC | 1107 | 603 | 68.3 ± 13.7 for BT 68.7 ± 14.7 for MT | Caucasian | 15.1 ± 6.4 for BT 14.3 ± 6.7 for MT |
| Mulder et al.2016[[74](#_ENREF_74)] | MR CLEAN | MC, PC, RCT | 233 | 203 | 65.4 (54.3-76.2)for BT 67.5 (61.5-77.8) for EVT | Caucasian | 18 (14–22)for BT 19 (14–22) for EVT |
| Wang et al. 2015[[75](#_ENREF_75)] | Local database | SC, RC | 193 | NA | 67 (56-75) | Caucasian | 17 (11-22) |
| Bourcier et al. 2015[[76](#_ENREF_76)] | NA | SC, RC | 73 | 40 | 59(25-85) median(range) | Caucasian | 18 (2-27) median (range) |
| Saposnik et al. 2015[[77](#_ENREF_77)] | NINDS tPA Stroke Studies (Parts I and II), SWIFT, STAR | MC, RC | 291 | 160 | 66.8 ± 13.2 for BT 69.2 ± 12.1 for MT | Caucasian | 16.8 ± 4.6 for BT 17.0 ± 4.9 for MT |
| Goyal M.et al. 2015[[78](#_ENREF_78)] | ESCAPE trial | MC, PC, RCT | 165 | 120 | 71 (60-81) | 144 for Caucasian 21 for Asian | 16 (13-20) |
| Jeromel et al. 2015[[79](#_ENREF_79)] | (IMS)III trial | SC, RC | 134 | 70 | 64.6 ± 11.7 | Caucasian | 15 (11-19) |
| Guedin et al. 2015[[80](#_ENREF_80)] | NA | MC, RC | 68 | 28 | 69.2 ± 13.5 for BT 64.6 ± 15.3 for MT | Caucasian | 18 (13-19) for BT 15 (10-20) for MT |
| Fujimoto et al. 2015[[81](#_ENREF_81)] | NA | SC, RC | 25 | 9 | 73 ± 14 | Caucasian | 20 ± 6 |
| Nogueira et al. 2015[[82](#_ENREF_82)] | NA | MC, RC | 1122 | 371 | 67 ± 15 | Caucasian | 17 ± 5 |
| Jankowitz et al. 2015[[83](#_ENREF_83)] | Local database | SC, RC | 112 | 46 | 66 (29-93) | Caucasian | 17 (4-36) |
| Jovin et al. 2015[[84](#_ENREF_84)] | REVASCAT | MC, PC, RCT | 103 | 70 | 65.7 ± 11.3 | Caucasian | 17.0 (14.0-20.0) |
| Leker et al. 2015[[85](#_ENREF_85)] | NA | SC, PC | 57 | 24 | 66.8 ± 13.7 for BT 64.4 ± 14.7 for MT | Caucasian | 19.2 ± 5.1 for BT 19.1 ± 5.8 for MT |
| Brinjikji et al. 2014[[86](#_ENREF_86)] | NIS | MC, RC | 631 | 340 | 58.7 ± 34.6 | Caucasian | NA |
| Spiotta.et al.2014[[87](#_ENREF_87)] | Local database | SC, RC | 158 | 64 | 66.4 ± 15.2 | Caucasian | 15.9 ± 5.9 |
| Mourand et al. 2014[[88](#_ENREF_88)] | NA | SC, PC | 31 | 19 | 61.2 ± 16.9 | Caucasian | 14 (7-38) |
| Kass-Hout et al. 2014[[89](#_ENREF_89)] | NA | SC, RC | 104 | 42 | 67.64 ± 14.85 for BT 69.26 ± 15.76 for EVT | Caucasian | 14.78 ± 4.7 for BT 16 ± 5.37 for EVT |
| Jankowitz et al. 2012[[90](#_ENREF_90)] | Local database | NA, RC | 191 | 61 | 67 (55-75) | Caucasian | 16 (12-20) |
| Da´valos et al. 2012[[91](#_ENREF_91)] | NA | MC, RC | 141 | 74 | 66.2 ± 12.7 for BT 66.4 ± 13.6 for MT | Caucasian | 17 (11-21) for BT 18 (15-20) for MT |
| Pfefferkorn et al. 2012[[92](#_ENREF_92)] | NA | SC, RC | 65 | 35 | 64.6 ± 12.9 for MT 62.17 ± 14.4 for BT | Caucasian | 19.6 ± 6 for MT 19 ± 7.1 for BT |
| Bang et al. 2011[[93](#_ENREF_93)] | NA | Two centers, PC | 222 | 80 | 65.2 ± 16.4 | Caucasian and Asian | 16.6 ± 6.6 |

Abbreviations: BT, bridging therapy; SD, standard deviation; IQR, interquartile range; NA, not available or not applicable; MT, mechanical thrombectomy; EVT, endovascular treatment; NIHSS, National Institutes of Health Stroke Scale; MC, multicenter; SC, single center; PC, prospective cohorts; RC, retrospective cohorts; RCT, randomized controlled trial; TICI, thrombolysis in cerebral infarction; sICH, symptomatic intracranial hemorrhage; NASCET, North American Symptomatic Carotid Endarterectomy Trial; ICA, internal carotid artery; MCA, middle cerebral artery.

^*^The sample size 193 was enrolled to evaluate the outcome of reperfusion success (TICI2b/3), and the sample size 152 was enrolled for the outcome of non-hemorrhagic neurological worsening (drop in the NIHSS ≥ 4 between admission NIHSS and 24 h NIHSS without the occurrence of sICH).

^#^ The 222 participants was admitted with an occlusion or high-grade stenosis (NASCET>70%) of the extracranial ICA with a concomitant ipsilateral occlusion of the intracranial ICA, the carotid-T or the MCA (M1 or M2 branch). The sample size 849 was admitted with acute intracranial vessel occlusion without concomitant ipsilateral ICA-occlusion or high-grade stenosis.

†The 408 participants was admitted with emergent large vessel occlusion, 339 participants was admitted with anterior circulation large vessel occlusions and 69 participants was admitted with posterior circulation large vessel occlusions.

^‡^INT: infarct in new territory (All infarcts located outside the immediate territory of the vessel implicated in the presenting stroke).

^¶^DCM: distal clot migration.

**Supplemental Table 3.** Characteristics of enrolled studies in this meta-analysis (2)

| **Study** | **IVT (dose)** | **Device(stent retrievers)** | **Time from onset to treatment (hours)** | **Location of occluded artery** | **Follow-up time** |
| --- | --- | --- | --- | --- | --- |
| Yang et al.2020[[1](#_ENREF_1)] | Alteplase (0.9 mg/kg) | NA | within 4.5 h | anterior circulation | 90 day |
| Rajah et al.2020[[2](#_ENREF_2)] | NA | NA | within 4.5 h | anterior and posterior circulation | 24h |
| Mohammaden et al.2020[[3](#_ENREF_3)] | NA | Solitaire FR | NA | anterior circulation | post thrombectomy |
| Hassan et al.2020[[4](#_ENREF_4)] | tPA (NA) | NA | within 4.5 h | anterior and posterior circulation | discharge |
| Uchikawa et al. 2019[[5](#_ENREF_5)] | tPA (NA) | Solitaire, Trevo and Revive | within 8 h and more than 8 h with large diffusion-perfusion mismatch in MRI | anterior and posterior circulation | NA |
| Wareham et al. 2019[[6](#_ENREF_6)] | rt-PA (NA) | NA | NA | anterior circulation | NA |
| Pikija et al. 2019[[7](#_ENREF_7)] | tPA (NA) | NA | within 6 h | anterior circulation tandem lesion | 90 day |
| Tajima et al. 2019[[8](#_ENREF_8)] | Alteplase (0.6 mg/kg) | Trevo | within 4.5 h | anterior circulation | 90 day |
| Gong et al. 2019[[9](#_ENREF_9)] | NA | Solitaire AB | NA | anterior circulation | 90 day |
| Lee et al. 2019[[10](#_ENREF_10)] | NA | NA | within 12 h for EVT | anterior circulation | 90 day |
| Kaesmacher et al. 2019[[11](#_ENREF_11)] | NA | Solitaire FR | NA | anterior circulation | 90 day |
| Balodis et al. 2019[[12](#_ENREF_12)] | rt-PA (0.9 mg/kg) | NA | within 4.5 h for IVT and 8 h for EVT | anterior circulation | 90 day |
| Meyer et al. 2019[[13](#_ENREF_13)] | NA | NA | NA | anterior circulation | 90 day |
| Hassan et al. 2019[[14](#_ENREF_14)] | tPA (NA) | NA | NA | anterior and posterior circulation | 90 day |
| Goyal N.et al. 2019[[15](#_ENREF_15)] | tPA (0.9 mg/kg) | Solumbra | NA | anterior and posterior circulation | 90 day |
| Casetta et al. 2019[[16](#_ENREF_16)] | NA (0.9 mg/kg, maximum 90 mg) | NA | within 4.5 h for IVT and 6 h for MT | anterior circulation | 90 day |
| Chalos et al. 2019[[17](#_ENREF_17)] | Alteplase (0.9 mg/kg over 1 hour with 10% initial bolus) | NA | within 6.5 h | anterior circulation | 90 day |
| Gamba et al. 2019[[18](#_ENREF_18)] | rt-PA (0.9 mg/kg) | Solitaire and Trevo | NA | anterior circulation | 90 day |
| Aoki et al. 2019[[19](#_ENREF_19)] | NA | Trevo ProVue, Solitaire, Revive Self Expanding and Merci | NA | anterior circulation | discharge |
| Goyal N. et al. 2019[[20](#_ENREF_20)] | tPA (NA) | Solumbra | NA | anterior and posterior circulation^‡^ | 90 day |
|  |  |  |  | anterior circulation^‡^ |  |
|  |  |  |  | posterior circulation^‡^ |  |
| Raychev et al. 2019[[21](#_ENREF_21)] | tPA (NA) | Solitaire | NA | anterior circulation | 90 day |
| Anadani et al. 2019[[22](#_ENREF_22)] | NA | NA | NA | anterior circulation | 90 day |
| Qureshi et al. 2019[[23](#_ENREF_23)] | rt-PA (NA) | Solitaire and Trevo | within 6 h | anterior and posterior circulation | discharge |
| Wollenweber et al. 2019[[24](#_ENREF_24)] | Alteplase (NA) | NA | NA | anterior and posterior circulation | 90 day |
| Panni et al. 2019[[25](#_ENREF_25)] | NA | NA | within 8 h | anterior circulation | 90 day |
| Tran et al.2019[[26](#_ENREF_26)] | NA | Solitaire and Solumbra | NA | posterior circulation | 90 day |
| Candel et al. 2019[[27](#_ENREF_27)] | Alteplase (0.9 mg/kg) | pREset | NA | anterior and posterior circulation | 90 day |
| Bücke et al. 2018[[28](#_ENREF_28)] | NA | NA | within 6 h | anterior circulation tandem lesion^#^ | 90 day |
|  |  |  |  | anterior circulation^#^ |  |
| Ren et al. 2018[[29](#_ENREF_29)] | NA | NA | within 4.5 h | anterior circulation | post thrombectomy |
| Anadani et al. 2018[[30](#_ENREF_30)] | IA-tPA (3-5 mg) (maximum of 15 mg) | NA | NA | anterior and posterior circulation | 90 day |
| Rocha et al. 2018[[31](#_ENREF_31)] | NA | NA | NA for MT 2.25 (1.45) for BT (median (IQR)) | anterior circulation | 90 day |
| Leker et al. 2018[[32](#_ENREF_32)] | tPA (NA) | NA | within 4 h | anterior circulation | 90 day |
| Gariel et al. 2018[[33](#_ENREF_33)] | NA | NA | within 6 h | anterior circulation | 90 day |
| Sallustio et al. 2018[[34](#_ENREF_34)] | rt-PA (0.9 mg/kg) | NA | within 4.5 h | anterior circulation | 90 day |
| DiMaria et al. 2018[[35](#_ENREF_35)] | NA | NA | within 6 h | anterior circulation | 90 day |
| Díaz-Pérez et al. 2018[[36](#_ENREF_36)] | rt-PA (0.9 mg/kg) | NA | within 4.5 h | anterior circulation | 90 day |
| Maingard et al. 2018[[37](#_ENREF_37)] | Alteplase (NA) | NA | within 12 h | anterior circulation | 90 day |
| Al-Khaled et al. 2018[[38](#_ENREF_38)] | rt-PA (0.9 mg/kg) | Trevo, Penumbra System, Solitaire, ReViveSE and Penumbra 3D Separator | within 4.5 h | anterior circulation | discharge |
| Ferrigno et al. 2018[[39](#_ENREF_39)] | rt-PA (0.9 mg/kg) | NA | within 4.5 h for IVT and 8 h for MT | anterior circulation | 90 day |
| Imbarrato et al. 2018[[40](#_ENREF_40)] | tPA (NA) | NA | within 8 h | anterior circulation | 90 day |
| Li et al. 2018[[41](#_ENREF_41)] | NA | Solitaire AB or FR | within 24 h | posterior circulation | 90 day |
| Wang et al. 2018[[42](#_ENREF_42)] | NA | NA | NA | anterior circulation | 90 day |
| Gory et al. 2018[[43](#_ENREF_43)] | tPA (NA) | NA | NA | anterior circulation tandem lesion | 90 day |
| Goyal N. et al. 2018[[44](#_ENREF_44)] | tPA (0.9 mg/kg) | NA | NA | anterior and posterior circulation | 90 day |
| Barral et al. 2018[[45](#_ENREF_45)] | NA | Solitaire FR, Trevo and ERIC | NA | anterior circulation | 90 day |
| Choi et al. 2018[[46](#_ENREF_46)] | tPA (NA) | NA | within 8 h | anterior circulation | 90 day |
| Gory B. et al. 2018[[47](#_ENREF_47)] | tPA (NA) | Solitaire, TrevoProvue, Revive, Capture and Eric | NA | posterior circulation | 90 day |
| Bourcier et al. 2018[[48](#_ENREF_48)] | NA | NA | within 4.5 h | anterior circulation | 90 day |
| Chu et al. 2018[[49](#_ENREF_49)] | rt-PA (24.2% receive 0.9 mg/kg) | NA | within 6 h for anterior circulation stroke and 24 h for posterior circulation stroke | anterior and posterior circulation | 90 day |
| Manceau et al. 2018[[50](#_ENREF_50)] | NA | Solitaire FR | within 6 h | anterior circulation | 90 day |
| Kaesmacher et al. 2018[[51](#_ENREF_51)] | rt-PA (NA) | NA | within 6 h for MT | anterior circulation | discharge |
| Rai et al. 2018[[52](#_ENREF_52)] | rt-PA | NA | within 4.5 h for IVT | anterior circulation | 90 day |
| Coutinho et al. 2017[[53](#_ENREF_53)] | tPA (0.62 mg/kg) | Solitaire FR | within 4.5 h for IVT and 8 h for MT | anterior and posterior circulation | 90 day |
| Bellwald et al. 2017[[54](#_ENREF_54)] | tPA (0.9 or 0.6 ml/kg) | NA | within 4.5 h | anterior circulation | 90 day |
| Kim et al. 2017[[55](#_ENREF_55)] | rt-PA (0.6 or 0.9 mg/kg) | Solitaire | within 4.5 h | anterior circulation | 24h |
| Uno et al. 2017[[56](#_ENREF_56)] | rt-PA (0.6 mg/kg with a loading dose of 10%) | Solitaire FR and TrevoProvue | within 8 h | posterior circulation | 90 day |
| Merlino et al. 2017[[57](#_ENREF_57)] | tPA (0.9 mg/kg over 1 h) | NA | within 4.5 h for IVT, 6 h for anterior circulation and 8 h for the posterior circulation | anterior and posterior circulation | 1 year |
| Wang et al. 2017[[58](#_ENREF_58)] | rt-PA (0.9 mg/kg) | Solitaire and Trevo | within 4.5 h | anterior circulation | 90 day |
| Park et al. 2017[[59](#_ENREF_59)] | NA (0.6 or 0.9 mg/kg) | Solitaire | within 8 h | anterior and posterior circulation | 90 day |
| Wee et al. 2017[[60](#_ENREF_60)] | Alteplase (0.9 mg/kg) | NA | within 4.5 h | anterior circulation | discharge |
| Abilleira et al. 2017[[61](#_ENREF_61)] | NA | NA | within 4.5 h | anterior circulation | 90 day |
| Maier et al. 2017[[62](#_ENREF_62)] | rt-PA(0.9 mg/kg over 1 h with 10% of initial bolus) | NA | within 6 h | anterior and posterior circulation | 90 day |
| Mundiyanapurath et al. 2017[[63](#_ENREF_63)] | NA | NA | NA | anterior circulation | 90 day |
| Leciñana et al. 2017[[64](#_ENREF_64)] | tPA (NA) | Solitaire and Trevo | within 4.5 h | anterior and posterior circulation | 90 day |
| Weber et al. 2017[[65](#_ENREF_65)] | NA | NA | within 4.5 h | anterior and posterior circulation | 90 day |
| Mistry et al.2017[[66](#_ENREF_66)] | tPA (NA) | NA | NA | anterior circulation | 48h |
| Nogueira et al. 2016[[67](#_ENREF_67)] | rt-PA (NA) | Solitaire FR | within 8 h for anterior circulation and 12 h for vertebrobasilar occlusion | anterior and posterior circulation | 90 day |
| Angermaier et al. 2016[[68](#_ENREF_68)] | rt-PA of 90 mg or 0.9 mg/kg | pREset | within 4.5 h for IVT and 6 h for EVT | anterior circulation | discharge |
| Rebello et al. 2016[[69](#_ENREF_69)] | tPA (NA) | NA | within 6 h | anterior and posterior circulation | 90 day |
| Broeg-Morvay et al. 2016[[70](#_ENREF_70)] | tPA (0.6 or 0.9 mL/kg) | NA | within 4.5 h | anterior circulation | 90 day |
| Behme et al. 2016[[71](#_ENREF_71)] | tPA (NA) | Penumbra Separator 3D and TrevoProvue | within 6 h | anterior circulation | discharge |
| Villwock.et al. 2016[[72](#_ENREF_72)] | rt-PA (NA) | NA | NA | anterior and posterior circulation | discharge |
| Minnerup et al. 2016[[73](#_ENREF_73)] | tPA (NA) | Solitaire, Preset, Penumbra System, Trevo, Revive, Merci and Other | within 4.5 h | anterior and posterior circulation | 90 day |
| Mulder et al.2016[[74](#_ENREF_74)] | Alteplase (NA) | NA | within 6 h | anterior circulation | 90 day |
| Wang et al. 2015[[75](#_ENREF_75)] | tPA (NA) | Merci, Penumbra and Solitaire | within 4.5 h for anterior circulation stroke and 24 h for posterior circulation strokes | anterior and posterior circulation | post thrombectomy |
| Bourcier et al. 2015[[76](#_ENREF_76)] | tPA (NA) | NA | NA | anterior circulation | 90 day |
| Saposnik et al. 2015[[77](#_ENREF_77)] | tPA or alteplase (NA) | Solitaire and Merci | within 8 h | anterior circulation | 90 day |
| Goyal M. et al. 2015[[78](#_ENREF_78)] | tPA (0.9 mg/kg) and alteplase (NA) | NA | within 12 h | anterior circulation | 90 day |
| Jeromel et al. 2015[[79](#_ENREF_79)] | NA | Catchdevice, Merci, PhenoxClot, Solitaire and Trevo | within 4.5 h for IVT and 5.5 h for EVT | anterior and posterior circulation | discharge |
| Guedin et al. 2015[[80](#_ENREF_80)] | rt-PA (0.9 mg/kg) | Solitaire FR | within 6 h | anterior circulation | 90 day |
| Fujimoto et al. 2015[[81](#_ENREF_81)] | tPA (0.9 mg/kg) | Merci, Penumbra System and Solitaire FR | NA | anterior circulation | discharge |
| Nogueira et al. 2015[[82](#_ENREF_82)] | tPA (NA) | Merci | within 8 h | anterior circulation | 90 day |
| Jankowitz et al. 2015[[83](#_ENREF_83)] | tPA (NA) | Merci, Solitaire and Trevo | NA | anterior and posterior circulation | 90 day |
| Jovin et al. 2015[[84](#_ENREF_84)] | Alteplase (NA) | NA | within 4.5 h for alteplase and 8 h for MT | anterior circulation | 90 day |
| Leker et al. 2015[[85](#_ENREF_85)] | tPA (NA) | Solitaire FR | within 8 h | anterior circulation | 90 day |
| Brinjikji et al. 2014[[86](#_ENREF_86)] | tPA (NA) | NA | NA | posterior circulation | discharge |
| Spiotta.et al.2014[[87](#_ENREF_87)] | tPA (NA) | Solitaire, Trevo Pro and Penumbra 3D separator | NA | anterior circulation | 90 day |
| Mourand et al. 2014[[88](#_ENREF_88)] | rt-PA (full dose 0.9 mg/kg with a loading dose of 10%) | Solitaire FR | within 24 h | posterior circulation | 180 day |
| Kass-Hout et al. 2014[[89](#_ENREF_89)] | tPA (0.9 mg/kg or 0.6 mg/kg) | Merci and Penumbra system | within 3 h | anterior and posterior circulation | 90 day |
| Jankowitz et al. 2012[[90](#_ENREF_90)] | tPA (NA) | Merci | NA | anterior circulation | 90 day |
| Da´valos et al. 2012[[91](#_ENREF_91)] | tPA (NA) | Solitaire FR | within 8 h and more than 8 h with large diffusion-perfusion mismatch in MRI | anterior and posterior circulation | 90 day |
| Pfefferkorn et al. 2012[[92](#_ENREF_92)] | NA | Penumbra System, AngioJet, Goose Neck Snare, MERCI and Phenox | within 8h | anterior and posterior circulation | 90 day |
| Bang et al. 2011[[93](#_ENREF_93)] | tPA (NA) | Merci and others | NA | anterior circulation | discharge |

Abbreviations: BT, bridging therapy; IVT, intravenous thrombolysis; IA, intra-arterial; IQR, interquartile range; NA, not available or not applicable; MT, mechanical thrombectomy; EVT, endovascular treatment; MRI, magnetic resonance imaging; tPA, tissue plasminogen activator; rt-PA, recombinant tissue plasminogen activator.

^#^ The 222 participants was admitted with an occlusion or high-grade stenosis (NASCET>70%) of the extracranial ICA with a concomitant ipsilateral occlusion of the intracranial ICA, the carotid-T or the MCA (M1 or M2 branch). The sample size 849 was admitted with acute intracranial vessel occlusion without concomitant ipsilateral ICA-occlusion or high-grade stenosis.

^‡^ The studies analyzed the occurrence of infarct in new territory (INT) with the patients of anterior and posterior circulation occlusions, patients of anterior circulation occlusion and patients of posterior circulation occlusion, respectively.

**Supplemental Table 4.** Time of procedure and presentation in enrolled studies

| **Study** | **Symptom onset to groin puncture time (Mean±SD/ Median (IQR))(min)** | **Groin puncture to reperfusion time (Mean±SD/ Median (IQR))(min)** | **Symptom onset to reperfusion time (Mean±SD/ Median (IQR))(min)** |
| --- | --- | --- | --- |
| Yang et al.2020[[1](#_ENREF_1)] | NA | NA | NA |
| Rajah et al.2020[[2](#_ENREF_2)] | NA | NA | NA |
| Mohammaden et al.2020[[3](#_ENREF_3)] | 272 (178-330) for DCM^¶^ 263 (190-342) for non-DCM^¶^ | 44 (34-97) for DCM^¶^ 30 (20-56) for non-DCM^¶^ | NA |
| Hassan et al.2020[[4](#_ENREF_4)] | 172 for EVT 181 for BT (median) | NA | 206.5 for EVT 224 for BT (median) |
| Uchikawa et al.2019[[5](#_ENREF_5)] | NA | NA | NA |
| Wareham et al.2019[[6](#_ENREF_6)] | NA | NA | NA |
| Pikija et al. 2019 [[7](#_ENREF_7)] | 181 (160-227) mean (IQR) | NA | 276 (207-323) mean (IQR) |
| Tajima et al. 2019[[8](#_ENREF_8)] | NA | 63 ± 45 for BT 58 ± 45 for MT | 221 ± 82 for BT 233 ± 74 for MT |
| Gong et al. 2019[[9](#_ENREF_9)] | 95.9 (35.2) for BT  96.64 (34.8) for MT median(SD) | NA | 295.1 (87.5) for BT 306.8 (123.75) for MT |
| Lee et al.2019[[10](#_ENREF_10)] | 251.4 ± 117.6 | NA | NA |
| Kaesmacher et al. 2019[[11](#_ENREF_11)] | 257 (196-393)^*^ | NA | NA |
|  | NA^*^ |  |  |
| Balodis et al. 2019[[12](#_ENREF_12)] | NA | 37 (27-50) for BT 31 (22-59) for MT | 260 (240-320) for BT 280 (240-347) for MT |
| Meyer et al. 2019[[13](#_ENREF_13)] | NA | 39 (25-57) | NA |
| Hassan et al. 2019[[14](#_ENREF_14)] | NA | NA | NA |
| Goyal N.et al. 2019[[15](#_ENREF_15)] | 223 (172-291) for BT 360 (243-562) for MT | 48 (33-70)for BT 70 (44-98)for MT | 285 (226-353) for BT 415 (308-601) for MT |
| Casetta et al. 2019 [[16](#_ENREF_16)] | 230 (185-275) for BT 210 (170-270) for MT | 55 (27.5-90) for BT 60 (34.5–90) for MT | NA |
| Chalos et al. 2019[[17](#_ENREF_17)] | 206 (160-260) for BT 215 (158-294) for EVT | NA | NA |
| Gamba et al. 2019[[18](#_ENREF_18)] | 194.1 ± 59.9 for BT 204.8 ± 60.4 for MT | NA | 245.9 ± 75.8 for BT 245.1 ± 58.6 for MT |
| Aoki et al. 2019[[19](#_ENREF_19)] | 206 (142-316) for Successful recanalization 198 (127-333) for not successful recanalization | 39 (23-64) | 277 (194-431) |
| Goyal N.et al. 2019[[20](#_ENREF_20)] | 246 (180-324) for no INT^‡^ 288 (200-440) for INT^‡^ | 52 (35-79) for no INT^‡^ 61 (40-88) for INT^‡^ | NA |
| Raychev et al. 2019[[21](#_ENREF_21)] | NA | NA | NA |
| Anadani et al. 2019[[22](#_ENREF_22)] | 234 (100) for BT 256 (234) for MT | 75 (48) for BT 85 (44) for MT | NA |
| Qureshi et al. 2019[[23](#_ENREF_23)] | 318 ± 90 for BT 678 ± 990 for MT | NA | NA |
| Wollenweber et al. 2019[[24](#_ENREF_24)] | NA | 70 (46-100) | 249 (191-325) |
| Panni et al. 2019[[25](#_ENREF_25)] | 247.1 ± 93.3 | 60 (36-91) | 320.9 ± 109.7 |
| Tran et al.2019[[26](#_ENREF_26)] | NA | NA | NA |
| Candel et al. 2019[[27](#_ENREF_27)] | NA | NA | NA |
| Bücke et al. 2018[[28](#_ENREF_28)] | 216 ± 66^#^ | NA | 360 ± 108^#^ |
|  | 222 ± 114^#^ | NA | 306 ± 90^#^ |
| Ren et al. 2018[[29](#_ENREF_29)] | 240 (186-294) for BT 252 (186-330) for MT | NA | NA |
| Anadani et al. 2018[[30](#_ENREF_30)] | within 360 min | NA | NA |
| Rocha et al. 2018[[31](#_ENREF_31)] | 245 (140) for BT 267.5 (203) for MT | NA | 308 (146) for BT 336.5 (217) for MT |
| Leker et al. 2018[[32](#_ENREF_32)] | 258 ± 90 for BT 276 ± 126 for MT | 48.5 ± 40 for BT 42.6 ± 39 for MT | NA |
| Gariel et al. 2018[[33](#_ENREF_33)] | 225 (183-280) for BT 230 (170-282) for MT | NA | NA |
| Sallustio et al. 2018[[34](#_ENREF_34)] | 225 (77-380) for BT 222.5 (70-809) for MT median (range) | 58 (5-180) for BT 60 (7-356) for MT median (range) | 288 (141-435) for BT 300 (90-845) for MT median (range) |
| DiMaria et al. 2018[[35](#_ENREF_35)] | 235 (190-285) for BT 244 (185-308) for MT | NA | NA |
| Díaz-Pérez et al. 2018[[36](#_ENREF_36)] | 257 (74) for BT 232.5 (111) for MT | 63 (62) for BT 75.5 (69) for MT | 327.5 (154) for BT 314.5 (111) for MT |
| Maingard et al. 2018[[37](#_ENREF_37)] | 257 ± 104 for BT 342 ± 223 for MT | 32 ± 30 for BT 33 ± 38 for MT | 297 ± 110 for BT 385 ± 225 for MT |
| Al-Khaled et al. 2018[[38](#_ENREF_38)] | NA | 50 (29-93) for BT 52 (21-129) for MT | NA |
| Ferrigno et al. 2018[[39](#_ENREF_39)] | 259 (198-337) for BT 251 (178-351) for MT | NA | NA |
| Imbarrato et al. 2018[[40](#_ENREF_40)] | NA | NA | NA |
| Li et al. 2018[[41](#_ENREF_41)] | 395 (312-517) | 90 (65-118) | NA |
| Wang et al. 2018[[42](#_ENREF_42)] | 235 (180-300) | 95 (64-140) | NA |
| Gory et al. 2018[[43](#_ENREF_43)] | NA | NA | 303 (245-387) |
| Goyal N. et al. 2018[[44](#_ENREF_44)] | 223 (158-295) for BT 303 (195-455) for MT | 45 (26-66) for BT 37 (24-63) for MT | NA |
| Barral et al. 2018[[45](#_ENREF_45)] | 260 (220-327) | NA | 304 (239.75-380.25) |
| Choi et al. 2018[[46](#_ENREF_46)] | 204.7 ± 63.7 for BT 221.6 ± 110.5 for MT | NA | NA |
| Gory B. et al. 2018[[47](#_ENREF_47)] | 311 (228-453) for alive 315 (247-387) for dead | NA | NA |
| Bourcier et al. 2018[[48](#_ENREF_48)] | 240 (200-300) for BT 200 (140-280) for MT | NA | 294 (240-360) for BT 270 (203-340) for MT |
| Chu et al. 2018[[49](#_ENREF_49)] | 200 (148.5-273) | 29.5 (18-50) | 231.5 (180-297) |
| Manceau et al. 2018[[50](#_ENREF_50)] | NA | NA | NA |
| Kaesmacher et al. 2018[[51](#_ENREF_51)] | 195 (157-247) for BT 195 (135-256) for MT | NA | 261 (199-305) for BT 273 (198.75-304.75) for MT |
| Rai et al. 2018[[52](#_ENREF_52)] | 319 ± 270 for EVT 106 ± 52 for BT | NA | NA |
| Coutinho et al.2017[[53](#_ENREF_53)] | 254 (195-305) for BT 262 (201-375) for MT | NA | 308 (253-361) for BT 315 (242-424) for MT |
| Bellwald et al. 2017[[54](#_ENREF_54)] | 255.6 (121.2) for BT 193.80 (82.2) for MT | NA | NA |
| Kim et al. 2017[[55](#_ENREF_55)] | NA | NA | 320(270–415) |
| Uno et al. 2017[[56](#_ENREF_56)] | NA | NA | 197 (160–256) |
| Merlino et al. 2017[[57](#_ENREF_57)] | 218.0 ± 66.8 for BT 217.8 ± 76.7 for MT | 90.4 ± 39.1 for BT 88.1 ± 42.3 for MT | NA |
| Wang et al. 2017[[58](#_ENREF_58)] | NA | 106 (78-156) for BT  95 (69-126) for MT | NA |
| Park et al. 2017[[59](#_ENREF_59)] | 215 ± 95 for BT 296 ± 101 for EVT | 89 ± 57 for BT 90 ± 54 for EVT | NA |
| Wee et al. 2017[[60](#_ENREF_60)] | 165 ± 60 for BT 220 ± 187 for MT | NA | 227 ± 72 for BT 273 ± 188 for MT |
| Abilleira et al. 2017[[61](#_ENREF_61)] | 246 (186-318) for BT 300 (174-564) for MT | NA | NA |
| Maier et al. 2017[[62](#_ENREF_62)] | 153 (120-212.5) for BT 173.5 (119.8-252.5) for MT | 54 (36.5-76.5) for BT 50 (39-81) for MT | NA |
| Mundiyanapurath et al. 2017[[63](#_ENREF_63)] | NA | NA | NA |
| Leciñana et al. 2017[[64](#_ENREF_64)] | 275 (225-345) for BT 210 (168-257) for MT | 60 (40-84) for BT 69 (47-117) for MT | 325 (280-390) for BT 259 (215-328) for MT |
| Weber et al. 2017[[65](#_ENREF_65)] | 233 (198-295) for BT 210 (152-330) for MT | NA | NA |
| Mistry et al.2017[[66](#_ENREF_66)] | 260 (180-375.8) | NA | NA |
| Nogueira et al. 2016[[67](#_ENREF_67)] | 274 ± 112 for BT 436.7 ± 289 for MT | NA | NA |
| Angermaier et al. 2016[[68](#_ENREF_68)] | NA | NA | NA |
| Rebello et al. 2016[[69](#_ENREF_69)] | 235 (181-289) for BT 250 (181-304) for MT | 66 (48-98) for BT 70 (48-111) for MT | NA |
| Broeg-Morvay et al. 2016[[70](#_ENREF_70)] | 262 ± 85 for BT 229 ± 79 for MT | NA | NA |
| Behme et al. 2016[[71](#_ENREF_71)] | 194 (83-396) for BT 192 (72-329) for MT median (range) | NA | NA |
| Villwock.et al. 2016[[72](#_ENREF_72)] | NA | NA | NA |
| Minnerup et al. 2016[[73](#_ENREF_73)] | 233 ± 179 for BT 294 ± 227 for MT | NA | 144 ± 137 for BT unknown for MT |
| Mulder et al.2016[[74](#_ENREF_74)] | 265 (214-315) for IVT 242 (200-300) for no IVT | 72 (52-97) for IVT 67 (43-88) for no IVT | 343 (283-394) for IVT 310 (242-404) for no IVT |
| Wang et al. 2015[[75](#_ENREF_75)] | 347.9 ± 235.6 | NA | NA |
| Bourcier et al. 2015[[76](#_ENREF_76)] | 18 cases for ≤ 270 min 28 cases for 270 min to 360 min 27 cases for >360 min | NA | NA |
| Saposnik et al. 2015[[77](#_ENREF_77)] | NA | NA | NA |
| Goyal M.et al. 2015[[78](#_ENREF_78)] | NA | 30 (18-46) | 241(176-359) |
| Jeromel et al. 2015[[79](#_ENREF_79)] | 215 (162-289) | NA | NA |
| Guedin et al. 2015[[80](#_ENREF_80)] | 240 (187-275) for BT 204 (175-290) for MT | 35 (21-60) for BT 60 (25-91) for MT | NA |
| Fujimoto et al. 2015[[81](#_ENREF_81)] | 352 (230-480) | 352 (230-480) | NA |
| Nogueira et al. 2015[[82](#_ENREF_82)] | NA | NA | NA |
| Jankowitz et al. 2015[[83](#_ENREF_83)] | 267 (69.6-2058) | 70 (15-289) | NA |
| Jovin et al. 2015[[84](#_ENREF_84)] | 269 (201-340) | NA | 355 (269-430) |
| Leker et al. 2015[[85](#_ENREF_85)] | NA | NA | NA |
| Brinjikji et al. 2014[[86](#_ENREF_86)] | NA | NA | NA |
| Spiotta.et al.2014[[87](#_ENREF_87)] | NA | NA | NA |
| Mourand et al. 2014[[88](#_ENREF_88)] | NA | NA | NA |
| Kass-Hout et al. 2014[[89](#_ENREF_89)] | NA | 65.67 ± 31.20 for BT 74.56 ± 43.12 for EVT | NA |
| Jankowitz et al.2012[[90](#_ENREF_90)] | NA | NA | NA |
| Da´valos et al. 2012[[91](#_ENREF_91)] | 290 (225-360) for BT 330 (195-605) for MT median (range) | 44 (30-54) for BT 37 (28-54) for MT median (range) | NA |
| Pfefferkorn et al. 2012[[92](#_ENREF_92)] | NA | NA | 360 ± 84 for no IVT 360 ± 90 for IVT |
| Bang et al. 2011[[93](#_ENREF_93)] | 318 ± 132 | NA | NA |

Abbreviations: BT, bridging therapy; SD, standard deviation; IQR, interquartile range; NA, not available or not applicable; MT, mechanical thrombectomy; EVT, endovascular treatment; IVT, intravenous thrombolysis

^*^The symptom onset to groin puncture time 257 (196–393) (Median (IQR) was calculated with the sample size 193 which was enrolled to evaluate the outcome of reperfusion success (TICI2b/3), and the symptom onset to groin puncture time was not available for the sample size 152 which was enrolled for the outcome of non-hemorrhagic neurological worsening (drop in the NIHSS ≥ 4 between admission NIHSS and 24 h NIHSS without the occurrence of sICH).

^#^The symptom onset to groin puncture time 216 ± 66 (Mean±SD) and the symptom onset to reperfusion time 360 ± 108 (Mean±SD) were calculated with the 222 participants which were admitted with an occlusion or high-grade stenosis (NASCET>70%) of the extracranial ICA with a concomitant ipsilateral occlusion of the intracranial ICA, the carotid-T or the MCA (M1 or M2 branch). The symptom onset to groin puncture time 222 ± 114 (Mean±SD) and the symptom onset to reperfusion time 306 ± 90 (Mean±SD) were calculated with the sample size 849 was admitted with acute intracranial vessel occlusion without concomitant ipsilateral ICA-occlusion or high-grade stenosis.

^‡^INT: infarct in new territory (All infarcts located outside the immediate territory of the vessel implicated in the presenting stroke).

^¶^DCM: distal clot migration.

**Supplemental Table 5.** The confounders were used for adjustment in enrolled studies

| **Study** | **Confounders adjustment** |
| --- | --- |
| Yang et al.2020[[1](#_ENREF_1)] | Adjusted for age, the NIHSS score at baseline, the time from stroke onset to randomization, the modified Rankin Scale score before stroke onset, and cerebral collateral blood-flow status |
| Rajah et al.2020[[2](#_ENREF_2)] | Adjusted for tPA, NIHSS, CT to Angio time, age, atrial Fibrillation, hypertension, and diabetes |
| Mohammaden et al.2020[[3](#_ENREF_3)] | Adjusted for groin puncture to recanalization, prior IV-tPA administration, and technique used (stentrievers) |
| Hassan et al.2020[[4](#_ENREF_4)] | Adjusted for diabetes mellitus, hypertension, age, and atrial fibrillation |
| Uchikawa et al. 2019[[5](#_ENREF_5)] | NA |
| Wareham et al. 2019[[6](#_ENREF_6)] | NA |
| Pikija et al. 2019[[7](#_ENREF_7)] | Adjusted for age and the NIHSS score at admission were entered as continuous variables, whereas the binary co-variables included final infarct volume, success of recanalization, and use of intravenous tPA. |
| Tajima et al. 2019[[8](#_ENREF_8)] | NA |
| Gong et al. 2019[[9](#_ENREF_9)] | Adjusted for propensity score-matching |
| Lee et al.2019[[10](#_ENREF_10)] | Adjusted for age, male, NIHSS, hypertension, diabetes mellitus, pre-stroke antithrombotic, creatinine, initial random glucose, onset to EVT start time, and preceding IVT |
| Kaesmacher et al. 2019[[11](#_ENREF_11)] | NA |
| Balodis et al. 2019[[12](#_ENREF_12)] | NA |
| Meyer et al. 2019[[13](#_ENREF_13)] | Adjust for age, sex, NIHSS, ASPECTS, number of passes, intravenous thrombolysis, TICI >0, and groin to recanalization time |
| Hassan et al. 2019[[14](#_ENREF_14)] | Adjust for NIHSS at admission and IVT |
| Goyal N.et al. 2019[[15](#_ENREF_15)] | Adjust for demographics, vascular risk factors, baseline ASPECTS, blood pressure and serum glucose levels, onset-to-groin-puncture time, location of occlusion, pretreatment with antiplatelets, anticoagulants, and statins |
| Casetta et al. 2019[[16](#_ENREF_16)] | Adjust for age, sex, history of diabetes, atrial fibrillation, hypertension, previous stroke or transient ischemic attack in the previous three months, the presence of carotid stenosis>70%, baseline NIHSS score, baseline ASPECTS score, onset to endovascular-capable center arrival time, onset to groin puncture time, and site of occlusion |
| Chalos et al. 2019[[17](#_ENREF_17)] | Adjust for age, baseline NIHSS, history of atrial fibrillation, diabetes mellitus, hypertension, ischemic stroke, myocardial infarction, pre-stroke mRS, prior use of anticoagulant medication, and onset-to-first noncontrast CT time |
| Gamba et al. 2019[[18](#_ENREF_18)] | Adjust for baseline NIHSS, ASPECTS score, TICI 2b or 3 reperfusion, IVT+MT, site of occlusion, time from symptoms onset to recanalization, and first-pass success |
| Aoki et al. 2019[[19](#_ENREF_19)] | Adjust for NIHSS score, age, gender, atrial fibrillation, intravenous thrombolysis, DWI-ASPECTS, internal carotid artery occlusion, number of passes, puncture to first-pass, and Merci retriever |
| Goyal N.et al. 2019[[20](#_ENREF_20)] | Adjust for hypertension, IV tPA, good collaterals, pretreatment with antiplatelets, onset-to-groin puncture time, and occlusion site in posterior circulation |
| Raychev et al. 2019[[21](#_ENREF_21)] | Adjust for IV rt-PA and nset to groin puncture |
| Anadani et al. 2019[[22](#_ENREF_22)] | Adjust for age, current smoking, NIHSS, heparin use, ASPECTS > 7, intravenous thrombolysis, symptoms onset to groin puncture time, groin puncture to mTICI 2b-3 time, number of antiplatelet used, and intracranial occlusion location |
| Qureshi et al. 2019[[23](#_ENREF_23)] | NA |
| Wollenweber et al. 2019[[24](#_ENREF_24)] | Adjust for age, sex, NIHSS, intravenous alteplase use, ASPECTS, and time from symptom onset to groin puncture |
| Panni et al. 2019[[25](#_ENREF_25)] | NA |
| Tran et al.2019[[26](#_ENREF_26)] | NA |
| Candel et al. 2019[[27](#_ENREF_27)] | NA |
| Bücke et al. 2018[[28](#_ENREF_28)] | Considering possible confounders (based on literature research and *P* < 0.05 in baseline characteristics) |
| Ren et al. 2018[[29](#_ENREF_29)] | NA |
| Anadani et al. 2018[[30](#_ENREF_30)] | Adjust for ASPECT score, onset to groin time 6 hours, IV-tPA use, location of occlusion (classified as Internal carotid artery, middle cerebral artery, anterior cerebral artery, or posterior circulation), and final mTICI score. |
| Rocha et al. 2018[[31](#_ENREF_31)] | Adjust for age, NIHSS, ASPECTS, intracranial ICA occlusion, cardioembolic stroke, and time from symptom onset to recanalization |
| Leker et al. 2018[[32](#_ENREF_32)] | Adjust for admission NIHSS (per point), age (per year), gender, time to endovascular treatment, and stroke subtype |
| Gariel et al. 2018[[33](#_ENREF_33)] | Adjust for center, first-line strategy, and prespecified confounding factors (age, hypertension, diabetes mellitus, admission NIHSS, and ASPECTS scores, site of occlusion, onset to puncture time)^†^ |
|  | Adjust for center and first-line strategy^‡^ |
| Sallustio et al. 2018[[34](#_ENREF_34)] | Adjusted for age and NIHSS |
| DiMaria et al. 2018[[35](#_ENREF_35)] | Adjusted for age, sex, direct admission, hypertension, diabetes, hypercholesterolemia, current smoking, antiplatelet use, anticoagulation, NIHSS score, ASPECTS score, pre-stroke mRS≥1, site of occlusion, favorable collaterals, stroke etiology, onset-to-groin puncture, general anesthesia, and first-line strategy (ADAPT, stent retriever) |
|  |  |
| Díaz-Pérez et al. 2018[[36](#_ENREF_36)] | NA |
| Maingard et al. 2018[[37](#_ENREF_37)] | Adjusted for age, sex, hypertension, hyperlipidaemia, ischaemic heart disease, diabetes, atrial fibrillation, previous stroke, NIHSS, ASPECTS, collateral grade, general anaesthesia during EVT, number of passes, successful reperfusion mTICI ≥2b, sICH, interval from symptom onset to groin puncture, and time to reperfusion |
|  |  |
| Al-Khaled et al. 2018[[38](#_ENREF_38)] | Adjusted for age, sex, NIHSS at admission, and arterial hypertension, diabetes mellitus, hypercholesterolemia, previous stroke, and atrial fibrillation |
| Ferrigno et al. 2018[[39](#_ENREF_39)] | Adjusted for age, hypertension, diabetes mellitus, hypercholesterolemia, smoking, previous or current atrial fibrillation, previous transient ischemic attack or stroke, previous antithrombotic medication, unknown onset stroke, admission NIHSS and ASPECTS score, onset to puncture time, and site of occlusion |
| Imbarrato et al. 2018[[40](#_ENREF_40)] | NA |
| Li et al. 2018[[41](#_ENREF_41)] | NA |
| Wang et al. 2018[[42](#_ENREF_42)] | Adjusted for atrial fibrillation, NLR, INR, glucose, baseline NIHSS score, baseline ASPECTS, collateral status, prior IVT, from puncture to recanalization, passes of stent retriever, and sICH |
| Gory et al. 2018[[43](#_ENREF_43)] | Adjusted for admission NIHSS score, ASPECTS≥7, prior use of IV t-PA, extracranial ICA stenting, and antiplatelets |
| Goyal N. et al. 2018[[44](#_ENREF_44)] | Adjusted for age, sex, admission NIHSS, occlusion in posterior circulation, onset-to-groin puncture, and groin puncture-to-recanalization |
| Barral et al. 2018[[45](#_ENREF_45)] | Adjusted for Sex, diabetes, thrombolysis, general anesthesia, reperfusion, age, NIHSS, and DWI-ASPECTS |
| Choi et al. 2018[[46](#_ENREF_46)] | Adjusted for sex, baseline NIHSS score, hypertension, oral anticoagulants, tPA injection before MT, onset to puncture time, and TICI 2b or 3 reperfusions |
| Gory B. et al. 2018[[47](#_ENREF_47)] | Adjusted for age, prior use of antithrombotic medications, admission NIHSS, pc-ASPECTS, intravenous thrombolysis, general anesthesia, dilatation procedure, successful recanalization, and rescue thrombectomy |
| Bourcier et al. 2018[[48](#_ENREF_48)] | Adjusted for age, gender, wake-up stroke, hypertension, dyslipidemia, smoking, diabetes, obesity, imaging at the neurointerventional center, baseline NIHSS score, time from onset to imaging, baseline ASPECT score, cervical lesion, and anterior collaterality |
| Chu et al. 2018[[49](#_ENREF_49)] | Adjust for factors of age, gender, initial NIHSS scores, IV rt-PA, time from onset to puncture, and successful recanalization |
| Manceau et al. 2018[[50](#_ENREF_50)] | Adjust for age, smoking, glycaemia, intravenous thrombolysis, DWI-ASPECTS, symptoms onset to angiography, and recanalization |
| Kaesmacher et al. 2018[[51](#_ENREF_51)] | NA |
| Rai et al. 2018[[52](#_ENREF_52)] | NA |
| Coutinho et al. 2017[[53](#_ENREF_53)] | Adjusted for age, sex, NIHSS, ASPECTS, location of occlusion, international normalized ratio, antiplatelet use, atrial fibrillation, diabetes, site of recruitment, and systolic blood pressure^*^ |
|  |  |
|  | Adjusted for age, sex, NIHSS, ASPECTS, location of occlusion, atrial fibrillation, diabetes, site of recruitment, and interval from symptom onset to hospital arrival^#^ |
|  |  |
| Bellwald et al. 2017[[54](#_ENREF_54)] | Adjusted for age, baseline NIHSS, history of coronary heart disease, time to diagnosis, and presence of ICA lesions |
| Kim et al. 2017[[55](#_ENREF_55)] | Adjust for initial glucose, initial systolic blood pressure, baseline NIHSS score, intravenous tPA before EVT, onset-to-recanalization time, and symptomatic intracranial hemorrhage |
| Uno et al. 2017[[56](#_ENREF_56)] | Adjust for NIHSS score, pc-ASPECT score, time from onset to recanalization, and IV rt-PA use |
| Merlino et al. 2017[[57](#_ENREF_57)] | Adjust for age, NIHSS at admission, pre-stroke mRS, use of anticoagulants at admission, and time from symptoms onset to EVT |
| Wang et al. 2017[[58](#_ENREF_58)] | Adjust for age, sex, previous stroke, pre-morbid mRS, time from onset to door, stroke etiology, occlusion site, baseline ASPECTS, baseline NIHSS score, and collateral status |
| Park et al. 2017[[59](#_ENREF_59)] | Adjust for history of stroke or TIA, prestroke anticoagulants, interval from onset to hospital arrival, interval from onset to EVT start, interval from hospital arrival to EVT start, age, initial stroke severity, location of occluded artery, and EVT methods (In the analysis of sHT, initialrandom glucose was added) |
| Wee et al. 2017[[60](#_ENREF_60)] | NA |
| Abilleira et al. 2017[[61](#_ENREF_61)] | Adjust for age, sex, baseline NIHSS, hypertension, hypercholesterolemia, diabetes mellitus, coronary heart disease, heart failure, previous stroke/transient ischemic attack, atrial fibrillation, oral anticoagulation, antiplatelet medication, pathogenesis (atherotrombotic, cardioembolism, other), clot location (internal carotid artery/tandem or middle cerebral artery), and time from onset to puncture |
|  |  |
|  |  |
| Maier et al. 2017[[62](#_ENREF_62)] | NA |
| Mundiyanapurath et al. 2017[[63](#_ENREF_63)] | Adjust for gender, hypertension, previous stroke, and Tirofiban |
| Leciñana et al. 2017[[64](#_ENREF_64)] | Adjust for age, time to reperfusion, treatment with anticoagulants, NIHSS on admission, and type of treatment |
| Weber et al. 2017[[65](#_ENREF_65)] | NA |
| Mistry et al.2017[[66](#_ENREF_66)] | NA |
| Nogueira et al. 2016[[67](#_ENREF_67)] | Adjust for age, baseline NIHSS, occlusion site, initial systolic blood pressure, IV rt-PA, time to revascularization or end of procedure, general anesthesia, use of balloon guide catheter, mean number of passes, use of rescue therapy, mTICI ≥2a, mTICI ≥2b, and sICH |
| Angermaier et al. 2016[[68](#_ENREF_68)] | Adjusted for occlusion localization, IVT, endovascular revascularization treatment, MT, and passes of thrombectomy |
| Rebello et al. 2016[[69](#_ENREF_69)] | NA |
| Broeg-Morvay et al. 2016[[70](#_ENREF_70)] | Adjusted for age, gender, baseline NIHSS, current diabetes mellitus, current hypertension (treated or untreated), current coronary heart disease, current hypercholesterolemia (treated or untreated), lesion location (MCA, ICA), TOAST classification (cardioembolic, macroangiopathy, unclear diagnosis with complete or with incomplete information, multiple causes), active smoking, and time from symptom onset to diagnosis |
| Behme et al. 2016[[71](#_ENREF_71)] | Adjusted for age, thrombus length, thrombus length (≤15 mm), no of stent retriever passes ≤2, concomitant IVT, time from groin puncture to final recanalization, and time from groin puncture to final recanalization (≤1 h) |
| Villwock.et al. 2016[[72](#_ENREF_72)] | Adjust for age, gender, rt-PA administration, number of comorbidities, APR-DRG risk of mortality, and year of treatment |
| Minnerup et al. 2016[[73](#_ENREF_73)] | NA |
| Mulder et al.2016[[74](#_ENREF_74)] | NA |
| Wang et al. 2015[[75](#_ENREF_75)] | Adjust for age, gender, type 2 diabetes mellitus, hypertension, hypercholesterolemia, atrial fibrillation, ischaemic heart disease, previous stroke or TIA, baseline NIHSS, IV tPA, IA urokinase, IA tPA, anterior circulation occlusion, and the time to DSA |
|  |  |
| Bourcier et al. 2015[[76](#_ENREF_76)] | Adjust for age, ICA occlusion, susceptibility vessel sign on MRI, lack of spontaneous hyperattenuation on CT, day 1 NIHSS, and TICI |
| Saposnik et al. 2015[[77](#_ENREF_77)] | NA |
| Goyal M.et al. 2015[[78](#_ENREF_78)] | NA |
| Jeromel et al. 2015[[79](#_ENREF_79)] | Adjust for the interval period, age, use of IVT, and duration of the procedure on the clinical outcome |
| Guedin et al. 2015[[80](#_ENREF_80)] | NA |
| Fujimoto et al. 2015[[81](#_ENREF_81)] | Adjust for FLAIR clot intensity, intravenous t-PA, and Solitaire FR revascularization device |
| Nogueira et al. 2015[[82](#_ENREF_82)] | Adjust for NIHSS, clot location, diabetes mellitus, time from symptoms onset to puncture, Merci device, IV tPA prior to intervention, and multimodal therapy |
| Jankowitz et al. 2015[[83](#_ENREF_83)] | NA |
| Jovin et al. 2015[[84](#_ENREF_84)] | NA |
| Leker et al. 2015[[85](#_ENREF_85)] | Adjust for age, prior use of tPA, collateral state, the need for more than 1 pass of the device, and admission NIHSS score |
| Brinjikji et al. 2014[[86](#_ENREF_86)] | Adjust for age, the charlson comorbidity index, gender, nonwhite race, and IV-tPA |
| Spiotta.et al.2014[[87](#_ENREF_87)] | Adjust for age, time from symptom onset to CT, time from CT to groin puncture, IV tPA, IA tPA, recanalization time, recanalization time (10 min unit), atrial fibrillation, diabetes, hypertension, sedation: general anesthetic, baseline NIHSS, post TICI Flow: ‘good’ (2b or 3), hemorrhage, and procedural complications |
| Mourand et al. 2014[[88](#_ENREF_88)] | NA |
| Kass-Hout et al. 2014[[89](#_ENREF_89)] | NA |
| Jankowitz et al. 2012[[90](#_ENREF_90)] | NA |
| Da´valos et al. 2012[[91](#_ENREF_91)] | NA |
| Pfefferkorn et al. 2012[[92](#_ENREF_92)] | NA |
| Bang et al. 2011[[93](#_ENREF_93)] | Adjust for atrial fibrillation, a-glucose on admission, NIHSS score on admission, mode of endovascular treatment, IV tPA before endovascular therapy, collateral grading, and TIMI status |

Abbreviations: NIHSS, National Institutes of Health Stroke Scale; ASPECTS, Alberta Stroke Program Early Computed Tomography Score; NA, not available or not applicable; tPA, tissue plasminogen activator; EVT, endovascular treatment; IVT, intravenous thrombolysis; IV, intravenous; ADAPT, a direct aspiration first pass technique; mTICI, modified thrombolysis in Cerebral Infarction; ICA, internal carotid artery; sICH, symptomatic intracranial hemorrhage; NLR: Neutrophil-to-lymphocyte ratio; INR, international normalized ratio; DWI, Diffusion Weighted Imaging; MT, mechanical thrombectomy; TICI, thrombolysis in Cerebral Infarction; pc-ASPECTS, posterior circulation-Alberta Stroke Program Early computed tomography Score; mRS, modified Rankin Scale; TIA, transient ischemic attacks; sHT, symptomatic hemorrhagic transformation; TOAST, Trial of ORG 10172 in Acute Stroke Treatment; MCA, middle cerebral artery; IA, intra-arterial; DSA, digital substraction angiography; MRI, magnetic resonance imaging; APR-DRG, all patient refined-diagnosis related group; FLAIR, fluid attenuated inversion recovery; CT, computed tomography; TIMI, thrombolysis in myocardial infarction; rt-PA, recombinant tissue plasminogen activator.

^*^The confounders adjustment was to evaluate the outcome of successful reperfusion, mTICI 3, >3 passes with stent retriever, sICH, emboli to uninvolved territory and vasospasm.

^#^The confounders adjustment was to evaluate the outcome of good outcomes and mortality.

^†^The confounders adjustment was to evaluate the outcome of the use of rescue therapy, reperfusion at end of procedure, no. of passes >2, procedural complications, emboli, favorable outcome, excellent outcome, 90-d mortality, any ICH and PH.

^‡^The confounders adjustment was to evaluate the outcome of the sICH.

**Supplemental Table 6.** Overview of the pooled results with unadjusted analyses

| **Outcome** | **Subgroup** | **Unadjusted Analyses** | | | | | | |
| --- | --- | --- | --- | --- | --- | --- | --- | --- |
|  |  | **No. of studies (subgroups/ subtypes)^*^** | **cOR/ cRR (95%CI)** | **Heterogeneity** | | | **Overall effect** | **Egger's test** |
|  |  |  |  | **Chi^2^, d.f.** | **I^2^, *P* for Cochran Q** | **Tau^2^** | **Z value, *P* value** | ***P* value** |
| **Excellent outcome at 90 days** | **All** | **16** | **1.354(1.170-1.566)** | **27.07,d.f.=15** | **44.6%, *P*=0.028** | **0.0348** | **4.08, *P*=0.000** | **0.308** |
|  | Study type |  |  |  |  |  |  |  |
|  | Prospective | 9 | 1.383(1.144-1.673) | 14.94,d.f.=8 | 46.4%, *P*=0.060 | 0.0349 | 3.35, *P*=0.001 |  |
|  | Retrospective | 7 | 1.316(1.038-1.668) | 10.74,d.f.=6 | 44.1%, *P*=0.097 | 0.0424 | 2.27, *P*=0.023 |  |
|  | Location of occluded artery |  |  |  |  |  |  |  |
|  | Anterior circulation | 14 | 1.340(1.127-1.593) | 26.54,d.f.=13 | 51.0%, *P*=0.014 | 0.0485 | 3.31, *P*=0.001 |  |
|  | Anterior and posterior circulation | 2 | 1.413(1.113-1.794) | 0.53,d.f.=1 | 0.0%, *P*=0.468 | 0.0000 | 2.84, *P*=0.005 |  |
|  | Ethnicity |  |  |  |  |  |  |  |
|  | Caucasian | 12 | 1.486(1.299-1.700) | 15.08,d.f.=11 | 27.1%, *P*=0.179 | 0.0142 | 5.77, *P*=0.000 |  |
|  | Caucasian and Asian | 2 | 0.837(0.473-1.482) | 0.20,d.f.=1 | 0.0%, *P*=0.651 | 0.0000 | 0.61, *P*=0.542 |  |
|  | Asian | 2 | 0.954(0.714-1.276) | 0.33,d.f.=1 | 0.0%, *P*=0.566 | 0.0000 | 0.31, *P*=0.753 |  |
| **Excellent outcome in-hospital/ at discharge** | **All** | **2** | **1.006(0.593-1.708)** | **2.61,d.f.=1** | **61.7%, *P*=0.106** | **0.0962** | **0.02, *P*=0.981** | **NA** |
| **Good outcome at 90 days** | **All** | **58(59)** | **1.361(1.234-1.502)** | **111.42,**  **d.f.=58** | **47.9%, *P*=0.000** | **0.0571** | **6.16, *P*=0.000** | **0.270** |
|  | Study type |  |  |  |  |  |  |  |
|  | Prospective | 21(22) | 1.352(1.165-1.568) | 45.78,d.f.=21 | 54.1%, *P*=0.001 | 0.0540 | 3.98, *P*=0.000 |  |
|  | Retrospective | 37 | 1.371(1.199-1.568) | 65.65,d.f.=36 | 45.2%, *P*=0.002 | 0.0670 | 4.62, *P*=0.000 |  |
|  | Location of occluded artery |  |  |  |  |  |  |  |
|  | Anterior and posterior circulation | 18 | 1.456(1.230-1.724) | 29.31,d.f.=17 | 42.0%, *P*=0.032 | 0.0487 | 4.37, *P*=0.000 |  |
|  | Anterior circulation | 38(39) | 1.301(1.155-1.465) | 74.96,d.f.=38 | 49.3%, *P*=0.000 | 0.0568 | 4.35, *P*=0.000 |  |
|  | Posterior circulation | 2 | 7.048(1.913-25.963) | 0.04,d.f.=1 | 0.0%, *P*=0.838 | 0.0000 | 2.94, *P*=0.003 |  |
|  | Tandem lesion occluded | 3 | 1.427(0.739-2.757) | 4.94, d.f.=1 | 59.5%, *P*=0.085 | 0.1879 | 1.06, *P*=0.290 | 0.497 |
|  | Ethnicity |  |  |  |  |  |  |  |
|  | Caucasian | 45(46) | 1.401(1.261-1.555) | 83.12,d.f.=45 | 45.9%, *P*=0.000 | 0.0490 | 6.30, *P*=0.000 |  |
|  | Caucasian and Asian | 2 | 0.883(0.518-1.504) | 0.37,d.f.=1 | 0.0%, *P*=0.541 | 0.0000 | 0.46, *P*=0.646 |  |
|  | Asian | 11 | 1.297(0.978-1.720) | 22.05,d.f.=10 | 54.7%, *P*=0.015 | 0.1017 | 1.80, *P*=0.071 |  |
| **Good outcome in-hospital/ at discharge** | **All** | **8** | **1.691(1.203-2.377)** | **18.38,d.f.=7** | **61.9%, *P*=0.010** | **0.1282** | **3.02, *P*=0.003** | **0.277** |
|  | Study type |  |  |  |  |  |  |  |
|  | Prospective | 4 | 2.162(1.130-4.137) | 12.31,d.f.=3 | 75.6%, *P*=0.006 | 0.3025 | 2.33, *P*=0.020 |  |
|  | Retrospective | 4 | 1.403(0.892-2.206) | 5.95,d.f.=3 | 49.6%, *P*=0.114 | 0.1033 | 1.47, *P*=0.143 |  |
|  | Location of occluded artery |  |  |  |  |  |  |  |
|  | Anterior circulation | 1 | 3.476(1.934-6.248) | 0.00,d.f.=0 | NA | 0.0000 | 4.16, *P*=0.000 |  |
|  | Anterior and posterior circulation | 7 | 1.459(1.100-1.934) | 9.61,d.f.=6 | 37.6%, *P*=0.142 | 0.0482 | 2.62, *P*=0.009 |  |
|  | Ethnicity |  |  |  |  |  |  |  |
|  | Caucasian | 7 | 1.751(1.135-2.701) | 18.36,d.f.=6 | 67.3%, *P*=0.005 | 0.2043 | 2.53, *P*=0.011 |  |
|  | Asian | 1 | 1.530(1.031-2.271) | 0.00,d.f.=0 | NA | 0.0000 | 2.11, *P*=0.035 |  |
| **Good outcome at 6 months/ 1 year** | **All** | **2** | **2.065(0.235-18.146)** | **5.22,d.f.=1** | **80.9%, *P*=0.022** | **1.9932** | **0.65, *P*=0.513** | **NA** |
| **Favorable outcome at 90 days** | **All** | **6** | **1.278(0.919-1.778)** | **11.83,d.f.=5** | **57.7%, *P*=0.037** | **0.0776** | **1.46, *P*=0.145** | **0.952** |
|  | Study type |  |  |  |  |  |  |  |
|  | Prospective | 1 | 0.841(0.618-1.144) | 0.00,d.f.=0 | NA | 0.0000 | 1.10, *P*=0.270 |  |
|  | Retrospective | 5 | 1.471(1.203-1.799) | 2.96,d.f.=4 | 0.0%, *P*=0.565 | 0.0000 | 3.76, *P*=0.000 |  |
|  | Location of occluded artery |  |  |  |  |  |  |  |
|  | Anterior and posterior circulation | 1 | 2.429(0.725-8.135) | 0.00,d.f.=0 | NA | 0.0000 | 1.44, *P*=0.150 |  |
|  | Anterior circulation | 4 | 1.250(0.888-1.760) | 9.48,d.f.=3 | 68.3%, *P*=0.024 | 0.0771 | 1.28, *P*=0.201 |  |
|  | Posterior circulation | 1 | 0.225(0.010-4.968) | 0.00,d.f.=0 | NA | 0.0000 | 0.94, *P*=0.345 |  |
|  | Ethnicity |  |  |  |  |  |  |  |
|  | Caucasian | 4 | 1.483(1.212-1.814) | 1.54,d.f.=3 | 0.0%, *P*=0.674 | 0.0000 | 3.83, *P*=0.000 |  |
|  | Asian | 2 | 0.830(0.611-1.128) | 0.69,d.f.=1 | 0.0%, *P*=0.406 | 0.0000 | 1.19, *P*=0.234 |  |
| **All mortality** | **All** | **48(49)** | **0.624(0.566-0.688)** | **65.16,d.f.=48** | **26.3%, *P*=0.050** | **0.0269** | **9.49, *P*=0.000** | **0.204** |
|  | Study type |  |  |  |  |  |  |  |
|  | Prospective | 18(19) | 0.615(0.527-0.716) | 31.24,d.f.=18 | 42.4%, *P*=0.027 | 0.0409 | 6.22, *P*=0.000 |  |
|  | Retrospective | 30 | 0.636(0.560-0.722) | 33.58,d.f.=29 | 13.6%, *P*=0.255 | 0.0160 | 6.98, *P*=0.000 |  |
|  | Location of occluded artery |  |  |  |  |  |  |  |
|  | Anterior and posterior circulation | 16 | 0.608(0.527-0.701) | 6.71,d.f.=15 | 0.0%, *P*=0.965 | 0.0000 | 6.85, *P*=0.000 |  |
|  | Anterior circulation | 29(30) | 0.619(0.537-0.715) | 51.32,d.f.=29 | 43.5%, *P*=0.006 | 0.0557 | 6.55, *P*=0.000 |  |
|  | Posterior circulation | 3 | 0.570(0.258-1.257) | 5.07,d.f.=2 | 60.6%, *P*=0.079 | 0.2670 | 1.39, *P*=0.163 |  |
|  | Ethnicity |  |  |  |  |  |  |  |
|  | Caucasian | 40(41) | 0.615(0.561-0.675) | 47.02,d.f.=40 | 14.9%, *P*=0.207 | 0.0124 | 10.28, *P*=0.000 |  |
|  | Asian | 7 | 0.706(0.451-1.106) | 12.59,d.f.=6 | 52.4%, *P*=0.050 | 0.1430 | 1.52, *P*=0.128 |  |
|  | Caucasian and Asian | 1 | 0.288(0.104-0.800) | 0.00,d.f.=0 | NA | 0.0000 | 2.39, *P*=0.017 |  |
| **Mortality within 90 days** | **All** | **41(42)** | **0.619(0.560-0.684)** | **53.99,d.f.=41** | **24.1%, *P*=0.084** | **0.0228** | **9.39, *P*=0.000** | **0.236** |
|  | Study type |  |  |  |  |  |  |  |
|  | Prospective | 17(18) | 0.628(0.539-0.732) | 28.81, d.f.=17 | 41.0%, *P*=0.036 | 0.0373 | 5.97, *P*=0.000 |  |
|  | Retrospective | 24 | 0.612(0.535-0.701) | 25.15, d.f.=23 | 8.6%, *P*=0.342 | 0.0094 | 7.14, *P*=0.000 |  |
|  | Location of occluded artery |  |  |  |  |  |  |  |
|  | Anterior and posterior circulation | 14 | 0.608(0.526-0.704) | 4.91,d.f.=13 | 0.0%, *P*=0.977 | 0.0000 | 6.66, *P*=0.000 |  |
|  | Anterior circulation | 25(26) | 0.627(0.540-0.728) | 46.32,d.f.=25 | 46.0%, *P*=0.006 | 0.0560 | 6.14, *P*=0.000 |  |
|  | Posterior circulation | 2 | 0.367(0.172-0.783) | 0.73,d.f.=1 | 0.0%, *P*=0.391 | 0.0000 | 2.59, *P*=0.009 |  |
|  | Ethnicity |  |  |  |  |  |  |  |
|  | Caucasian | 33(34) | 0.606(0.553-0.664) | 35.43,d.f.=33 | 6.9%, *P*=0.354 | 0.0049 | 10.79, *P*=0.000 |  |
|  | Asian | 7 | 0.706(0.451-1.106) | 12.59,d.f.=6 | 52.4%, *P*=0.050 | 0.1430 | 1.52, *P*=0.128 |  |
|  | Caucasian and Asian | 1 | 0.288(0.104-0.800) | 0.00,d.f.=0 | NA | 0.0000 | 2.39, *P*=0.017 |  |
| **In-hospital mortality** | **All** | **11** | **0.714(0.592-0.862) ^#^** | **8.66,d.f.=10** | **0.0%, *P*=0.564** | **NA** | **3.50, *P*=0.000** | **0.850** |
|  | Study type |  |  |  |  |  |  |  |
|  | Prospective | 4 | 0.633(0.472-0.849) ^#^ | 1.86,d.f.=3 | 0.0%, *P*=0.601 | NA | 3.05, *P*=0.002 |  |
|  | Retrospective | 7 | 0.777(0.608-0.993) ^#^ | 5.69,d.f.=6 | 0.0%, *P*=0.459 | NA | 2.02, *P*=0.044 |  |
|  | Location of occluded artery |  |  |  |  |  |  |  |
|  | Anterior circulation | 5 | 0.587(0.395-0.871) ^#^ | 5.47,d.f.=4 | 26.9%, *P*=0.242 | NA | 2.65, *P*=0.008 |  |
|  | Anterior and posterior circulation | 5 | 0.670(0.502-0.894) ^#^ | 0.42,d.f.=4 | 0.0%, *P*=0.981 | NA | 2.72, *P*=0.006 |  |
|  | Posterior circulation | 1 | 0.879(0.639-1.210) ^#^ | 0.00,d.f.=0 | NA | NA | 0.79, *P*=0.428 |  |
| **Successful reperfusion** | **All** | **55(56)** | **1.271(1.149-1.406)** | **84.20, d.f.=55** | **34.7%, *P*=0.007** | **0.0380** | **4.65, *P=* 0.000** | **0.894** |
|  | Study type |  |  |  |  |  |  |  |
|  | Prospective | 21(22) | 1.281(1.173-1.399) | 20.36, d.f.=21 | 0.0%, *P*=0.498 | 0.0000 | 5.50, *P=* 0.000 |  |
|  | Retrospective | 34 | 1.271(1.072-1.506) | 63.83, d.f.=33 | 48.3%, *P*=0.001 | 0.0992 | 2.76, *P=* 0.006 |  |
|  | Location of occluded artery |  |  |  |  |  |  |  |
|  | Anterior and posterior circulation | 19 | 1.217(1.053-1.407) | 15.62, d.f.=18 | 0.0%, *P*=0.619 | 0.0000 | 2.65, *P=* 0.008 |  |
|  | Anterior circulation | 36(37) | 1.302(1.140-1.488) | 67.88, d.f.=36 | 47.0%, *P*=0.001 | 0.0556 | 3.88, *P=* 0.000 |  |
|  | Tandem lesion occluded | 2 | 1.552(1.138-2.117)^#^ | 1.10, d.f.=1 | 9.5%, *P*=0.293 | NA | 2.77, *P=* 0.006 |  |
|  | Ethnicity |  |  |  |  |  |  |  |
|  | Caucasian | 47(48) | 1.290(1.170-1.422) | 62.63, d.f.=47 | 25.0%, *P*=0.063 | 0.0227 | 5.10, *P=* 0.000 |  |
|  | Asian | 7 | 1.206(0.731-1.989) | 19.44, d.f.=6 | 69.1%, *P*=0.003 | 0.2630 | 0.73, *P=* 0.464 |  |
|  | Caucasian and Asian | 1 | 0.704(0.312-1.589) | 0.00,d.f.=0 | NA | 0.0000 | 0.85, *P=* 0.398 |  |
| **Complete reperfusion** | **All** | **20** | **1.084(0.947-1.241)** | **32.07, d.f.=19** | **40.8%, *P*=0.031** | **0.0329** | **1.18, *P=* 0.240** | **0.926** |
|  | Study type |  |  |  |  |  |  |  |
|  | Prospective | 8 | 1.021(0.888-1.173) | 7.76, d.f.=7 | 9.8%, *P*=0.354 | 0.0041 | 0.29, *P=* 0.772 |  |
|  | Retrospective | 12 | 1.168(0.935-1.460) | 22.46, d.f.=11 | 51.0%, *P*=0.021 | 0.0677 | 1.37, *P=* 0.171 |  |
|  | Location of occluded artery |  |  |  |  |  |  |  |
|  | Anterior and posterior circulation | 7 | 1.033(0.858-1.243) | 3.72, d.f.=6 | 0.0%, *P*=0.715 | 0.0000 | 0.34, *P=* 0.733 |  |
|  | Anterior circulation | 13 | 1.113(0.919-1.349) | 27.93, d.f.=12 | 57.0%, *P*=0.006 | 0.0575 | 1.10, *P=* 0.272 |  |
|  | Ethnicity |  |  |  |  |  |  |  |
|  | Caucasian | 18 | 1.061(0.929-1.211) | 27.71, d.f.=17 | 38.7%, *P*=0.048 | 0.0277 | 0.87, *P=* 0.385 |  |
|  | Asian | 2 | 1.964(1.012-3.811) | 1.06, d.f.=1 | 5.4%, *P*=0.304 | 0.0124 | 2.00, *P=* 0.046 |  |
| **Any bleeding** | **All** | **53(61)** | **1.095(0.978-1.226)** | **84.8, d.f.=60** | **29.2%, *P*=0.019** | **0.0471** | **1.57, *P*=0.116** | **0.996** |
|  | Study type |  |  |  |  |  |  |  |
|  | Prospective | 19(22) | 1.077(0.956-1.214) | 19.22, d.f.=21 | 0.0%, *P*=0.571 | 0.0000 | 1.22, *P*=0.223 |  |
|  | Retrospective | 34(39) | 1.091(0.917-1.297) | 65.49, d.f.=38 | 42.0%, *P*=0.004 | 0.1031 | 0.98, *P*=0.325 |  |
|  | Location of occluded artery |  |  |  |  |  |  |  |
|  | Anterior circulation | 33(38) | 1.162(1.031-1.310) | 44.70, d.f.=37 | 17.2%, *P*=0.180 | 0.0207 | 2.46, *P*=0.014 |  |
|  | Anterior and posterior circulation | 18(21) | 1.052(0.860-1.287) | 23.51, d.f.=20 | 14.9%, *P*=0.264 | 0.0306 | 0.49, *P*=0.622 |  |
|  | Posterior circulation | 2 | 0.496(0.260-0.943) | 1.07, d.f.=1 | 6.4%, *P*=0.301 | 0.0640 | 2.14, *P*=0.033 |  |
|  | Ethnicity |  |  |  |  |  |  |  |
|  | Caucasian | 45(51) | 1.066(0.935-1.216) | 74.32, d.f.=50 | 32.7%, *P*=0.014 | 0.0581 | 0.96, *P*=0.337 |  |
|  | Asian | 7(9) | 1.191(0.973-1.458) | 6.75, d.f.=8 | 0.0%, *P*=0.564 | 0.0000 | 1.69, *P*=0.090 |  |
|  | Caucasian and Asian | 1 | 1.719(0.989-2.988) | 0.00,d.f.=0 | NA | 0.0000 | 1.92, *P*=0.055 |  |
| **Any ICH** | **All** | **46(54)** | **1.153(1.026-1.295)** | **68.92, d.f.=53** | **23.1%, *P*=0.070** | **0.0348** | **2.39, *P*=0.017** | **0.550** |
|  | Study type |  |  |  |  |  |  |  |
|  | Prospective | 17(20) | 1.075(0.949-1.219) | 14.47, d.f.=19 | 0.0%, *P*=0.756 | 0.0000 | 1.14, *P*=0.256 |  |
|  | Retrospective | 29(34) | 1.194(0.992-1.437) | 53.17, d.f.=33 | 37.9%, *P*=0.014 | 0.0907 | 1.88, *P*=0.061 |  |
|  | Location of occluded artery |  |  |  |  |  |  |  |
|  | Anterior circulation | 29(35) | 1.179(1.064-1.306) | 33.78, d.f.=34 | 0.0%, *P*=0.478 | 0.0000 | 3.15, *P*=0.002 |  |
|  | Anterior and posterior circulation | 15(17) | 1.182(0.943-1.483) | 18.01, d.f.=16 | 11.1%, *P*=0.323 | 0.0241 | 1.45, *P*=0.147 |  |
|  | Posterior circulation | 2 | 0.496(0.260-0.943) | 1.07, d.f.=1 | 6.4%, *P*=0.301 | 0.0640 | 2.14, *P*=0.033 |  |
|  | Ethnicity |  |  |  |  |  |  |  |
|  | Caucasian | 40(46) | 1.139(0.995-1.305) | 63.19, d.f.=45 | 28.8%, *P*=0.038 | 0.0486 | 1.88, *P*=0.060 |  |
|  | Asian | 6(8) | 1.259(1.015-1.562) | 4.61, d.f.=7 | 0.0%, *P*=0.708 | 0.0000 | 2.09, *P*=0.036 |  |
| **aICH** | **All** | **7** | **1.524(1.233-1.882) ^#^** | **6.19, d.f.=6** | **3.1%, *P*=0.402** | **NA** | **3.91, *P*=0.000** | **0.088** |
|  | Study type |  |  |  |  |  |  |  |
|  | Prospective | 2 | 1.215(0.895-1.649) ^#^ | 1.91, d.f.=1 | 47.5%, *P*=0.167 | NA | 1.25, *P*=0.213 |  |
|  | Retrospective | 5 | 1.874(1.399-2.510) ^#^ | 0.25, d.f.=4 | 0.0%, *P*=0.993 | NA | 4.21, *P*=0.000 |  |
|  | Location of occluded artery |  |  |  |  |  |  |  |
|  | Anterior circulation | 5 | 1.461(1.163-1.834) ^#^ | 5.24, d.f.=4 | 23.7%, *P*=0.263 | NA | 3.26, *P*=0.001 |  |
|  | Anterior and posterior circulation | 2 | 1.977(1.122-3.481) ^#^ | 0.01, d.f.=1 | 0.0%, *P*=0.941 | NA | 2.36, *P*=0.018 |  |
|  | Ethnicity |  |  |  |  |  |  |  |
|  | Caucasian | 5 | 2.021(1.401-2.917) ^#^ | 0.22, d.f.=4 | 0.0%, *P*=0.994 | NA | 3.76, *P*=0.000 |  |
|  | Asian | 2 | 1.378(0.896-2.117) | 2.56, d.f.=1 | 61.0%, *P*=0.110 | 0.0594 | 1.46, *P*=0.144 |  |
| **sICH** | **All** | **36(37)** | **1.062(0.915-1.232) ^#^** | **29.50, d.f.=36** | **0.0%, *P*=0.770** | NA | **0.79, *P*=0.429** | **0.515** |
|  | Study type |  |  |  |  |  |  |  |
|  | Prospective | 14(15) | 1.079(0.867-1.342) ^#^ | 11.70, d.f.=14 | 0.0%, *P*=0.630 | NA | 0.68, *P*=0.497 |  |
|  | Retrospective | 22 | 1.047(0.855-1.282) ^#^ | 17.76, d.f.=21 | 0.0%, *P*=0.664 | NA | 0.45, *P*=0.654 |  |
|  | Location of occluded artery |  |  |  |  |  |  |  |
|  | Anterior circulation | 24(25) | 1.067(0.900-1.266) ^#^ | 15.51, d.f.=24 | 0.0%, *P*=0.905 | NA | 0.75, *P*=0.456 |  |
|  | Anterior and posterior circulation | 12 | 1.045(0.773-1.412) ^#^ | 13.97, d.f.=11 | 21.3%, *P*=0.234 | NA | 0.29, *P*=0.775 |  |
|  | Ethnicity |  |  |  |  |  |  |  |
|  | Caucasian | 32(33） | 1.045(0.892-1.224) ^#^ | 28.46, d.f.=32 | 0.0%, *P*=0.646 | NA | 0.55, *P*=0.583 |  |
|  | Asian | 4 | 1.190(0.775-1.827) ^#^ | 0.73, d.f.=3 | 0.0%, *P*=0.867 | NA | 0.80, *P*=0.426 |  |
| **SAH** | **All** | **9** | **0.820(0.566-1.187) ^#^** | **2.84, d.f.=8** | **0.0%, *P*=0.944** | NA | **1.05, *P*=0.292** | **0.786** |
|  | Study type |  |  |  |  |  |  |  |
|  | Prospective | 4 | 0.669(0.380-1.179) ^#^ | 0.67, d.f.=3 | 0.0%, *P*=0.881 | NA | 1.39, *P*=0.164 |  |
|  | Retrospective | 5 | 0.954(0.585-1.554) ^#^ | 1.31, d.f.=4 | 0.0%, *P*=0.859 | NA | 0.19, *P*=0.849 |  |
|  | Location of occluded artery |  |  |  |  |  |  |  |
|  | Anterior circulation | 3 | 0.921(0.192-4.419) ^#^ | 1.17, d.f.=2 | 0.0%, *P*=0.558 | NA | 0.10, *P*=0.918 |  |
|  | Anterior and posterior circulation | 6 | 0.814(0.556-1.191) ^#^ | 1.65, d.f.=5 | 0.0%, *P*=0.895 | NA | 1.06, *P*=0.290 |  |
| **HI** | **All** | **6(11)** | **1.236(0.989-1.544) ^#^** | **13.82, d.f.=10** | **27.6%, *P*=0.181** | **NA** | **1.86, *P*=0.063** | **0.160** |
|  | Study type |  |  |  |  |  |  |  |
|  | Prospective | 3(6) | 1.103(0.704-1.728) ^#^ | 2.10, d.f.=5 | 0.0%, *P*=0.836 | NA | 0.43, *P*=0.668 |  |
|  | Retrospective | 3(5) | 0.882(0.450-1.730) | 11.40, d.f.=4 | 64.9%, *P*=0.022 | 0.3501 | 0.36, *P*=0.715 |  |
|  | Location of occluded artery |  |  |  |  |  |  |  |
|  | Anterior circulation | 3(5) | 1.329(1.016-1.737) ^#^ | 6.35, d.f.=4 | 37.0%, *P*=0.175 | NA | 2.08, *P*=0.038 |  |
|  | Anterior and posterior circulation | 3(6) | 1.050(0.703-1.568) ^#^ | 6.56, d.f.=5 | 23.7%, *P*=0.256 | NA | 0.24, *P*=0.811 |  |
|  | Subtype | 10^‡^ | 0.907(0.639-1.288) ^#^ | 8.80, d.f.=9 | 0.0%, *P*=0.456 | NA | 0.55, *P*=0.585 |  |
|  | HI-1 | 5 | 0.748(0.464-1.204) ^#^ | 3.85, d.f.=4 | 0.0%, *P*=0.427 | NA | 1.20, *P*=0.231 |  |
|  | HI-2 | 5 | 1.139(0.679-1.911) ^#^ | 3.58, d.f.=4 | 0.0%, *P*=0.466 | NA | 0.49, *P*=0.622 |  |
| **HT** | **All** | **19(29)** | **1.152(1.021-1.301) ^#^** | **28.83, d.f.=28** | **2.9%, *P*=0.421** | **NA** | **2.29, *P*=0.022** | **0.160** |
|  | Study type |  |  |  |  |  |  |  |
|  | Prospective | 7(13) | 1.332(1.045-1.697) ^#^ | 5.74, d.f.=12 | 0.0%, *P*=0.929 | NA | 2.32, *P*=0.020 |  |
|  | Retrospective | 12(16) | 1.098(0.954-1.263) ^#^ | 21.26, d.f.=15 | 29.4%, *P*=0.129 | NA | 1.31, *P*=0.192 |  |
|  | Location of occluded artery |  |  |  |  |  |  |  |
|  | Anterior circulation | 11(17) | 1.192(1.039-1.367) ^#^ | 16.43, d.f.=16 | 2.6%, *P*=0.423 | NA | 2.50, *P*=0.012 |  |
|  | Anterior and posterior circulation | 8(12) | 1.023(0.790-1.324) ^#^ | 11.35, d.f.=11 | 3.1%, *P*=0.414 | NA | 0.17, *P*=0.866 |  |
|  | Ethnicity |  |  |  |  |  |  |  |
|  | Caucasian | 17(27) | 1.148(1.011-1.304) ^#^ | 25.23, d.f.=26 | 0.0%, *P*=0.506 | NA | 2.13, *P*=0.033 |  |
|  | Caucasian and Asian | 1 | 1.719(0.989-2.988) ^#^ | 0.00,d.f.=0 | NA | NA | 1.92, *P*=0.055 |  |
|  | Asian | 1 | 0.790(0.440-1.419) ^#^ | 0.00,d.f.=0 | NA | NA | 0.79, *P*=0.430 |  |
| **PH** | **All** | **15(19)** | **1.092(0.894-1.332) ^#^** | **14.84, d.f.=18** | **0.0%, *P*=0.673** | **NA** | **0.86, *P*=0.388** | **0.834** |
|  | Study type |  |  |  |  |  |  |  |
|  | Prospective | 5(6) | 1.347(0.962-1.888) ^#^ | 2.14, d.f.=5 | 0.0%, *P*=0.829 | NA | 1.73, *P*=0.083 |  |
|  | Retrospective | 10(13) | 0.975(0.762-1.248) ^#^ | 10.39, d.f.=12 | 0.0%, *P*=0.582 | NA | 0.20, *P*=0.840 |  |
|  | Location of occluded artery |  |  |  |  |  |  |  |
|  | Anterior circulation | 10(12) | 1.150(0.928-1.426) ^#^ | 7.34, d.f.=11 | 0.0%, *P*=0.771 | NA | 1.28, *P*=0.201 |  |
|  | Anterior and posterior circulation | 5(7) | 0.789(0.462-1.345) ^#^ | 5.84, d.f.=6 | 0.0%, *P*=0.441 | NA | 0.87, *P*=0.383 |  |
|  | Subtype | 12^§^ | 1.048(0.743-1.477) ^#^ | 11.02,d.f.=11 | 0.1%, *P*=0.442 | NA | 0.27, *P*=0.790 |  |
|  | PH-1 | 5 | 0.797(0.488-1.302) ^#^ | 4.28,d.f=4 | 6.6%, *P*=0.369 | NA | 0.91, *P*=0.365 |  |
|  | PH-2 | 7 | 1.362(0.843-2.201) ^#^ | 4.39,d.f.=6 | 0.0%, *P*=0.624 | NA | 1.26, *P*=0.207 |  |
| **Early recovery at 24 hours after admission** | **All** | **6** | **1.306(0.906-1.881)** | **9.91,d.f.=5** | **49.6%, *P*=0.078** | **0.0913** | **1.43, *P*=0.153** | **0.785** |
|  | Study type |  |  |  |  |  |  |  |
|  | Prospective | 2 | 1.000(0.495-2.019) | 4.47,d.f.=1 | 77.6%, *P*=0.034 | 0.2028 | 0.00, *P*=0.1000 |  |
|  | Retrospective | 4 | 1.618(0.993-2.639) | 4.14,d.f.=3 | 27.6%, *P*=0.246 | 0.0734 | 1.93, *P*=0.054 |  |
|  | Ethnicity |  |  |  |  |  |  |  |
|  | Caucasian | 5 | 1.226(0.834-1.801) | 8.49,d.f.=4 | 52.9%, *P*=0.075 | 0.0917 | 1.04, *P*=0.300 |  |
|  | Asian | 1 | 2.375(0.854-6.605) | 0.00,d.f.=0 | NA | 0.0000 | 1.66, *P*=0.097 |  |
| **Dramatic improvement at discharge/ 7days** | **All** | **5** | **1.402(1.143-1.719) ^#^** | **0.65,d.f.=4** | **0.0%, *P*=0.958** | **NA** | **3.25, *P*=0.001** | **0.756** |
|  | Study type |  |  |  |  |  |  |  |
|  | Prospective | 2 | 1.352(1.068-1.713) **^#^** | 0.03,d.f.=1 | 0.0%, *P*=0.863 | NA | 2.50, *P*=0.012 |  |
|  | Retrospective | 3 | 1.556(1.040-2.326) **^#^** | 0.27,d.f.=2 | 0.0%, *P*=0.873 | NA | 2.15, *P*=0.031 |  |
|  | Location of occluded artery |  |  |  |  |  |  |  |
|  | Anterior and posterior circulation | 3 | 1.362(1.089-1.702) **^#^** | 0.06,d.f.=2 | 0.0%, *P*=0.970 | NA | 2.71, *P*=0.007 |  |
|  | Anterior circulation | 2 | 1.621(0.983-2.673) **^#^** | 0.20,d.f.=1 | 0.0%, *P*=0.657 | NA | 1.89, *P*=0.058 |  |
| **Good improvement at 3 months** | **All** | **2** | **1.499(0.866-2.595) ^#^** | **0.93,d.f.=1** | **0.0%, *P*=0.335** | **NA** | **1.45, *P*=0.148** | **NA** |
| **Good improvement at**  **discharge/ 7days** | **All** | **3** | **2.623(0.993-6.931)** | **7.65,d.f.=2** | **73.9%, *P*=0.022** | **0.5367** | **1.95, *P*=0.052** | **0.675** |
|  | Study type |  |  |  |  |  |  |  |
|  | Prospective | 1 | 1.788(0.566-5.649) | 0.00,d.f.=0 | NA | 0.0000 | 0.99, *P*=0.322 |  |
|  | Retrospective | 2 | 3.125(0.720-13.554) | 7.45,d.f.=1 | 86.6%, *P*=0.006 | 0.9734 | 1.52, *P*=0.128 |  |
|  | Location of occluded artery |  |  |  |  |  |  |  |
|  | Anterior and posterior circulation | 2 | 3.686(0.985-13.796) | 3.26,d.f.=1 | 69.4%, *P*=0.071 | 0.6323 | 1.94, *P*=0.053 |  |
|  | Anterior circulation | 1 | 1.540(0.862-2.752) | 0.00,d.f.=0 | NA | 0.0000 | 1.46, *P*=0.145 |  |
| **Number of passes of the thrombectomy device≤2** | **All** | **10** | **1.870(1.344-2.603)** | **30.77, d.f.=9** | **70.7%, *P*=0.000** | **0.1539** | **3.71, *P*=0.000** | **0.024** |
|  | Study type |  |  |  |  |  |  |  |
|  | Prospective | 4 | 1.287(0.947-1.750) | 4.48,d.f.=3 | 33.0%, *P*=0.214 | 0.0331 | 1.61, *P*=0.107 |  |
|  | Retrospective | 6 | 2.685(1.423-5.065) | 24.12,d.f.=5 | 79.3%, *P*=0.000 | 0.4570 | 3.05, *P*=0.002 |  |
|  | Location of occluded artery |  |  |  |  |  |  |  |
|  | Anterior circulation | 8 | 1.438(1.145-1.806) | 11.43,d.f.=7 | 38.8%, *P*=0.121 | 0.0340 | 3.13, *P*=0.002 |  |
|  | Anterior and posterior circulation | 2 | 5.825(0.978-34.697) | 6.63,d.f.=1 | 84.9%, *P*=0.010 | 1.4103 | 1.94, *P*=0.05 |  |
| **Number of passes of the thrombectomy device=1** | **All** | **3** | **1.605(0.926-2.781)** | **4.69,d.f.=2** | **57.3%, *P*=0.096** | **0.1346** | **1.69, *P*=0.092** | **0.521** |
|  | Study type |  |  |  |  |  |  |  |
|  | Prospective | 1 | 1.488(0.515-4.298) | 0.00,d.f.=0 | NA | 0.0000 | 0.73, *P*=0.463 |  |
|  | Retrospective | 2 | 1.691(0.775-3.689) | 4.66,d.f.=1 | 78.5%, *P*=0.031 | 0.2545 | 1.32, *P*=0.187 |  |
| **Any procedural complications** | **All** | **18(33)** | **1.069(0.871-1.312)** | **68.15,d.f.=32** | **53.0%, *P*=0.000** | **0.1457** | **0.64, *P*=0.525** | **0.287** |
|  | Study type |  |  |  |  |  |  |  |
|  | Prospective | 6(18) | 1.016(0.848-1.219) | 23.46,d.f.=17 | 27.5%, *P*=0.135 | 0.0370 | 0.18, *P*=0.860 |  |
|  | Retrospective | 12(15) | 1.243(0.759-2.035) | 44.69,d.f.=14 | 68.7%, *P*=0.000 | 0.5161 | 0.86, *P*=0.388 |  |
|  | Location of occluded artery |  |  |  |  |  |  |  |
|  | Anterior circulation | 12(20) | 0.969(0.791-1.188) | 32.72,d.f.=19 | 41.9%, *P*=0.026 | 0.0730 | 0.30, *P*=0.762 |  |
|  | Anterior and posterior circulation | 6(13) | 1.327(0.816-2.158) | 32.10,d.f.=12 | 62.6%, *P*=0.001 | 0.4133 | 1.14, *P*=0.255 |  |
|  | Ethnicity |  |  |  |  |  |  |  |
|  | Caucasian | 15(30) | 1.051(0.845-1.306) | 63.06,d.f.=29 | 54.0%, *P*=0.000 | 0.1522 | 0.44, *P*=0.656 |  |
|  | Asian | 3 | 1.483(0.561-3.919) | 4.95,d.f.=2 | 59.6%, *P*=0.084 | 0.4421 | 0.79, *P*=0.427 |  |
| **Clot migration** | **All** | **14** | **1.071(0.731-1.570)** | **42.31,d.f.=13** | **69.3%, *P*=0.000** | **0.2895** | **0.35, *P*=0.724** | **0.401** |
|  | Study type |  |  |  |  |  |  |  |
|  | Prospective | 6 | 0.928(0.719-1.198) | 5.73,d.f.=5 | 12.7%, *P*=0.334 | 0.0132 | 0.57, *P*=0.566 |  |
|  | Retrospective | 8 | 1.100(0.475-2.550) | 36.52,d.f.=7 | 80.8%, *P*=0.000 | 0.9401 | 0.22, *P*=0.823 |  |
|  | Location of occluded artery |  |  |  |  |  |  |  |
|  | Anterior circulation | 9 | 0.968(0.600-1.560) | 27.87,d.f.=8 | 71.3%, *P*=0.000 | 0.2916 | 0.14, *P*=0.892 |  |
|  | Anterior and posterior circulation | 5 | 1.371(0.641-2.932) | 14.14,d.f.=4 | 71.7%, *P*=0.007 | 0.4873 | 0.81, *P*=0.416 |  |
|  | Ethnicity |  |  |  |  |  |  |  |
|  | Caucasian | 13 | 1.107(0.719-1.704) | 42.23,d.f.=12 | 71.6%, *P*=0.000 | 0.3501 | 0.46, *P*=0.645 |  |
|  | Asian | 1 | 0.868(0.521-1.446) | 0.00,d.f.=0 | NA | 0.0000 | 0.54, *P*=0.586 |  |
| **Groin hematoma** | **All** | **4** | **1.948(0.744-5.100) ^#^** | **0.84,d.f.=3** | **0.0%, *P*=0.840** | **NA** | **1.36, *P*=0.174** | **0.668** |
|  | Study type |  |  |  |  |  |  |  |
|  | Prospective | 2 | 1.301(0.343-4.933) ^#^ | 0.01,d.f.=1 | 0.0%, *P*=0.931 | NA | 0.39, *P*=0.699 |  |
|  | Retrospective | 2 | 3.025(0.753-12.156) ^#^ | 0.09,d.f.=1 | 0.0%, *P*=0.759 | NA | 1.56, *P*=0.119 |  |
|  | Location of occluded artery |  |  |  |  |  |  |  |
|  | Anterior circulation | 2 | 3.025(0.753-12.156) ^#^ | 0.09,d.f.=1 | 0.0%, *P*=0.759 | NA | 1.56, *P*=0.119 |  |
|  | Anterior and posterior circulation | 2 | 1.301(0.343-4.933) ^#^ | 0.01,d.f.=1 | 0.0%, *P*=0.931 | NA | 0.39, *P*=0.699 |  |
|  | Ethnicity |  |  |  |  |  |  |  |
|  | Caucasian | 3 | 1.772(0.643-4.883) ^#^ | 0.50,d.f.=2 | 0.0%, *P*=0.780 | NA | 1.11, *P*=0.268 |  |
|  | Asian | 1 | 4.639(0.216-99.671) ^#^ | 0.00,d.f.=0 | NA | NA | 0.98, *P*=0.327 |  |
| **Pneumonia** | **All** | **2** | **0.956(0.684-1.337) ^#^** | **0.18,d.f.=1** | **0.0%, *P*=0.667** | NA | **0.26, *P*=0.792** |  |
| **Rescue therapy** | **All** | **2** | **0.928(0.727-1.185) ^#^** | **0.19,d.f.=1** | **0.0%, *P*=0.663** | NA | **0.60, *P*=0.550** |  |
| **Vasospasm** | **All** | **3** | **1.823(0.599-5.551)** | **8.26,d.f.=2** | **75.8%, *P*=0.016** | **0.6978** | **1.06, *P*=0.291** | **0.829** |
|  | Study type |  |  |  |  |  |  |  |
|  | Prospective | 2 | 1.249(0.372-4.191) | 5.33,d.f.=1 | 81.2%, *P*=0.021 | 0.6218 | 0.36, *P*=0.719 |  |
|  | Retrospective | 1 | 6.500(1.258-33.582) | 0.00,d.f.=0 | NA | 0.0000 | 2.23, *P*=0.025 |  |
|  | Location of occluded artery |  |  |  |  |  |  |  |
|  | Anterior and posterior circulation | 2 | 2.878(1.187-6.977) | 1.41,d.f.=1 | 29.2%, *P*=0.234 | 0.1660 | 2.34, *P*=0.019 |  |
|  | Anterior circulation | 1 | 0.650(0.279-1.514) | 0.00,d.f.=0 | NA | 0.0000 | 1.00, *P*=0.318 |  |
|  | Ethnicity |  |  |  |  |  |  |  |
|  | Caucasian | 2 | 1.249(0.372-4.191) | 5.33,d.f.=1 | 81.2%, *P*=0.021 | 0.6218 | 0.36, *P*=0.719 |  |
|  | Asian | 1 | 6.500(1.258-33.582) | 0.00,d.f.=0 | NA | 0.0000 | 2.23, *P*=0.025 |  |
| **Vessel dissection** | **All** | **3** | **1.246(0.761-2.039) ^#^** | **2.96,d.f.=2** | **32.3%, *P*=0.228** | **NA** | **0.87, *P*=0.382** | **0.068** |
|  | Study type |  |  |  |  |  |  |  |
|  | Prospective | 2 | 1.171(0.405-3.387) | 2.65,d.f.=1 | 62.3%, *P*=0.104 | 0.3768 | 0.29, *P*=0.771 |  |
|  | Retrospective | 1 | 1.010(0.414-2.464) | 0.00,d.f.=0 | NA | 0.0000 | 0.02, *P*=0.983 |  |
|  | Location of occluded artery |  |  |  |  |  |  |  |
|  | Anterior circulation | 2 | 0.835(0.415-1.680) ^#^ | 0.46,d.f.=1 | 0.0%, *P*=0.499 | NA | 0.51, *P*=0.613 |  |
|  | Anterior and posterior circulation | 1 | 1.848(0.923-3.700) ^#^ | 0.00,d.f.=0 | NA | NA | 1.73, *P*=0.083 |  |
|  | Ethnicity |  |  |  |  |  |  |  |
|  | Caucasian | 2 | 1.471(0.851-2.545) ^#^ | 1.10,d.f.=1 | 8.9%, *P*=0.295 | NA | 1.38, *P*=0.167 |  |
|  | Asian | 1 | 0.615(0.199-1.901) ^#^ | 0.00,d.f.=0 | NA | NA | 0.84, *P*=0.398 |  |
| **Vessel perforation** | **All** | **3** | **1.055(0.651-1.709) ^#^** | **0.87,d.f.=2** | **0.0%, *P*=0.649** | **NA** | **0.22, *P*=0.828** | **0.122** |
|  | Location of occluded artery |  |  |  |  |  |  |  |
|  | Anterior and posterior circulation | 2 | 0.699(0.195-2.502) ^#^ | 0.40,d.f.=1 | 0.0%, *P*=0.528 | NA | 0.55, *P*=0.582 |  |
|  | Anterior circulation | 1 | 1.130(0.671-1.903) ^#^ | 0.00,d.f.=0 | NA | NA | 0.46, *P*=0.646 |  |
| **Recurrent stroke** | **All** | **4** | **0.733(0.372-1.443) ^#^** | **0.71, d.f.=3** | **0.0%, *P*=0.871** | **NA** | **0.90, *P*=0.369** | **0.725** |
|  | Study type |  |  |  |  |  |  |  |
|  | Prospective | 2 | 0.683(0.324-1.440) **^#^** | 0.44,d.f.=1 | 0.0%, *P*=0.505 | NA | 1.00, *P*=0.317 |  |
|  | Retrospective | 2 | 1.023(0.200-5.218) **^#^** | 0.07,d.f.=1 | 0.0%, *P*=0.790 | NA | 0.03, *P*=0.978 |  |
|  | Location of occluded artery |  |  |  |  |  |  |  |
|  | Anterior and posterior circulation | 1 | 0.875(0.119-6.425) **^#^** | 0.00,d.f.=0 | NA | NA | 0.13, *P*=0.896 |  |
|  | Anterior circulation | 3 | 0.716(0.348-1.472) **^#^** | 0.67,d.f.=2 | 0.0%, *P*=0.714 | NA | 0.91, *P*=0.363 |  |
|  | Ethnicity |  |  |  |  |  |  |  |
|  | Caucasian | 3 | 0.642(0.227-1.815) **^#^** | 0.60,d.f.=2 | 0.0%, *P*=0.741 | NA | 0.84, *P*=0.403 |  |
|  | Asian | 1 | 0.808(0.330-1.977) **^#^** | 0.00,d.f.=0 | NA | NA | 0.47, *P*=0.641 |  |

Abbreviations: NA, not available or not applicable; d.f., degrees of freedom; cOR, crude odds ratio; cRR, crude risk ratio; ICH, intracranial hemorrhage; SAH, subarachnoid hemorrhage; sICH, symptomatic intracranial hemorrhage; aICH, asymptomatic intracranial hemorrhage; HT, hemorrhagic transformation; PH, parenchymal hematoma; HI, hemorrhagic infarction.

^*^ If one study included many subtypes of the outcomes or many subgroups of the patients, and the subtypes or subgroups were pooled and analyzed into one outcome, we calculated the number of the study and the subtype or subgroup respectively.

^#^ The value was calculated with fixed effects analysis.

^‡^ The study (Nogueira et al. 2015) which analyzed the outcome of HI without the subtype (HI-1 and HI-2) was not included in the subgroup of subtype.

^§^ The studies (Anadani et al. 2019, Rai et al. 2018, Rebello et al. 2016, Kaesmacher et al. 2018, Ferrigno et al. 2018, Gariel et al. 2018, Carmen et al. 2019, Wee et al. 2017 and Nogueira et al. 2015) which analyzed the outcome of PH without the subtype (PH-1 and PH-2) were not included in the subgroup of subtype. The duplicated SWIFT study data for outcome of PH were reported in the study Coutinho et al. 2017 and Raychev et al. 2019, we included the study with larger sample of patients (Raychev et al. 2019) for the outcome of PH. However, the study (Raychev et al. 2019) didn’t analyze the outcome of subtype (PH-1 and PH-2), so the study (Coutinho et al. 2017) was included in the subgroup of subtype.

**Supplemental Table 7.** Overview of the pooled results with adjusted (for potential confounders) analyses

| **Outcome** | **Subgroup** | **Adjusted Analyses** | | | | | | |
| --- | --- | --- | --- | --- | --- | --- | --- | --- |
|  |  | **No. of studies (subgroups/ subtypes)^*^** | **aOR/aRR (95%CI)** | **Heterogeneity** | | | **Overall effect** | **Egger's test** |
|  |  |  |  | **Chi^2^, d.f.** | **I^2^, *P* for Cochran Q** | **Tau^2^** | **Z value, *P* value** | ***P* value** |
| **Excellent outcome at 90 days** | **All** | **12** | **1.328(1.118-1.577)** | **17.56,d.f.=11** | **37.4%, *P=*0.092** | **0.0302** | **3.23, *P=*0.001** | **0.717** |
|  | Study type |  |  |  |  |  |  |  |
|  | Prospective | 6 | 1.381(1.085-1.758) | 10.59,d.f.=5 | 52.8%, *P=*0.060 | 0.0430 | 2.63, *P=*0.009 |  |
|  | Retrospective | 6 | 1.240(0.963-1.596) | 5.91,d.f.=5 | 15.5%, *P=*0.315 | 0.0161 | 1.67, *P=*0.095 |  |
|  | Location of occluded artery |  |  |  |  |  |  |  |
|  | Anterior circulation | 11 | 1.325(1.099-1.598) | 17.55,d.f.=10 | 43.0%, *P=*0.063 | 0.0371 | 2.95, *P* =0.003 |  |
|  | Anterior and posterior circulation | 1 | 1.320(0.764-2.280) | 0.00,d.f.=0 | NA | 0.0000 | 1.00, *P*=0.320 |  |
|  | Ethnicity |  |  |  |  |  |  |  |
|  | Caucasian | 10 | 1.408(1.181-1.680) | 12.71,d.f.=9 | 29.2%, *P=*0.176 | 0.0211 | 3.81, *P=*0.000 |  |
|  | Asian | 2 | 1.018(0.743-1.395) | 0.90,d.f.=1 | 0.0%, *P=*0.343 | 0.0000 | 0.11, *P=*0.909 |  |
| **Good outcome at 90 days** | **All** | **35** | **1.369(1.217-1.540)** | **48.71,d.f.=34** | **30.2%, *P=*0.049** | **0.0312** | **5.23, *P=*0.000** | **0.018** |
|  | Study type |  |  |  |  |  |  |  |
|  | Prospective | 14 | 1.363(1.152-1.613) | 23.86,d.f.=13 | 45.5%, *P=*0.032 | 0.0391 | 3.60, *P=*0.000 |  |
|  | Retrospective | 21 | 1.384(1.166-1.644) | 24.62,d.f.=20 | 18.8%, *P=*0.216 | 0.0277 | 3.71, *P=*0.000 |  |
|  | Location of occluded artery |  |  |  |  |  |  |  |
|  | Anterior and posterior circulation | 10 | 1.608(1.332-1.942) | 6.62,d.f.=9 | 0.0%, *P=*0.677 | 0.0000 | 4.94, *P=*0.000 |  |
|  | Anterior circulation | 24 | 1.264(1.112-1.438) | 31.25,d.f.=23 | 26.4%, *P=*0.117 | 0.0229 | 3.58, *P=*0.000 |  |
|  | Posterior circulation | 1 | 15.450(1.965-121.455) | 0.00, d.f.=0 | NA | 0.0000 | 2.60, *P=*0.009 |  |
|  | Tandem lesion occluded | 1 | 12.045(1.004-144.392) | NA | NA | NA | NA |  |
|  | Ethnicity |  |  |  |  |  |  |  |
|  | Caucasian | 26 | 1.385(1.212-1.582) | 36.50,d.f.=25 | 31.5%, *P=*0.064 | 0.0308 | 4.80, *P=*0.000 |  |
|  | Asian | 9 | 1.334(1.013-1.758) | 11.91,d.f.=8 | 32.9%, *P=*0.155 | 0.0515 | 2.05, *P=*0.040 |  |
| **Good outcome in-hospital/ at discharge** | **All** | **3** | **2.032(1.022-4.043)** | **7.86,d.f.=2** | **74.6%, *P=*0.020** | **0.2741** | **2.02, *P=*0.043** | **0.537** |
|  | Study type |  |  |  |  |  |  |  |
|  | Prospective | 1 | 4.300(2.188-8.452) | 0.00, d.f.=0 | NA | 0.0000 | 4.23, *P=*0.000 |  |
|  | Retrospective | 2 | 1.422(0.969-2.087) | 0.07,d.f.=1 | 0.0%, *P=*0.784 | 0.0000 | 1.80, *P=*0.072 |  |
|  | Location of occluded artery |  |  |  |  |  |  |  |
|  | Anterior circulation | 1 | 4.300(2.188-8.452) | 0.00, d.f.=0 | NA | 0.0000 | 4.23, *P=*0.000 |  |
|  | Anterior and posterior circulation | 2 | 1.422(0.969-2.087) | 0.07,d.f.=1 | 0.0%, *P=*0.784 | 0.0000 | 1.80, *P=*0.072 |  |
|  | Ethnicity |  |  |  |  |  |  |  |
|  | Caucasian | 2 | 2.568(0.937-7.043) | 4.48,d.f.=1 | 77.7%, *P=*0.034 | 0.4116 | 1.83, *P=*0.067 |  |
|  | Asian | 1 | 1.370(0.859-2.186） | 0.00, d.f.=0 | NA | 0.0000 | 1.32, *P=*0.187 |  |
| **Favorable outcome at 90 days** | **All** | **4** | **1.155(0.757-1.764)** | **17.25,d.f.=3** | **82.6%, *P=*0.001** | **0.1402** | **0.67, *P=*0.504** | **0.466** |
|  | Study type |  |  |  |  |  |  |  |
|  | Prospective | 1 | 0.800(0.569-1.125) | 0.00, d.f.=0 | NA | 0.0000 | 1.28, *P=*0.199 |  |
|  | Retrospective | 3 | 1.359(0.769-2.402) | 14.46,d.f.=2 | 86.2%, *P=*0.001 | 0.1965 | 1.06, *P=*0.291 |  |
|  | Location of occluded artery |  |  |  |  |  |  |  |
|  | Anterior circulation | 3 | 1.362(0.756-2.454) | 11.58,d.f.=2 | 82.7%, *P=*0.003 | 0.2063 | 1.03, *P=*0.304 |  |
|  | Posterior circulation | 1 | 0.831(0.654-1.056) | 0.00, d.f.=0 | NA | 0.0000 | 1.51, *P=*0.130 |  |
|  | Ethnicity |  |  |  |  |  |  |  |
|  | Caucasian | 2 | 1.804(1.016-3.201) | 1.89,d.f.=1 | 47.0%, *P=*0.169 | 0.0990 | 2.02, *P=*0.044 |  |
|  | Asian | 2 | 0.821(0.674-0.998) | 0.03,d.f.=1 | 0.0%, *P=*0.858 | 0.0000 | 1.98, *P=*0.048 |  |
| **All mortality** | **All** | **28** | **0.742(0.640-0.860)** | **58.21,d.f.=27** | **53.6%,*P=*0.000** | **0.0594** | **3.96, *P=*0.000** | **0.620** |
|  | Study type |  |  |  |  |  |  |  |
|  | Prospective | 11 | 0.682(0.520-0.894) | 26.90,d.f.=10 | 62.8%, *P=*0.003 | 0.1075 | 2.77, *P=*0.006 |  |
|  | Retrospective | 17 | 0.798(0.666-0.956) | 27.01,d.f.=16 | 40.8%, *P=*0.041 | 0.0428 | 2.45, *P=*0.014 |  |
|  | Location of occluded artery |  |  |  |  |  |  |  |
|  | Anterior and posterior circulation | 8 | 0.722(0.538-0.968) | 13.20,d.f.=7 | 47.0%, *P=*0.067 | 0.0669 | 2.18,*P=*0.029 |  |
|  | Anterior circulation | 18 | 0.763(0.616-0.944) | 41.00,d.f.=17 | 58.5%, *P=*0.001 | 0.1053 | 2.48,*P=*0.013 |  |
|  | Posterior circulation | 2 | 0.577(0.211-1.578) | 3.23,d.f.=1 | 69.0%, *P=*0.072 | 0.3877 | 1.07, *P=*0.284 |  |
|  | Ethnicity |  |  |  |  |  |  |  |
|  | Caucasian | 25 | 0.741(0.632-0.868) | 55.95,d.f.=24 | 57.1%, *P=*0.000 | 0.0656 | 3.70, *P=*0.000 |  |
|  | Asian | 3 | 0.742(0.497-1.109) | 2.20,d.f.=2 | 9.0%, *P=*0.333 | 0.0134 | 1.46, *P=*0.146 |  |
| **Mortality within 90 days** | **All** | **22** | **0.718(0.594-0.868)** | **46.37,d.f.=21** | **54.7%, *P=*0.001** | **0.0954** | **3.43, *P=*0.001** | **0.912** |
|  | Study type |  |  |  |  |  |  |  |
|  | Prospective | 9 | 0.639(0.500-0.816) | 18.31,d.f.=8 | 56.3%, *P=*0.019 | 0.0686 | 3.59, *P=*0.000 |  |
|  | Retrospective | 13 | 0.804(0.601-1.076) | 25.45,d.f.=12 | 52.9%, *P=*0.013 | 0.1380 | 1.47, *P=*0.142 |  |
|  | Location of occluded artery |  |  |  |  |  |  |  |
|  | Anterior and posterior circulation | 5 | 0.584(0.428-0.796) | 1.99,d.f.=4 | 0.0%, *P=*0.738 | 0.0000 | 3.41, *P=*0.001 |  |
|  | Anterior circulation | 16 | 0.778(0.621-0.975) | 39.88,d.f.=15 | 62.4%, *P=*0.000 | 0.1129 | 2.18, *P=*0.029 |  |
|  | Posterior circulation | 1 | 0.298(0.100-0.891) | 0.00,d.f.=0 | NA | 0.0000 | 2.17, *P=*0.030 |  |
|  | Ethnicity |  |  |  |  |  |  |  |
|  | Caucasian | 19 | 0.712(0.577-0.879) | 44.13,d.f.=18 | 59.2%, *P=*0.001 | 0.1104 | 3.16, *P=*0.002 |  |
|  | Asian | 3 | 0.742(0.497-1.109) | 2.20,d.f.=2 | 9.0%, *P=*0.333 | 0.0134 | 1.46, *P=*0.146 |  |
| **In-hospital mortality** | **All** | **6** | **0.805(0.741-0.874) ^#^** | **1.64,d.f.=5** | **0.0%, *P=*0.896** | **NA** | **5.16, *P=*0.000** | **0.461** |
|  | Study type |  |  |  |  |  |  |  |
|  | Prospective | 2 | 0.815(0.422-1.572) **^#^** | 0.09,d.f.=1 | 0.0%, *P=*0.770 | NA | 0.61, *P=*0.542 |  |
|  | Retrospective | 4 | 0.804(0.740-0.874) **^#^** | 1.55,d.f.=3 | 0.0%, *P=*0.670 | NA | 5.13, *P=*0.000 |  |
|  | Location of occluded artery |  |  |  |  |  |  |  |
|  | Anterior circulation | 3 | 0.690(0.386-1.234) **^#^** | 1.22,d.f.=2 | 0.0%, *P=*0.543 | NA | 1.25, *P=*0.211 |  |
|  | Anterior and posterior circulation | 2 | 0.804(0.738-0.876) **^#^** | 0.03,d.f.=1 | 0.0%, *P=*0.873 | NA | 4.99, *P=*0.000 |  |
|  | Posterior circulation | 1 | 0.860(0.593-1.247) **^#^** | 0.00,d.f.=0 | NA | NA | 0.80, *P=*0.426 |  |
| **Successful reperfusion** | **All** | **22** | **1.267(1.095-1.465)** | **50.19, d.f.=21** | **58.2%, *P*=0.000** | **0.0426** | **3.19, *P=* 0.001** | **0.053** |
|  | Study type |  |  |  |  |  |  |  |
|  | Prospective | 7 | 1.206(1.027-1.415) | 15.46, d.f.=6 | 61.2%, *P*=0.017 | 0.0212 | 2.29, *P=* 0.022 |  |
|  | Retrospective | 15 | 1.341(1.031-1.744) | 29.71, d.f.=14 | 52.9%, *P*=0.008 | 0.1135 | 2.19, *P=* 0.029 |  |
|  | Location of occluded artery |  |  |  |  |  |  |  |
|  | Anterior and posterior circulation | 5 | 1.366(0.986-1.893) | 6.13, d.f.=4 | 34.8%, *P*=0.190 | 0.0474 | 1.87, *P=* 0.061 |  |
|  | Anterior circulation | 17 | 1.241(1.055-1.459) | 40.80, d.f.=16 | 60.8%, *P*=0.001 | 0.0413 | 2.61, *P=* 0.009 |  |
|  | Ethnicity |  |  |  |  |  |  |  |
|  | Caucasian | 17 | 1.263(1.092-1.461) | 35.31, d.f.=16 | 54.7%, *P=* 0.004 | 0.0314 | 3.14, *P=* 0.002 |  |
|  | Asian | 5 | 1.178(0.643-2.159) | 13.60, d.f.=4 | 70.6%, *P=* 0.009 | 0.2884 | 0.53, *P=* 0.596 |  |
| **Complete reperfusion** | **All** | **8** | **0.988(0.800-1.219)** | **15.68, d.f.=7** | **55.4%, *P=* 0.028** | **0.0445** | **0.12, *P=* 0.908** | **0.194** |
|  | Study type |  |  |  |  |  |  |  |
|  | Prospective | 6 | 0.962(0.761-1.217) | 11.52, d.f.=7 | 56.6%, *P=* 0.042 | 0.0450 | 0.32, *P=* 0.747 |  |
|  | Retrospective | 2 | 0.750(0.190-2.962) | 2.83, d.f.=1 | 64.6%, *P=* 0.093 | 0.7148 | 0.41, *P=* 0.682 |  |
|  | Location of occluded artery |  |  |  |  |  |  |  |
|  | Anterior and posterior circulation | 1 | 1.380(0.759-2.508) | 0.00,d.f.=0 | NA | 0.0000 | 1.06, *P*= 0.291 |  |
|  | Anterior circulation | 7 | 0.955(0.763,1.196) | 14.69, d.f.=6 | 59.2%, *P=* 0.023 | 0.0475 | 0.40, *P*= 0.690 |  |
| **Any bleeding** | **All** | **23(26)** | **1.215(1.040-1.420)** | **39.72, d.f.=25** | **37.1%, *P=* 0.031** | **0.0426** | **2.45, *P*= 0.014** | **0.825** |
|  | Study type |  |  |  |  |  |  |  |
|  | Prospective | 9(10) | 1.071(0.884-1.299) | 12.38, d.f.=9 | 27.3%, *P=* 0.193 | 0.0208 | 0.70, *P=* 0.482 |  |
|  | Retrospective | 14(16) | 1.354(1.088-1.685) | 21.66, d.f.=15 | 30.8%, *P=* 0.117 | 0.0541 | 2.72, *P=* 0.007 |  |
|  | Location of occluded artery |  |  |  |  |  |  |  |
|  | Anterior circulation | 17(20) | 1.251(1.043-1.502) | 31.73, d.f.=19 | 40.1%, *P=* 0.034 | 0.0483 | 2.41, *P=* 0.016 |  |
|  | Anterior and posterior circulation | 6 | 1.082(0.756-1.547) | 7.98, d.f.=5 | 37.4%, *P=* 0.157 | 0.0697 | 0.43, *P=* 0.668 |  |
|  | Ethnicity |  |  |  |  |  |  |  |
|  | Caucasian | 19(21) | 1.163(0.987-1.370) | 29.82, d.f.=20 | 32.9%, *P=* 0.073 | 0.0337 | 1.80, *P=*0.072 |  |
|  | Asian | 3(4) | 1.316(0.805-2.152) | 5.63, d.f.=3 | 46.7%, *P=*0.131 | 0.1143 | 1.09, *P=*0.274 |  |
|  | Caucasian and Asian | 1 | 2.085(1.096-3.968) | 0.00,d.f.=0 | NA | 0.0000 | 2.24, *P=*0.025 |  |
| **Any ICH** | **All** | **20(23)** | **1.214(1.040-1.417)** | **31.46, d.f.=22** | **30.1%, *P=*0.087** | **0.0303** | **2.46, *P=*0.014** | **0.805** |
|  | Study type |  |  |  |  |  |  |  |
|  | Prospective | 7 | 1.074(0.959-1.204) | 4.71, d.f.=6 | 0.0%, *P=*0.581 | 0.0000 | 1.23, *P=*0.218 |  |
|  | Retrospective | 13(16) | 1.390(1.119-1.725) | 19.76, d.f.=15 | 24.1%, *P=*0.181 | 0.0417 | 2.98, *P=*0.003 |  |
|  | Location of occluded artery |  |  |  |  |  |  |  |
|  | Anterior circulation | 15(3) | 1.224(1.028-1.457) | 24.15, d.f.=17 | 29.6%, *P=*0.115 | 0.0299 | 2.27, *P=*0.023 |  |
|  | Anterior and posterior circulation | 5 | 1.111(0.725-1.703) | 7.10, d.f.=4 | 43.6%, *P=*0.131 | 0.0953 | 0.49, *P=*0.628 |  |
|  | Ethnicity |  |  |  |  |  |  |  |
|  | Caucasian | 18(20) | 1.173(1.004-1.370) | 25.43, d.f.=19 | 25.3%, *P=*0.147 | 0.0230 | 2.02, *P=*0.044 |  |
|  | Asian | 2(3) | 1.532(0.886-2.651) | 3.33, d.f.=2 | 39.9%, *P=*0.189 | 0.0950 | 1.53, *P=*0.127 |  |
| **aICH** | **All** | **4** | **1.936(1.384-2.708)^#^** | **0.57, d.f.=3** | **0.0%, *P=*0.903** | **NA** | **3.86, *P=*0.000** | **0.155** |
|  | Location of occluded artery |  |  |  |  |  |  |  |
|  | Anterior circulation | 3 | 1.961(1.312-2.930) ^#^ | 0.56, d.f.=2 | 0.0%, *P=*0.756 | NA | 3.29, *P=*0.001 |  |
|  | Anterior and posterior circulation | 1 | 1.880(1.022-3.458) ^#^ | 0.00,d.f.=0 | NA | NA | 2.03, *P=*0.042 |  |
|  | Ethnicity |  |  |  |  |  |  |  |
|  | Caucasian | 3 | 1.832(1.166-2.880) ^#^ | 0.45, d.f.=2 | 0.0%, *P=*0.800 | NA | 2.63, *P=*0.009 |  |
|  | Asian | 1 | 2.071(1.256-3.414) ^#^ | 0.00,d.f.=0 | NA | NA | 2.85, *P=*0.004 |  |
| **sICH** | **All** | **16** | **1.204(1.021-1.421) ^#^** | **16.83, d.f.=15** | **10.9%, *P=*0.329** | **NA** | **2.20, *P=*0.028** | **0.140** |
|  | Study type |  |  |  |  |  |  |  |
|  | Prospective | 6 | 1.123(0.854-1.475) ^#^ | 5.11, d.f.=5 | 2.1%, *P=*0.403 | NA | 0.83, *P=*0.406 |  |
|  | Retrospective | 10 | 1.255(1.019-1.545) ^#^ | 11.32, d.f.=9 | 20.5%, *P=*0.254 | NA | 2.14, *P=*0.033 |  |
|  | Location of occluded artery |  |  |  |  |  |  |  |
|  | Anterior circulation | 13 | 1.206(0.988-1.473) ^#^ | 13.60, d.f.=12 | 11.8%, *P=*0.327 | NA | 1.84, *P=*0.066 |  |
|  | Anterior and posterior circulation | 3 | 1.201(0.894-1.613) ^#^ | 3.23, d.f.=2 | 38.0%, *P=*0.199 | NA | 1.21, *P=*0.225 |  |
|  | Ethnicity |  |  |  |  |  |  |  |
|  | Caucasian | 15 | 1.223(1.031-1.450) ^#^ | 16.31, d.f.=14 | 14.2%, *P=*0.295 | NA | 2.31, *P=*0.021 |  |
|  | Asian | 1 | 0.940(0.470-1.879) ^#^ | 0.00,d.f.=0 | NA | NA | 0.18, *P=*0.861 |  |
| **HT** | **All** | **8** | **1.355(1.014-1.811)** | **16.12,d.f.=7** | **56.6%, *P=*0.024** | **0.0838** | **2.05, *P=*0.040** | **0.117** |
|  | Study type |  |  |  |  |  |  |  |
|  | Prospective | 4 | 1.364(1.045-1.780) | 2.51, d.f.=3 | 0.0%, *P=*0.474 | 0.0000 | 2.28, *P=*0.022 |  |
|  | Retrospective | 4 | 1.376(0.811-2.336) | 12.86, d.f.=3 | 76.7%, *P=*0.005 | 0.1972 | 1.18, *P=*0.237 |  |
|  | Location of occluded artery |  |  |  |  |  |  |  |
|  | Anterior circulation | 6 | 1.434(1.038-1.981) | 14.67, d.f.=5 | 65.9%, *P=*0.012 | 0.0974 | 2.19, *P=*0.029 |  |
|  | Anterior and posterior circulation | 2 | 0.905(0.459-1.786) | 0.56, d.f.=1 | 0.0%, *P=*0.455 | 0.0000 | 0.29, *P=*0.774 |  |
|  | Ethnicity |  |  |  |  |  |  |  |
|  | Caucasian | 6 | 1.353(0.971-1.883) | 12.58,d.f.=5 | 60.3%, *P=*0.028 | 0.0855 | 1.79, *P=*0.074 |  |
|  | Caucasian and Asian | 1 | 2.085(1.096-3.968) | 0.00,d.f.=0 | NA | 0.0000 | 2.24, *P=*0.025 |  |
|  | Asian | 1 | 0.870(0.437-1.730) | 0.00,d.f.=0 | NA | 0.0000 | 0.40, *P=*0.691 |  |
| **PH** | **All** | **5(6)** | **1.525(0.884-2.632)** | **13.12,d.f.=5** | **61.9%, *P=*0.022** | **0.2300** | **1.52, *P=*0.129** | **0.343** |
|  | Study type |  |  |  |  |  |  |  |
|  | Prospective | 3 | 1.249(0.932-1.674) | 0.49,d.f.=2 | 0.0%, *P=*0.783 | 0.0000 | 1.49, *P=*0.136 |  |
|  | Retrospective | 2(3) | 2.198(0.449-10.759) | 12.17,d.f.=2 | 83.6%, *P=*0.002 | 1.6354 | 0.97, *P=*0.331 |  |
|  | Location of occluded artery |  |  |  |  |  |  |  |
|  | Anterior circulation | 4(5) | 1.510(0.858-2.659) | 12.78,d.f.=4, | 68.7%, *P=*0.012 | 0.2507 | 1.43, *P=*0.153 |  |
|  | Anterior and posterior circulation | 1 | 4.900(0.055-433.92) | 0.00,d.f.=0 | NA | 0.0000 | 0.69, *P=*0.487 |  |
| **PH-2** | **All** | **2** | **2.831(0.798-10.04) ^#^** | **0.06,d.f.=1** | **0.0%, *P=*0.803** | **NA** | **1.61, *P=*0.107** | **NA** |
| **Early recovery at 24 hours after admission** | **All** | **3** | **1.457(1.084-1.957) ^#^** | **1.77,d.f.=2** | **0.0%, *P=*0.413** | **NA** | **2.50, *P=*0.013** | **0.553** |
|  | Study type |  |  |  |  |  |  |  |
|  | Prospective | 2 | 1.280(0.689-2.377) | 1.76,d.f.=1 | 43.3%, *P=*0.184 | 0.1098 | 0.78, *P=*0.434 |  |
|  | Retrospective | 1 | 1.540(0.449-5.278) | 0.00, d.f.=0 | NA | 0.0000 | 0.69, *P=*0.492 |  |
|  | Ethnicity |  |  |  |  |  |  |  |
|  | Caucasian | 2 | 1.280(0.689-2.377) | 1.76,d.f.=1 | 43.3%, *P=*0.184 | 0.1098 | 0.78, *P=*0.434 |  |
|  | Asian | 1 | 1.540(0.449-5.278) | 0.00, d.f.=0 | NA | 0.0000 | 0.69, *P=*0.492 |  |
| **Number of passes of the thrombectomy device≤2** | **All** | **4** | **1.466(0.983-2.185)** | **19.73,d.f.=3** | **84.8%, *P=*0.000** | **0.1205** | **1.88, *P=*0.060** | **0.011** |
|  | Study type |  |  |  |  |  |  |  |
|  | Prospective | 2 | 1.122(0.930-1.353) | 1.52,d.f.=1 | 34.4%, *P=*0.217 | 0.0064 | 1.20, *P=*0.230 |  |
|  | Retrospective | 2 | 4.061(0.380-43.366) | 15.02,d.f.=1 | 93.3%, *P=*0.000 | 2.7348 | 1.16, *P=*0.246 |  |
|  | Location of occluded artery |  |  |  |  |  |  |  |
|  | Anterior circulation | 3 | 1.156(0.998-1.339) | 2.35,d.f.=2 | 14.9%, *P=*0.309 | 0.0026 | 1.94, *P=*0.053 |  |
|  | Anterior and posterior circulation | 1 | 14.63(4.460-47.995) | 0.00,d.f.=0 | NA | 0.0000 | 4.43, *P=*0.000 |  |
| **Any procedural complications** | **All** | **9(16)** | **1.031(0.839-1.267)** | **26.59,d.f.=15** | **43.6%, *P=*0.032** | **0.0628** | **0.29, *P=*0.774** | **0.002** |
|  | Study type |  |  |  |  |  |  |  |
|  | Prospective | 3(9) | 0.911(0.783-1.059) | 7.72,d.f.=8 | 0.0%, *P=*0.461 | 0.0000 | 1.21, *P=*0.226 |  |
|  | Retrospective | 6(7) | 1.631(0.886-3.002) | 16.56,d.f.=6 | 63.8%, *P=*0.011 | 0.3715 | 1.57, *P=*0.116 |  |
|  | Location of occluded artery |  |  |  |  |  |  |  |
|  | Anterior circulation | 6(12) | 0.974(0.804-1.180) | 17.43,d.f.=11 | 36.9%, *P=*0.096 | 0.0375 | 0.27, *P=*0.787 |  |
|  | Anterior and posterior circulation | 3(4) | 1.911(0.687-5.317) | 7.58,d.f.=3 | 60.4%, *P=*0.055 | 0.6021 | 1.24, *P=*0.215 |  |
| **Clot migration** | **All** | **8** | **1.393(0.874-2.219)** | **21.76,d.f.=7** | **67.8%, *P=*0.003** | **0.2263** | **1.39, *P=*0.164** | **0.016** |
|  | Study type |  |  |  |  |  |  |  |
|  | Prospective | 3 | 0.983(0.626-1.542) | 4.12,d.f.=2 | 51.5%, *P=*0.127 | 0.0761 | 0.08, *P=*0.939 |  |
|  | Retrospective | 5 | 2.434(0.905-6.547) | 16.43,d.f.=4 | 75.7%, *P=*0.002 | 0.8321 | 1.76, *P=*0.078 |  |
|  | Location of occluded artery |  |  |  |  |  |  |  |
|  | Anterior circulation | 5 | 1.246(0.772-2.010) | 13.69,d.f.=4 | 70.8%, *P=*0.008 | 0.1729 | 0.90, *P=*0.368 |  |
|  | Anterior and posterior circulation | 3 | 2.904(0.468-17.999) | 7.57,d.f.=2 | 73.6%, *P=*0.023 | 1.8491 | 1.15, *P=*0.252 |  |
| **Rescue therapy** | **All** | **2** | **0.811(0.638-1.030) ^#^** | **0.45,d.f.=1** | **0.0%,*P=*0.500** | **NA** | **1.72, *P=*0.086** |  |
| **Vasospasm** | **All** | **2** | **0.983(0.517-1.870) ^#^** | **1.34,d.f.=1** | **25.1%,*P=*0.248** | **NA** | **0.05, *P=*0.959** |  |

Abbreviations: NA, not available or not applicable; d.f., degrees of freedom; aOR, adjusted odds ratio; aRR, adjusted risk ratio; ICH, intracranial hemorrhage; sICH, symptomatic intracranial hemorrhage; aICH, asymptomatic intracranial hemorrhage; HT, hemorrhagic transformation; PH, parenchymal hematoma.

^*^ If one study included many subtypes of the outcomes or many subgroups of the patients, and the subtypes or subgroups were pooled and analyzed into one outcome, we calculated the number of the study and the subtype or subgroup respectively.

^#^ The value was calculated with fixed effects analysis.

**Supplemental Table 8.** The quality assessment with Newcastle-Ottawa Scale of enrolled studies in the meta-analysis

| Study | Selection | | | | Comparability | Outcome | | | Overall |
| --- | --- | --- | --- | --- | --- | --- | --- | --- | --- |
|  | Representativeness of the exposed cohort | Selection of the non-exposed cohort | Ascertainment of exposure | Outcome not present at start |  | Assessment of outcome | Follow-up  Long enough for outcomes | Adequacy of follow-up |  |
| Yang et al.2020[[1](#_ENREF_1)] | ☆ | ☆ | ☆ | ☆ | ☆☆ | ☆ | ☆ | ☆ | 9 |
| Rajah et al.2020[[2](#_ENREF_2)] | ☆ | ☆ | ☆ |  | ☆☆ |  | ☆ | ☆ | 7 |
| Mohammaden et al.2020[[3](#_ENREF_3)] | ☆ | ☆ | ☆ |  | ☆☆ | ☆ | ☆ | ☆ | 8 |
| Hassan et al.2020[[4](#_ENREF_4)] | ☆ | ☆ | ☆ |  | ☆☆ | ☆ | ☆ | ☆ | 8 |
| Uchikawa et al. 2019[[5](#_ENREF_5)] | ☆ | ☆ | ☆ |  |  | ☆ | ☆ | ☆ | 6 |
| Wareham et al. 2019[[6](#_ENREF_6)] | ☆ | ☆ | ☆ | ☆ |  | ☆ | ☆ | ☆ | 7 |
| Pikija et al. 2019[[7](#_ENREF_7)] | ☆ | ☆ | ☆ |  | ☆☆ |  | ☆ | ☆ | 7 |
| Tajima et al. 2019[[8](#_ENREF_8)] | ☆ | ☆ | ☆ |  | ☆ |  | ☆ | ☆ | 6 |
| Gong et al. 2019[[9](#_ENREF_9)] | ☆ | ☆ | ☆ |  | ☆☆ |  | ☆ | ☆ | 7 |
| Lee et al.2019[[10](#_ENREF_10)] | ☆ | ☆ | ☆ | ☆ | ☆☆ |  | ☆ | ☆ | 8 |
| Kaesmacher et al. 2019[[11](#_ENREF_11)] | ☆ | ☆ | ☆ |  |  | ☆ | ☆ | ☆ | 6 |
| Balodis et al. 2019[[12](#_ENREF_12)] | ☆ | ☆ | ☆ | ☆ | ☆ |  | ☆ | ☆ | 7 |
| Meyer et al. 2019[[13](#_ENREF_13)] | ☆ | ☆ | ☆ | ☆ | ☆☆ |  | ☆ | ☆ | 8 |
| Hassan et al. 2019[[14](#_ENREF_14)] | ☆ | ☆ | ☆ |  | ☆☆ |  | ☆ | ☆ | 7 |
| Goyal N.et al. 2019[[15](#_ENREF_15)] | ☆ | ☆ | ☆ |  | ☆☆ | ☆ | ☆ | ☆ | 8 |
| Casetta et al. 2019[[16](#_ENREF_16)] | ☆ | ☆ | ☆ |  | ☆☆ |  | ☆ | ☆ | 7 |
| Chalos et al. 2019[[17](#_ENREF_17)] | ☆ | ☆ | ☆ | ☆ | ☆☆ |  | ☆ | ☆ | 8 |
| Gamba et al. 2019[[18](#_ENREF_18)] | ☆ | ☆ | ☆ |  | ☆☆ |  | ☆ | ☆ | 7 |
| Aoki et al. 2019[[19](#_ENREF_19)] | ☆ | ☆ | ☆ | ☆ | ☆☆ | ☆ | ☆ | ☆ | 9 |
| Goyal N.et al. 2019[[20](#_ENREF_20)] | ☆ | ☆ | ☆ |  | ☆☆ | ☆ | ☆ | ☆ | 8 |
| Raychev et al. 2019[[21](#_ENREF_21)] | ☆ | ☆ | ☆ |  | ☆☆ | ☆ | ☆ | ☆ | 8 |
| Anadani et al. 2019[[22](#_ENREF_22)] | ☆ | ☆ | ☆ |  | ☆☆ |  | ☆ | ☆ | 7 |
| Qureshi et al. 2019[[23](#_ENREF_23)] | ☆ | ☆ | ☆ | ☆ | ☆☆ | ☆ | ☆ | ☆ | 9 |
| Wollenweber et al. 2019[[24](#_ENREF_24)] | ☆ | ☆ | ☆ | ☆ | ☆☆ | ☆ | ☆ | ☆ | 9 |
| Panni et al. 2019[[25](#_ENREF_25)] | ☆ | ☆ | ☆ |  |  | ☆ | ☆ | ☆ | 6 |
| Tran et al.2019[[26](#_ENREF_26)] | ☆ | ☆ | ☆ |  |  | ☆ | ☆ | ☆ | 6 |
| Candel et al. 2019[[27](#_ENREF_27)] | ☆ | ☆ | ☆ |  | ☆ |  | ☆ | ☆ | 6 |
| Bücke et al. 2018[[28](#_ENREF_28)] | ☆ | ☆ | ☆ | ☆ | ☆☆ |  | ☆ | ☆ | 8 |
| Ren et al. 2018[[29](#_ENREF_29)] | ☆ | ☆ | ☆ |  | ☆ | ☆ | ☆ | ☆ | 7 |
| Anadani et al. 2018[[30](#_ENREF_30)] | ☆ | ☆ | ☆ | ☆ | ☆☆ | ☆ | ☆ | ☆ | 9 |
| Rocha et al. 2018[[31](#_ENREF_31)] | ☆ | ☆ | ☆ | ☆ | ☆☆ | ☆ | ☆ | ☆ | 9 |
| Leker et al. 2018[[32](#_ENREF_32)] | ☆ | ☆ | ☆ | ☆ | ☆☆ |  | ☆ | ☆ | 8 |
| Gariel et al. 2018[[33](#_ENREF_33)] | ☆ | ☆ | ☆ | ☆ | ☆☆ |  | ☆ | ☆ | 8 |
| Sallustio et al. 2018[[34](#_ENREF_34)] | ☆ | ☆ | ☆ |  | ☆☆ |  | ☆ | ☆ | 7 |
| DiMaria et al. 2018[[35](#_ENREF_35)] | ☆ | ☆ | ☆ | ☆ | ☆☆ | ☆ | ☆ | ☆ | 9 |
| Díaz-Pérez et al. 2018[[36](#_ENREF_36)] | ☆ | ☆ | ☆ |  | ☆☆ |  | ☆ | ☆ | 7 |
| Maingard et al. 2018[[37](#_ENREF_37)] | ☆ | ☆ | ☆ |  | ☆☆ |  | ☆ | ☆ | 7 |
| Al-Khaled et al. 2018[[38](#_ENREF_38)] | ☆ | ☆ | ☆ | ☆ | ☆☆ | ☆ | ☆ | ☆ | 9 |
| Ferrigno et al. 2018[[39](#_ENREF_39)] | ☆ | ☆ | ☆ | ☆ | ☆☆ |  | ☆ | ☆ | 8 |
| Imbarrato et al. 2018[[40](#_ENREF_40)] | ☆ | ☆ | ☆ |  | ☆ |  | ☆ | ☆ | 6 |
| Li et al. 2018[[41](#_ENREF_41)] | ☆ | ☆ | ☆ |  | ☆☆ | ☆ | ☆ | ☆ | 8 |
| Wang et al. 2018[[42](#_ENREF_42)] | ☆ | ☆ | ☆ |  | ☆☆ | ☆ | ☆ | ☆ | 8 |
| Gory et al. 2018[[43](#_ENREF_43)] | ☆ | ☆ | ☆ |  | ☆☆ | ☆ | ☆ | ☆ | 8 |
| Goyal N. et al. 2018[[44](#_ENREF_44)] | ☆ | ☆ | ☆ |  | ☆☆ |  | ☆ | ☆ | 7 |
| Barral et al. 2018[[45](#_ENREF_45)] | ☆ | ☆ | ☆ |  | ☆☆ |  | ☆ | ☆ | 7 |
| Choi et al. 2018[[46](#_ENREF_46)] | ☆ | ☆ | ☆ |  | ☆☆ |  | ☆ | ☆ | 7 |
| Gory B. et al. 2018[[47](#_ENREF_47)] | ☆ | ☆ | ☆ |  | ☆☆ | ☆ | ☆ | ☆ | 8 |
| Bourcier et al. 2018[[48](#_ENREF_48)] | ☆ | ☆ | ☆ |  | ☆☆ |  | ☆ | ☆ | 7 |
| Chu et al. 2018[[49](#_ENREF_49)] | ☆ | ☆ | ☆ |  | ☆☆ |  | ☆ | ☆ | 7 |
| Manceau et al. 2018[[50](#_ENREF_50)] | ☆ | ☆ | ☆ |  | ☆☆ |  | ☆ | ☆ | 7 |
| Kaesmacher et al. 2018[[51](#_ENREF_51)] | ☆ | ☆ | ☆ |  | ☆ | ☆ | ☆ | ☆ | 7 |
| Rai et al. 2018[[52](#_ENREF_52)] | ☆ | ☆ | ☆ |  | ☆ |  | ☆ | ☆ | 6 |
| Coutinho et al. 2017[[53](#_ENREF_53)] | ☆ | ☆ | ☆ | ☆ | ☆☆ | ☆ | ☆ | ☆ | 9 |
| Bellwald et al. 2017[[54](#_ENREF_54)] | ☆ | ☆ | ☆ |  | ☆☆ | ☆ | ☆ | ☆ | 8 |
| Kim et al. 2017[[55](#_ENREF_55)] | ☆ | ☆ | ☆ |  | ☆☆ | ☆ | ☆ | ☆ | 8 |
| Uno et al. 2017[[56](#_ENREF_56)] | ☆ | ☆ | ☆ | ☆ | ☆☆ |  | ☆ | ☆ | 8 |
| Merlino et al. 2017[[57](#_ENREF_57)] | ☆ | ☆ | ☆ | ☆ | ☆☆ | ☆ | ☆ | ☆ | 9 |
| Wang et al. 2017[[58](#_ENREF_58)] | ☆ | ☆ | ☆ |  | ☆☆ | ☆ | ☆ | ☆ | 8 |
| Park et al. 2017[[59](#_ENREF_59)] | ☆ | ☆ | ☆ |  | ☆☆ | ☆ | ☆ | ☆ | 8 |
| Wee et al. 2017[[60](#_ENREF_60)] | ☆ | ☆ | ☆ |  | ☆ | ☆ | ☆ | ☆ | 7 |
| Abilleira et al. 2017[[61](#_ENREF_61)] | ☆ | ☆ | ☆ | ☆ | ☆☆ |  | ☆ | ☆ | 8 |
| Maier et al. 2017[[62](#_ENREF_62)] | ☆ | ☆ | ☆ | ☆ | ☆ | ☆ | ☆ | ☆ | 8 |
| Mundiyanapurath et al. 2017[[63](#_ENREF_63)] | ☆ | ☆ | ☆ |  | ☆☆ | ☆ | ☆ |  | 7 |
| Leciñana et al. 2017[[64](#_ENREF_64)] | ☆ | ☆ | ☆ | ☆ | ☆☆ |  | ☆ | ☆ | 8 |
| Weber et al. 2017[[65](#_ENREF_65)] | ☆ | ☆ | ☆ | ☆ | ☆☆ |  | ☆ | ☆ | 8 |
| Mistry et al.2017[[66](#_ENREF_66)] | ☆ | ☆ | ☆ |  | ☆☆ | ☆ | ☆ | ☆ | 8 |
| Nogueira et al. 2016[[67](#_ENREF_67)] | ☆ | ☆ | ☆ |  | ☆☆ | ☆ | ☆ | ☆ | 8 |
| Angermaier et al. 2016[[68](#_ENREF_68)] | ☆ | ☆ | ☆ |  | ☆☆ | ☆ | ☆ |  | 7 |
| Rebello et al. 2016[[69](#_ENREF_69)] | ☆ | ☆ | ☆ |  | ☆☆ | ☆ | ☆ | ☆ | 8 |
| Broeg-Morvay et al. 2016[[70](#_ENREF_70)] | ☆ | ☆ | ☆ |  | ☆☆ | ☆ | ☆ | ☆ | 8 |
| Behme et al. 2016[[71](#_ENREF_71)] | ☆ | ☆ | ☆ |  | ☆☆ | ☆ | ☆ | ☆ | 8 |
| Villwock.et al. 2016[[72](#_ENREF_72)] | ☆ | ☆ | ☆ |  | ☆☆ | ☆ | ☆ | ☆ | 8 |
| Minnerup et al. 2016[[73](#_ENREF_73)] | ☆ | ☆ | ☆ | ☆ | ☆ |  | ☆ | ☆ | 7 |
| Mulder et al.2016[[74](#_ENREF_74)] | ☆ | ☆ | ☆ | ☆ | ☆ | ☆ | ☆ | ☆ | 8 |
| Wang et al. 2015[[75](#_ENREF_75)] | ☆ | ☆ | ☆ |  | ☆☆ | ☆ | ☆ |  | 7 |
| Bourcier et al. 2015[[76](#_ENREF_76)] | ☆ | ☆ | ☆ |  | ☆☆ |  | ☆ | ☆ | 7 |
| Saposnik et al. 2015[[77](#_ENREF_77)] | ☆ | ☆ | ☆ |  |  | ☆ | ☆ | ☆ | 6 |
| Goyal M.et al. 2015[[78](#_ENREF_78)] | ☆ | ☆ | ☆ | ☆ |  |  | ☆ | ☆ | 6 |
| Jeromel et al. 2015[[79](#_ENREF_79)] | ☆ | ☆ | ☆ |  | ☆☆ | ☆ | ☆ | ☆ | 8 |
| Guedin et al. 2015[[80](#_ENREF_80)] | ☆ | ☆ | ☆ |  | ☆ | ☆ | ☆ | ☆ | 7 |
| Fujimoto et al. 2015[[81](#_ENREF_81)] | ☆ | ☆ | ☆ |  | ☆☆ | ☆ | ☆ | ☆ | 8 |
| Nogueira et al. 2015[[82](#_ENREF_82)] | ☆ | ☆ | ☆ |  | ☆☆ | ☆ | ☆ | ☆ | 8 |
| Jankowitz et al. 2015[[83](#_ENREF_83)] | ☆ | ☆ | ☆ |  |  | ☆ | ☆ | ☆ | 6 |
| Jovin et al. 2015[[84](#_ENREF_84)] | ☆ | ☆ | ☆ | ☆ | ☆ | ☆ | ☆ | ☆ | 8 |
| Leker et al. 2015[[85](#_ENREF_85)] | ☆ | ☆ | ☆ | ☆ | ☆☆ |  | ☆ | ☆ | 8 |
| Brinjikji et al. 2014[[86](#_ENREF_86)] | ☆ | ☆ | ☆ |  | ☆☆ | ☆ | ☆ | ☆ | 8 |
| Spiotta.et al.2014[[87](#_ENREF_87)] | ☆ | ☆ | ☆ |  | ☆☆ |  | ☆ | ☆ | 7 |
| Mourand et al. 2014[[88](#_ENREF_88)] | ☆ | ☆ | ☆ | ☆ |  |  | ☆ | ☆ | 6 |
| Kass-Hout et al. 2014[[89](#_ENREF_89)] | ☆ | ☆ | ☆ |  | ☆ |  | ☆ | ☆ | 6 |
| Jankowitz et al. 2012[[90](#_ENREF_90)] | ☆ | ☆ | ☆ |  | ☆☆ |  | ☆ | ☆ | 7 |
| Da´valos et al. 2012[[91](#_ENREF_91)] | ☆ | ☆ | ☆ |  | ☆ |  | ☆ | ☆ | 6 |
| Pfefferkorn et al. 2012[[92](#_ENREF_92)] | ☆ | ☆ | ☆ |  | ☆ |  | ☆ | ☆ | 6 |
| Bang et al. 2011[[93](#_ENREF_93)] | ☆ | ☆ | ☆ | ☆ | ☆☆ | ☆ | ☆ | ☆ | 9 |

**Supplemental Table 9**. Adjustment of publication bias with “Trim and fill” method in the analysis of outcomes

| **Outcome** | **No. of studies**  **(subgroups/ subtypes)^*^** | **Methods of analysis** | **Egger's test** | **“Trim and fill” adjustment method** | | | | |
| --- | --- | --- | --- | --- | --- | --- | --- | --- |
|  |  |  | ***P* value** | **Effects model** | **Unadjusted OR (95%CI)** | ***P* value** | **Adjusted OR(95%CI)** | ***P* value** |
| **aICH** | 7 | Unadjusted | 0.088 | Fixed | 1.524(1.233-1.882) | 0.000 | 1.472(1.203-1.802) | 0.000 |
|  |  |  |  | Random | 1.538(1.236-1.915) | 0.000 | 1.472(1.203-1.802) | 0.000 |
| **Passes number of the thrombectomy device≤2** | 10 | Unadjusted | 0.024 | Fixed | 1.429(1.243-1.643) | 0.000 | 1.303(1.139-1.489) | 0.000 |
|  |  |  |  | Random | 1.870(1.344-2.603) | 0.000 | 1.361(0.951-1.946) | 0.092 |
|  | 4 | Adjusted | 0.011 | Fixed | 1.188(1.040-1.357) | 0.011 | 1.188(1.040-1.357) | 0.011 |
|  |  |  |  | Random | 1.466(0.983-2.185) | 0.060 | 1.466(0.983-2.185) | 0.060 |
| **Vessel dissection** | 3 | Unadjusted | 0.068 | Fixed | 1.246(0.761-2.039) | 0.382 | 1.246(0.761-2.039) | 0.382 |
|  |  |  |  | Random | 1.178(0.635-2.183) | 0.604 | 1.178(0.635-2.183) | 0.604 |
| **Good outcome at 90 days** | 35 | Adjusted | 0.018 | Fixed | 1.325(1.215-1.446) | 0.000 | 1.283(1.178-1.398) | 0.000 |
|  |  |  |  | Random | 1.369(1.217-1.540) | 0.000 | 1.299(1.140-1.479) | 0.000 |
| **Successful reperfusion** | 22 | Adjusted | 0.053 | Fixed | 1.140(1.077-1.207) | 0.000 | 1.138(1.075-1.205) | 0.000 |
|  |  |  |  | Random | 1.267(1.095-1.465) | 0.001 | 1.250(1.076-1.452) | 0.004 |
| **Any complications** | 9(16) | Adjusted | 0.002 | Fixed | 0.960(0.839-1.099) | 0.555 | 0.904(0.792-1.031) | 0.132 |
|  |  |  |  | Random | 1.031(0.839-1.267) | 0.774 | 0.908(0.707-1.168) | 0.453 |
| **Clot migration** | 8 | Adjusted | 0.016 | Fixed | 1.038(0.846-1.272) | 0.722 | 0.988(0.808-1.209) | 0.910 |
|  |  |  |  | Random | 1.393(0.874-2.219) | 0.164 | 1.160(0.694-1.938) | 0.572 |

Abbreviations: aICH, asymptomatic intracranial hemorrhage; OR, odds ratio; 95% CI, 95% confidence interval.

^*^ If one study included many subtypes of the outcomes or many subgroups of the patients, and the subtypes or subgroups were pooled and analyzed into one outcome, we calculated the number of the study and the subtype or subgroup respectively.

**Supplemental Table 10.** Overview of the pooled RCTs results

| **Outcome** | **Unadjusted /adjusted Analyses** | **Analyses** | | | | | | |
| --- | --- | --- | --- | --- | --- | --- | --- | --- |
|  |  | **No. of studies (subgroups/ subtypes)** | **cOR/cRR/aOR/aRR**  **(95%CI)** | **Heterogeneity** | | | **Overall effect** | **Egger's test** |
|  |  |  |  | **Chi^2^, d.f.** | **I^2^, *P* for Cochran Q** | **Tau^2^** | **Z value, *P* value** | ***P* value** |
| **Excellent outcome at 90 days** | Unadjusted analyses | 4 | 1.035(0.803-1.334)^#^ | 3.80,d.f.=3 | 21.0%, *P*=0.284 | NA | 0.26, *P*=0.791 | 0.899 |
|  | Adjusted analyses | 2 | 1.015(0.781-1.319) ^#^ | 0.51, d.f.=1 | 0.0%, *P*=0.475 | NA | 0.11, *P*=0.912 | NA |
| **Good outcome at 90 days** | Unadjusted analyses | 5 | 1.293(0.940-1.779) | 10.25, d.f.=4 | 61.0%, *P*=0.036 | 0.0736 | 1.58, *P*=0.115 | 0.541 |
|  | Adjusted analyses | 3 | 1.201(0.987-1.461) ^#^ | 1.09, d.f.=2 | 0.0%, *P*=0.581 | NA | 1.83, *P*=0.068 | 0.957 |
| **Mortality within 90 days** | Unadjusted analyses | 4 | 0.567(0.349-0.921) | 12.99, d.f.=3 | 76.9%, *P*=0.005 | 0.1719 | 2.29, *P*=0.022 | 0.832 |
|  | Adjusted analyses | 2 | 0.584(0.446-0.765) ^#^ | 0.00, d.f.=1 | 0.0%, *P*=0.951 | NA | 3.91, *P*=0.000 | NA |
| **Successful reperfusion** | Unadjusted analyses | 4 | 1.228(1.011-1.492) ^#^ | 2.63, d.f.=3 | 0.0%, *P*=0.452 | NA | 2.07, *P*=0.038 | 0.732 |
|  | Adjusted analyses | 3 | 1.058(0.988-1.133) ^#^ | 1.94, d.f.=2 | 0.0%, *P*=0.378 | NA | 1.62, *P*=0.105 | 0.482 |
| **sICH** | Unadjusted analyses | 3 | 1.281(0.864-1.899) ^#^ | 0.36, d.f.=2 | 0.0%, *P*=0.834 | NA | 1.23, *P*=0.217 | 0.373 |
|  | Adjusted analyses | 2 | 1.323(0.871-2.010) ^#^ | 0.17, d.f.=1 | 0.0%, *P*=0.683 | NA | 1.31, *P*=0.189 | NA |

Abbreviations: RCTs, Randomized Controlled Trials; NA, not available or not applicable; d.f., degrees of freedom; aOR, adjusted odds ratio; aRR, adjusted risk ratio; cOR, crude odds ratio; cRR, crude risk ratio; sICH, symptomatic intracranial hemorrhage.

^#^ The value was calculated with fixed effects analysis.

**Reference**

[1]. Yang P, Zhang Y, Zhang L*, et al.* Endovascular Thrombectomy with or without Intravenous Alteplase in Acute Stroke. *The New England journal of medicine* 2020; **382:** 1981-1993.

[2]. Rajah G, Saber H, Lieber B*, et al.* A Moving Target? The Fate of Large Vessel Occlusion Strokes Pretreated with Intravenous Tissue Plasminogen Activator in the Era of Mechanical Thrombectomy. *World neurosurgery* 2020; **141:** e447-e452.

[3]. Mohammaden MH, Stapleton CJ, Brunozzi D*, et al.* Risk Factors for Distal Clot Migration during Mechanical Thrombectomy of Anterior Circulation Large Vessel Occlusion. *Cerebrovascular diseases (Basel, Switzerland)* 2020; **49:** 185-191.

[4]. Hassan AE, Ringheanu VM, Preston L, Tekle W, Qureshi AI. IV tPA is associated with increase in rates of intracerebral hemorrhage and length of stay in patients with acute stroke treated with endovascular treatment within 4.5 hours: should we bypass IV tPA in large vessel occlusion? *Journal of neurointerventional surgery* 2021; **13:** 114-118.

[5]. Uchikawa H, Kuroiwa T, Nishio A*, et al.* Vasospasm as a major complication after acute mechanical thrombectomy with stent retrievers. *Journal of clinical neuroscience : official journal of the Neurosurgical Society of Australasia* 2019; **64:** 163-168.

[6]. Wareham J, Goswami A, Renowden S*, et al.* Factors impacting on technical success in stroke thrombectomy: experience of a UK neuro-interventional unit. *Clinical radiology* 2019; **74:** 390-398.

[7]. Pikija S, Magdic J, Sztriha LK*, et al.* Endovascular Therapy for Tandem Occlusion in Acute Ischemic Stroke: Intravenous Thrombolysis Improves Outcomes. *Journal of clinical medicine* 2019; **8**.

[8]. Tajima Y, Hayasaka M, Ebihara K*, et al.* Effectiveness of Low-Dose Intravenous Tissue Plasminogen Activator before Stent Retriever or Aspiration Mechanical Thrombectomy. *Journal of vascular and interventional radiology : JVIR* 2019; **30:** 134-140.

[9]. Gong L, Zheng X, Feng L*, et al.* Bridging Therapy Versus Direct Mechanical Thrombectomy in Patients with Acute Ischemic Stroke due to Middle Cerebral Artery Occlusion: A Clinical- Histological Analysis of Retrieved Thrombi. *Cell transplantation* 2019; **28:** 684-690.

[10]. Lee S-H, Kim BJ, Han M-K*, et al.* Futile reperfusion and predicted therapeutic benefits after successful endovascular treatment according to initial stroke severity. *BMC neurology* 2019; **19:** 11.

[11]. Kaesmacher J, Chaloulos-Iakovidis P, Panos L*, et al.* Clinical effect of successful reperfusion in patients presenting with NIHSS < 8: data from the BEYOND-SWIFT registry. *Journal of neurology* 2019; **266:** 598-608.

[12]. Balodis A, Radzina M, Miglane E*, et al.* Endovascular thrombectomy in anterior circulation stroke and clinical value of bridging with intravenous thrombolysis. *Acta radiologica (Stockholm, Sweden : 1987)* 2019; **60:** 308-314.

[13]. Meyer L, Alexandrou M, Leischner H*, et al.* Mechanical thrombectomy in nonagenarians with acute ischemic stroke. *Journal of neurointerventional surgery* 2019; **11:** 1091-1094.

[14]. Hassan AE, Kotta H, Garza L*, et al.* Pre-thrombectomy intravenous thrombolytics are associated with increased hospital bills without improved outcomes compared with mechanical thrombectomy alone. *Journal of neurointerventional surgery* 2019; **11:** 1187-1190.

[15]. Goyal N, Tsivgoulis G, Pandhi A*, et al.* Impact of pretreatment with intravenous thrombolysis on reperfusion status in acute strokes treated with mechanical thrombectomy. *Journal of neurointerventional surgery* 2019; **11:** 1073-1079.

[16]. Casetta I, Pracucci G, Saletti A*, et al.* Combined intravenous and endovascular treatment versus primary mechanical thrombectomy. The Italian Registry of Endovascular Treatment in Acute Stroke. *International journal of stroke : official journal of the International Stroke Society* 2019; **14:** 898-907.

[17]. Chalos V, LeCouffe NE, Uyttenboogaart M*, et al.* Endovascular Treatment With or Without Prior Intravenous Alteplase for Acute Ischemic Stroke. *Journal of the American Heart Association* 2019; **8:** e011592.

[18]. Gamba M, Gilberti N, Premi E*, et al.* Intravenous fibrinolysis plus endovascular thrombectomy versus direct endovascular thrombectomy for anterior circulation acute ischemic stroke: clinical and infarct volume results. *BMC neurology* 2019; **19:** 103.

[19]. Aoki J, Suzuki K, Kanamaru T*, et al.* Association between initial NIHSS score and recanalization rate after endovascular thrombectomy. *Journal of the neurological sciences* 2019; **403:** 127-132.

[20]. Goyal N, Tsivgoulis G, Chang JJ*, et al.* Intravenous thrombolysis pretreatment and other predictors of infarct in a new previously unaffected territory (INT) in ELVO strokes treated with mechanical thrombectomy. *Journal of neurointerventional surgery* 2020; **12:** 142-147.

[21]. Raychev R, Saver JL, Jahan R*, et al.* The impact of general anesthesia, baseline ASPECTS, time to treatment, and IV tPA on intracranial hemorrhage after neurothrombectomy: pooled analysis of the SWIFT PRIME, SWIFT, and STAR trials. *Journal of neurointerventional surgery* 2020; **12:** 2-6.

[22]. Anadani M, Spiotta AM, Alawieh A*, et al.* Emergent Carotid Stenting Plus Thrombectomy After Thrombolysis in Tandem Strokes: Analysis of the TITAN Registry. *Stroke* 2019; **50:** 2250–2252.

[23]. Qureshi AI, Asif A, Aytac E*, et al.* Preprocedure Intravenous Recombinant Tissue Plasminogen Activator and Risk of Distal Embolization with Thrombectomy in Acute Ischemic Stroke. *Journal of stroke and cerebrovascular diseases : the official journal of National Stroke Association* 2019; **28:** 104362.

[24]. Wollenweber FA, Tiedt S, Alegiani A*, et al.* Functional Outcome Following Stroke Thrombectomy in Clinical Practice. *Stroke* 2019; **50:** 2500-2506.

[25]. Panni P, Gory B, Xie Y*, et al.* Acute Stroke With Large Ischemic Core Treated by Thrombectomy. *Stroke* 2019; **50:** 1164-1171.

[26]. Tran AT, Nguyen HA, Vu DL*, et al.* Basilar artery thrombectomy: assessment of outcome and identification of prognostic factors. *Acta neurologica Belgica* 2020; **120**.

[27]. Serna Candel C, Aguilar Pérez M, Hellstern V, AlMatter M, Bäzner H, Henkes H. Recanalization of Emergent Large Intracranial Vessel Occlusion through Intravenous Thrombolysis: Frequency, Clinical Outcome, and Reperfusion Pattern. *Cerebrovascular diseases (Basel, Switzerland)* 2019; **48:** 115-123.

[28]. Bücke P, Aguilar Pérez M, AlMatter M, Hellstern V, Bäzner H, Henkes H. Functional Outcome and Safety of Intracranial Thrombectomy After Emergent Extracranial Stenting in Acute Ischemic Stroke Due to Tandem Occlusions. *Frontiers in neurology* 2018; **9:** 940.

[29]. Ren Y, Churilov L, Mitchell P, Dowling R, Bush S, Yan B. Clot Migration Is Associated With Intravenous Thrombolysis in the Setting of Acute Ischemic Stroke. *Stroke* 2018; **49:** 3060-3062.

[30]. Anadani M, Ajinkya S, Alawieh A*, et al.* Intra-Arterial Tissue Plasminogen Activator Is a Safe Rescue Therapy with Mechanical Thrombectomy. *World neurosurgery* 2019; **123:** e604-e608.

[31]. Guimarães Rocha M, Carvalho A, Rodrigues M*, et al.* Primary Thrombectomy Versus Combined Mechanical Thrombectomy and Intravenous Thrombolysis in Large Vessel Occlusion Acute Ischemic Stroke. *Journal of stroke and cerebrovascular diseases : the official journal of National Stroke Association* 2019; **28:** 627-631.

[32]. Leker RR, Cohen JE, Tanne D*, et al.* Direct Thrombectomy versus Bridging for Patients with Emergent Large-Vessel Occlusions. *Interventional neurology* 2018; **7:** 403-412.

[33]. Gariel F, Lapergue B, Bourcier R*, et al.* Mechanical Thrombectomy Outcomes With or Without Intravenous Thrombolysis. *Stroke* 2018; **49:** 2383-2390.

[34]. Sallustio F, Koch G, Alemseged F*, et al.* Effect of mechanical thrombectomy alone or in combination with intravenous thrombolysis for acute ischemic stroke. *Journal of neurology* 2018; **265:** 2875-2880.

[35]. Di Maria F, Mazighi M, Kyheng M*, et al.* Intravenous Thrombolysis Prior to Mechanical Thrombectomy in Acute Ischemic Stroke: Silver Bullet or Useless Bystander? *Journal of stroke* 2018; **20:** 385-393.

[36]. Díaz-Pérez J, Parrilla G, Espinosa de Rueda M*, et al.* Mechanical Thrombectomy in Acute Stroke Due to Carotid Occlusion: A Series of 153 Consecutive Patients. *Cerebrovascular diseases (Basel, Switzerland)* 2018; **46:** 132-141.

[37]. Maingard J, Shvarts Y, Motyer R*, et al.* Outcomes of endovascular thrombectomy with and without bridging thrombolysis for acute large vessel occlusion ischaemic stroke. *Internal medicine journal* 2019; **49:** 345-351.

[38]. Al-Khaled M, Brüning T, Gottwald C, Roessler F, Royl G, Eckey T. Comparing outcome and recanalization results in patients with anterior circulation stroke following endovascular treatment with and without a treatment with rt-PA: A single-center study. *Brain and behavior* 2018; **8:** e00974.

[39]. Ferrigno M, Bricout N, Leys D*, et al.* Intravenous Recombinant Tissue-Type Plasminogen Activator: Influence on Outcome in Anterior Circulation Ischemic Stroke Treated by Mechanical Thrombectomy. *Stroke* 2018; **49:** 1377-1385.

[40]. Imbarrato G, Bentley J, Gordhan A. Clinical Outcomes of Endovascular Thrombectomy in Tissue Plasminogen Activator versus Non-Tissue Plasminogen Activator Patients at Primary Stroke Care Centers. *Journal of neurosciences in rural practice* 2018; **9:** 240-244.

[41]. Li C, Zhao W, Wu C*, et al.* Outcome of endovascular treatment for acute basilar artery occlusion in the modern era: a single institution experience. *Neuroradiology* 2018; **60:** 651-659.

[42]. Wang H, Zhang M, Hao Y*, et al.* Early Prediction of Poor Outcome Despite Successful Recanalization After Endovascular Treatment for Anterior Large Vessel Occlusion Stroke. *World neurosurgery* 2018; **115:** e312-e321.

[43]. Gory B, Haussen DC, Piotin M*, et al.* Impact of intravenous thrombolysis and emergent carotid stenting on reperfusion and clinical outcomes in patients with acute stroke with tandem lesion treated with thrombectomy: a collaborative pooled analysis. *European journal of neurology* 2018; **25:** 1115-1120.

[44]. Goyal N, Tsivgoulis G, Frei D*, et al.* Comparative safety and efficacy of combined IVT and MT with direct MT in large vessel occlusion. *Neurology* 2018; **90:** e1274-e1282.

[45]. Barral M, Lassalle L, Dargazanli C*, et al.* Predictors of favorable outcome after mechanical thrombectomy for anterior circulation acute ischemic stroke in octogenarians. *Journal of neuroradiology = Journal de neuroradiologie* 2018; **45:** 211-216.

[46]. Choi JH, Im SH, Lee KJ, Koo JS, Kim BS, Shin YS. Comparison of Outcomes After Mechanical Thrombectomy Alone or Combined with Intravenous Thrombolysis and Mechanical Thrombectomy for Patients with Acute Ischemic Stroke due to Large Vessel Occlusion. *World neurosurgery* 2018; **114:** e165-e172.

[47]. Gory B, Mazighi M, Labreuche J*, et al.* Predictors for Mortality after Mechanical Thrombectomy of Acute Basilar Artery Occlusion. *Cerebrovascular diseases (Basel, Switzerland)* 2018; **45:** 61-67.

[48]. Bourcier R, Alexandre P-L, Eugène F*, et al.* Is bridging therapy still required in stroke due to carotid artery terminus occlusions? *Journal of neurointerventional surgery* 2018; **10:** 625-628.

[49]. Chu H-J, Tang S-C, Lee C-W, Jeng J-S, Liu H-M. Endovascular thrombectomy for acute ischemic stroke: A single-center experience in Taiwan. *Journal of the Formosan Medical Association = Taiwan yi zhi* 2018; **117:** 806-813.

[50]. Manceau PF, Soize S, Gawlitza M*, et al.* Is there a benefit of mechanical thrombectomy in patients with large stroke (DWI-ASPECTS ≤ 5)? *European journal of neurology* 2018; **25:** 105-110.

[51]. Kaesmacher J, Kleine JF. Bridging Therapy with i. v. rtPA in MCA Occlusion Prior to Endovascular Thrombectomy: a Double-Edged Sword? *Clinical neuroradiology* 2018; **28:** 81-89.

[52]. Rai AT, Boo S, Buseman C*, et al.* Intravenous thrombolysis before endovascular therapy for large vessel strokes can lead to significantly higher hospital costs without improving outcomes. *Journal of neurointerventional surgery* 2018; **10:** 17-21.

[53]. Coutinho JM, Liebeskind DS, Slater L-A*, et al.* Combined Intravenous Thrombolysis and Thrombectomy vs Thrombectomy Alone for Acute Ischemic Stroke: A Pooled Analysis of the SWIFT and STAR Studies. *JAMA neurology* 2017; **74:** 268-274.

[54]. Bellwald S, Weber R, Dobrocky T*, et al.* Direct Mechanical Intervention Versus Bridging Therapy in Stroke Patients Eligible for Intravenous Thrombolysis: A Pooled Analysis of 2 Registries. *Stroke* 2017; **48:** 3282-3288.

[55]. Kim D-H, Nah H-W, Park H-S, Choi J-H, Kang M-J, Cha J-K. Factors associated with early dramatic recovery following successful recanalization of occluded artery by endovascular treatment in anterior circulation stroke. *Journal of clinical neuroscience : official journal of the Neurosurgical Society of Australasia* 2017; **46:** 171-175.

[56]. Uno J, Kameda K, Otsuji R*, et al.* Mechanical Thrombectomy for Acute Basilar Artery Occlusion in Early Therapeutic Time Window. *Cerebrovascular diseases (Basel, Switzerland)* 2017; **44:** 217-224.

[57]. Merlino G, Sponza M, Petralia B*, et al.* Short and long-term outcomes after combined intravenous thrombolysis and mechanical thrombectomy versus direct mechanical thrombectomy: a prospective single-center study. *Journal of thrombosis and thrombolysis* 2017; **44:** 203-209.

[58]. Wang H, Zi W, Hao Y*, et al.* Direct endovascular treatment: an alternative for bridging therapy in anterior circulation large-vessel occlusion stroke. *European journal of neurology* 2017; **24:** 935-943.

[59]. Park H-K, Chung J-W, Hong J-H*, et al.* Preceding Intravenous Thrombolysis in Patients Receiving Endovascular Therapy. *Cerebrovascular diseases (Basel, Switzerland)* 2017; **44:** 51-58.

[60]. Wee C-K, McAuliffe W, Phatouros CC*, et al.* Outcomes of Endovascular Thrombectomy with and without Thrombolysis for Acute Large Artery Ischaemic Stroke at a Tertiary Stroke Centre. *Cerebrovascular diseases extra* 2017; **7**.

[61]. Abilleira S, Ribera A, Cardona P*, et al.* Outcomes After Direct Thrombectomy or Combined Intravenous and Endovascular Treatment Are Not Different. *Stroke* 2017; **48:** 375-378.

[62]. Maier IL, Behme D, Schnieder M*, et al.* Bridging-therapy with intravenous recombinant tissue plasminogen activator improves functional outcome in patients with endovascular treatment in acute stroke. *Journal of the neurological sciences* 2017; **372:** 300-304.

[63]. Mundiyanapurath S, Tillmann A, Möhlenbruch MA, Bendszus M, Ringleb PA. Endovascular stroke therapy may be safe in patients with elevated international normalized ratio. *Journal of neurointerventional surgery* 2017; **9:** 1187-1190.

[64]. Alonso de Leciñana M, Martínez-Sánchez P, García-Pastor A*, et al.* Mechanical thrombectomy in patients with medical contraindications for intravenous thrombolysis: a prospective observational study. *Journal of neurointerventional surgery* 2017; **9:** 1041-1046.

[65]. Weber R, Nordmeyer H, Hadisurya J*, et al.* Comparison of outcome and interventional complication rate in patients with acute stroke treated with mechanical thrombectomy with and without bridging thrombolysis. *Journal of neurointerventional surgery* 2017; **9:** 229-233.

[66]. Mistry EA, Mistry AM, Nakawah MO*, et al.* Systolic Blood Pressure Within 24 Hours After Thrombectomy for Acute Ischemic Stroke Correlates With Outcome. *Journal of the American Heart Association* 2017; **6**.

[67]. Nogueira RG, Zaidat OO, Castonguay AC*, et al.* Rescue Thrombectomy in Large Vessel Occlusion Strokes Leads to Better Outcomes than Intravenous Thrombolysis Alone: A 'Real World' Applicability of the Recent Trials. *Interventional neurology* 2016; **5:** 101-110.

[68]. Angermaier A, Michel P, Khaw AV, Kirsch M, Kessler C, Langner S. Intravenous Thrombolysis and Passes of Thrombectomy as Predictors for Endovascular Revascularization in Ischemic Stroke. *Journal of stroke and cerebrovascular diseases : the official journal of National Stroke Association* 2016; **25:** 2488-2495.

[69]. Rebello LC, Haussen DC, Grossberg JA*, et al.* Early Endovascular Treatment in Intravenous Tissue Plasminogen Activator-Ineligible Patients. *Stroke* 2016; **47:** 1131-1134.

[70]. Broeg-Morvay A, Mordasini P, Bernasconi C*, et al.* Direct Mechanical Intervention Versus Combined Intravenous and Mechanical Intervention in Large Artery Anterior Circulation Stroke: A Matched-Pairs Analysis. *Stroke* 2016; **47:** 1037-1044.

[71]. Behme D, Kabbasch C, Kowoll A*, et al.* Intravenous Thrombolysis Facilitates Successful Recanalization with Stent-Retriever Mechanical Thrombectomy in Middle Cerebral Artery Occlusions. *Journal of stroke and cerebrovascular diseases : the official journal of National Stroke Association* 2016; **25:** 954-959.

[72]. Villwock MR, Padalino DJ, Deshaies EM. Trends in mortality following mechanical thrombectomy for the treatment of acute ischemic stroke in the USA. *Journal of neurointerventional surgery* 2016; **8:** 457-460.

[73]. Minnerup J, Wersching H, Teuber A*, et al.* Outcome After Thrombectomy and Intravenous Thrombolysis in Patients With Acute Ischemic Stroke: A Prospective Observational Study. *Stroke* 2016; **47:** 1584-1592.

[74]. Mulder MJ, Berkhemer OA, Fransen PS*, et al.* Treatment in patients who are not eligible for intravenous alteplase: MR CLEAN subgroup analysis. *International journal of stroke : official journal of the International Stroke Society* 2016; **11:** 637-645.

[75]. Wang DT, Churilov L, Dowling R, Mitchell P, Yan B. Successful recanalization post endovascular therapy is associated with a decreased risk of intracranial haemorrhage: a retrospective study. *BMC neurology* 2015; **15:** 185.

[76]. Bourcier R, Volpi S, Guyomarch B*, et al.* Susceptibility Vessel Sign on MRI Predicts Favorable Clinical Outcome in Patients with Anterior Circulation Acute Stroke Treated with Mechanical Thrombectomy. *AJNR American journal of neuroradiology* 2015; **36:** 2346-2353.

[77]. Saposnik G, Lebovic G, Demchuk A*, et al.* Added Benefit of Stent Retriever Technology for Acute Ischemic Stroke: A Pooled Analysis of the NINDS tPA, SWIFT, and STAR Trials. *Neurosurgery* 2015; **77:** 454-461.

[78]. Goyal M, Demchuk AM, Menon BK*, et al.* Randomized assessment of rapid endovascular treatment of ischemic stroke. *The New England journal of medicine* 2015; **372:** 1019-1030.

[79]. Jeromel M, Miloševicˇ Z, Zaletel M, Žvan B, Švigelj V, Oblak JP. Endovascular therapy for acute stroke is a safe and efficient evolving method: a single-center retrospective analysis. *Journal of vascular and interventional radiology : JVIR* 2015; **26:** 1025-1030.

[80]. Guedin P, Larcher A, Decroix J-P*, et al.* Prior IV Thrombolysis Facilitates Mechanical Thrombectomy in Acute Ischemic Stroke. *Journal of stroke and cerebrovascular diseases : the official journal of National Stroke Association* 2015; **24:** 952-957.

[81]. Fujimoto M, Salamon N, Takemoto K*, et al.* Correlation of clot imaging with endovascular recanalization in internal carotid artery terminus occlusion. *Journal of neurointerventional surgery* 2015; **7:** 131-134.

[82]. Nogueira RG, Gupta R, Jovin TG*, et al.* Predictors and clinical relevance of hemorrhagic transformation after endovascular therapy for anterior circulation large vessel occlusion strokes: a multicenter retrospective analysis of 1122 patients. *Journal of neurointerventional surgery* 2015; **7:** 16-21.

[83]. Jankowitz B, Grandhi R, Horev A*, et al.* Primary manual aspiration thrombectomy (MAT) for acute ischemic stroke: safety, feasibility and outcomes in 112 consecutive patients. *Journal of neurointerventional surgery* 2015; **7:** 27-31.

[84]. Jovin TG, Chamorro A, Cobo E*, et al.* Thrombectomy within 8 hours after symptom onset in ischemic stroke. *The New England journal of medicine* 2015; **372:** 2296-2306.

[85]. Leker RR, Pikis S, Gomori JM, Cohen JE. Is Bridging Necessary? A Pilot Study of Bridging versus Primary Stentriever-Based Endovascular Reperfusion in Large Anterior Circulation Strokes. *Journal of stroke and cerebrovascular diseases : the official journal of National Stroke Association* 2015; **24:** 1163-1167.

[86]. Brinjikji W, Rabinstein AA, Cloft HJ. Outcomes of endovascular mechanical thrombectomy and intravenous tissue plasminogen activator for the treatment of vertebrobasilar stroke. *Journal of clinical neurology (Seoul, Korea)* 2014; **10:** 17-23.

[87]. Spiotta AM, Vargas J, Turner R, Chaudry MI, Battenhouse H, Turk AS. The golden hour of stroke intervention: effect of thrombectomy procedural time in acute ischemic stroke on outcome. *Journal of neurointerventional surgery* 2014; **6:** 511-516.

[88]. Mourand I, Machi P, Milhaud D*, et al.* Mechanical thrombectomy with the Solitaire device in acute basilar artery occlusion. *Journal of neurointerventional surgery* 2014; **6:** 200-204.

[89]. Kass-Hout T, Kass-Hout O, Mokin M*, et al.* Is bridging with intravenous thrombolysis of any benefit in endovascular therapy for acute ischemic stroke? *World neurosurgery* 2014; **82:** e453-e458.

[90]. Jankowitz B, Aghaebrahim A, Zirra A*, et al.* Manual aspiration thrombectomy: adjunctive endovascular recanalization technique in acute stroke interventions. *Stroke* 2012; **43:** 1408-1411.

[91]. Dávalos A, Pereira VM, Chapot R, Bonafé A, Andersson T, Gralla J. Retrospective multicenter study of Solitaire FR for revascularization in the treatment of acute ischemic stroke. *Stroke* 2012; **43:** 2699-2705.

[92]. Pfefferkorn T, Holtmannspötter M, Patzig M*, et al.* Preceding intravenous thrombolysis facilitates endovascular mechanical recanalization in large intracranial artery occlusion. *International journal of stroke : official journal of the International Stroke Society* 2012; **7:** 14-18.

[93]. Bang OY, Saver JL, Kim SJ*, et al.* Collateral Flow Averts Hemorrhagic Transformation After Endovascular Therapy for Acute Ischemic Stroke. *Stroke* 2011; **42:** 2235-U2329.
